# Supplementary material for: YTHDC1 delays cellular senescence and pulmonary fibrosis by activating ATR in an m6A-independent manner
Source: EMBO J. 2023 Dec 15;43(1):4. doi: 10.1038/s44318-023-00003-2 (PMC10883269; doi:10.1038/s44318-023-00003-2)
Supplement: Supplementary file 1 — Appendix [file 44318_2023_3_MOESM1_ESM.pdf]

Appendix

Appendix Figure S1 p. 2

Appendix Figure S2 p. 3

Appendix Figure S1

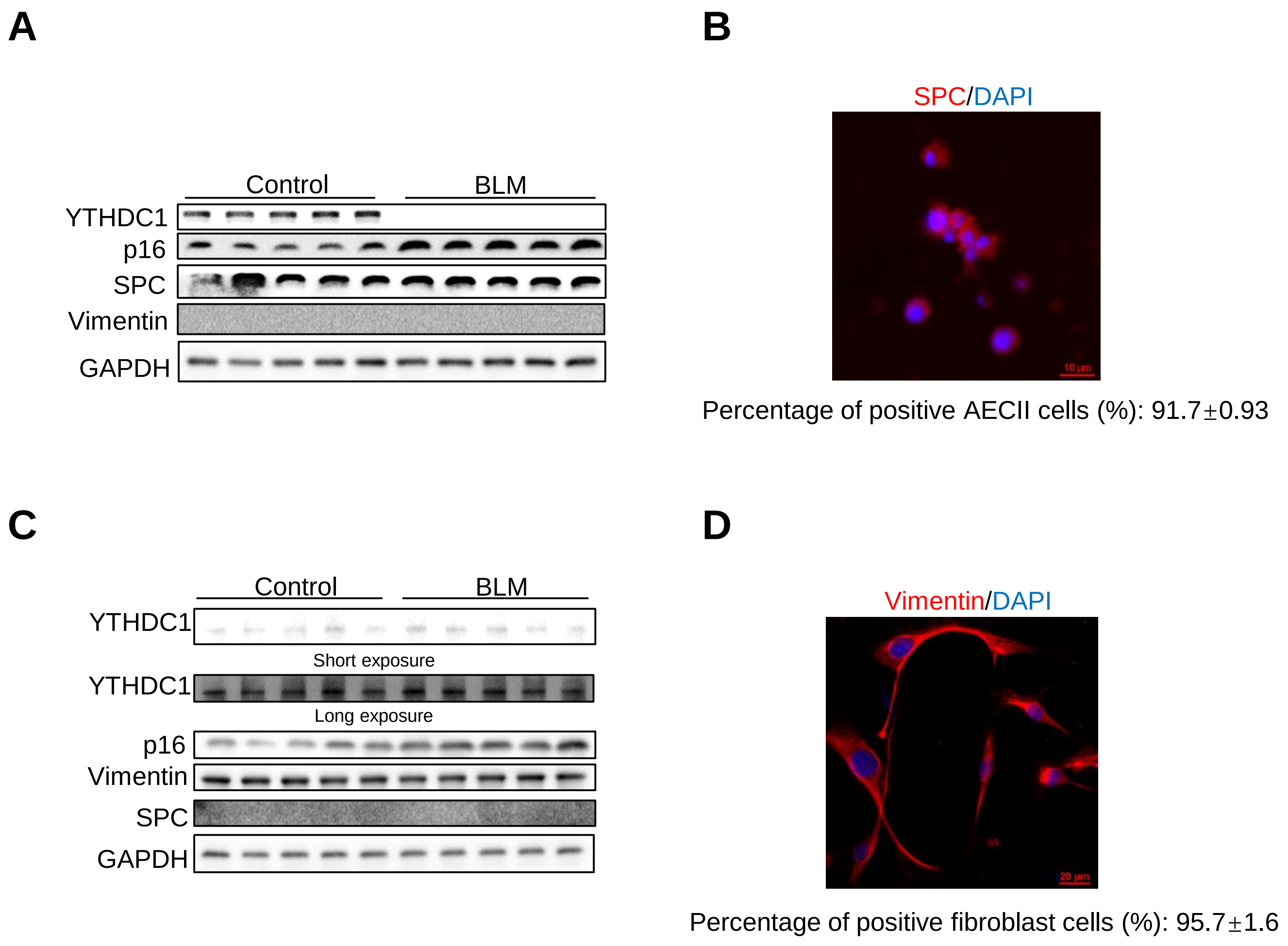

Appendix Figure S1. The expression level of YTHDC1 decreased mainly in AECII upon BLM treatment.

(A) Immunoblot analysis of YTHDC1, p16, SPC (the marker of AECII cells) and Vimentin (the marker of fibroblast) in primary AECII cells from mice lung treated with BLM or saline (n=5per group).

(B) Up: IF detection of SPC in AECII cell from panel (A). Scale bar: 10μm. Down: Quantification of the percentage of SPC positive cells ((n=5 per group).).

(C) Immunoblot analysis of YTHDC1, p16, SPC and Vimentin in fibroblast from mice lung treated with BLM or saline (n=5per group)..

(D) Up: IF detection of Vimentin in fibroblast from panel (C). Scale bar: 20μm. Down: Quantification of the percentage of Vimentin positive cells (n=5per group).

Appendix Figure S2

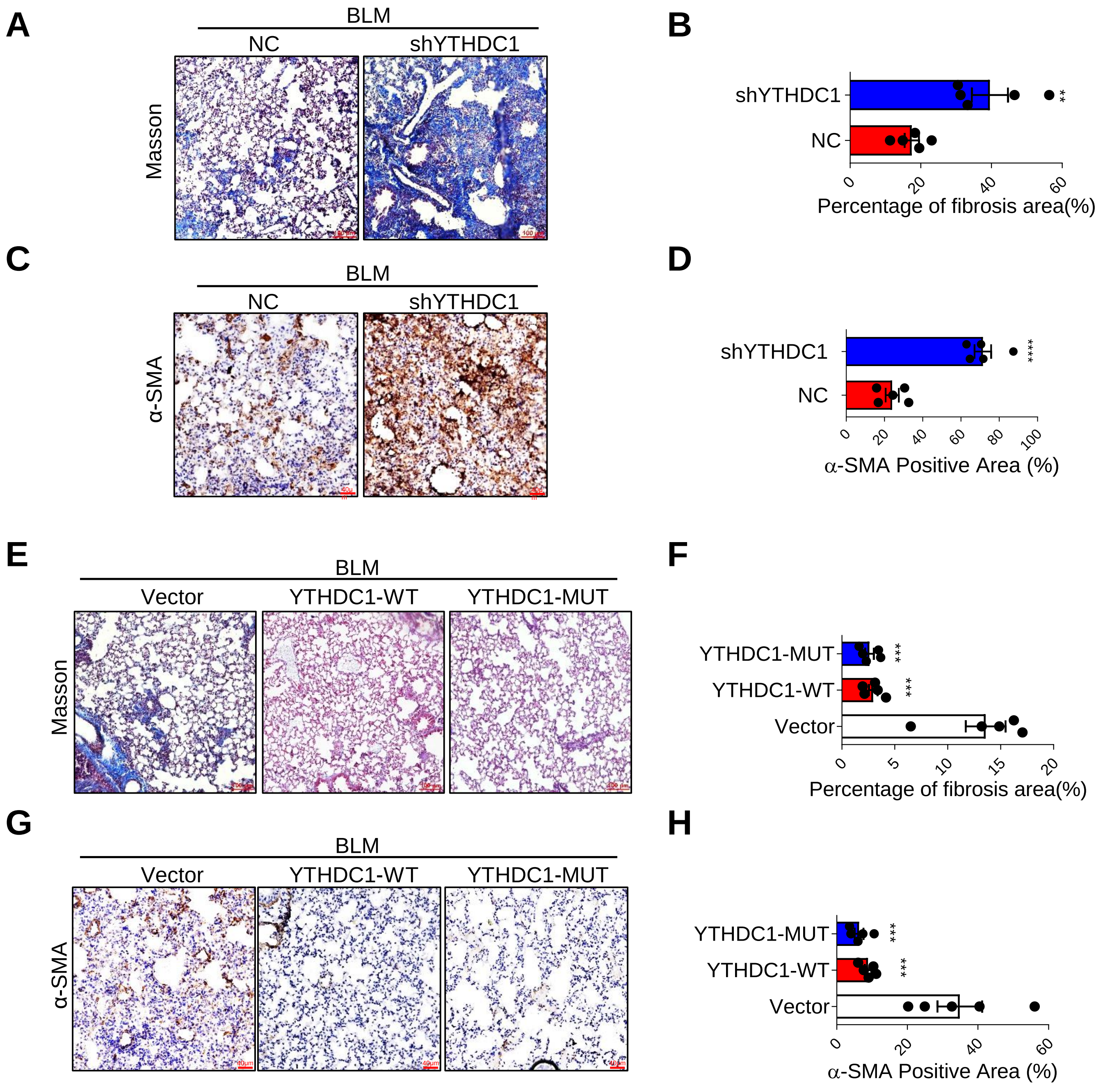

Appendix Figure S2. YTHDC1 regulates bleomycin induced pulmonary fibrosis.

(A) Masson's trichrome was performed to determine the level of fibrosis from the mice lung tissues. C57/BL6 mice transfected with indicated shAAV vectors were treated with BLM for 14 days. (n≥5per group). Scale bar: 100μm.

(B) Quantification of panel (A). The percentage of fibrosis area (blue) was calculated.

(C) Representative images of α-SMA in the mice lungs from panel (A) (n≥5 per group). Scale bar: 40μm.

(D) Quantification of panel (C) . The percentage of α-SMA positive area was calculated.

(E-H) as in panels (A-D), except the mice lungs overexpressed with YTHDC1-WT, YTHDC1-MUT or Vector. (n≥5 per group).
