# Supplementary material for: YTHDC1 delays cellular senescence and pulmonary fibrosis by activating ATR in an m6A-independent manner
Source: EMBO J. 2023 Dec 15;43(1):4. doi: 10.1038/s44318-023-00003-2 (PMC10883269; doi:10.1038/s44318-023-00003-2)
Supplement: Supplementary file 3 — Source Data EV Table 1 [file 44318_2023_3_MOESM3_ESM.docx]

| **Table EV1 Sequences of oligonucleotides** |  |
| --- | --- |
| NAME | SEQUENCE |
| siYTHDC1-1 (Human and Rat) | GUCGACCAGAAGAUUAUGAUA |
| siYTHDC1-2 | AUCGAGUAUGCAAAUAUUGAA |
| siRAD9A | GAAGAACAGUGAGCGGAAC |
| siRAD17 | GACAAAGUAUAACAAGUUA |
| siMETTL3-1 | CUGCAAGUAUGUUCACUAUGA |
| siMETTL3-2 | CCGCGUGAGAAUUGGCUAU |
| siMETTL14-1 | GCUAAAGGAUGAGUUAAU |
| siMETTL14-2 | GCAUUGGUGCCGUGUUAAA |
| siWTAP-1 | CACAGAUCUUAACUCUAAU |
| siWTAP-2 | GGGAAAACAUCCUUGUAA |
| siFTO-1 | GGACCUGGUUAGGAUCCAA |
| siFTO-2 | GAGCUGGCAUCAUGAUGAA |
| siALKBH5-1 | ACAAGUACUUCUUCGGCG |
| siALKBH5-2 | GCGCCGUCAUCAACGACUA |
| siYTHDF2-1 | CGGUCCAUUAAUAACUAUAAC |
| siYTHDF2-2 | GCUACUCUGAGGACGAUAUU |
| siIGF2BP1 | AAGCUGAAUGGCCACCAGUUG |
| siIGF2BP2 | GCGAAAGGAUGGUCAUCAU |
| siIGF2BP3 | AAUCGAUGUCCACCAGUAAAGA |
| siMRE11 | CAGUCAACUUUGGUUUUAGUAAG |
| siATR | GCCGCUAAUCUUCUAACAU |
| siTopBP1 (Rat) | CAUUAUCUGUGUGACUGGCUU |
| shYTHDC1 | GATCCGACAGGAAATTGAACTTGATTCAAGAGATCAAGTTCAATTTCCTGTCTTTTTTC |
| GAPDH-F | GGAGCGAGATCCCTCCAAAAT |
| GAPDH-R | GGCTGTTGTCATACTTCTCATGG |
| METTL14-F | GAACACAGAGCTTAAATCCCCA |
| METTL14-R | TGTCAGCTAAACCTACATCCCTG |
| WTAP-F | CTTCCCAAGAAGGTTCGATTGA |
| WTAP-R | TCAGACTCTCTTAGGCCAGTTAC |
| FTO-F | ACTTGGCTCCCTTATCTGACC |
| FTO-R | TGTGCAGTGTGAGAAAGGCTT |
| ALKBH5-F | CGGCGAAGGCTACACTTACG |
| ALKBH5-R R | CCACCAGCTTTTGGATCACCA |
| YTHDF2-F | CCTTAGGTGGAGCCATGATTG |
| YTHDF2-R | TCTGTGCTACCCAACTTCAGT |
| IGF2BP1-F | GCGGCCAGTTCTTGGTCAA |
| IGF2BP1-R | TTGGGCACCGAATGTTCAATC |
| IGF2BP2-F | AGTGGAATTGCATGGGAAAATCA |
| IGF2BP2-R | CAACGGCGGTTTCTGTGTC |
| IGF2BP3-F | TATATCGGAAACCTCAGCGAGA |
| IGF2BP3-R | GGACCGAGTGCTCAACTTCT |
| GAPDH-mice-F | AGGTCGGTGTGAACGGATTTG |
| GAPDH-mice-R | TGTAGACCATGTAGTTGAGGTCA |
| YTHDC1-mice-F | GAGAATGGAGTCTACTGACACCA |
| YTHDC1-mice-R | ACAGACGAATTTTTCGATCAGCA |
| TGF-β-mice-F | CCGAATGTCTGACGTATTGAAGA |
| TGF-β-mice-R | GCGGACTACTATGCTAAAGAGG |
| IL6-mice-F | TAGTCCTTCCTACCCCAATTTCC |
| IL6-mice-R | TTGGTCCTTAGCCACTCCTTC |
| IL1α-mice-F | CGAAGACTACAGTTCTGCCATT |
| IL1α-mice-R | GACGTTTCAGAGGTTCTCAGAG |
| IL1β-mice-F | GCAACTGTTCCTGAACTCAACT |
| IL1β-mice-R | ATCTTTTGGGGTCCGTCAACT |
| CCL2-mice-F | TAAAAACCTGGATCGGAACCAAA |
| CCL2-mice-R | GCATTAGCTTCAGATTTACGGGT |
