# Supplementary figures and images for: YTHDC1 delays cellular senescence and pulmonary fibrosis by activating ATR in an m6A-independent manner (part 1 of 4)
Source: EMBO J. 2023 Dec 15;43(1):4. doi: 10.1038/s44318-023-00003-2 (PMC10883269; doi:10.1038/s44318-023-00003-2)

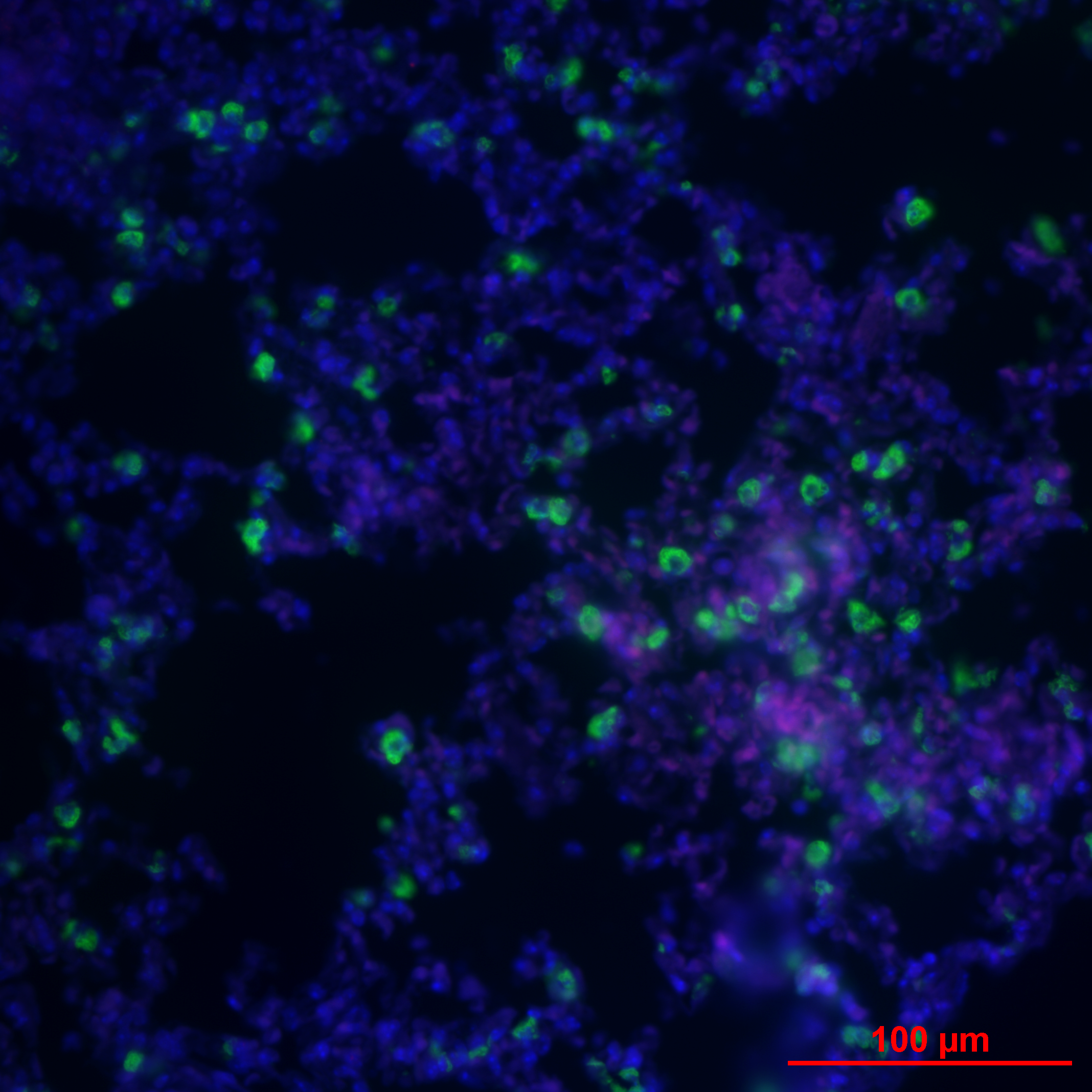

Supplement: Supplementary file 4 — Source Data Fig. 1 [file 44318_2023_3_MOESM4_ESM.zip › Figure1/1a-b/BLM YTHDC1 and SPC.tif]

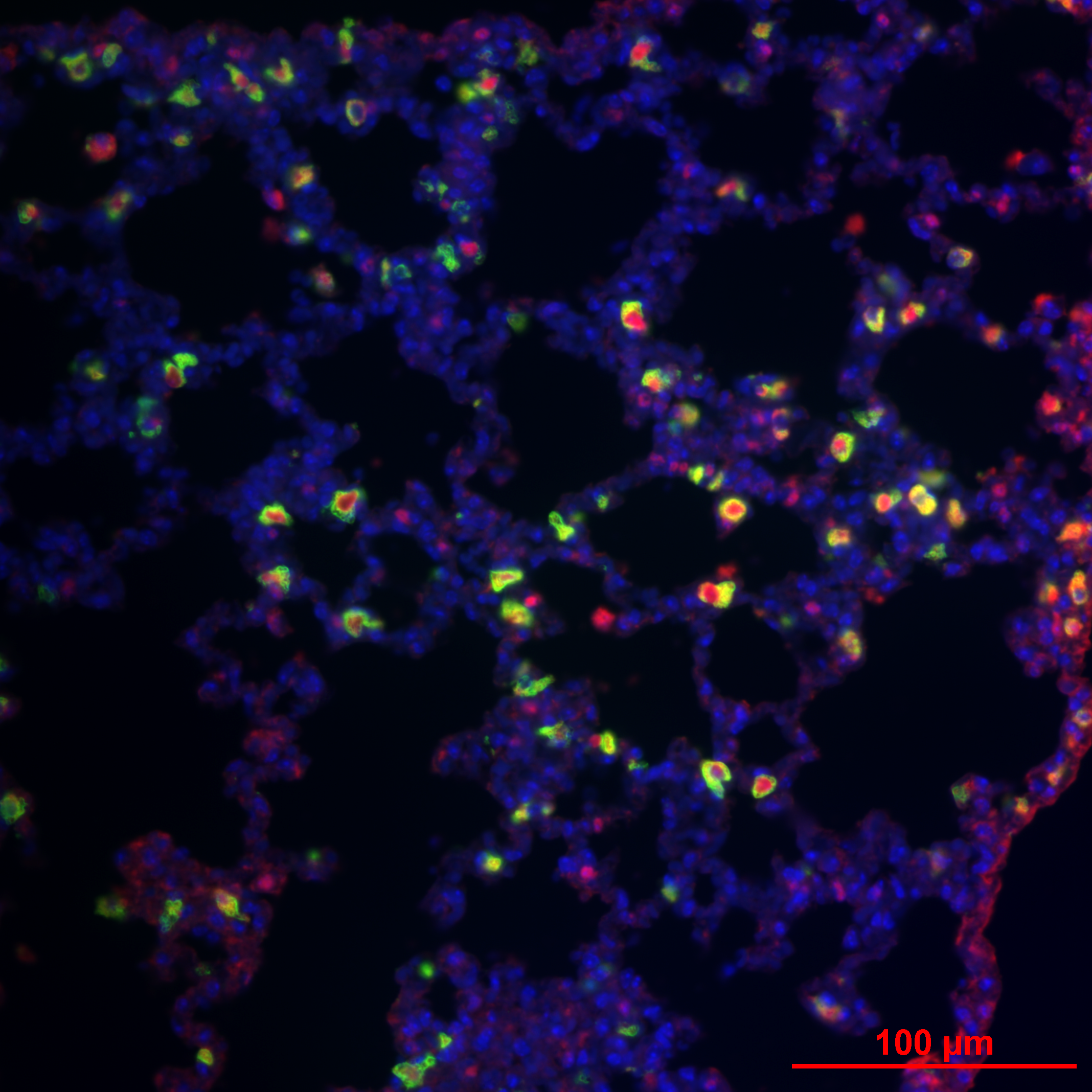

Supplement: Supplementary file 4 — Source Data Fig. 1 [file 44318_2023_3_MOESM4_ESM.zip › Figure1/1a-b/Saline YTHDC1 and SPC.tif]

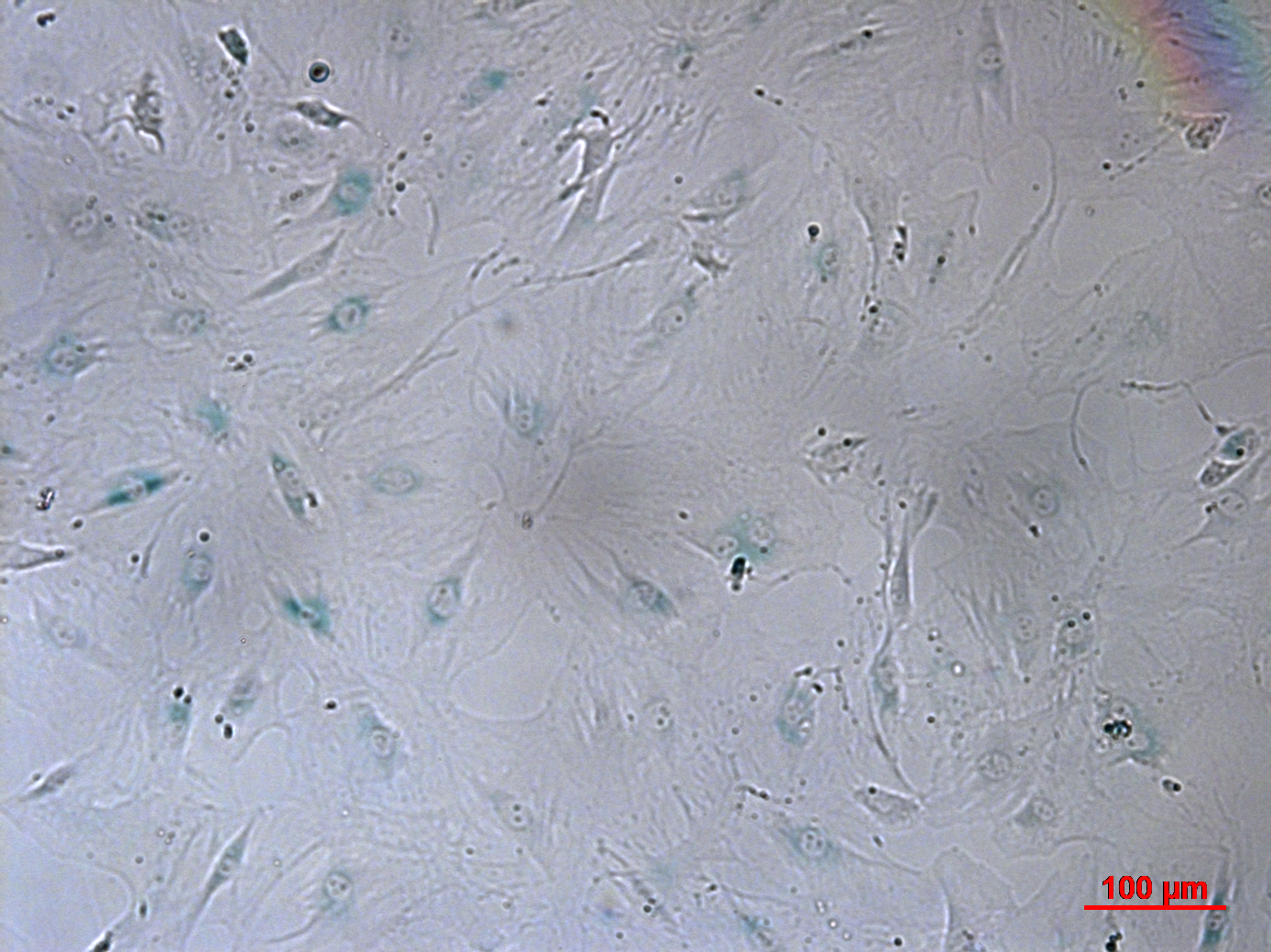

Supplement: Supplementary file 4 — Source Data Fig. 1 [file 44318_2023_3_MOESM4_ESM.zip › Figure1/1d-e/nc SA-β-gal/nc SA-β-gal.TIF]

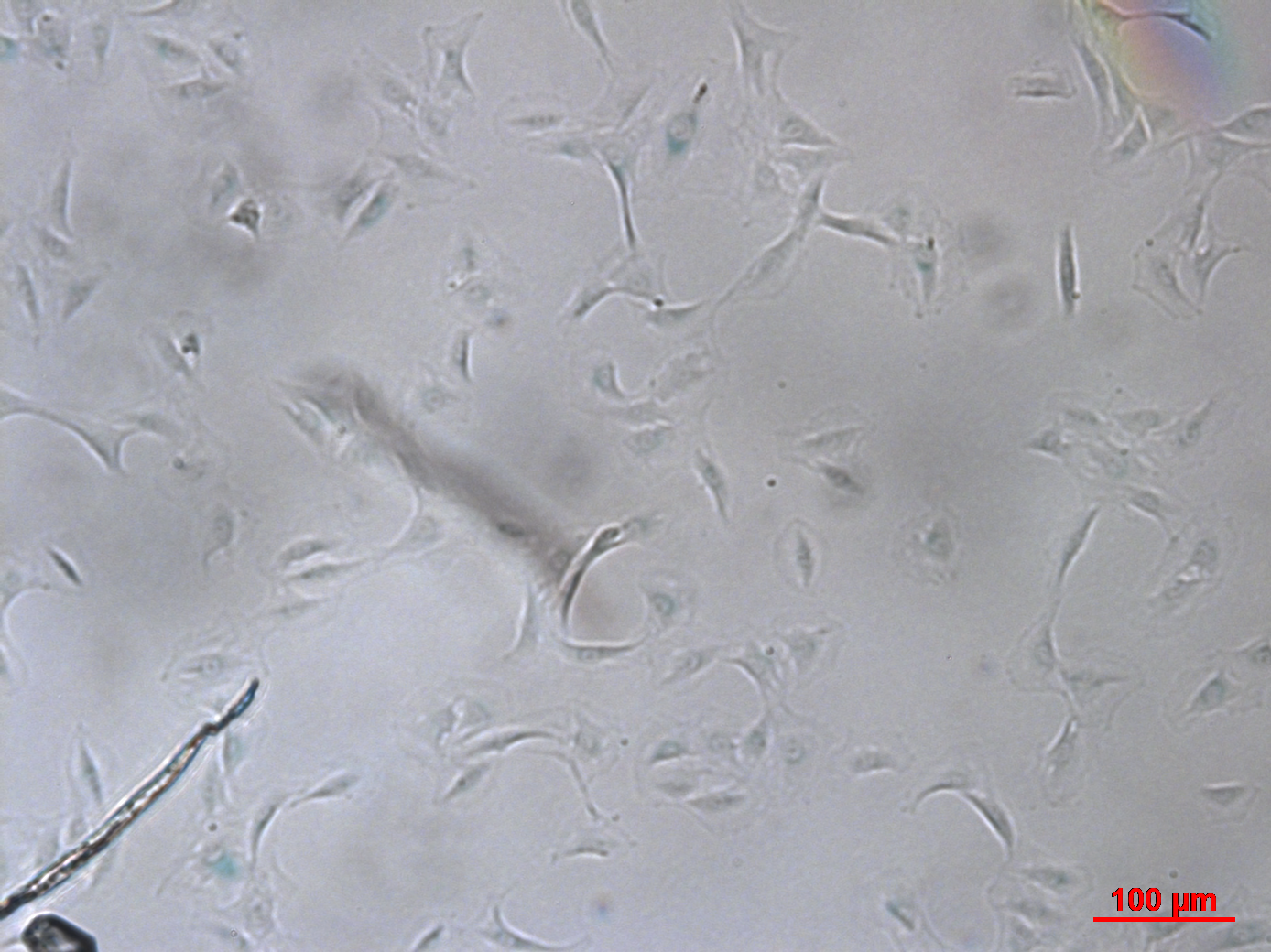

Supplement: Supplementary file 4 — Source Data Fig. 1 [file 44318_2023_3_MOESM4_ESM.zip › Figure1/1d-e/saline SA-β-gal/saline SA-β-gal.TIF]

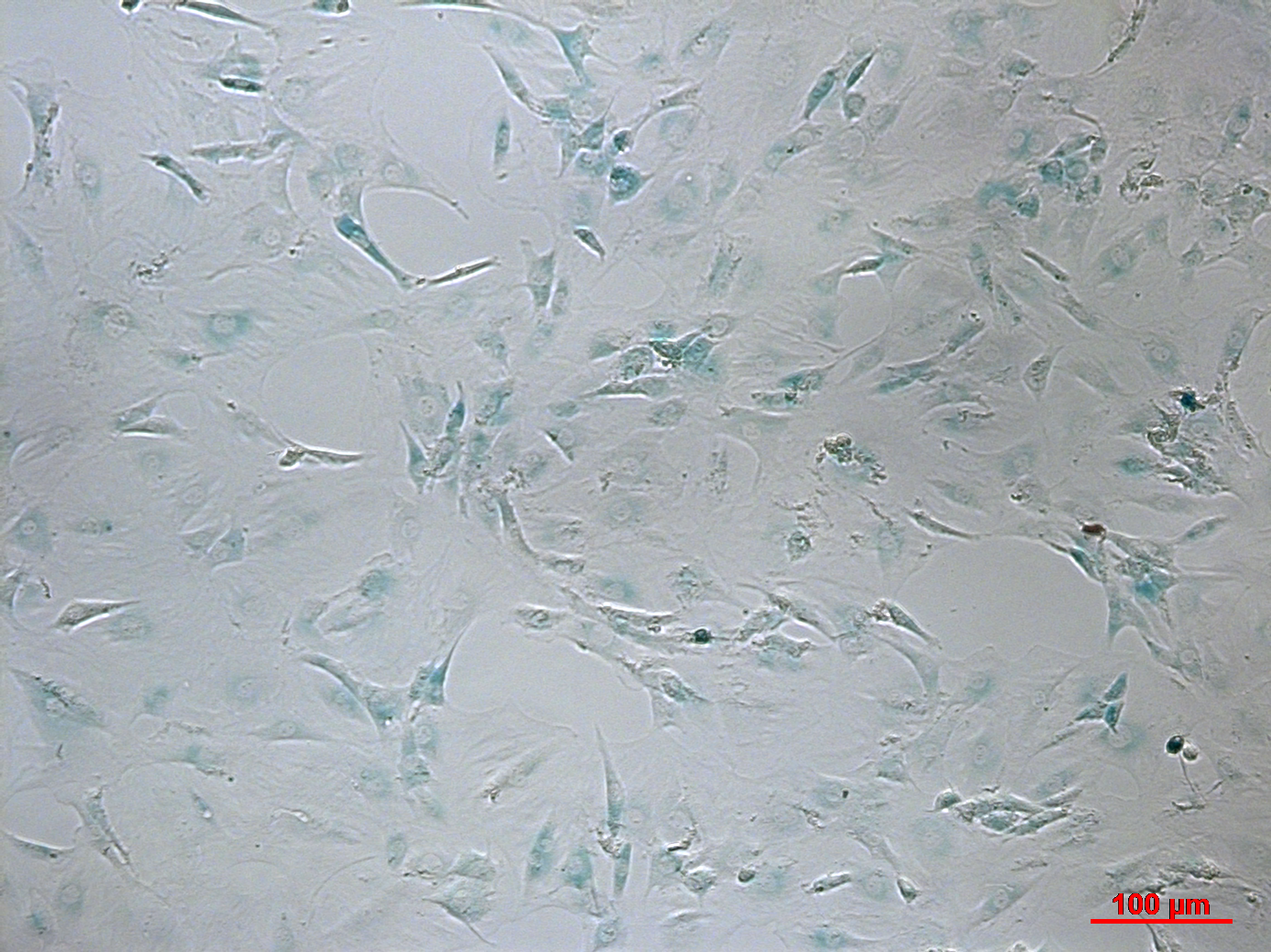

Supplement: Supplementary file 4 — Source Data Fig. 1 [file 44318_2023_3_MOESM4_ESM.zip › Figure1/1d-e/siYTHDC1 SA-β-gal/siYTHDC1 SA-β-gal.TIF]

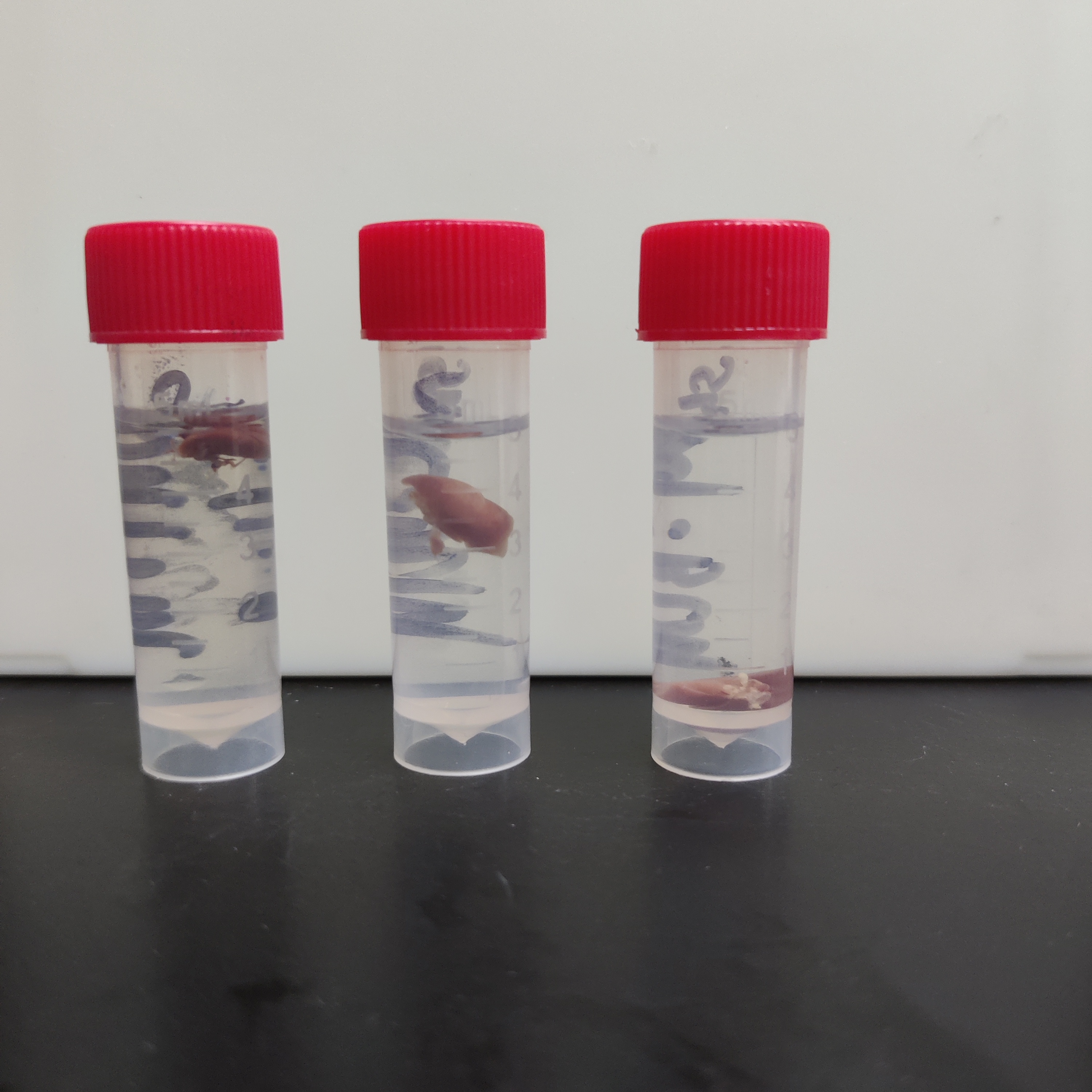

Supplement: Supplementary file 4 — Source Data Fig. 1 [file 44318_2023_3_MOESM4_ESM.zip › Figure1/1g-h/lung in water .jpg]

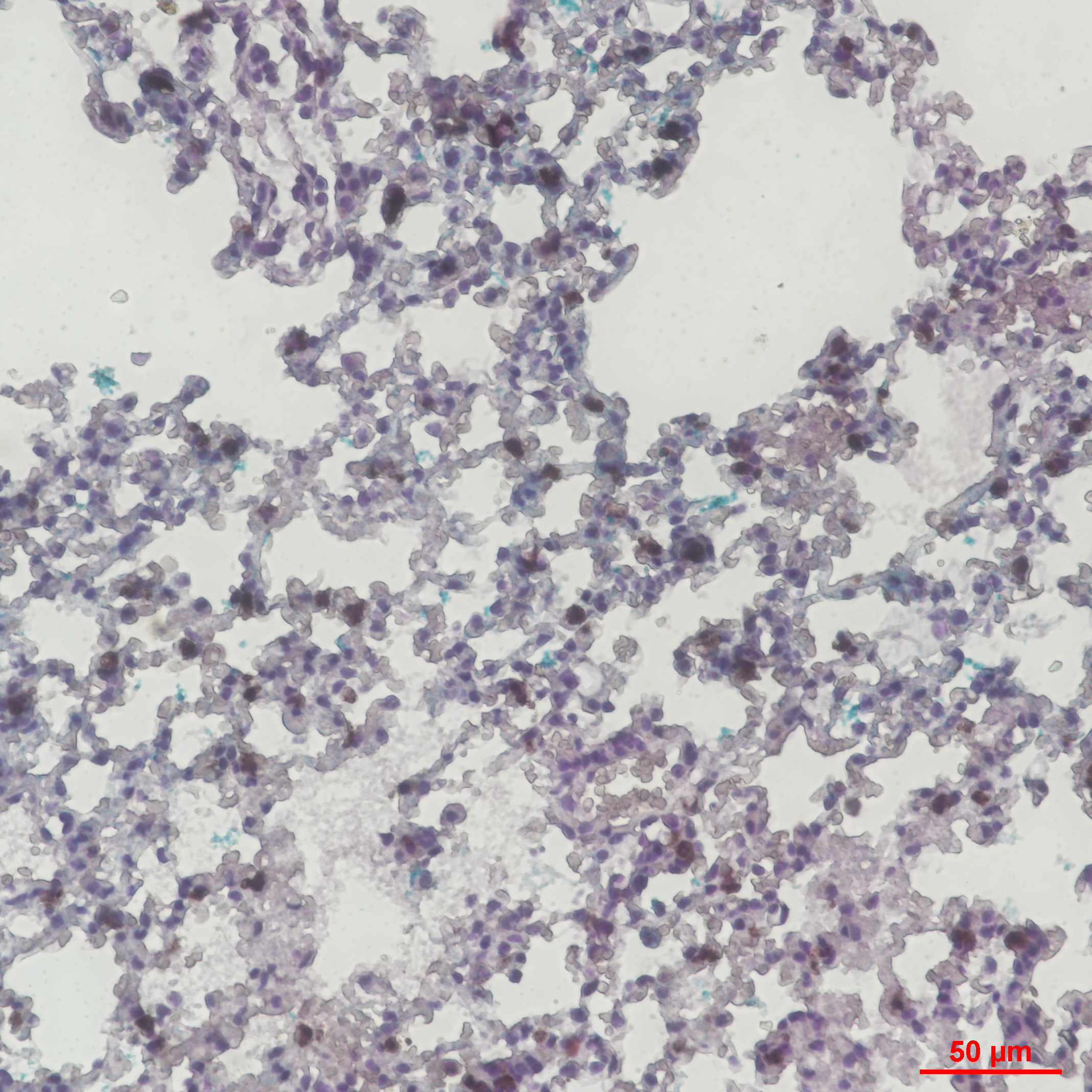

Supplement: Supplementary file 4 — Source Data Fig. 1 [file 44318_2023_3_MOESM4_ESM.zip › Figure1/1j-k/nc SPC and SA-β-gal.tif]

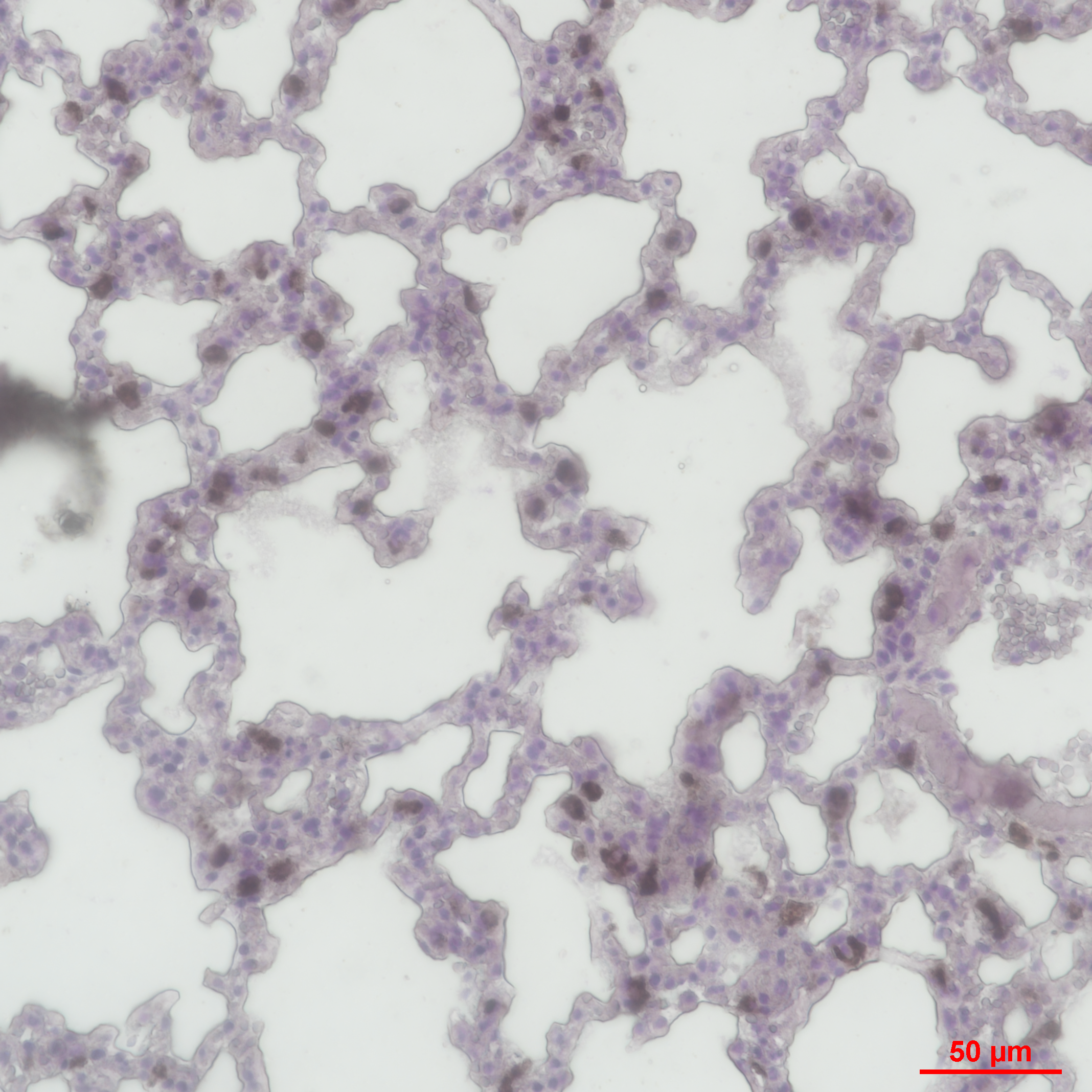

Supplement: Supplementary file 4 — Source Data Fig. 1 [file 44318_2023_3_MOESM4_ESM.zip › Figure1/1j-k/saline SPC and SA-β-gal.tif]

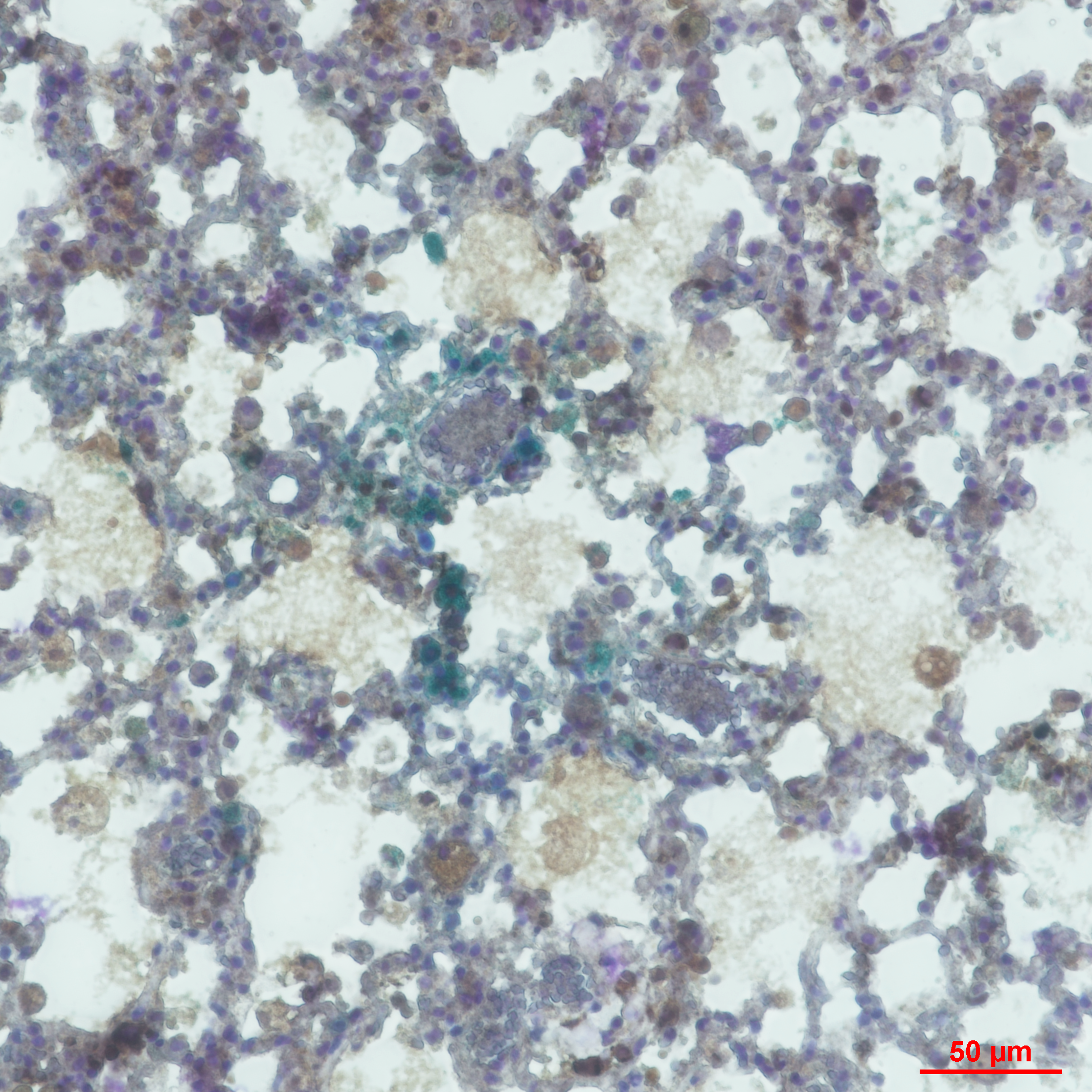

Supplement: Supplementary file 4 — Source Data Fig. 1 [file 44318_2023_3_MOESM4_ESM.zip › Figure1/1j-k/shYTHDC1 SPC and SA-β-gal.tif]

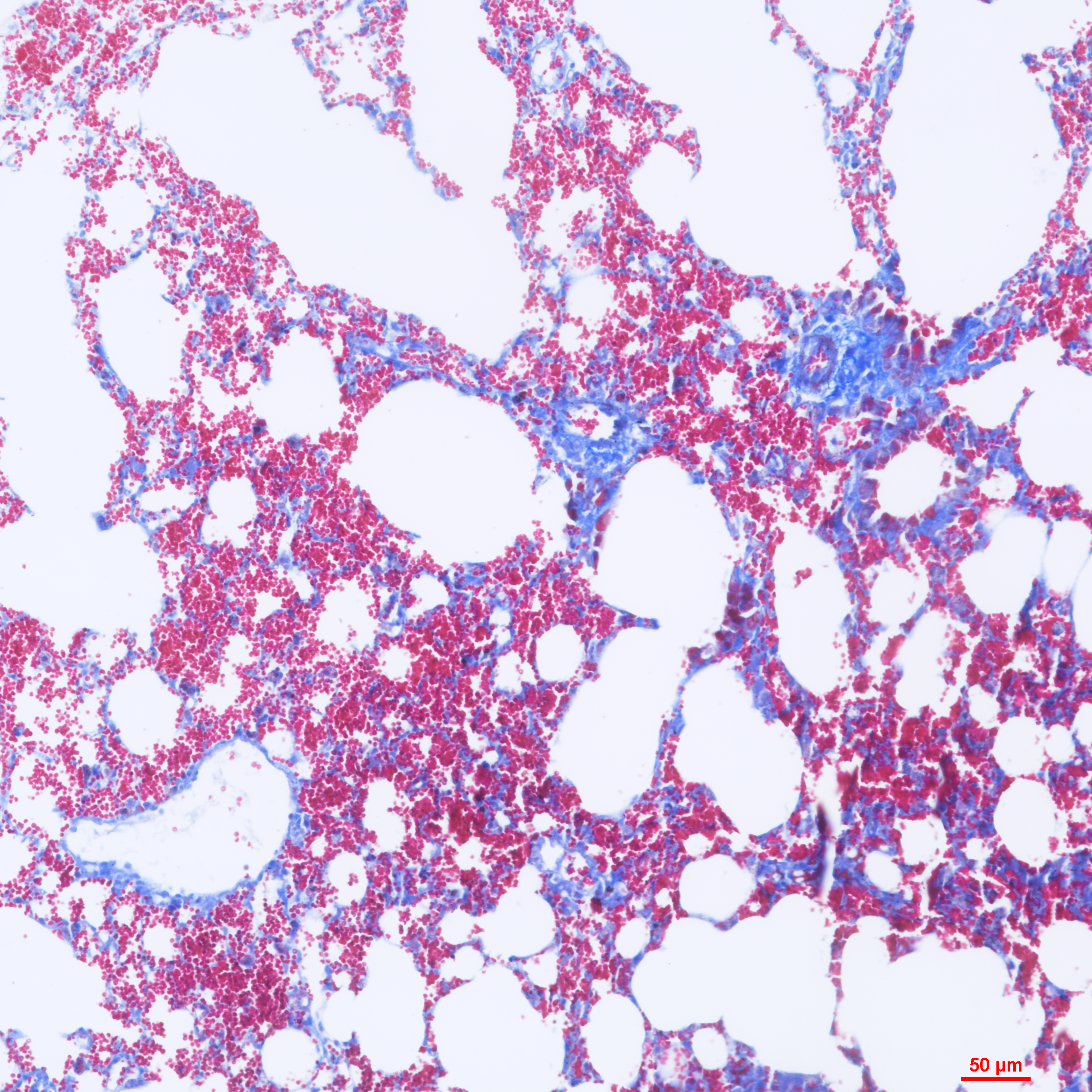

Supplement: Supplementary file 4 — Source Data Fig. 1 [file 44318_2023_3_MOESM4_ESM.zip › Figure1/1l-m/nc-BLM masson.tif]

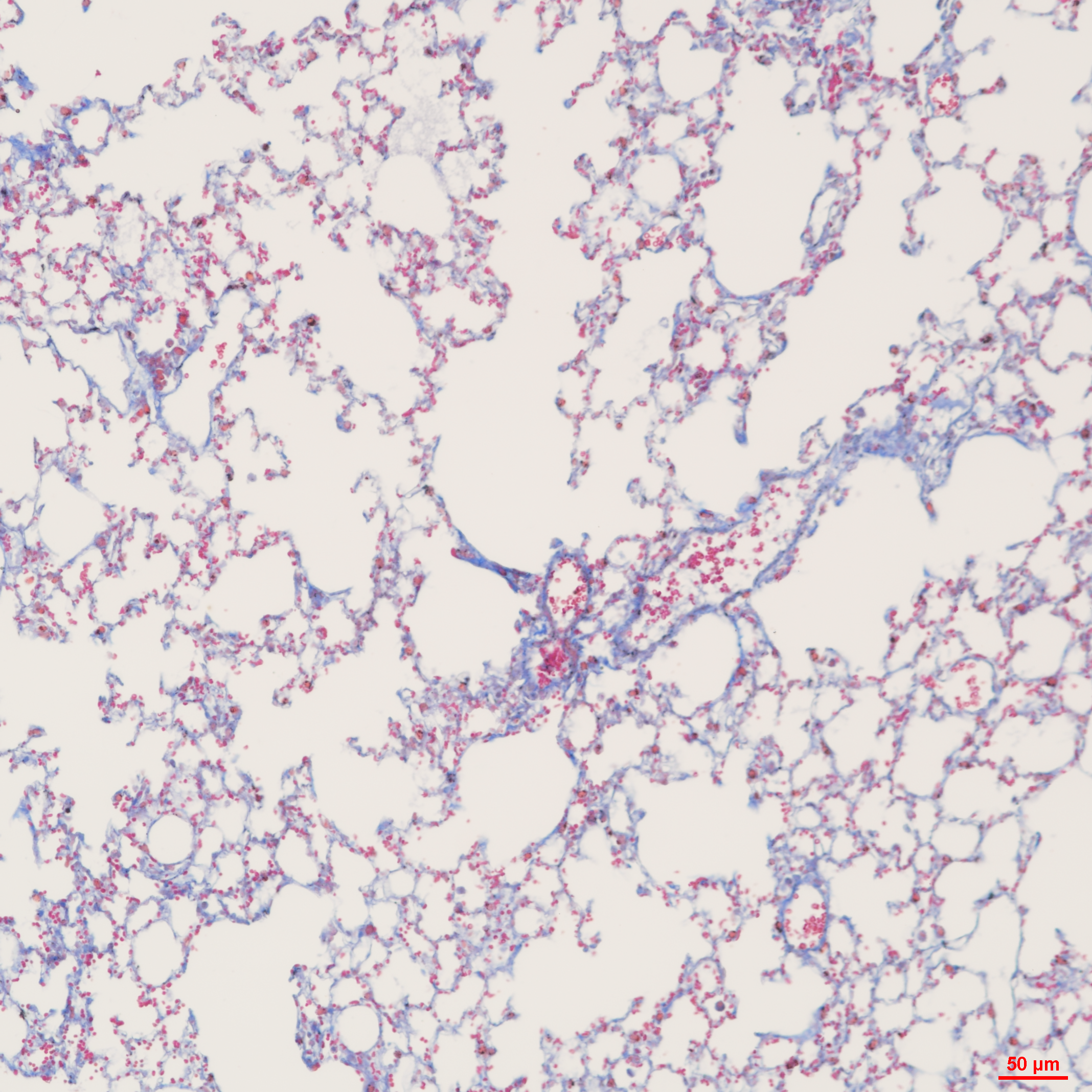

Supplement: Supplementary file 4 — Source Data Fig. 1 [file 44318_2023_3_MOESM4_ESM.zip › Figure1/1l-m/saline masson.tif]

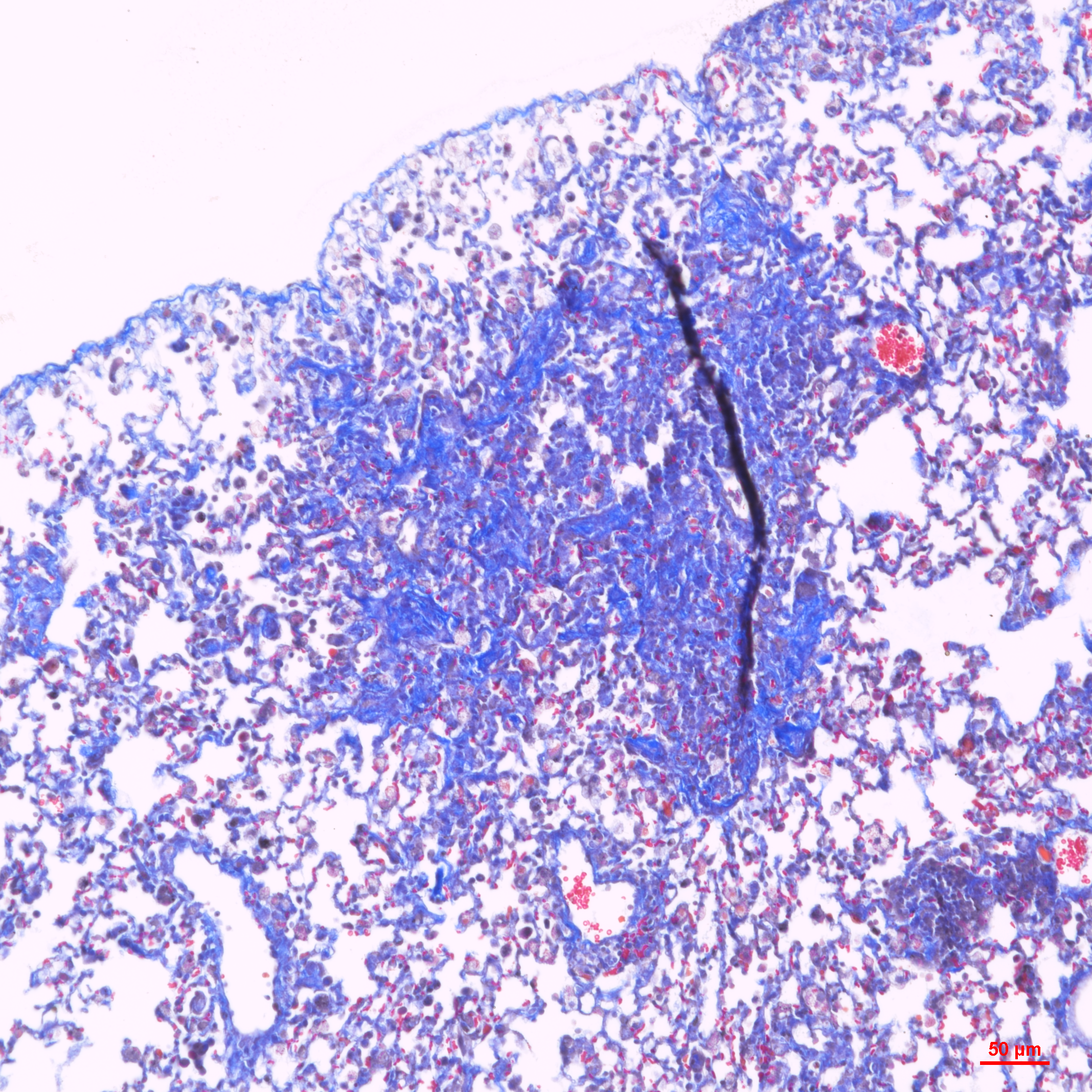

Supplement: Supplementary file 4 — Source Data Fig. 1 [file 44318_2023_3_MOESM4_ESM.zip › Figure1/1l-m/shYTHDC1-BLM masson.tif]

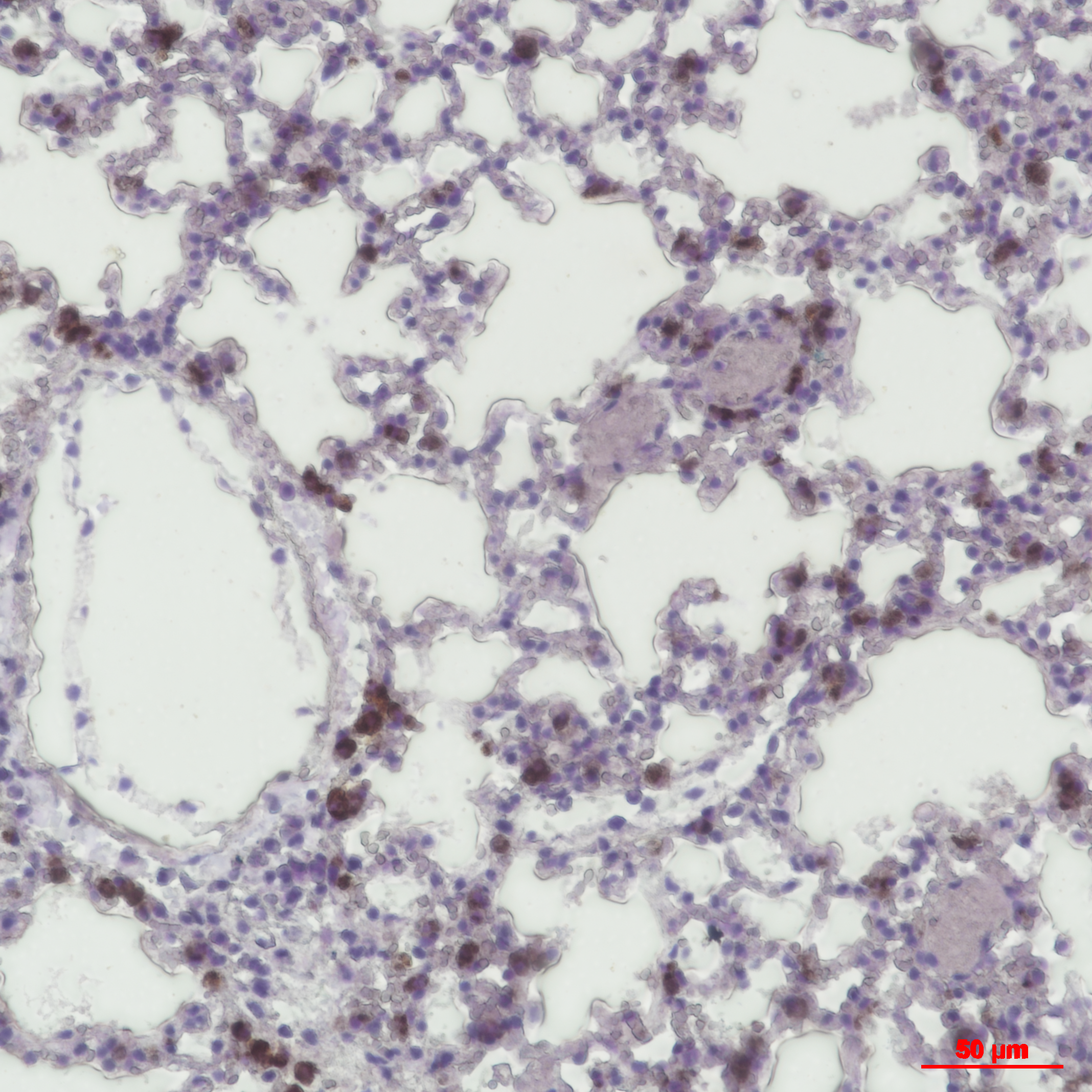

Supplement: Supplementary file 4 — Source Data Fig. 1 [file 44318_2023_3_MOESM4_ESM.zip › Figure1/1n-o/nc-blm yh2ax.tif]

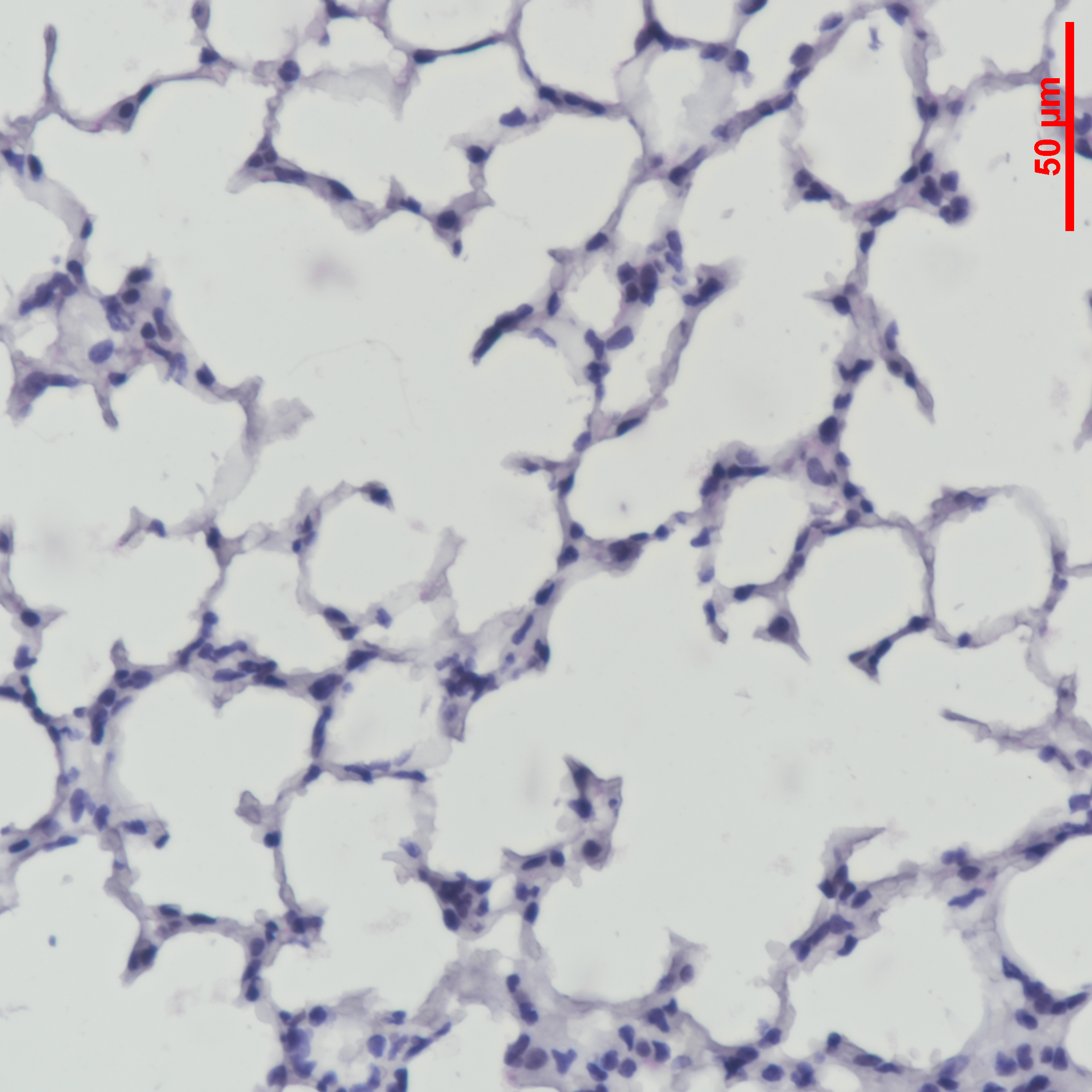

Supplement: Supplementary file 4 — Source Data Fig. 1 [file 44318_2023_3_MOESM4_ESM.zip › Figure1/1n-o/saline yh2ax.tif]

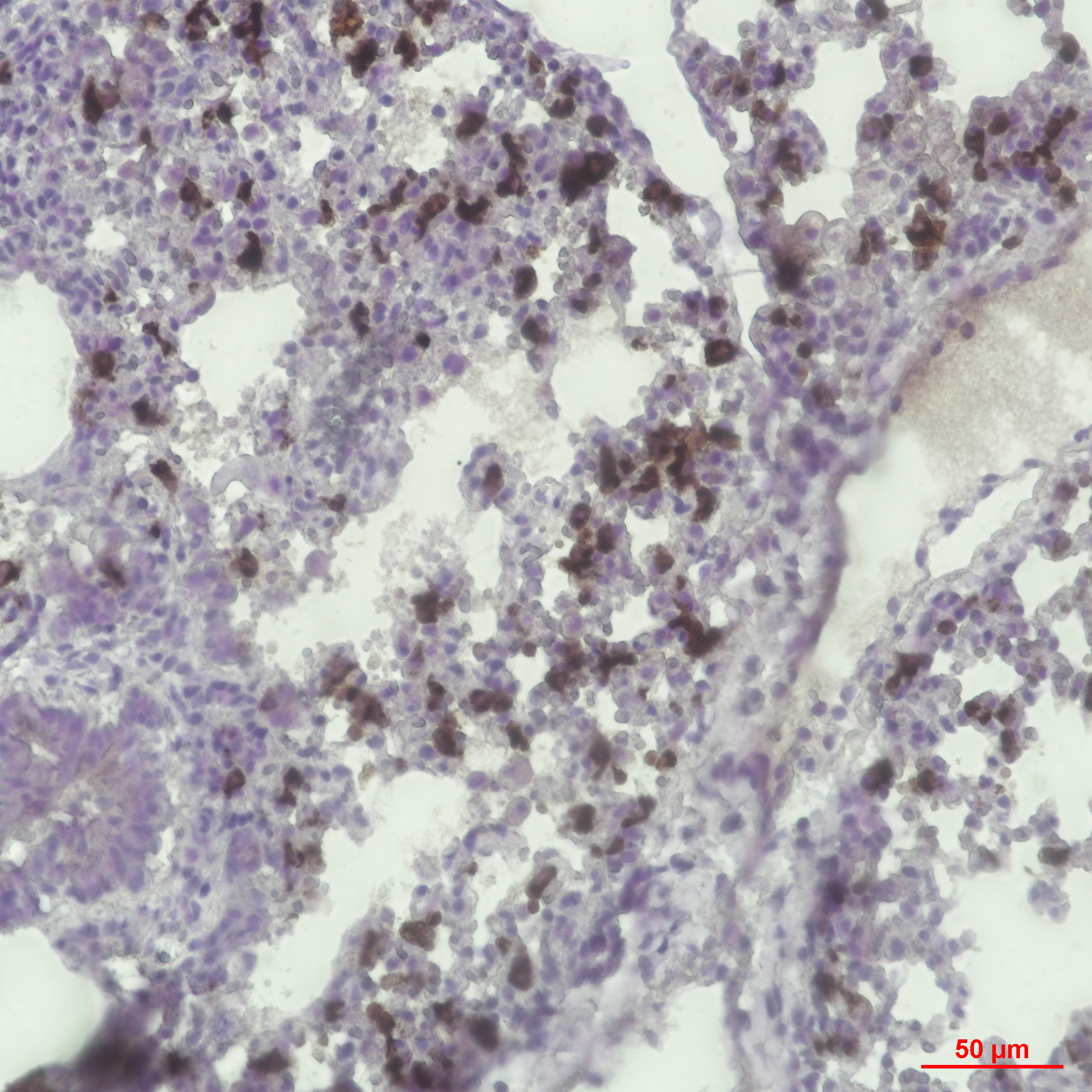

Supplement: Supplementary file 4 — Source Data Fig. 1 [file 44318_2023_3_MOESM4_ESM.zip › Figure1/1n-o/shYTHDC1 yh2ax.tif]

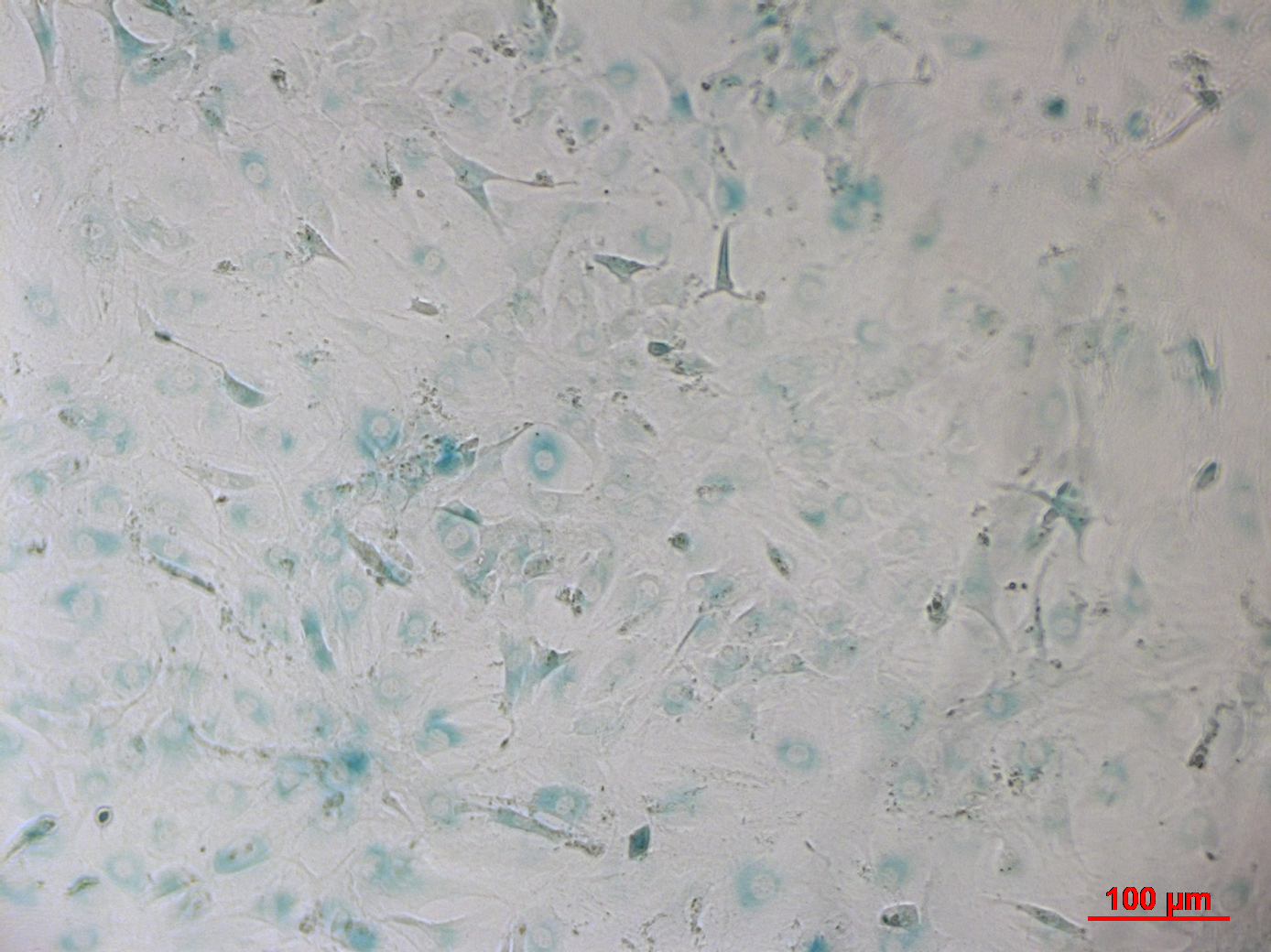

Supplement: Supplementary file 5 — Source Data Fig. 2 [file 44318_2023_3_MOESM5_ESM.zip › Figure2/2a-b/vector SA-β-gal/vector.TIF]

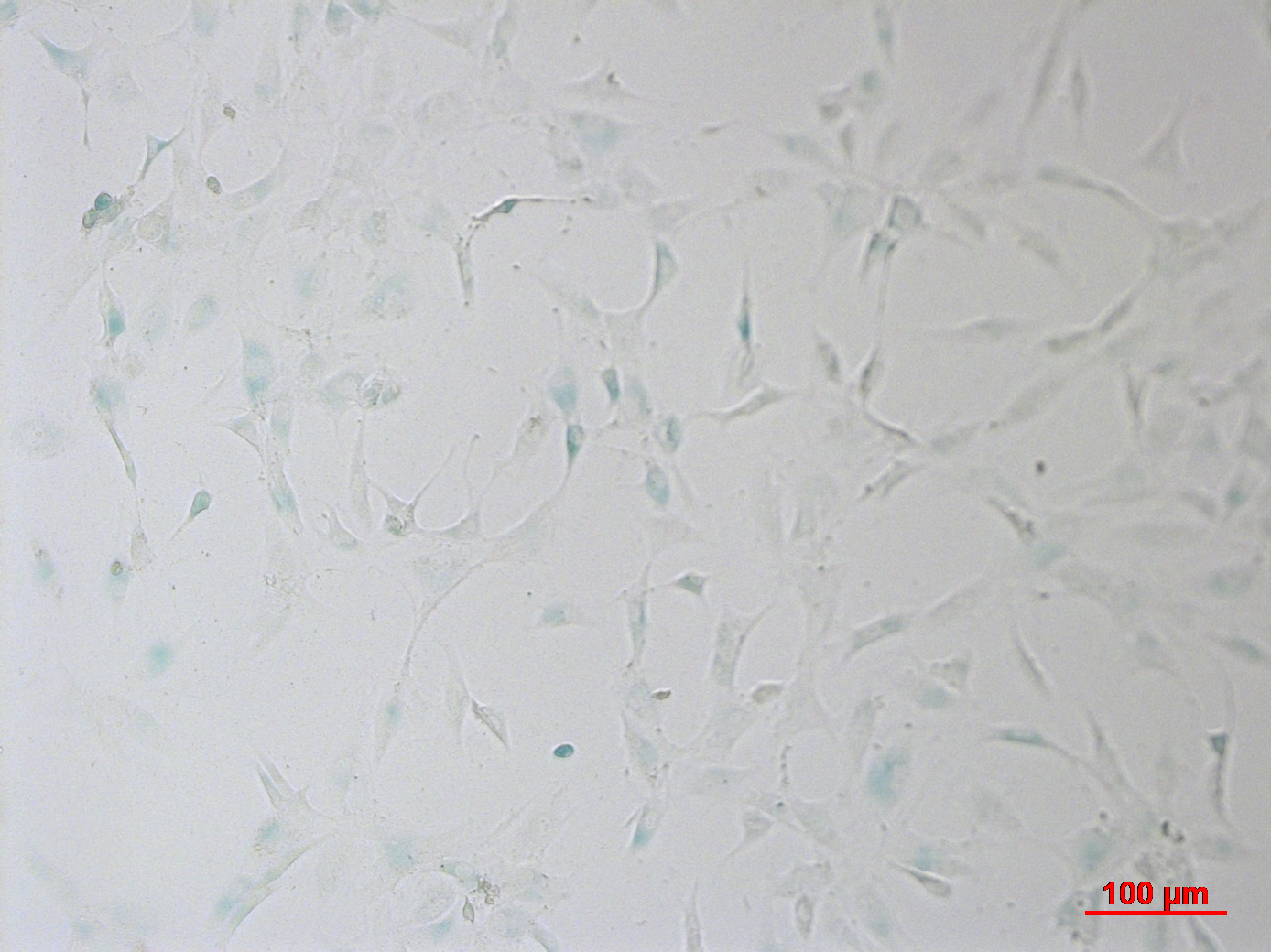

Supplement: Supplementary file 5 — Source Data Fig. 2 [file 44318_2023_3_MOESM5_ESM.zip › Figure2/2a-b/YTHDC1-mut SA-β-gal/YTHDC1-mut.TIF]

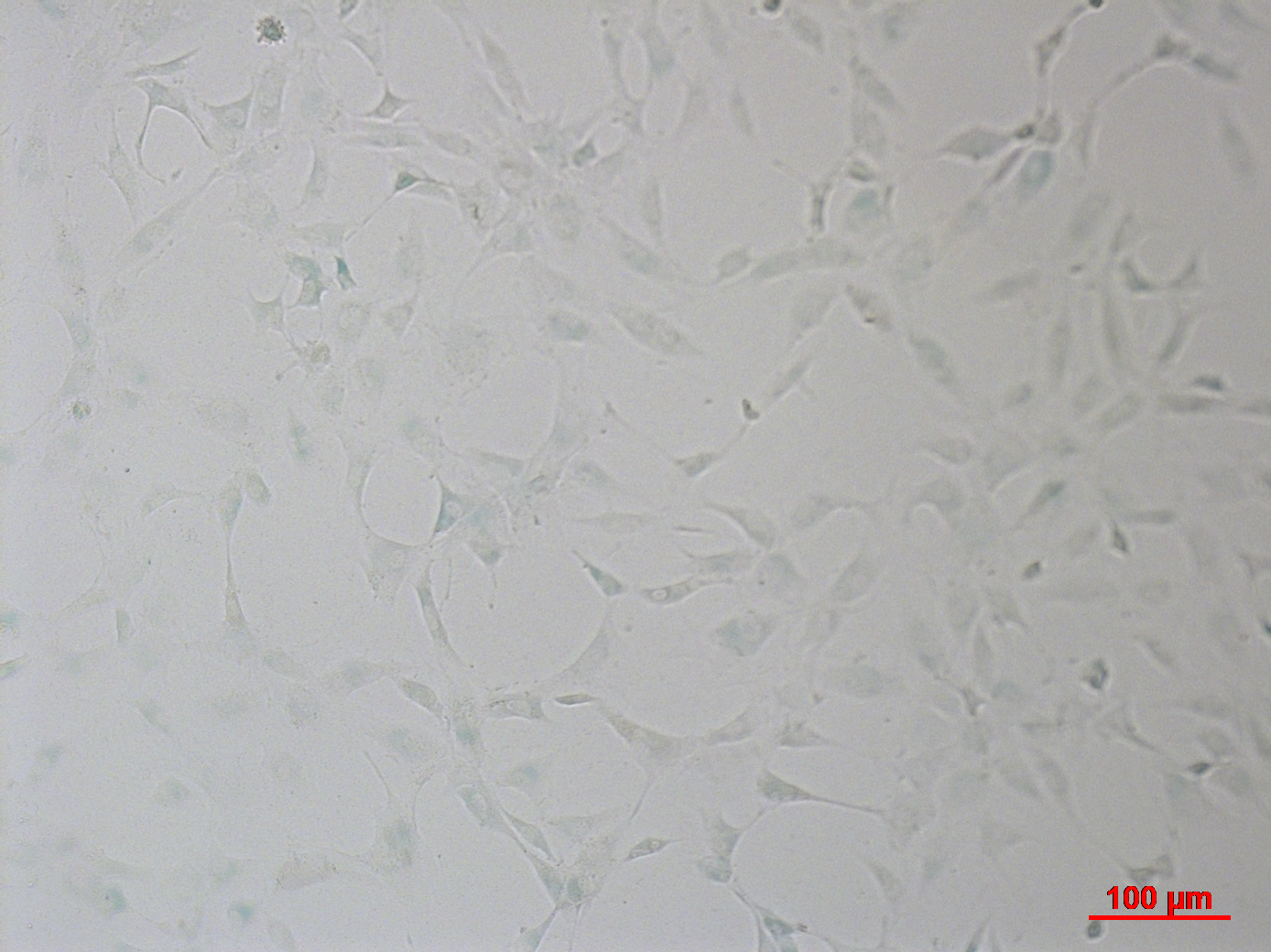

Supplement: Supplementary file 5 — Source Data Fig. 2 [file 44318_2023_3_MOESM5_ESM.zip › Figure2/2a-b/YTHDC1-WT SA-β-gal/YTHDC1-WT.TIF]

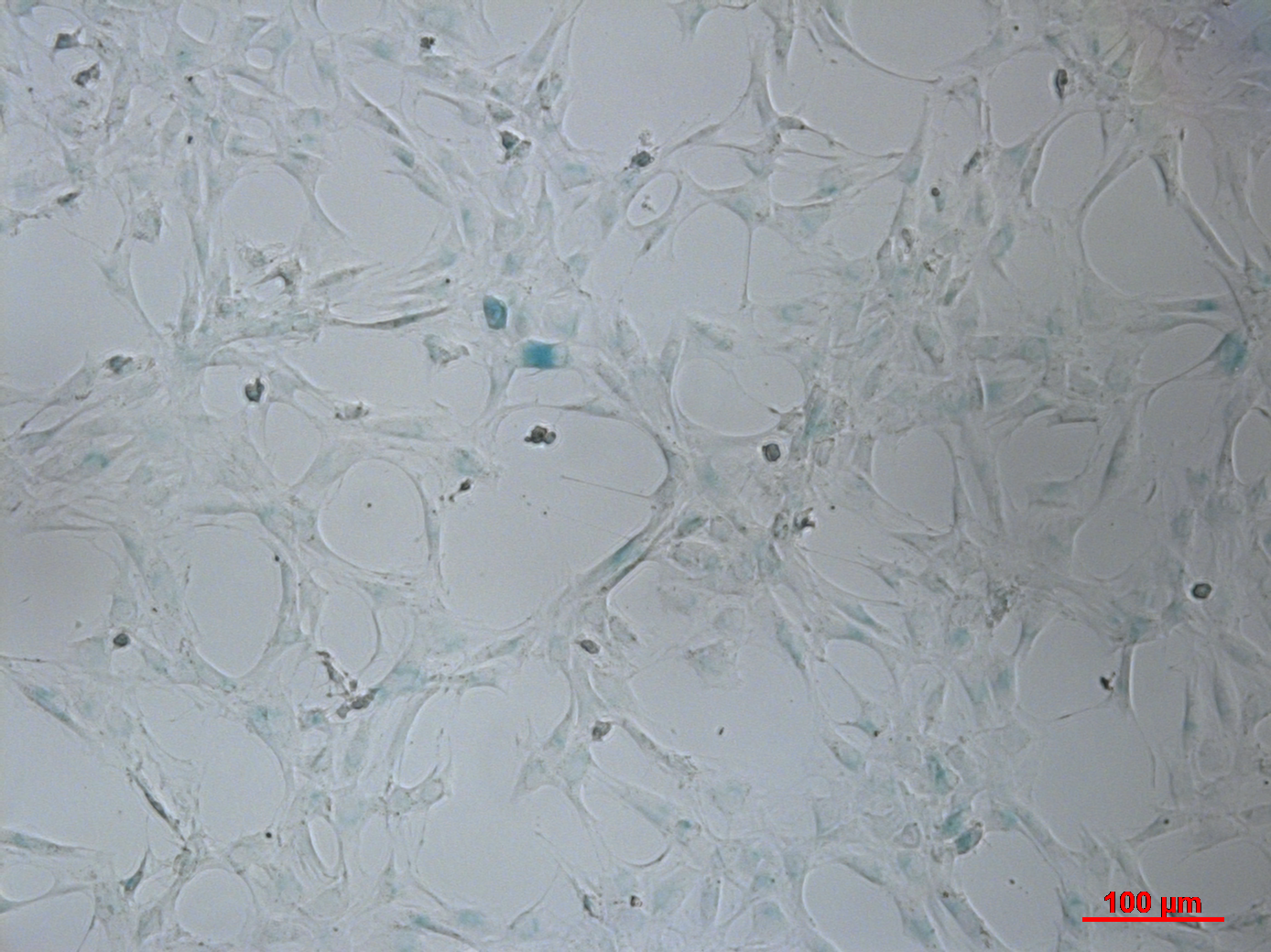

Supplement: Supplementary file 5 — Source Data Fig. 2 [file 44318_2023_3_MOESM5_ESM.zip › Figure2/2c-d/nc SA-β-gal/nc 2_(c1).TIF]

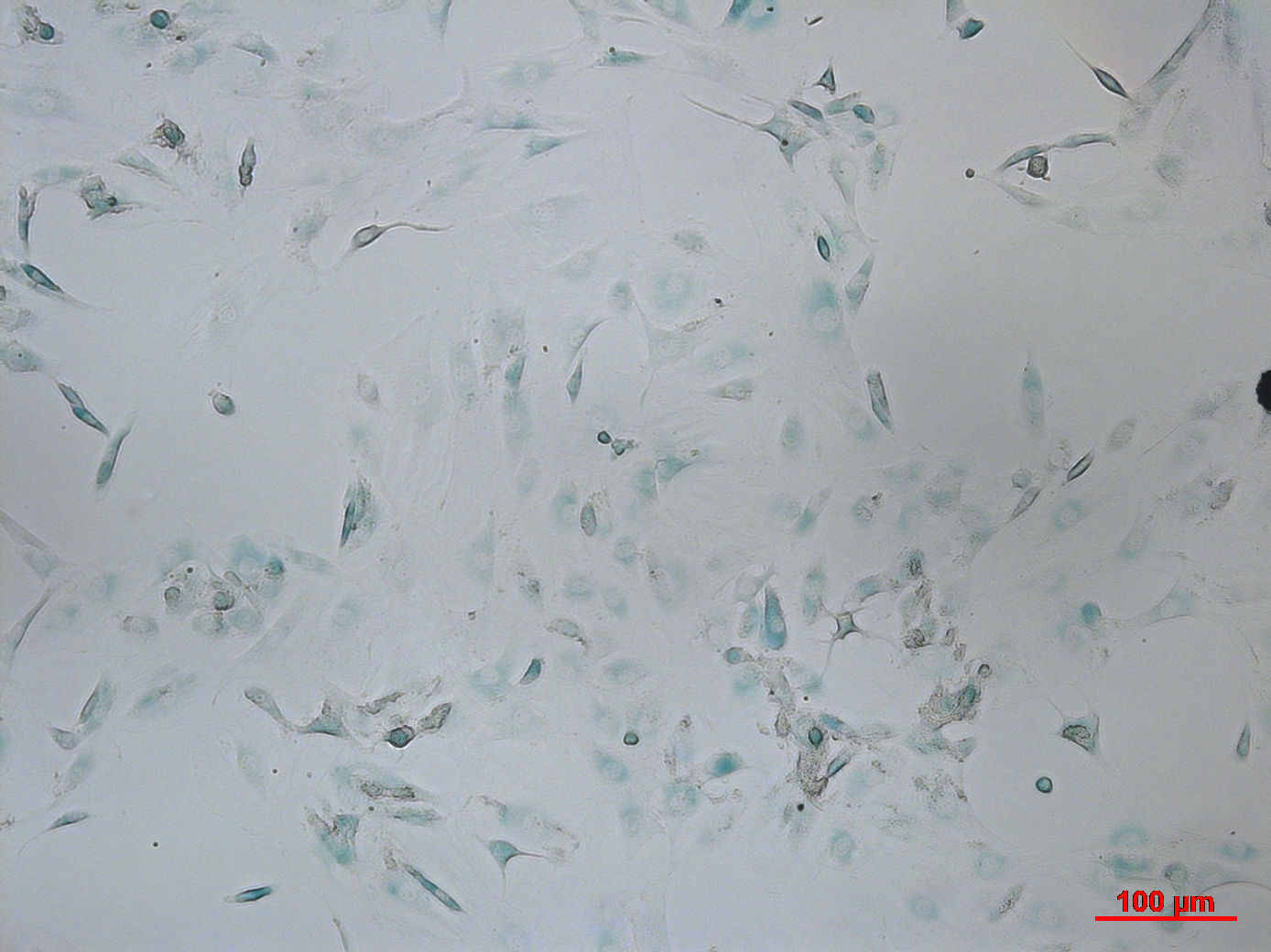

Supplement: Supplementary file 5 — Source Data Fig. 2 [file 44318_2023_3_MOESM5_ESM.zip › Figure2/2c-d/siMETTL3 SA-β-gal/siM3 3_(c1).TIF]

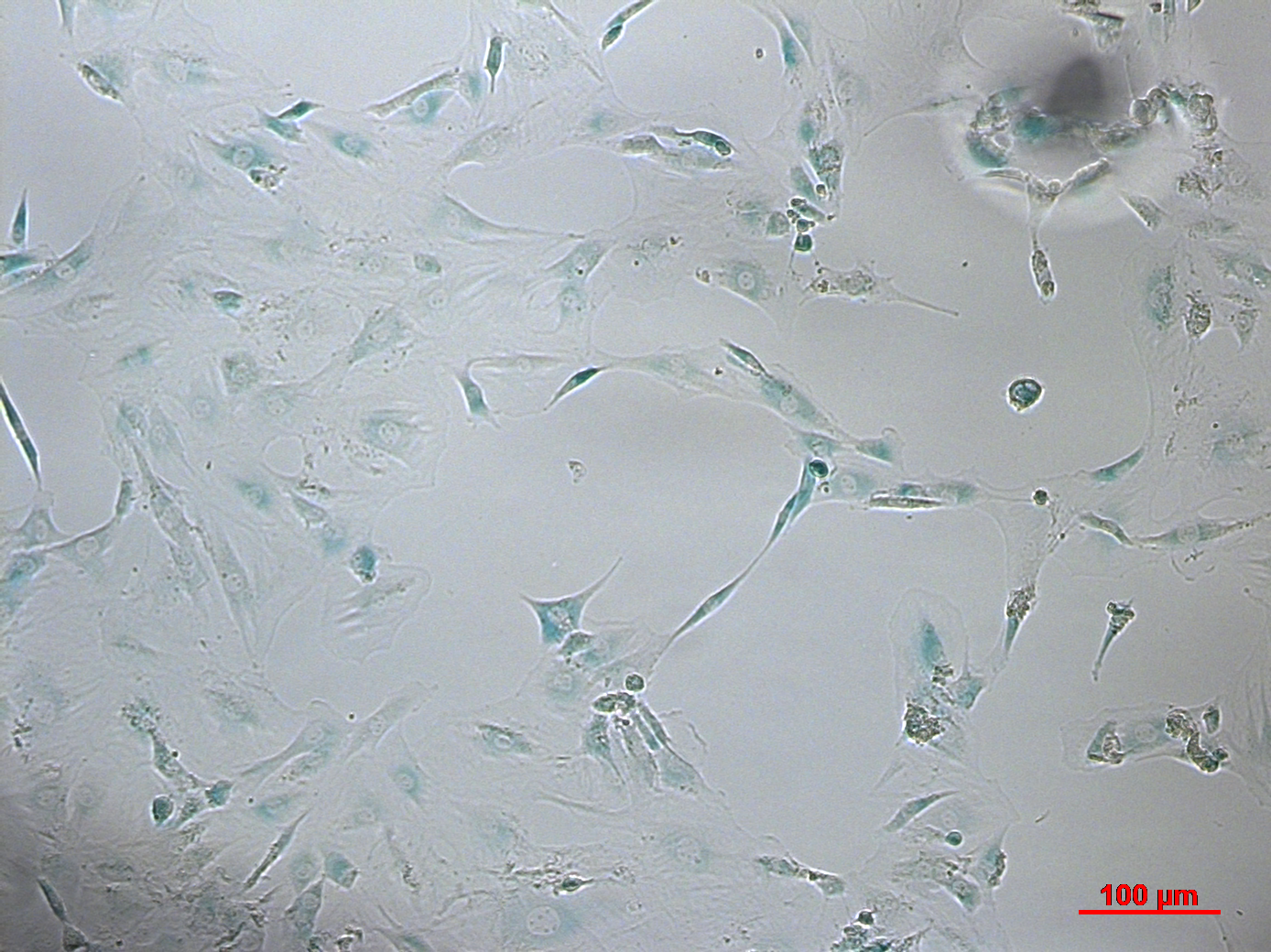

Supplement: Supplementary file 5 — Source Data Fig. 2 [file 44318_2023_3_MOESM5_ESM.zip › Figure2/2c-d/siYTHDC1 SA-β-gal/siC1_(c1).TIF]

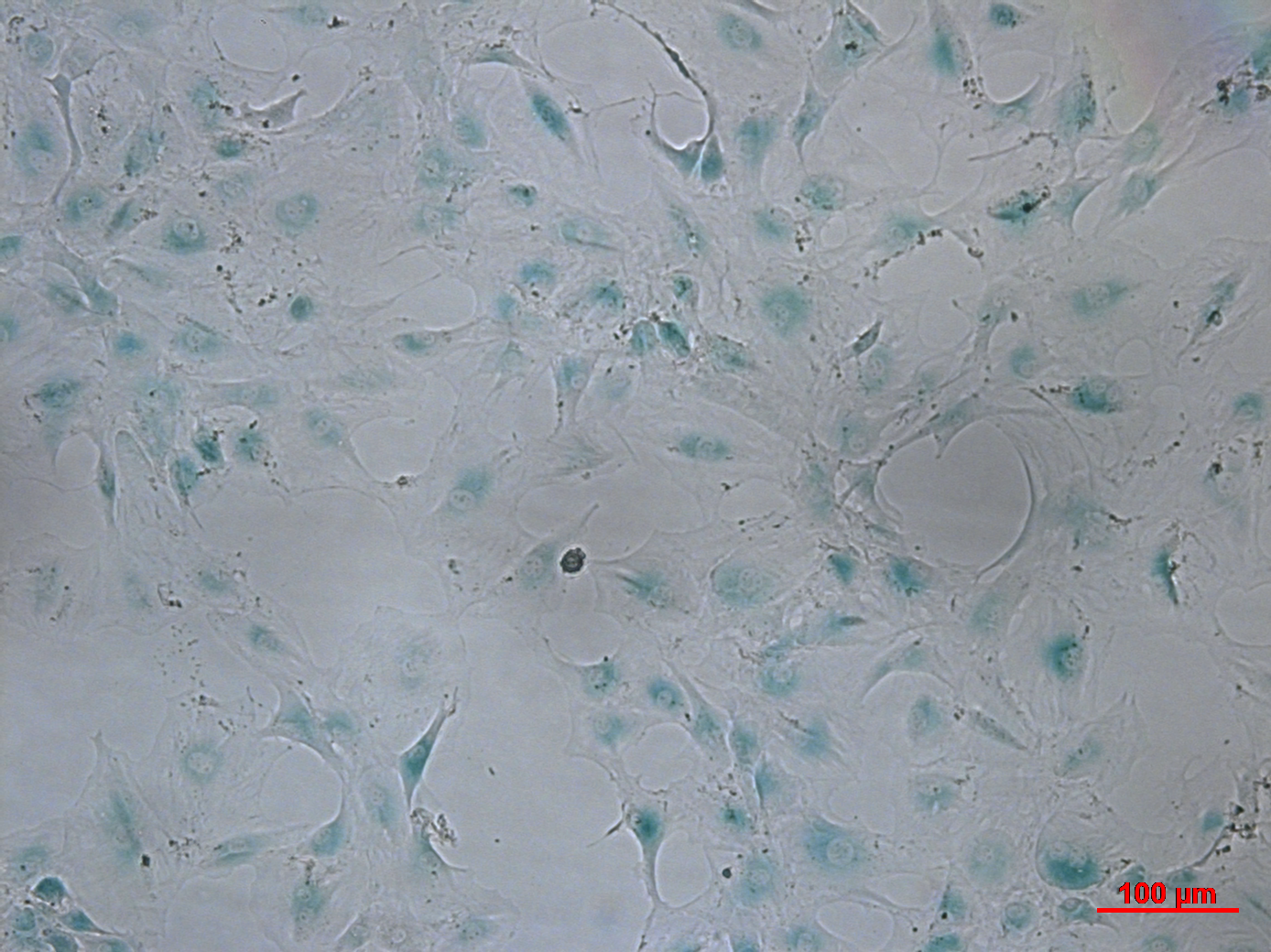

Supplement: Supplementary file 5 — Source Data Fig. 2 [file 44318_2023_3_MOESM5_ESM.zip › Figure2/2c-d/siYTHDC1+siMETTL3 SA-β-gal/siC1+siM3 7_(c1).TIF]

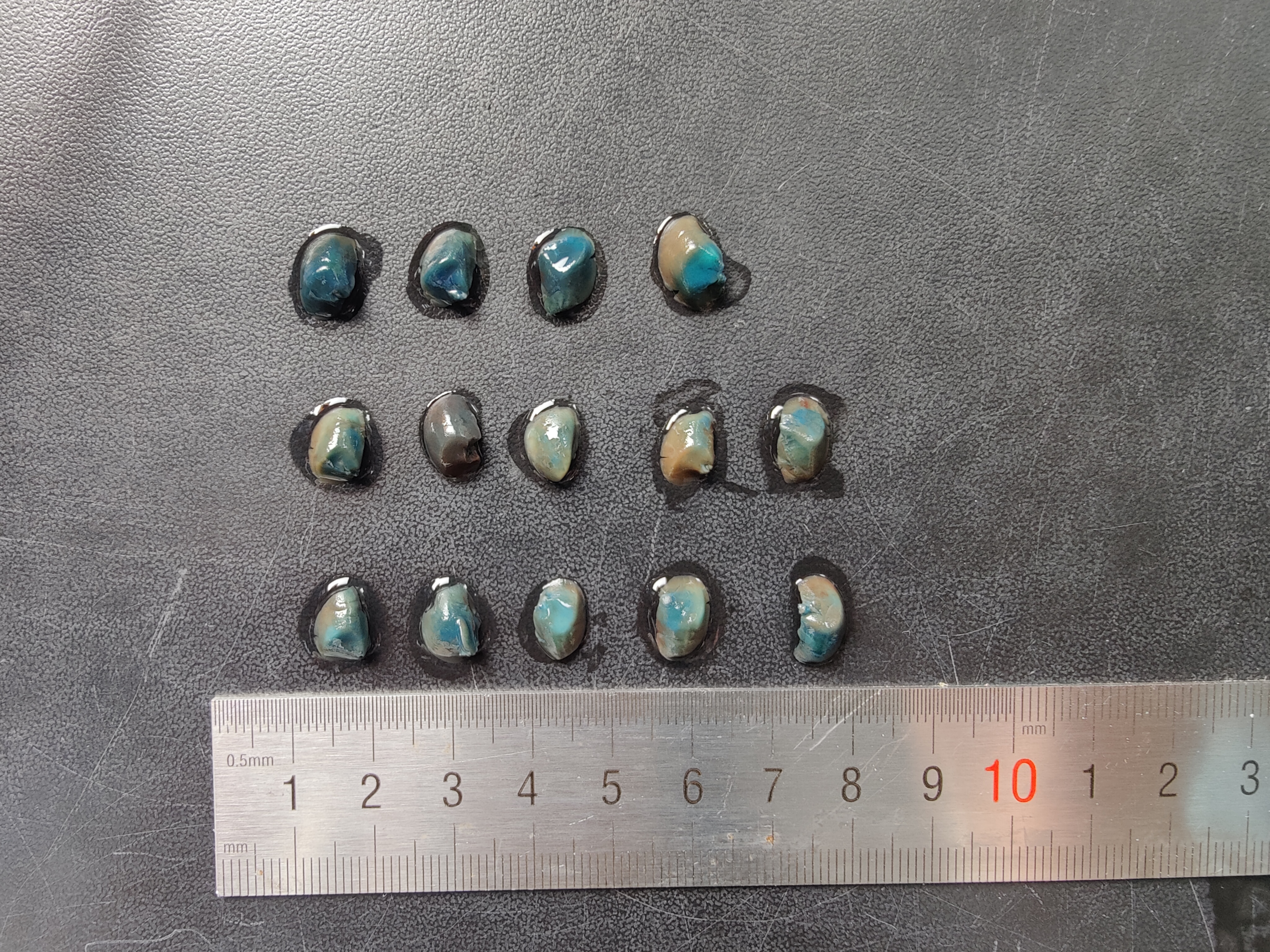

Supplement: Supplementary file 5 — Source Data Fig. 2 [file 44318_2023_3_MOESM5_ESM.zip › Figure2/2f/β-gal staining.jpg]

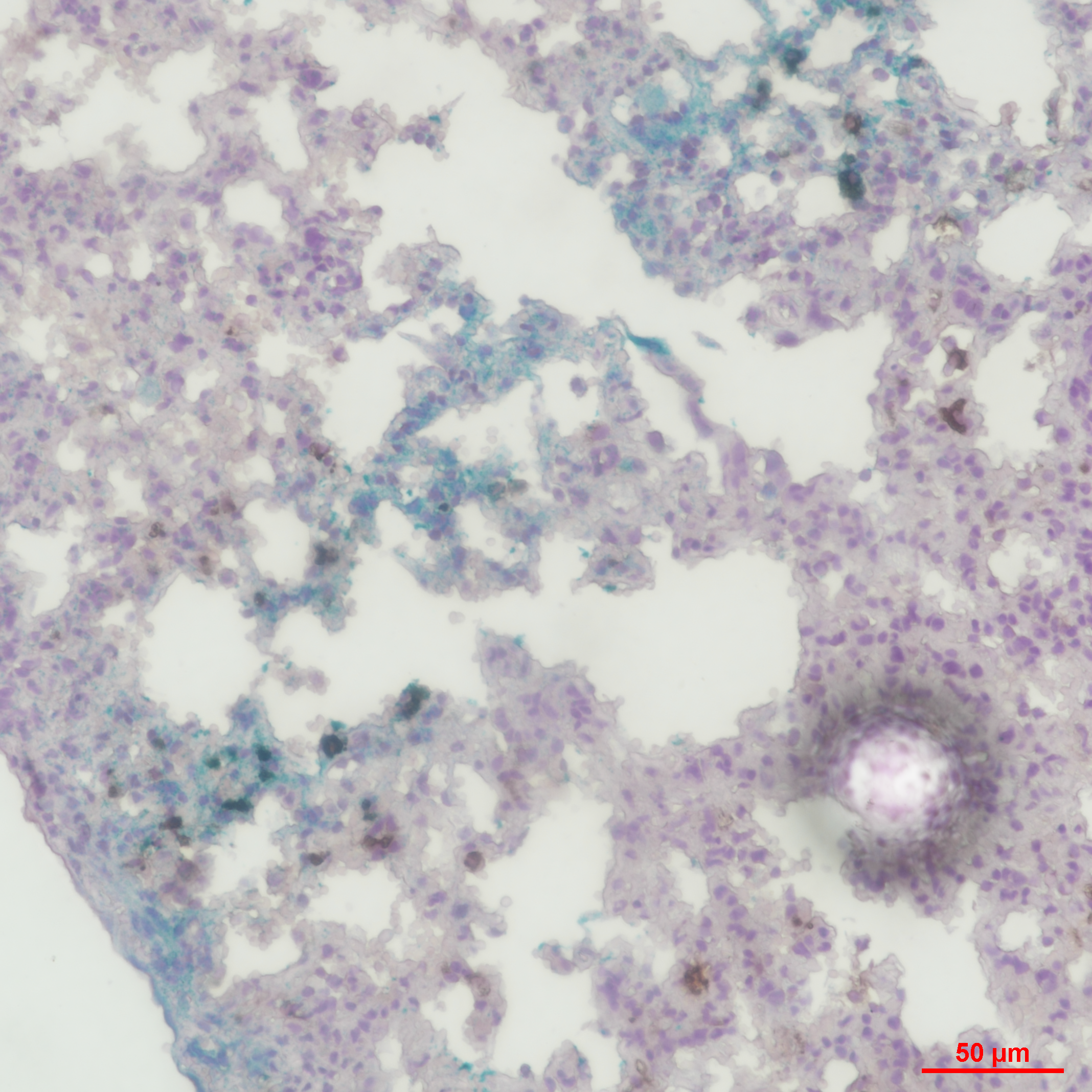

Supplement: Supplementary file 5 — Source Data Fig. 2 [file 44318_2023_3_MOESM5_ESM.zip › Figure2/2h-i/vector SPC and SA-β-gal.tif]

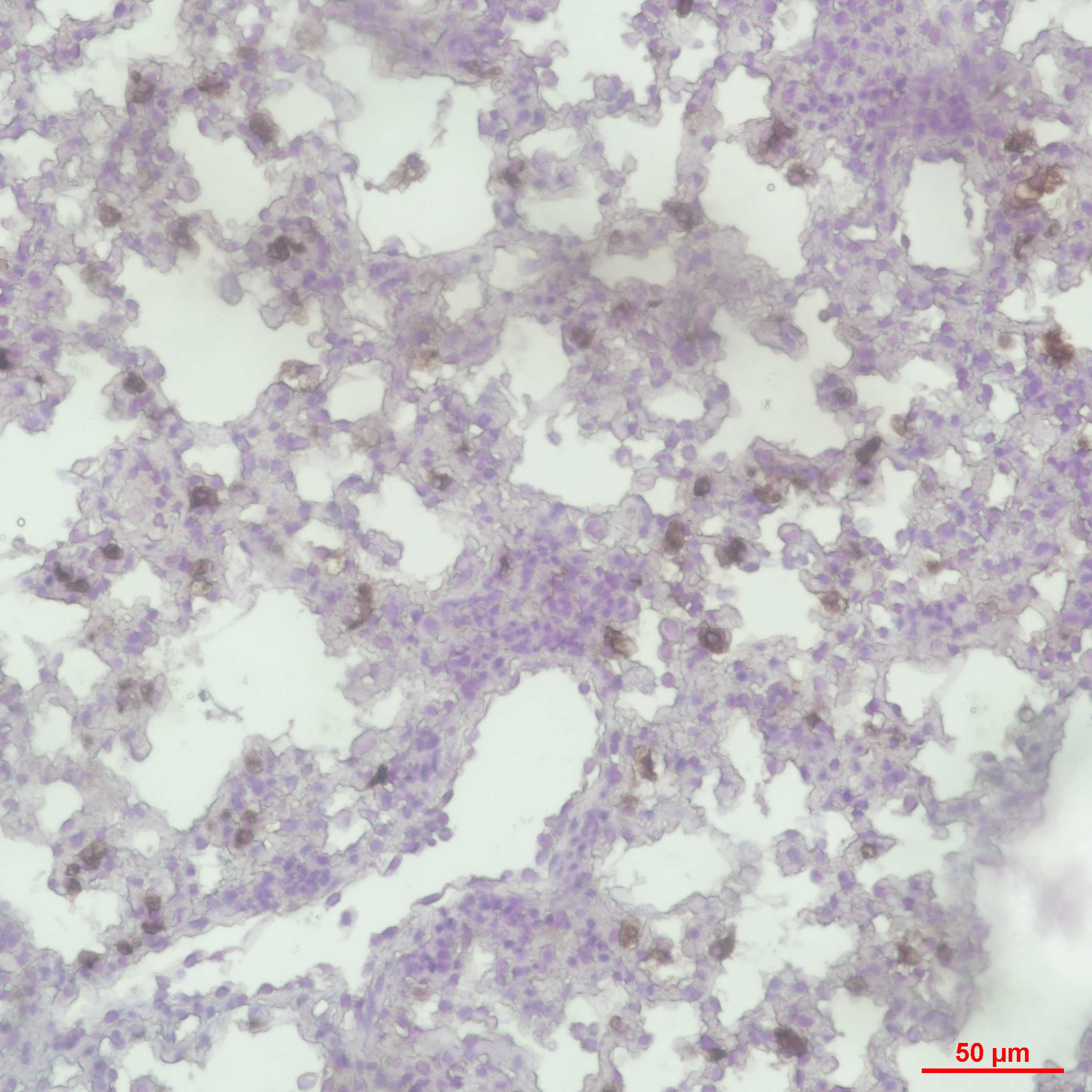

Supplement: Supplementary file 5 — Source Data Fig. 2 [file 44318_2023_3_MOESM5_ESM.zip › Figure2/2h-i/YTHDC1-MUT SPC and SA-β-gal.tif]

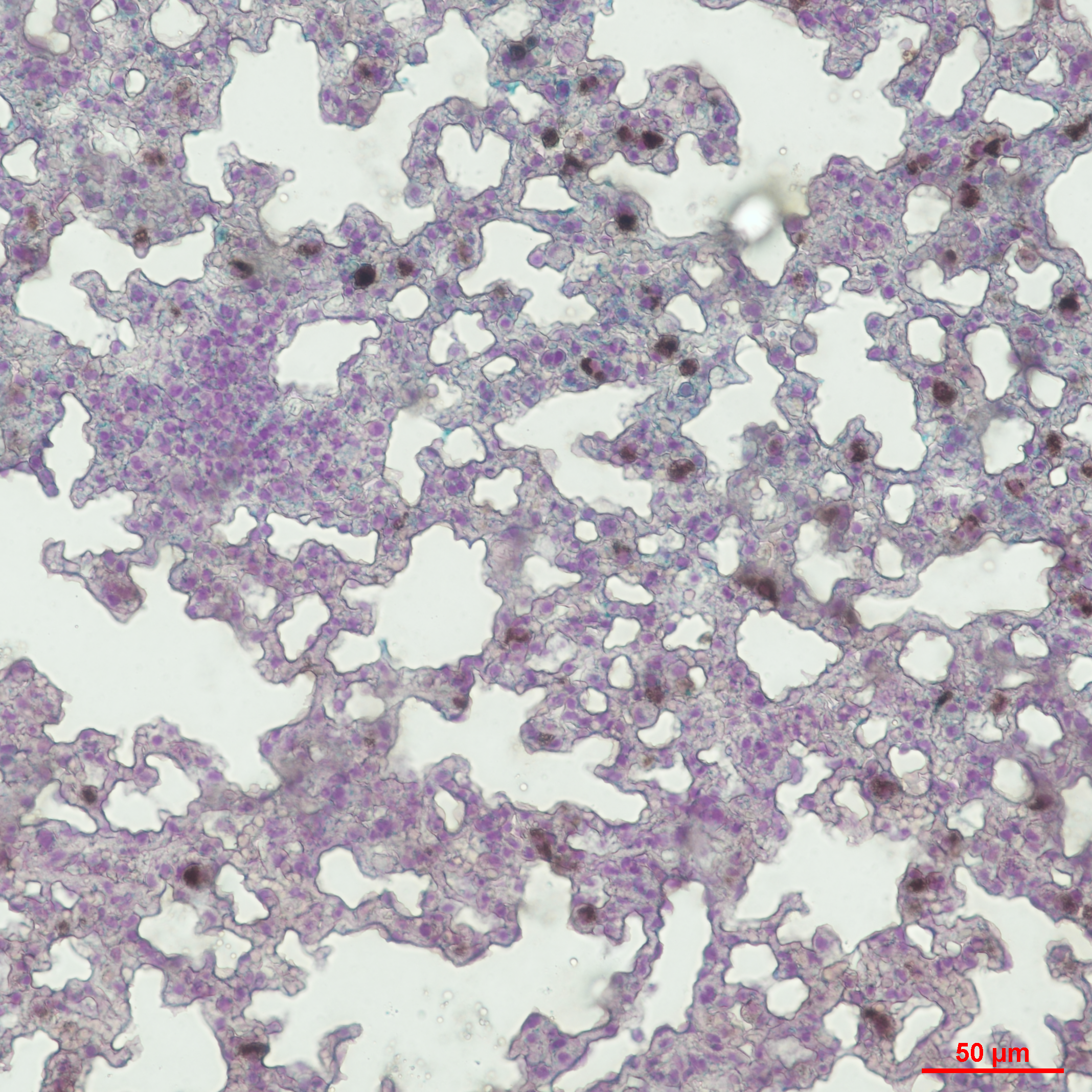

Supplement: Supplementary file 5 — Source Data Fig. 2 [file 44318_2023_3_MOESM5_ESM.zip › Figure2/2h-i/YTHDC1-WT SPC and SA-β-gal.tif]

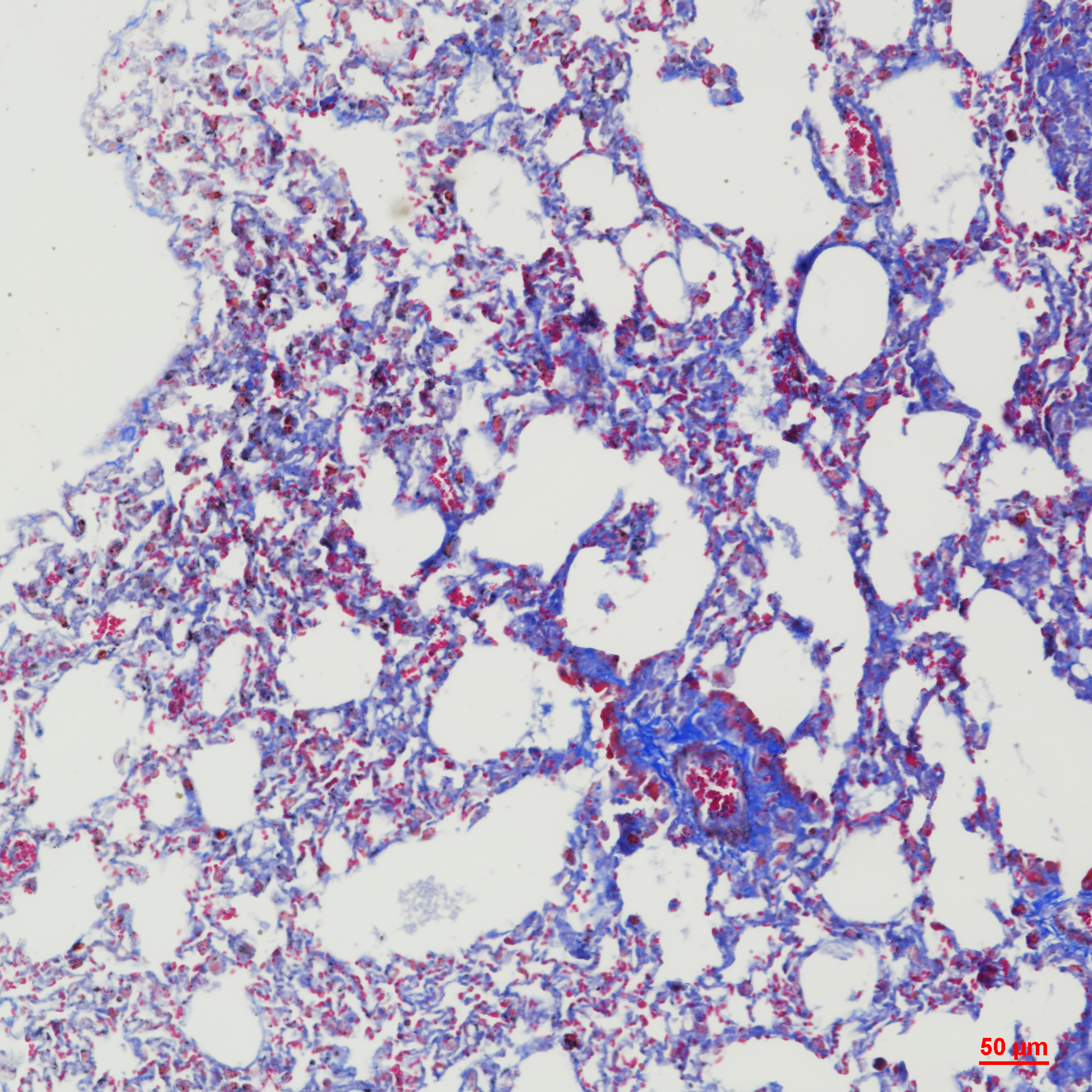

Supplement: Supplementary file 5 — Source Data Fig. 2 [file 44318_2023_3_MOESM5_ESM.zip › Figure2/2j-k/VECTOR masson.tif]

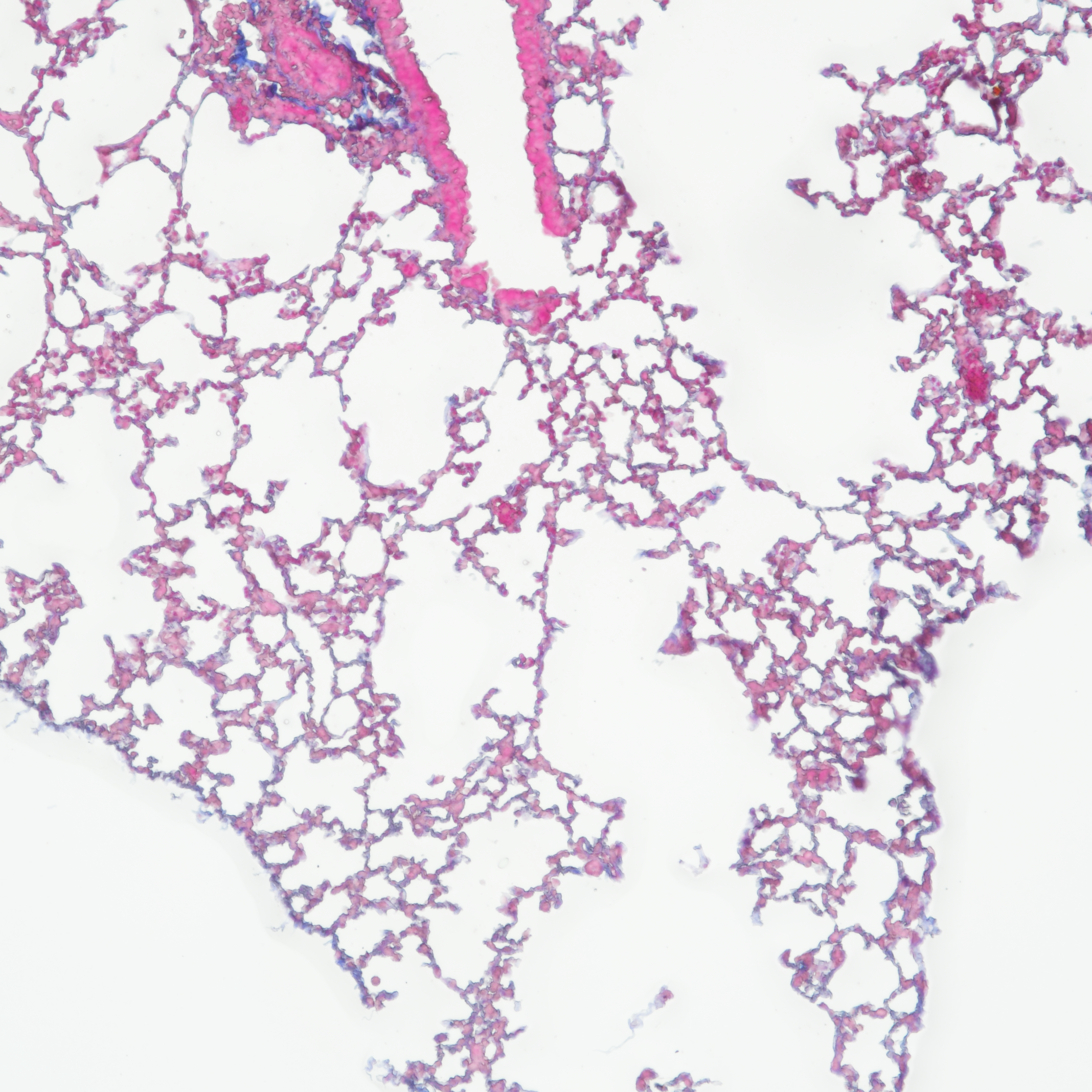

Supplement: Supplementary file 5 — Source Data Fig. 2 [file 44318_2023_3_MOESM5_ESM.zip › Figure2/2j-k/YTHDC1-MUT masson.tif]

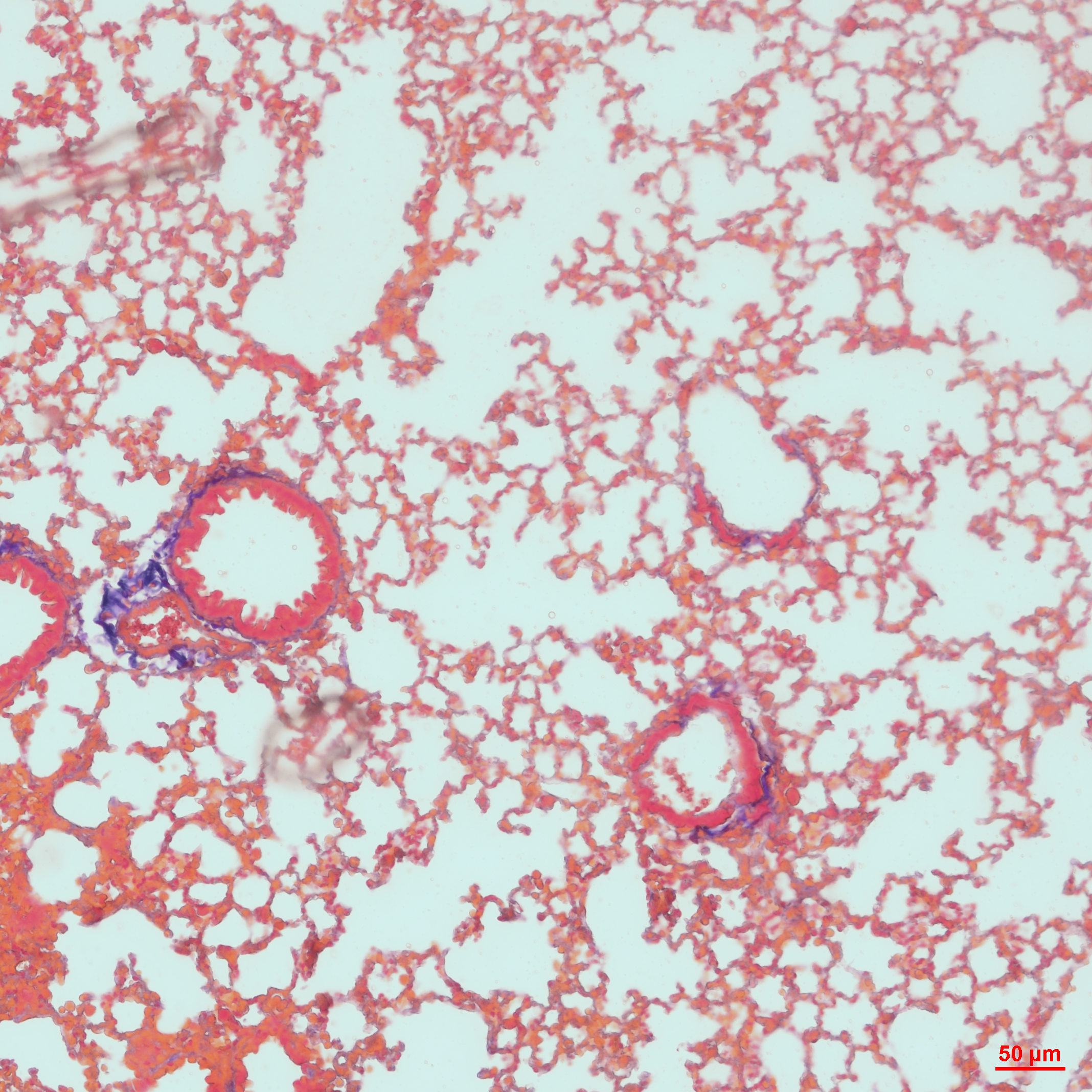

Supplement: Supplementary file 5 — Source Data Fig. 2 [file 44318_2023_3_MOESM5_ESM.zip › Figure2/2j-k/YTHDC1-WT masson.tif]

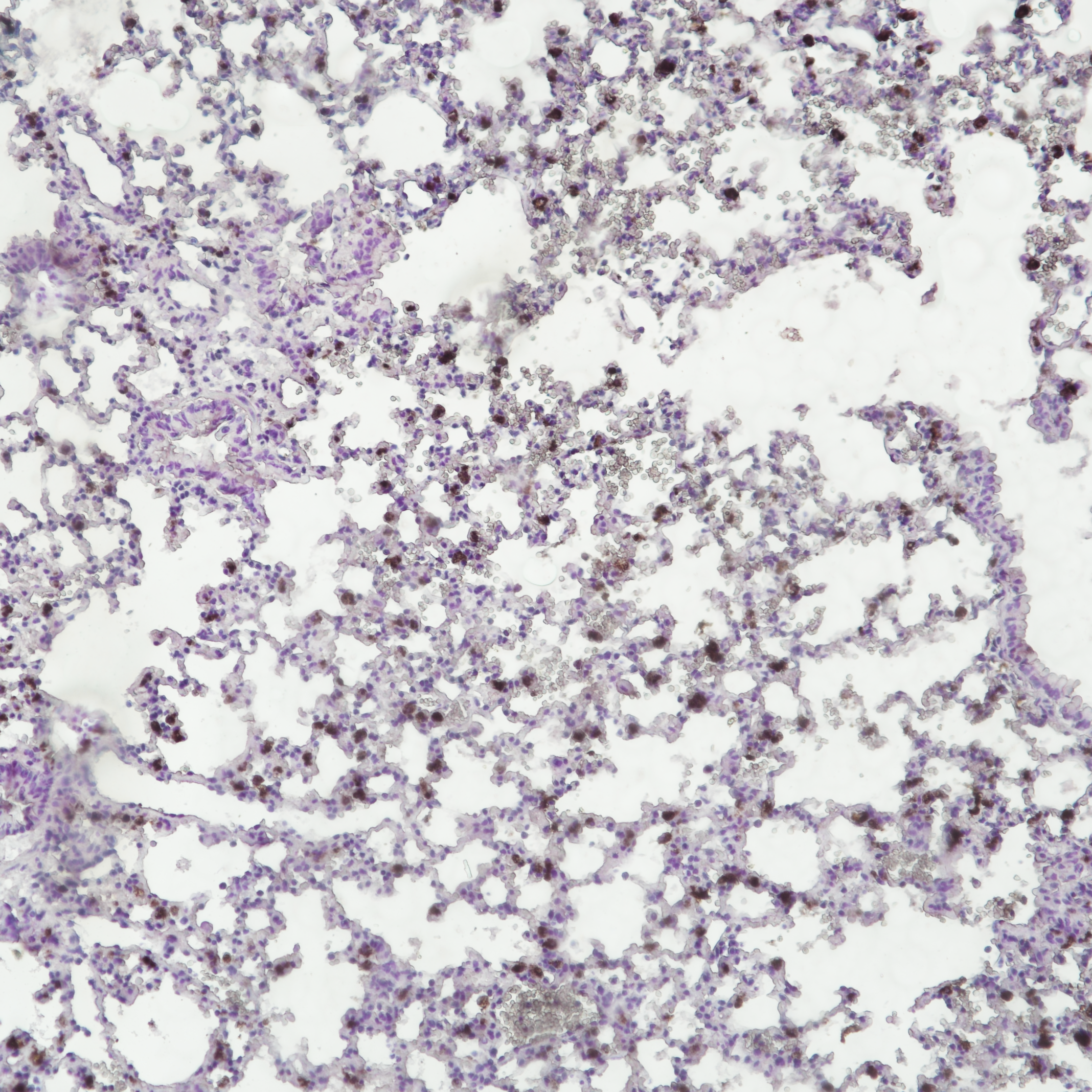

Supplement: Supplementary file 5 — Source Data Fig. 2 [file 44318_2023_3_MOESM5_ESM.zip › Figure2/2l-m/Vector yh2ax.tif]

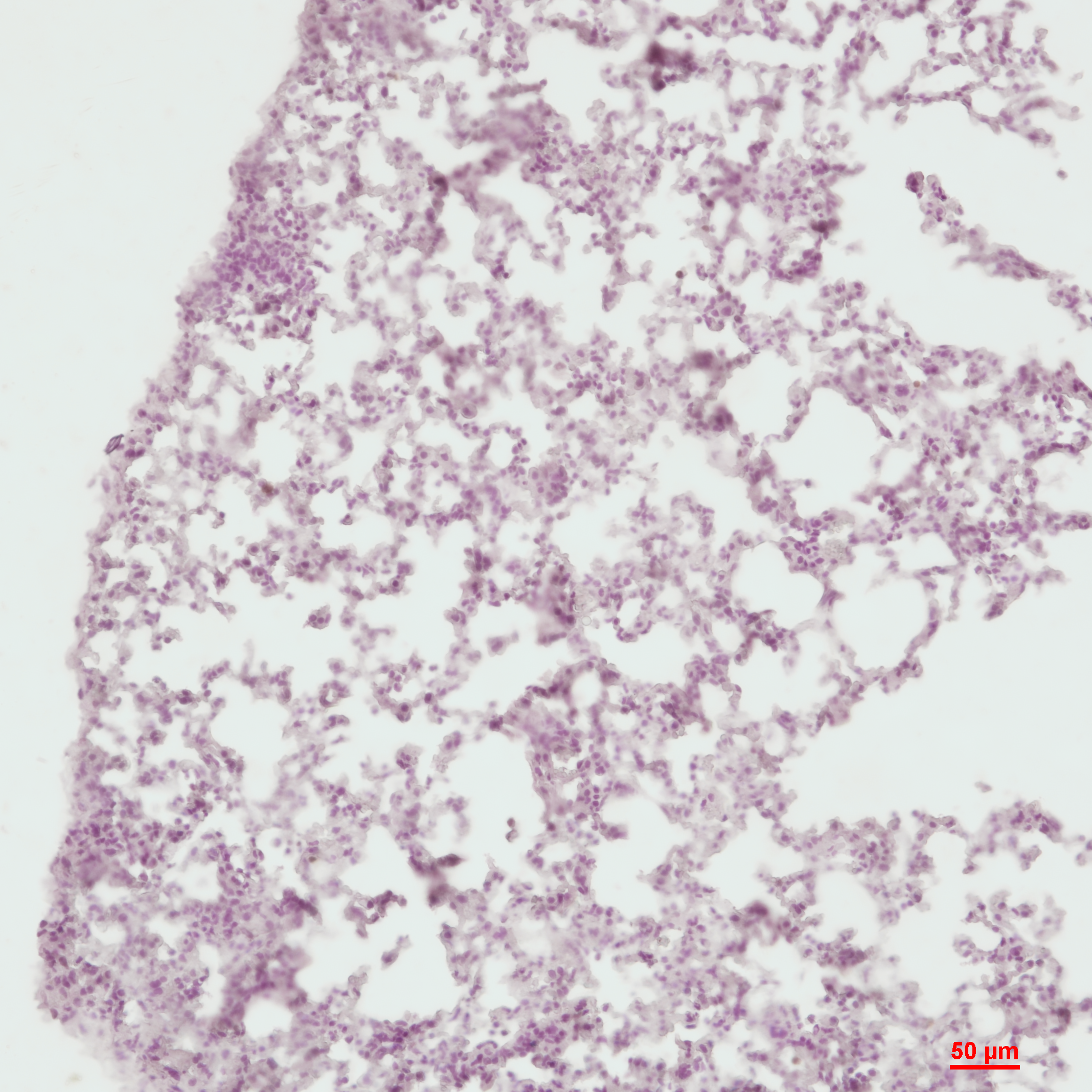

Supplement: Supplementary file 5 — Source Data Fig. 2 [file 44318_2023_3_MOESM5_ESM.zip › Figure2/2l-m/YTHDC1-MUT yh2ax.tif]

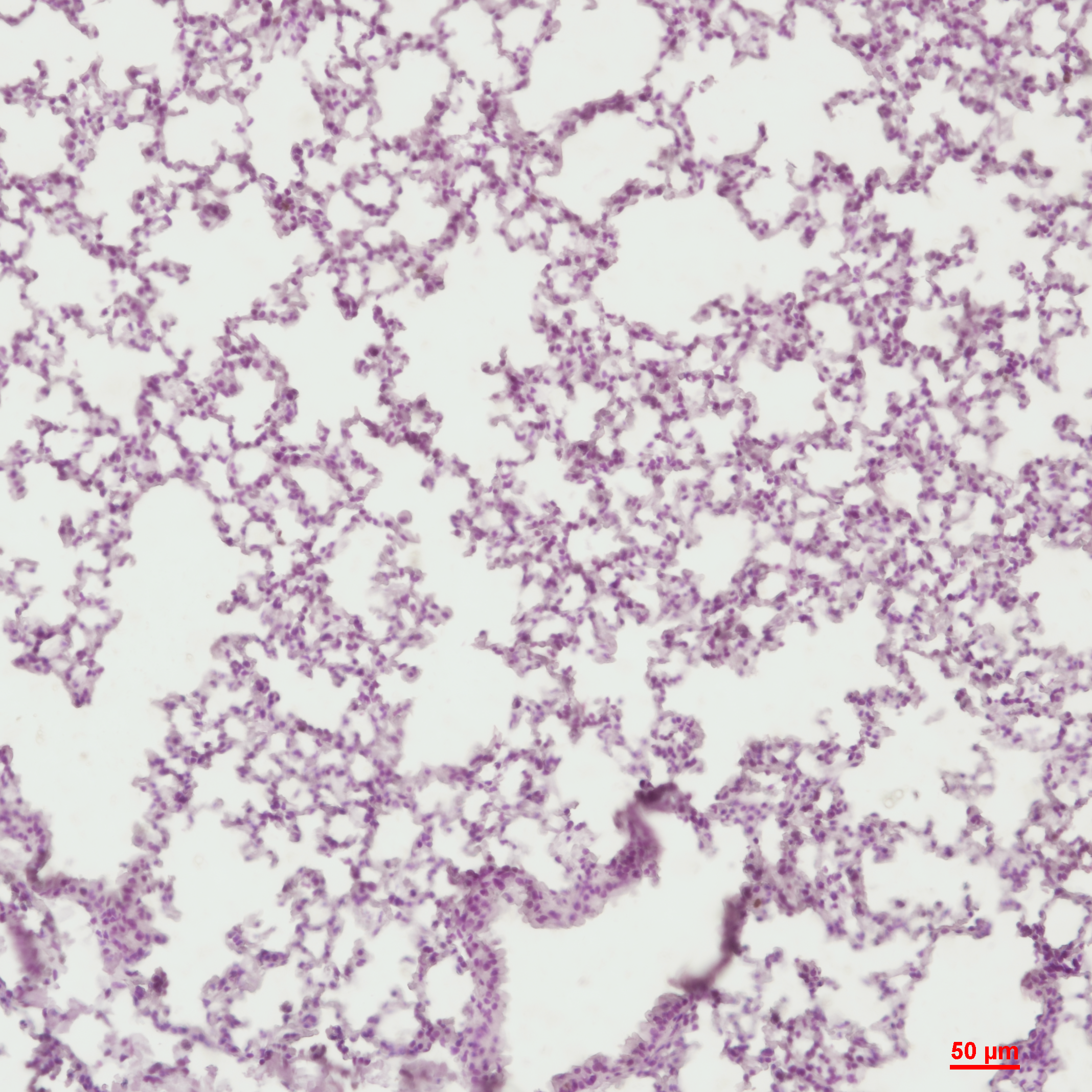

Supplement: Supplementary file 5 — Source Data Fig. 2 [file 44318_2023_3_MOESM5_ESM.zip › Figure2/2l-m/YTHDC1-WT yh2ax.tif]

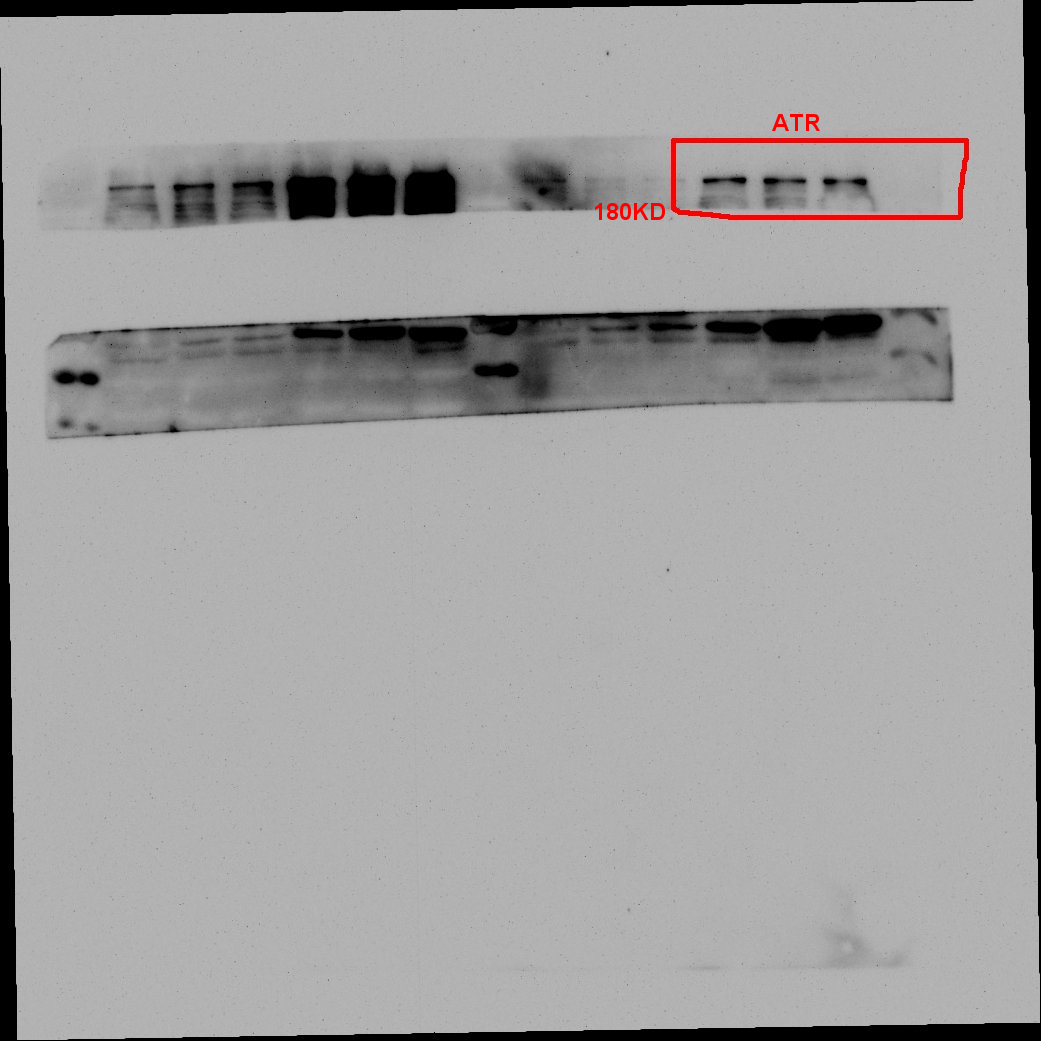

Supplement: Supplementary file 6 — Source Data Fig. 3 [file 44318_2023_3_MOESM6_ESM.zip › Figure3/3a-c/20200927 ATR (UP) nc,siDC1-1,SiDC1-3VP16.tif]

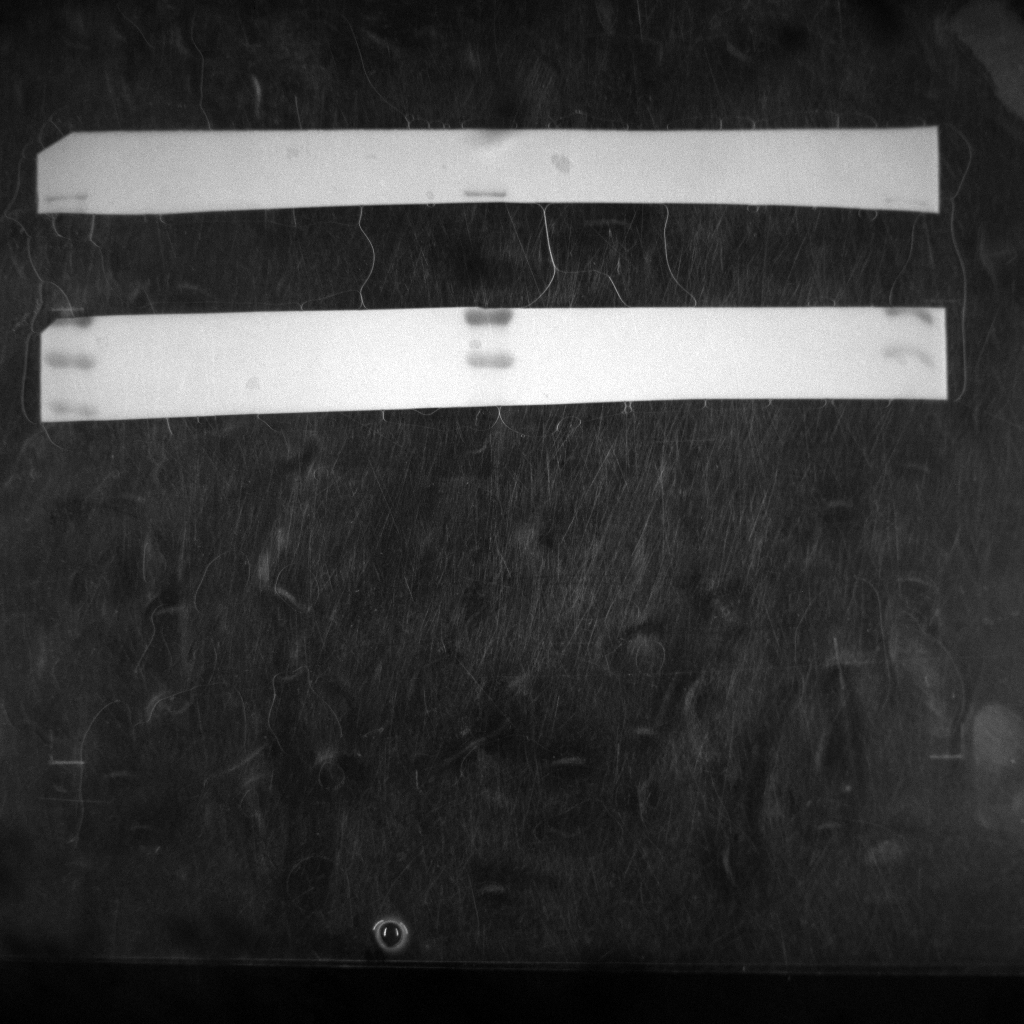

Supplement: Supplementary file 6 — Source Data Fig. 3 [file 44318_2023_3_MOESM6_ESM.zip › Figure3/3a-c/20200927 ATR (UP) nc,siDC1-1,SiDC1-3VP16white .tif]

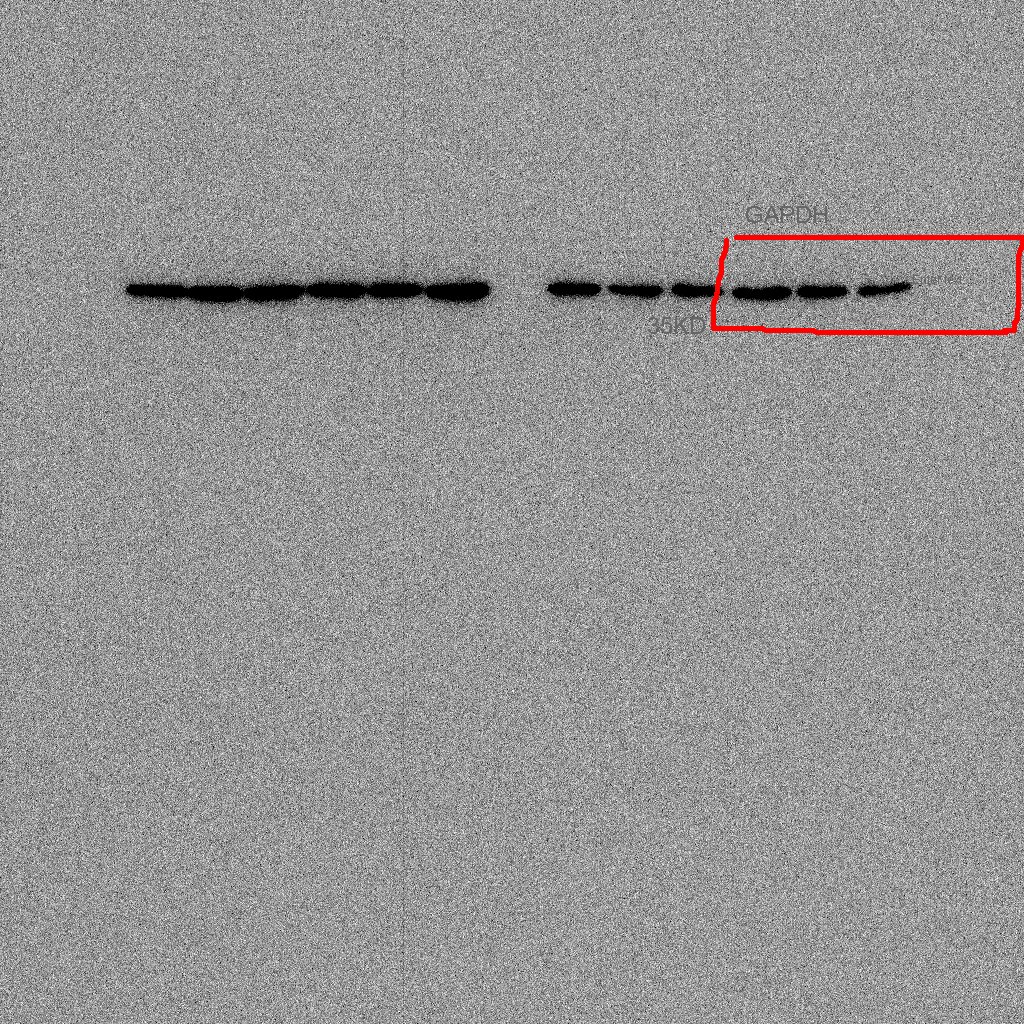

Supplement: Supplementary file 6 — Source Data Fig. 3 [file 44318_2023_3_MOESM6_ESM.zip › Figure3/3a-c/20200927 GAPDH nc,siDC1-1,SiDC1-3(VP-16).jpg]

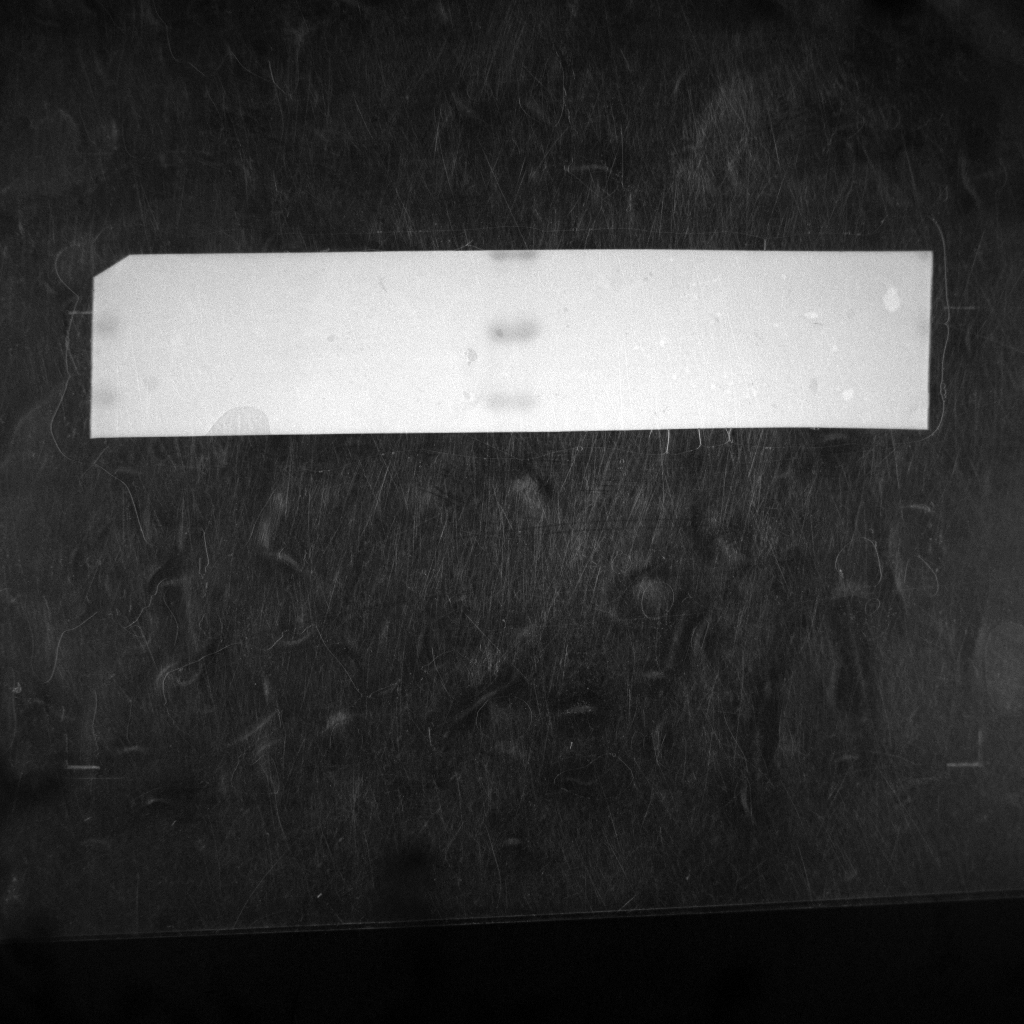

Supplement: Supplementary file 6 — Source Data Fig. 3 [file 44318_2023_3_MOESM6_ESM.zip › Figure3/3a-c/20200927 GAPDH nc,siDC1-1,SiDC1-3VP-16 white .tif]

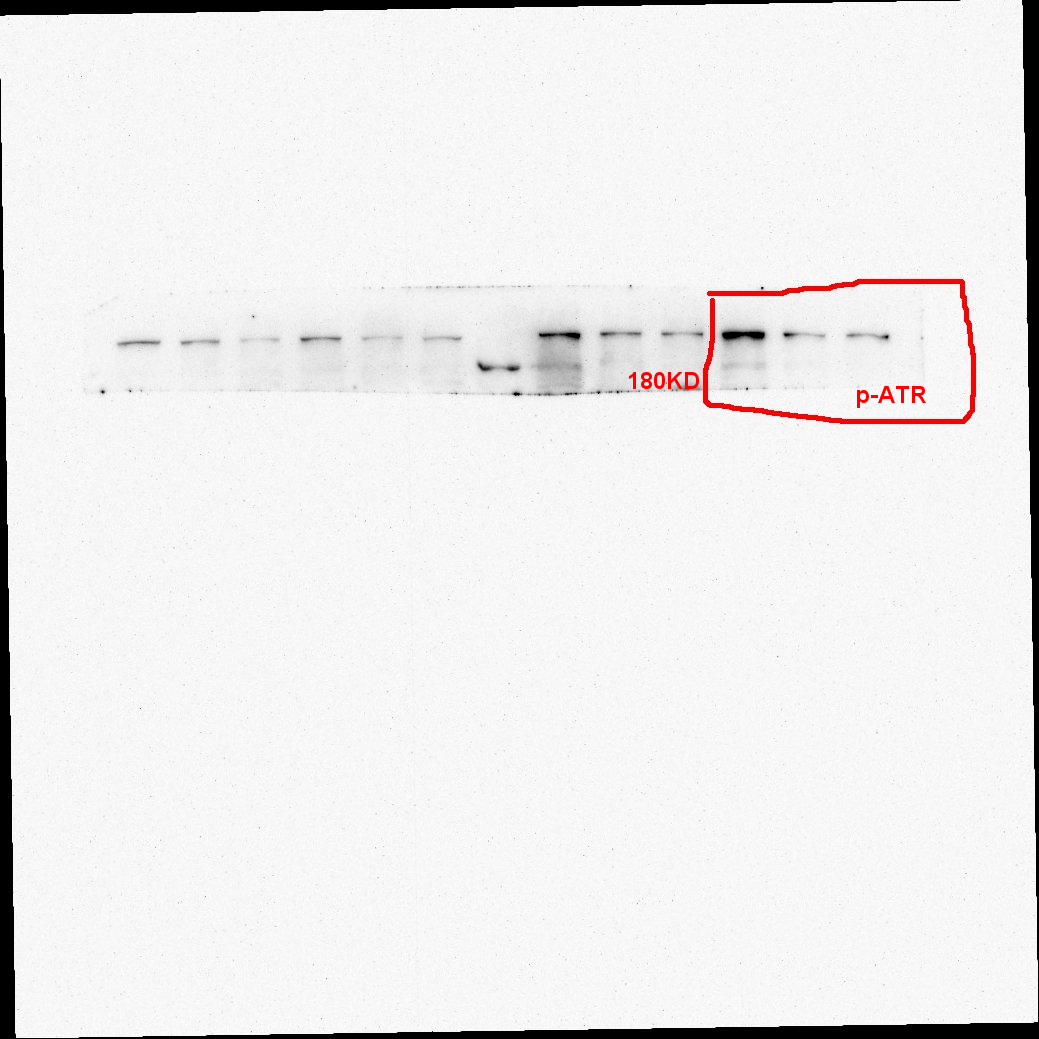

Supplement: Supplementary file 6 — Source Data Fig. 3 [file 44318_2023_3_MOESM6_ESM.zip › Figure3/3a-c/20200927 p-ATR nc,siDC1-1,SiDC1-3 VP-16.tif]

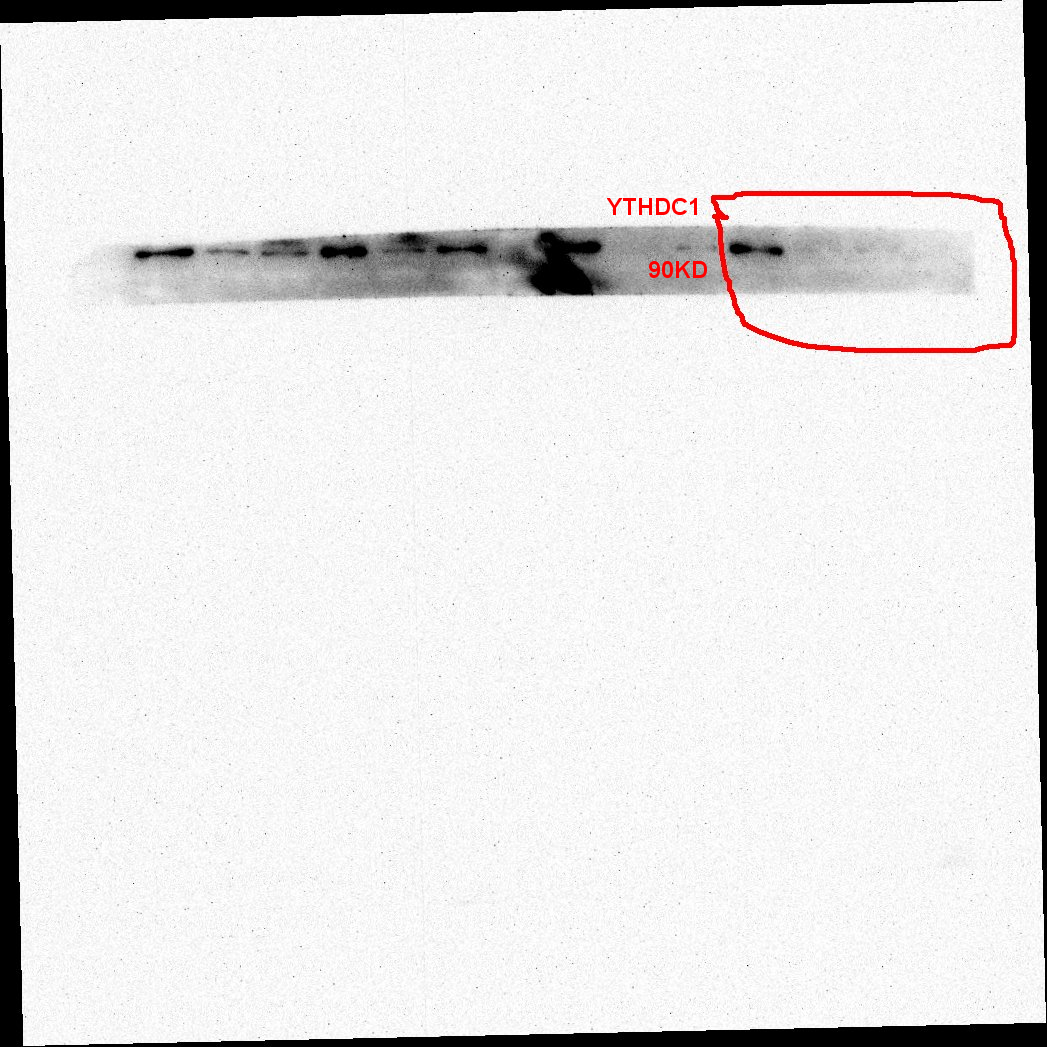

Supplement: Supplementary file 6 — Source Data Fig. 3 [file 44318_2023_3_MOESM6_ESM.zip › Figure3/3a-c/20200927 YTHDC1 nc,siDC1-1,SiDC1-3(VP-16) .tif]

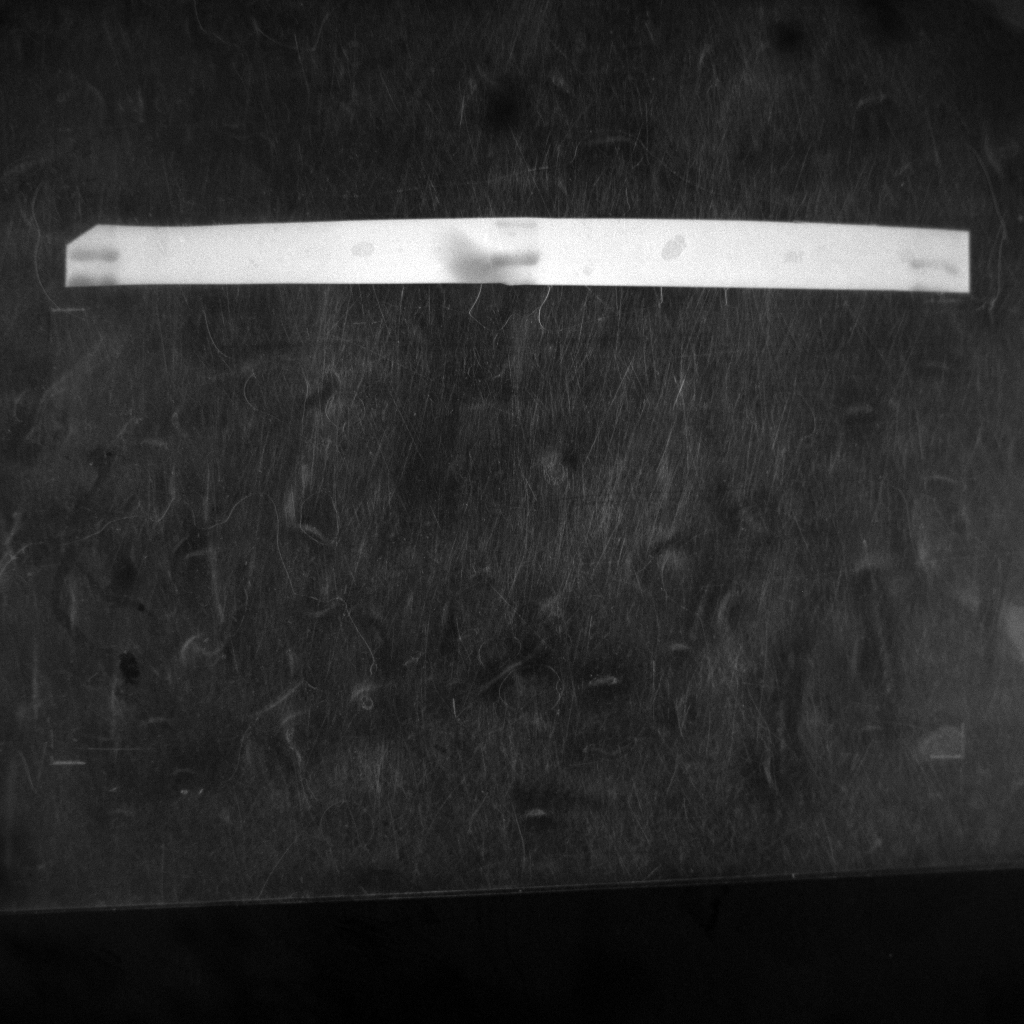

Supplement: Supplementary file 6 — Source Data Fig. 3 [file 44318_2023_3_MOESM6_ESM.zip › Figure3/3a-c/20200927 YTHDC1 nc,siDC1-1,SiDC1-3(VP-16) White .tif]

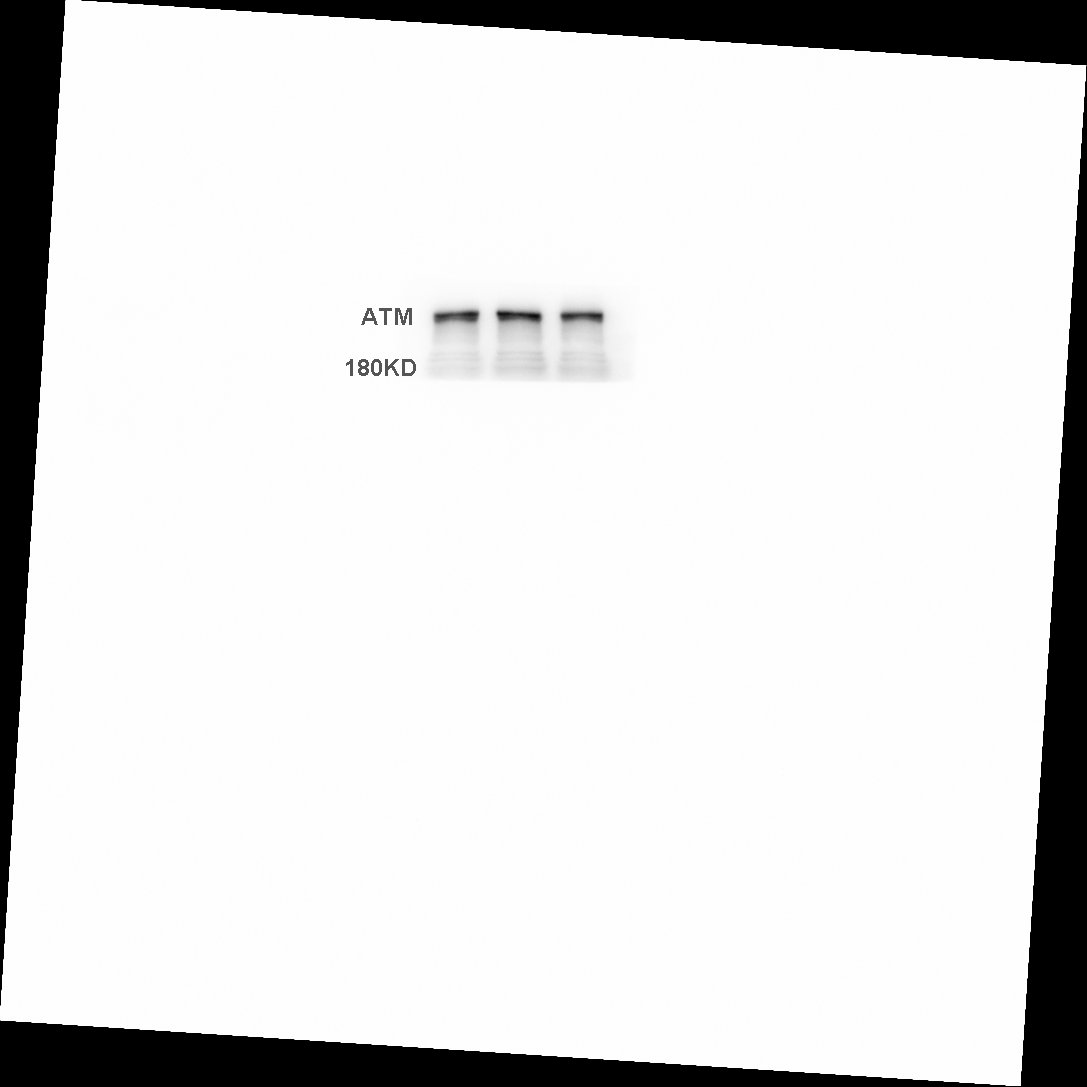

Supplement: Supplementary file 6 — Source Data Fig. 3 [file 44318_2023_3_MOESM6_ESM.zip › Figure3/3a-c/20201011 ATM nc shDC1-1 shDC1-2 VP-16 .jpg]

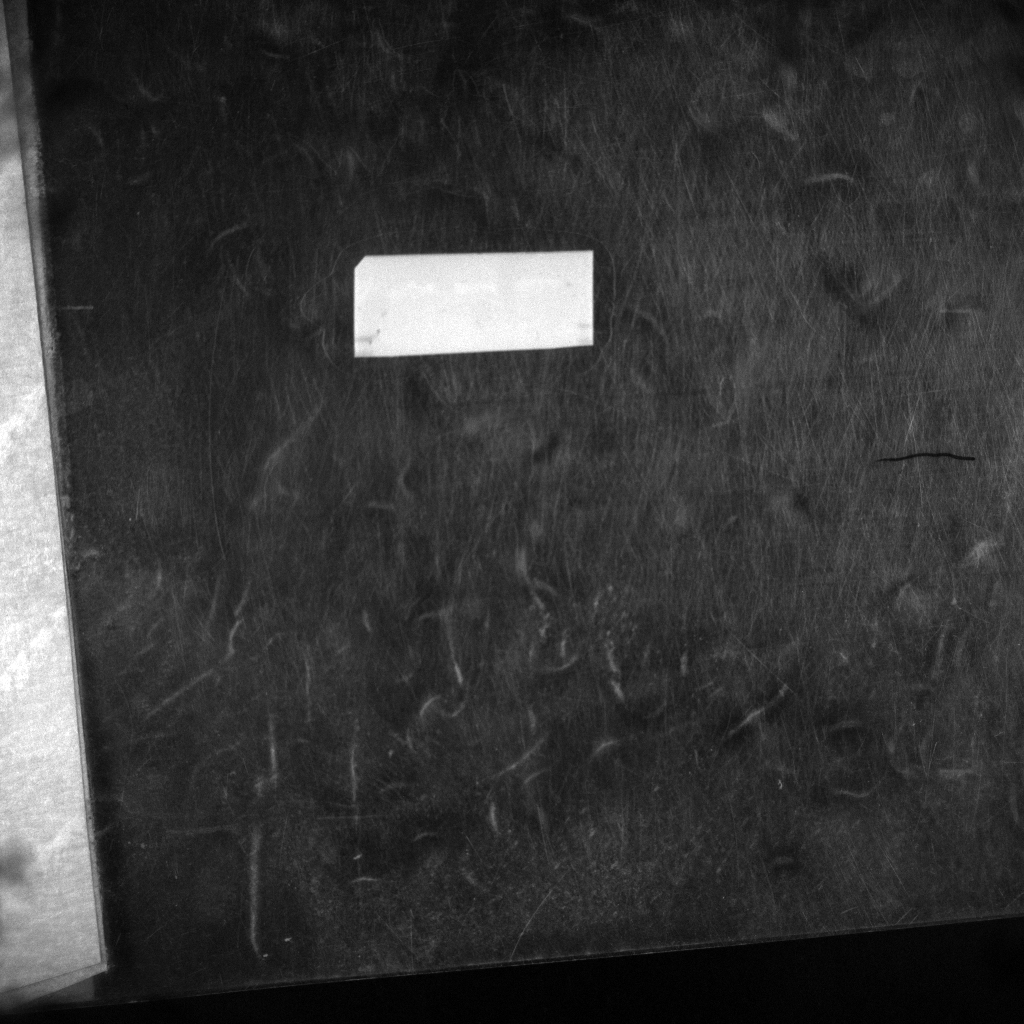

Supplement: Supplementary file 6 — Source Data Fig. 3 [file 44318_2023_3_MOESM6_ESM.zip › Figure3/3a-c/20201011 ATM nc siDC1-1 siDC1-2 VP-16 white .tif]

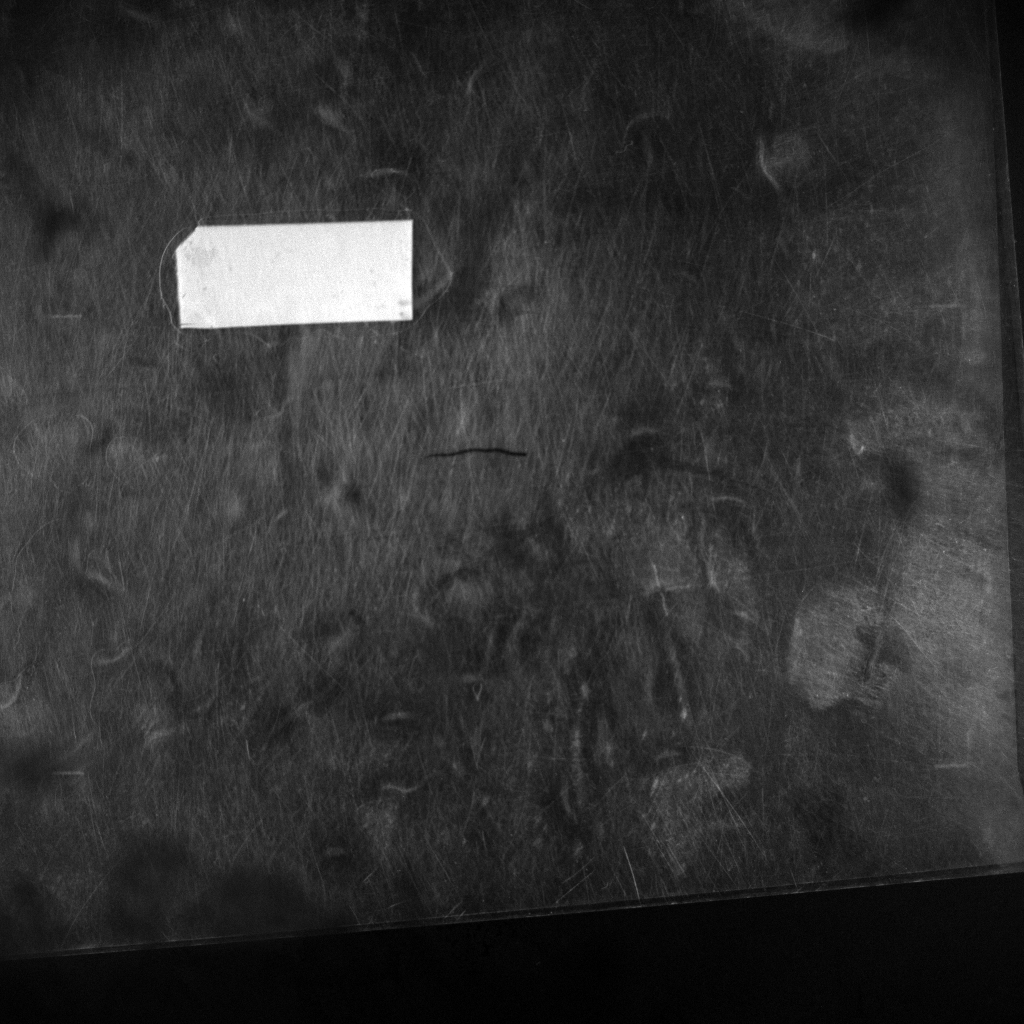

Supplement: Supplementary file 6 — Source Data Fig. 3 [file 44318_2023_3_MOESM6_ESM.zip › Figure3/3a-c/20201012 p-ATM nc siDC1-1 siDC1-2 white.tif]

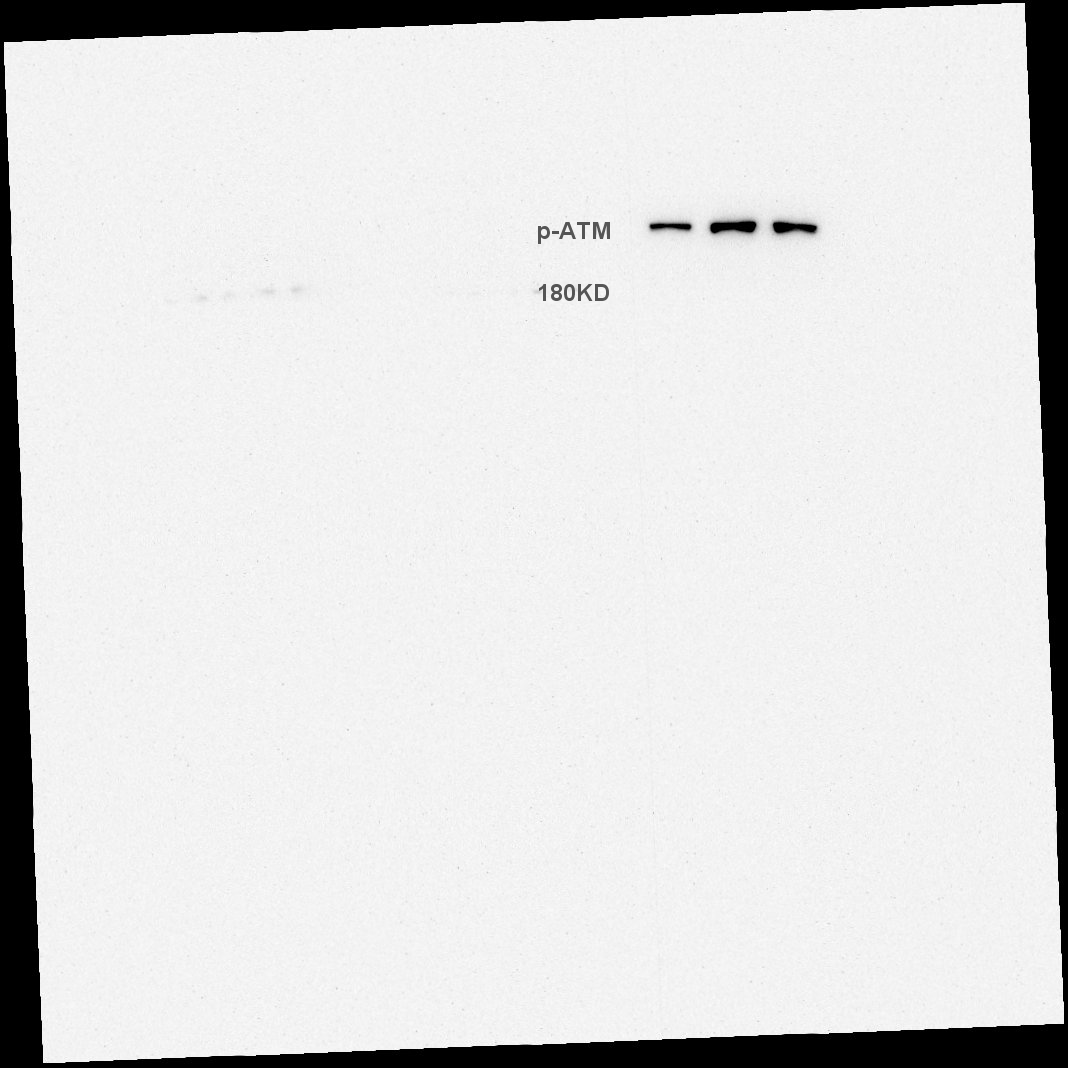

Supplement: Supplementary file 6 — Source Data Fig. 3 [file 44318_2023_3_MOESM6_ESM.zip › Figure3/3a-c/20201012 p-ATM nc siDC1-1 siDC1-2 VP-16 .tif]

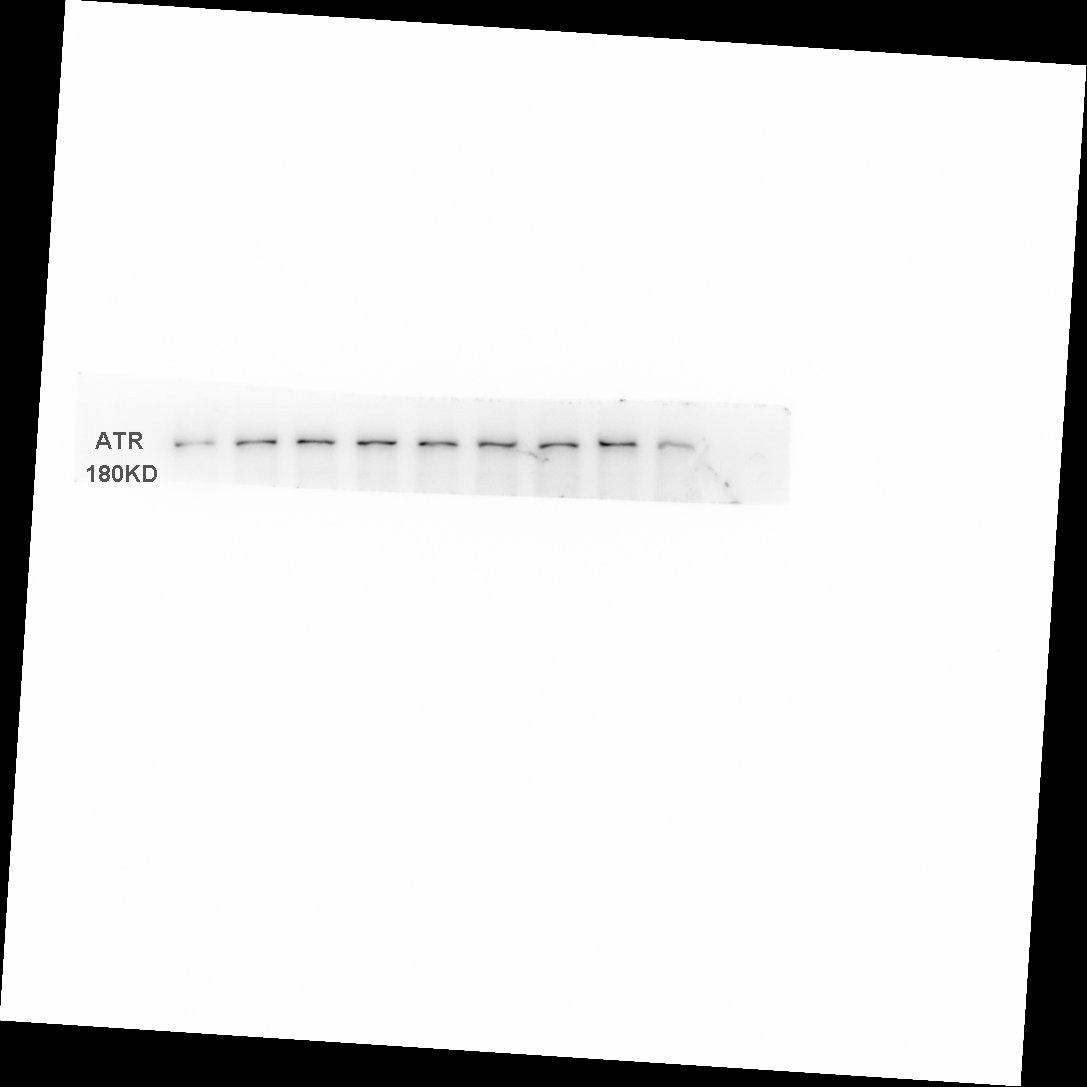

Supplement: Supplementary file 6 — Source Data Fig. 3 [file 44318_2023_3_MOESM6_ESM.zip › Figure3/3d-f/ATR DMSO NC siDC1-1 -2 siDF2-1 -2 siIGF2BP1 2 3 .jpg]

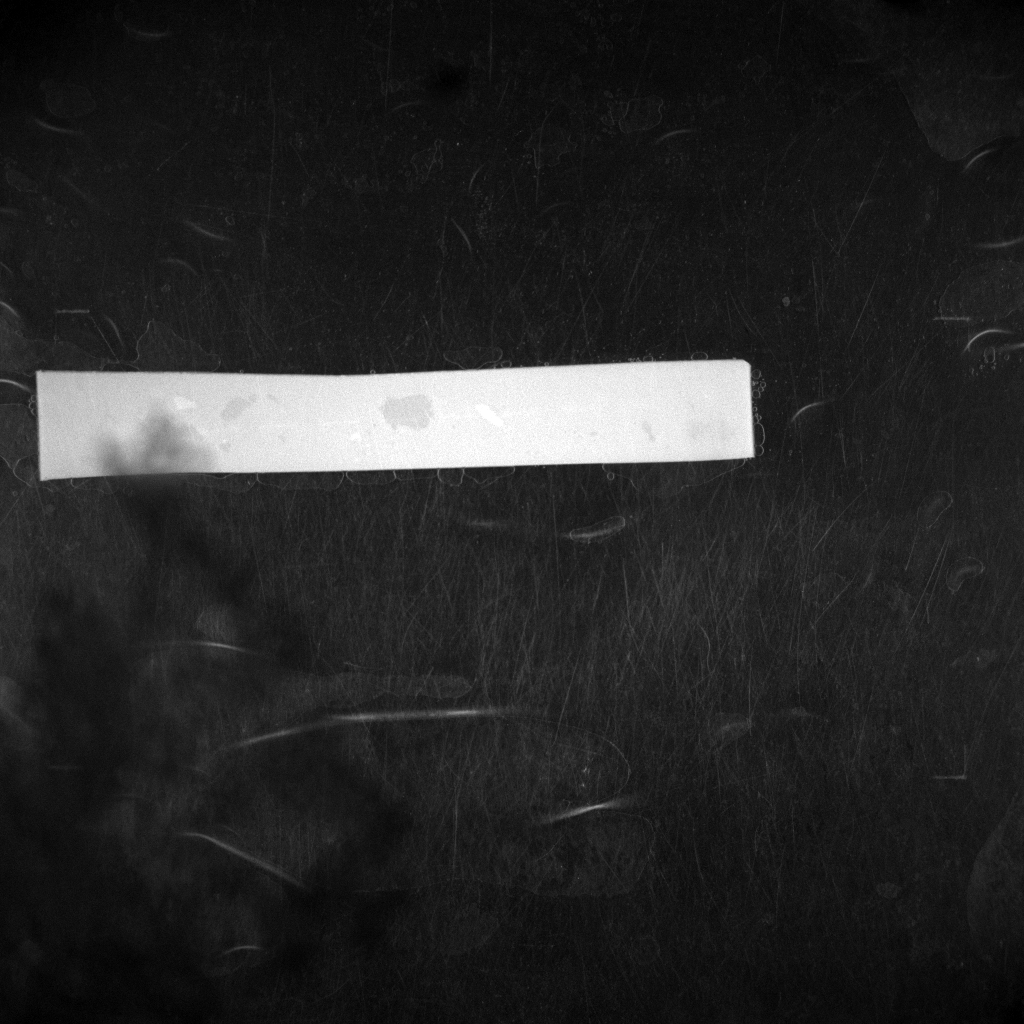

Supplement: Supplementary file 6 — Source Data Fig. 3 [file 44318_2023_3_MOESM6_ESM.zip › Figure3/3d-f/ATR DMSO NC siDC1-1 -2 siDF2-1 -2 siIGF2BP1 2 3 W.tif]

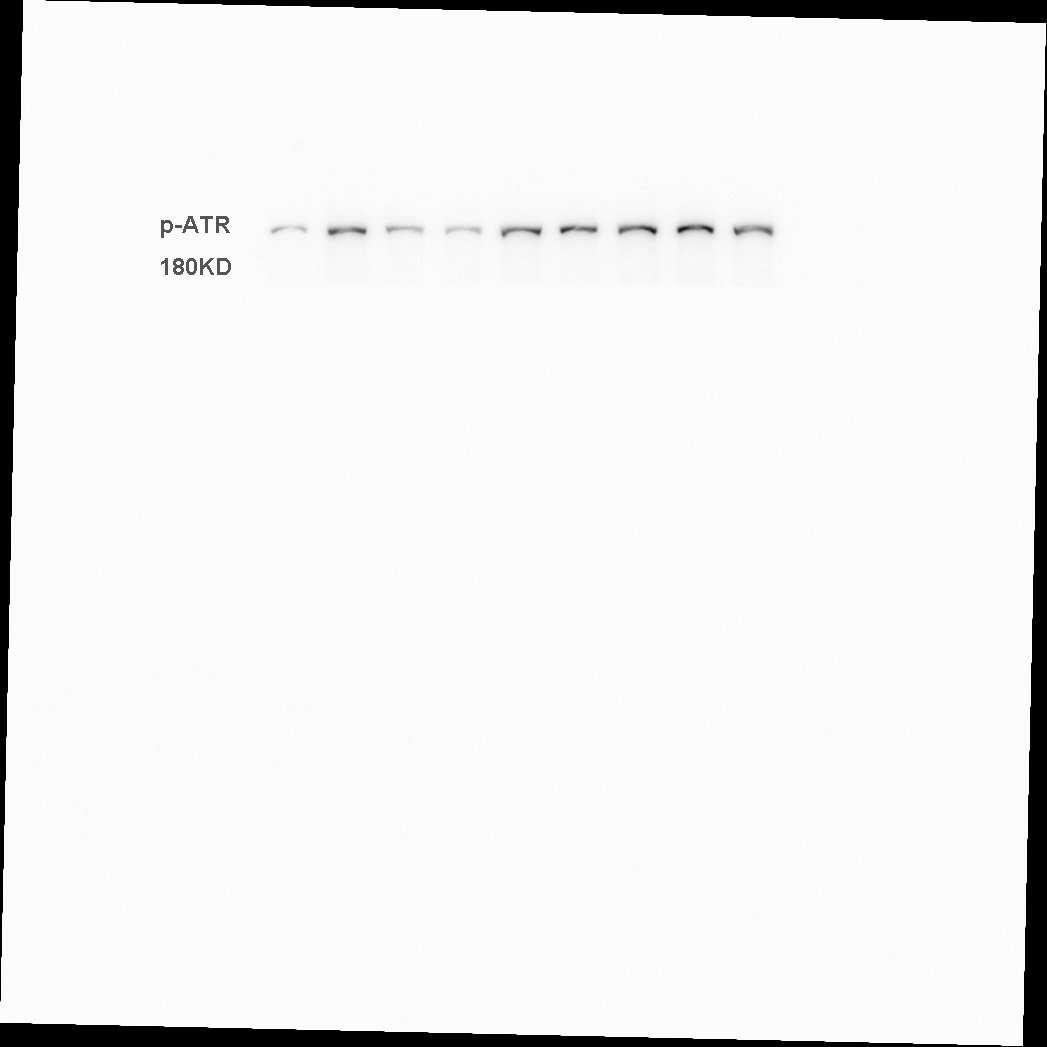

Supplement: Supplementary file 6 — Source Data Fig. 3 [file 44318_2023_3_MOESM6_ESM.zip › Figure3/3d-f/p-ATR DMSO NC siDC1-1 -2 siDF2-1 -2 siIGF2BP1 2 3 .jpg]

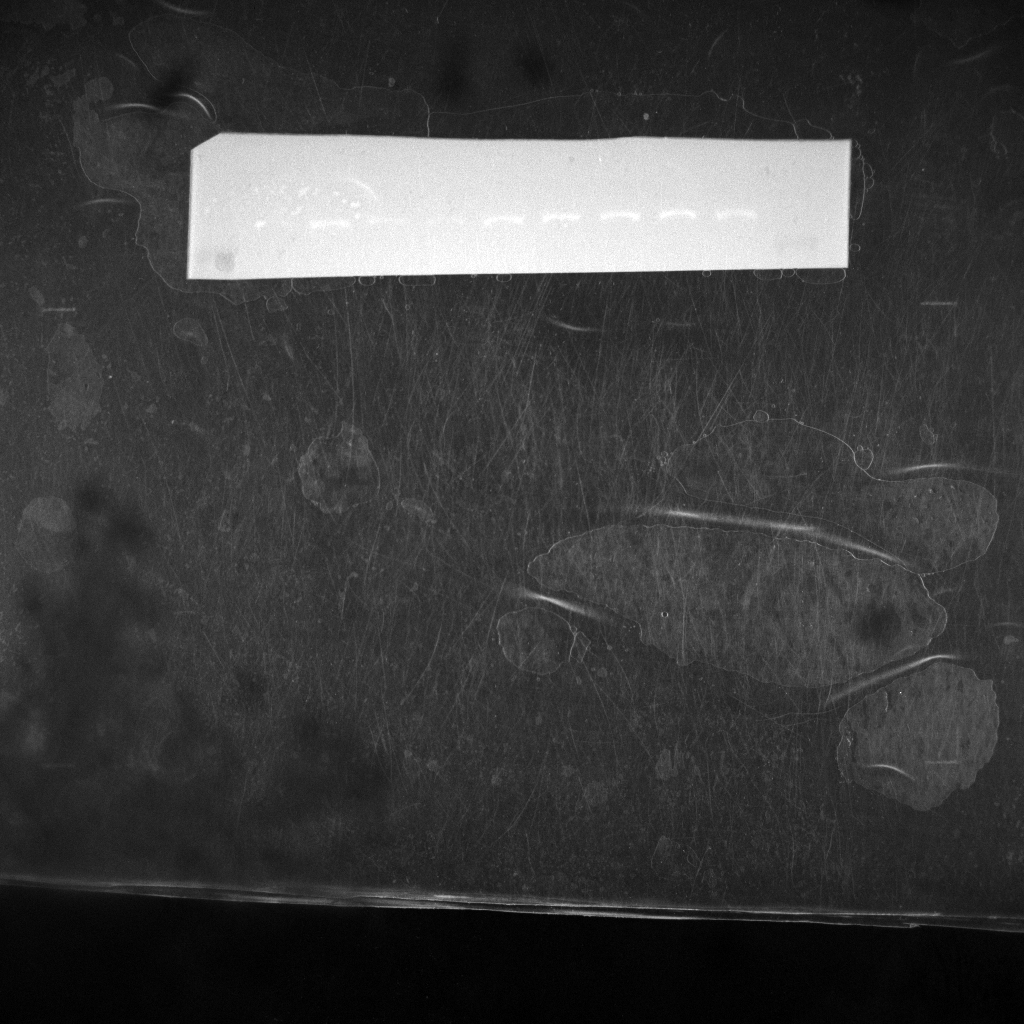

Supplement: Supplementary file 6 — Source Data Fig. 3 [file 44318_2023_3_MOESM6_ESM.zip › Figure3/3d-f/p-ATR DMSO NC siDC1-1 -2 siDF2-1 -2 siIGF2BP1 2 3 W .tif]

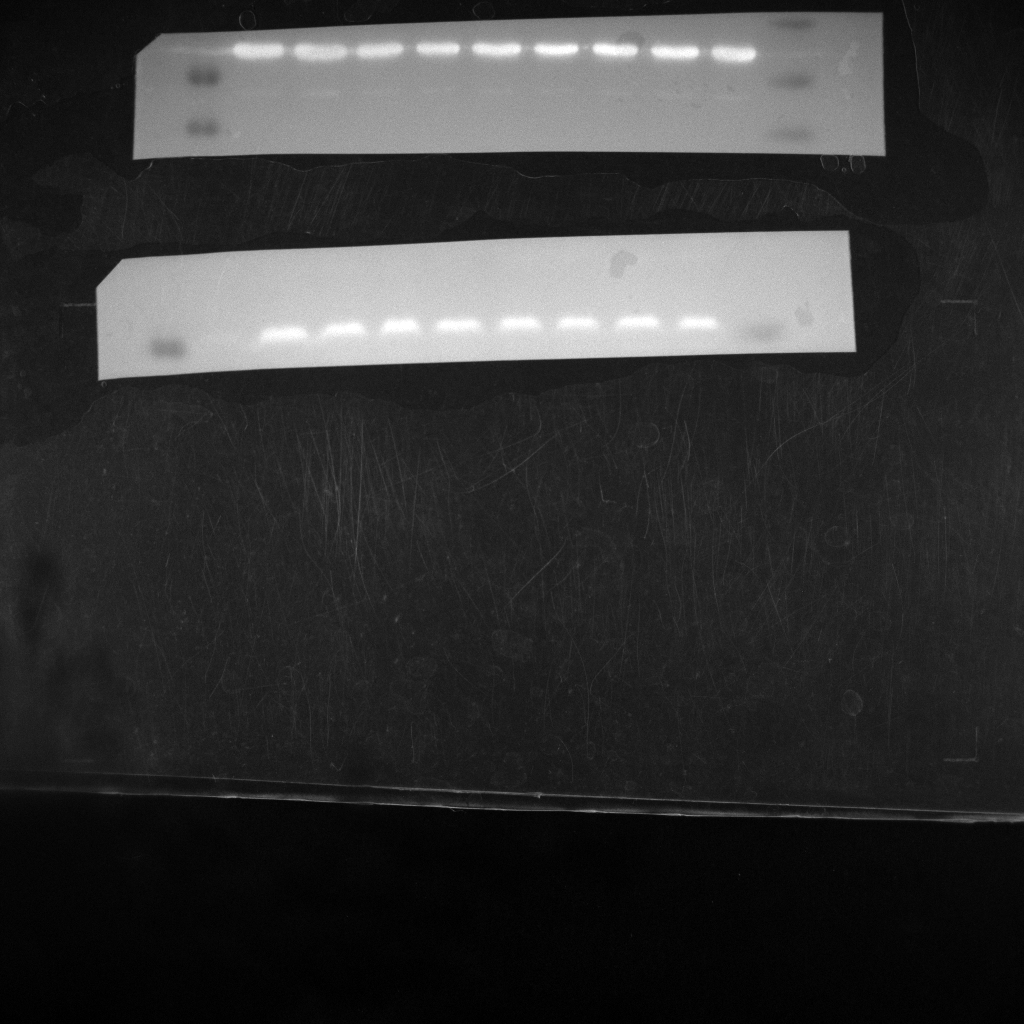

Supplement: Supplementary file 6 — Source Data Fig. 3 [file 44318_2023_3_MOESM6_ESM.zip › Figure3/3d-f/up GAPDH down uh2ax NC-DMSO NC-VP16 siDC1-1 -2 siDF2-1 -2 siIGFBP1 BP2 BP3 w .tif]

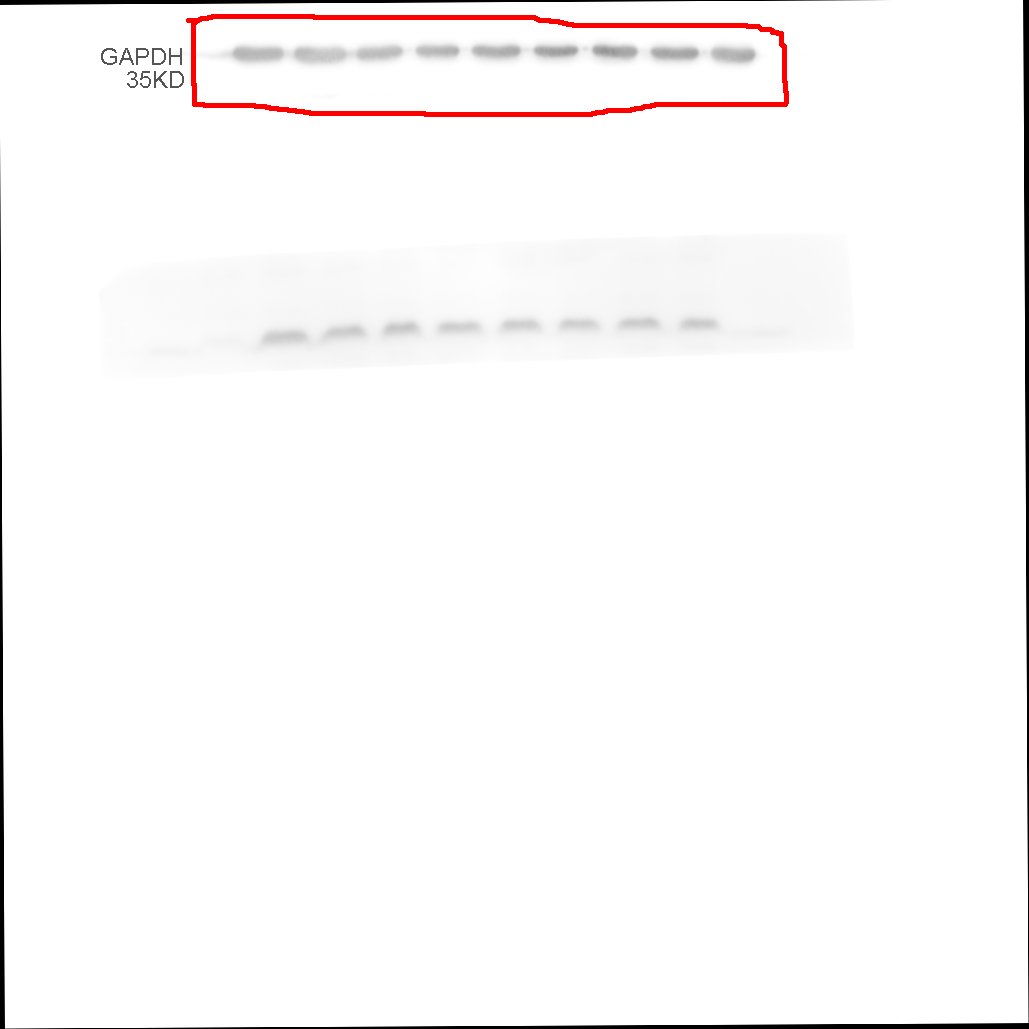

Supplement: Supplementary file 6 — Source Data Fig. 3 [file 44318_2023_3_MOESM6_ESM.zip › Figure3/3d-f/up GAPDH down yh2ax NC-DMSO NC-VP16 siDC1-1 -2 siDF2-1 -2 siIGFBP1 BP2 BP3.jpg]

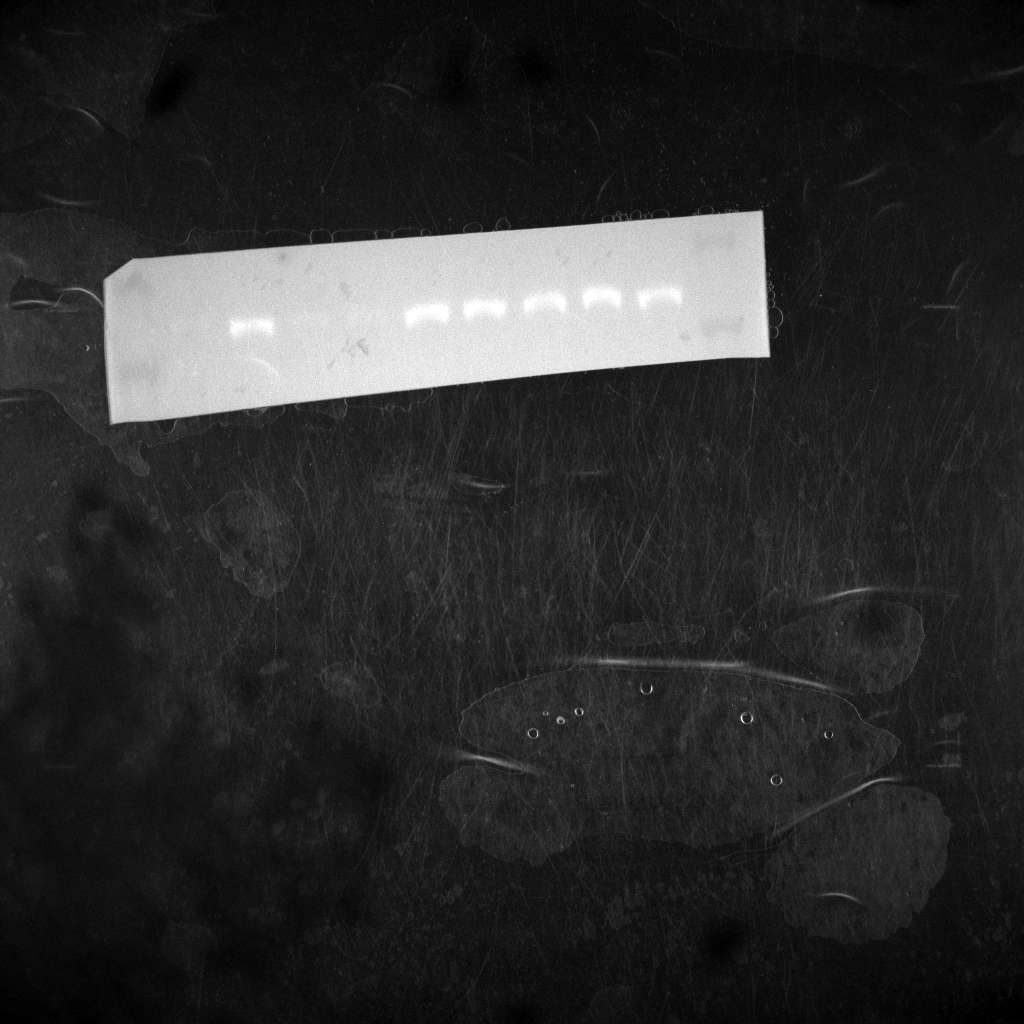

Supplement: Supplementary file 6 — Source Data Fig. 3 [file 44318_2023_3_MOESM6_ESM.zip › Figure3/3d-f/YTHDC1 DMSO NC siDC1-1 -2 siDF2-1 -2 siIGF2BP1 2 3 W.tif]

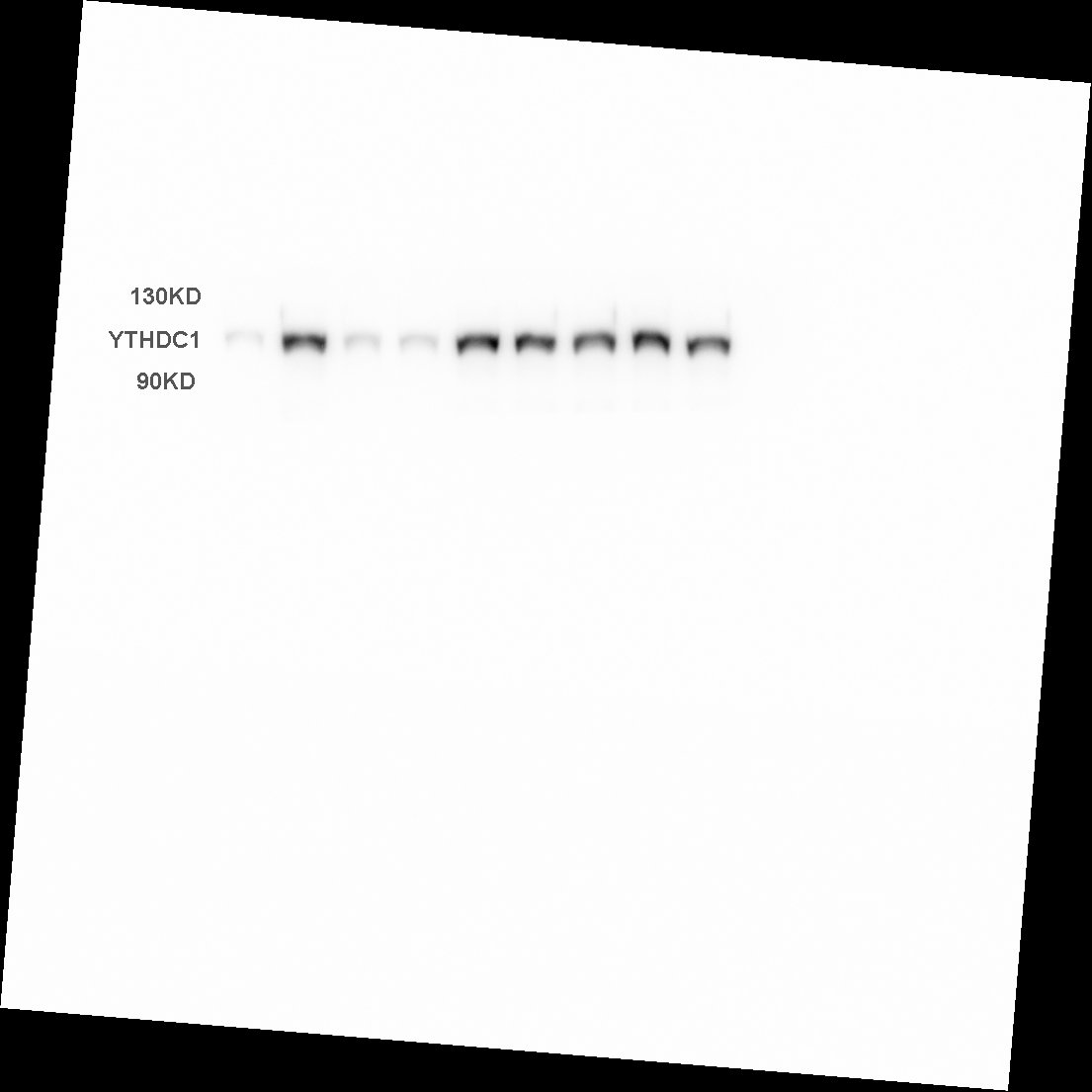

Supplement: Supplementary file 6 — Source Data Fig. 3 [file 44318_2023_3_MOESM6_ESM.zip › Figure3/3d-f/YTHDC1 DMSO NC siDC1-1 -2 siDF2-1 -2 siIGF2BP1 2 3.jpg]

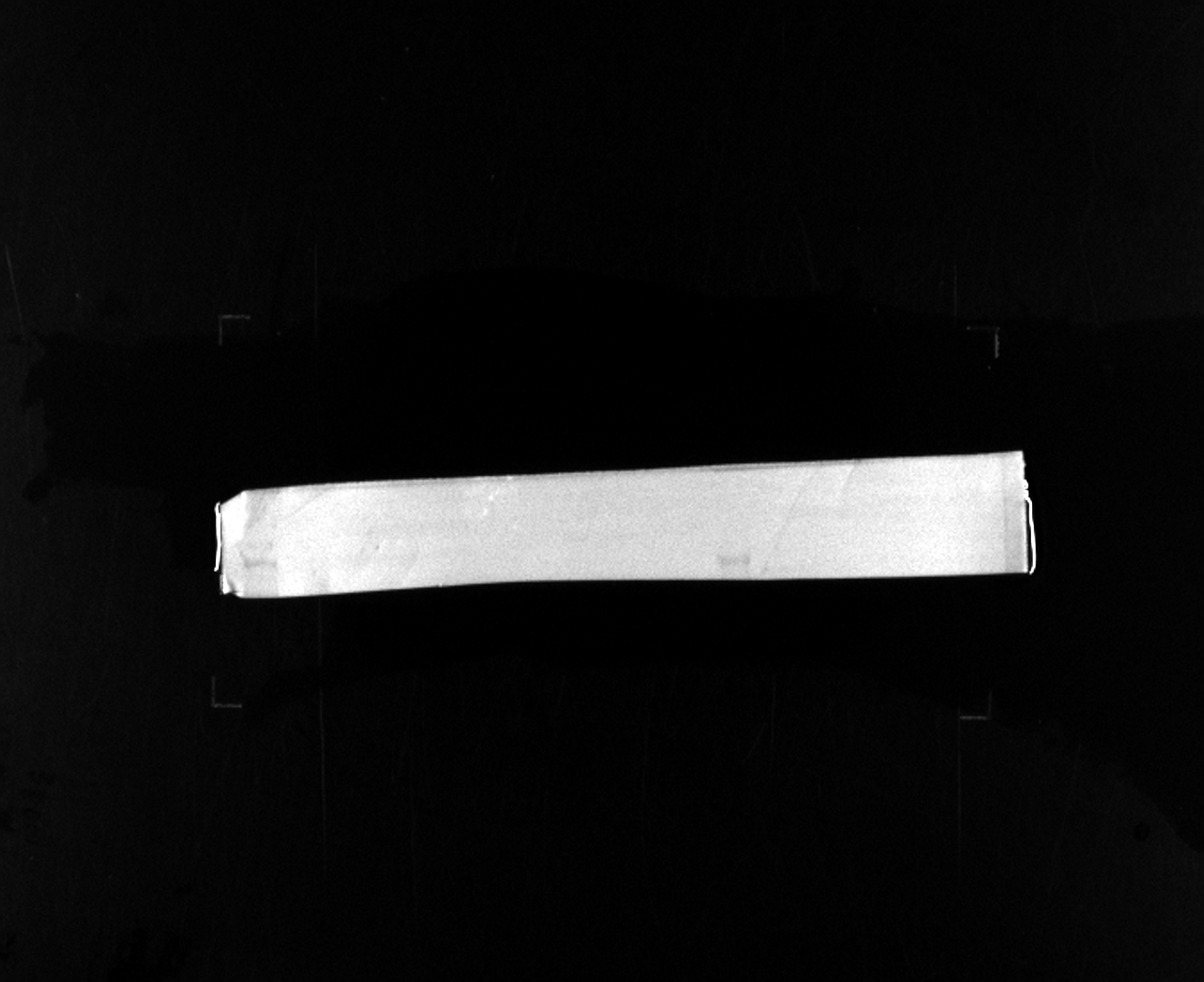

Supplement: Supplementary file 6 — Source Data Fig. 3 [file 44318_2023_3_MOESM6_ESM.zip › Figure3/3g-i/atr and p-atr nc shm3 14 wtap m hct116 nc shx-2 -4 shy-3 -4 w.Tif]

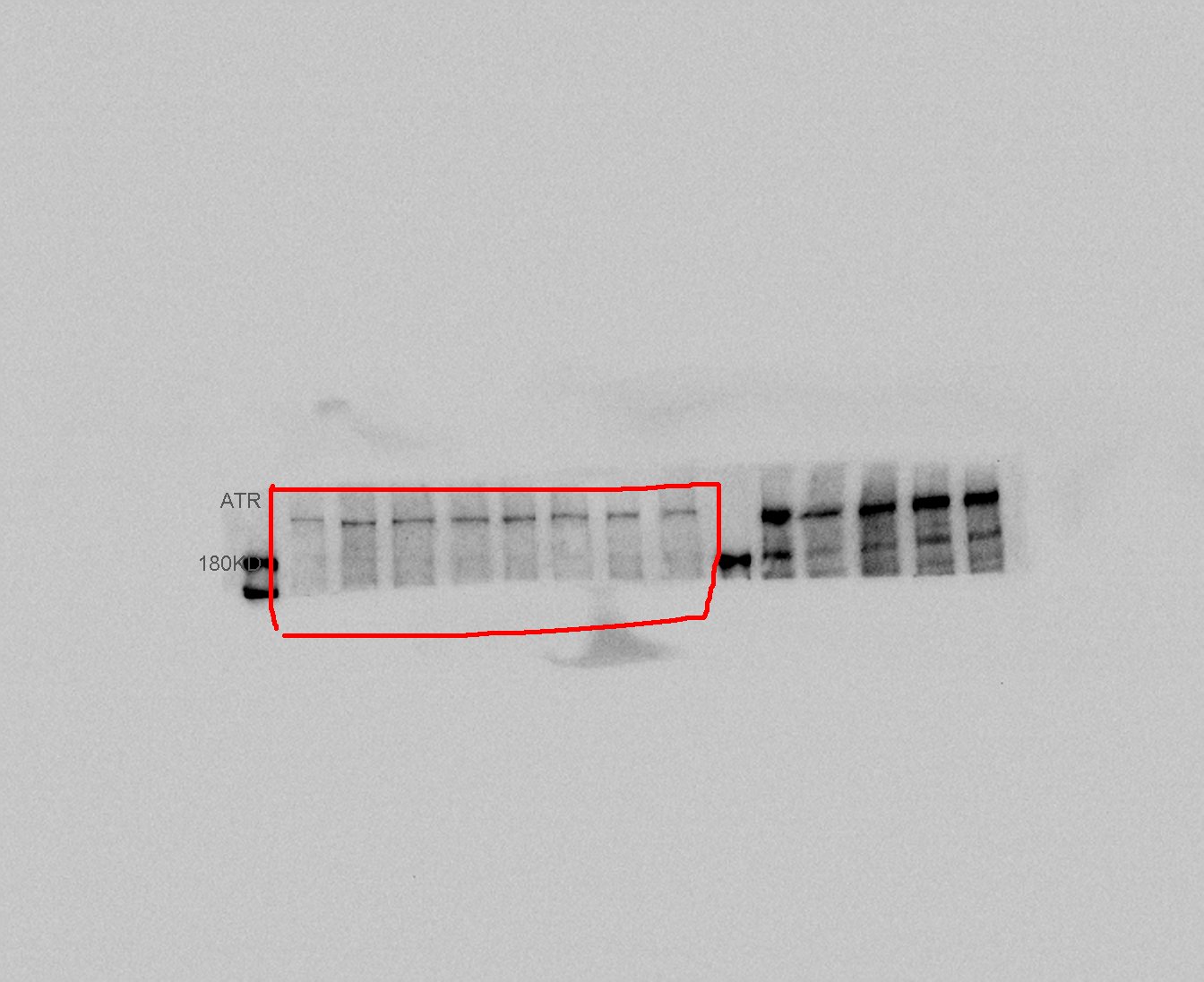

Supplement: Supplementary file 6 — Source Data Fig. 3 [file 44318_2023_3_MOESM6_ESM.zip › Figure3/3g-i/atr and p-atr nc shm3 14 wtap m hct116 nc shx-2 -4 shy-3 -4 VP-16 .jpg]

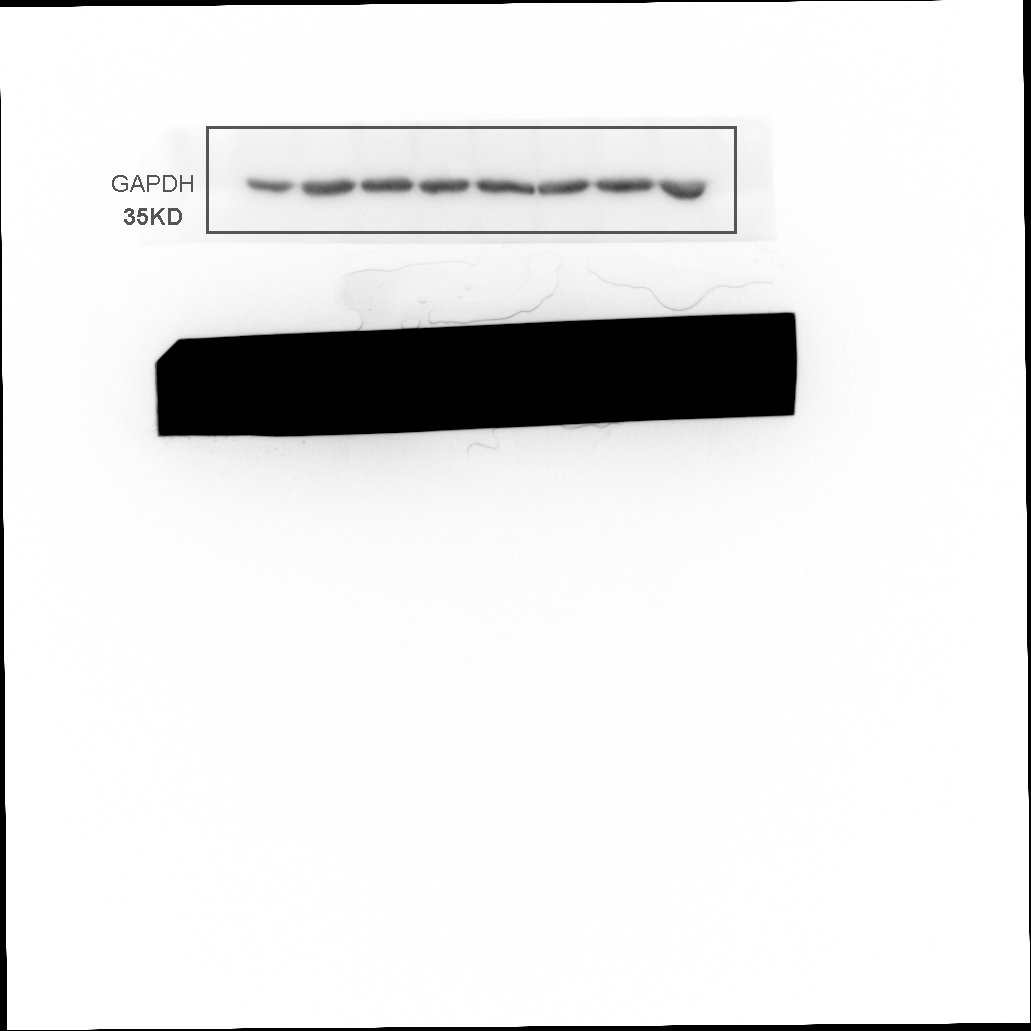

Supplement: Supplementary file 6 — Source Data Fig. 3 [file 44318_2023_3_MOESM6_ESM.zip › Figure3/3g-i/gapdh nc-DMSO NC siM3-1 -2 siM14-1 -2 siWTAP-1 -2 (VP16) .jpg]

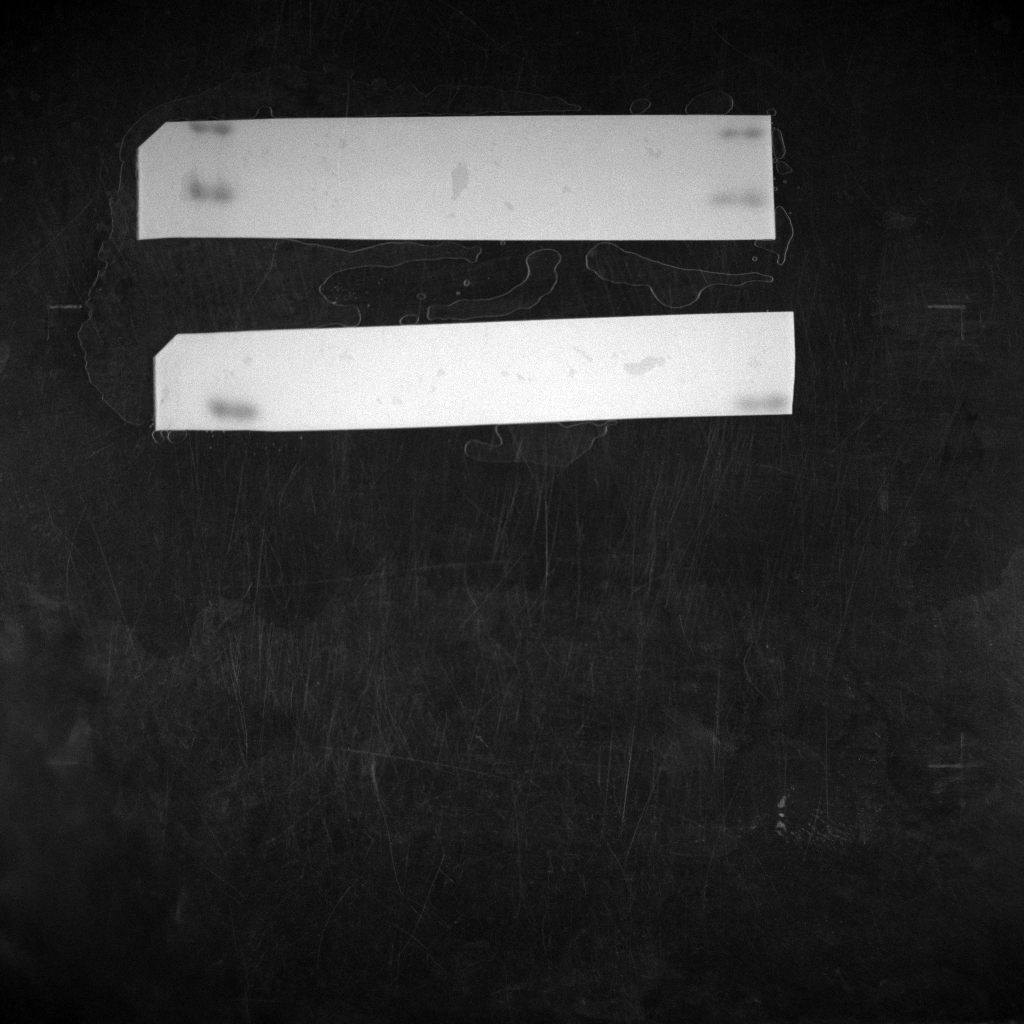

Supplement: Supplementary file 6 — Source Data Fig. 3 [file 44318_2023_3_MOESM6_ESM.zip › Figure3/3g-i/gapdh nc-DMSO NC siM3-1 -2 siM14-1 -2 siWTAP-1 -2 (VP16) W .tif]

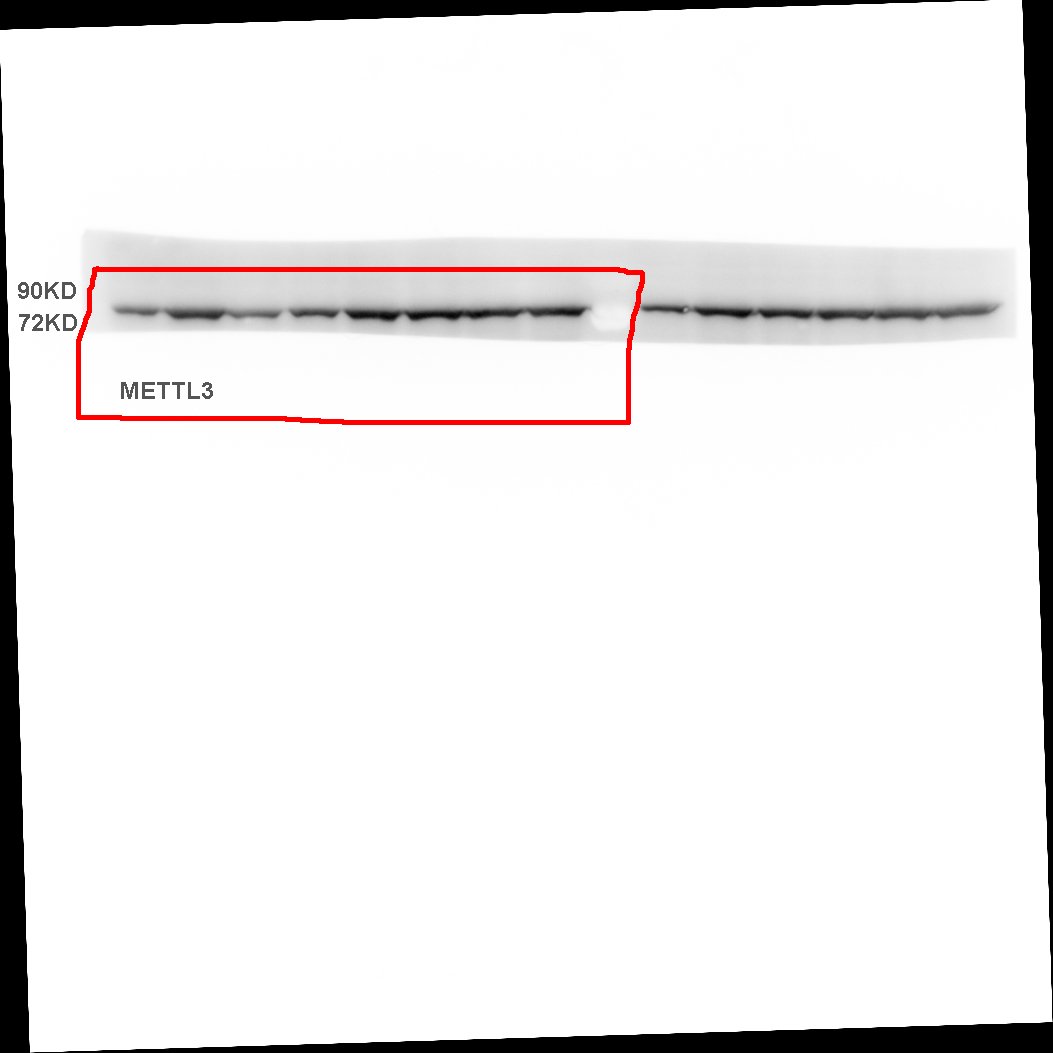

Supplement: Supplementary file 6 — Source Data Fig. 3 [file 44318_2023_3_MOESM6_ESM.zip › Figure3/3g-i/M3 nc-dmso nc siM3-1 -2 siM14-1 -2 siW-1 -2 (vp16) M nc-DMSO NC siF-1 siF-2 siA-1 -2 .jpg]

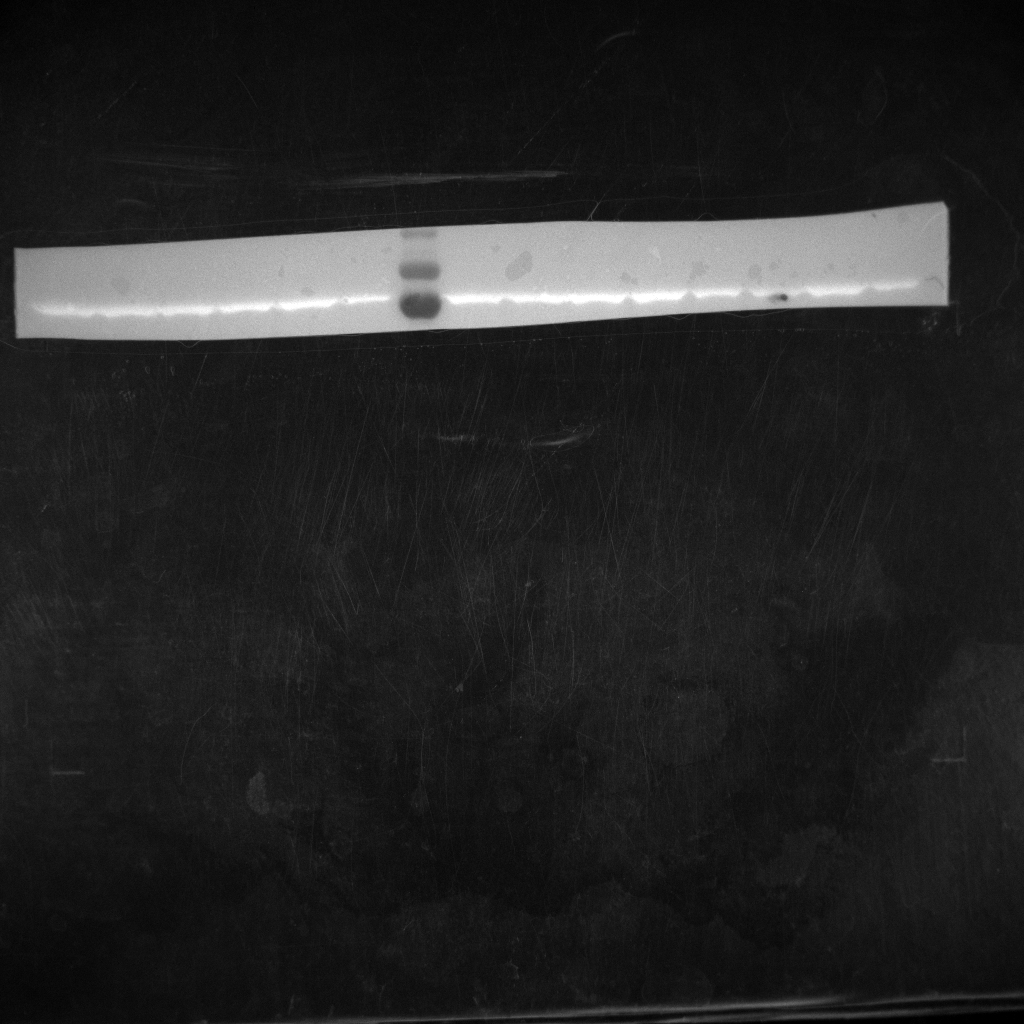

Supplement: Supplementary file 6 — Source Data Fig. 3 [file 44318_2023_3_MOESM6_ESM.zip › Figure3/3g-i/M3 nc-dmso nc siM3-1 -2 siM14-1 -2 siW-1 -2 (vp16) M nc-DMSO NC siF-1 siF-2 siA-1 -2 W .tif]

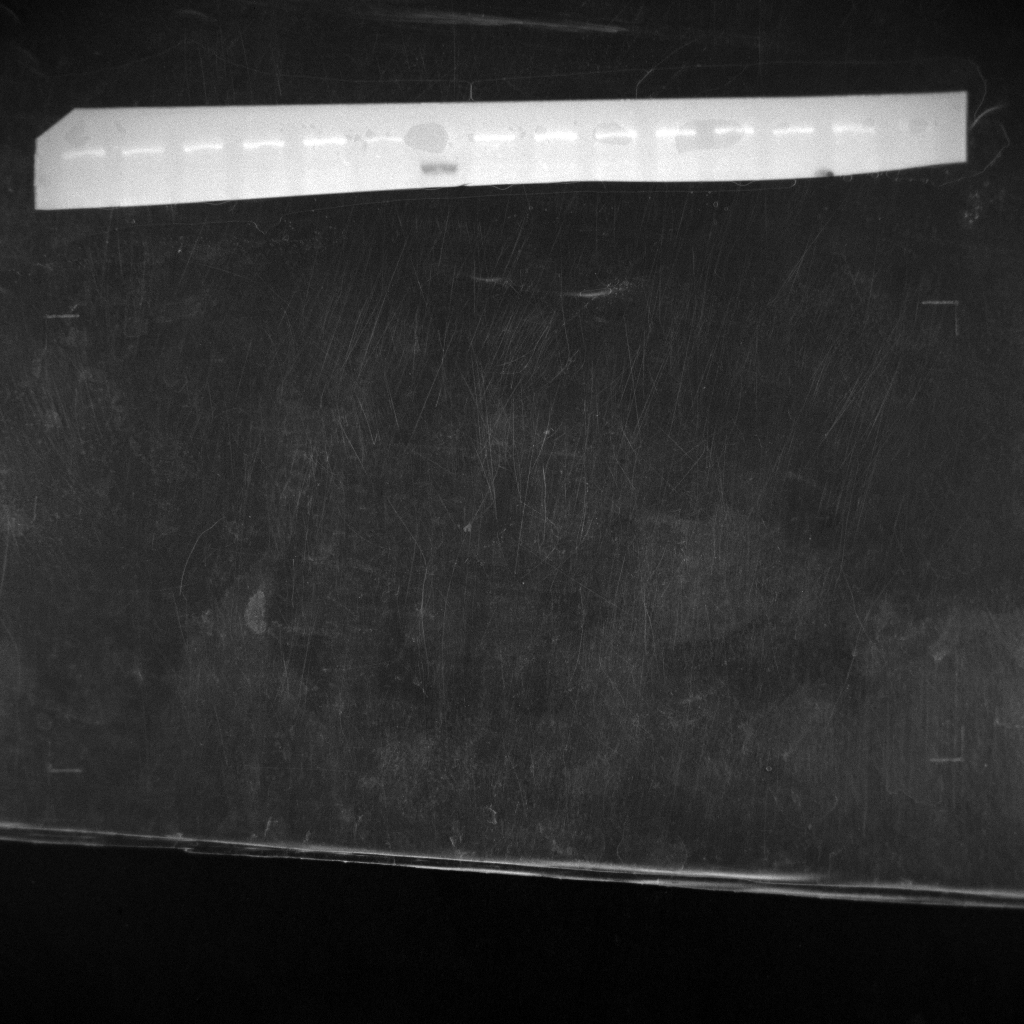

Supplement: Supplementary file 6 — Source Data Fig. 3 [file 44318_2023_3_MOESM6_ESM.zip › Figure3/3g-i/p-ATR nc-dmso nc siM3-1 -2 siM14-1 -2 siW-1 -2 (vp16) M nc-DMSO NC siF-1 siF-2 siA-1 -2 W .tif]

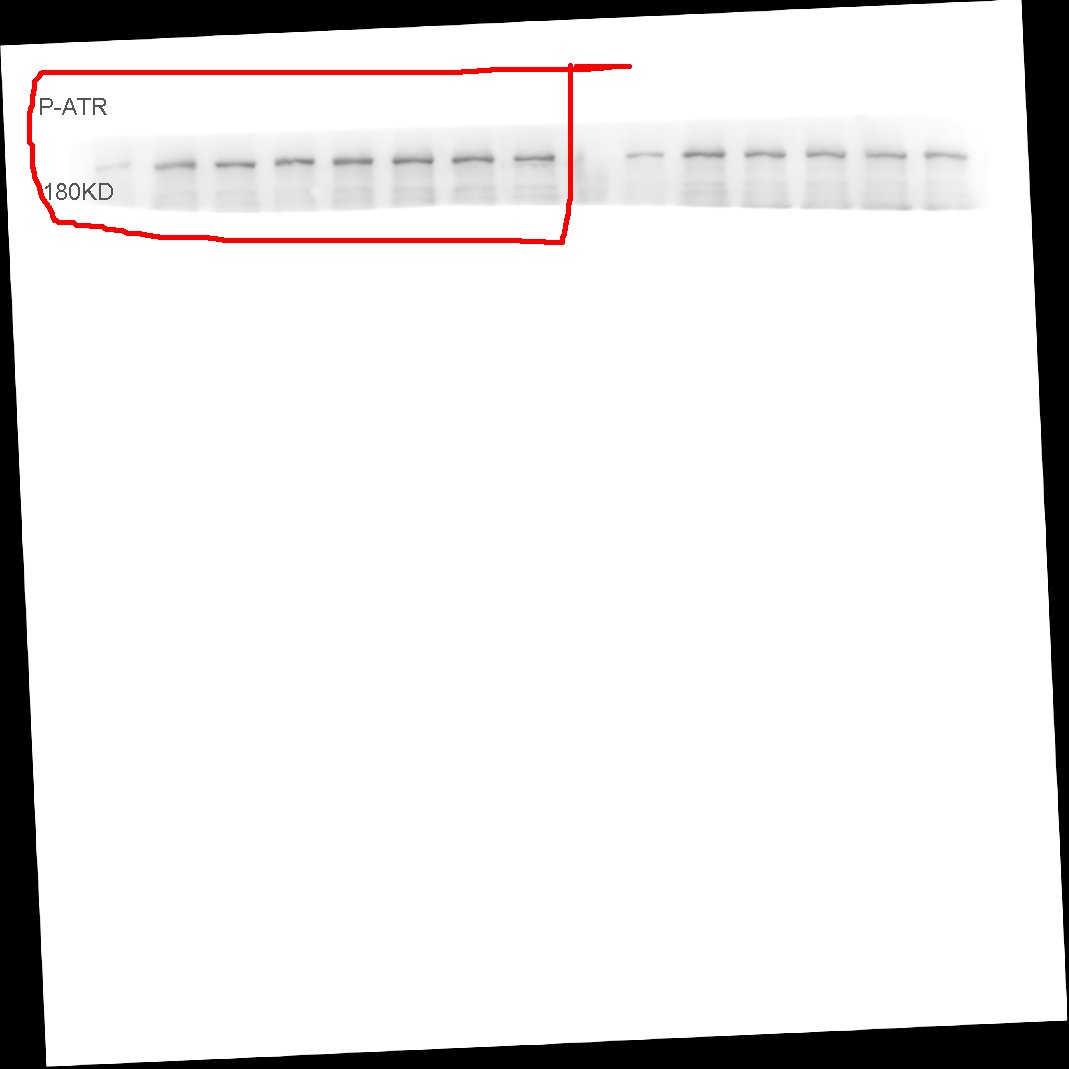

Supplement: Supplementary file 6 — Source Data Fig. 3 [file 44318_2023_3_MOESM6_ESM.zip › Figure3/3g-i/p-ATR nc-dmso nc siM3-1 -2 siM14-1 -2 siW-1 -2 (vp16) M nc-DMSO NC siF-1 siF-2 siA-1 -2.jpg]

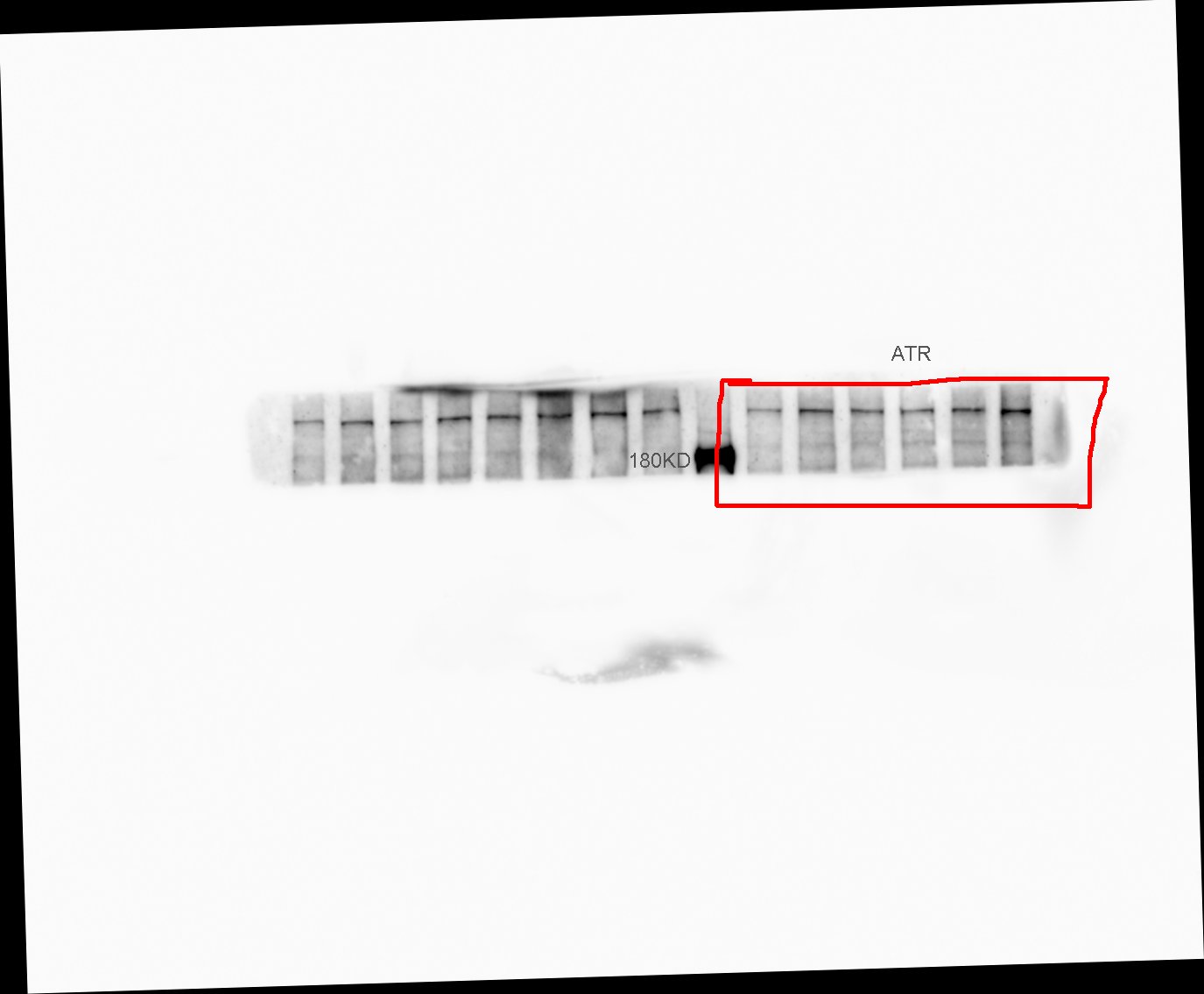

Supplement: Supplementary file 6 — Source Data Fig. 3 [file 44318_2023_3_MOESM6_ESM.zip › Figure3/3j-l/atr nc shm3 14 wtap m nc sifto alk .jpg]

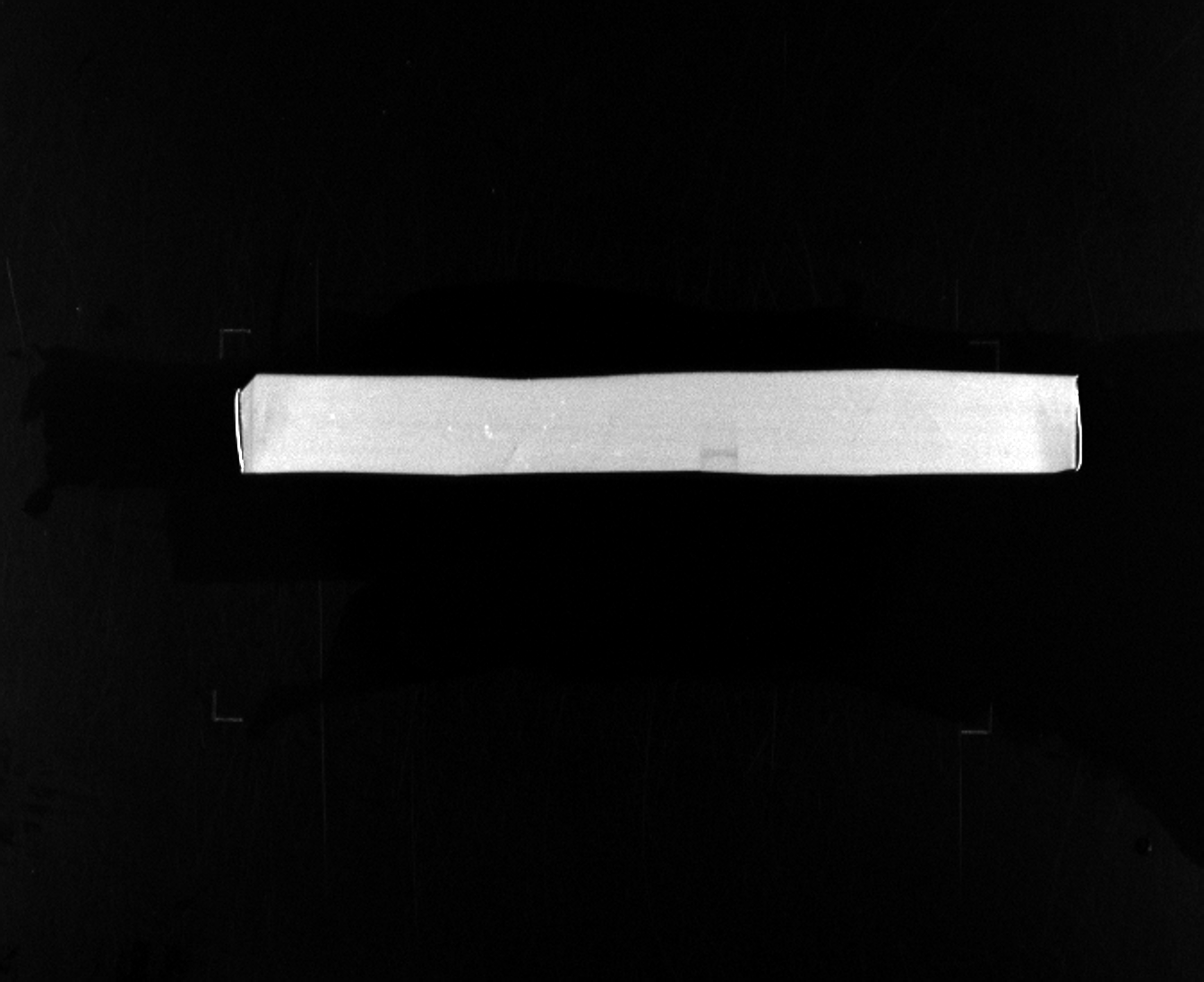

Supplement: Supplementary file 6 — Source Data Fig. 3 [file 44318_2023_3_MOESM6_ESM.zip › Figure3/3j-l/atr nc shm3 14 wtap m nc sifto alk w.Tif]

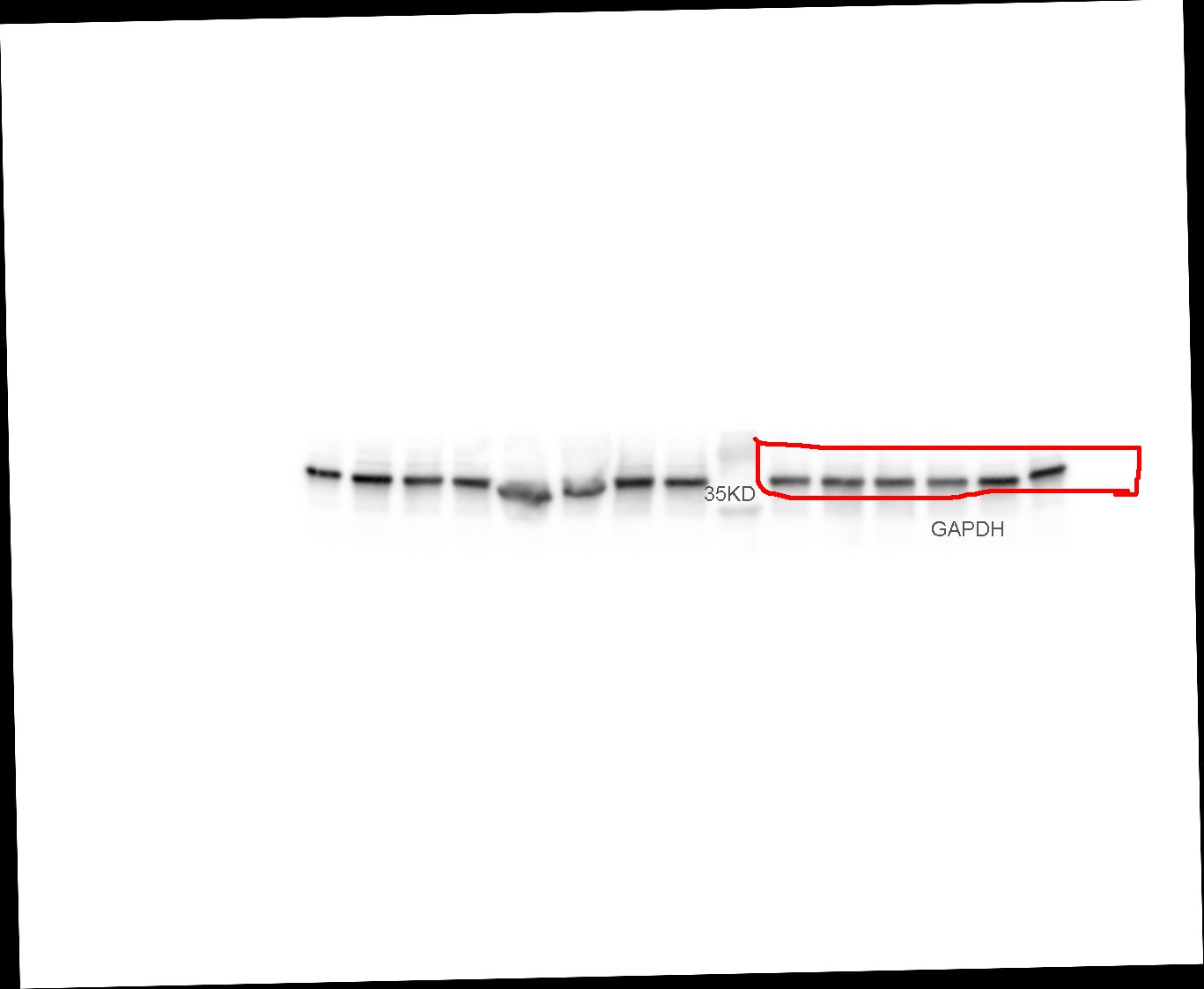

Supplement: Supplementary file 6 — Source Data Fig. 3 [file 44318_2023_3_MOESM6_ESM.zip › Figure3/3j-l/gapdh nc shm3 14 wtap m nc sifto alk .jpg]

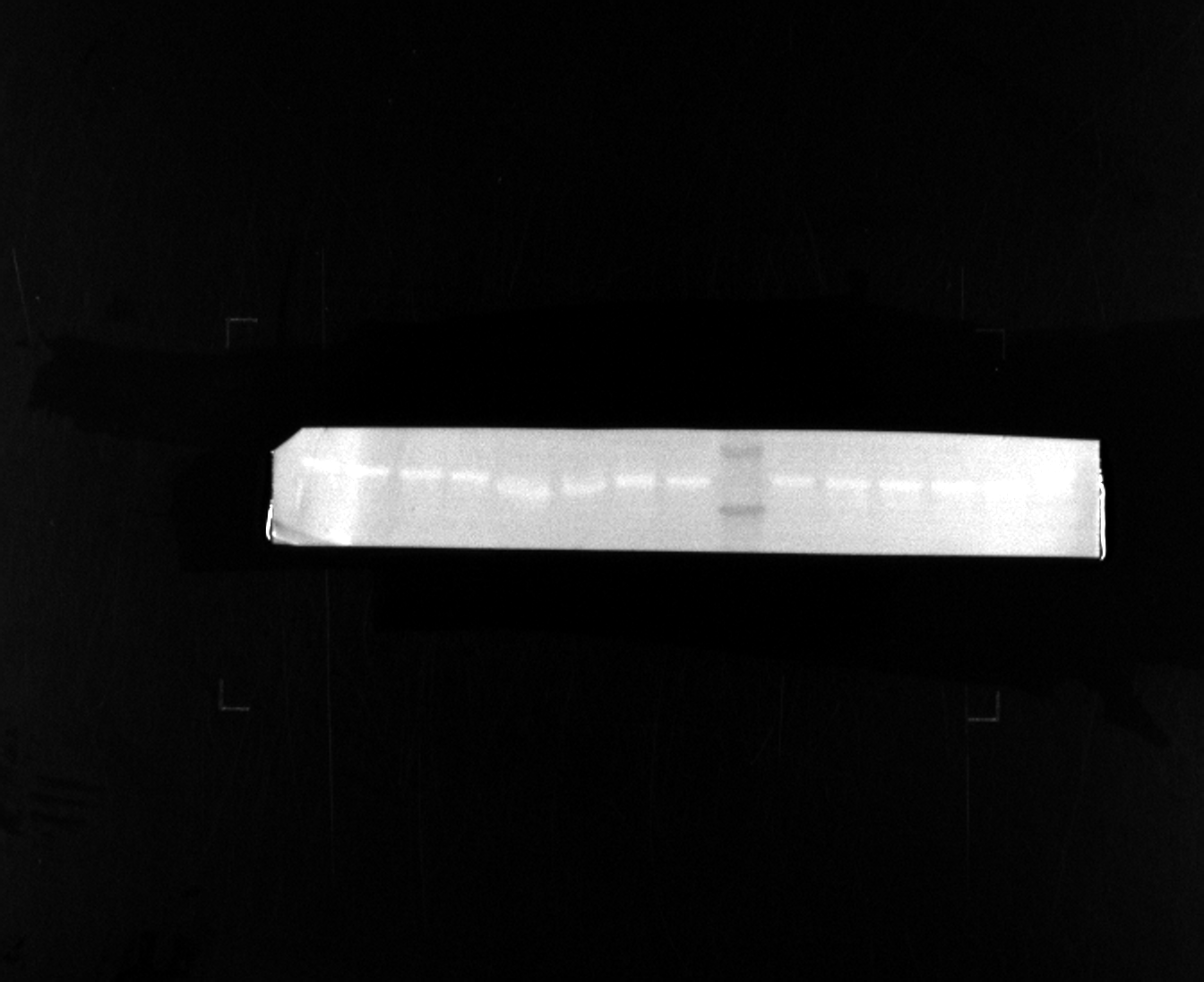

Supplement: Supplementary file 6 — Source Data Fig. 3 [file 44318_2023_3_MOESM6_ESM.zip › Figure3/3j-l/gapdh nc shm3 14 wtap m nc sifto alk w.Tif]

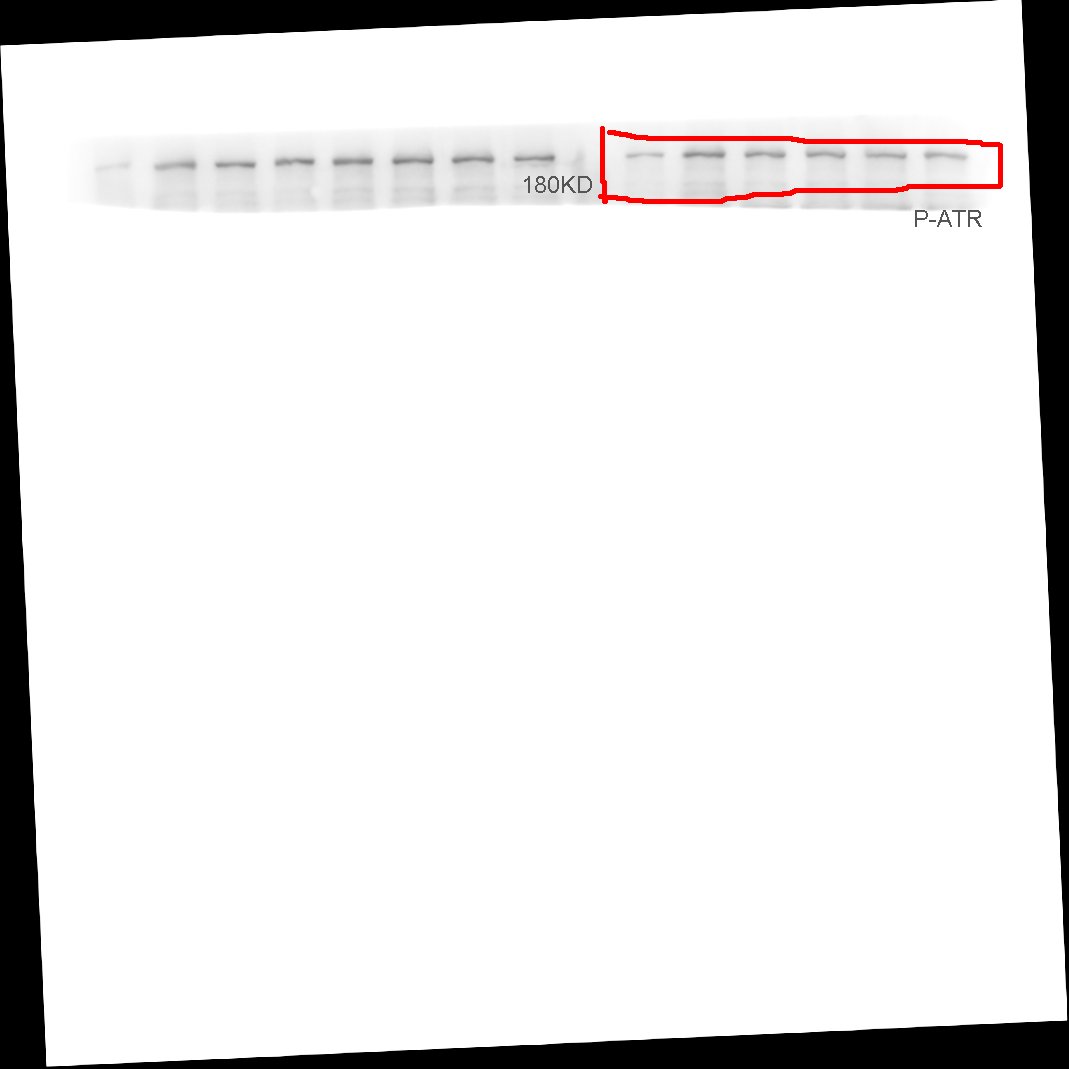

Supplement: Supplementary file 6 — Source Data Fig. 3 [file 44318_2023_3_MOESM6_ESM.zip › Figure3/3j-l/p-ATR nc-dmso nc siM3-1 -2 siM14-1 -2 siW-1 -2 (vp16) M nc-DMSO NC siF-1 siF-2 siA-1 -2.jpg]

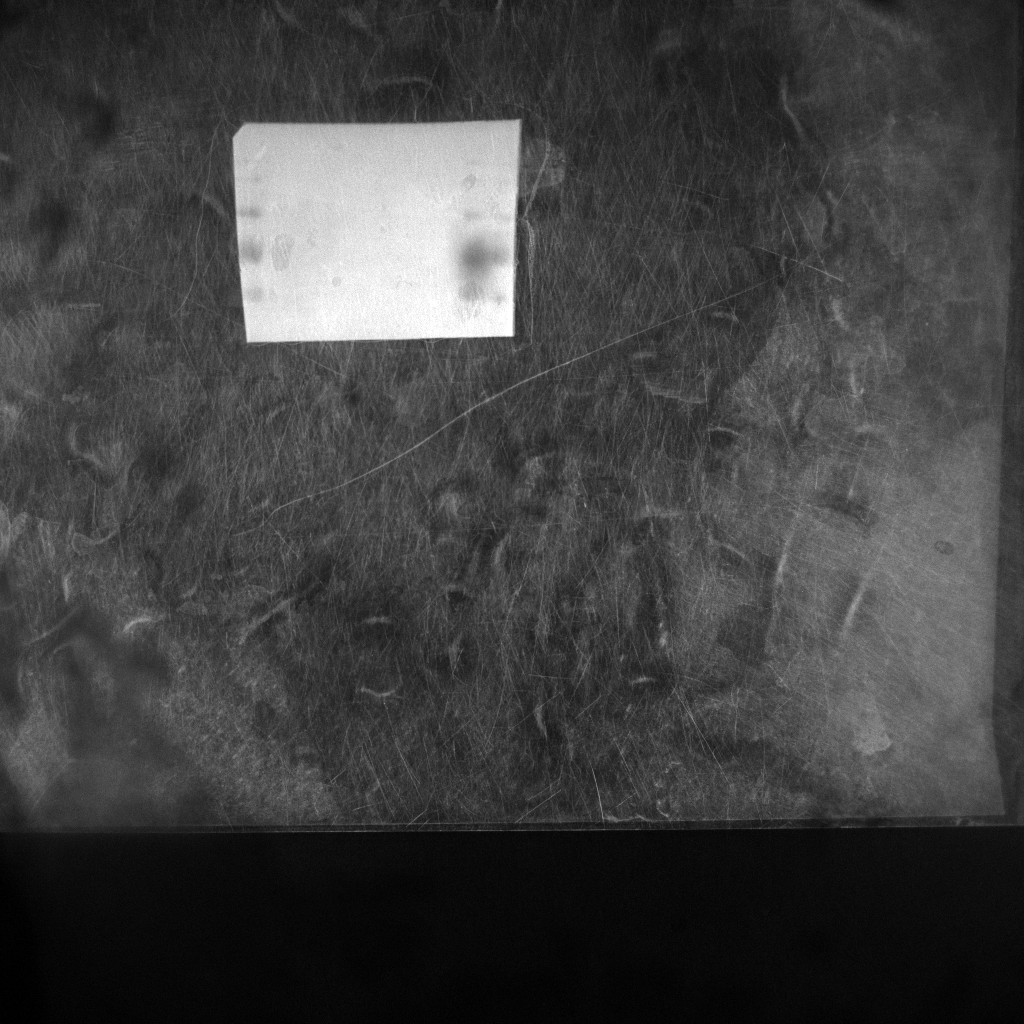

Supplement: Supplementary file 6 — Source Data Fig. 3 [file 44318_2023_3_MOESM6_ESM.zip › Figure3/3m-o/20210112 GAPDH nc shDC1-1 (V WT MUT) white .tif]

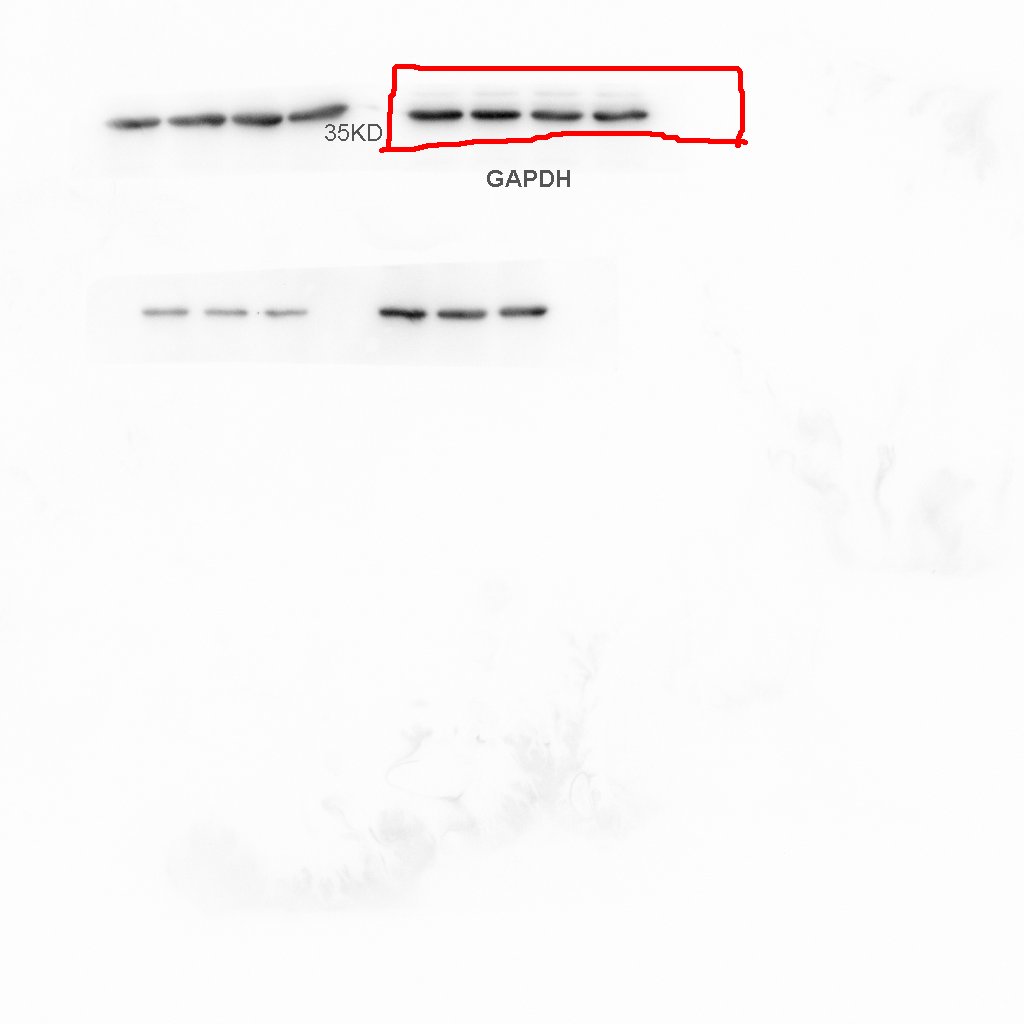

Supplement: Supplementary file 6 — Source Data Fig. 3 [file 44318_2023_3_MOESM6_ESM.zip › Figure3/3m-o/20210112 GAPDH nc shDC1-1 (V WT MUT) .jpg]

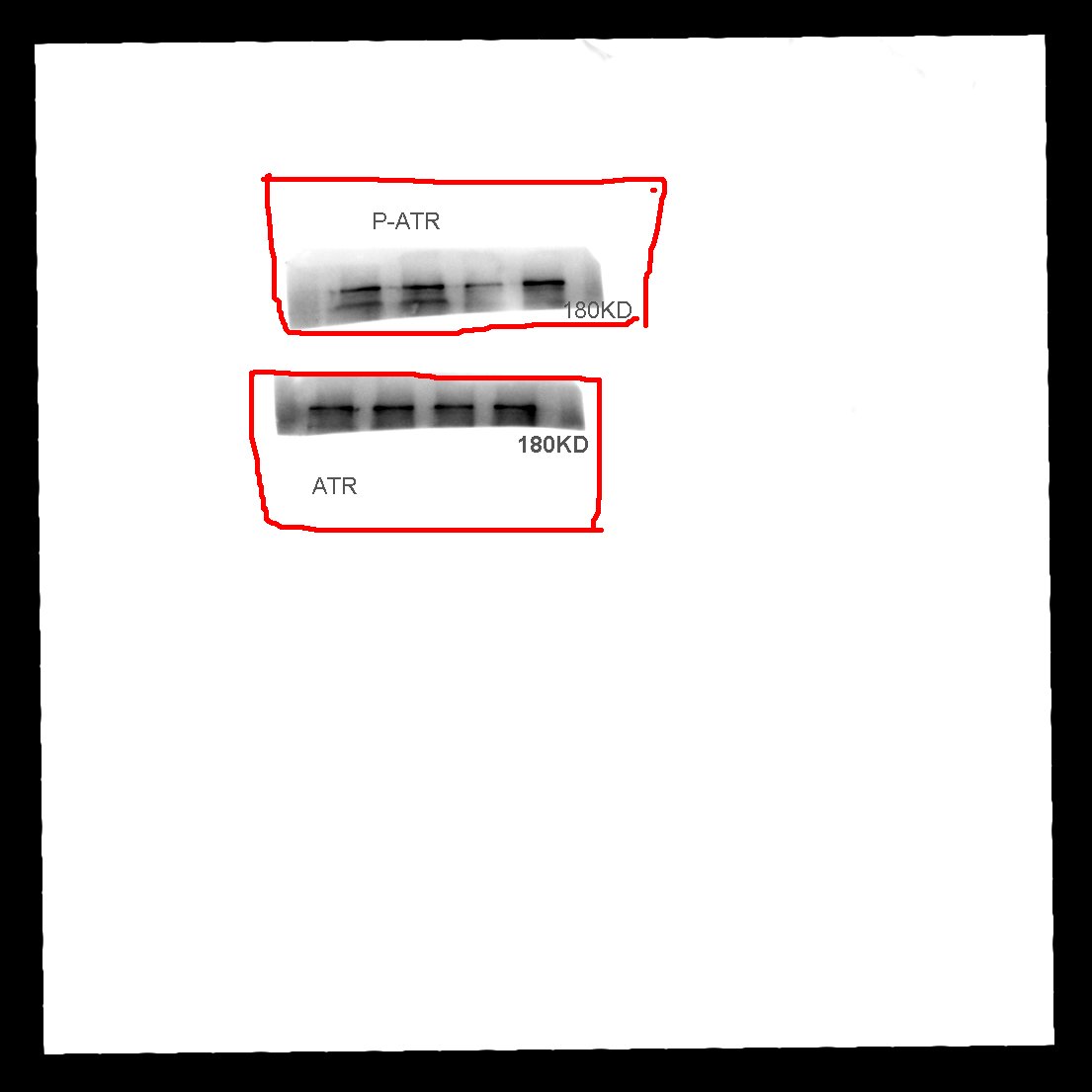

Supplement: Supplementary file 6 — Source Data Fig. 3 [file 44318_2023_3_MOESM6_ESM.zip › Figure3/3m-o/20210112 up p-ATR down ATR Loading sequence (right to left nc shDC1-1 (V WT MUT) ).jpg]

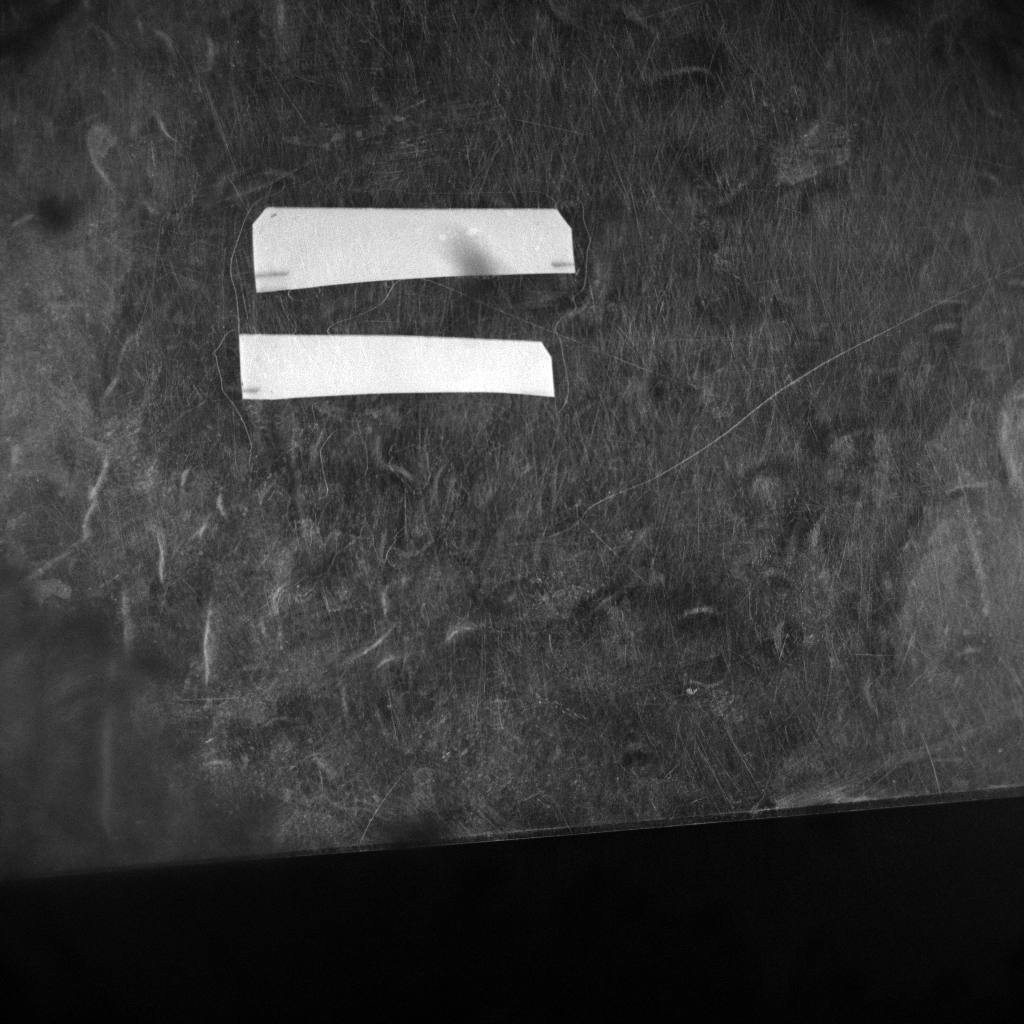

Supplement: Supplementary file 6 — Source Data Fig. 3 [file 44318_2023_3_MOESM6_ESM.zip › Figure3/3m-o/20210112 up p-ATR down ATR Loading sequence (right to left nc shDC1-1 (V WT MUT) ) white.tif]

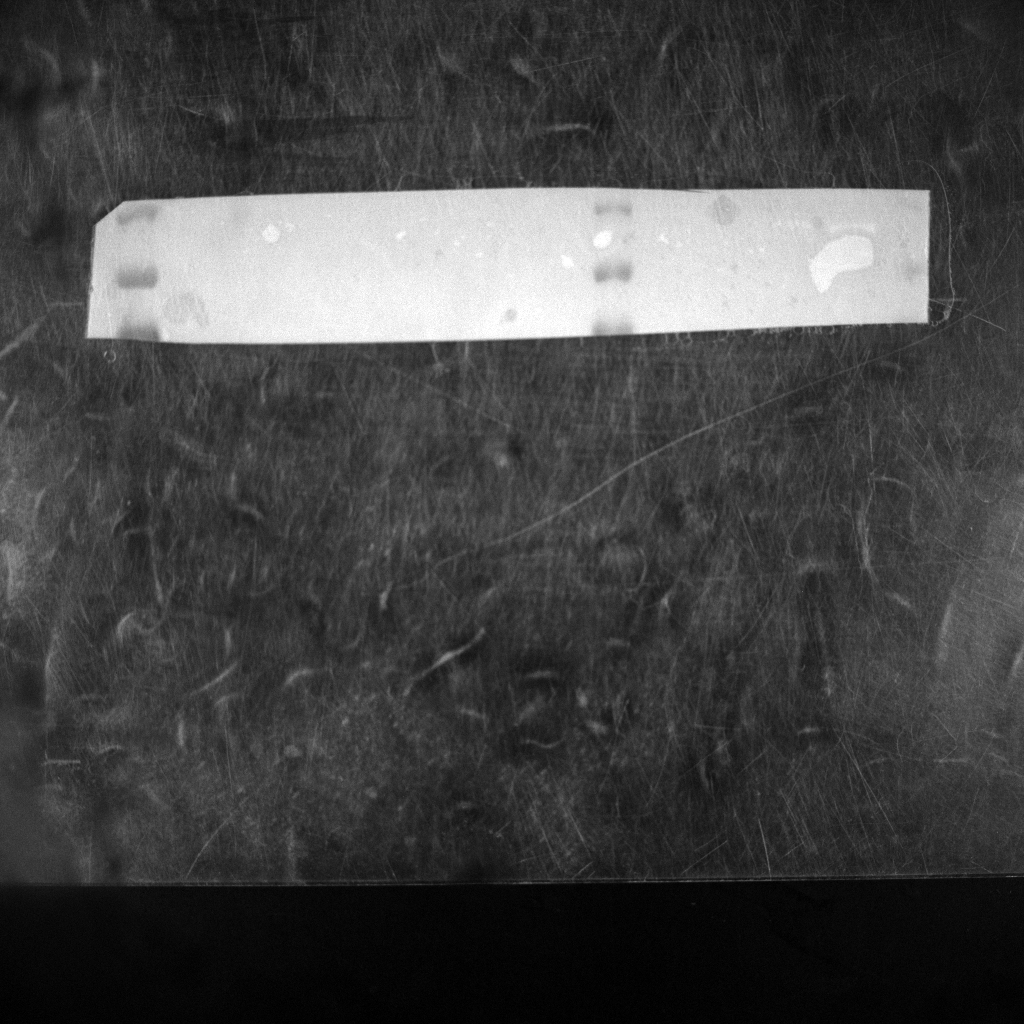

Supplement: Supplementary file 6 — Source Data Fig. 3 [file 44318_2023_3_MOESM6_ESM.zip › Figure3/3m-o/YTHDC1 control I-PpoI (nc siDC1-1 -2 si53BP1-1 -2 siRAD50 ) M NC shDC1-1 (v wt mut ) W.tif]

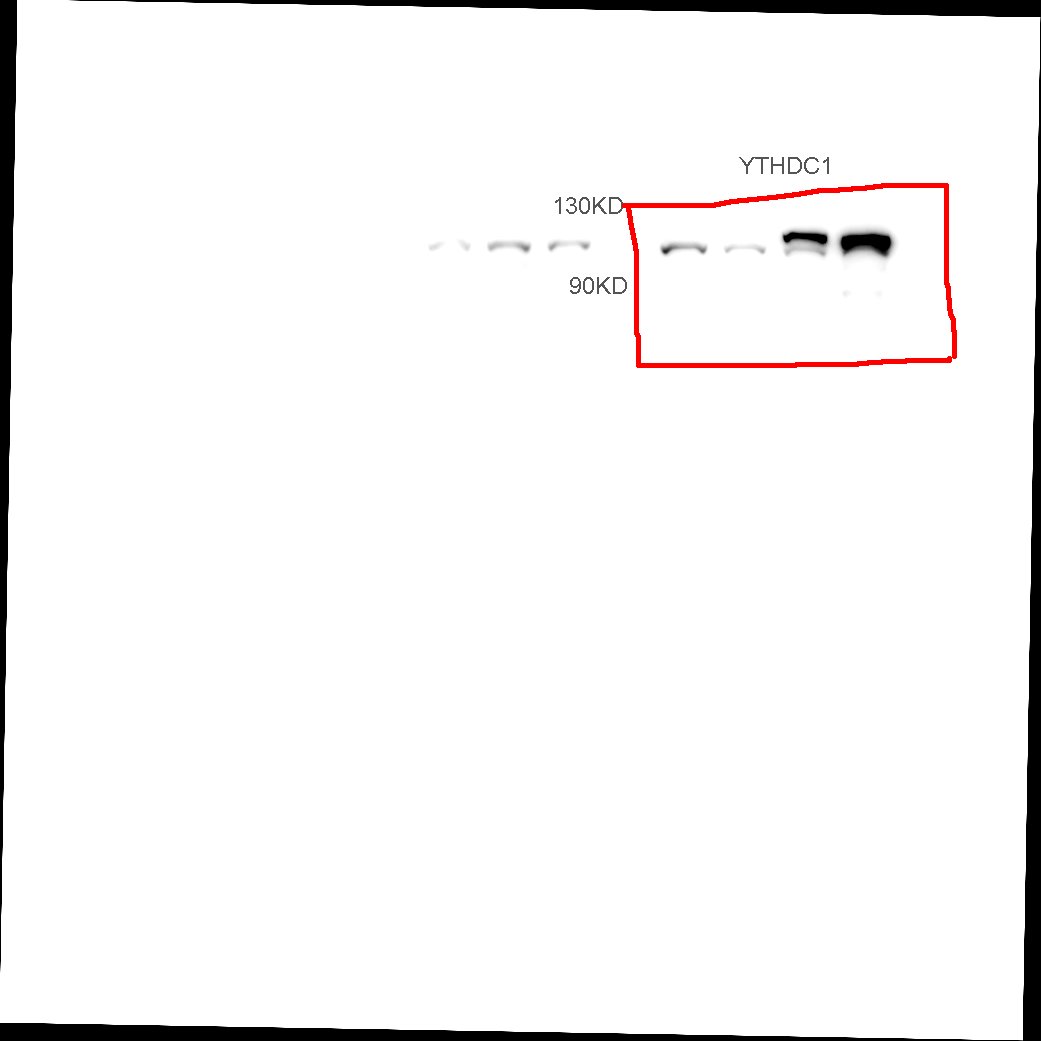

Supplement: Supplementary file 6 — Source Data Fig. 3 [file 44318_2023_3_MOESM6_ESM.zip › Figure3/3m-o/YTHDC1 control I-PpoI (nc siDC1-1 -2 si53BP1-1 -2 siRAD50 ) M NC shDC1-1 (v wt mut ).jpg]

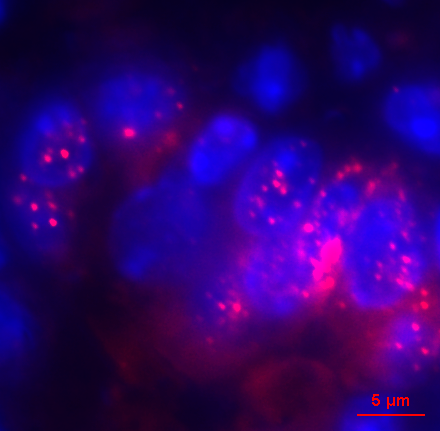

Supplement: Supplementary file 6 — Source Data Fig. 3 [file 44318_2023_3_MOESM6_ESM.zip › Figure3/3p-r/nc blm p-ATR IF/nc merge.tif]

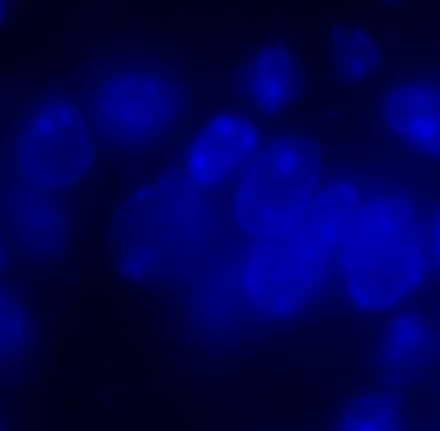

Supplement: Supplementary file 6 — Source Data Fig. 3 [file 44318_2023_3_MOESM6_ESM.zip › Figure3/3p-r/nc blm p-ATR IF/nc2 010_crop_RGB_DAPI.tif]

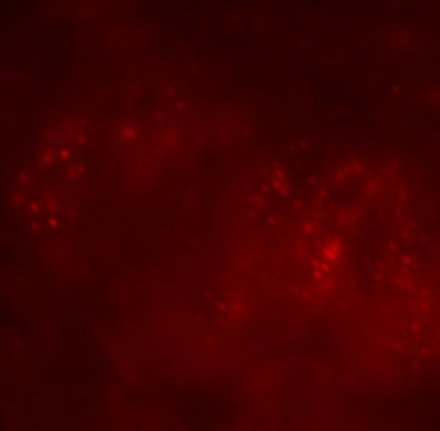

Supplement: Supplementary file 6 — Source Data Fig. 3 [file 44318_2023_3_MOESM6_ESM.zip › Figure3/3p-r/nc blm p-ATR IF/nc2 010_crop_RGB_TxRed.tif]

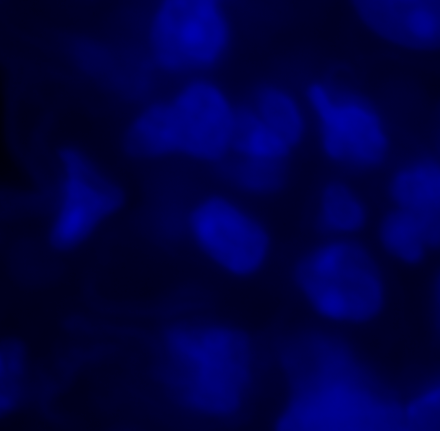

Supplement: Supplementary file 6 — Source Data Fig. 3 [file 44318_2023_3_MOESM6_ESM.zip › Figure3/3p-r/saline p-ATR IF/c2 007_crop_RGB_DAPI.tif]

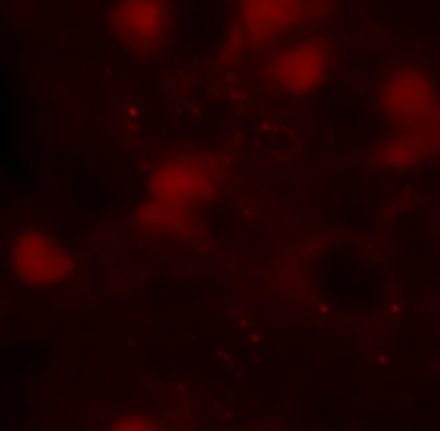

Supplement: Supplementary file 6 — Source Data Fig. 3 [file 44318_2023_3_MOESM6_ESM.zip › Figure3/3p-r/saline p-ATR IF/c2 007_crop_RGB_TxRed.tif]

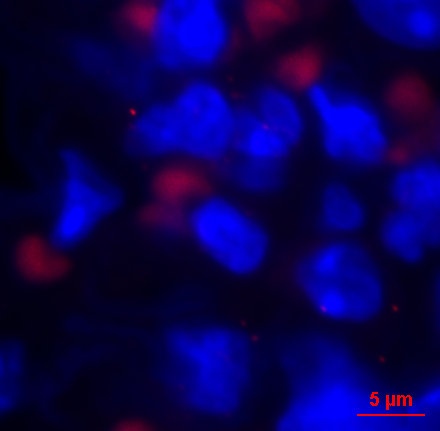

Supplement: Supplementary file 6 — Source Data Fig. 3 [file 44318_2023_3_MOESM6_ESM.zip › Figure3/3p-r/saline p-ATR IF/c2 merge.tif]

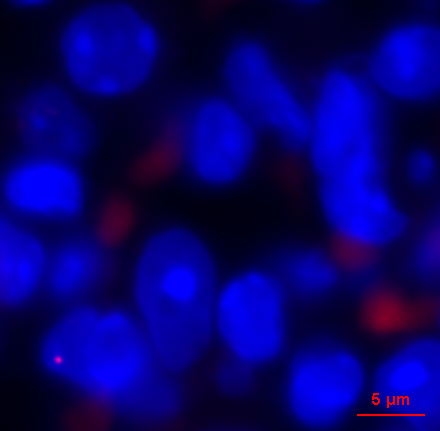

Supplement: Supplementary file 6 — Source Data Fig. 3 [file 44318_2023_3_MOESM6_ESM.zip › Figure3/3p-r/shYTHDC1 blm p-ATR IF/sh merge.tif]

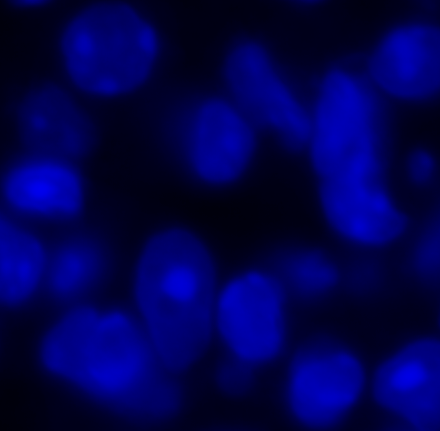

Supplement: Supplementary file 6 — Source Data Fig. 3 [file 44318_2023_3_MOESM6_ESM.zip › Figure3/3p-r/shYTHDC1 blm p-ATR IF/sh1 _crop_RGB_DAPI.tif]

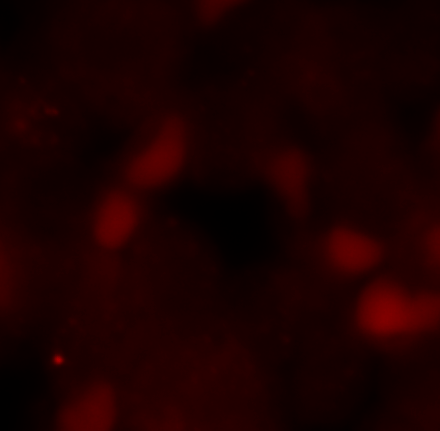

Supplement: Supplementary file 6 — Source Data Fig. 3 [file 44318_2023_3_MOESM6_ESM.zip › Figure3/3p-r/shYTHDC1 blm p-ATR IF/sh1 _crop_RGB_TxRed.tif]

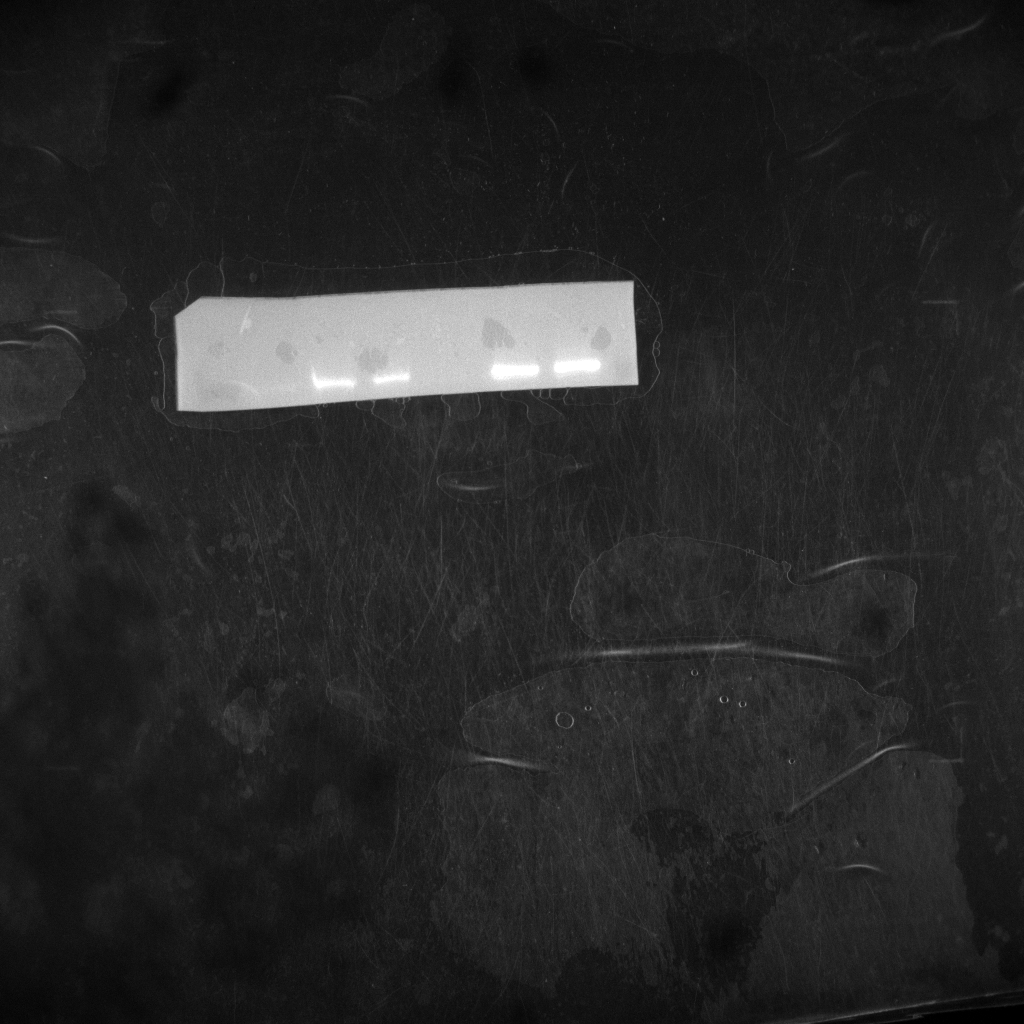

Supplement: Supplementary file 7 — Source Data Fig. 4 [file 44318_2023_3_MOESM7_ESM.zip › Figure4/4a/flag V TopBP1 (NC siDC1) (Input IP) w.tif]

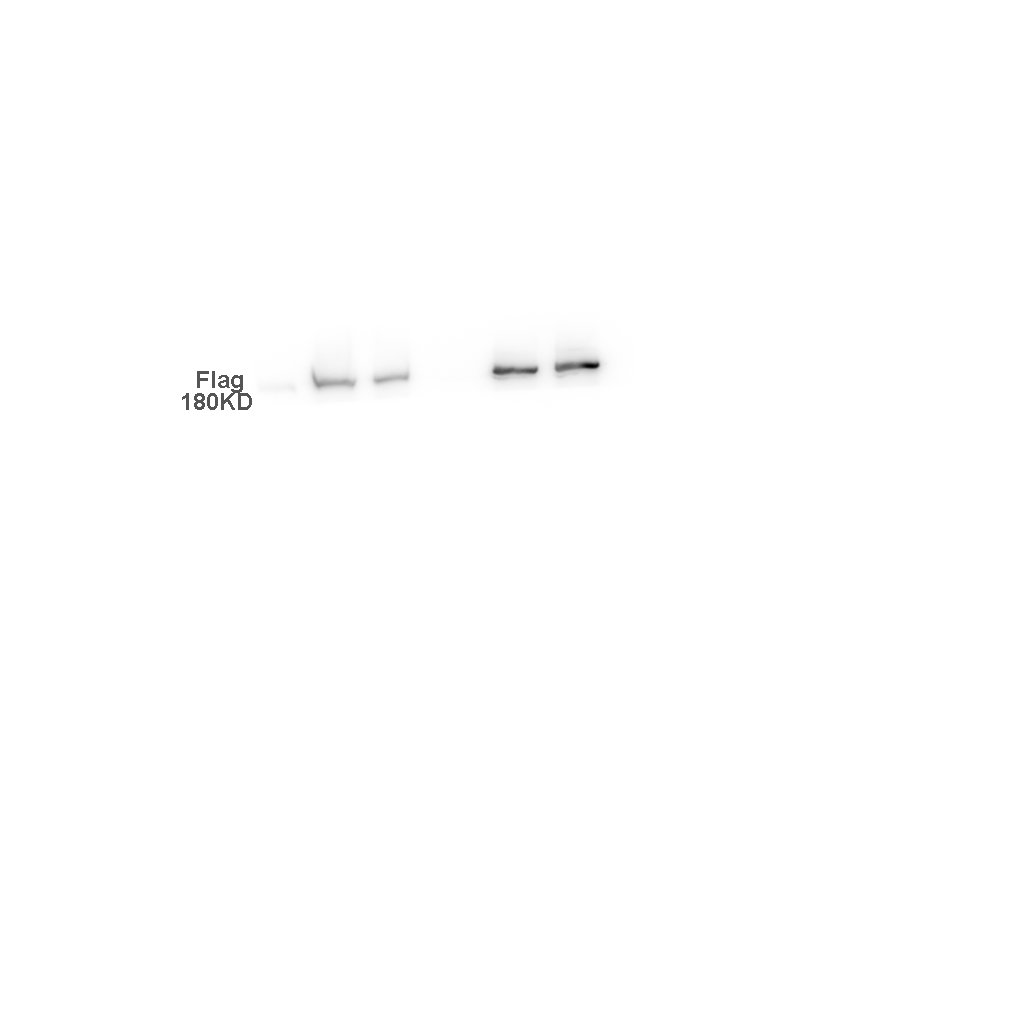

Supplement: Supplementary file 7 — Source Data Fig. 4 [file 44318_2023_3_MOESM7_ESM.zip › Figure4/4a/flag V TopBP1 (NC siDC1) (Input IP) .jpg]

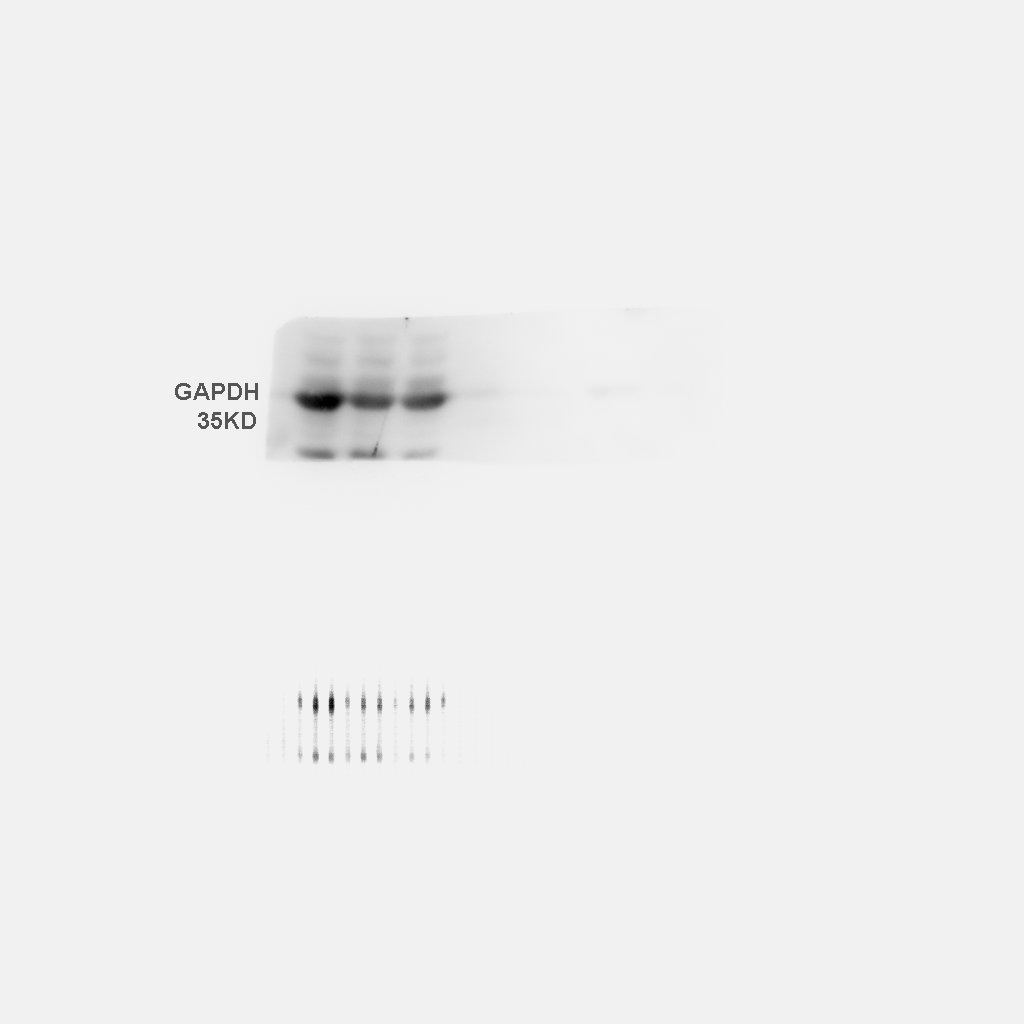

Supplement: Supplementary file 7 — Source Data Fig. 4 [file 44318_2023_3_MOESM7_ESM.zip › Figure4/4a/gapdh V TopBP1 (NC siDC1) (Input IP) -1 .jpg]

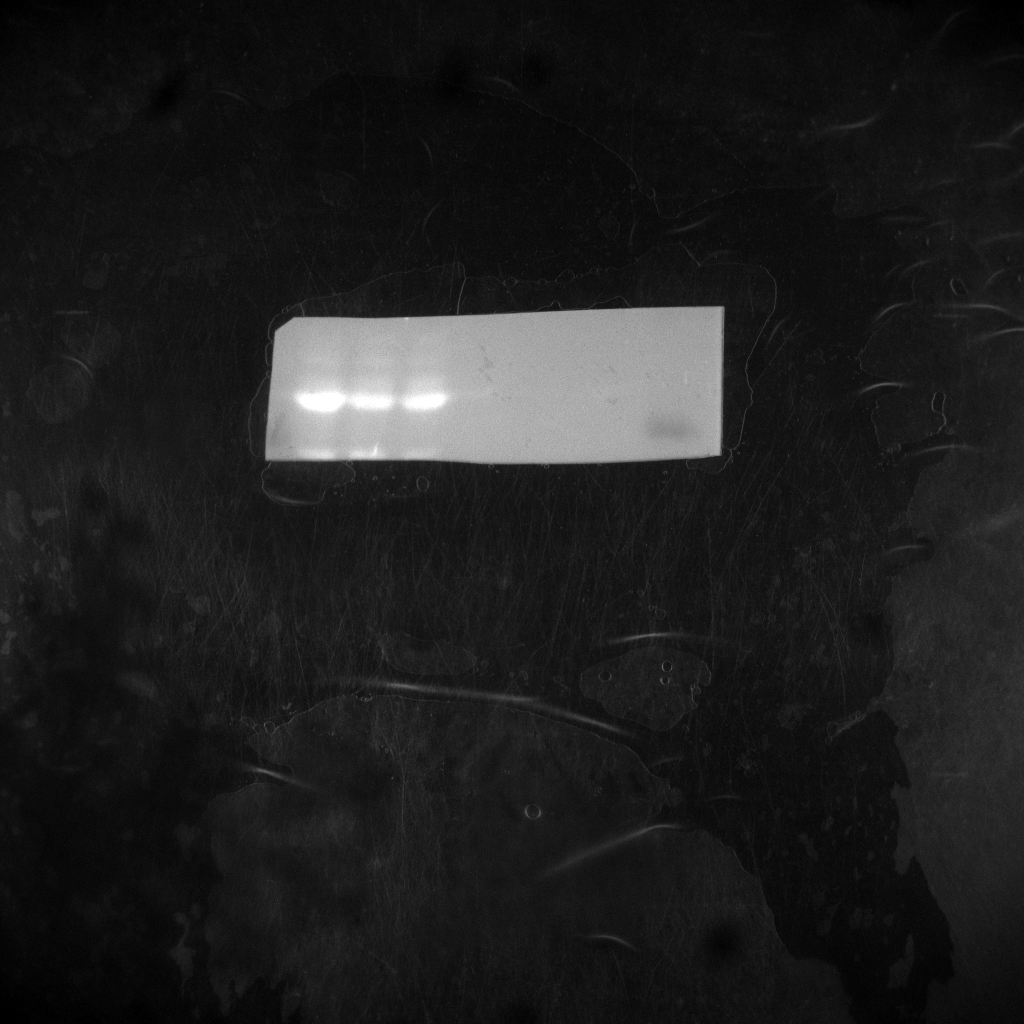

Supplement: Supplementary file 7 — Source Data Fig. 4 [file 44318_2023_3_MOESM7_ESM.zip › Figure4/4a/gapdh V TopBP1 (NC siDC1) (Input IP) -1 w .tif]

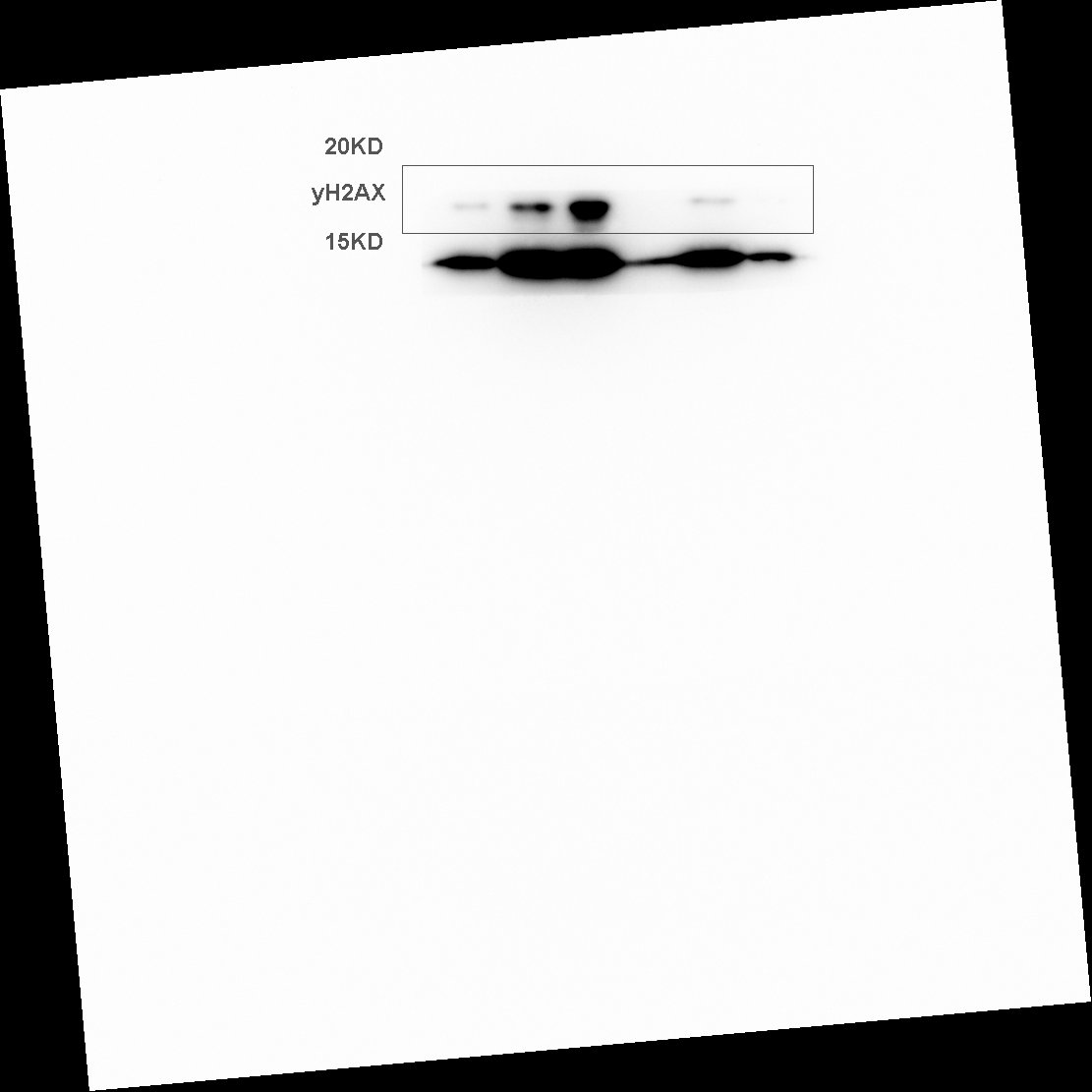

Supplement: Supplementary file 7 — Source Data Fig. 4 [file 44318_2023_3_MOESM7_ESM.zip › Figure4/4a/yh2ax V NC siDC1(TOPBP1) input IP .jpg]

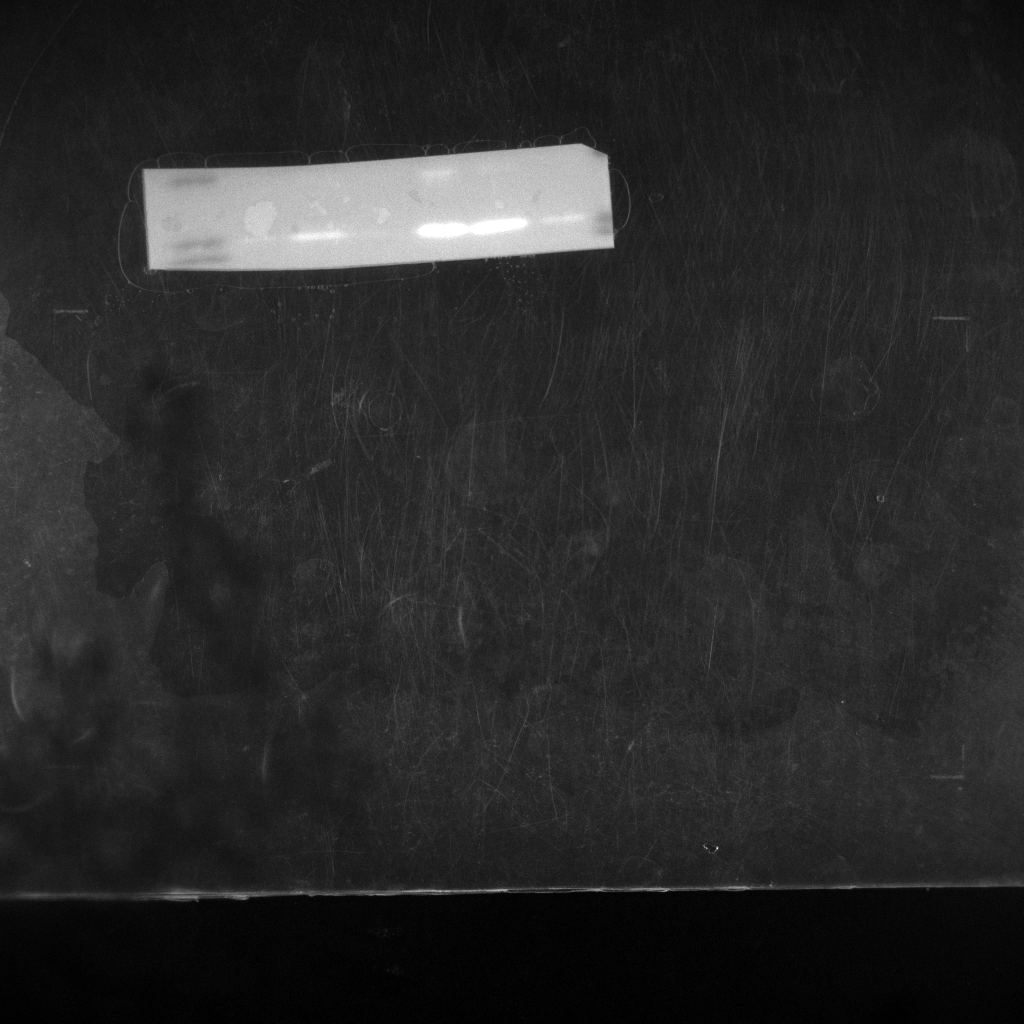

Supplement: Supplementary file 7 — Source Data Fig. 4 [file 44318_2023_3_MOESM7_ESM.zip › Figure4/4a/yh2ax V NC siDC1(TOPBP1) input IP W.tif]

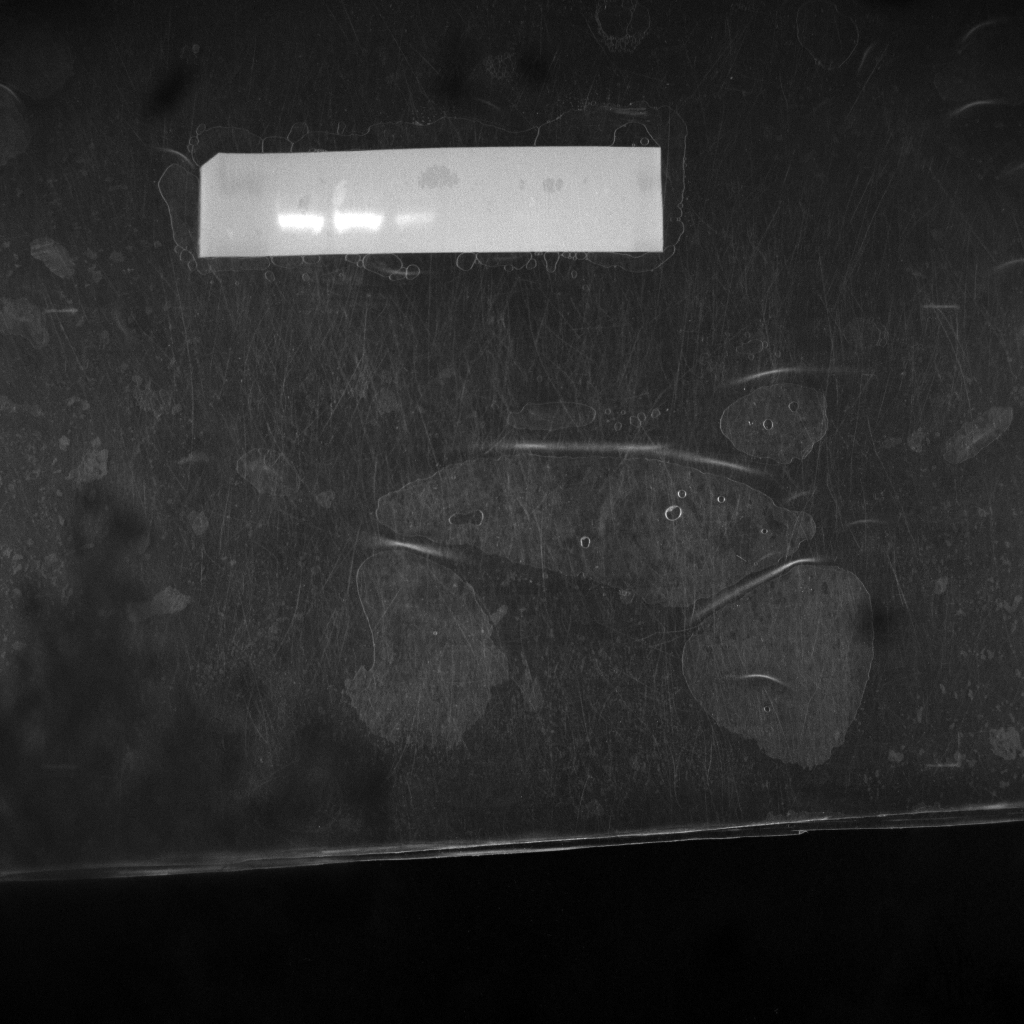

Supplement: Supplementary file 7 — Source Data Fig. 4 [file 44318_2023_3_MOESM7_ESM.zip › Figure4/4a/YTHDC1 V TopBP1 (NC siDC1) (Input IP) w.tif]

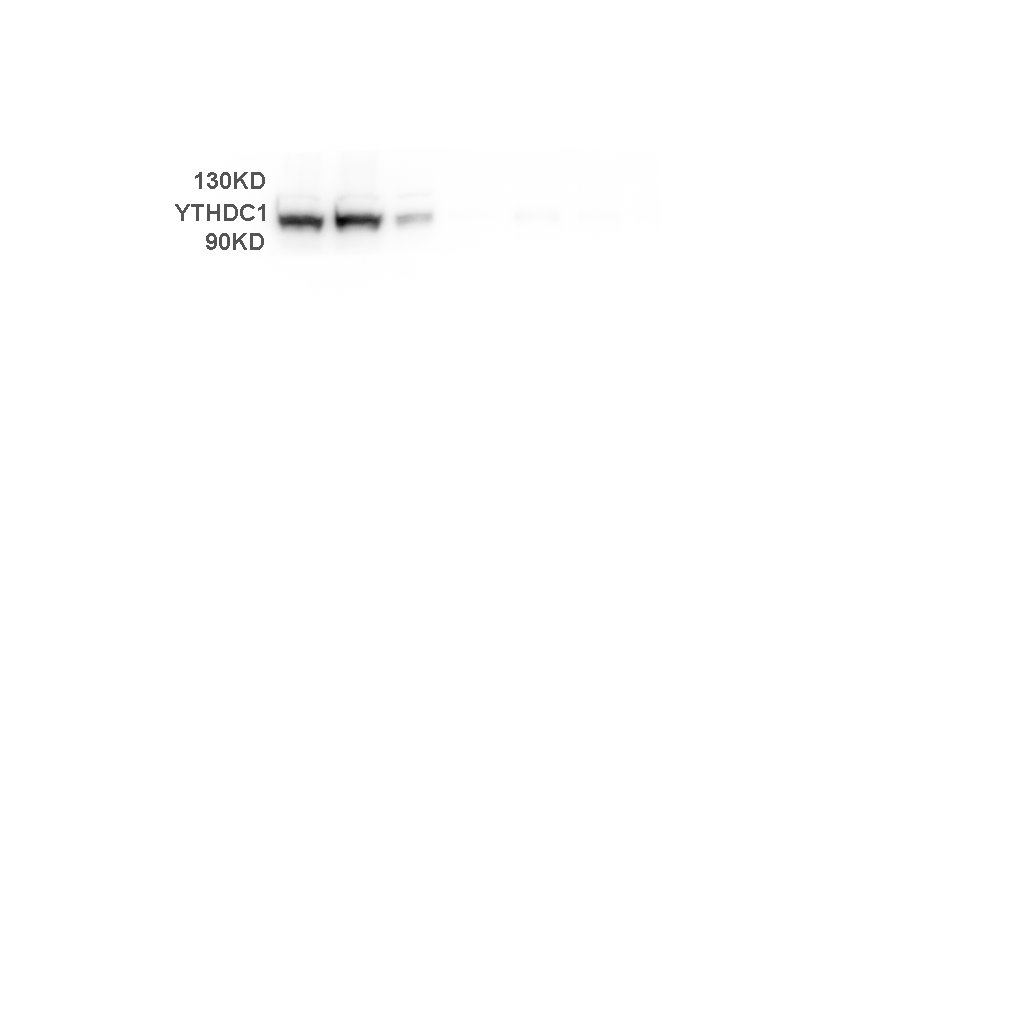

Supplement: Supplementary file 7 — Source Data Fig. 4 [file 44318_2023_3_MOESM7_ESM.zip › Figure4/4a/YTHDC1 V TopBP1 (NC siDC1) (Input IP).jpg]

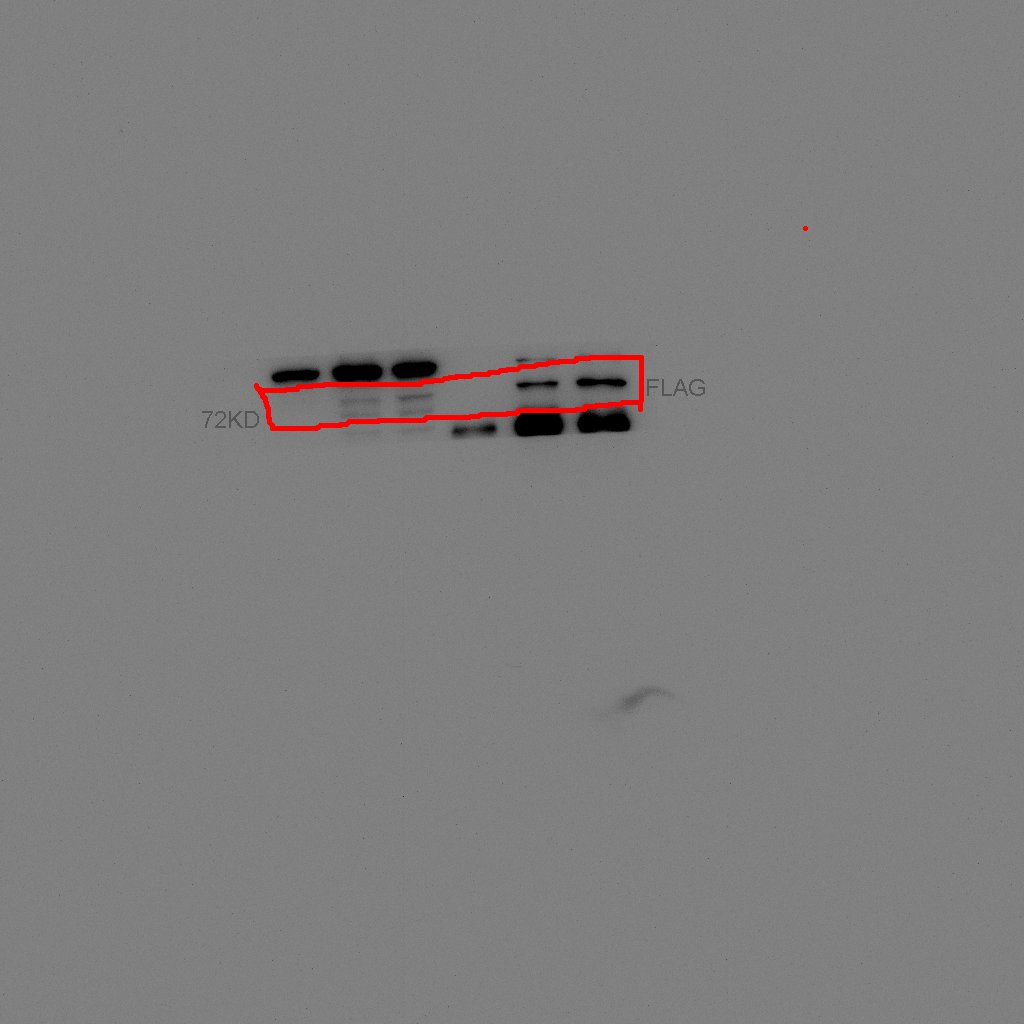

Supplement: Supplementary file 7 — Source Data Fig. 4 [file 44318_2023_3_MOESM7_ESM.zip › Figure4/4b/FLAG ATRIP-IP V NC siDC1-1 (INPUT IP) .jpg]

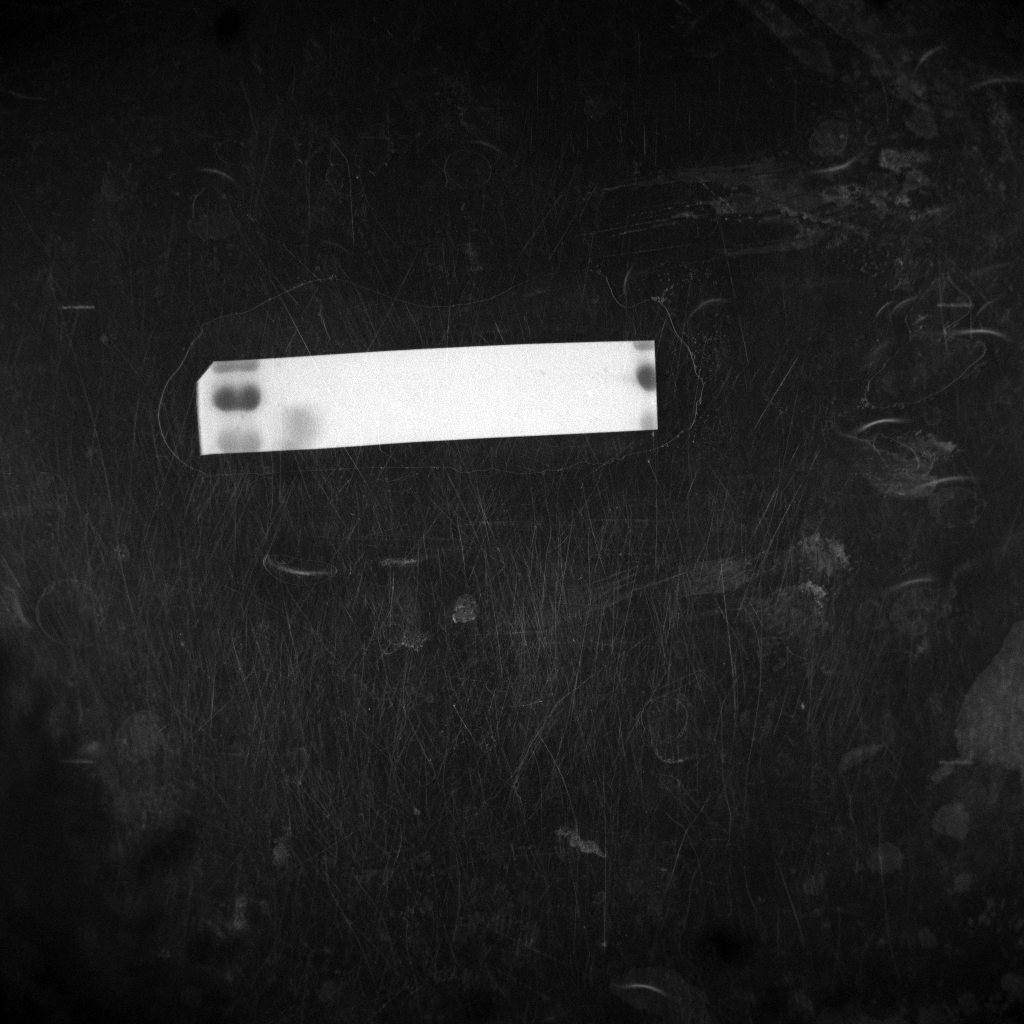

Supplement: Supplementary file 7 — Source Data Fig. 4 [file 44318_2023_3_MOESM7_ESM.zip › Figure4/4b/FLAG ATRIP-IP V NC siDC1-1 (INPUT IP) W .tif]

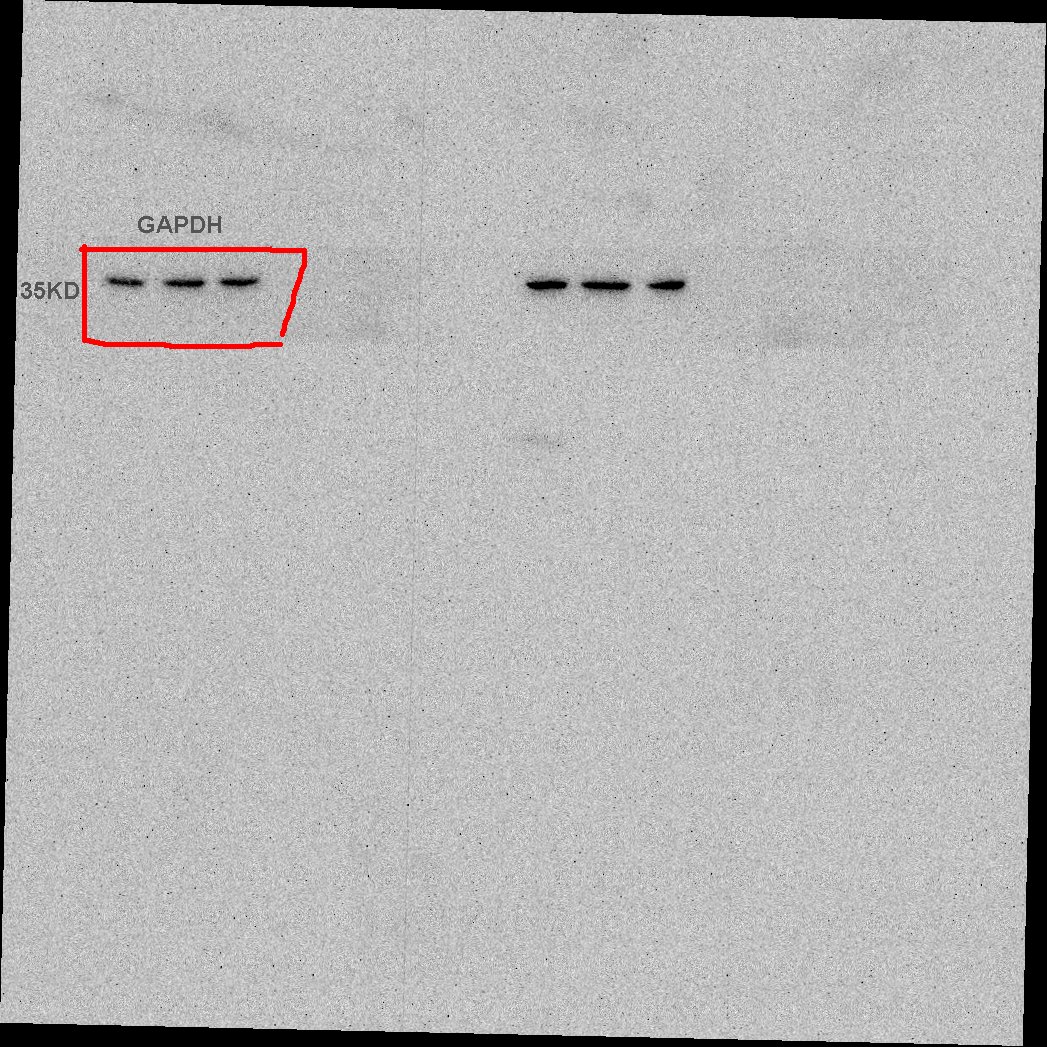

Supplement: Supplementary file 7 — Source Data Fig. 4 [file 44318_2023_3_MOESM7_ESM.zip › Figure4/4b/gapdh V NC siDC1-1 (INPUT ATRIP-IP) M V NC siDC1-1 (INPUT RAD17-IP) .jpg]

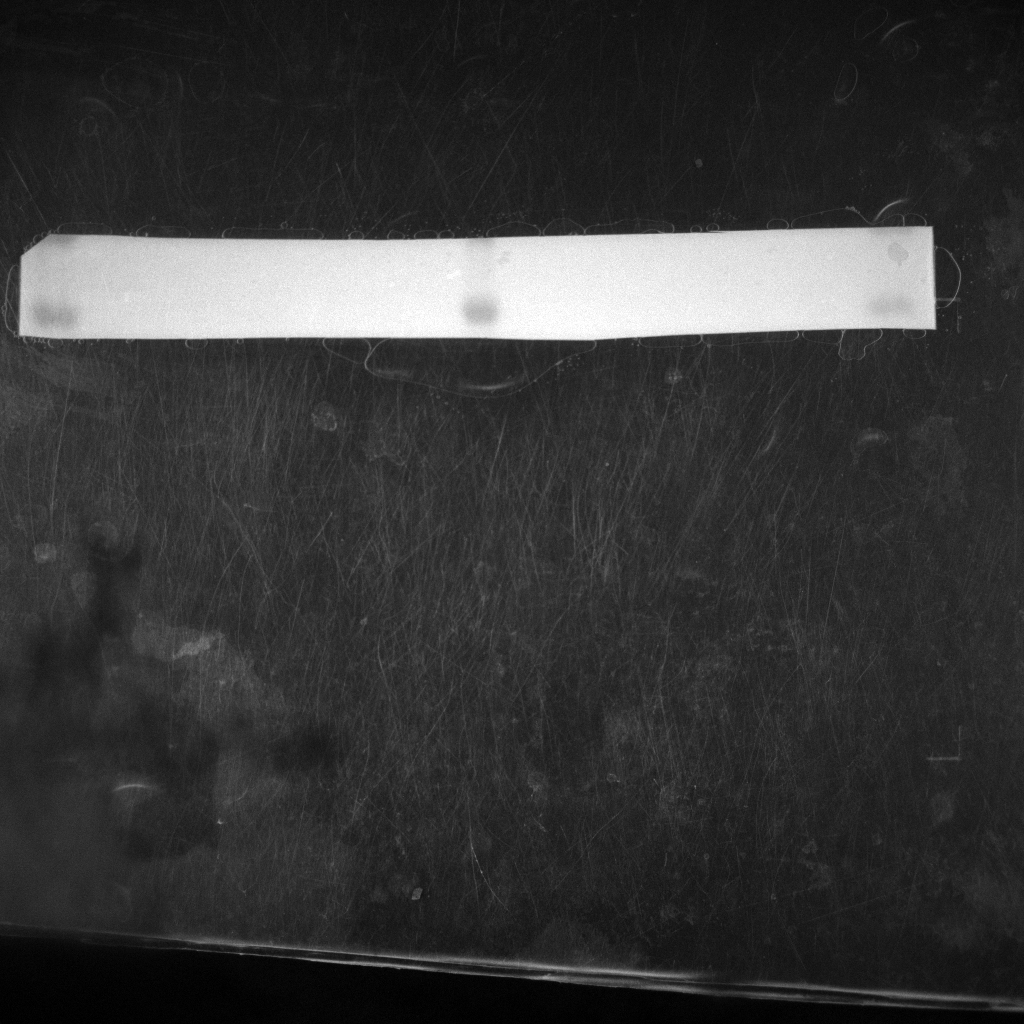

Supplement: Supplementary file 7 — Source Data Fig. 4 [file 44318_2023_3_MOESM7_ESM.zip › Figure4/4b/gapdh V NC siDC1-1 (INPUT ATRIP-IP) M V NC siDC1-1 (INPUT RAD17-IP) w .tif]

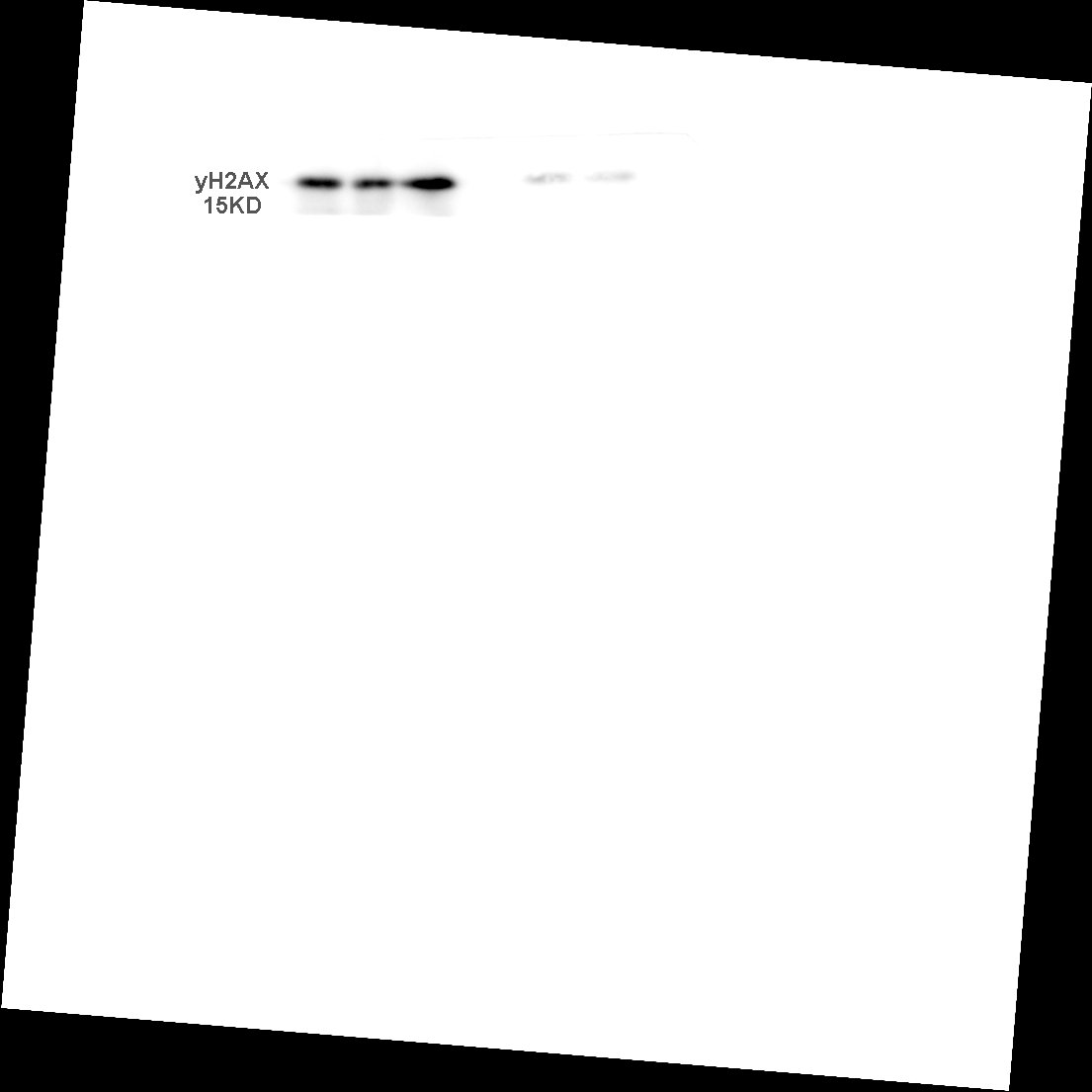

Supplement: Supplementary file 7 — Source Data Fig. 4 [file 44318_2023_3_MOESM7_ESM.zip › Figure4/4b/yh2ax ATRIP-IP V NC siDC1-1 (INPUT IP) .jpg]

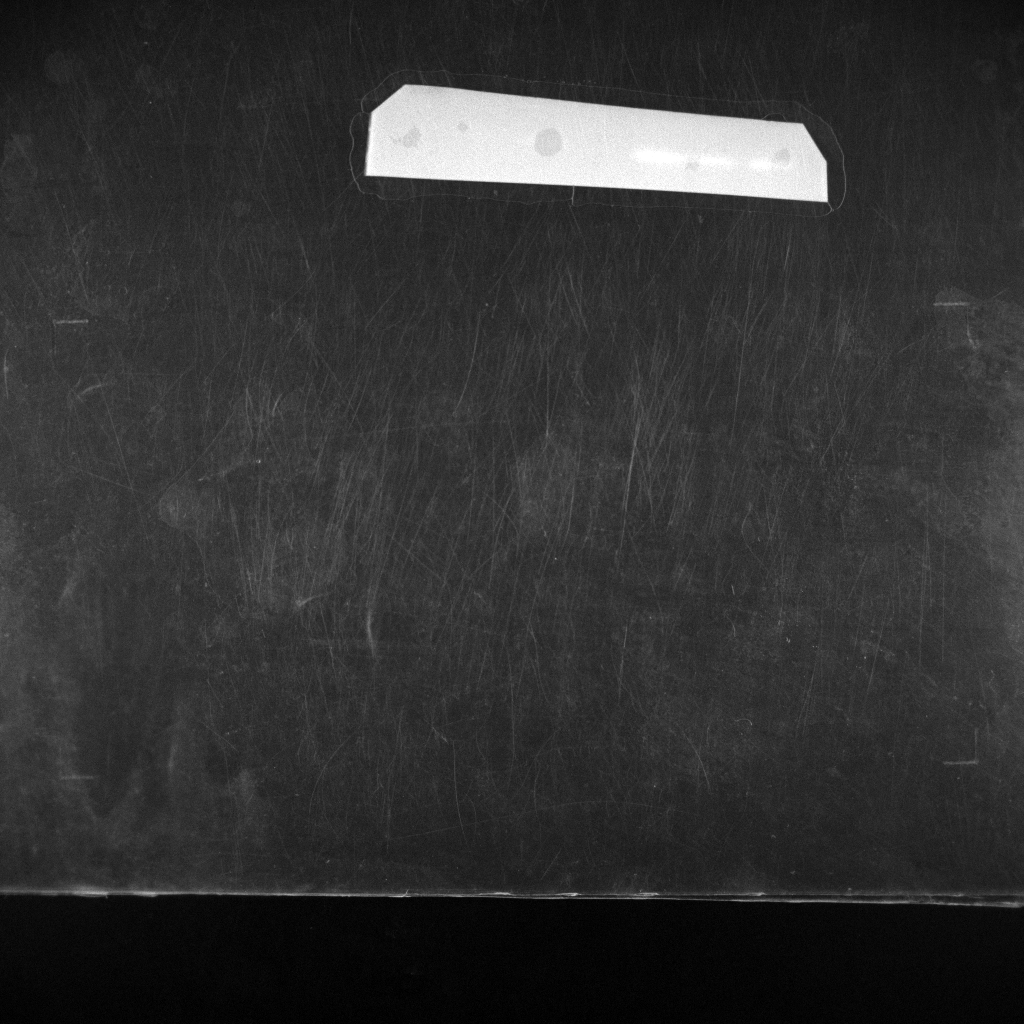

Supplement: Supplementary file 7 — Source Data Fig. 4 [file 44318_2023_3_MOESM7_ESM.zip › Figure4/4b/yh2ax ATRIP-IP V NC siDC1-1 (INPUT IP) w .tif]

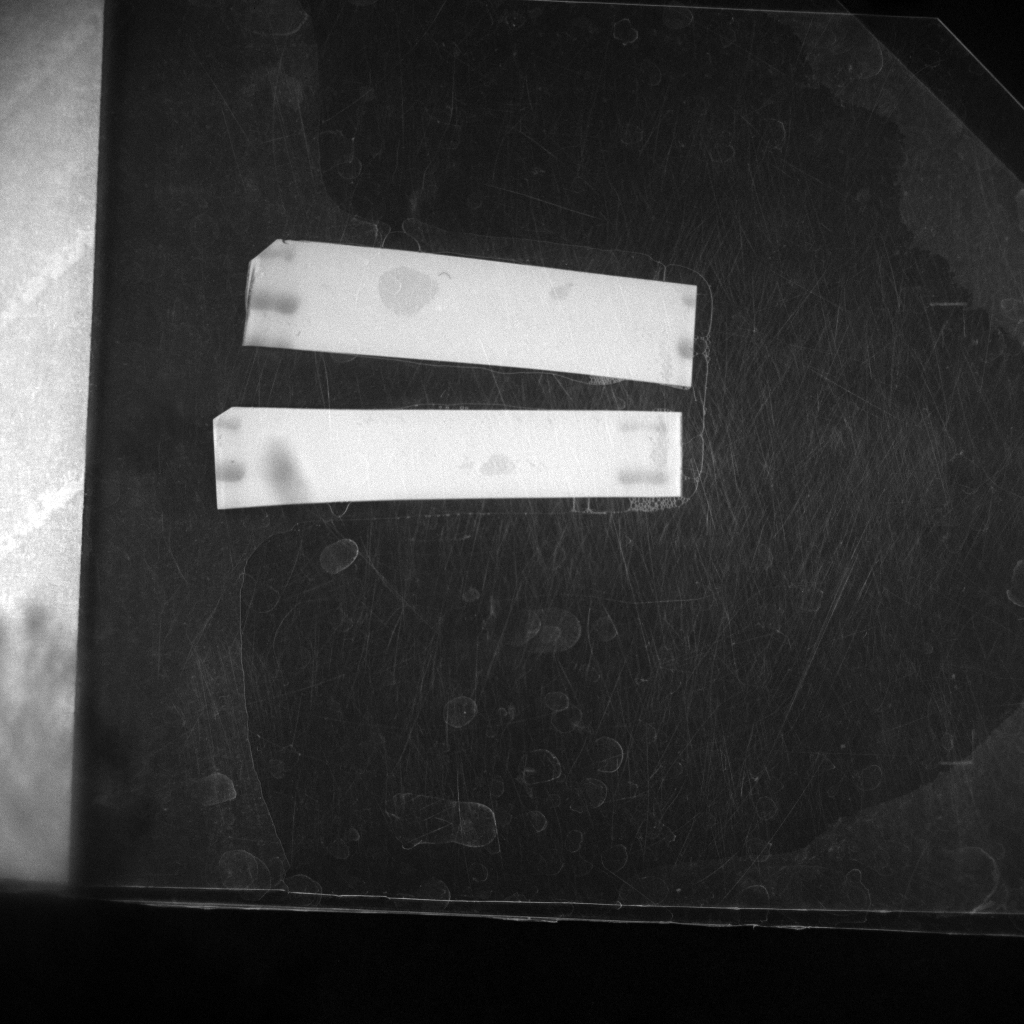

Supplement: Supplementary file 7 — Source Data Fig. 4 [file 44318_2023_3_MOESM7_ESM.zip › Figure4/4b/YTHDC1 up V NC siDC1-1 (INPUT ATRIP-IP) M down V NC siDC1-1 (INPUT RAD17-IP) w.tif]

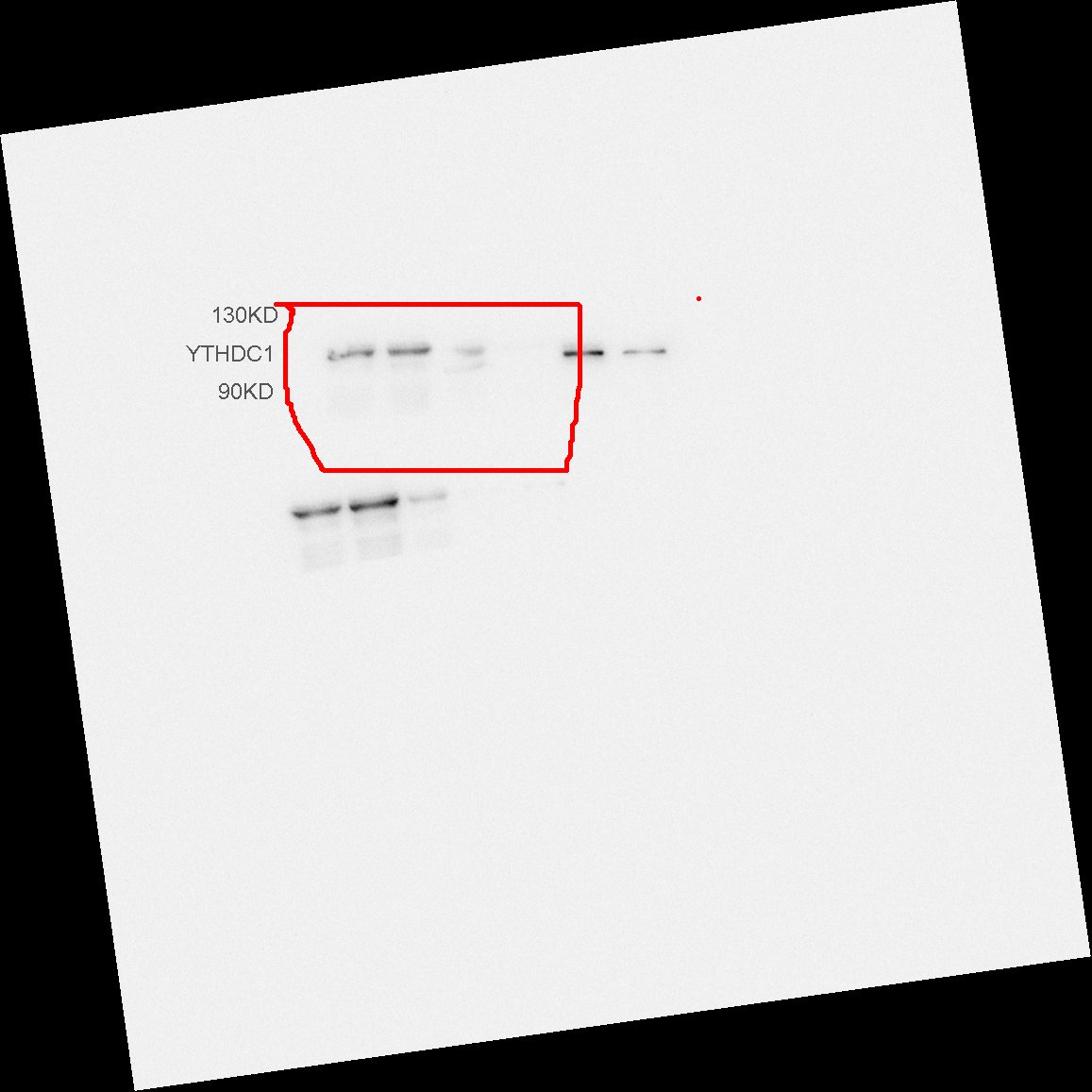

Supplement: Supplementary file 7 — Source Data Fig. 4 [file 44318_2023_3_MOESM7_ESM.zip › Figure4/4b/YTHDC1 up V NC siDC1-1 (INPUT ATRIP-IP) M down V NC siDC1-1 (INPUT RAD17-IP).jpg]

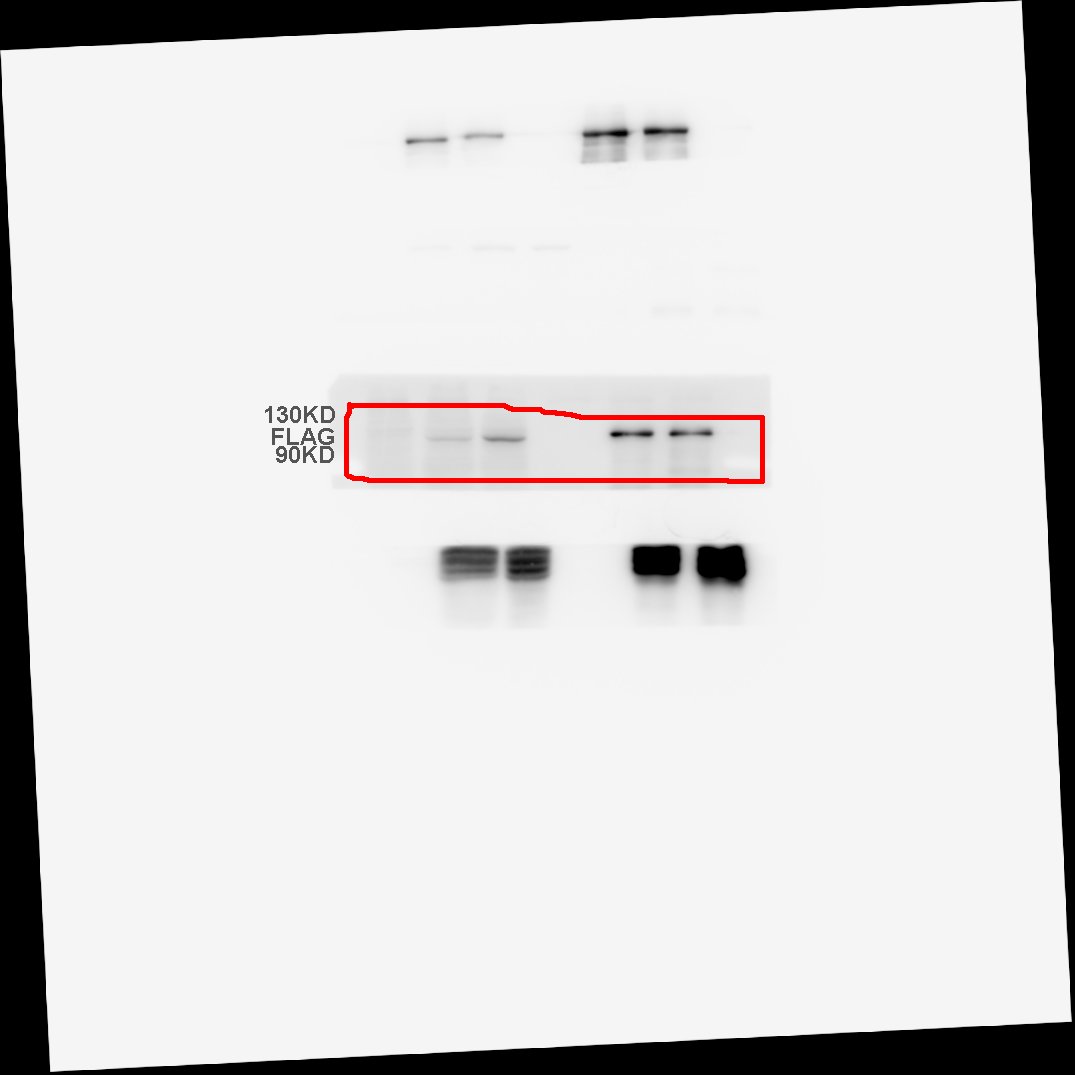

Supplement: Supplementary file 7 — Source Data Fig. 4 [file 44318_2023_3_MOESM7_ESM.zip › Figure4/4c/FLAG up to down Topbp1-IP ATRIP-IP RAD17-IP RAD9-IP V NC siDC1-1 (INPUT IP) .jpg]

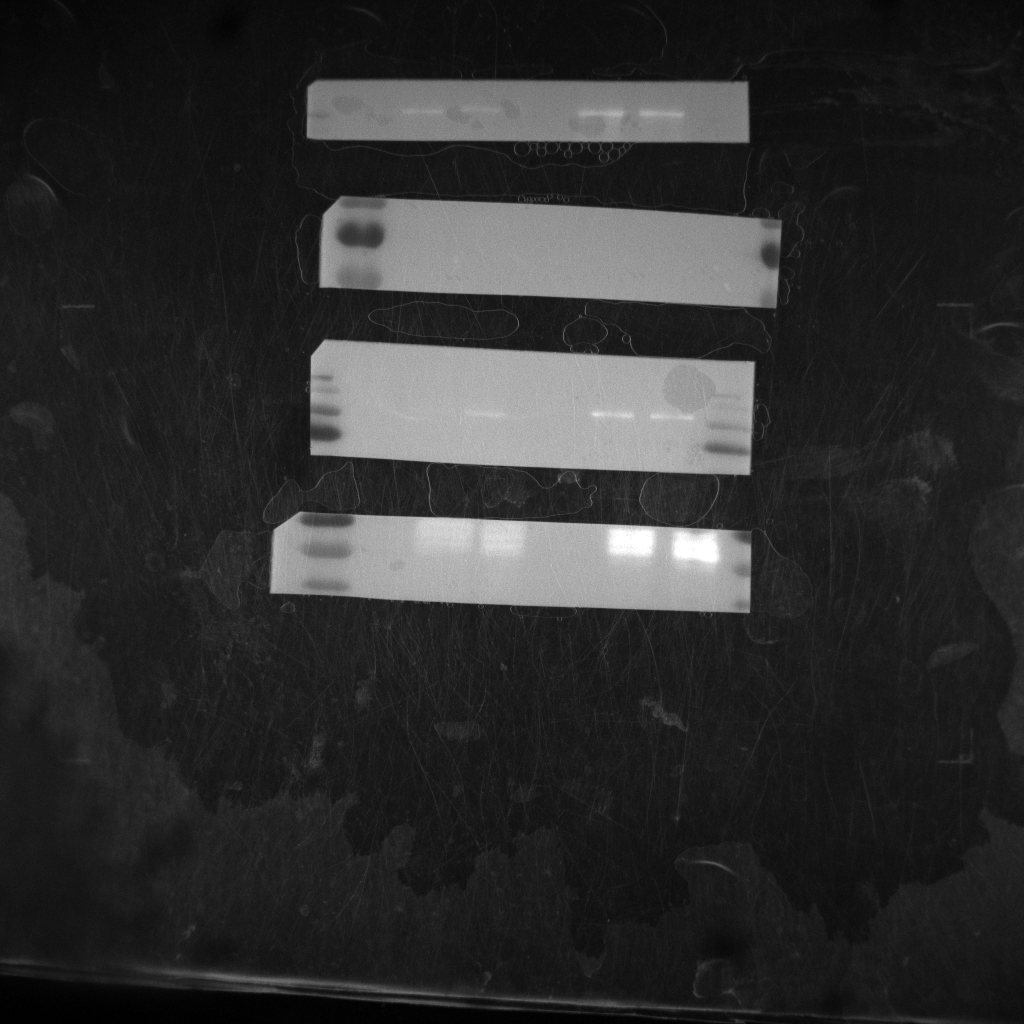

Supplement: Supplementary file 7 — Source Data Fig. 4 [file 44318_2023_3_MOESM7_ESM.zip › Figure4/4c/FLAG up to down Topbp1-IP ATRIP-IP RAD17-IP RAD9-IP V NC siDC1-1 (INPUT IP) w .tif]

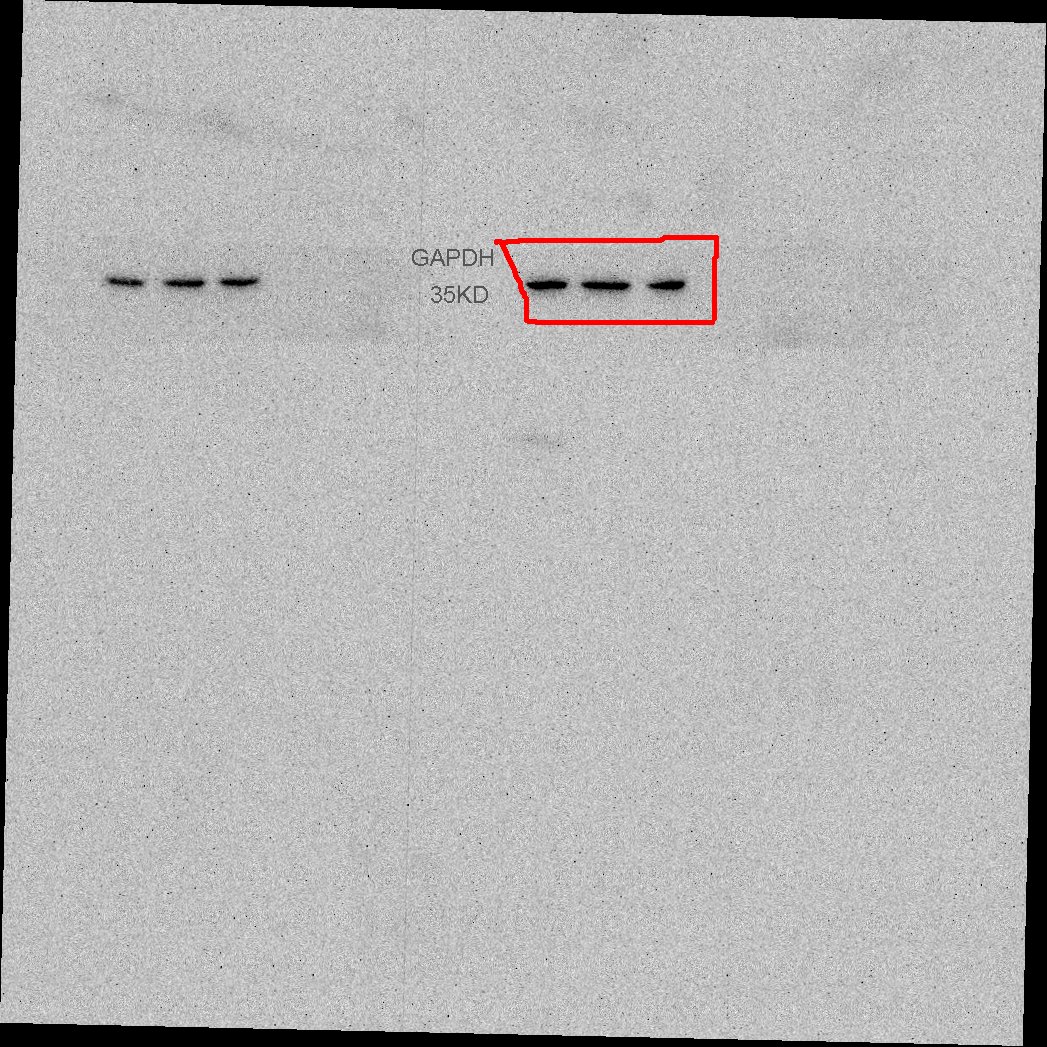

Supplement: Supplementary file 7 — Source Data Fig. 4 [file 44318_2023_3_MOESM7_ESM.zip › Figure4/4c/gapdh V NC siDC1-1 (INPUT ATRIP-IP) M V NC siDC1-1 (INPUT RAD17-IP) .jpg]

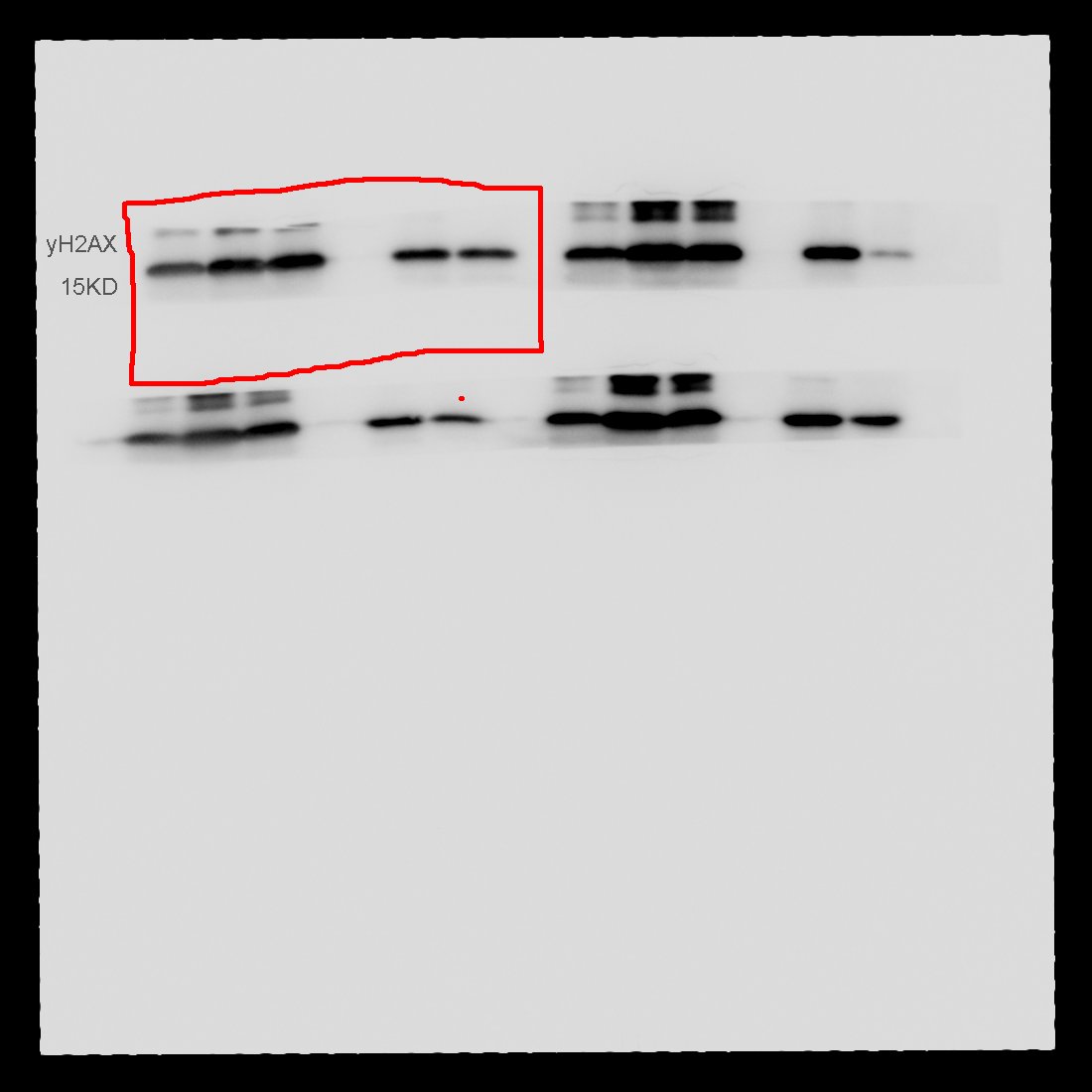

Supplement: Supplementary file 7 — Source Data Fig. 4 [file 44318_2023_3_MOESM7_ESM.zip › Figure4/4c/yh2ax up and left RAD17-IP V NC siDC1-1 (INPUT IP) .jpg]

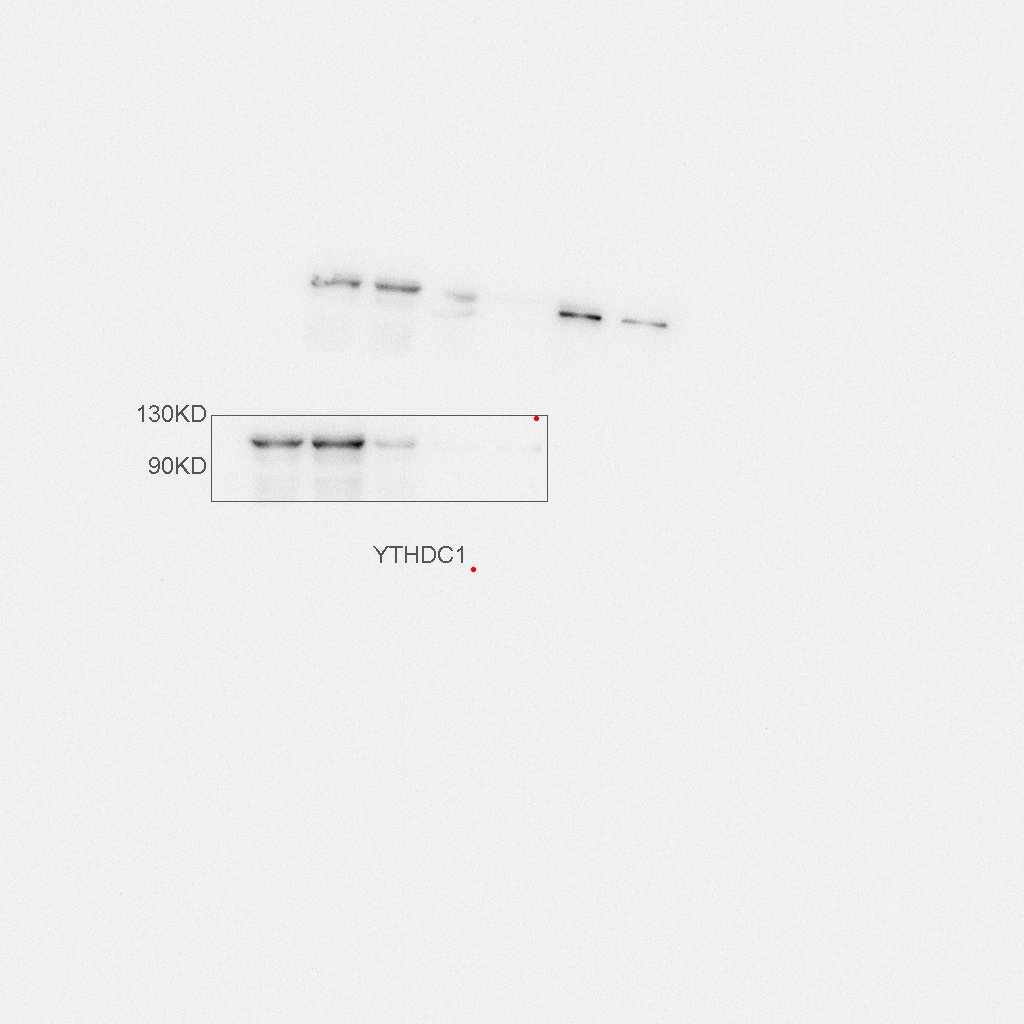

Supplement: Supplementary file 7 — Source Data Fig. 4 [file 44318_2023_3_MOESM7_ESM.zip › Figure4/4c/YTHDC1 up V NC siDC1-1 (INPUT ATRIP-IP) M down V NC siDC1-1 (INPUT RAD17-IP).jpg]
