# Supplementary figures and images for: YTHDC1 delays cellular senescence and pulmonary fibrosis by activating ATR in an m6A-independent manner (part 2 of 4)
Source: EMBO J. 2023 Dec 15;43(1):4. doi: 10.1038/s44318-023-00003-2 (PMC10883269; doi:10.1038/s44318-023-00003-2)

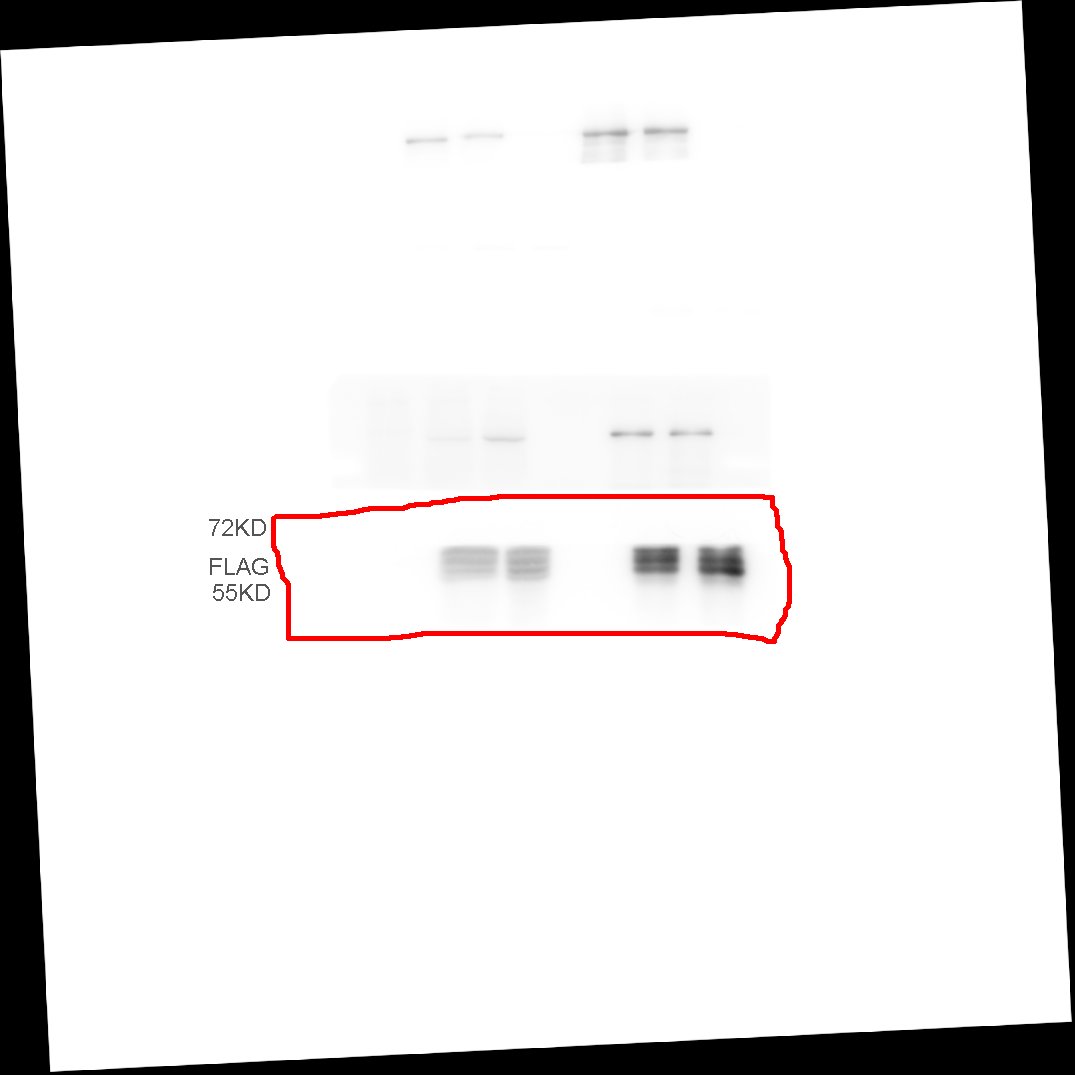

Supplement: Supplementary file 7 — Source Data Fig. 4 [file 44318_2023_3_MOESM7_ESM.zip › Figure4/4d/flag down RAD9-IP V NC siDC1-1 (INPUT IP) .jpg]

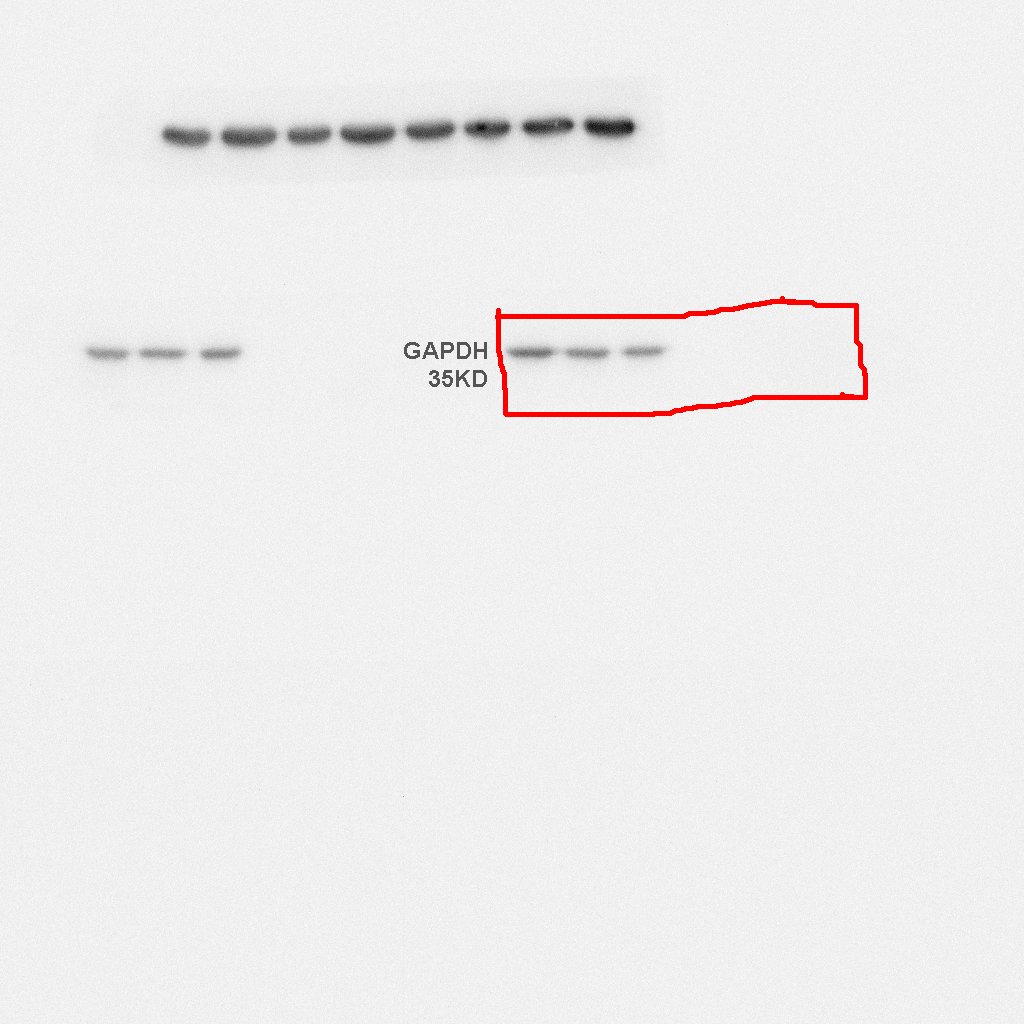

Supplement: Supplementary file 7 — Source Data Fig. 4 [file 44318_2023_3_MOESM7_ESM.zip › Figure4/4d/GAPDH down and right rad9-IP V NC siDC1-1 (INPUT IP) .jpg]

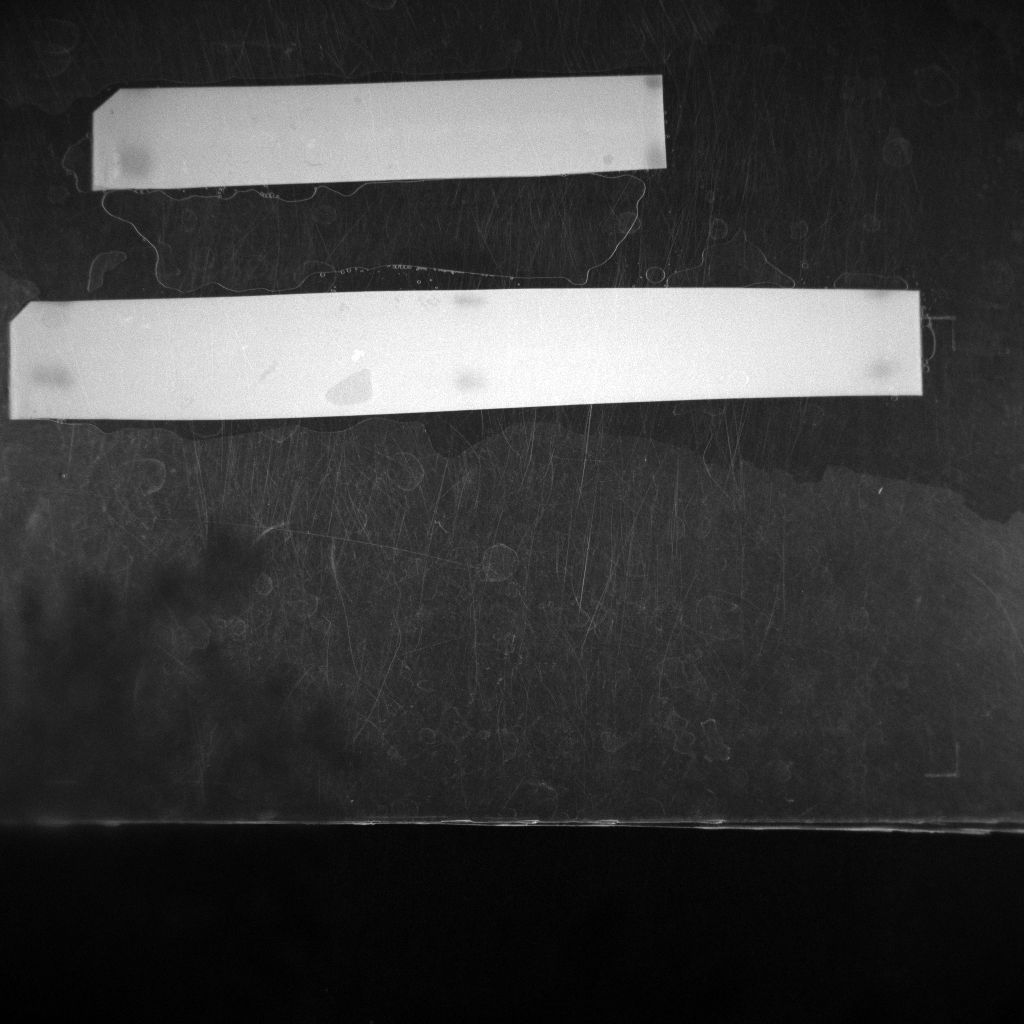

Supplement: Supplementary file 7 — Source Data Fig. 4 [file 44318_2023_3_MOESM7_ESM.zip › Figure4/4d/GAPDH down and right rad9-IP V NC siDC1-1 (INPUT IP) w .tif]

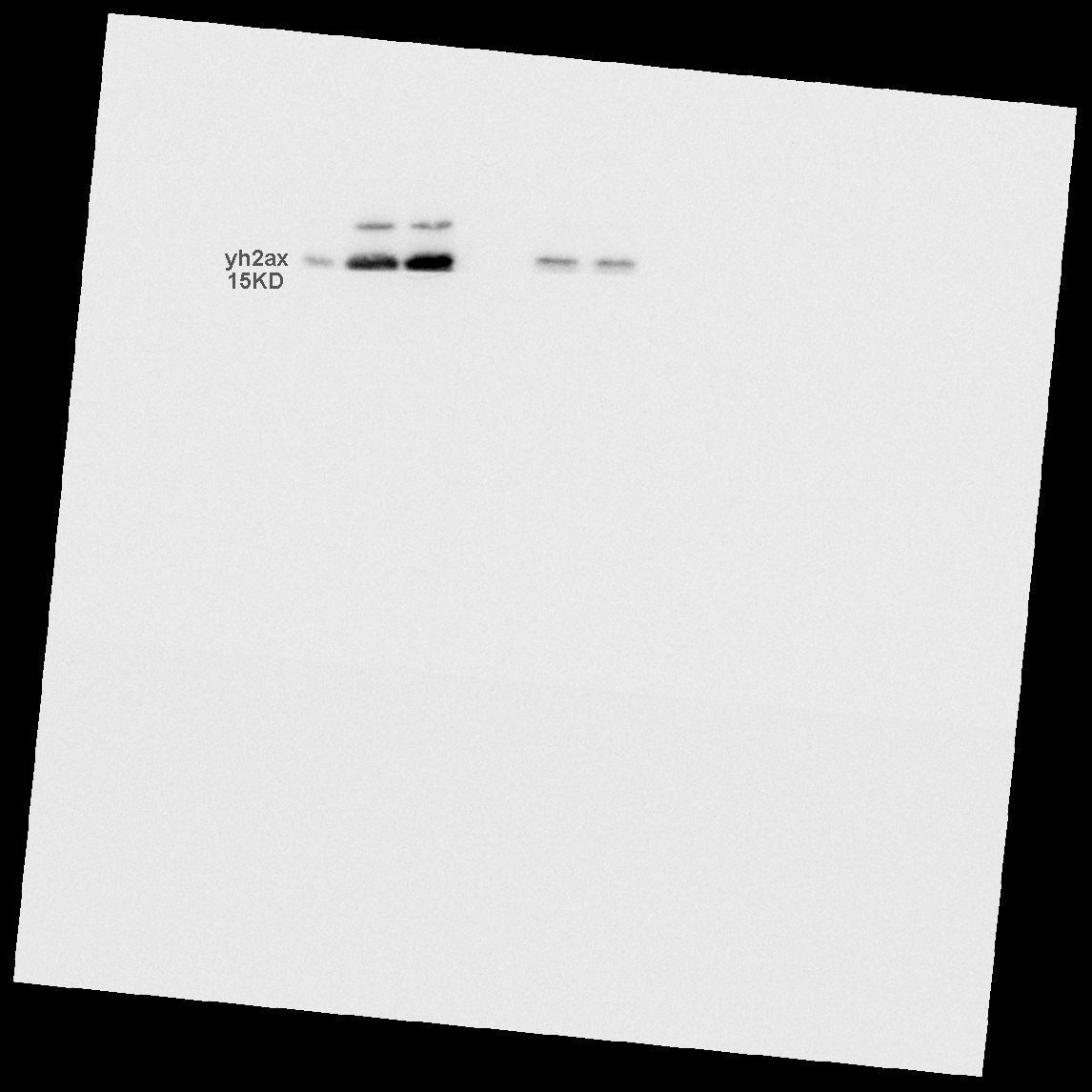

Supplement: Supplementary file 7 — Source Data Fig. 4 [file 44318_2023_3_MOESM7_ESM.zip › Figure4/4d/yh2ax RAD9A -IP V NC siDC1-1 (INPUT IP) .jpg]

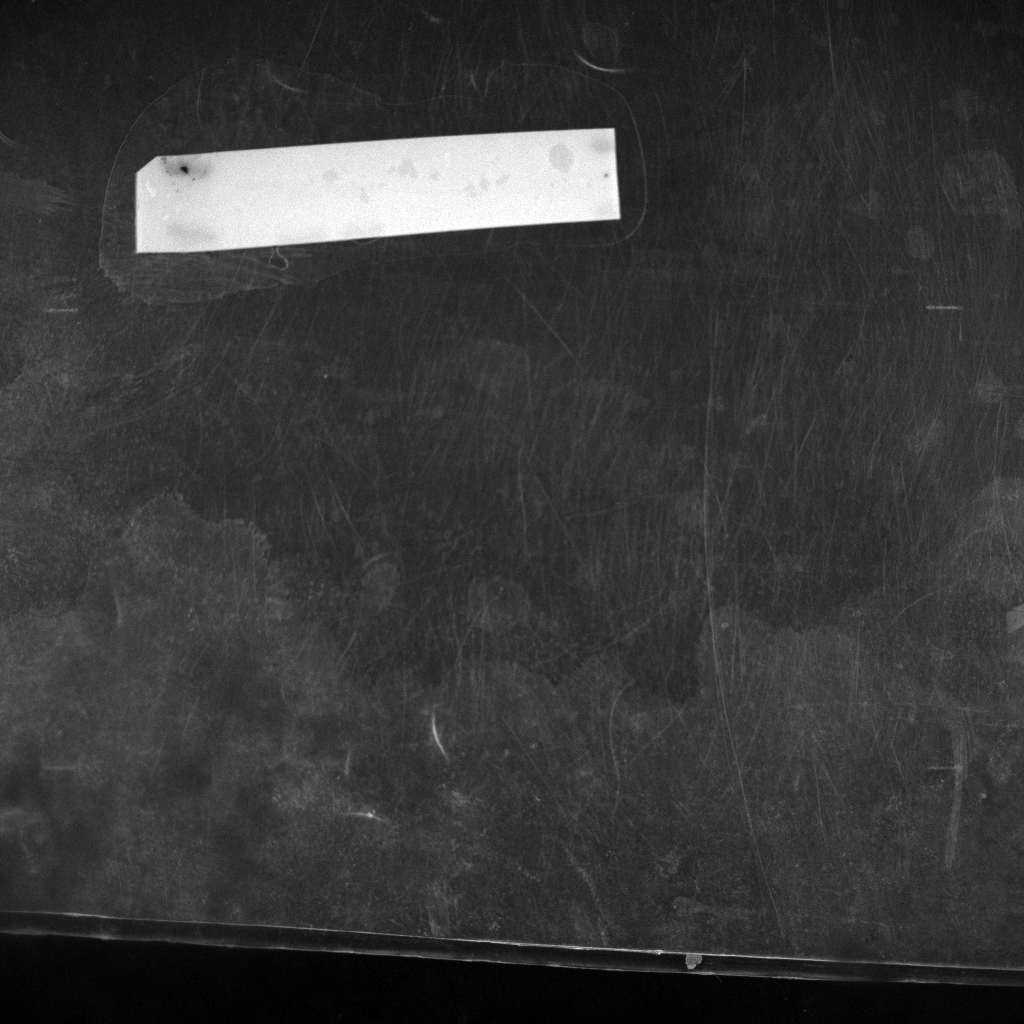

Supplement: Supplementary file 7 — Source Data Fig. 4 [file 44318_2023_3_MOESM7_ESM.zip › Figure4/4d/yh2ax RAD9A -IP V NC siDC1-1 (INPUT IP) w .tif]

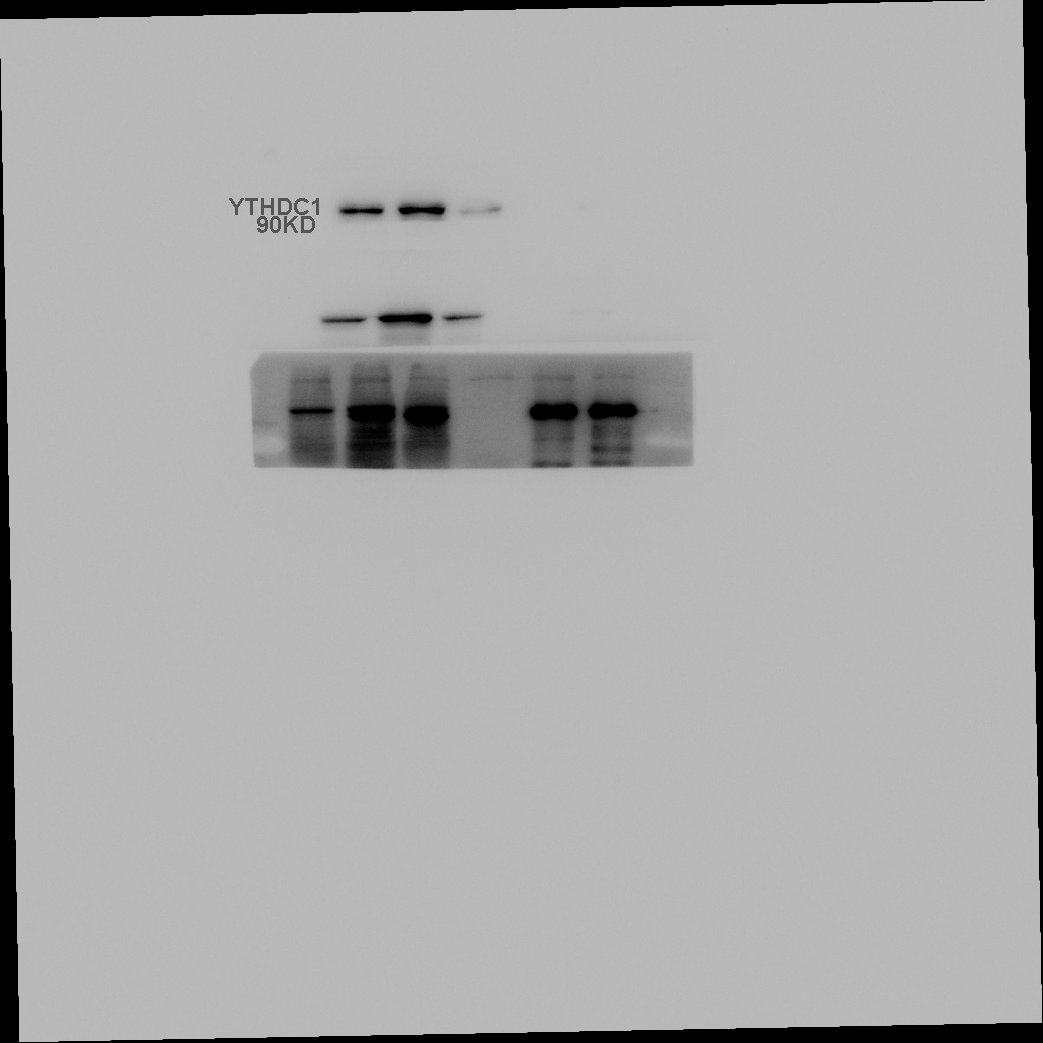

Supplement: Supplementary file 7 — Source Data Fig. 4 [file 44318_2023_3_MOESM7_ESM.zip › Figure4/4d/YTHDC1 up rad9-IP V NC siDC1-1 (INPUT IP) .jpg]

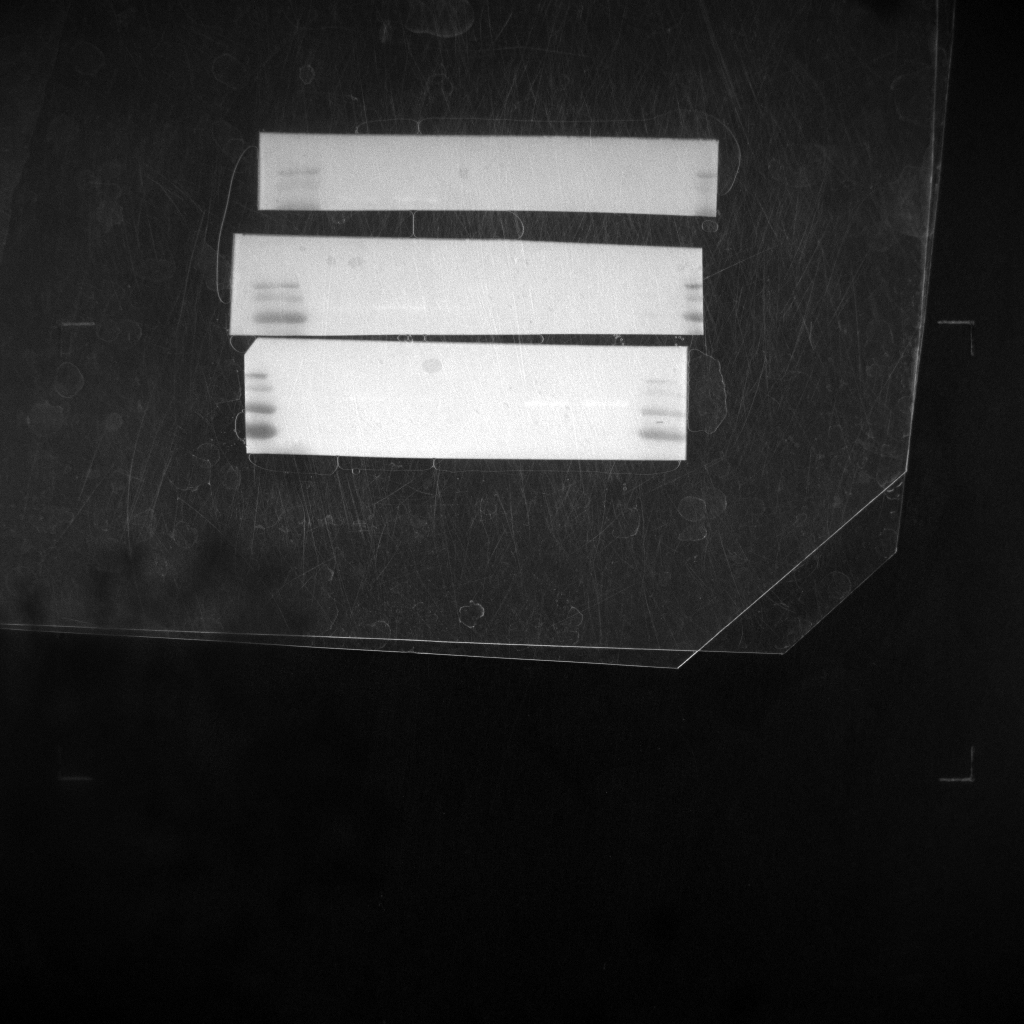

Supplement: Supplementary file 7 — Source Data Fig. 4 [file 44318_2023_3_MOESM7_ESM.zip › Figure4/4d/YTHDC1 up rad9-IP V NC siDC1-1 (INPUT IP) w .tif]

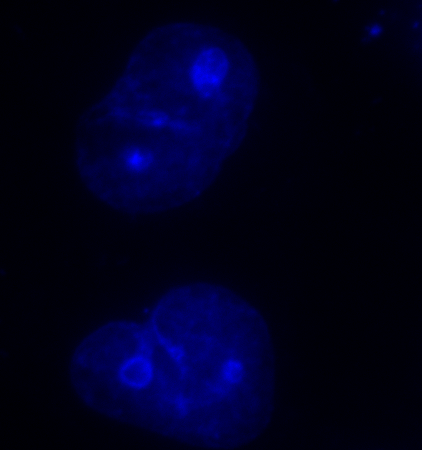

Supplement: Supplementary file 7 — Source Data Fig. 4 [file 44318_2023_3_MOESM7_ESM.zip › Figure4/4e-g/NC Topbp1 and yh2ax IF/Image0001_DAPI.TIF]

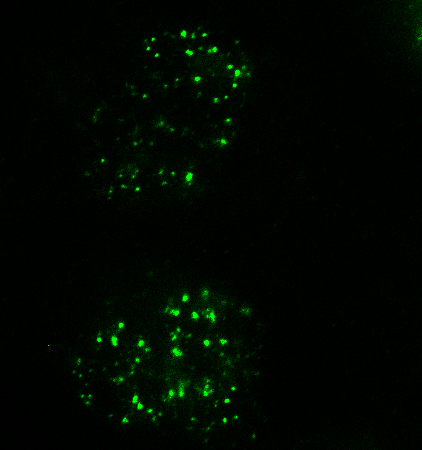

Supplement: Supplementary file 7 — Source Data Fig. 4 [file 44318_2023_3_MOESM7_ESM.zip › Figure4/4e-g/NC Topbp1 and yh2ax IF/Image0001_FITC.TIF]

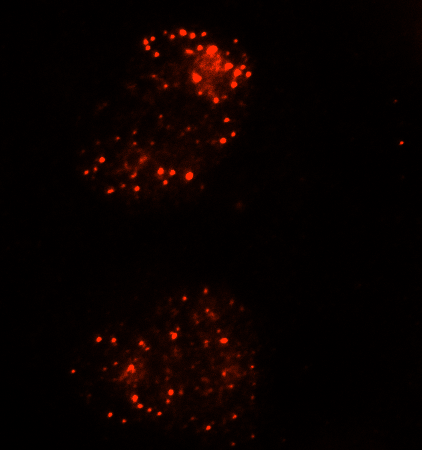

Supplement: Supplementary file 7 — Source Data Fig. 4 [file 44318_2023_3_MOESM7_ESM.zip › Figure4/4e-g/NC Topbp1 and yh2ax IF/Image0001_Texasred.TIF]

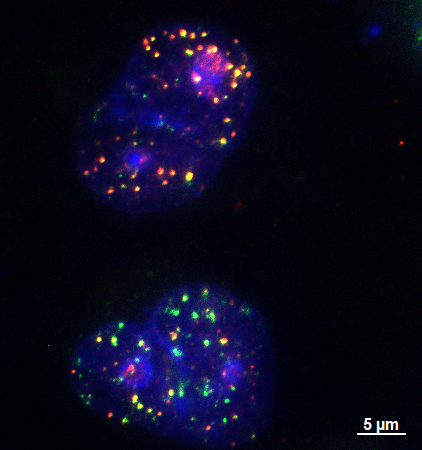

Supplement: Supplementary file 7 — Source Data Fig. 4 [file 44318_2023_3_MOESM7_ESM.zip › Figure4/4e-g/NC Topbp1 and yh2ax IF/MERGE.TIF]

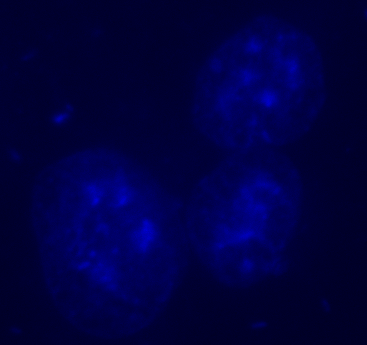

Supplement: Supplementary file 7 — Source Data Fig. 4 [file 44318_2023_3_MOESM7_ESM.zip › Figure4/4e-g/siYTHDC1-1 Topbp1 and yh2ax IF/Image0004_DAPI.TIF]

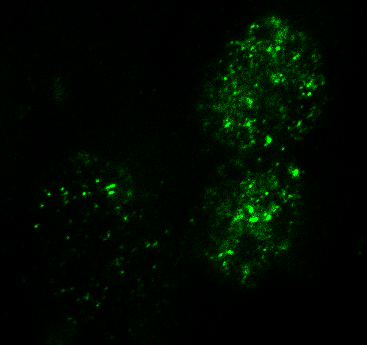

Supplement: Supplementary file 7 — Source Data Fig. 4 [file 44318_2023_3_MOESM7_ESM.zip › Figure4/4e-g/siYTHDC1-1 Topbp1 and yh2ax IF/Image0004_FITC.TIF]

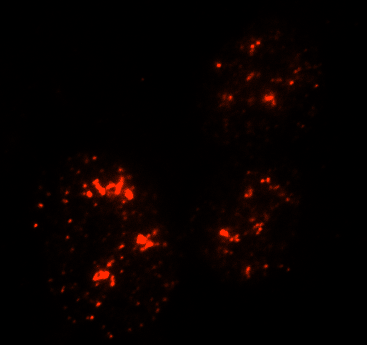

Supplement: Supplementary file 7 — Source Data Fig. 4 [file 44318_2023_3_MOESM7_ESM.zip › Figure4/4e-g/siYTHDC1-1 Topbp1 and yh2ax IF/Image0004_Texasred.TIF]

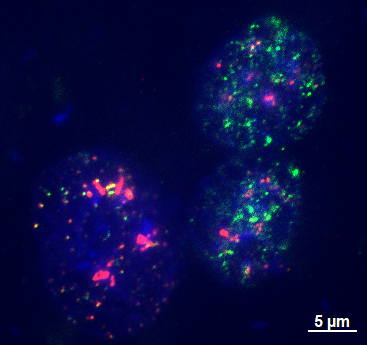

Supplement: Supplementary file 7 — Source Data Fig. 4 [file 44318_2023_3_MOESM7_ESM.zip › Figure4/4e-g/siYTHDC1-1 Topbp1 and yh2ax IF/MERGE.TIF]

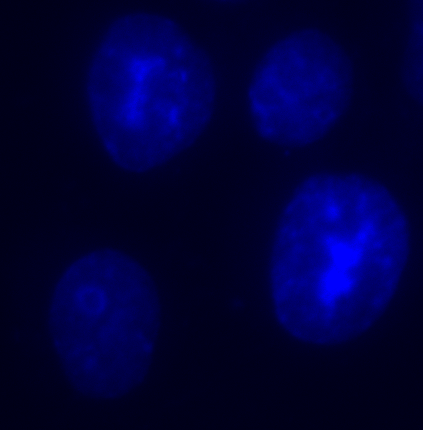

Supplement: Supplementary file 7 — Source Data Fig. 4 [file 44318_2023_3_MOESM7_ESM.zip › Figure4/4e-g/siYTHDC1-2 Topbp1 and yh2ax IF/Image0007_DAPI.TIF]

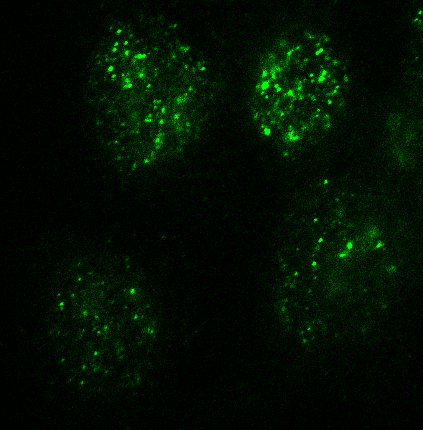

Supplement: Supplementary file 7 — Source Data Fig. 4 [file 44318_2023_3_MOESM7_ESM.zip › Figure4/4e-g/siYTHDC1-2 Topbp1 and yh2ax IF/Image0007_FITC.TIF]

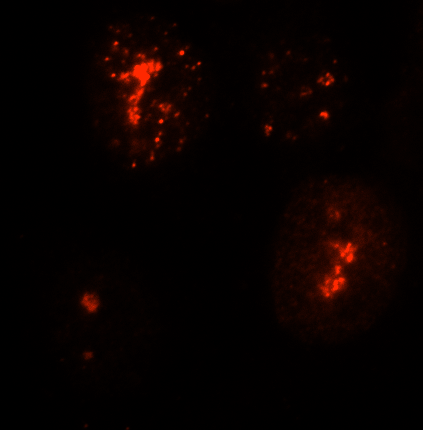

Supplement: Supplementary file 7 — Source Data Fig. 4 [file 44318_2023_3_MOESM7_ESM.zip › Figure4/4e-g/siYTHDC1-2 Topbp1 and yh2ax IF/Image0007_Texasred.TIF]

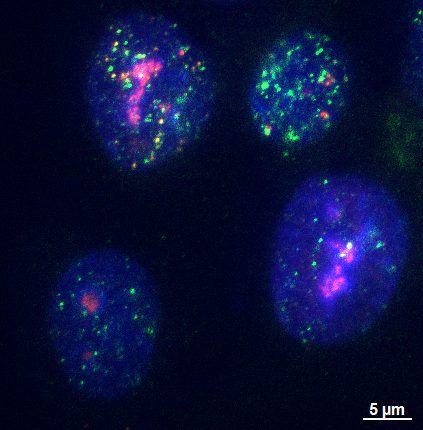

Supplement: Supplementary file 7 — Source Data Fig. 4 [file 44318_2023_3_MOESM7_ESM.zip › Figure4/4e-g/siYTHDC1-2 Topbp1 and yh2ax IF/MERGE.TIF]

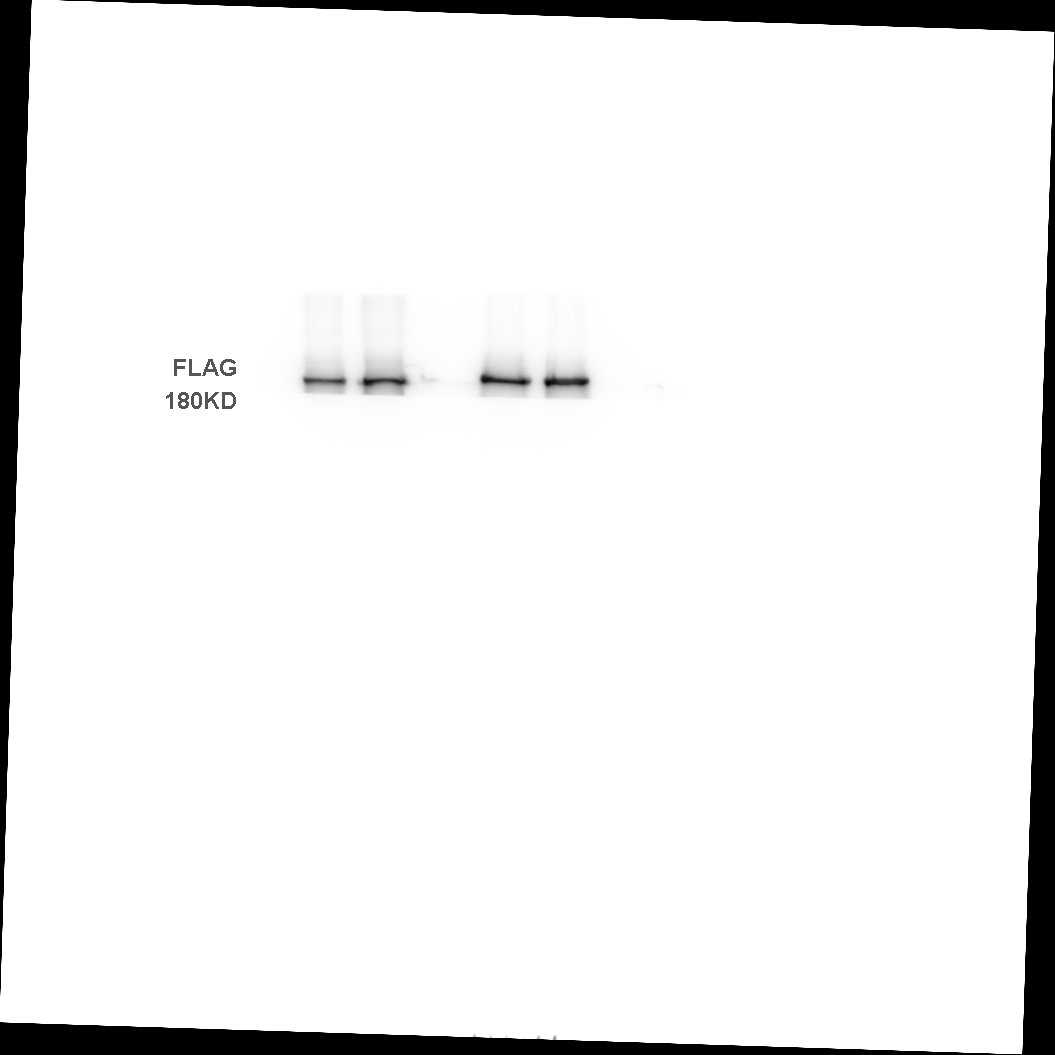

Supplement: Supplementary file 7 — Source Data Fig. 4 [file 44318_2023_3_MOESM7_ESM.zip › Figure4/4h/Flag V NC siDC1(TOPBP1) input IP .jpg]

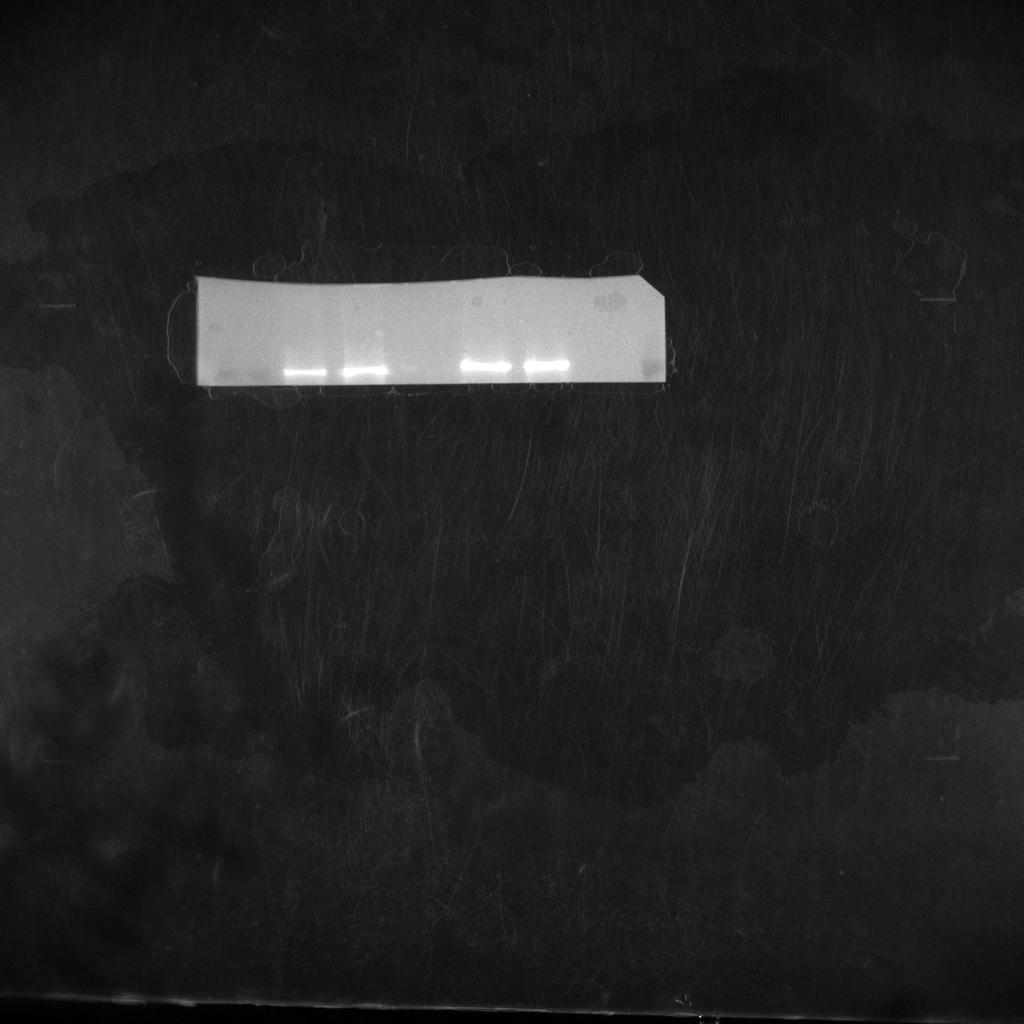

Supplement: Supplementary file 7 — Source Data Fig. 4 [file 44318_2023_3_MOESM7_ESM.zip › Figure4/4h/Flag V NC siDC1(TOPBP1) input IP W.tif]

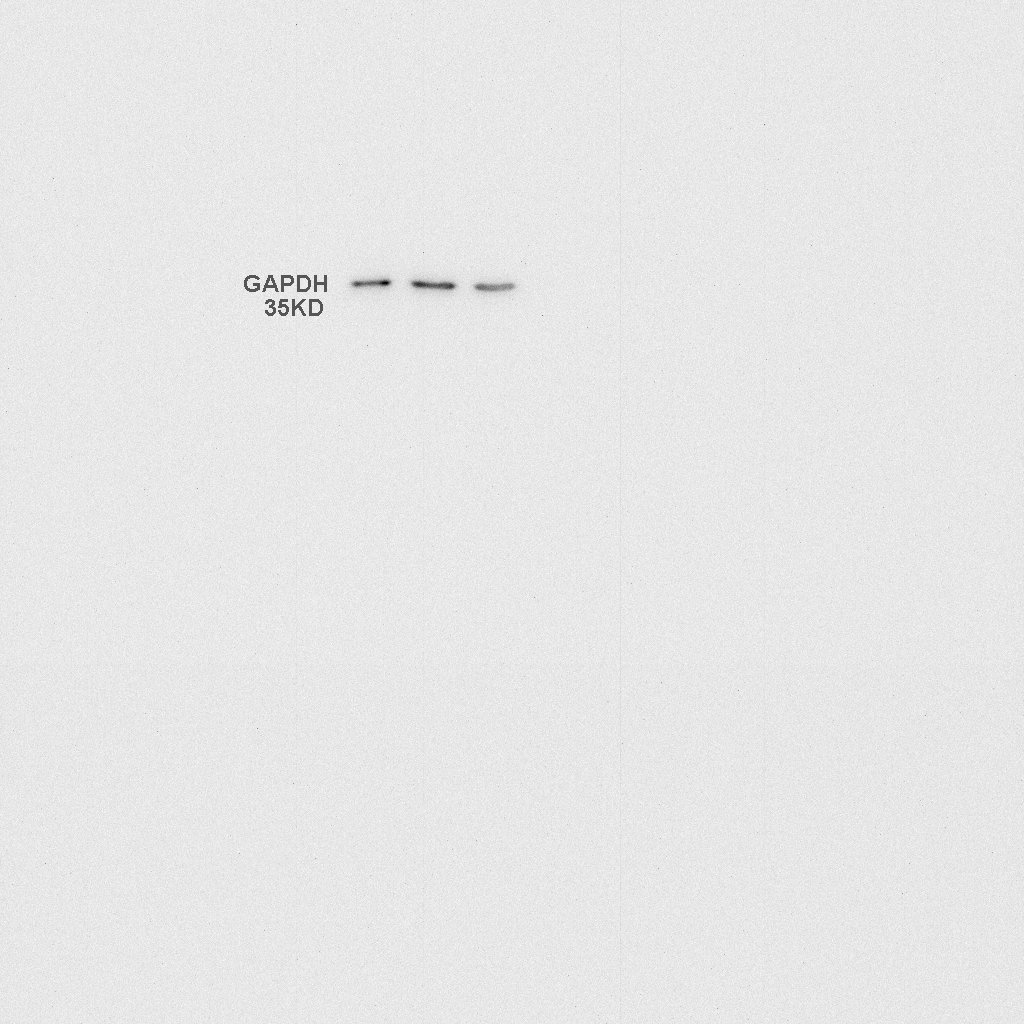

Supplement: Supplementary file 7 — Source Data Fig. 4 [file 44318_2023_3_MOESM7_ESM.zip › Figure4/4h/gapdh V NC siDC1(TOPBP1) input IP .jpg]

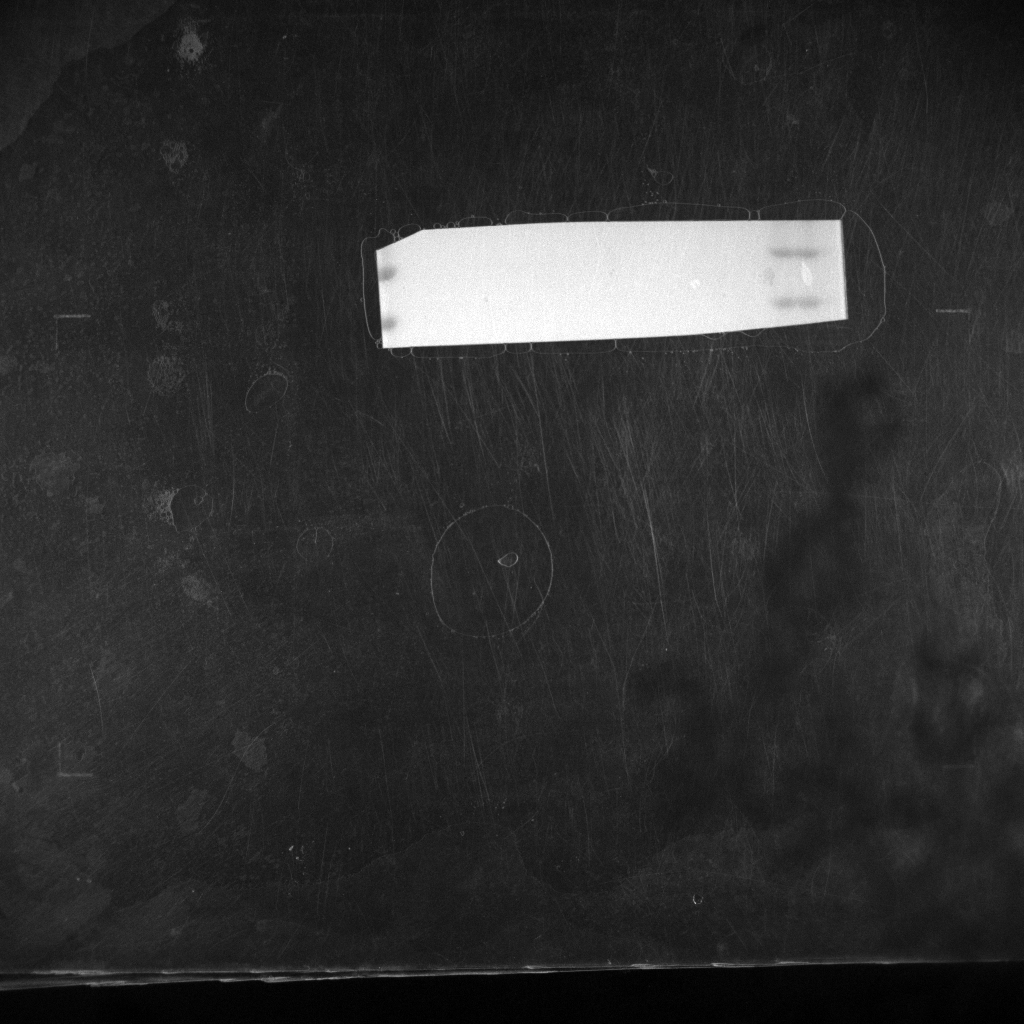

Supplement: Supplementary file 7 — Source Data Fig. 4 [file 44318_2023_3_MOESM7_ESM.zip › Figure4/4h/gapdh V NC siDC1(TOPBP1) input IP w .tif]

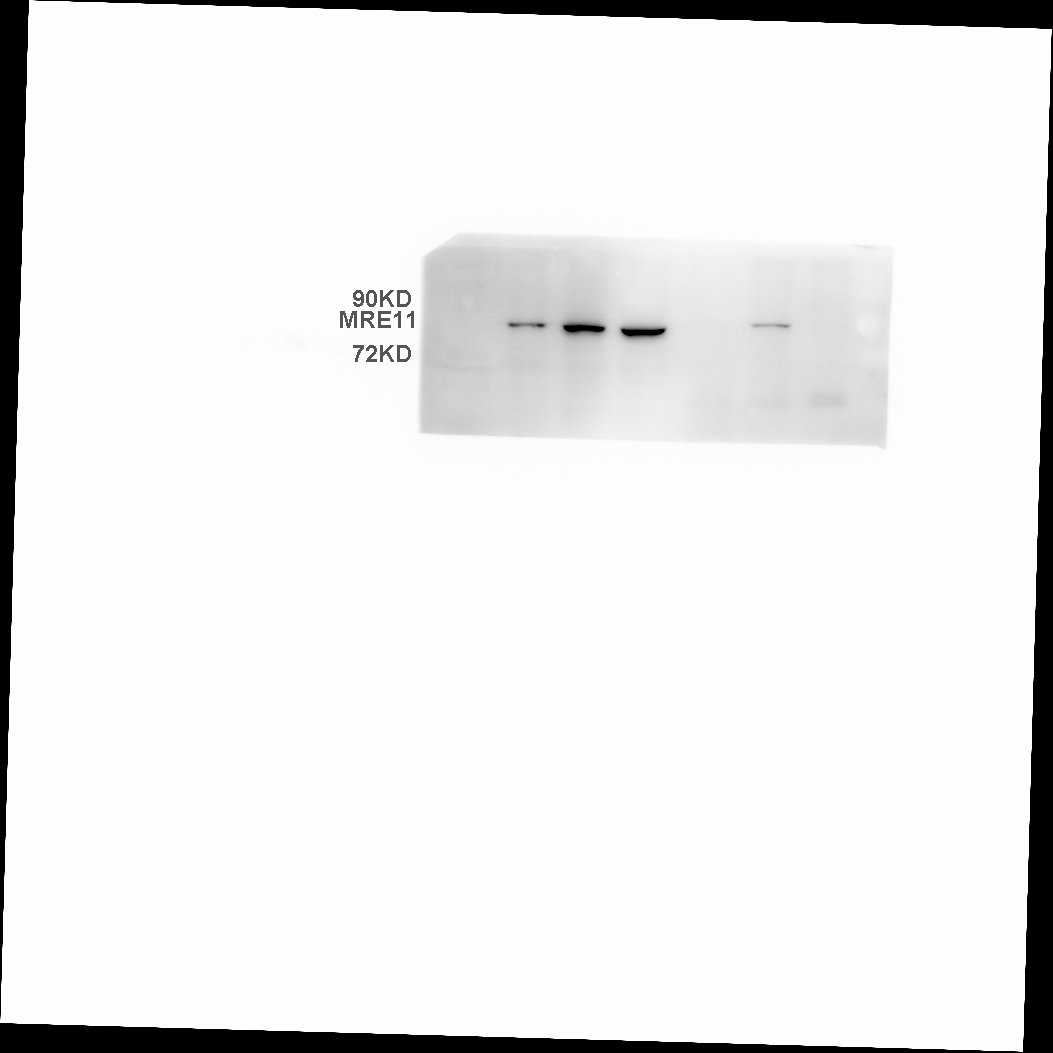

Supplement: Supplementary file 7 — Source Data Fig. 4 [file 44318_2023_3_MOESM7_ESM.zip › Figure4/4h/MRE11 V NC siDC1(TOPBP1) input IP-2 .jpg]

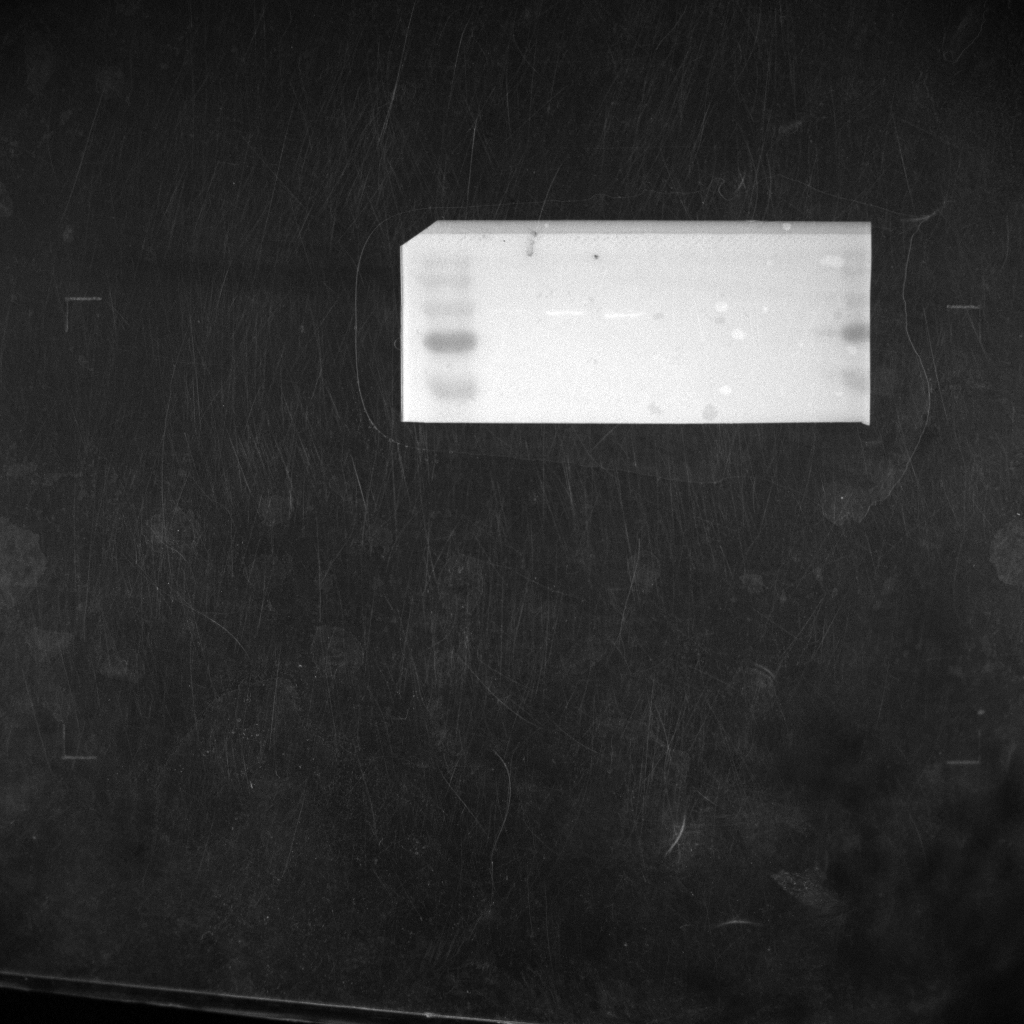

Supplement: Supplementary file 7 — Source Data Fig. 4 [file 44318_2023_3_MOESM7_ESM.zip › Figure4/4h/MRE11 V NC siDC1(TOPBP1) input IP-2 W .tif]

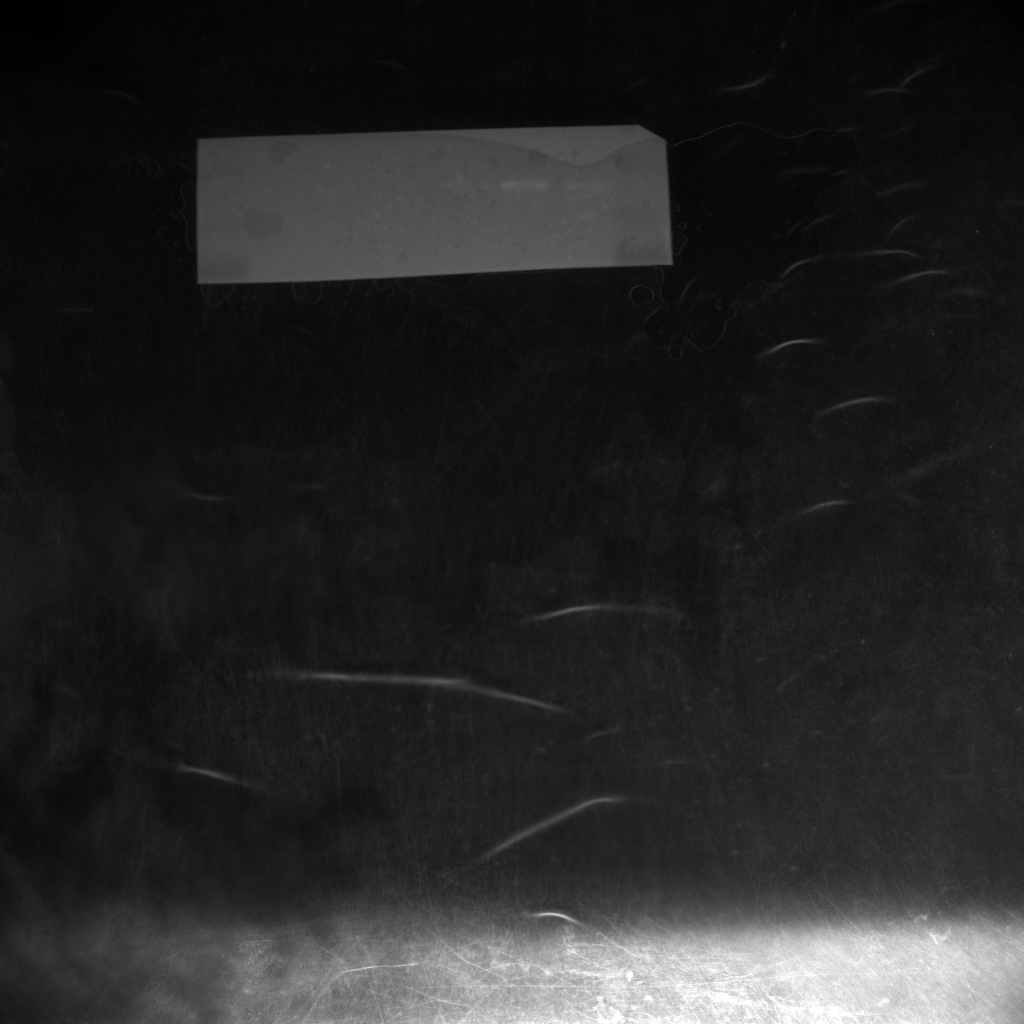

Supplement: Supplementary file 7 — Source Data Fig. 4 [file 44318_2023_3_MOESM7_ESM.zip › Figure4/4h/YTHDC1 V TopBP1 (NC siDC1) (Input IP) -1 W.tif]

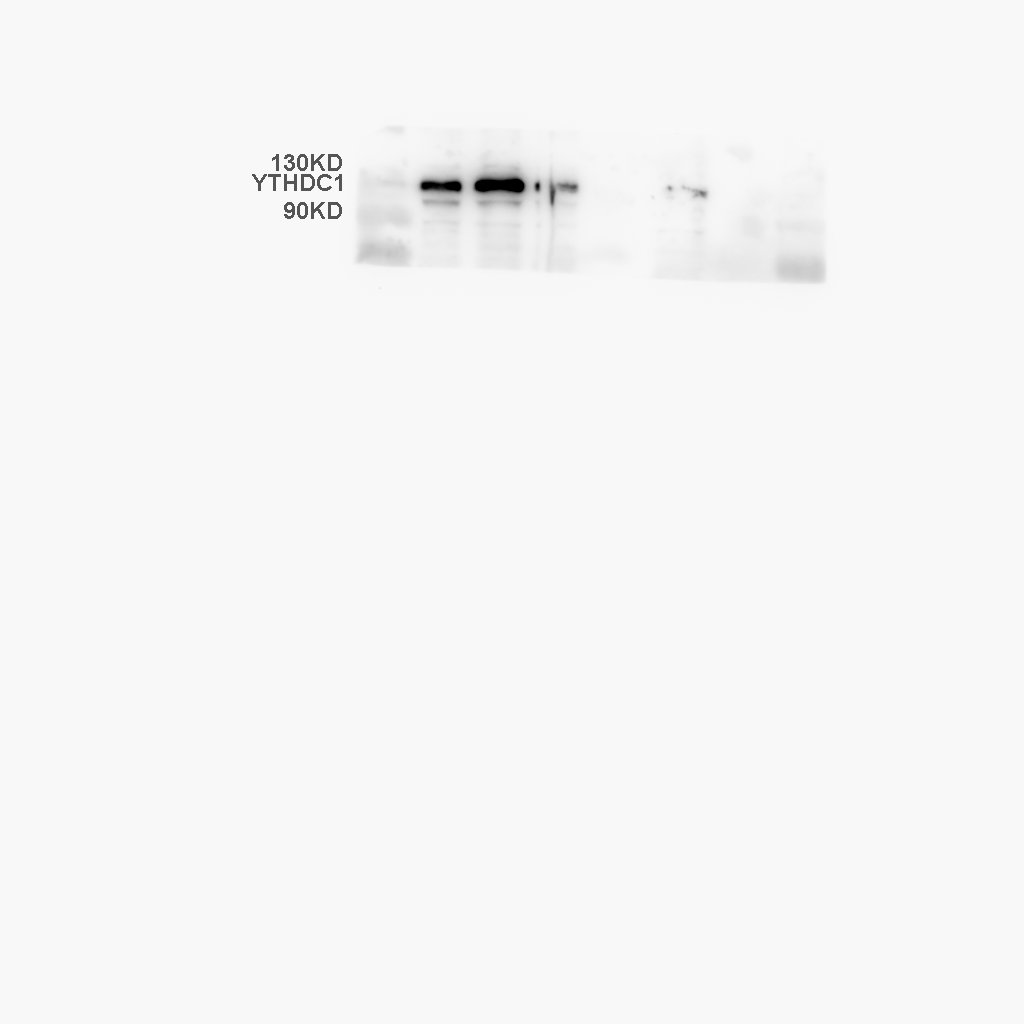

Supplement: Supplementary file 7 — Source Data Fig. 4 [file 44318_2023_3_MOESM7_ESM.zip › Figure4/4h/YTHDC1 right to left V TopBP1 (NC siDC1) (Input IP) -1.jpg]

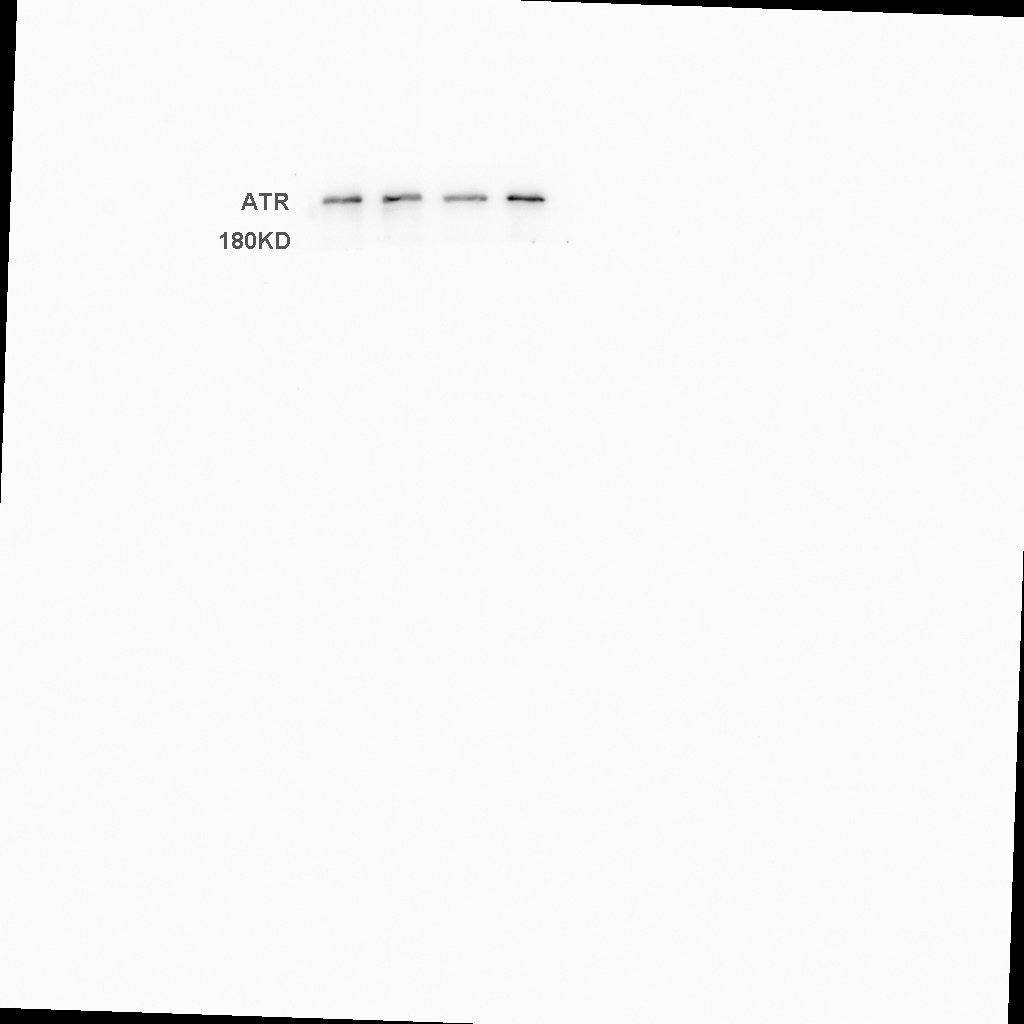

Supplement: Supplementary file 7 — Source Data Fig. 4 [file 44318_2023_3_MOESM7_ESM.zip › Figure4/4i/ATR NC siDC1 siMRE11 siDC1+siRMRE11 .jpg]

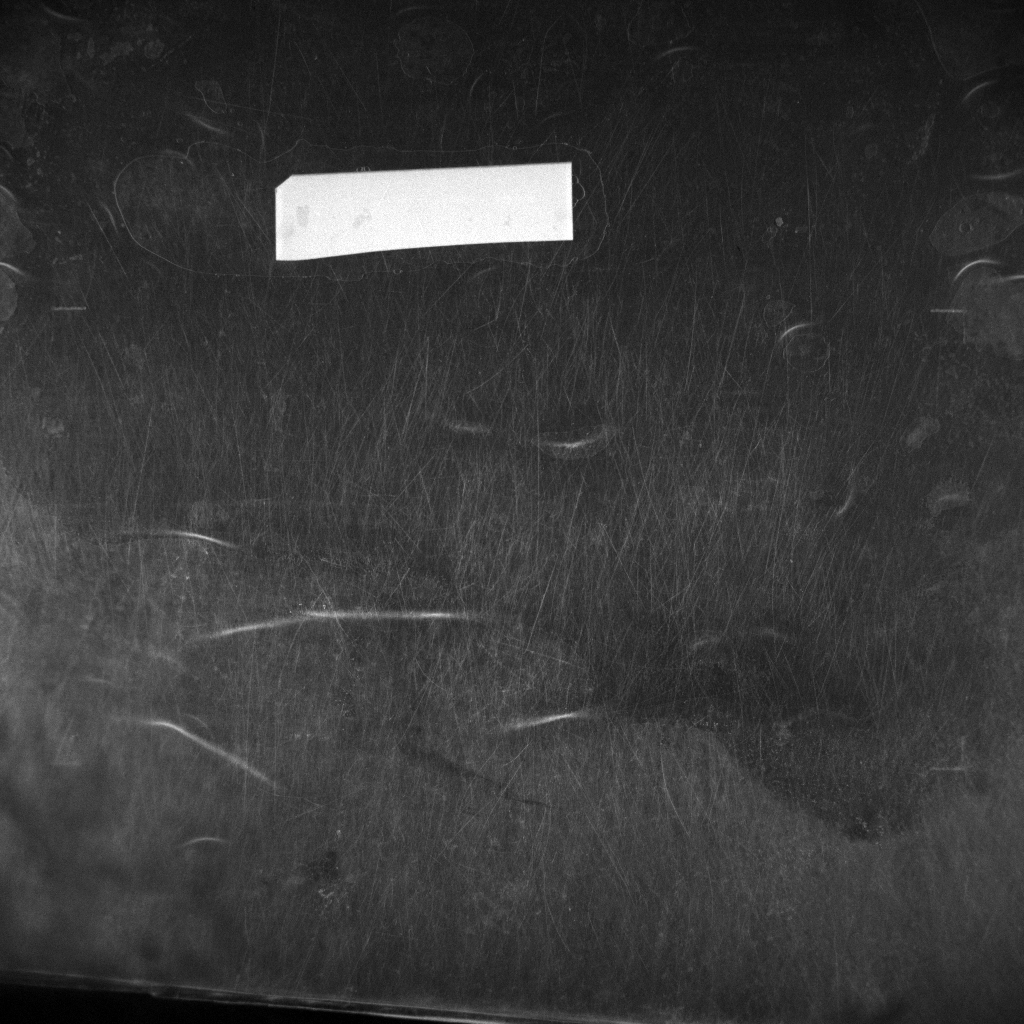

Supplement: Supplementary file 7 — Source Data Fig. 4 [file 44318_2023_3_MOESM7_ESM.zip › Figure4/4i/ATRNC siDC1 siMRE11 siDC1+siRMRE11 W .tif]

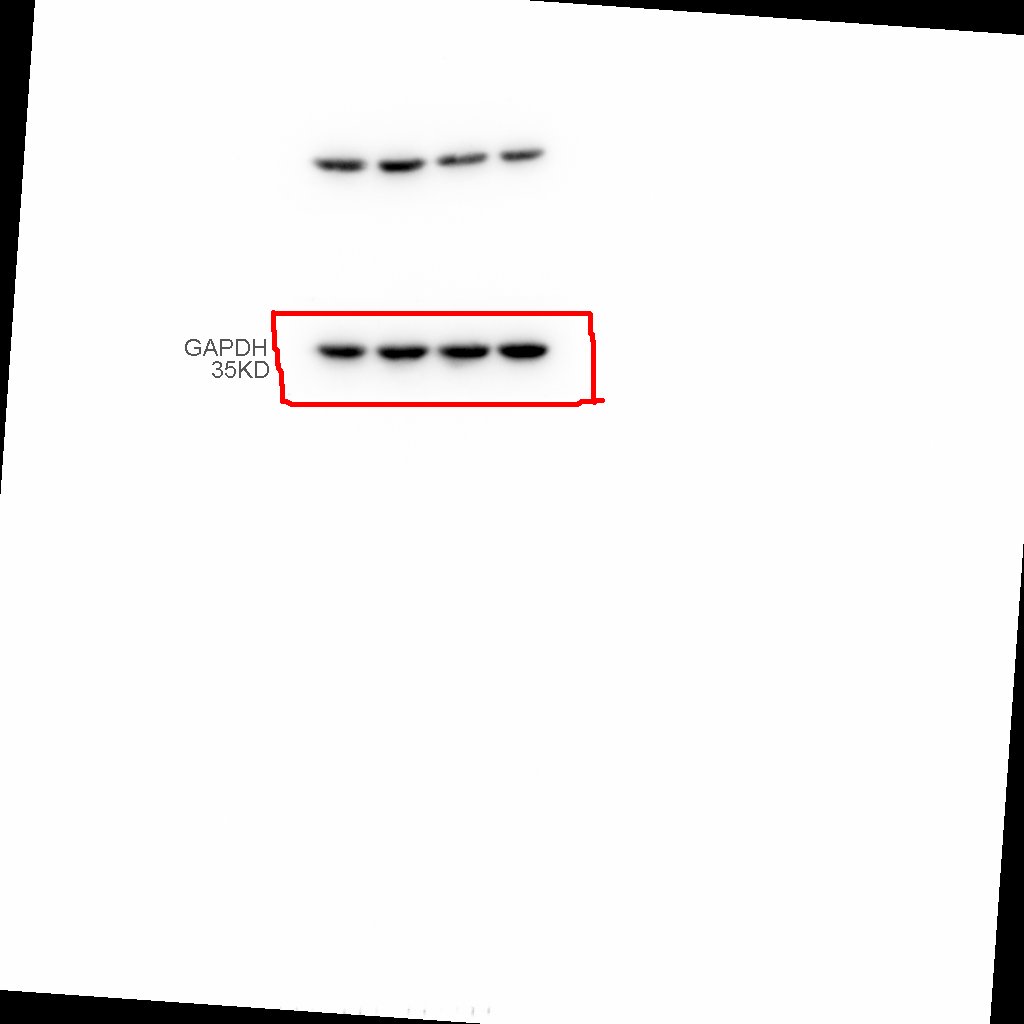

Supplement: Supplementary file 7 — Source Data Fig. 4 [file 44318_2023_3_MOESM7_ESM.zip › Figure4/4i/GAPDH down to up NC siDC1 siMRE11 siDC1+siRMRE11 .jpg]

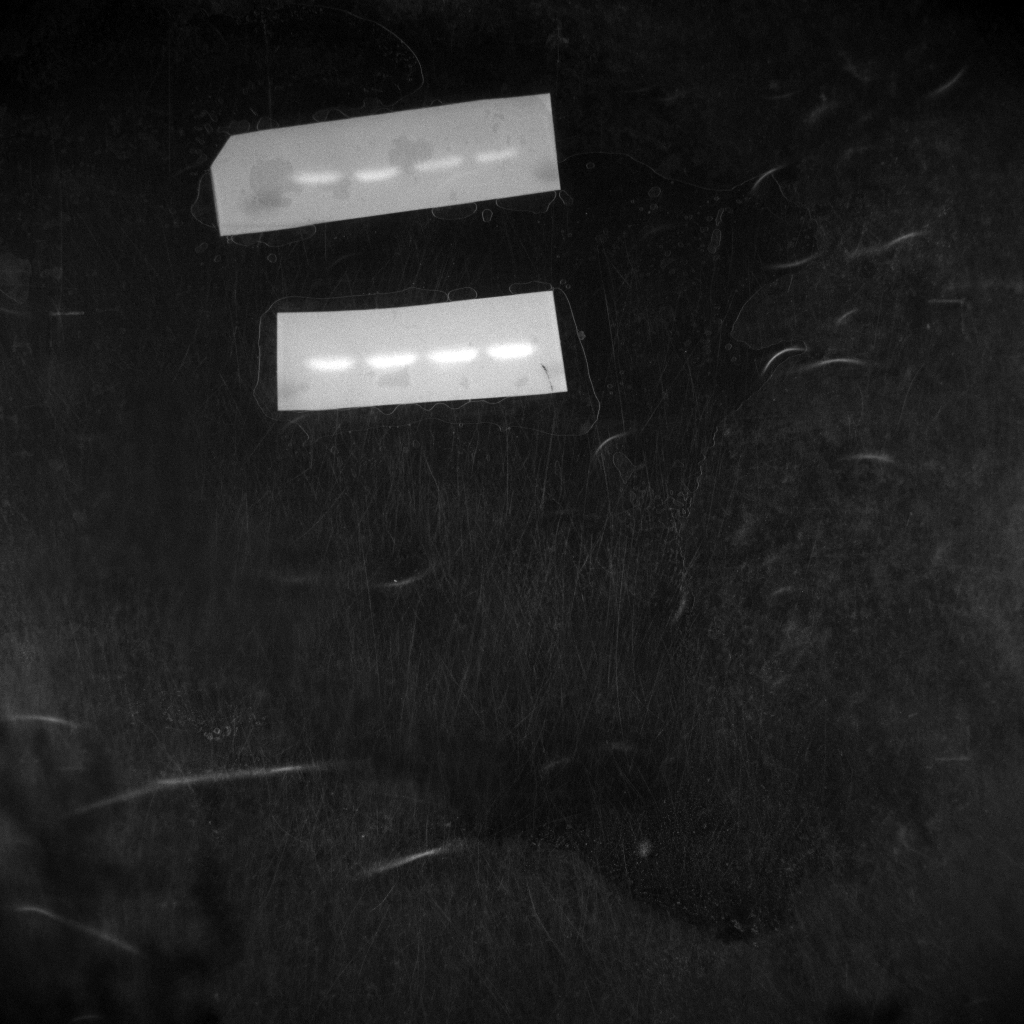

Supplement: Supplementary file 7 — Source Data Fig. 4 [file 44318_2023_3_MOESM7_ESM.zip › Figure4/4i/GAPDH down to upNC siDC1 siMRE11 siDC1+siRMRE11 W.tif]

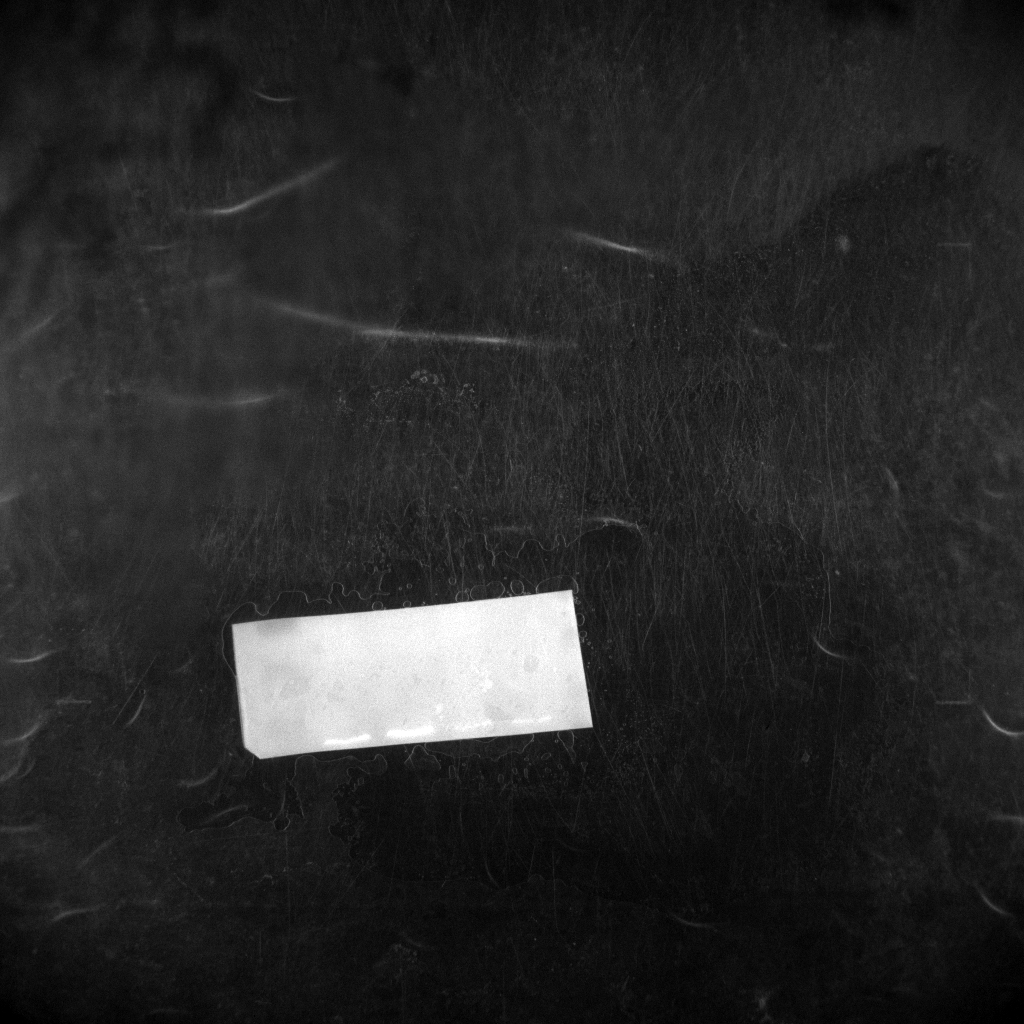

Supplement: Supplementary file 7 — Source Data Fig. 4 [file 44318_2023_3_MOESM7_ESM.zip › Figure4/4i/MRE11 NC siDC1 siMRE11 siDC1+siRMRE11 W.tif]

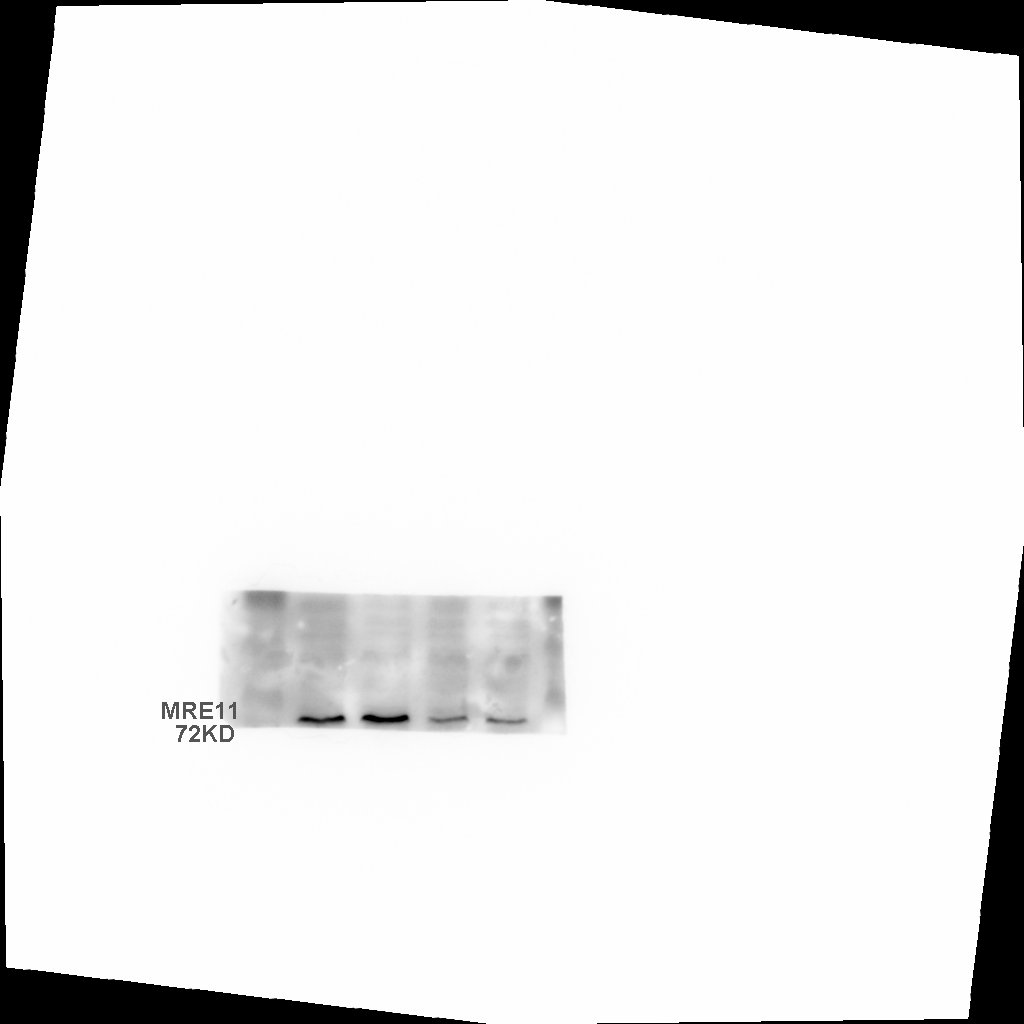

Supplement: Supplementary file 7 — Source Data Fig. 4 [file 44318_2023_3_MOESM7_ESM.zip › Figure4/4i/MRE11 NC siDC1 siMRE11 siDC1+siRMRE11 .jpg]

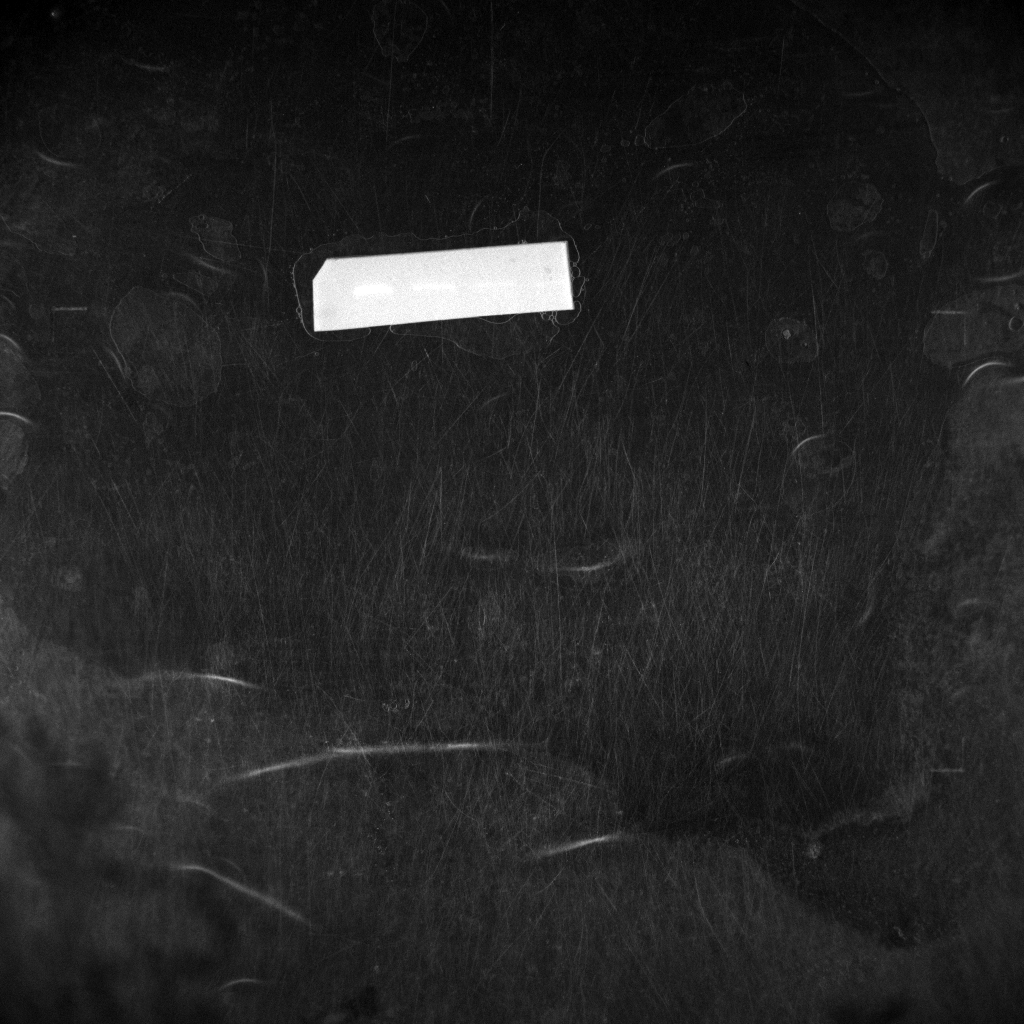

Supplement: Supplementary file 7 — Source Data Fig. 4 [file 44318_2023_3_MOESM7_ESM.zip › Figure4/4i/p-ATR NC siDC1 siMRE11 siDC1+siRMRE11 -2 W .tif]

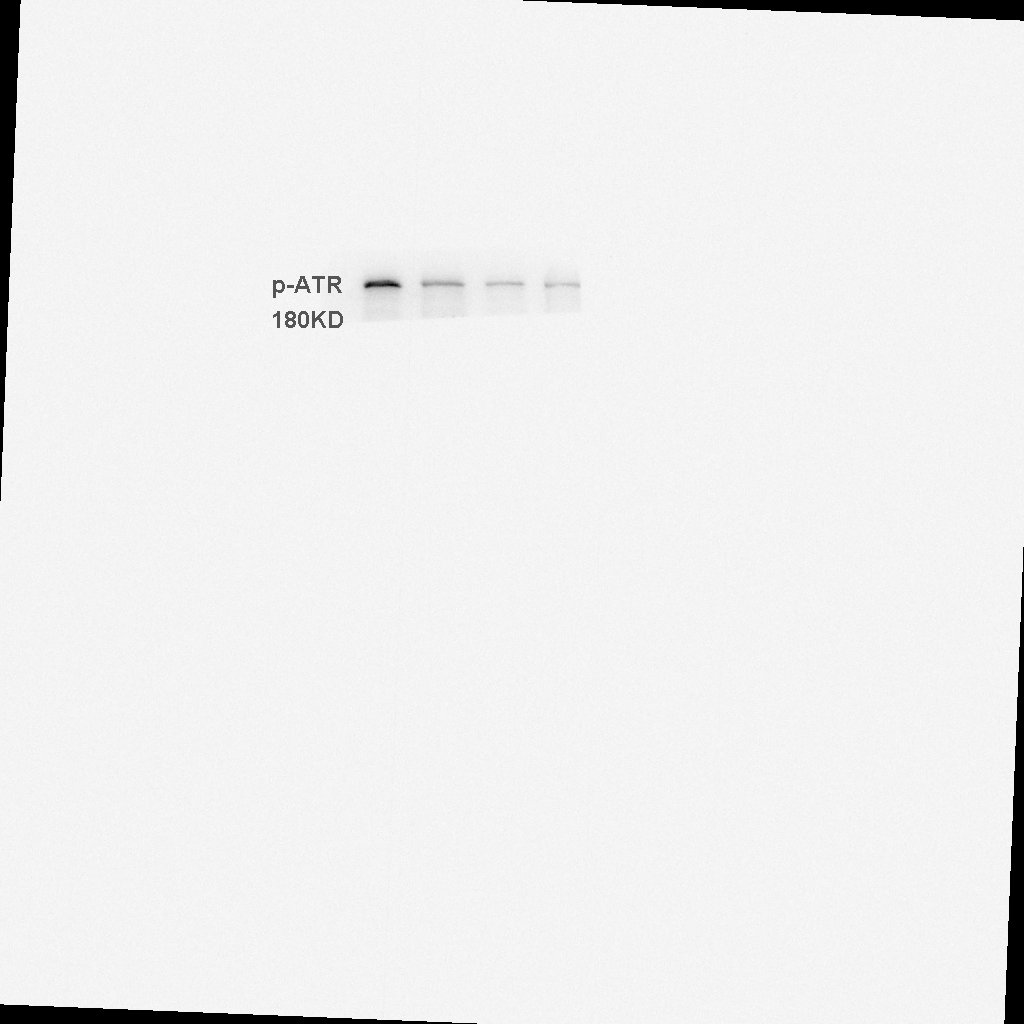

Supplement: Supplementary file 7 — Source Data Fig. 4 [file 44318_2023_3_MOESM7_ESM.zip › Figure4/4i/p-ATR NC siDC1 siMRE11 siDC1+siRMRE11 -2 .jpg]

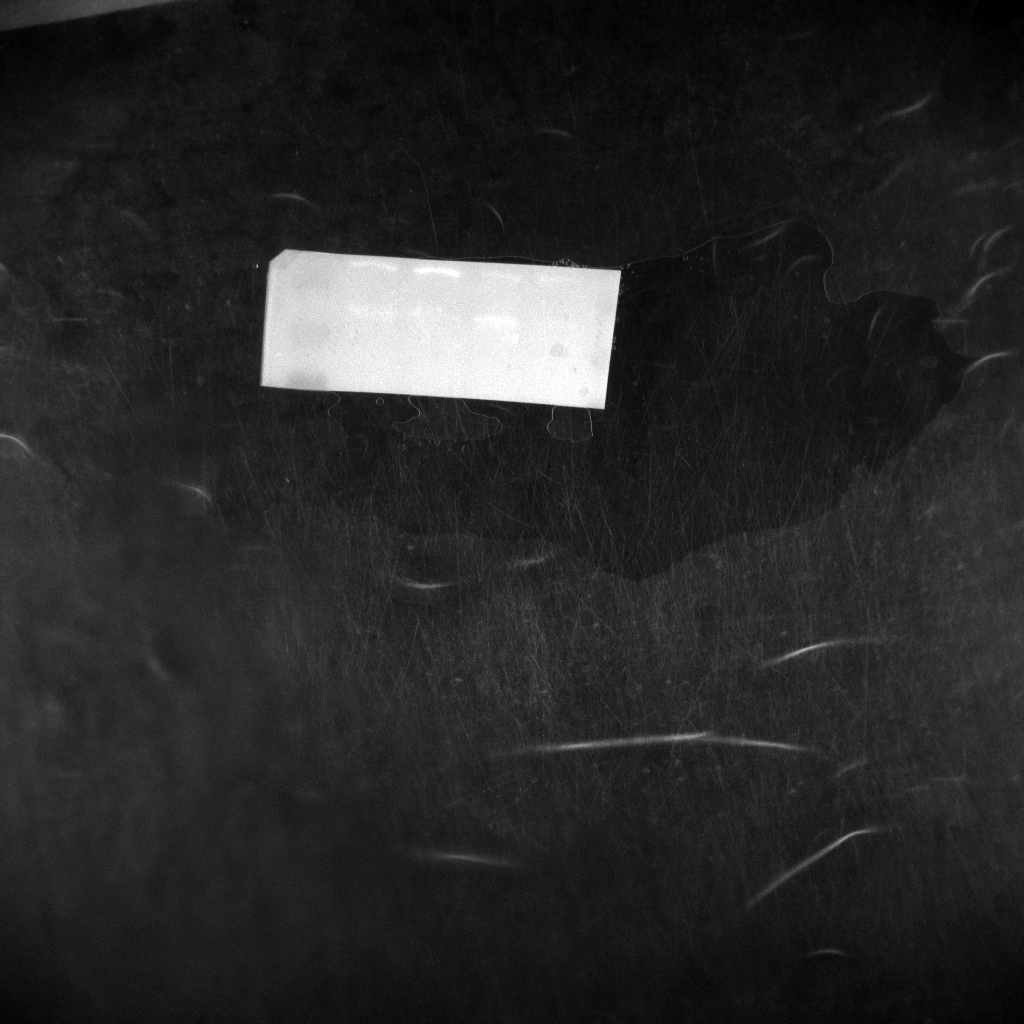

Supplement: Supplementary file 7 — Source Data Fig. 4 [file 44318_2023_3_MOESM7_ESM.zip › Figure4/4i/YTHDC1 NC siDC1 siMRE11 siDC1+siRMRE11 W.tif]

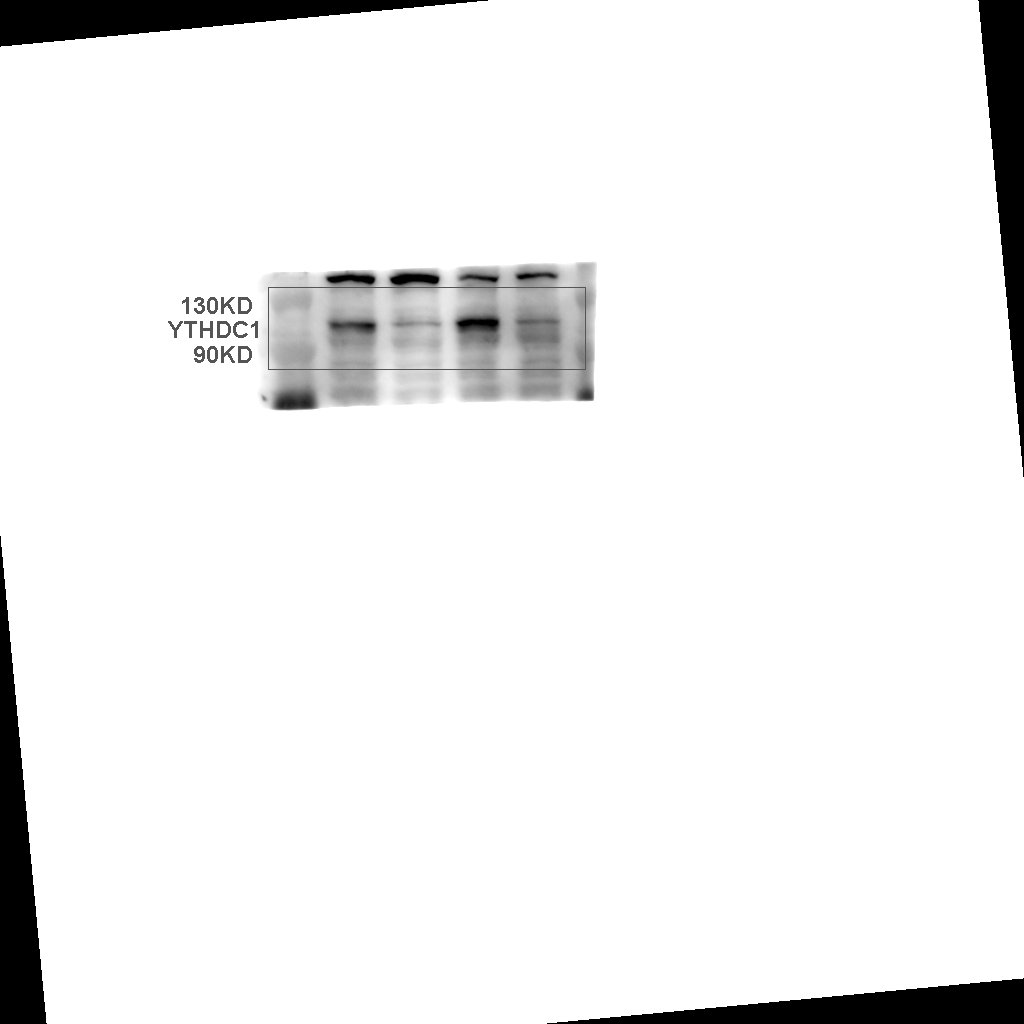

Supplement: Supplementary file 7 — Source Data Fig. 4 [file 44318_2023_3_MOESM7_ESM.zip › Figure4/4i/YTHDC1 NC siDC1 siMRE11 siDC1+siRMRE11.jpg]

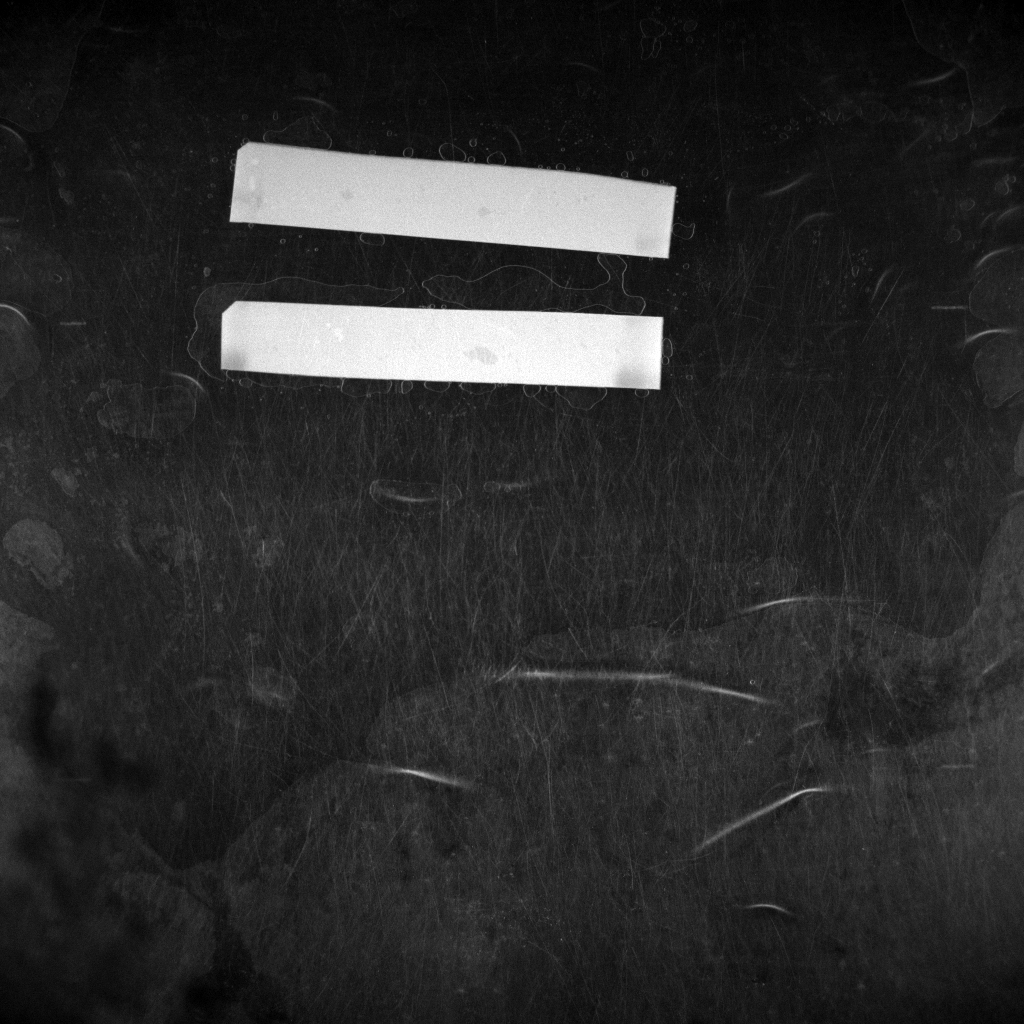

Supplement: Supplementary file 8 — Source Data Fig. 5 [file 44318_2023_3_MOESM8_ESM.zip › Figure5/5a/up topbp1 down mre11 V DC1-DMSO DC1-VP16 (input IP) w.tif]

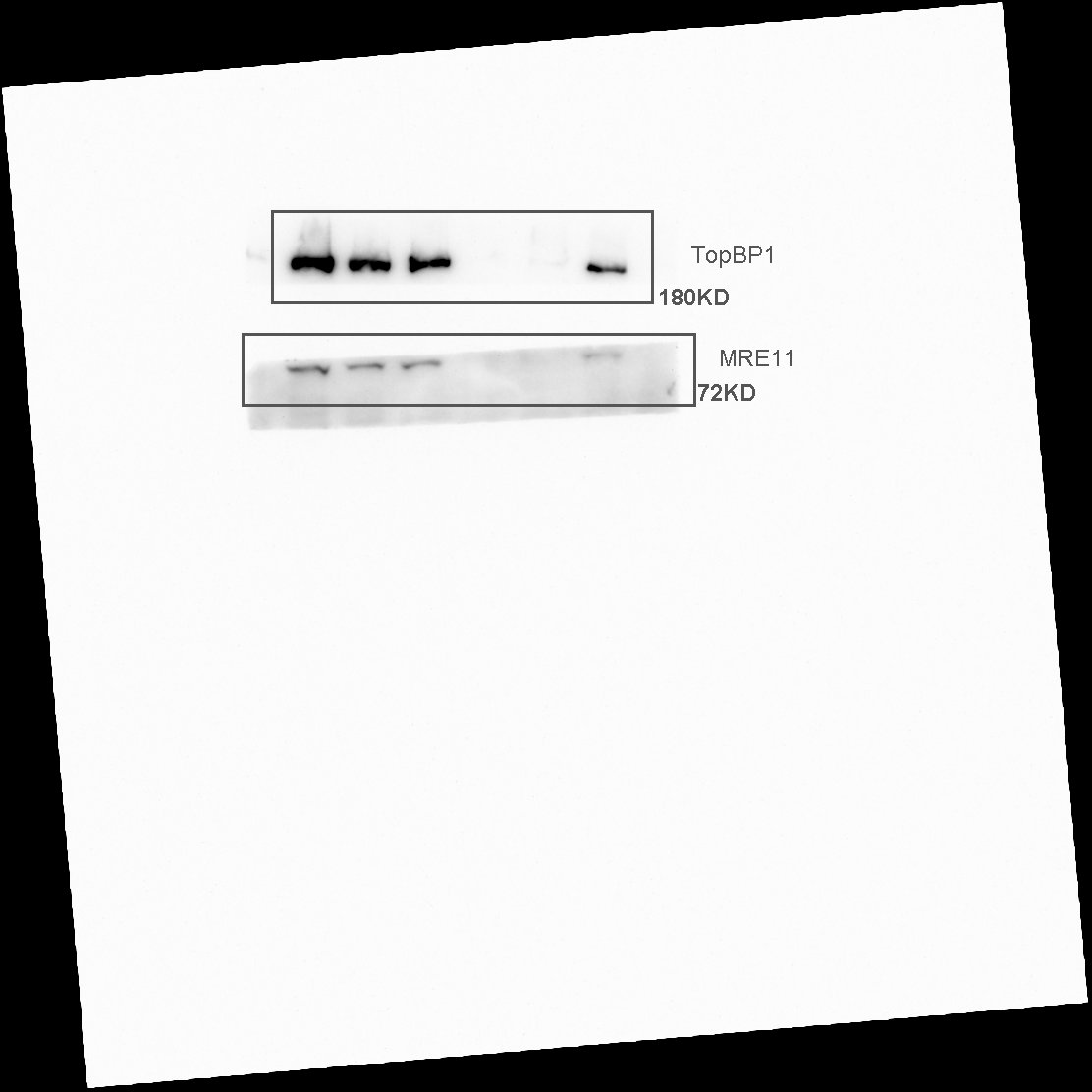

Supplement: Supplementary file 8 — Source Data Fig. 5 [file 44318_2023_3_MOESM8_ESM.zip › Figure5/5a/up topbp1 down mre11 V DC1-DMSO DC1-VP16 (input IP).jpg]

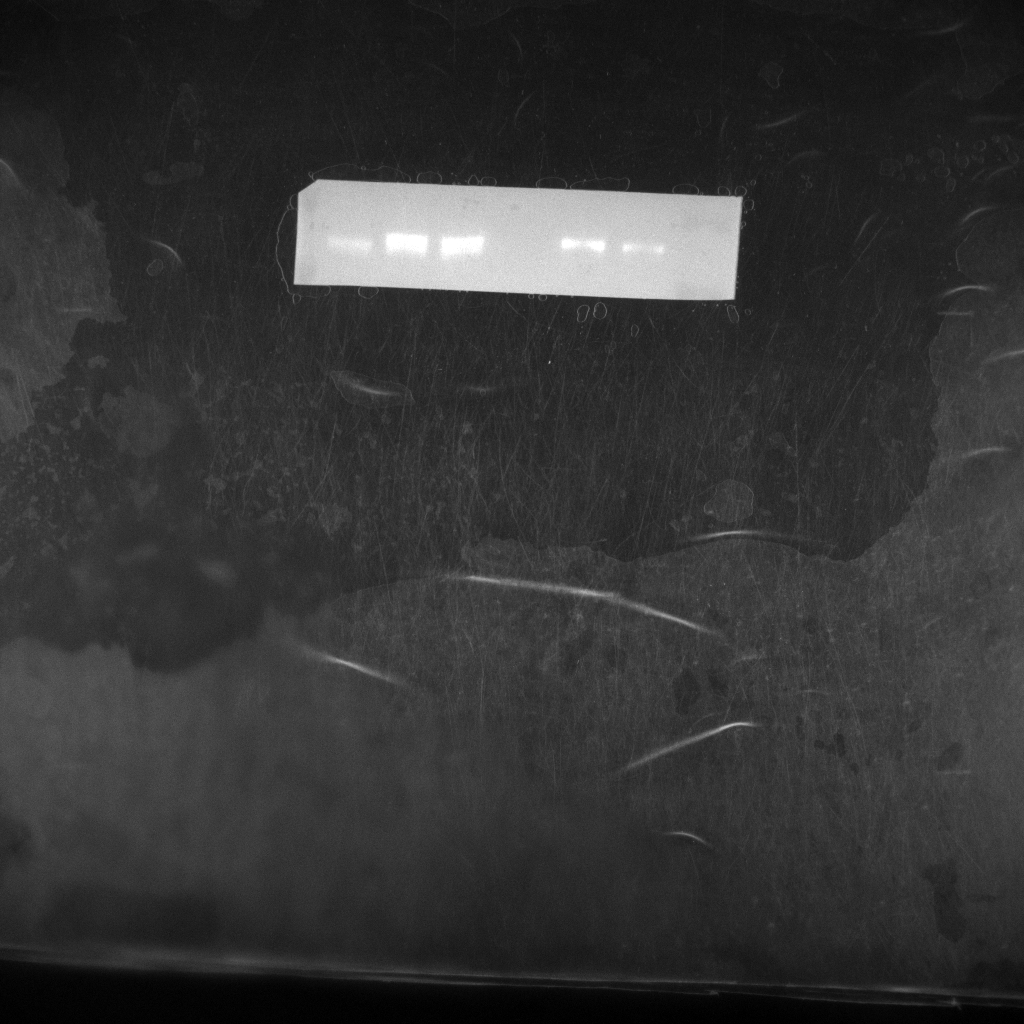

Supplement: Supplementary file 8 — Source Data Fig. 5 [file 44318_2023_3_MOESM8_ESM.zip › Figure5/5a/YTHDC1 V DC1-DMSO DC1-VP16 (input IP) w.tif]

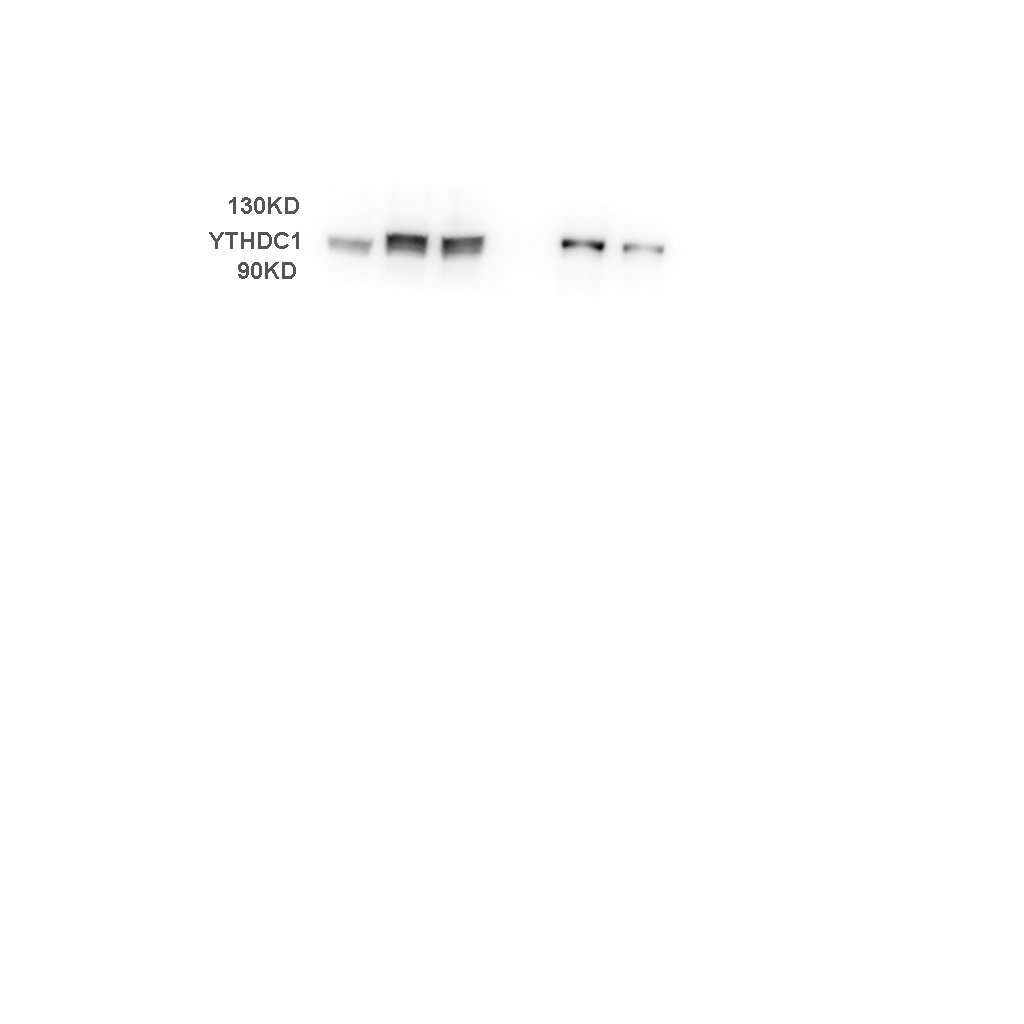

Supplement: Supplementary file 8 — Source Data Fig. 5 [file 44318_2023_3_MOESM8_ESM.zip › Figure5/5a/YTHDC1 V DC1-DMSO DC1-VP16 (input IP).jpg]

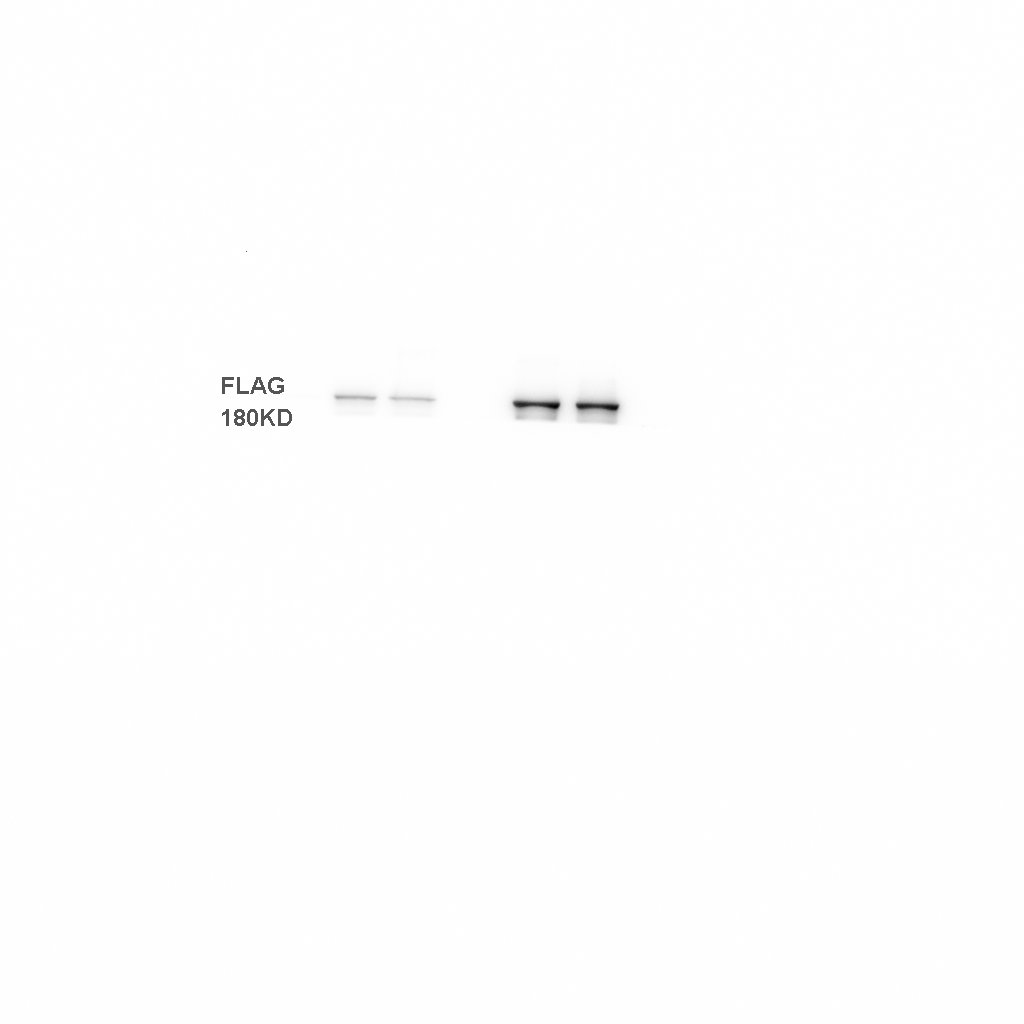

Supplement: Supplementary file 8 — Source Data Fig. 5 [file 44318_2023_3_MOESM8_ESM.zip › Figure5/5b/flag topbp1-IP V topbp1-DMSO topbp1-VP16 (INPUT IP) .jpg]

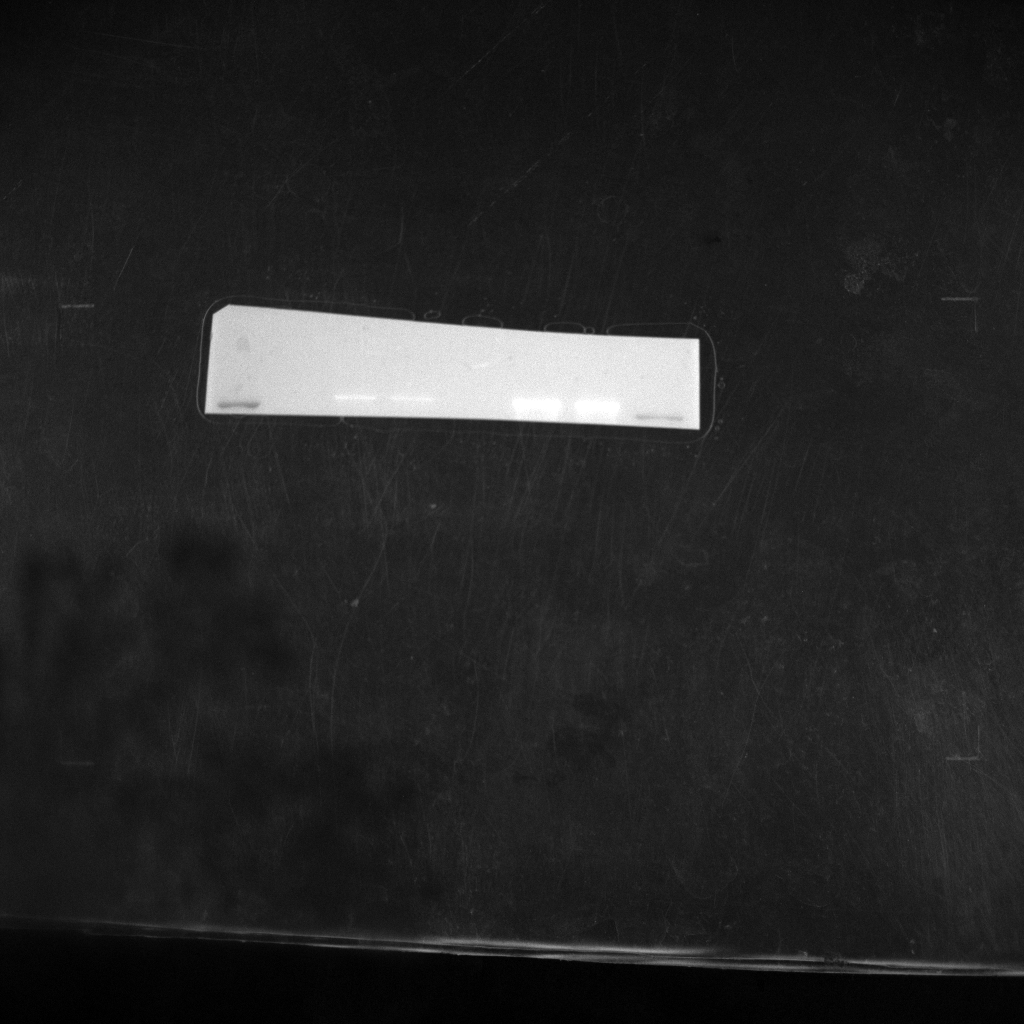

Supplement: Supplementary file 8 — Source Data Fig. 5 [file 44318_2023_3_MOESM8_ESM.zip › Figure5/5b/flag topbp1-IP V topbp1-DMSO topbp1-VP16 (INPUT IP) w .tif]

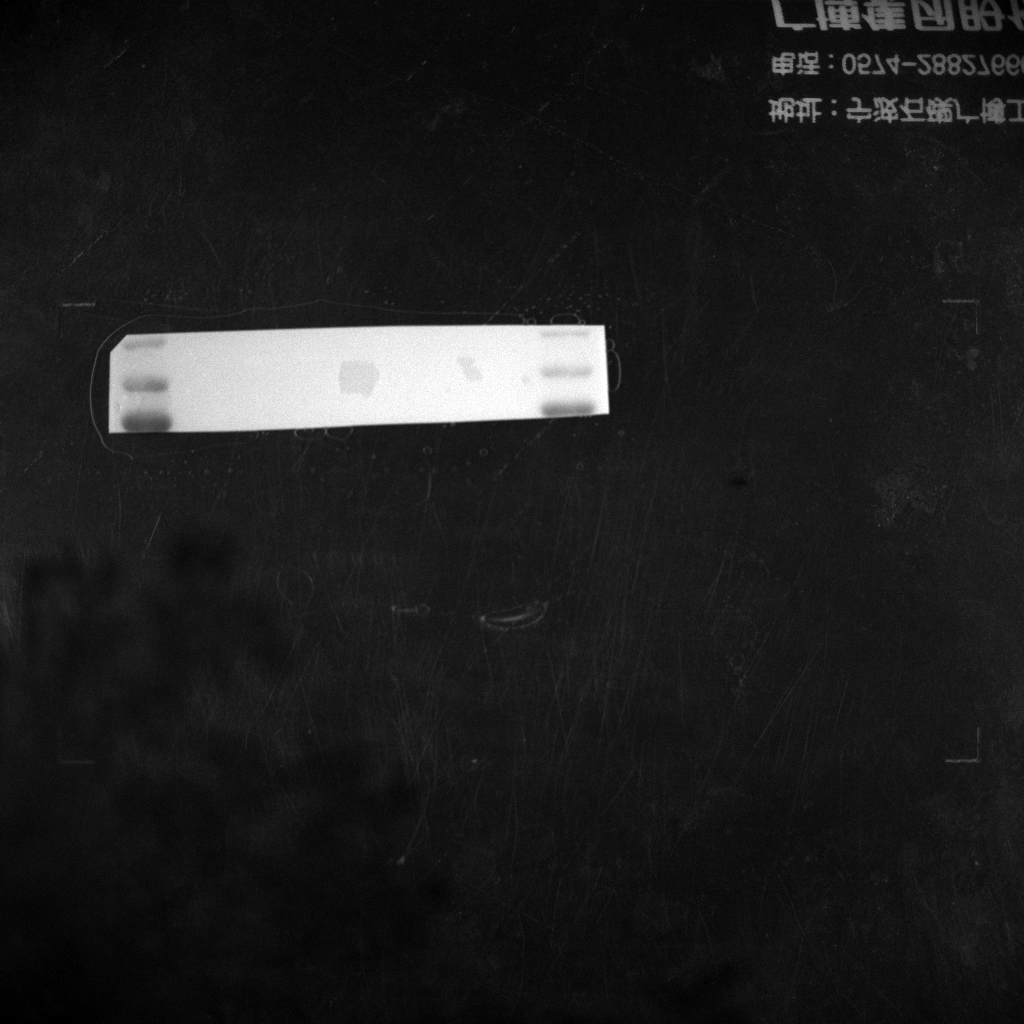

Supplement: Supplementary file 8 — Source Data Fig. 5 [file 44318_2023_3_MOESM8_ESM.zip › Figure5/5b/YTHDC1 topbp1-IP V topbp1-DMSO topbp1-VP16 (INPUT IP) w.tif]

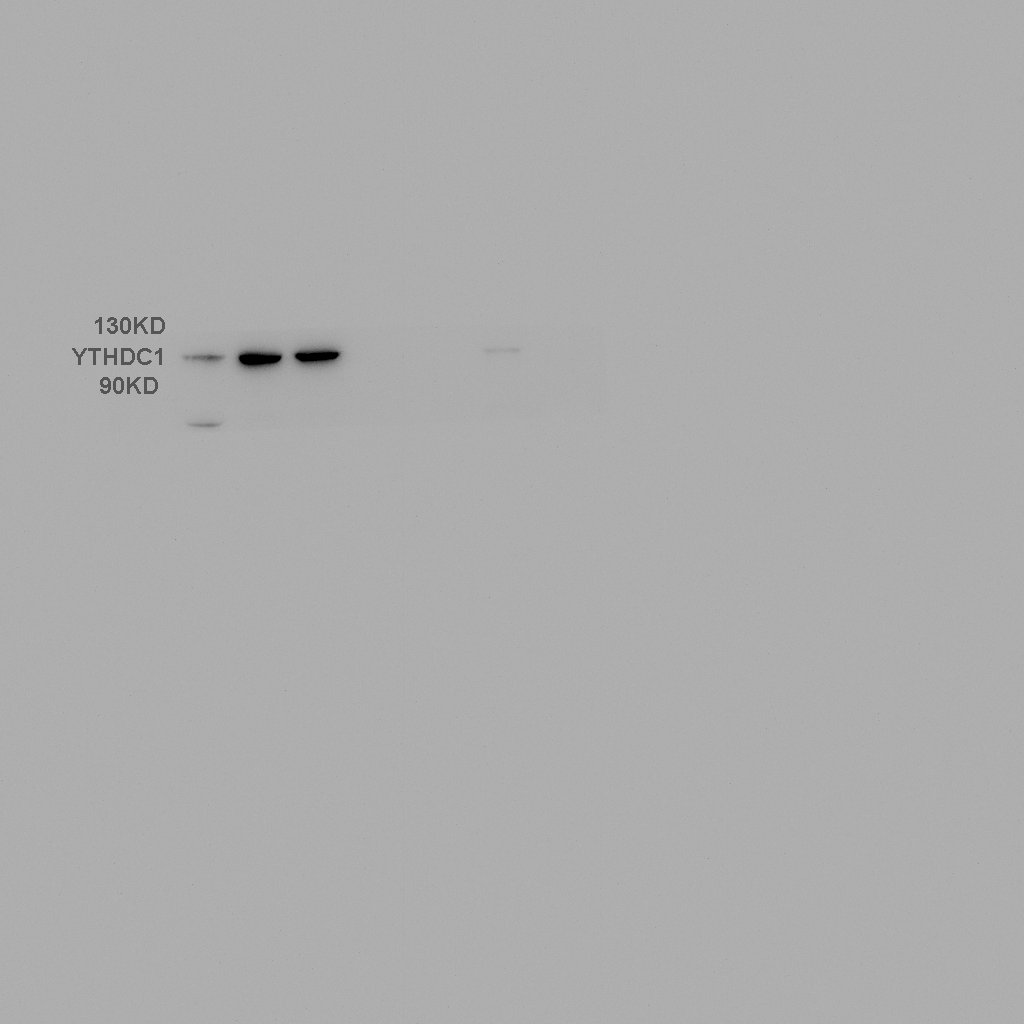

Supplement: Supplementary file 8 — Source Data Fig. 5 [file 44318_2023_3_MOESM8_ESM.zip › Figure5/5b/YTHDC1 topbp1-IP V topbp1-DMSO topbp1-VP16 (INPUT IP).jpg]

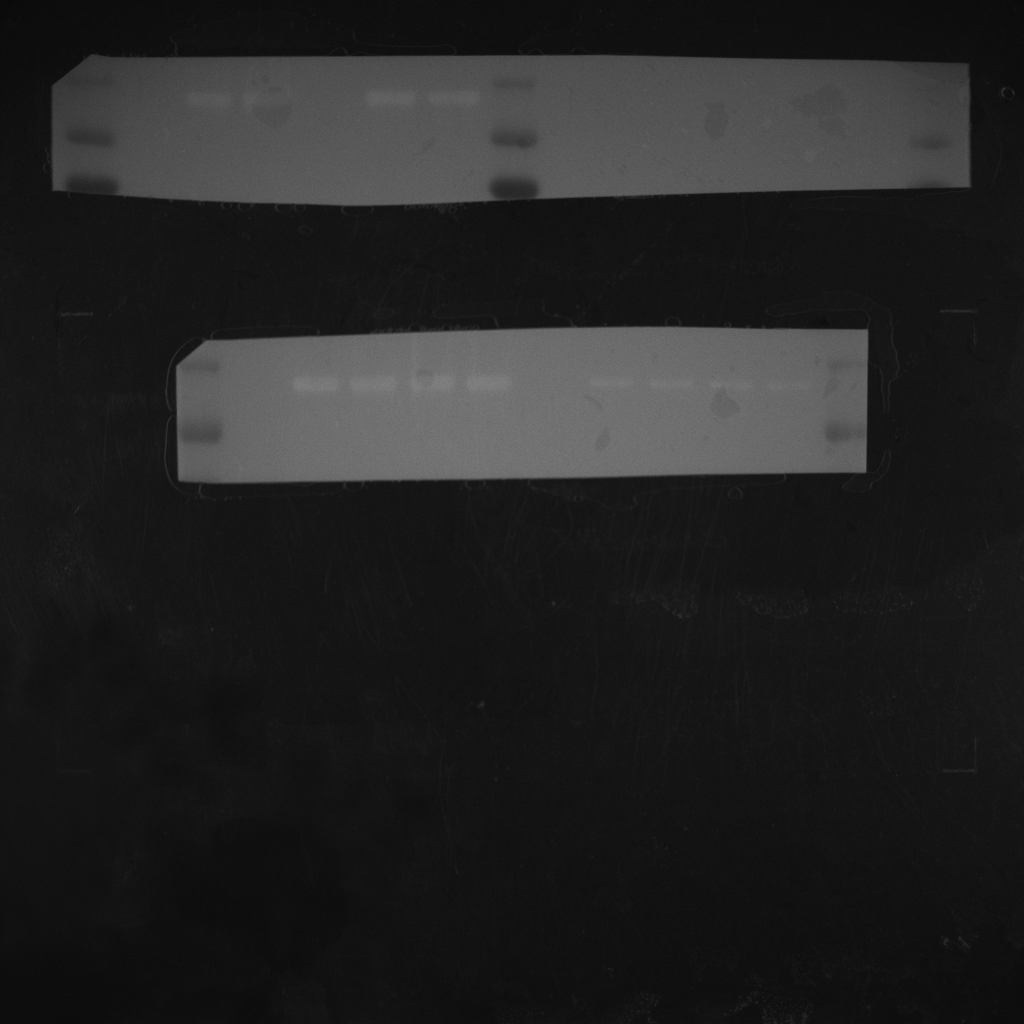

Supplement: Supplementary file 8 — Source Data Fig. 5 [file 44318_2023_3_MOESM8_ESM.zip › Figure5/5c/flag down DC1-IP V DC1-WT mut WT-RNase A WT-MBN (VP16 IP input) w.tif]

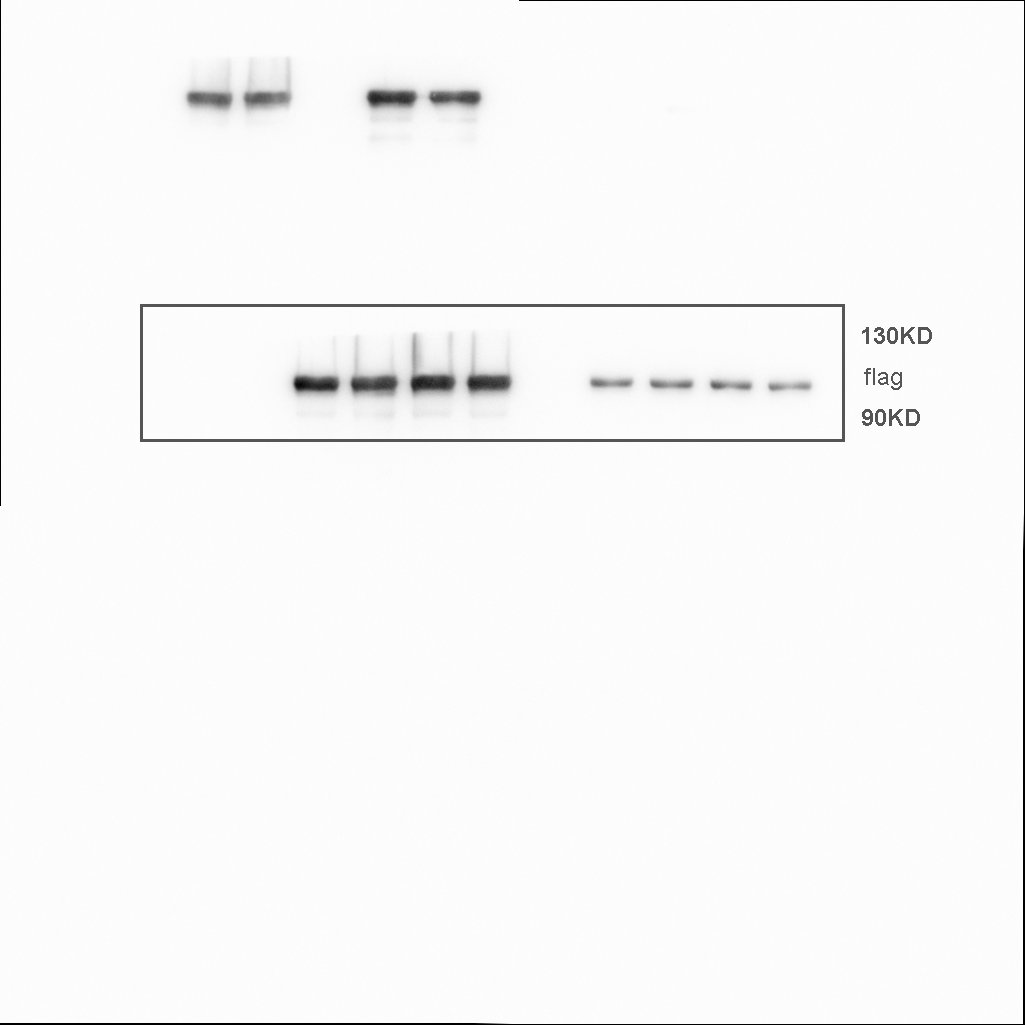

Supplement: Supplementary file 8 — Source Data Fig. 5 [file 44318_2023_3_MOESM8_ESM.zip › Figure5/5c/flag down DC1-IP V DC1-WT mut WT-RNase A WT-MBN (VP16 IP input).jpg]

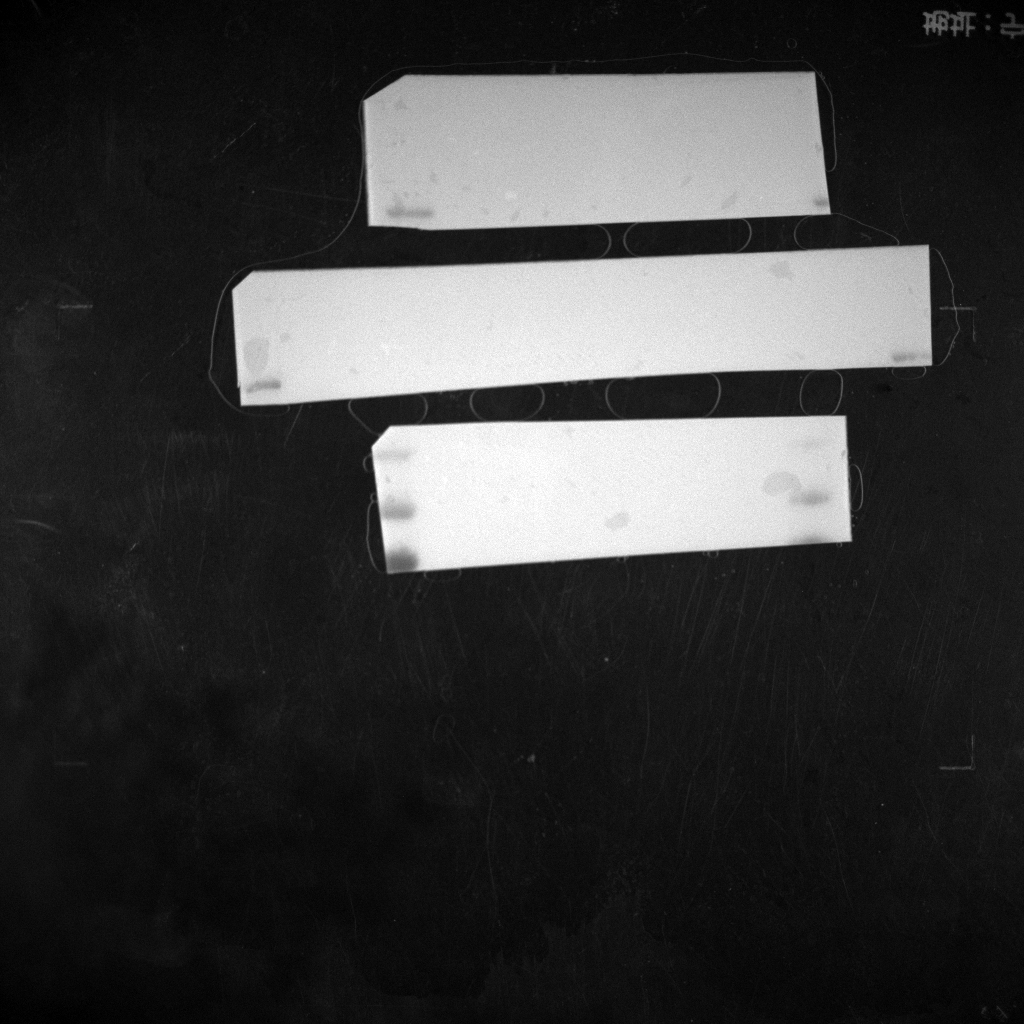

Supplement: Supplementary file 8 — Source Data Fig. 5 [file 44318_2023_3_MOESM8_ESM.zip › Figure5/5c/Topbp1 middle DC1-IP V DC1-WT mut WT-RNase A WT-MBN (VP16 IP input) W .tif.tif]

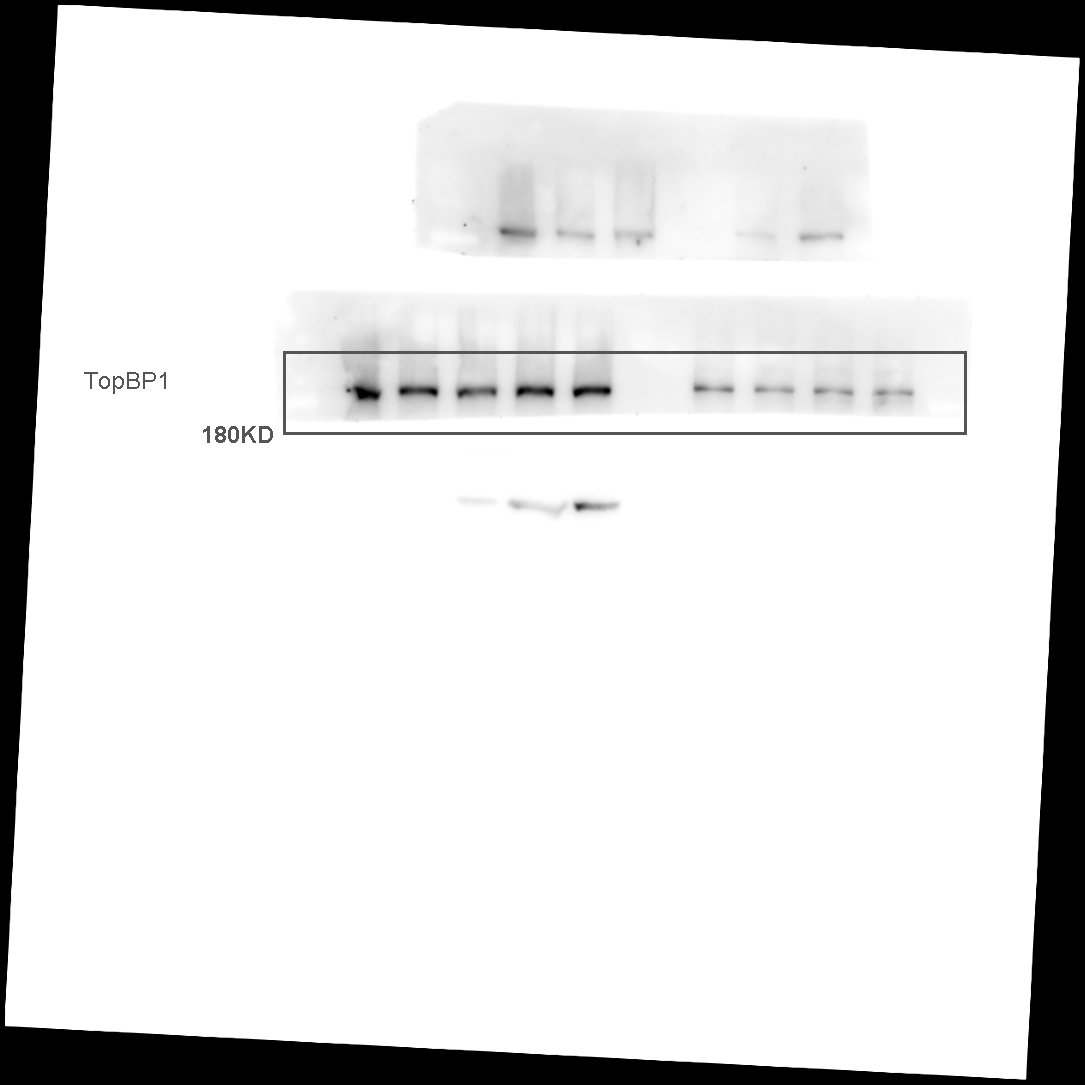

Supplement: Supplementary file 8 — Source Data Fig. 5 [file 44318_2023_3_MOESM8_ESM.zip › Figure5/5c/Topbp1 middle DC1-IP V DC1-WT mut WT-RNase A WT-MBN (VP16 IP input).jpg]

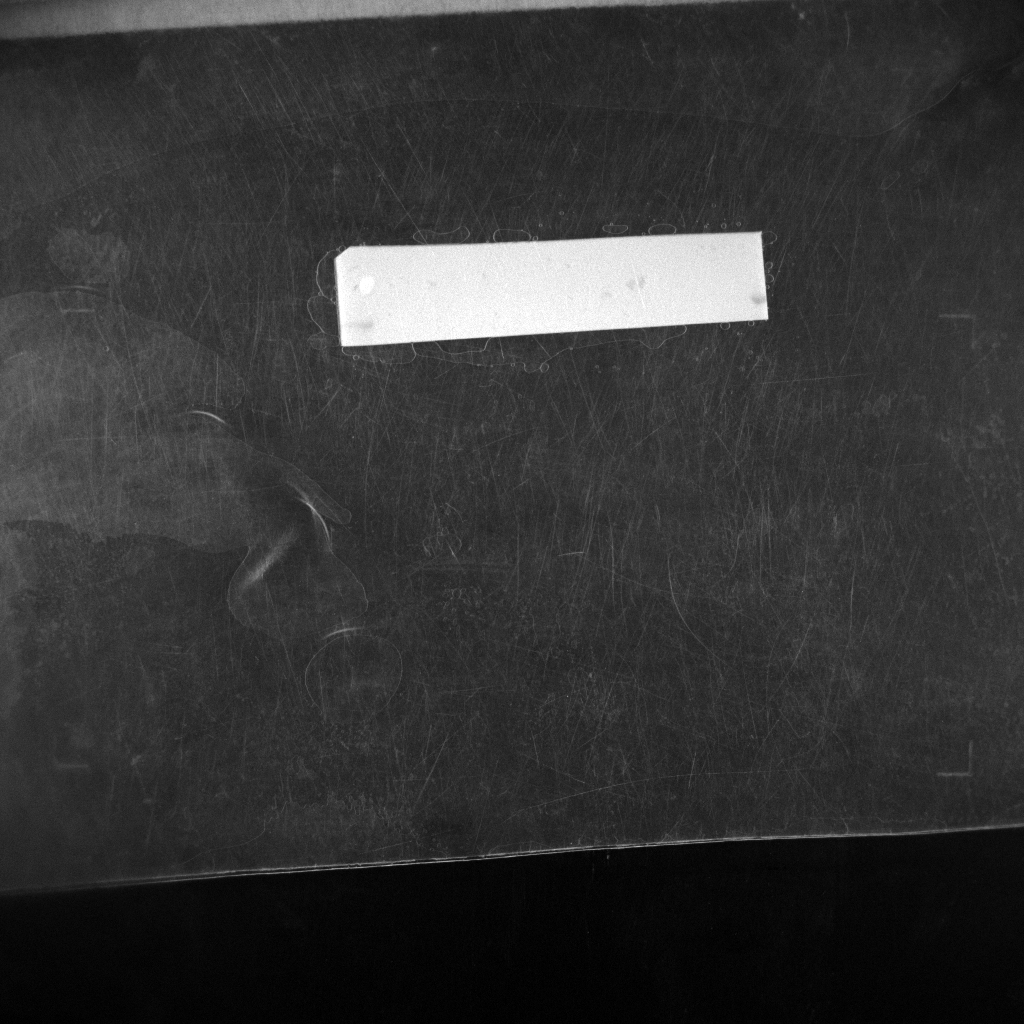

Supplement: Supplementary file 8 — Source Data Fig. 5 [file 44318_2023_3_MOESM8_ESM.zip › Figure5/5d/20210709 Topbp1 mcherry mcherry-DC1-WT mcherry-DC1-mut (input IP) W.tif]

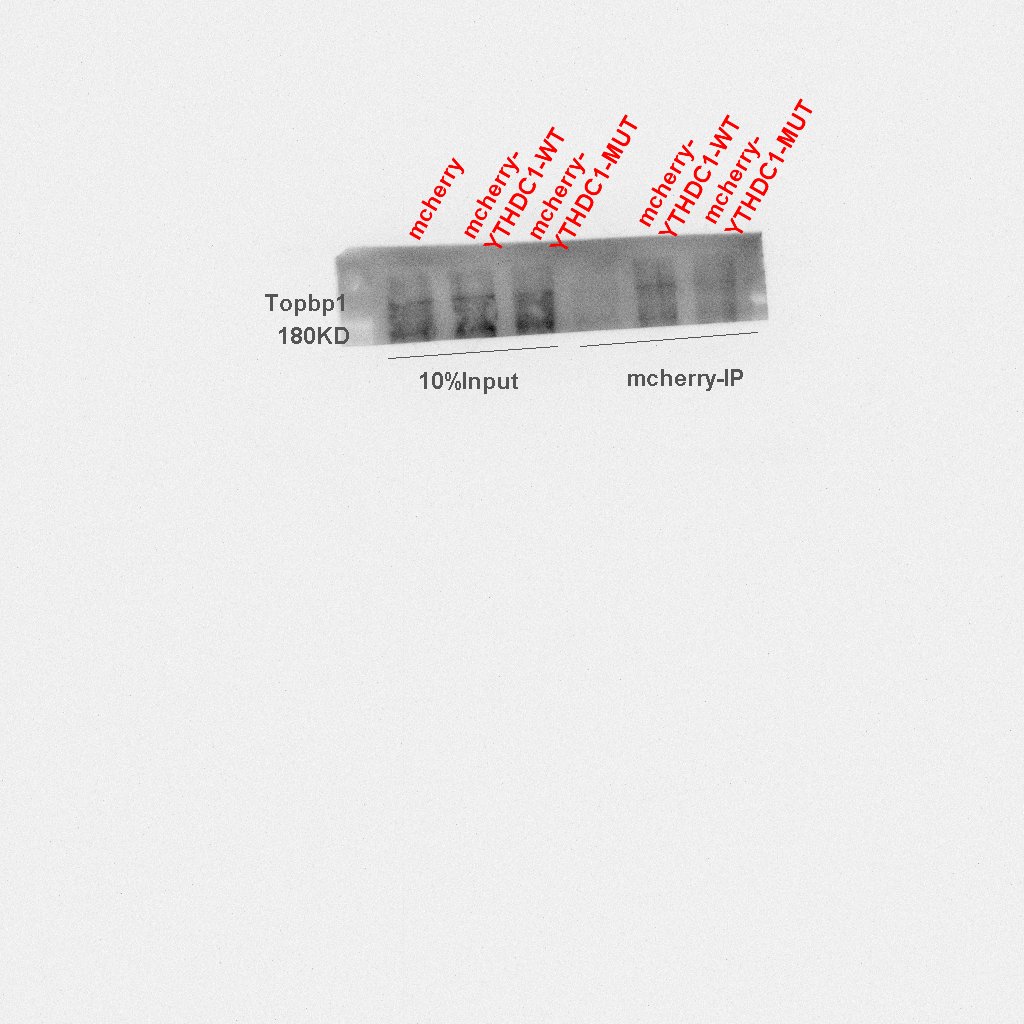

Supplement: Supplementary file 8 — Source Data Fig. 5 [file 44318_2023_3_MOESM8_ESM.zip › Figure5/5d/20210709 Topbp1 mcherry mcherry-DC1-WT mcherry-DC1-mut (input IP).jpg]

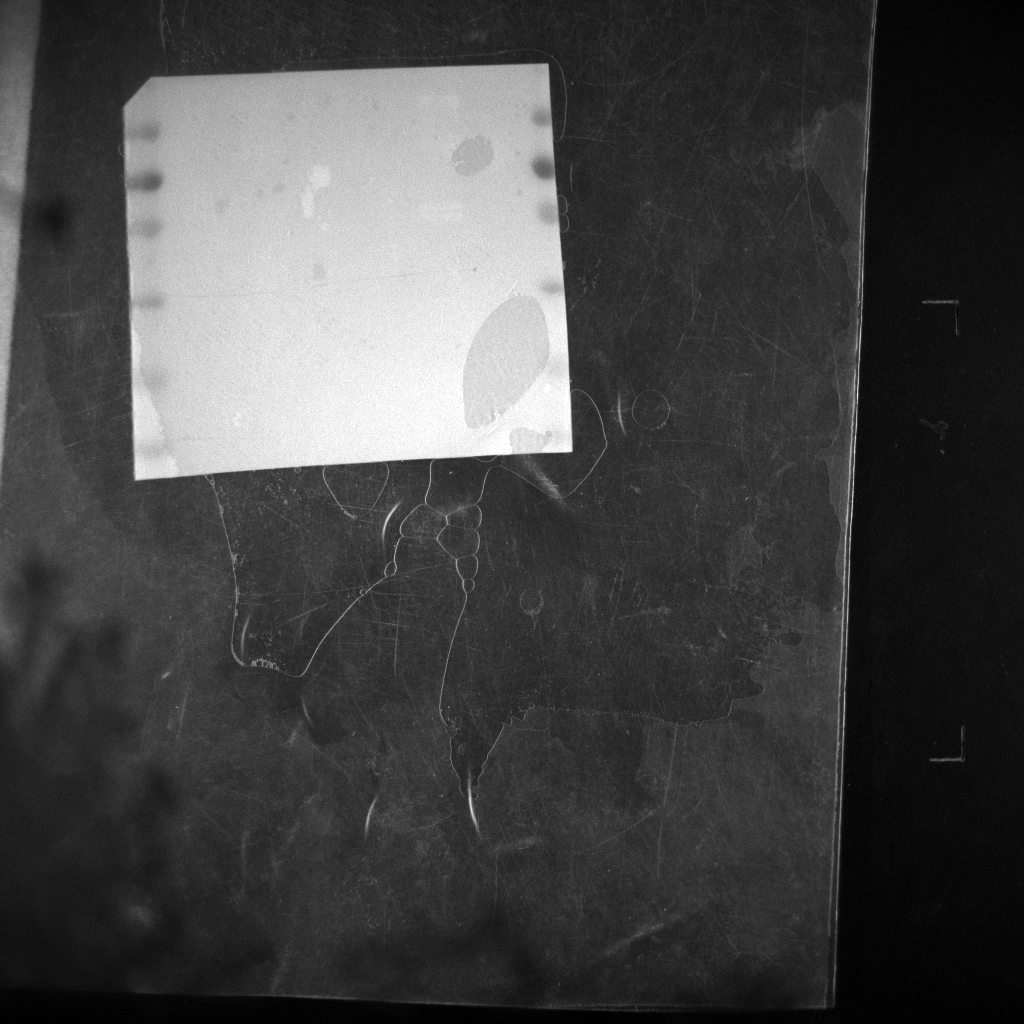

Supplement: Supplementary file 8 — Source Data Fig. 5 [file 44318_2023_3_MOESM8_ESM.zip › Figure5/5d/20210709 YTHDC1 mcherry mcherry-DC1-WT mcherry-DC1-mut (input IP) W.tif]

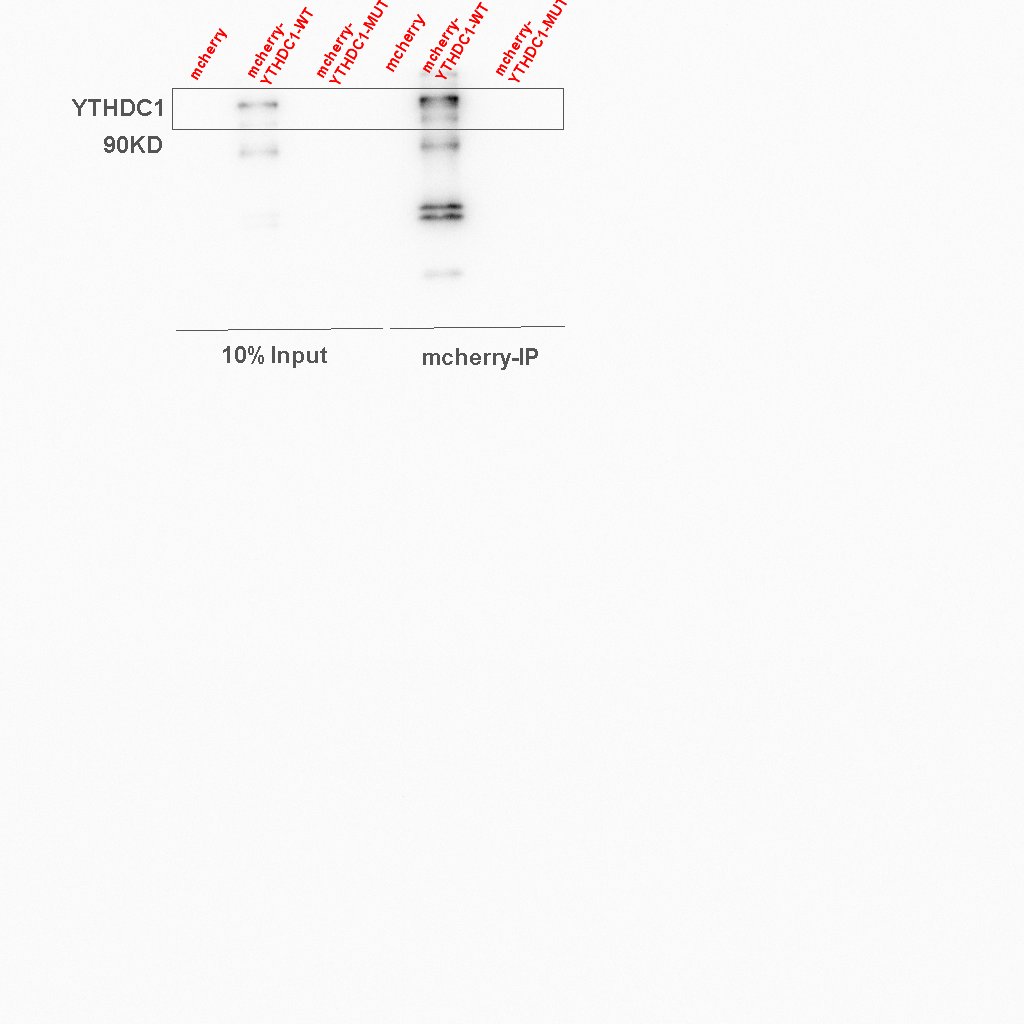

Supplement: Supplementary file 8 — Source Data Fig. 5 [file 44318_2023_3_MOESM8_ESM.zip › Figure5/5d/20210709 YTHDC1 mcherry mcherry-DC1-WT mcherry-DC1-mut (input IP).jpg]

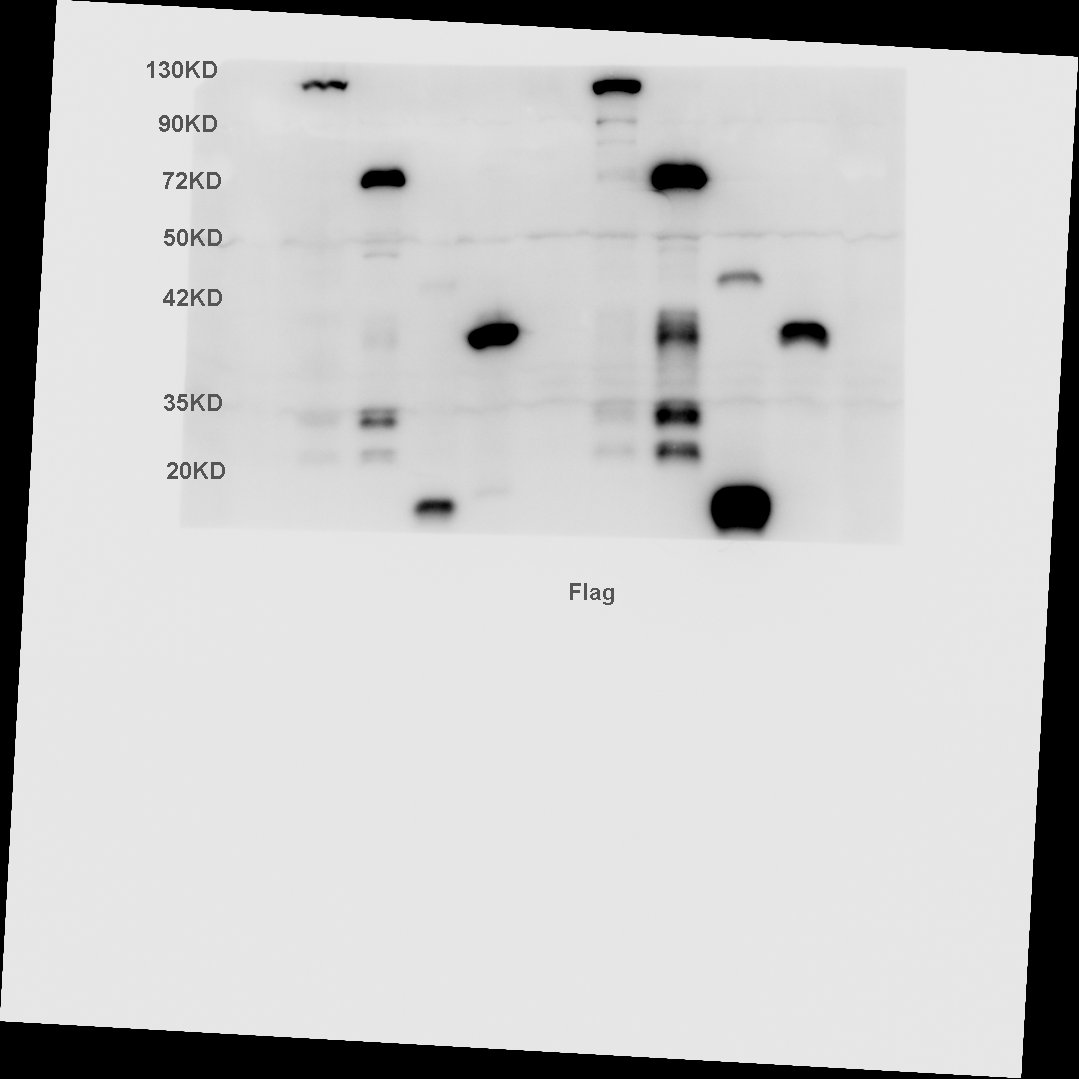

Supplement: Supplementary file 8 — Source Data Fig. 5 [file 44318_2023_3_MOESM8_ESM.zip › Figure5/5e/Flag v FL N YTH C (10%Input IP) .jpg]

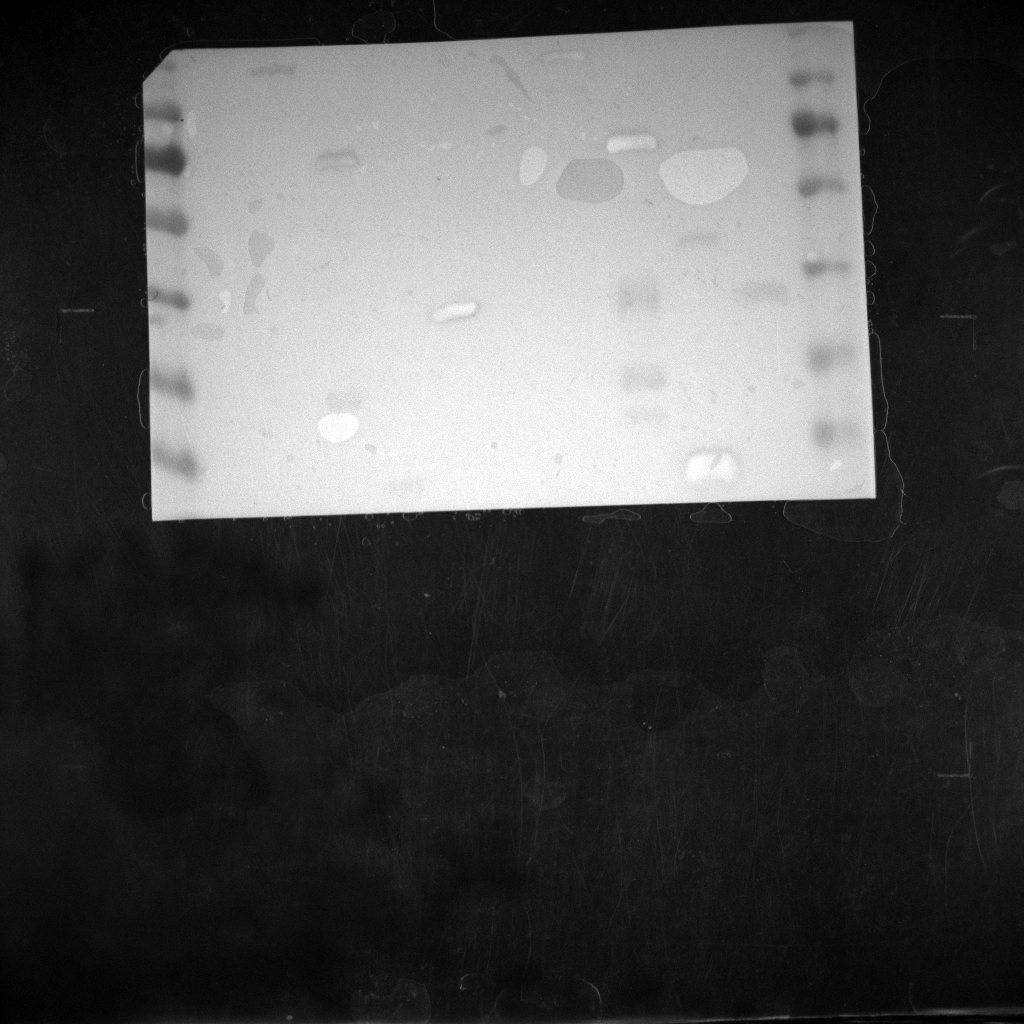

Supplement: Supplementary file 8 — Source Data Fig. 5 [file 44318_2023_3_MOESM8_ESM.zip › Figure5/5e/Flag v FL N YTH C (10%Input IP) w .tif]

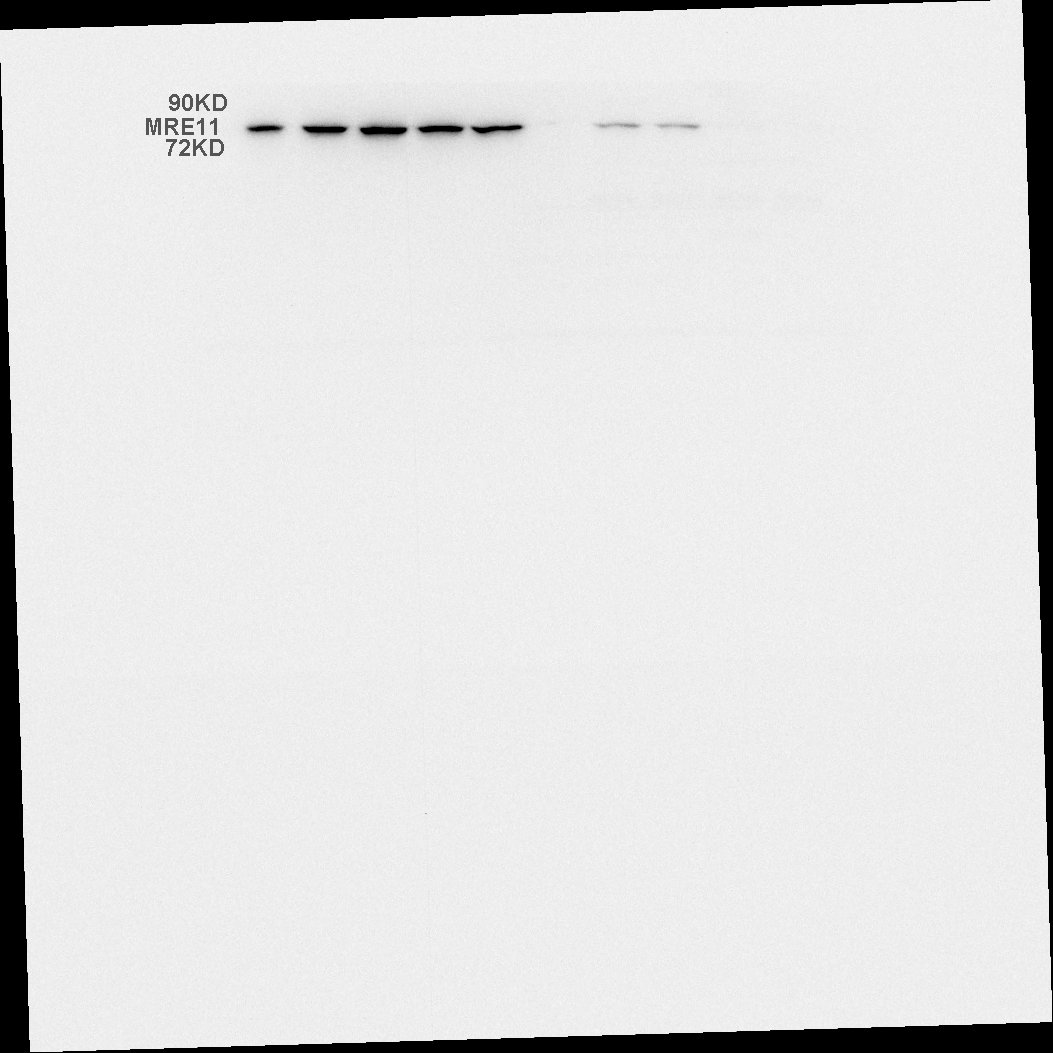

Supplement: Supplementary file 8 — Source Data Fig. 5 [file 44318_2023_3_MOESM8_ESM.zip › Figure5/5e/MRE11 v FL N YTH C (10%Input IP) .jpg]

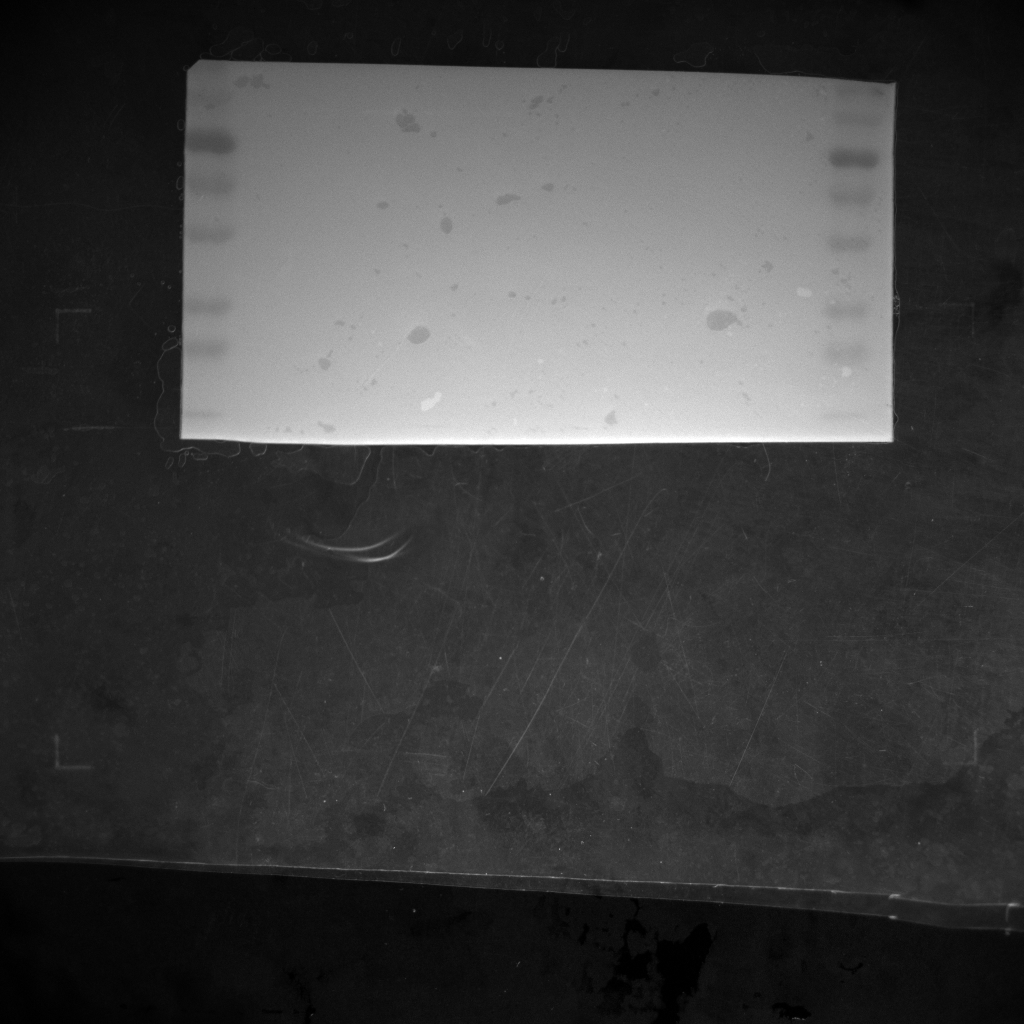

Supplement: Supplementary file 8 — Source Data Fig. 5 [file 44318_2023_3_MOESM8_ESM.zip › Figure5/5e/MRE11 v FL N YTH C (10%Input IP) w .tif]

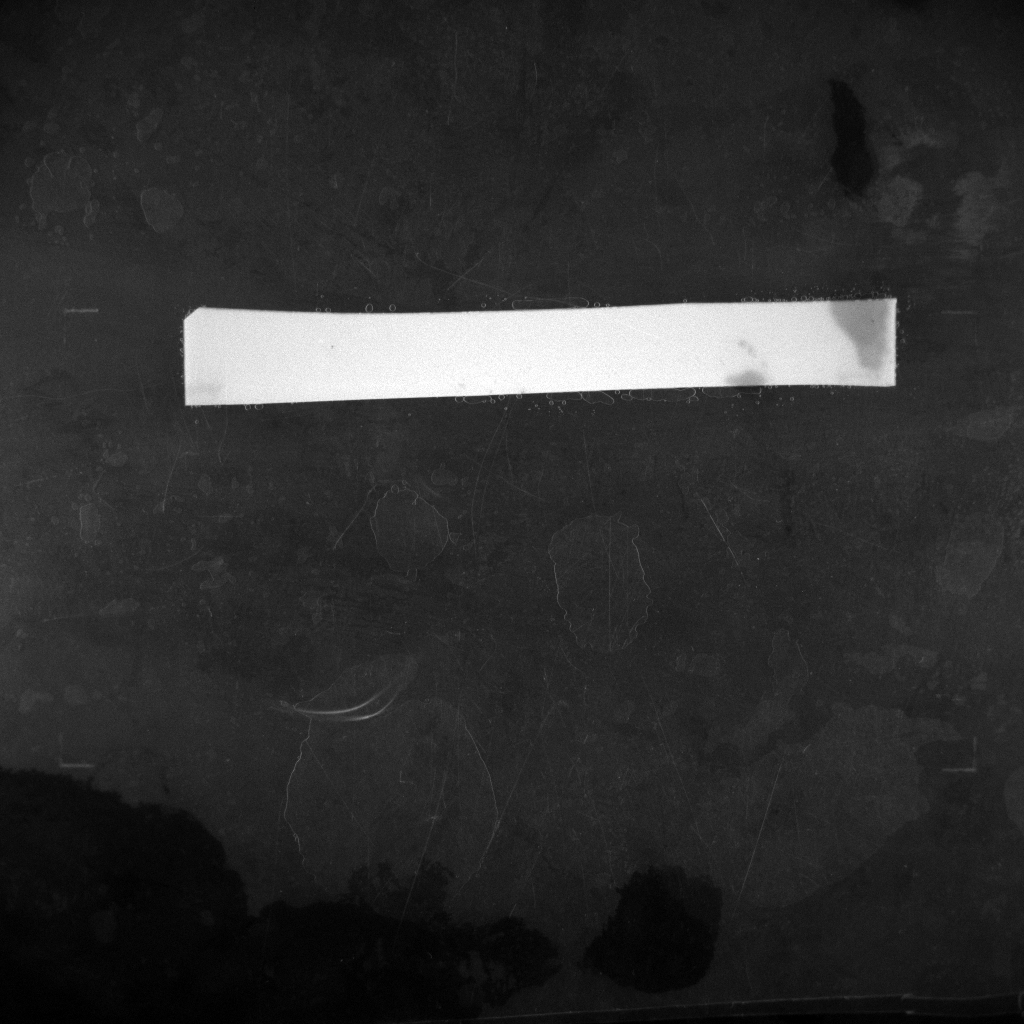

Supplement: Supplementary file 8 — Source Data Fig. 5 [file 44318_2023_3_MOESM8_ESM.zip › Figure5/5e/ToPBP1 v FL N YTH C (10%Input IP) w.tif]

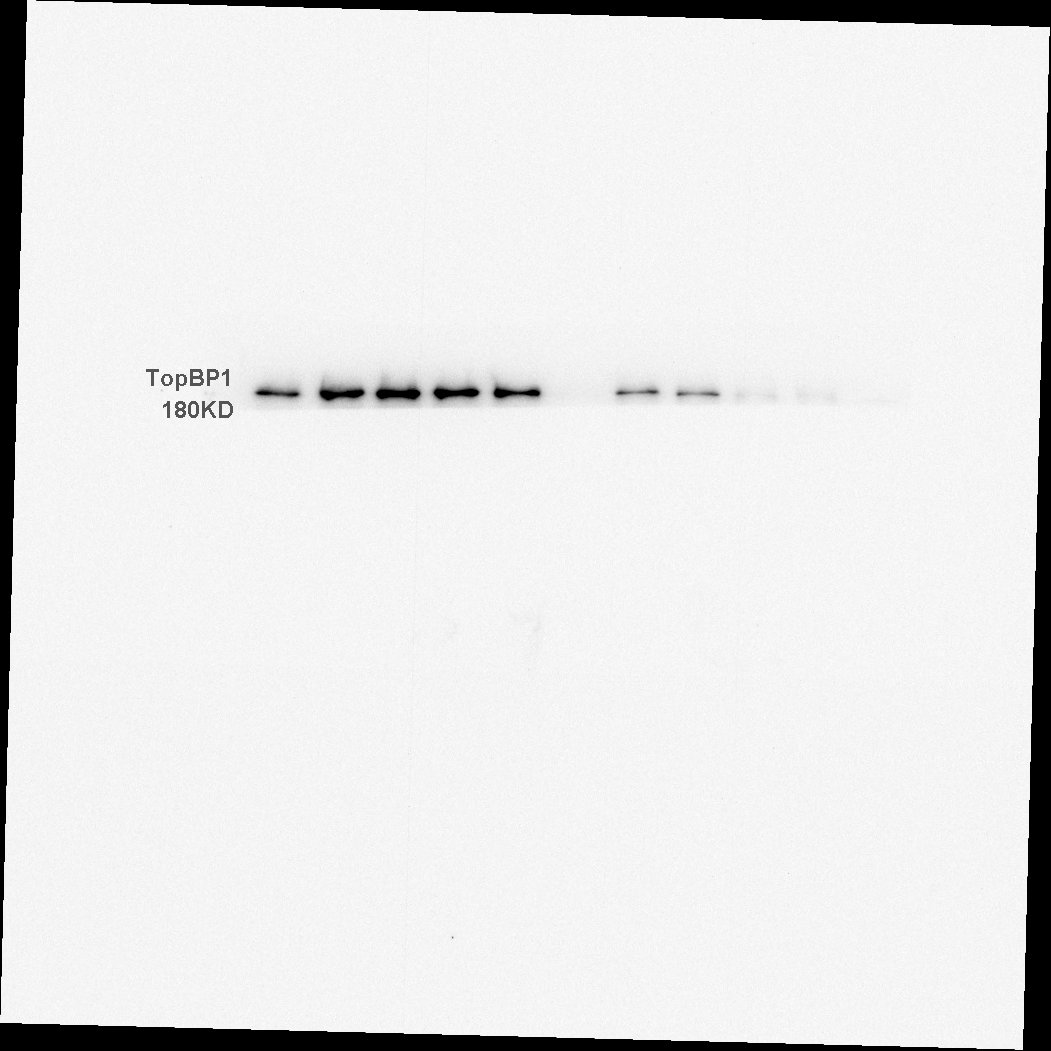

Supplement: Supplementary file 8 — Source Data Fig. 5 [file 44318_2023_3_MOESM8_ESM.zip › Figure5/5e/ToPBP1 v FL N YTH C (10%Input IP).jpg]

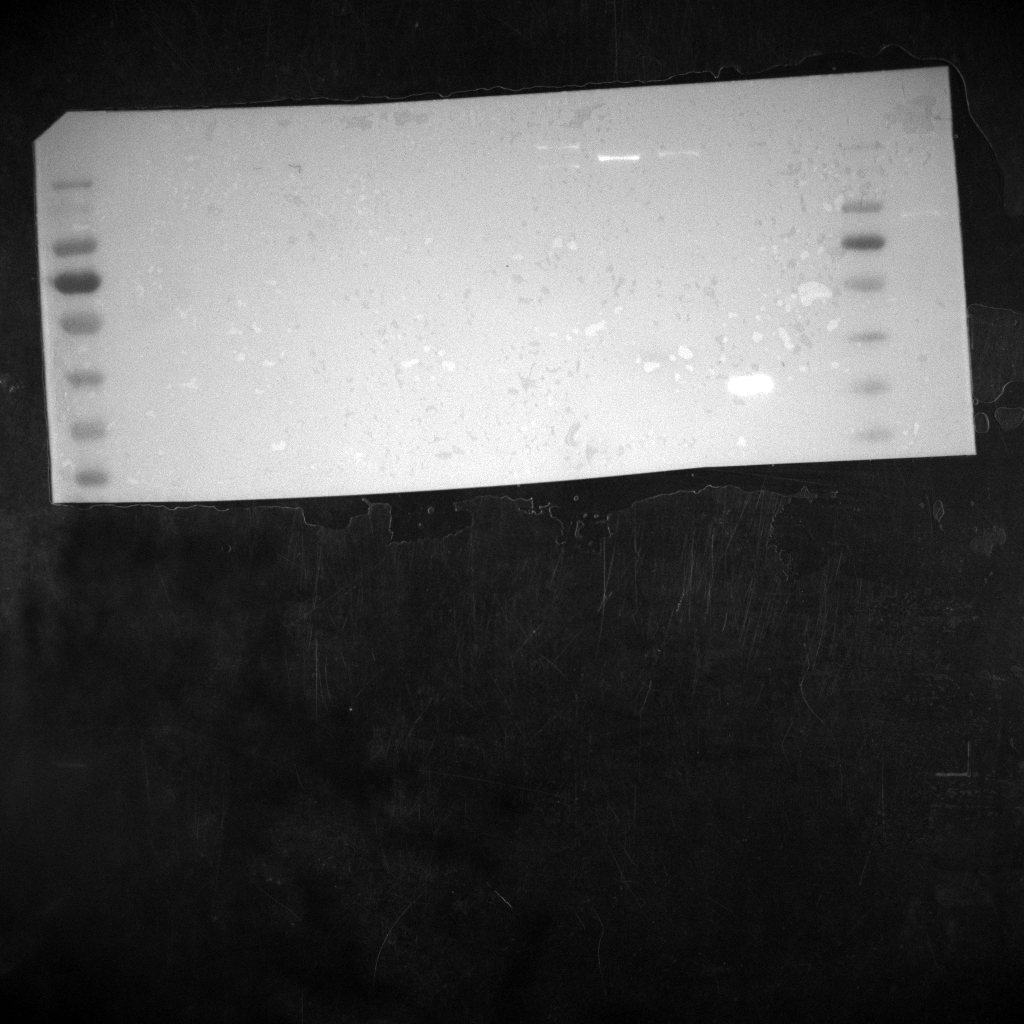

Supplement: Supplementary file 8 — Source Data Fig. 5 [file 44318_2023_3_MOESM8_ESM.zip › Figure5/5f/Flag V FL de1-2 de7-8 1-2 1-3 (input IP) W .tif]

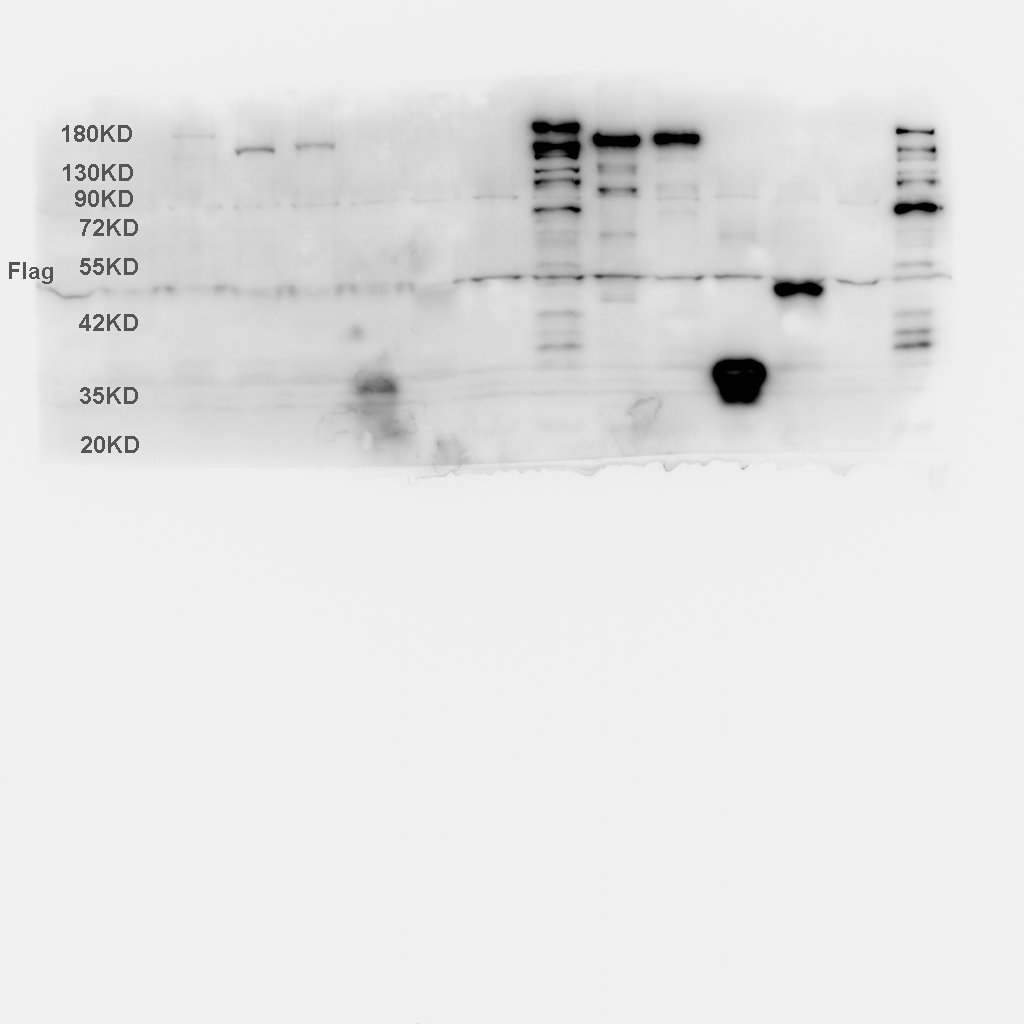

Supplement: Supplementary file 8 — Source Data Fig. 5 [file 44318_2023_3_MOESM8_ESM.zip › Figure5/5f/Flag V FL de1-2 de7-8 1-2 1-3 (input IP).jpg]

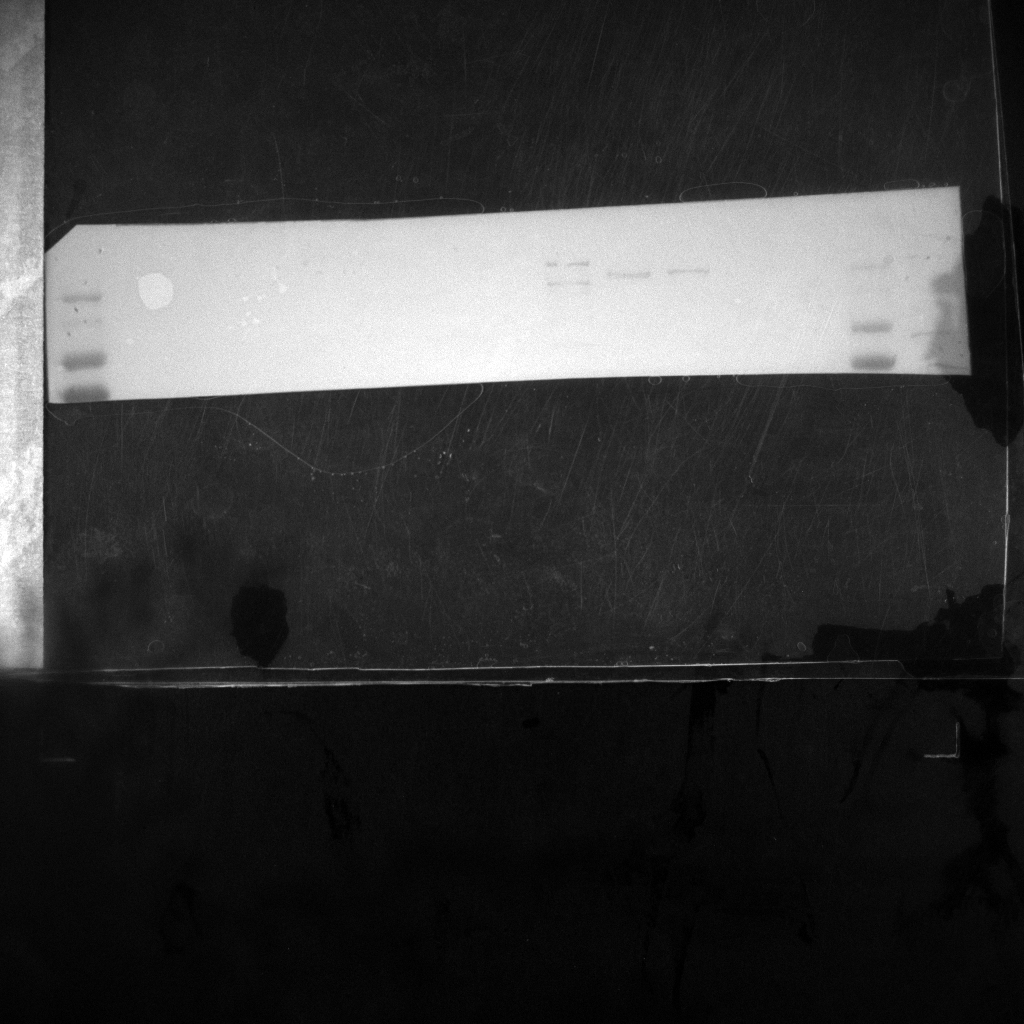

Supplement: Supplementary file 8 — Source Data Fig. 5 [file 44318_2023_3_MOESM8_ESM.zip › Figure5/5f/YTHDC1 V FL de1-2 de7-8 1-2 1-3 (input IP) W.tif]

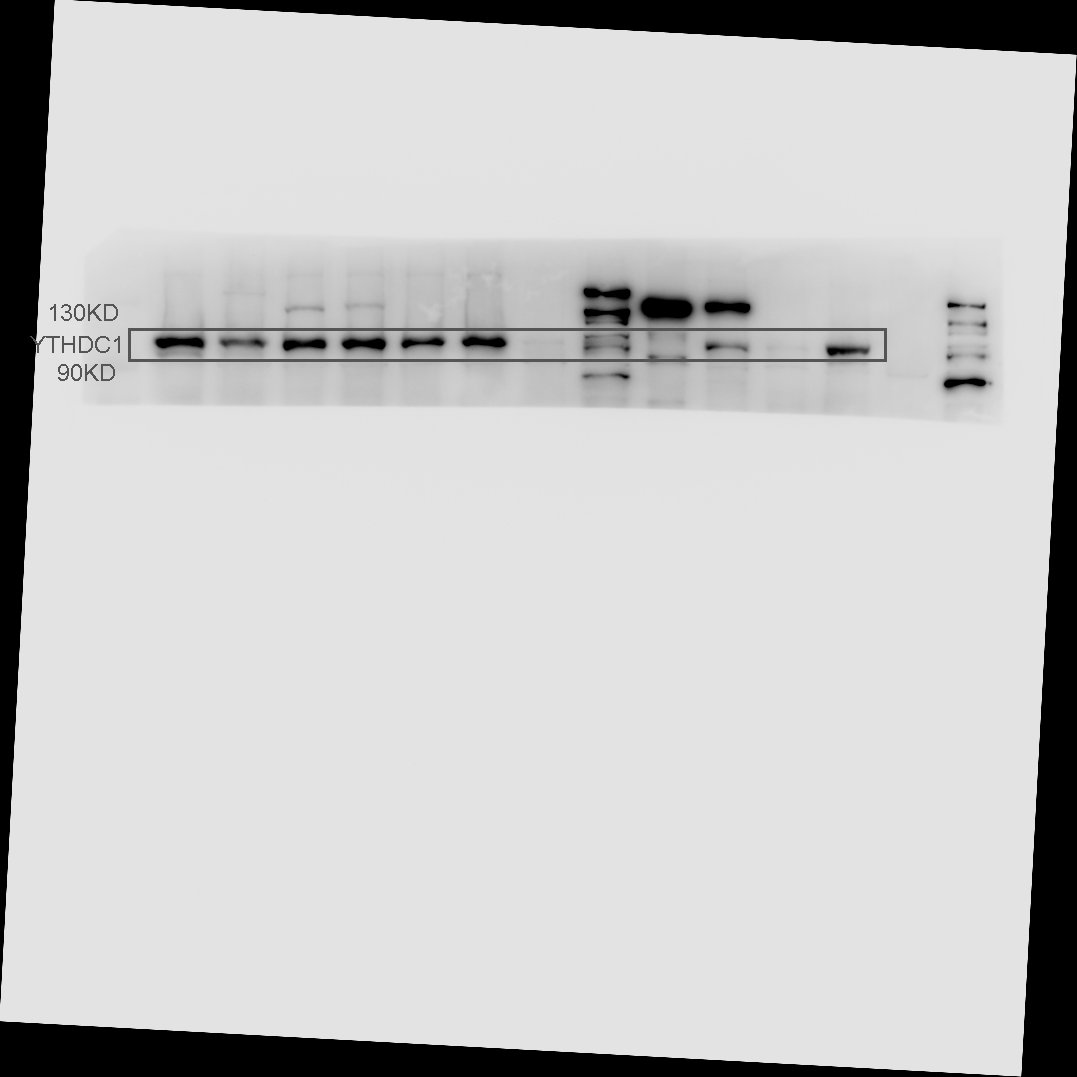

Supplement: Supplementary file 8 — Source Data Fig. 5 [file 44318_2023_3_MOESM8_ESM.zip › Figure5/5f/YTHDC1 V FL de1-2 de7-8 1-2 1-3 (input IP).jpg]

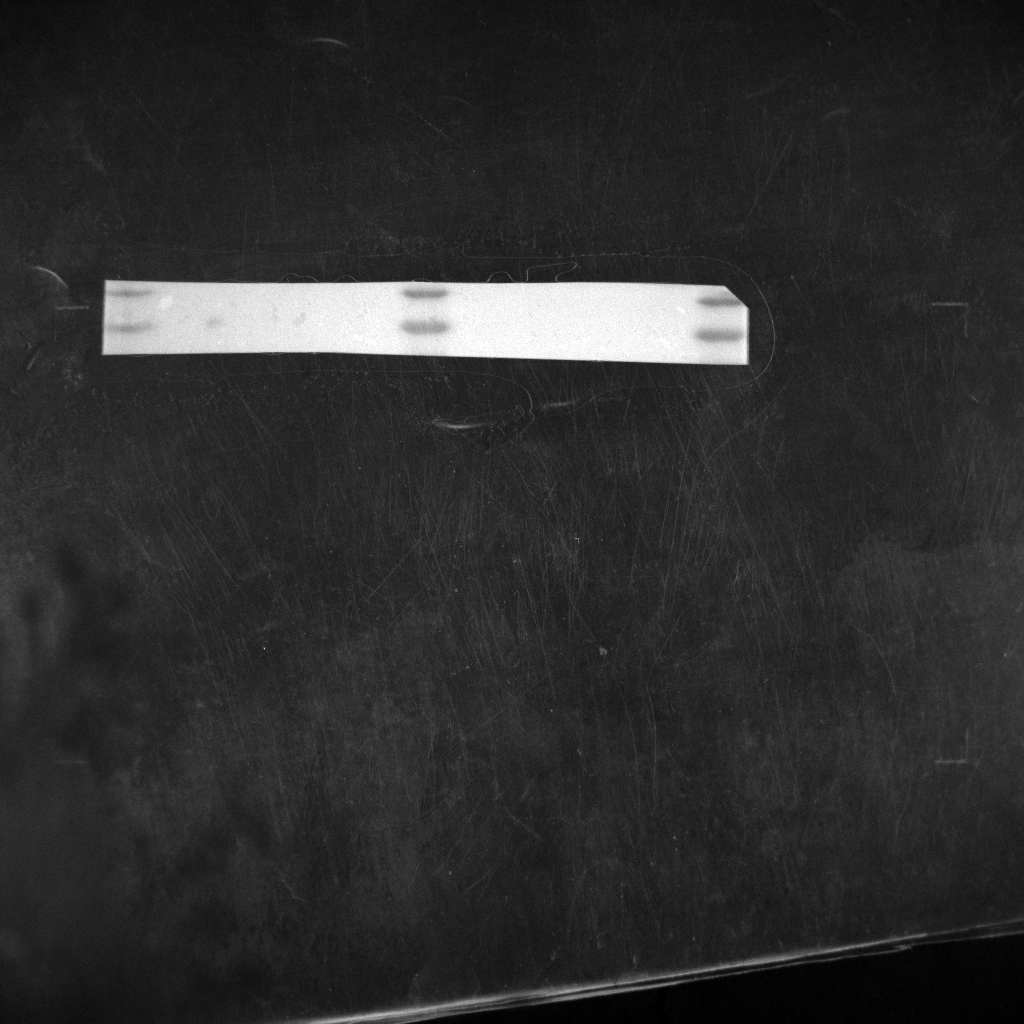

Supplement: Supplementary file 9 — Source Data Fig. 6 [file 44318_2023_3_MOESM9_ESM.zip › Figure6/6a/GAPDH left DMSO NC siDC1-1 -2-VP16 W .tif]

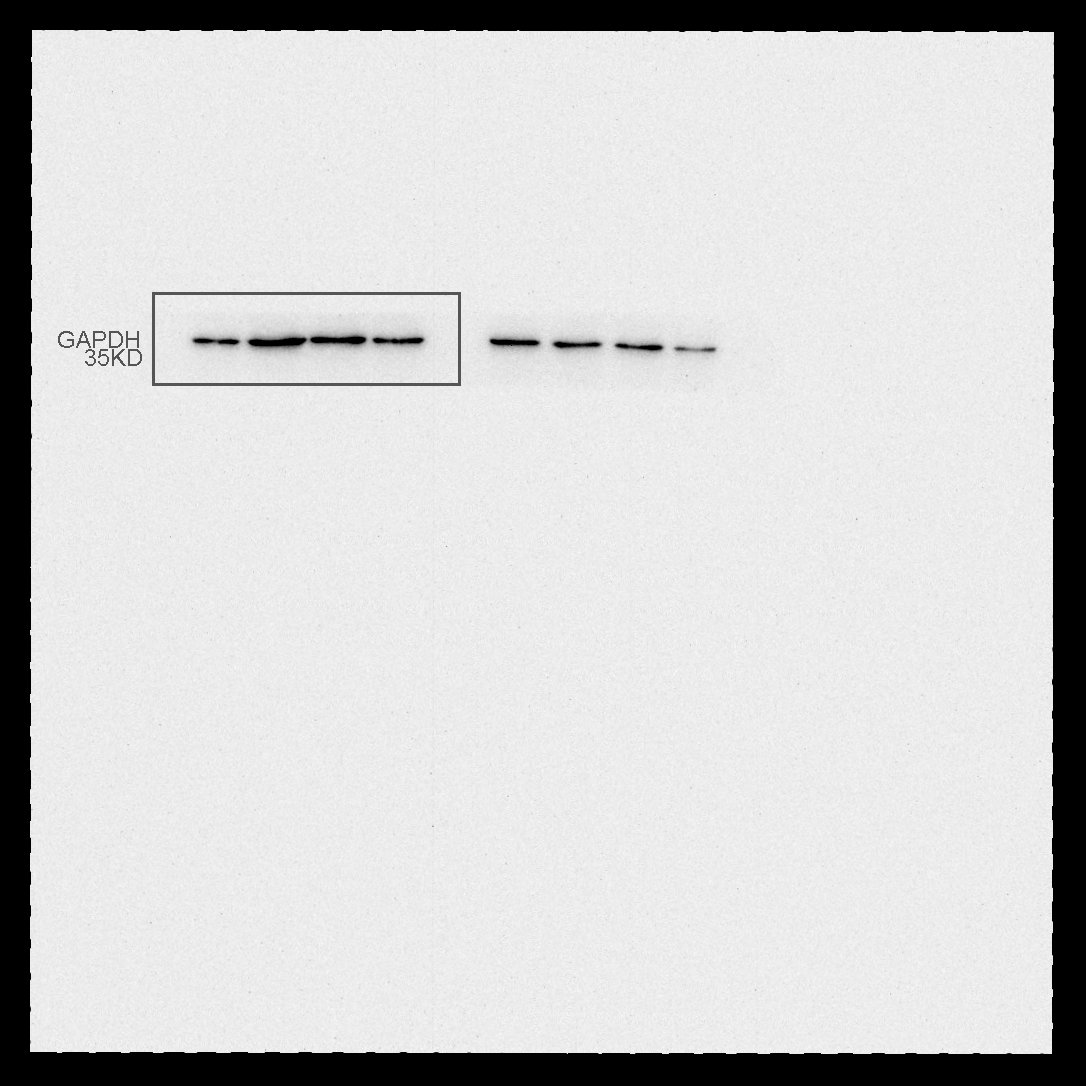

Supplement: Supplementary file 9 — Source Data Fig. 6 [file 44318_2023_3_MOESM9_ESM.zip › Figure6/6a/GAPDH left DMSO NC siDC1-1 -2-VP16 .jpg]

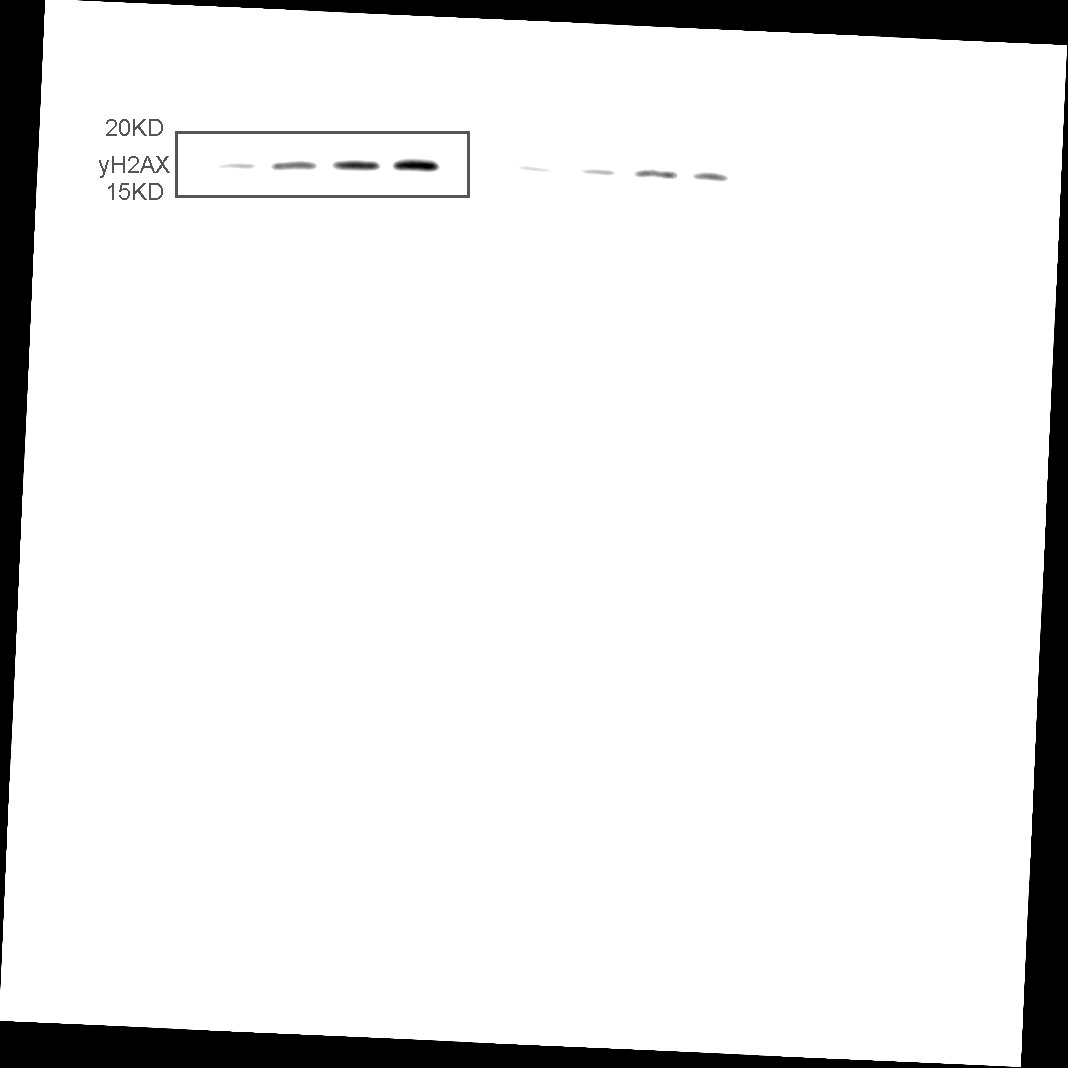

Supplement: Supplementary file 9 — Source Data Fig. 6 [file 44318_2023_3_MOESM9_ESM.zip › Figure6/6a/yh2ax left DMSO NC siDC1-1 -2-VP16 .jpg]

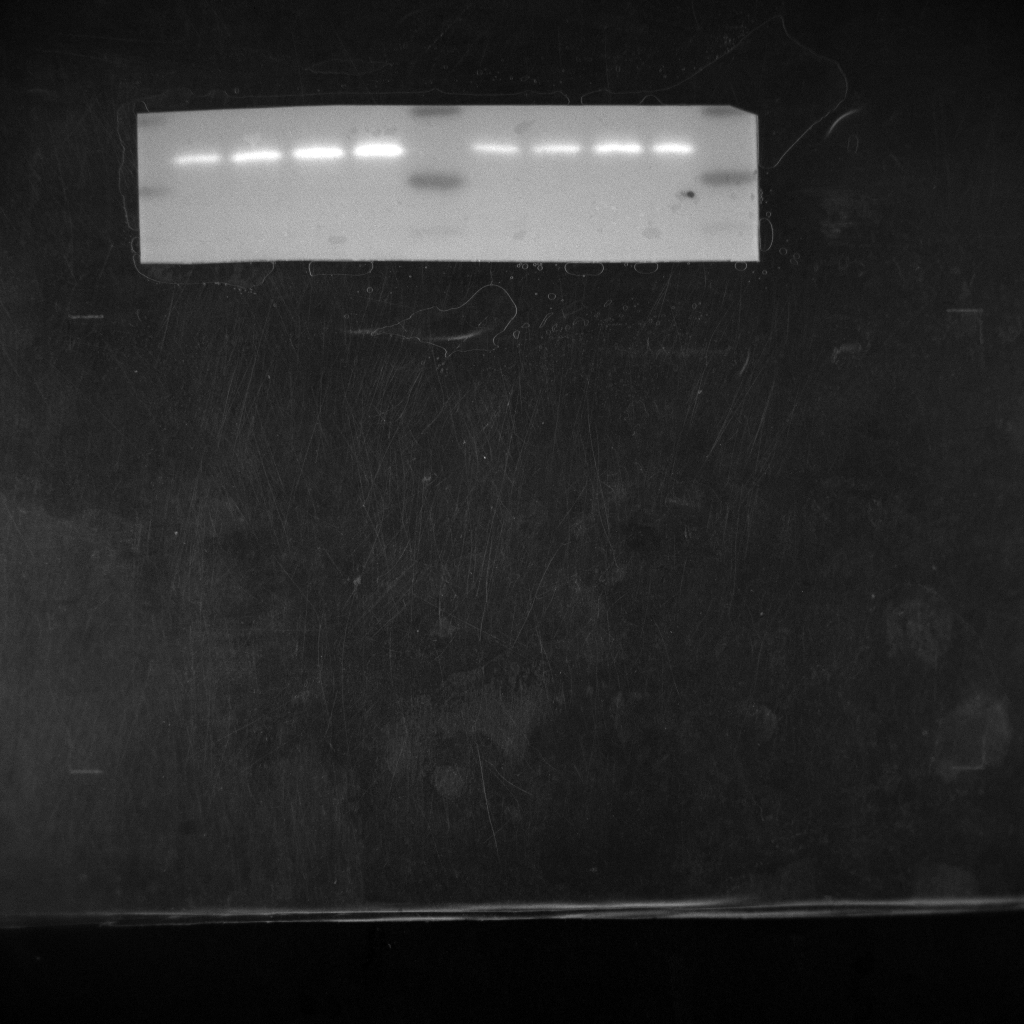

Supplement: Supplementary file 9 — Source Data Fig. 6 [file 44318_2023_3_MOESM9_ESM.zip › Figure6/6a/yh2ax left DMSO NC siDC1-1 -2-VP16 w .tif]

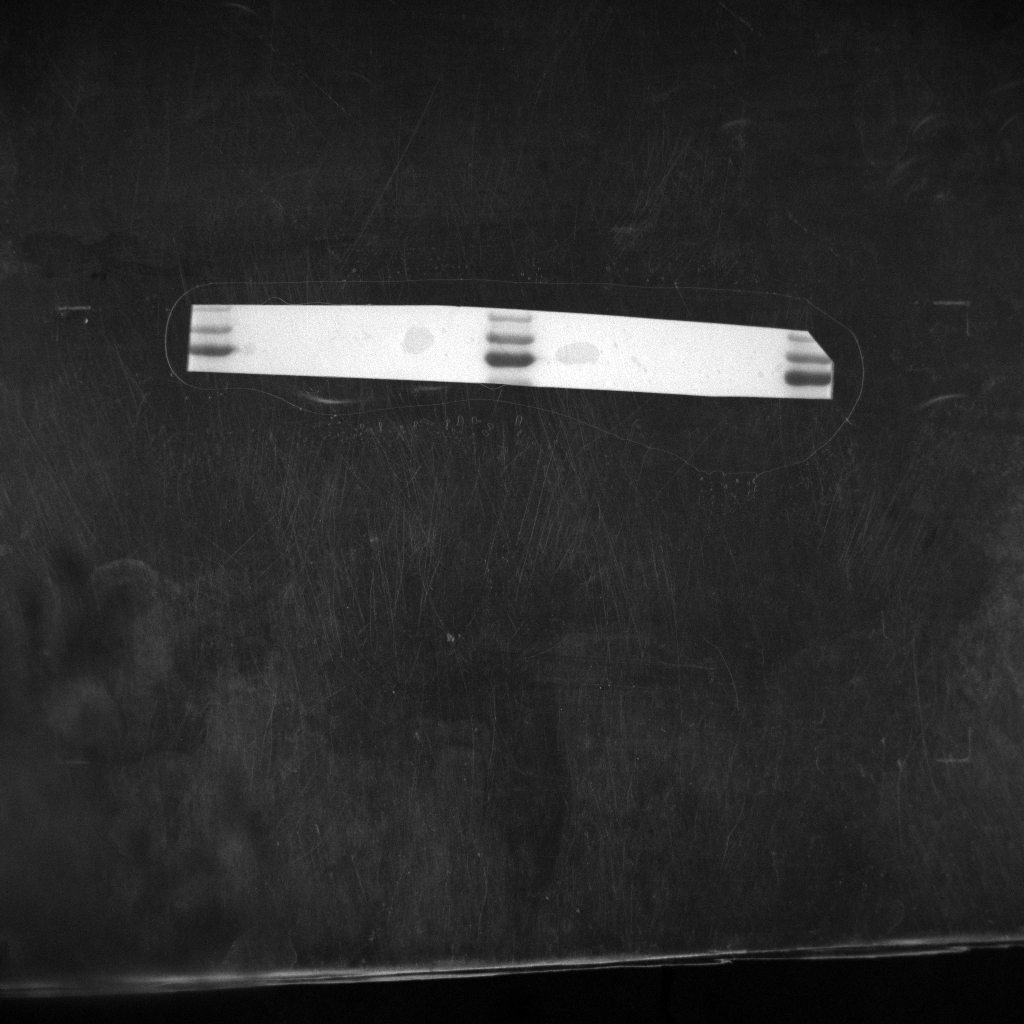

Supplement: Supplementary file 9 — Source Data Fig. 6 [file 44318_2023_3_MOESM9_ESM.zip › Figure6/6a/YTHDC1 left DMSO NC siDC1-1 -2-VP16 w .tif]

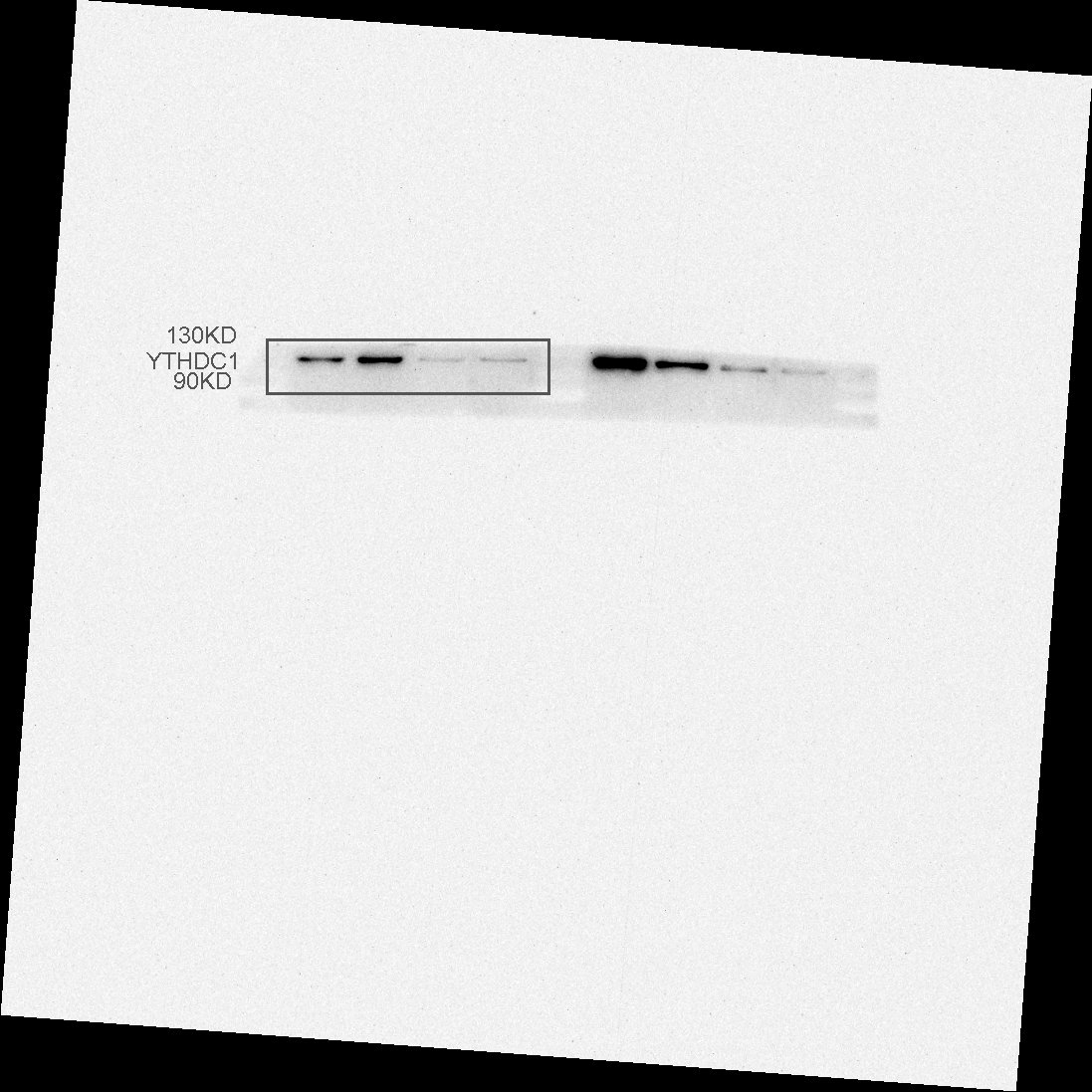

Supplement: Supplementary file 9 — Source Data Fig. 6 [file 44318_2023_3_MOESM9_ESM.zip › Figure6/6a/YTHDC1 left DMSO NC siDC1-1 -2-VP16 .jpg]

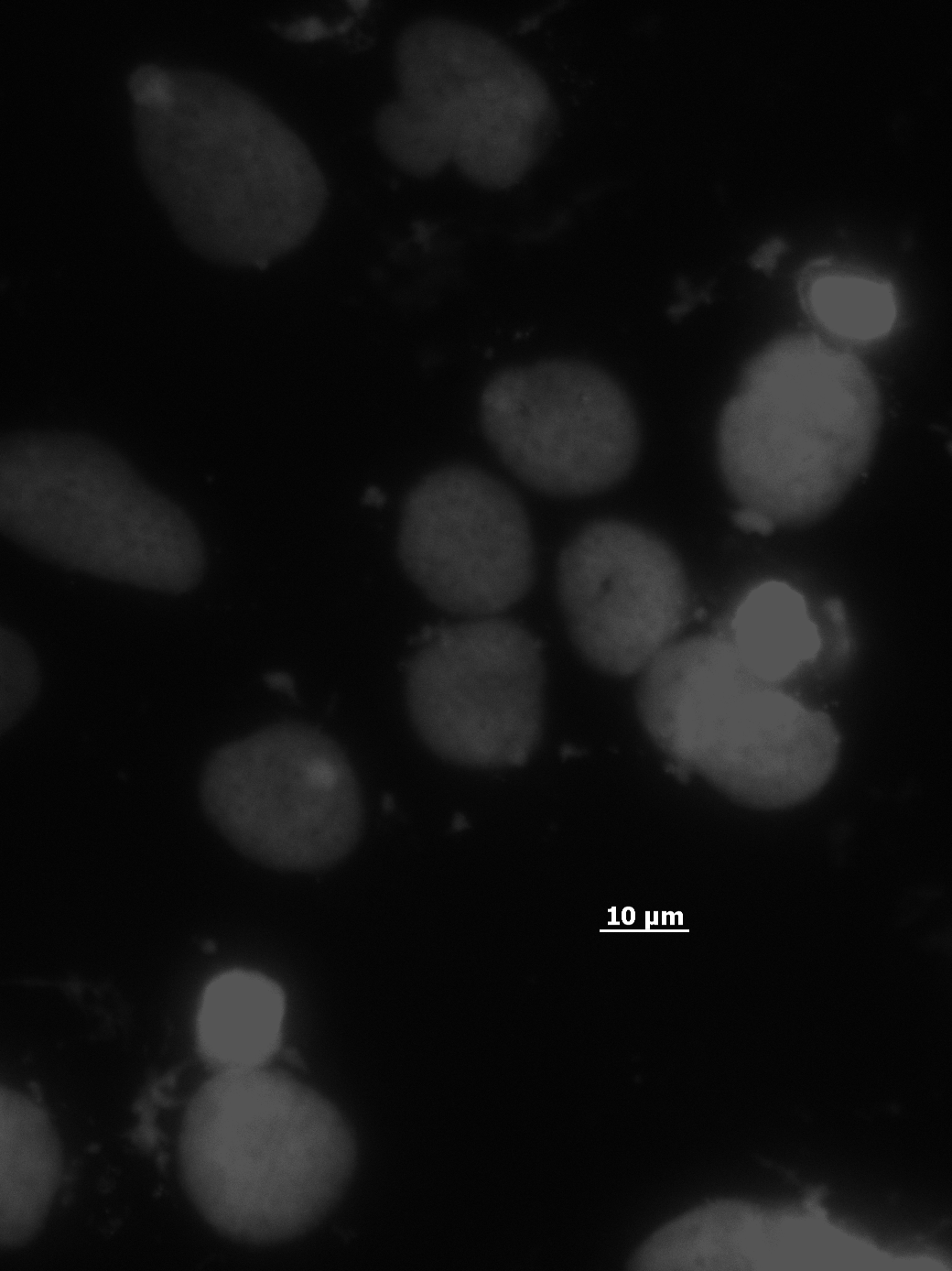

Supplement: Supplementary file 9 — Source Data Fig. 6 [file 44318_2023_3_MOESM9_ESM.zip › Figure6/6c-d/siYTHDC1 micronuclei/siYTHDC1 micronuclei.TIF]

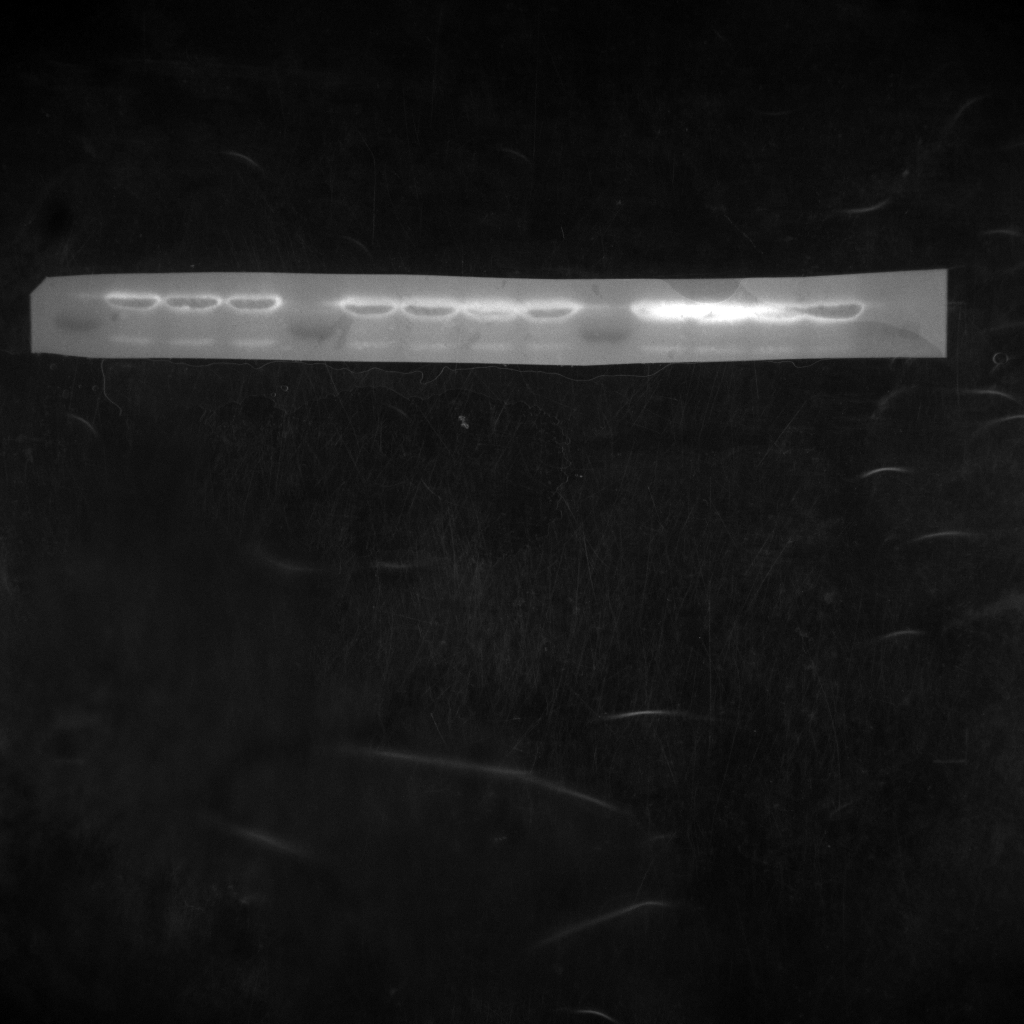

Supplement: Supplementary file 9 — Source Data Fig. 6 [file 44318_2023_3_MOESM9_ESM.zip › Figure6/6e/GAPDH L2-NC shDC1-1-Vector shDC1-1-N W.tif]

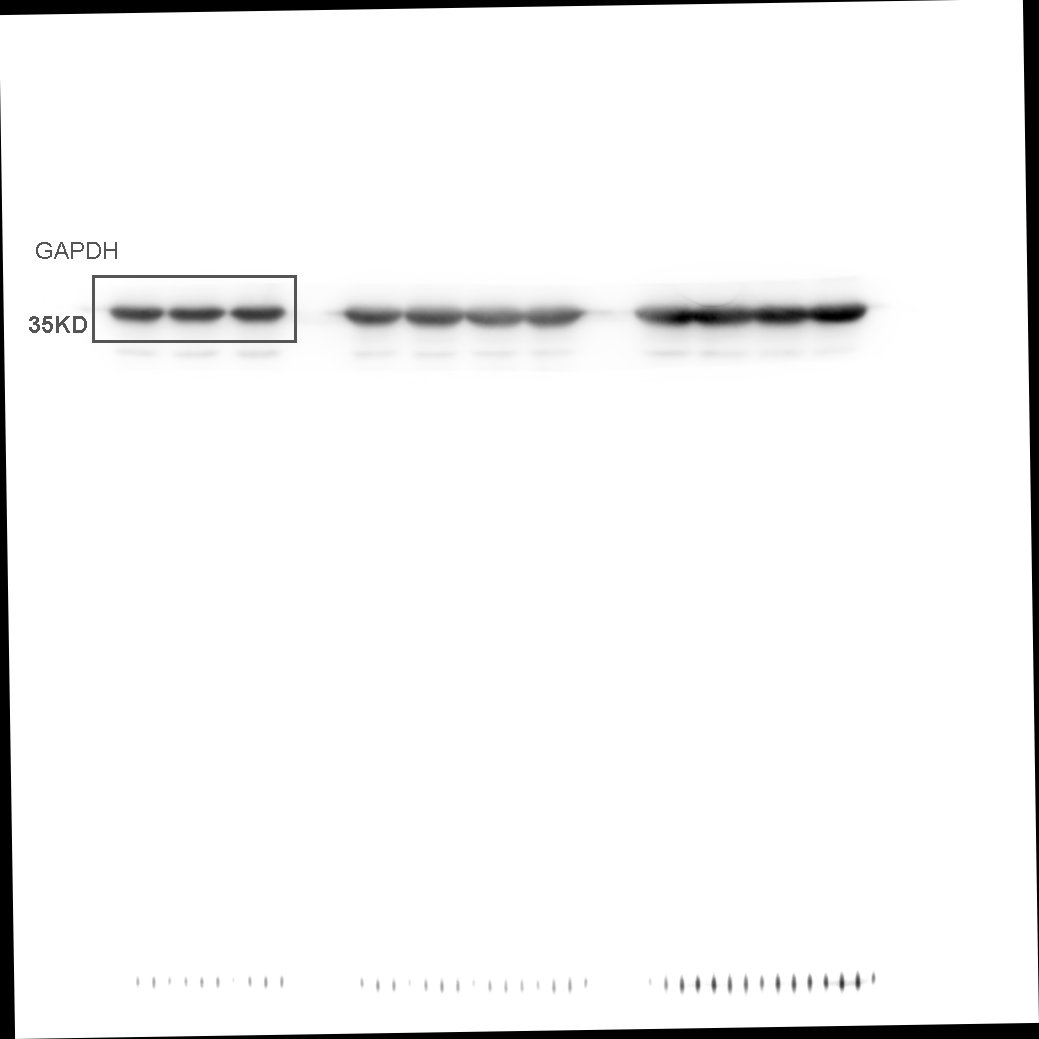

Supplement: Supplementary file 9 — Source Data Fig. 6 [file 44318_2023_3_MOESM9_ESM.zip › Figure6/6e/GAPDH L2-NC shDC1-1-Vector shDC1-1-N.jpg]

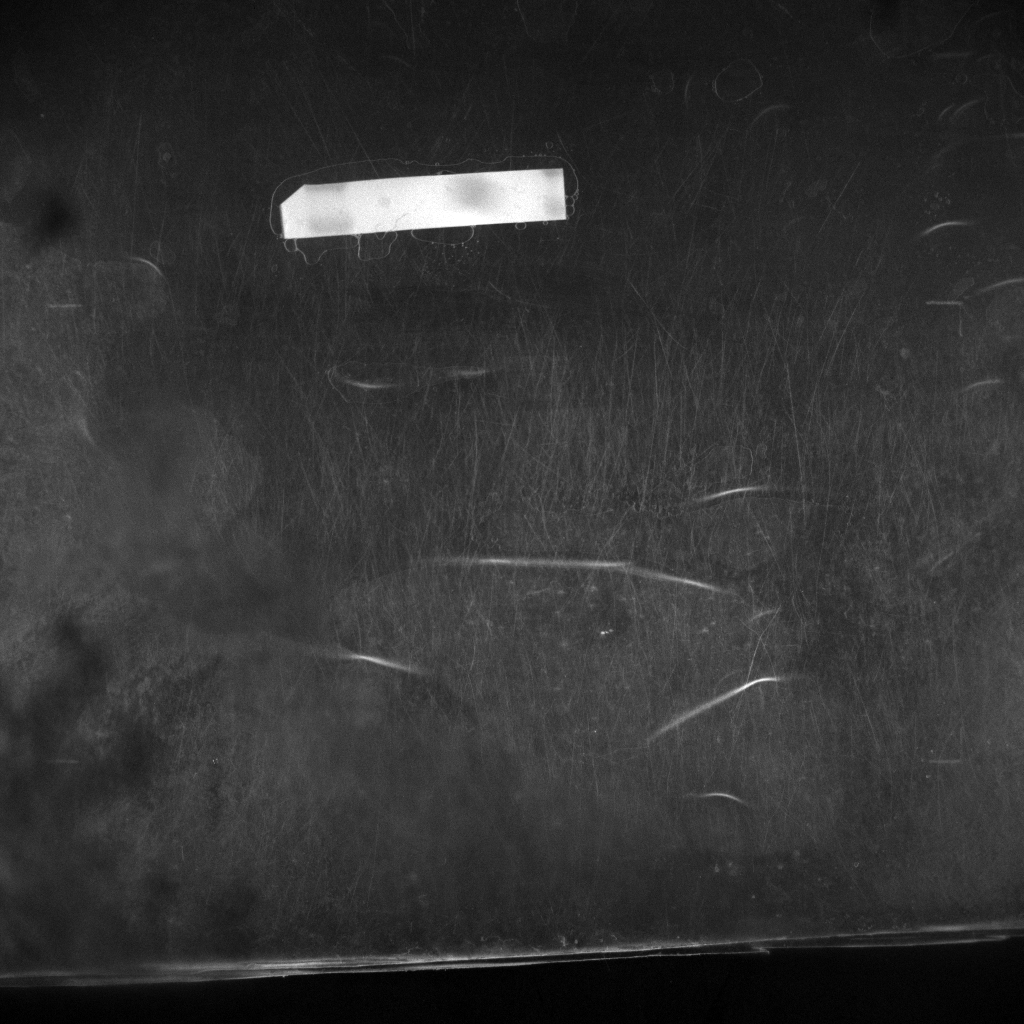

Supplement: Supplementary file 9 — Source Data Fig. 6 [file 44318_2023_3_MOESM9_ESM.zip › Figure6/6e/GFP L2-NC shDC1-1-Vector shDC1-1-N w.tif]

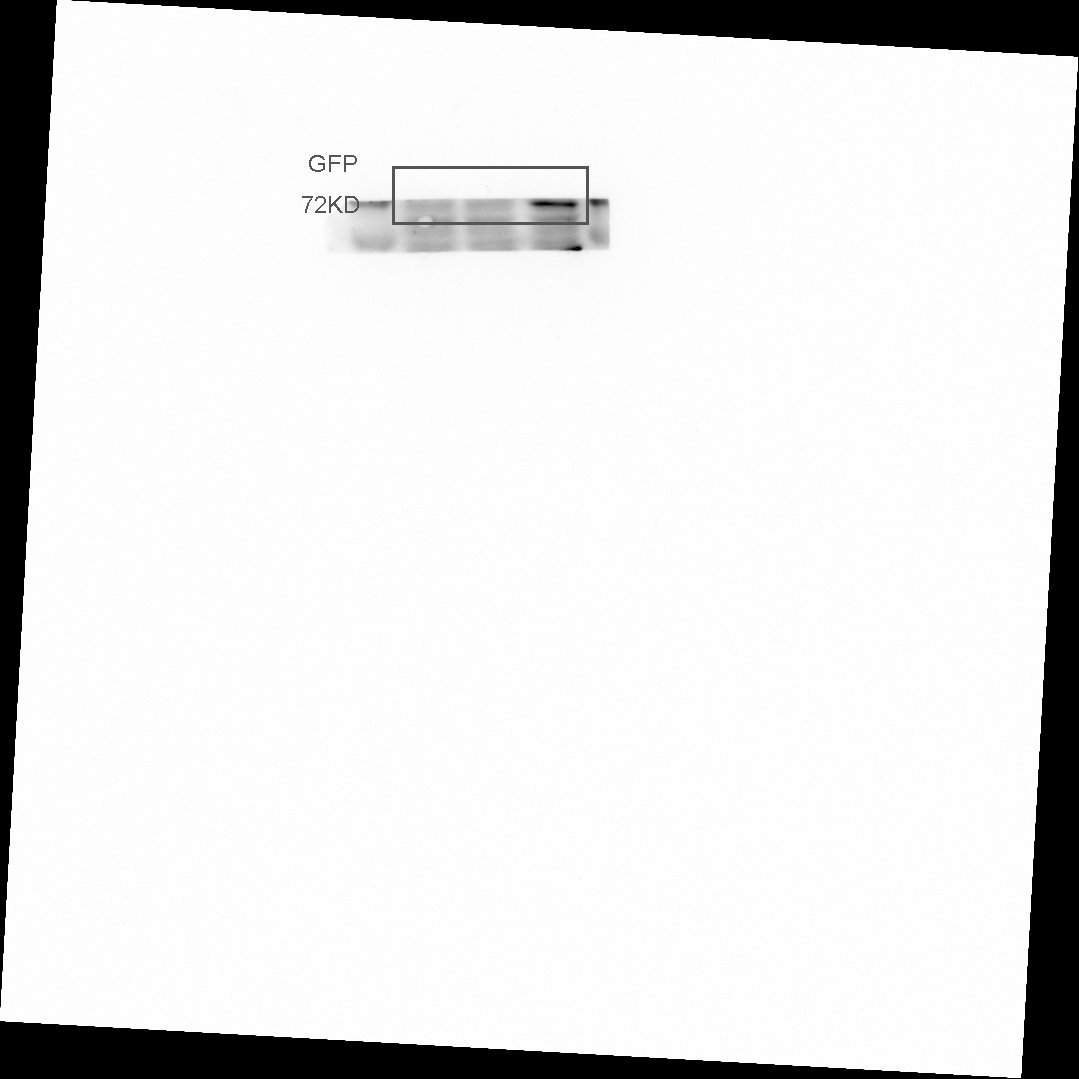

Supplement: Supplementary file 9 — Source Data Fig. 6 [file 44318_2023_3_MOESM9_ESM.zip › Figure6/6e/GFP L2-NC shDC1-1-Vector shDC1-1-N.jpg]

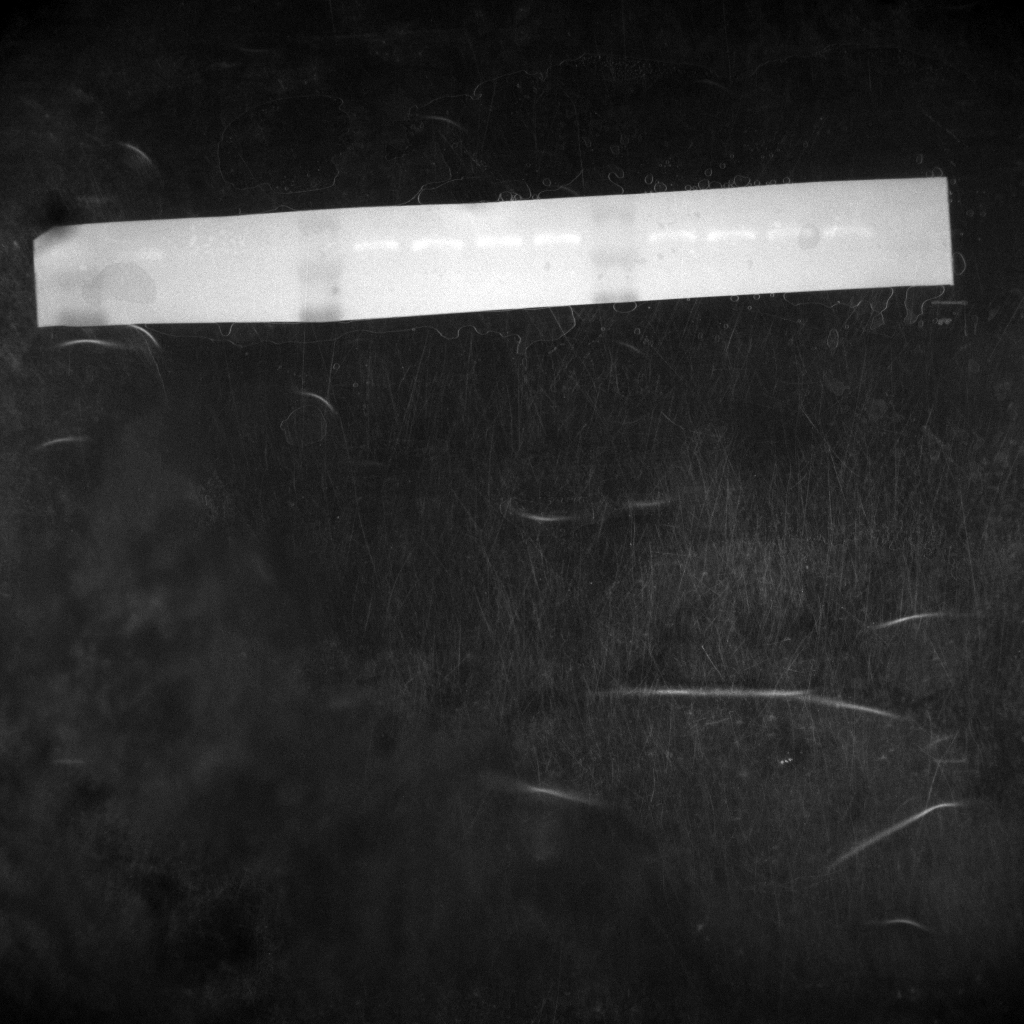

Supplement: Supplementary file 9 — Source Data Fig. 6 [file 44318_2023_3_MOESM9_ESM.zip › Figure6/6e/YTHDC1 L2-NC shDC1-1-Vector shDC1-1-N W.tif]

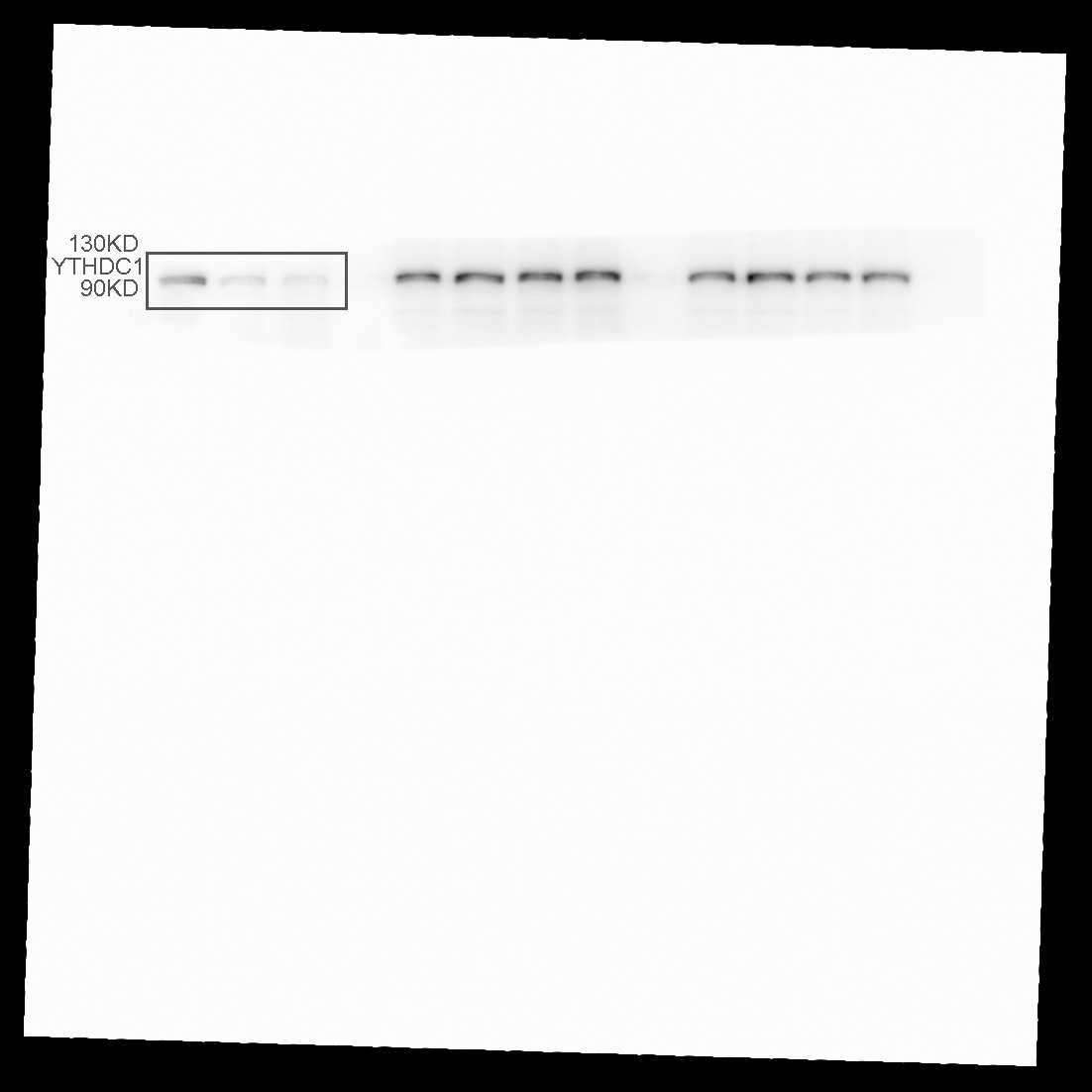

Supplement: Supplementary file 9 — Source Data Fig. 6 [file 44318_2023_3_MOESM9_ESM.zip › Figure6/6e/YTHDC1 L2-NC shDC1-1-Vector shDC1-1-N.jpg]

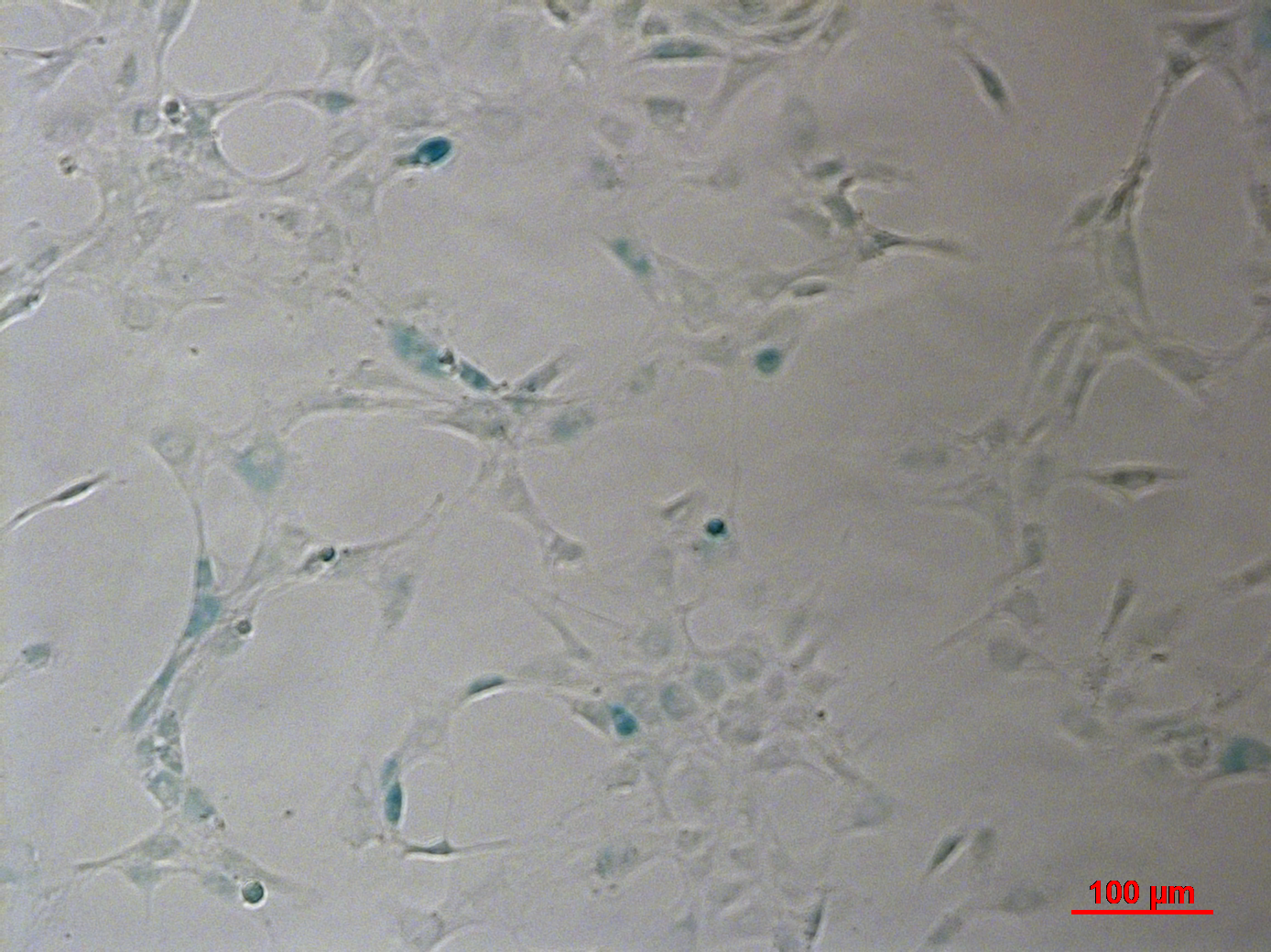

Supplement: Supplementary file 9 — Source Data Fig. 6 [file 44318_2023_3_MOESM9_ESM.zip › Figure6/6f-g/nc SA-β-gal/nc.TIF]

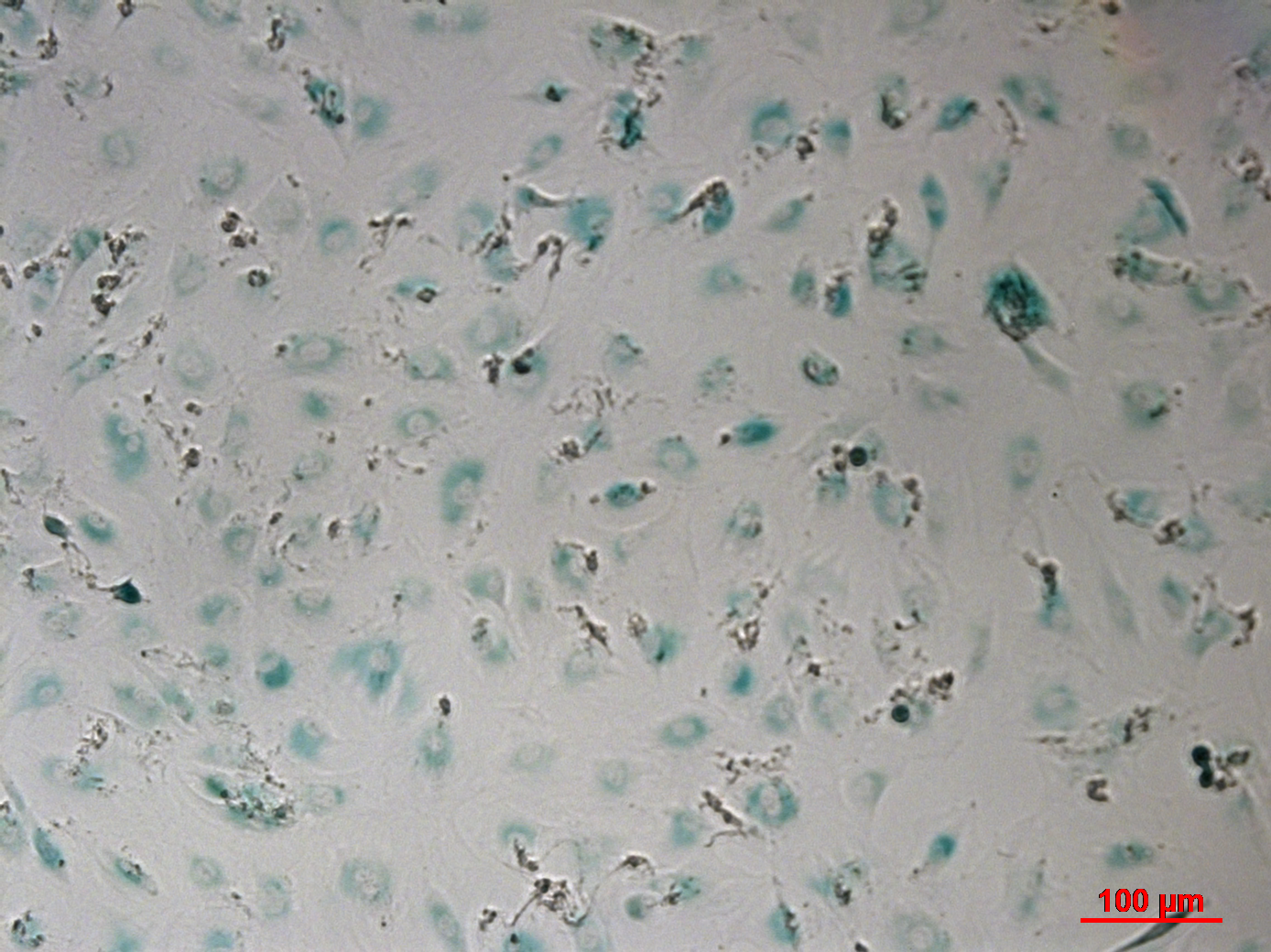

Supplement: Supplementary file 9 — Source Data Fig. 6 [file 44318_2023_3_MOESM9_ESM.zip › Figure6/6f-g/siYTHDC1 SA-β-gal/siDC1.TIF]

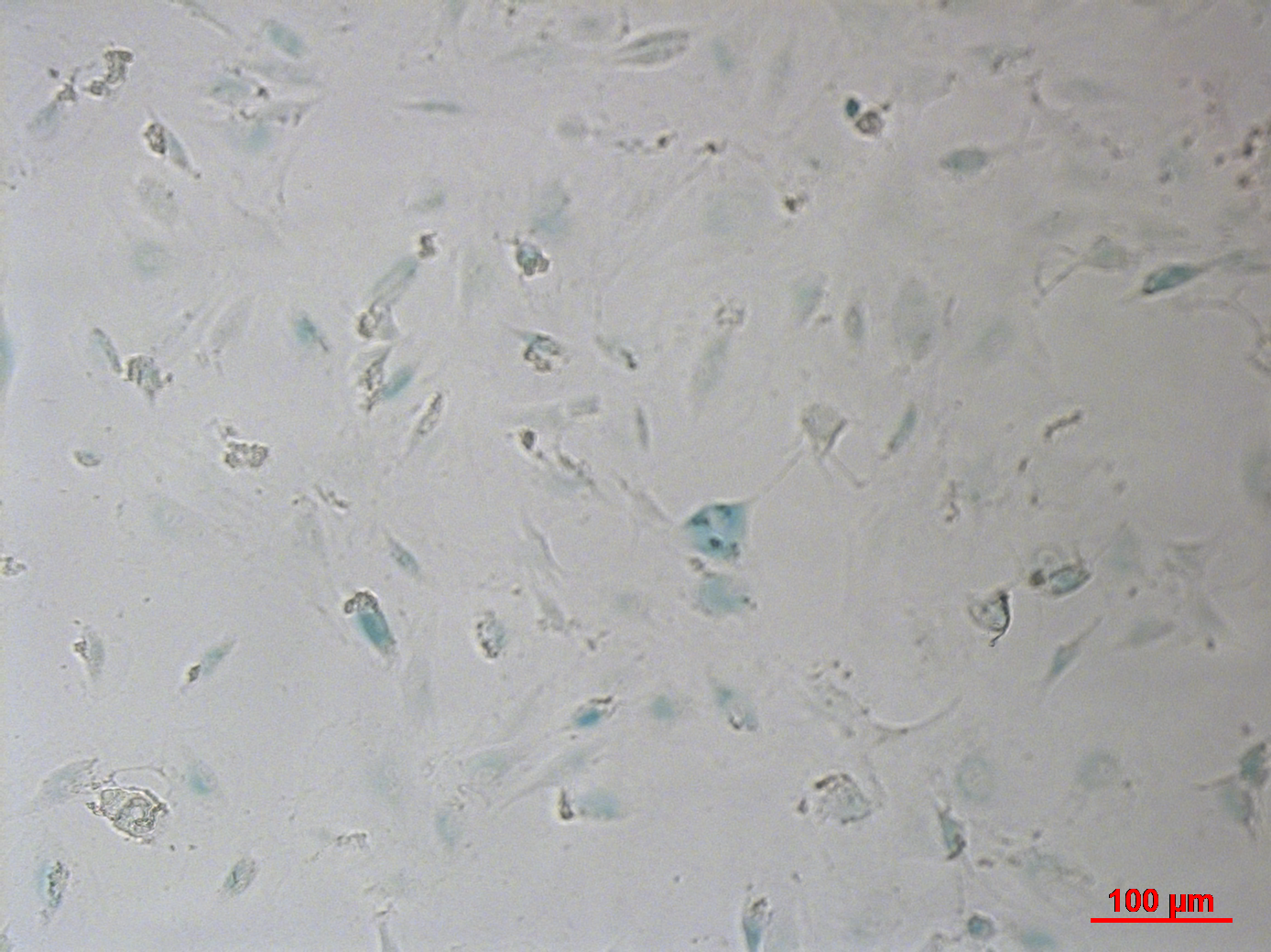

Supplement: Supplementary file 9 — Source Data Fig. 6 [file 44318_2023_3_MOESM9_ESM.zip › Figure6/6f-g/siYTHDC1+NTD SA-β-gal/siDC1+NTD.TIF]

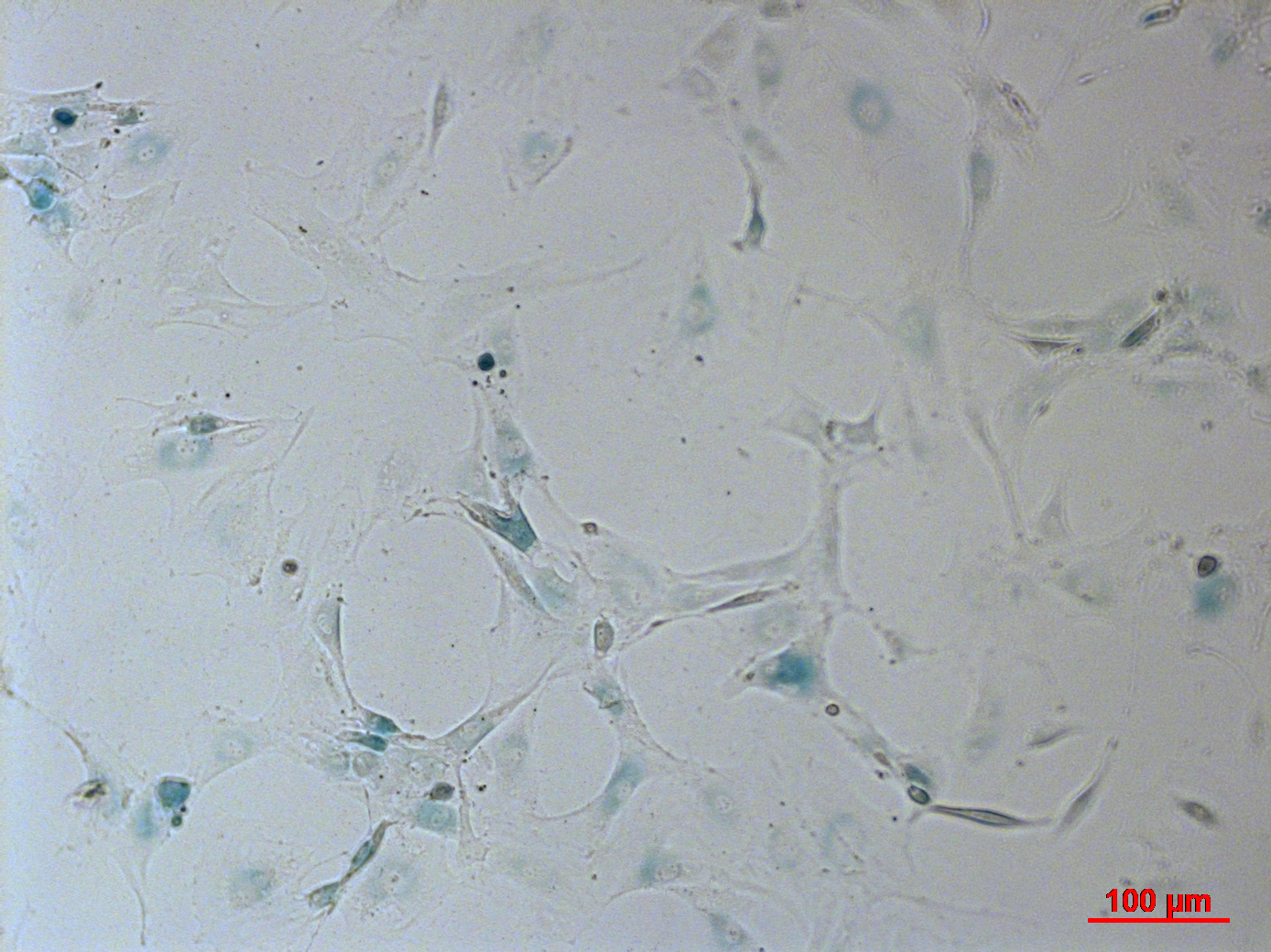

Supplement: Supplementary file 9 — Source Data Fig. 6 [file 44318_2023_3_MOESM9_ESM.zip › Figure6/6i-j/vector+NC/vector+NC.TIF]

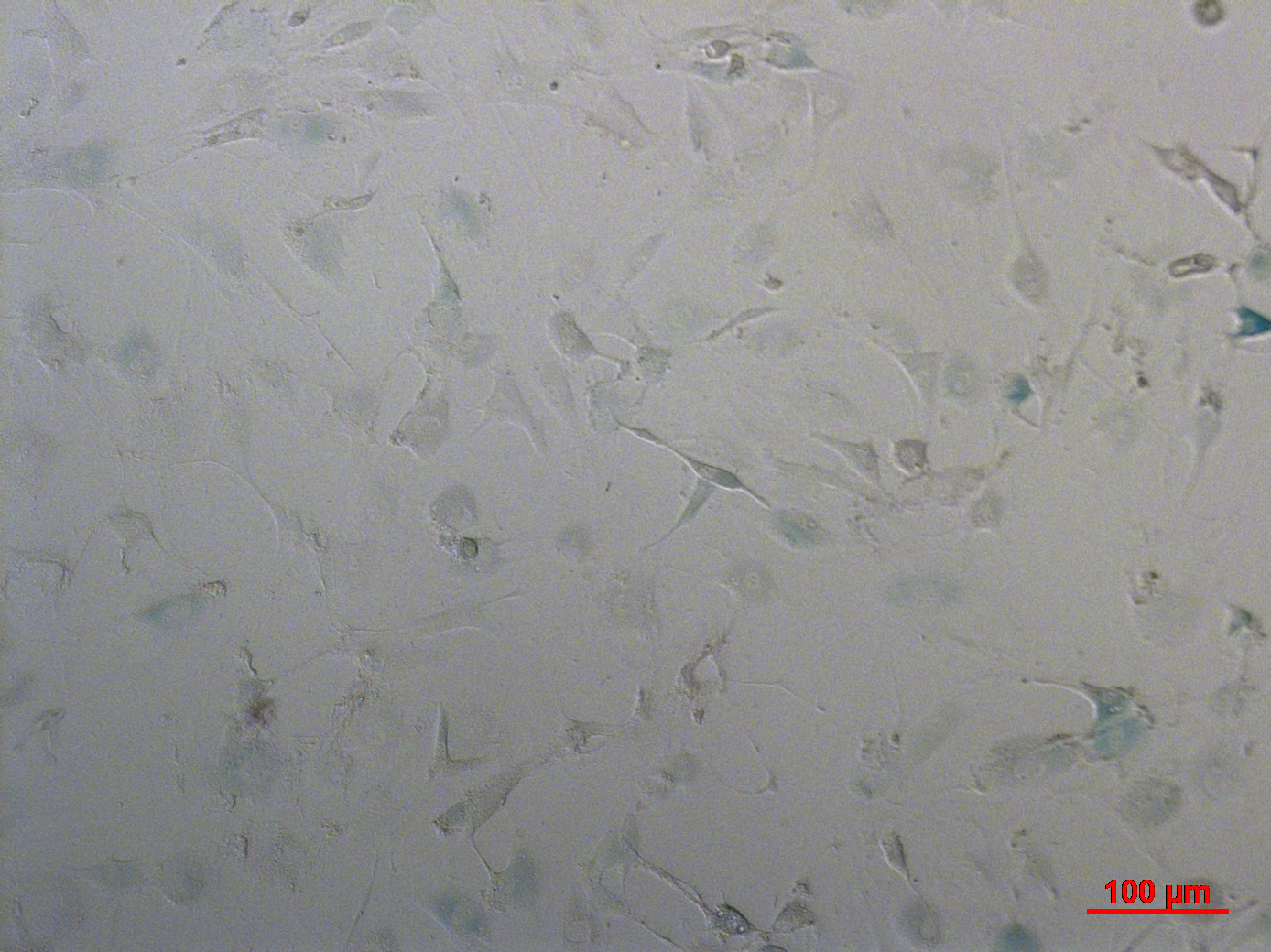

Supplement: Supplementary file 9 — Source Data Fig. 6 [file 44318_2023_3_MOESM9_ESM.zip › Figure6/6i-j/YTHDC1+NC/YTHDC1+NC.TIF]

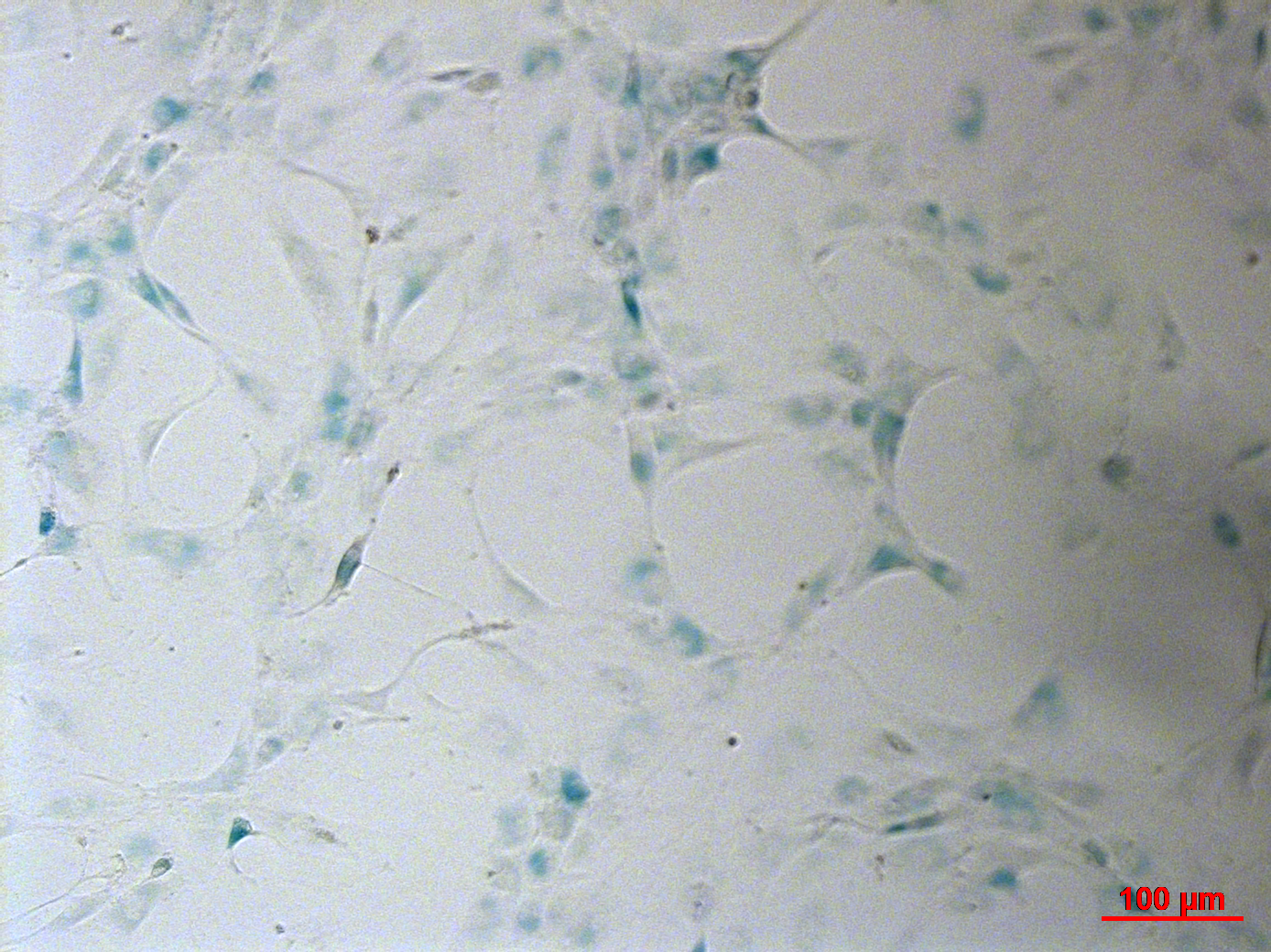

Supplement: Supplementary file 9 — Source Data Fig. 6 [file 44318_2023_3_MOESM9_ESM.zip › Figure6/6i-j/YTHDC1+siTopBP1/YTHDC1+siTopBP1.TIF]

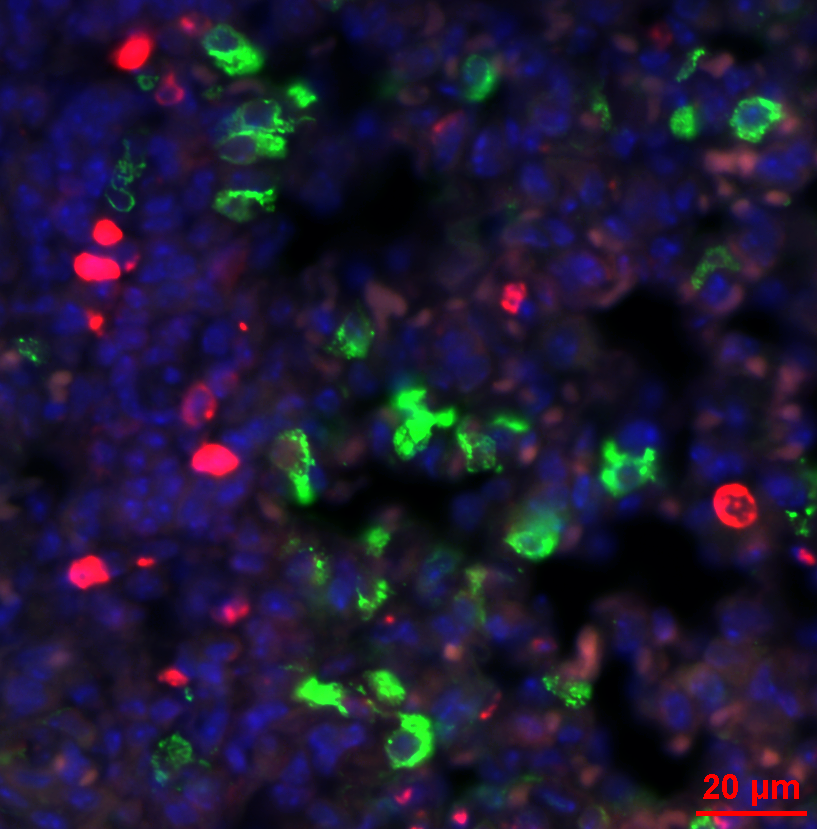

Supplement: Supplementary file 10 — Source Data EV Fig. 1 [file 44318_2023_3_MOESM10_ESM.zip › Figure EV1/1c-d/FTO SPC/blm FTO SPC _crop/blm fto spc _crop.tif]

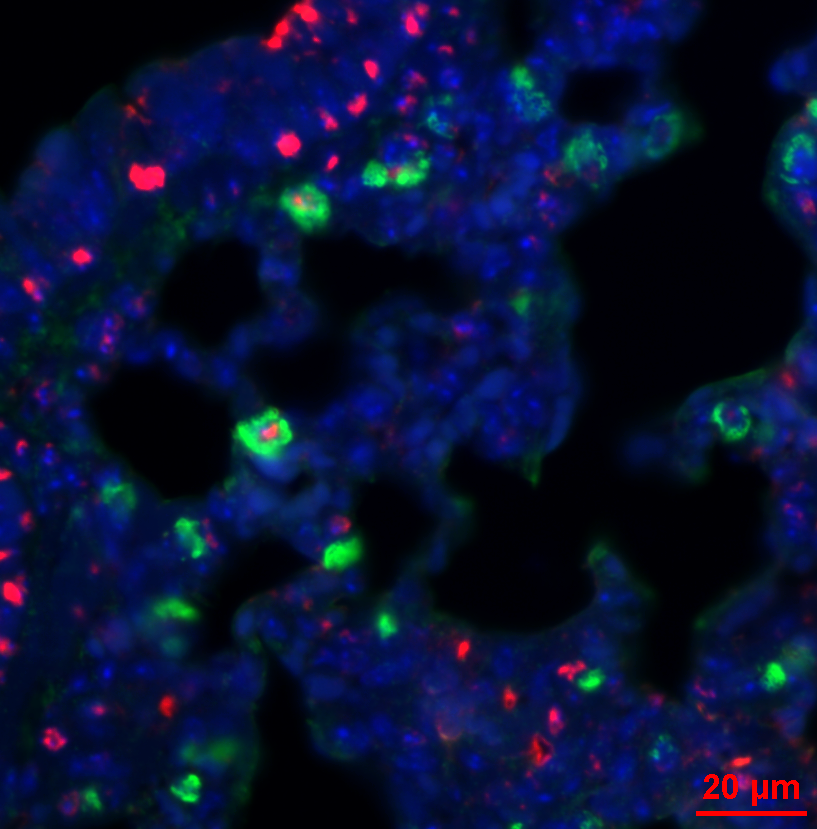

Supplement: Supplementary file 10 — Source Data EV Fig. 1 [file 44318_2023_3_MOESM10_ESM.zip › Figure EV1/1c-d/FTO SPC/saline FTO SPC _crop/saline fto spc _crop.tif]

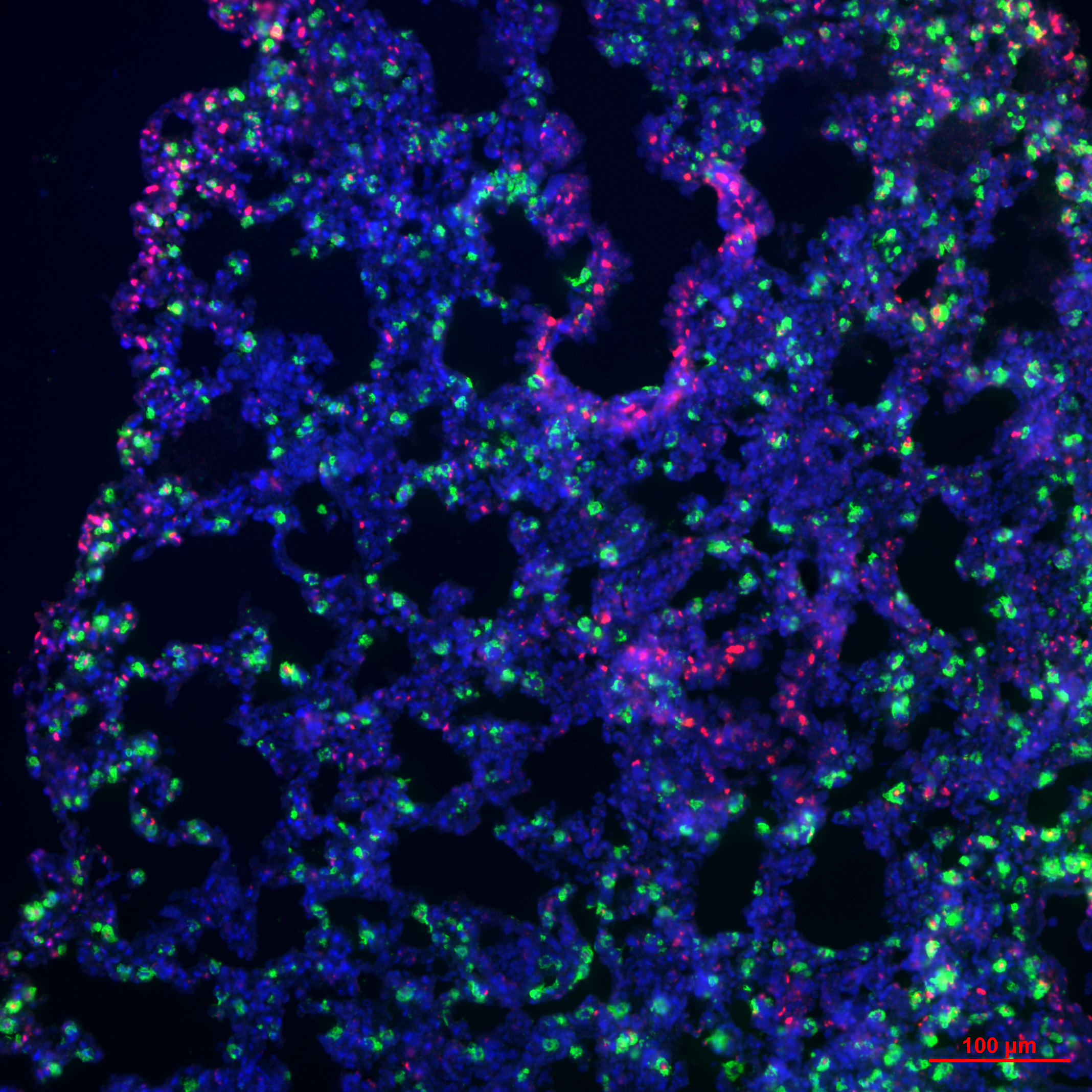

Supplement: Supplementary file 10 — Source Data EV Fig. 1 [file 44318_2023_3_MOESM10_ESM.zip › Figure EV1/1c-d/WTAP SPC/BLM WTAP SPC.tif]

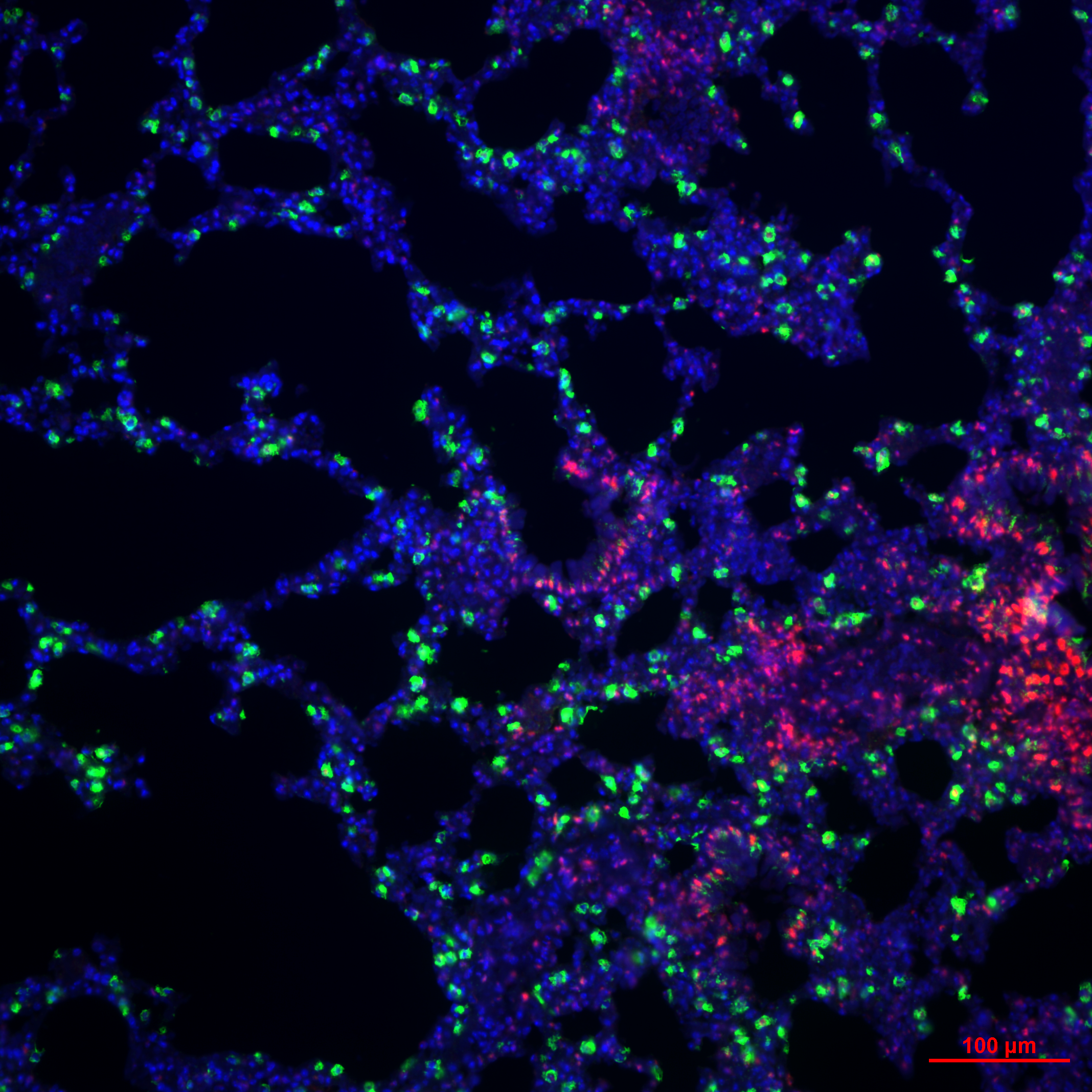

Supplement: Supplementary file 10 — Source Data EV Fig. 1 [file 44318_2023_3_MOESM10_ESM.zip › Figure EV1/1c-d/WTAP SPC/saline WTAP SPC.tif]

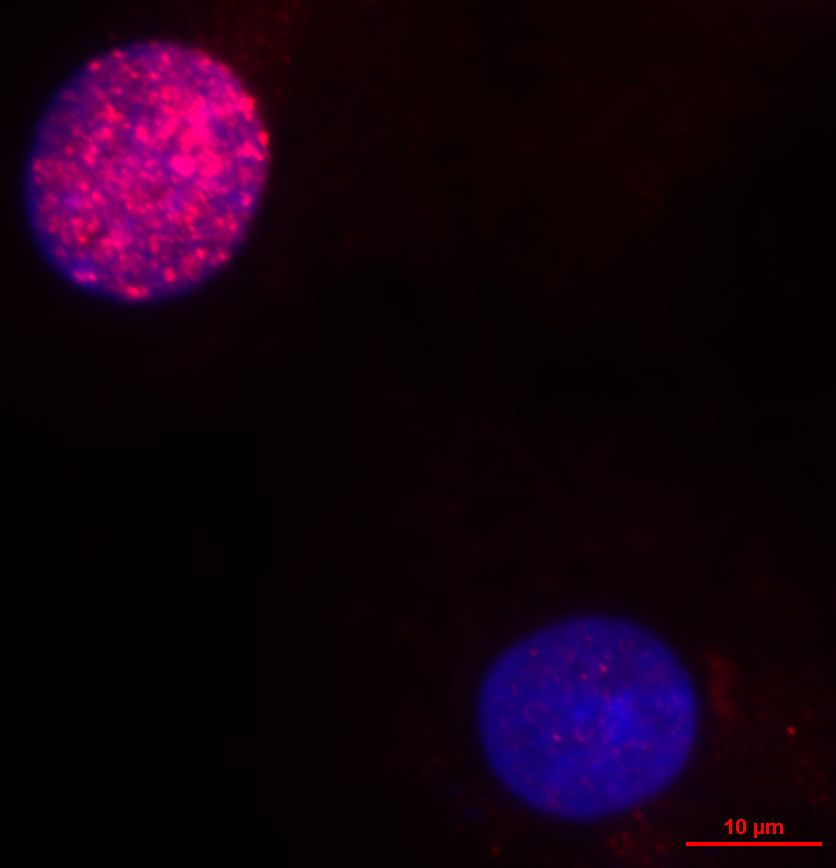

Supplement: Supplementary file 10 — Source Data EV Fig. 1 [file 44318_2023_3_MOESM10_ESM.zip › Figure EV1/1e-f/nc blm ki67.tif]

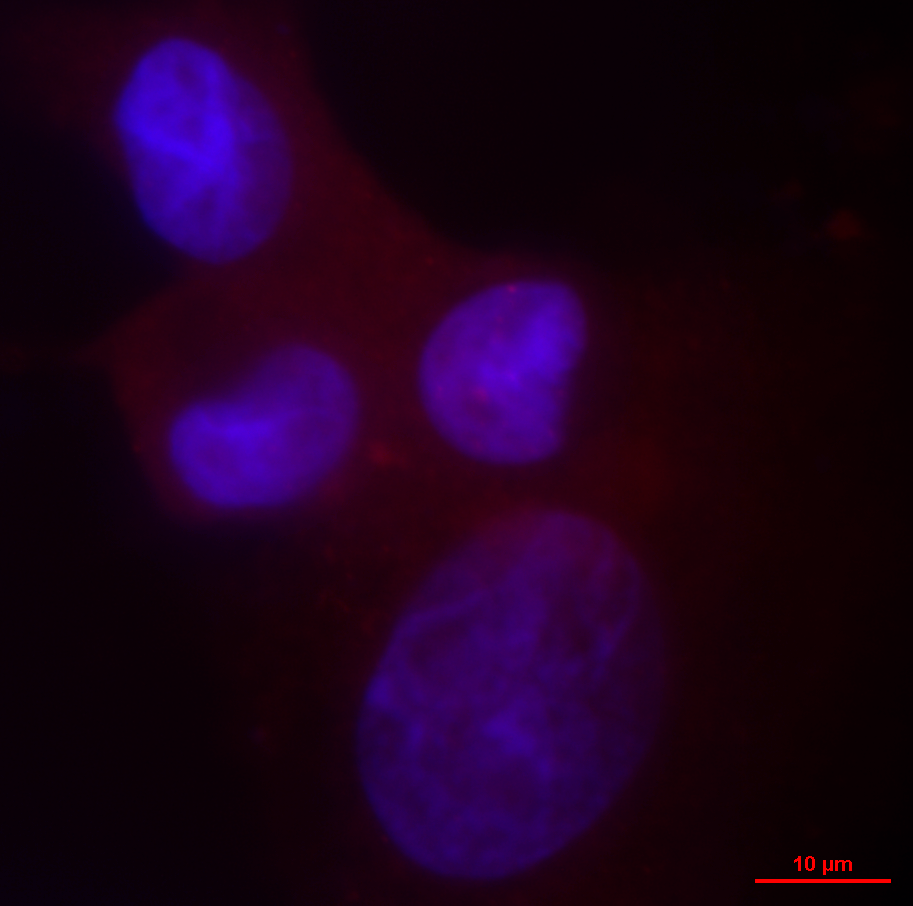

Supplement: Supplementary file 10 — Source Data EV Fig. 1 [file 44318_2023_3_MOESM10_ESM.zip › Figure EV1/1e-f/siYTHDC1 BLM ki67.tif]

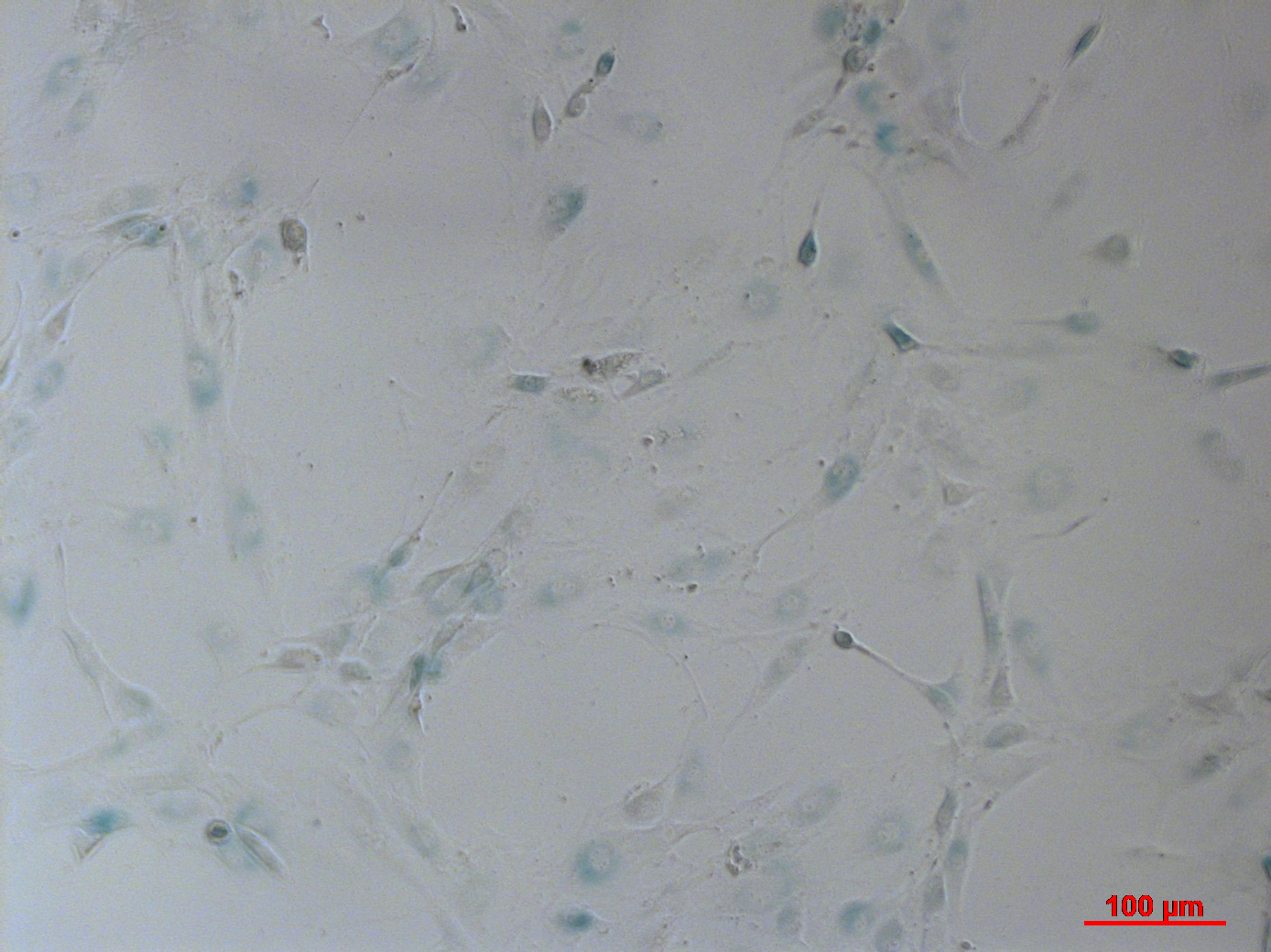

Supplement: Supplementary file 10 — Source Data EV Fig. 1 [file 44318_2023_3_MOESM10_ESM.zip › Figure EV1/1h-i/nc β-gal/nc.TIF]

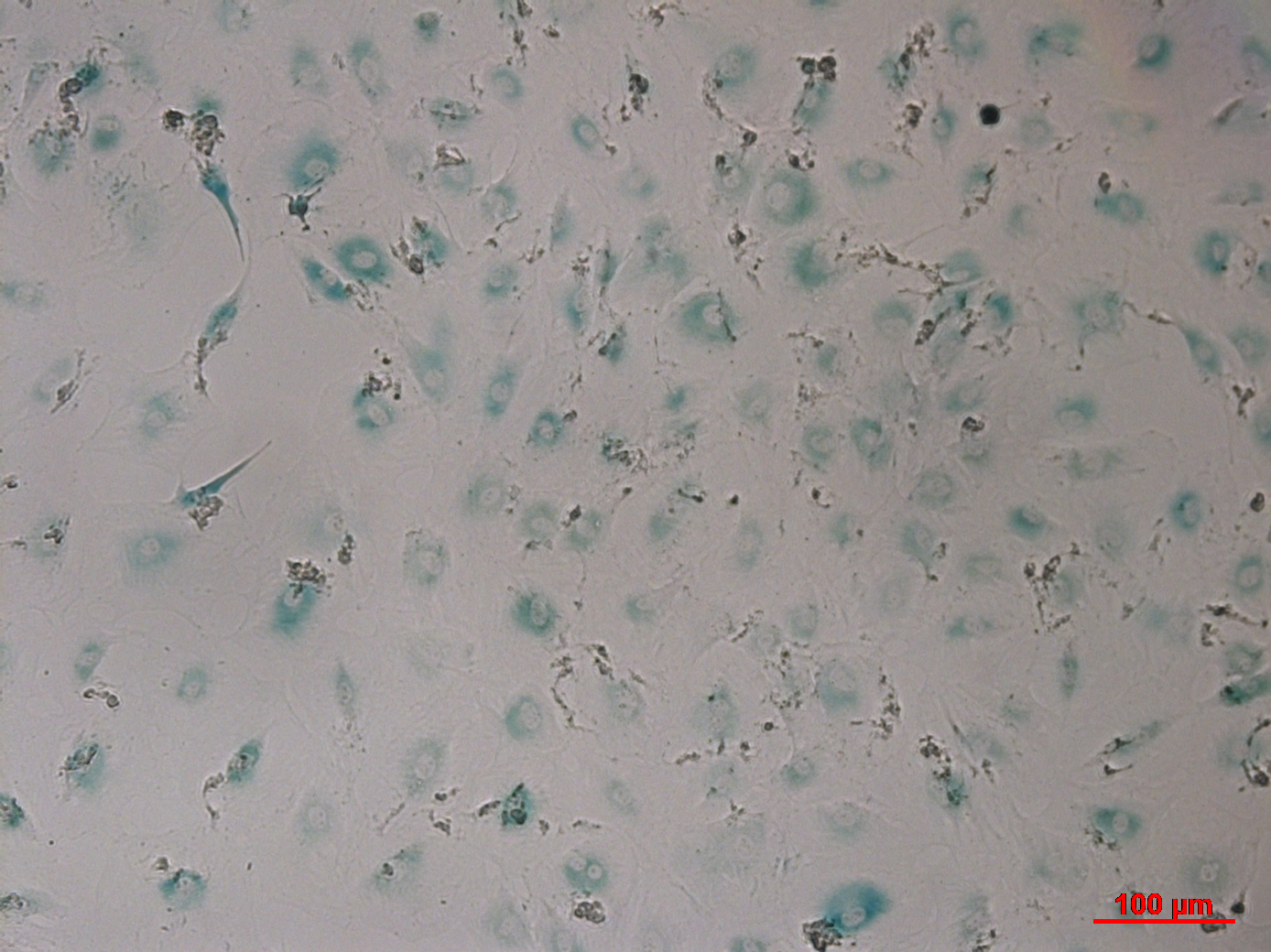

Supplement: Supplementary file 10 — Source Data EV Fig. 1 [file 44318_2023_3_MOESM10_ESM.zip › Figure EV1/1h-i/siYTHDC1 β-gal/siDC1-2.TIF]

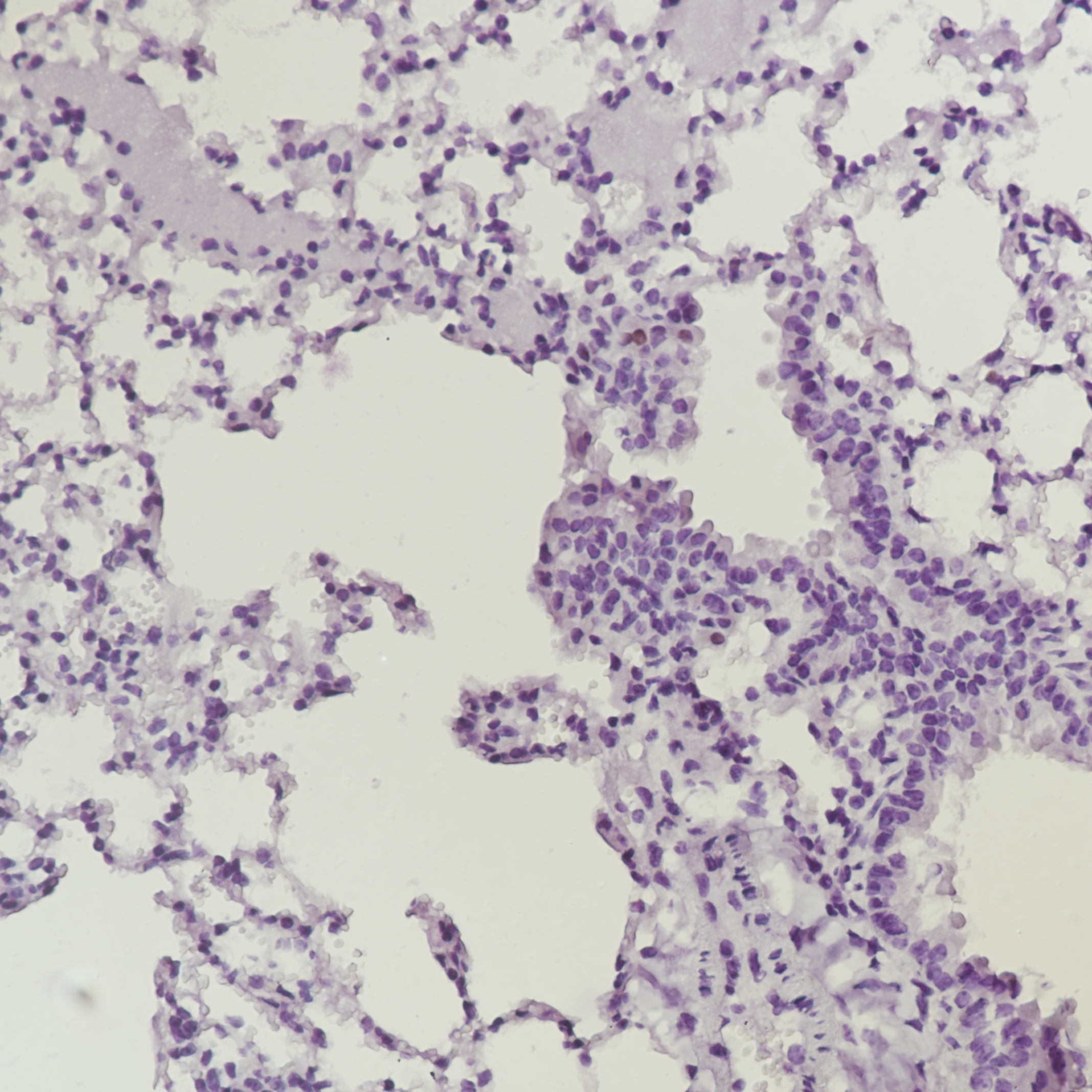

Supplement: Supplementary file 10 — Source Data EV Fig. 1 [file 44318_2023_3_MOESM10_ESM.zip › Figure EV1/1j-k/nc p21.tif]

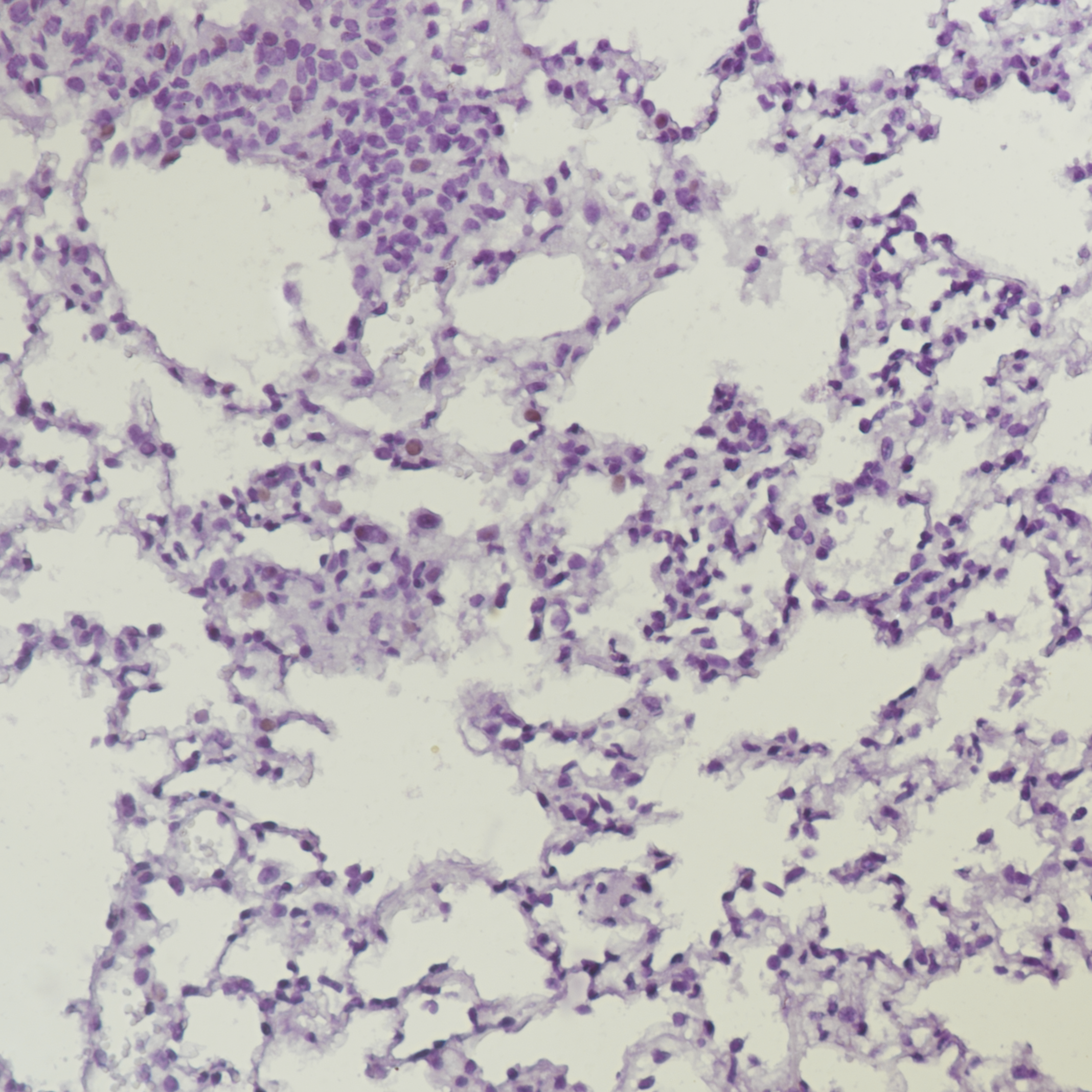

Supplement: Supplementary file 10 — Source Data EV Fig. 1 [file 44318_2023_3_MOESM10_ESM.zip › Figure EV1/1j-k/saline p21.tif]

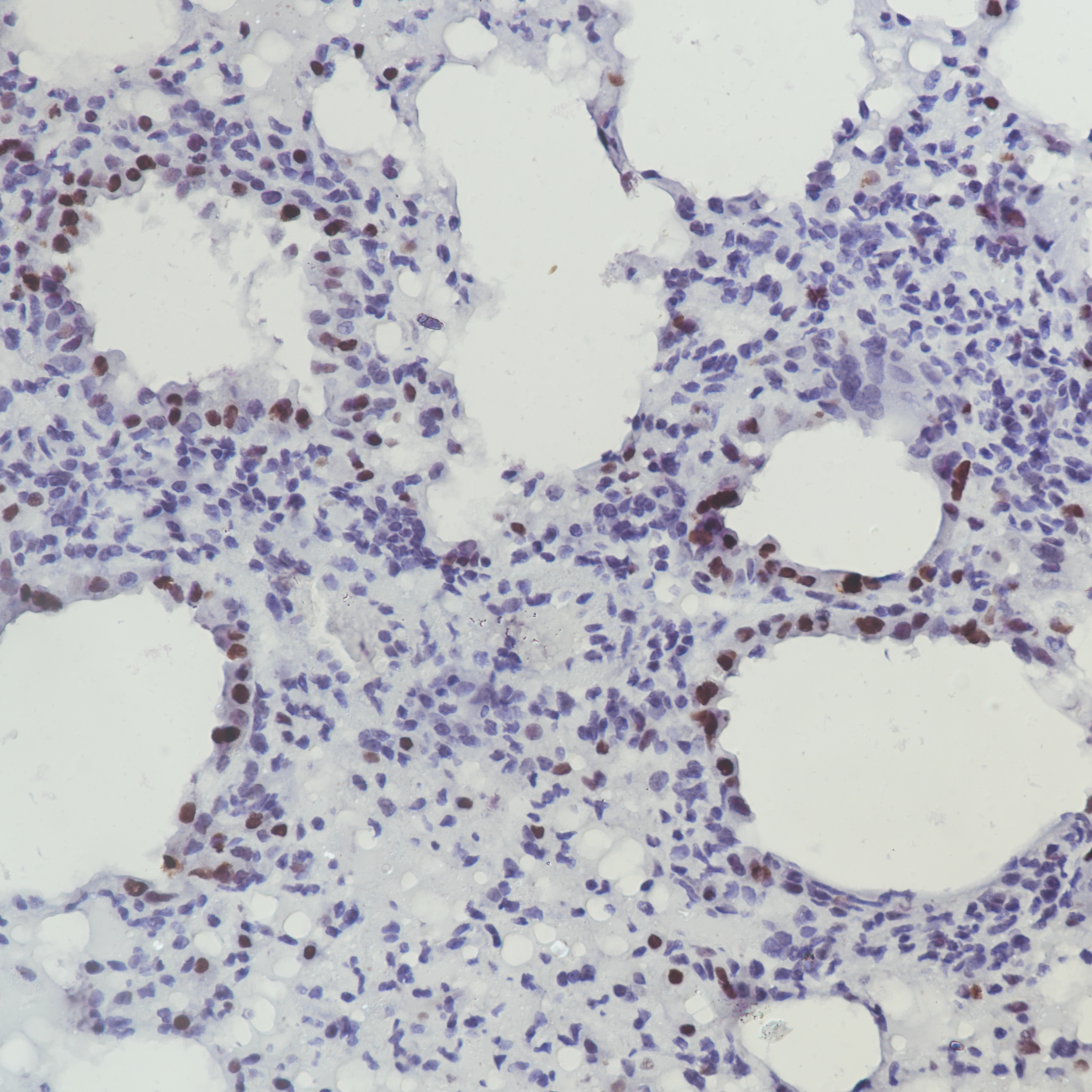

Supplement: Supplementary file 10 — Source Data EV Fig. 1 [file 44318_2023_3_MOESM10_ESM.zip › Figure EV1/1j-k/shYTHDC1 P21.tif]

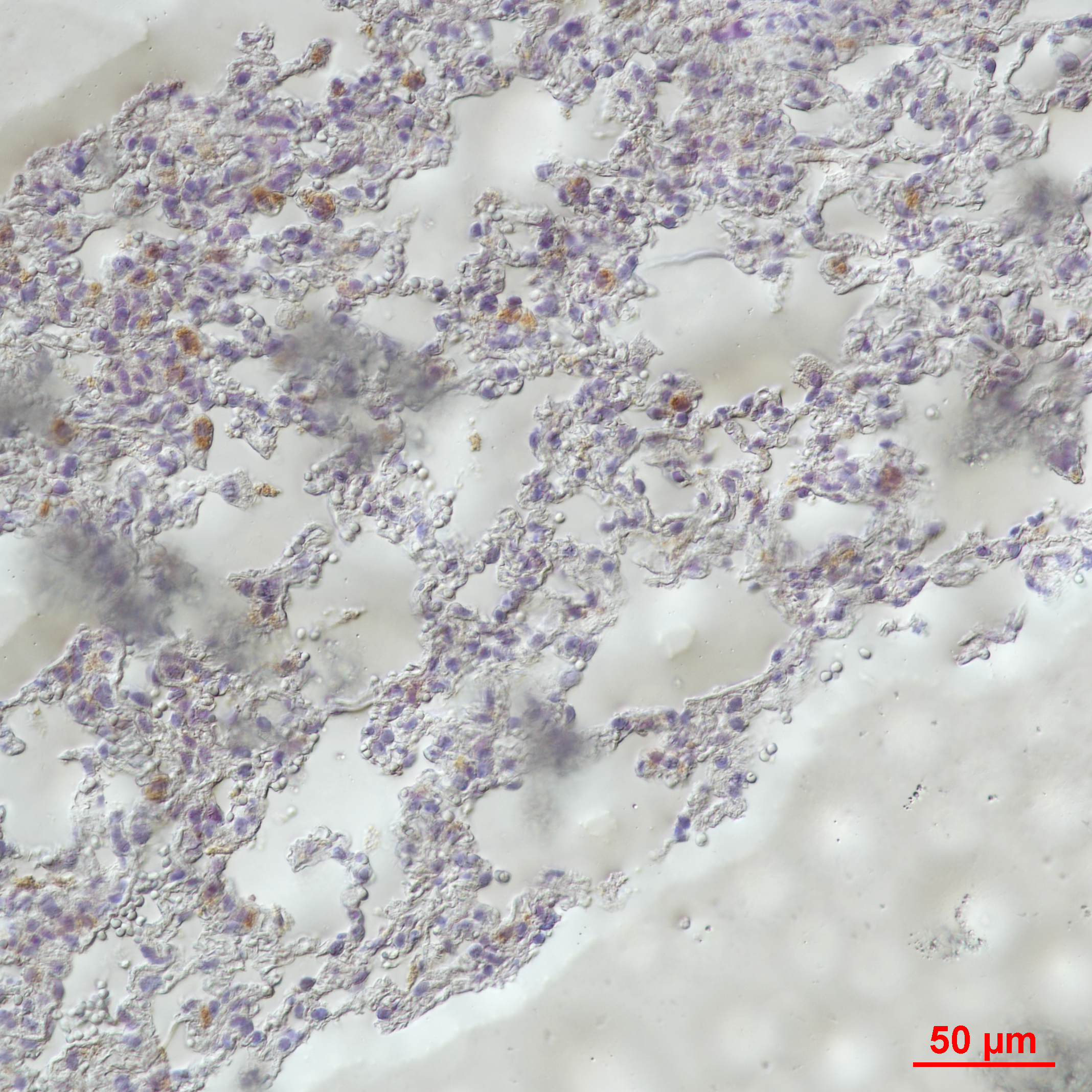

Supplement: Supplementary file 10 — Source Data EV Fig. 1 [file 44318_2023_3_MOESM10_ESM.zip › Figure EV1/1l-m/nc p16.tif]

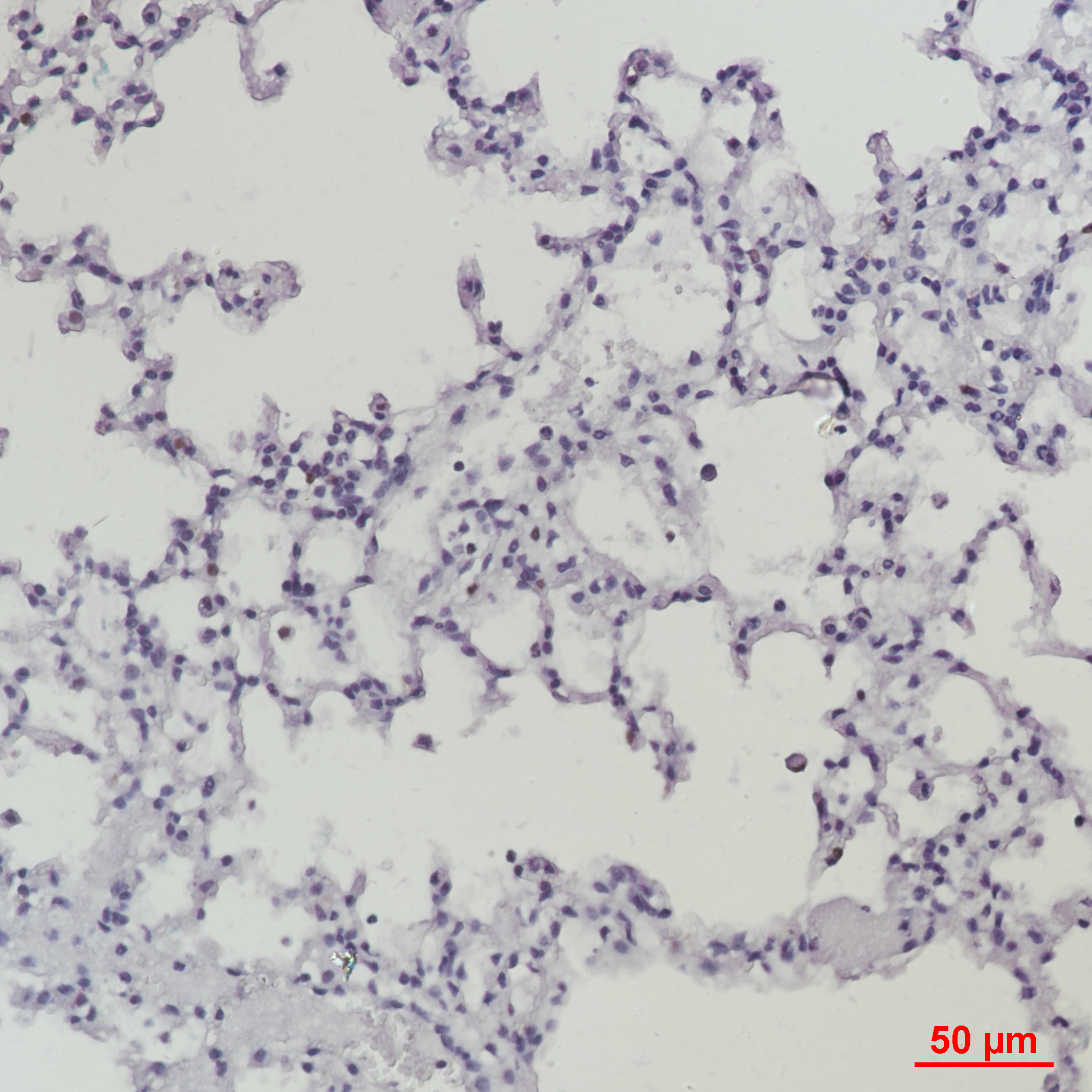

Supplement: Supplementary file 10 — Source Data EV Fig. 1 [file 44318_2023_3_MOESM10_ESM.zip › Figure EV1/1l-m/saline P16.tif]

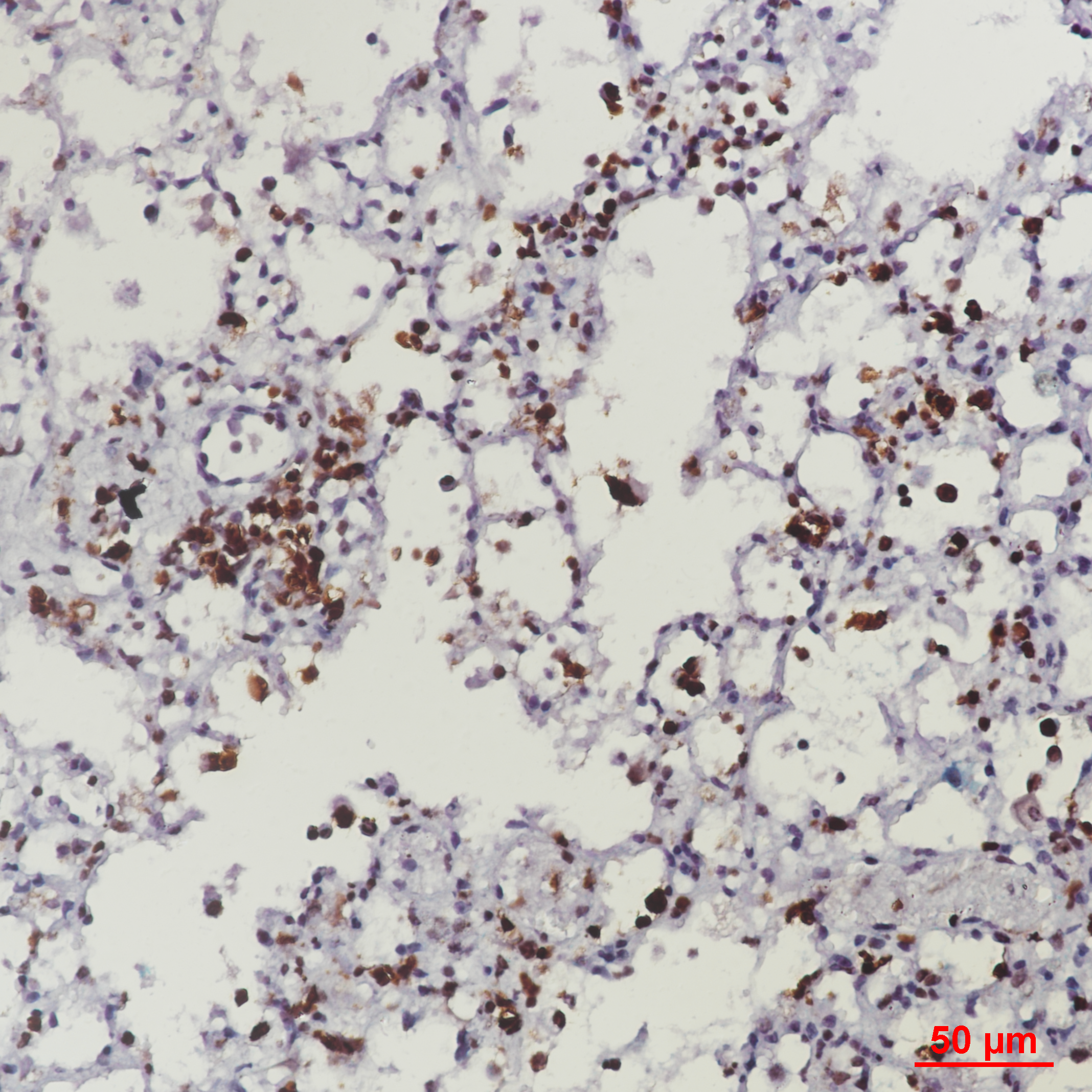

Supplement: Supplementary file 10 — Source Data EV Fig. 1 [file 44318_2023_3_MOESM10_ESM.zip › Figure EV1/1l-m/shYTHDC1 p16.tif]

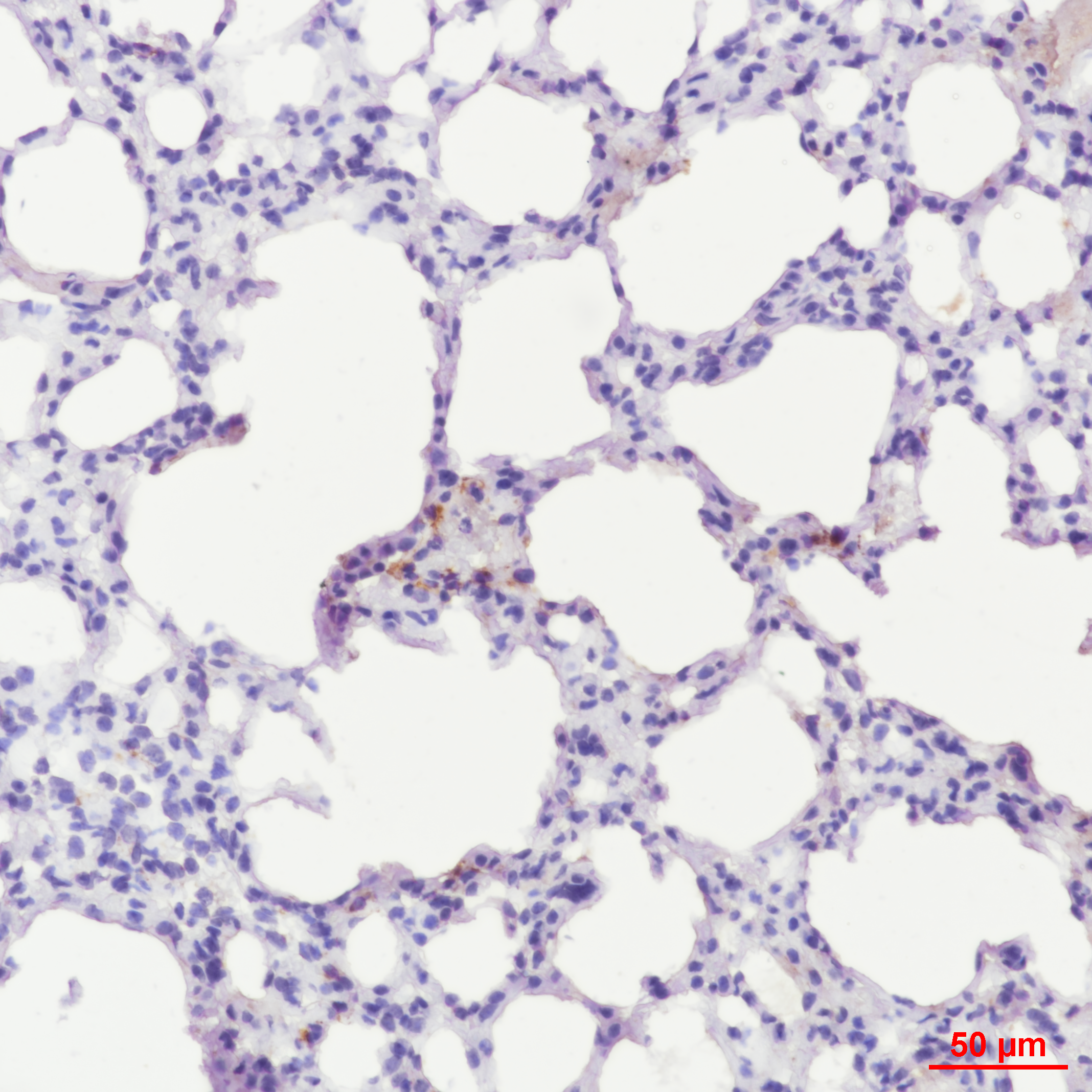

Supplement: Supplementary file 10 — Source Data EV Fig. 1 [file 44318_2023_3_MOESM10_ESM.zip › Figure EV1/1n-o/NC a-SMA.tif]

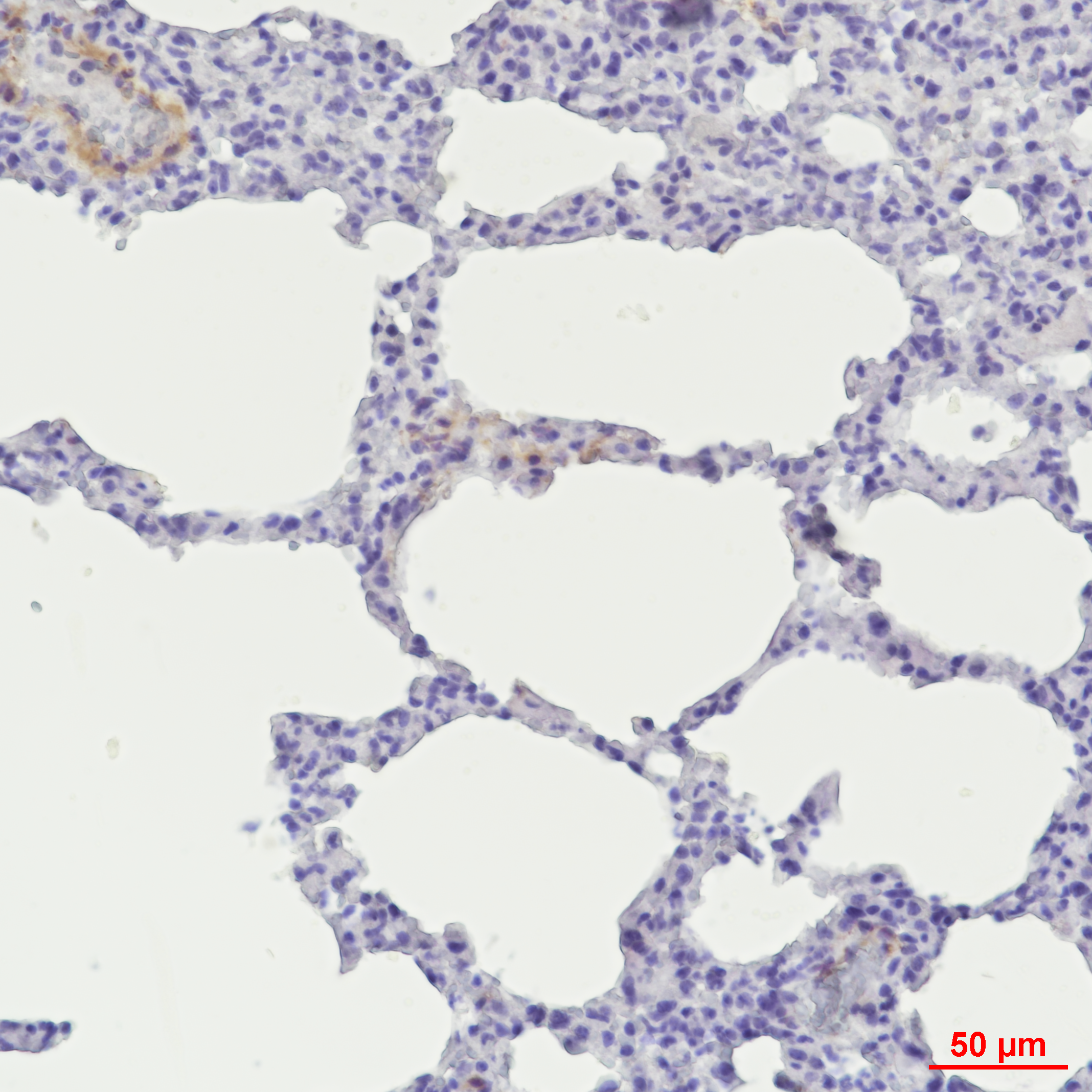

Supplement: Supplementary file 10 — Source Data EV Fig. 1 [file 44318_2023_3_MOESM10_ESM.zip › Figure EV1/1n-o/saline a-SMA.tif]

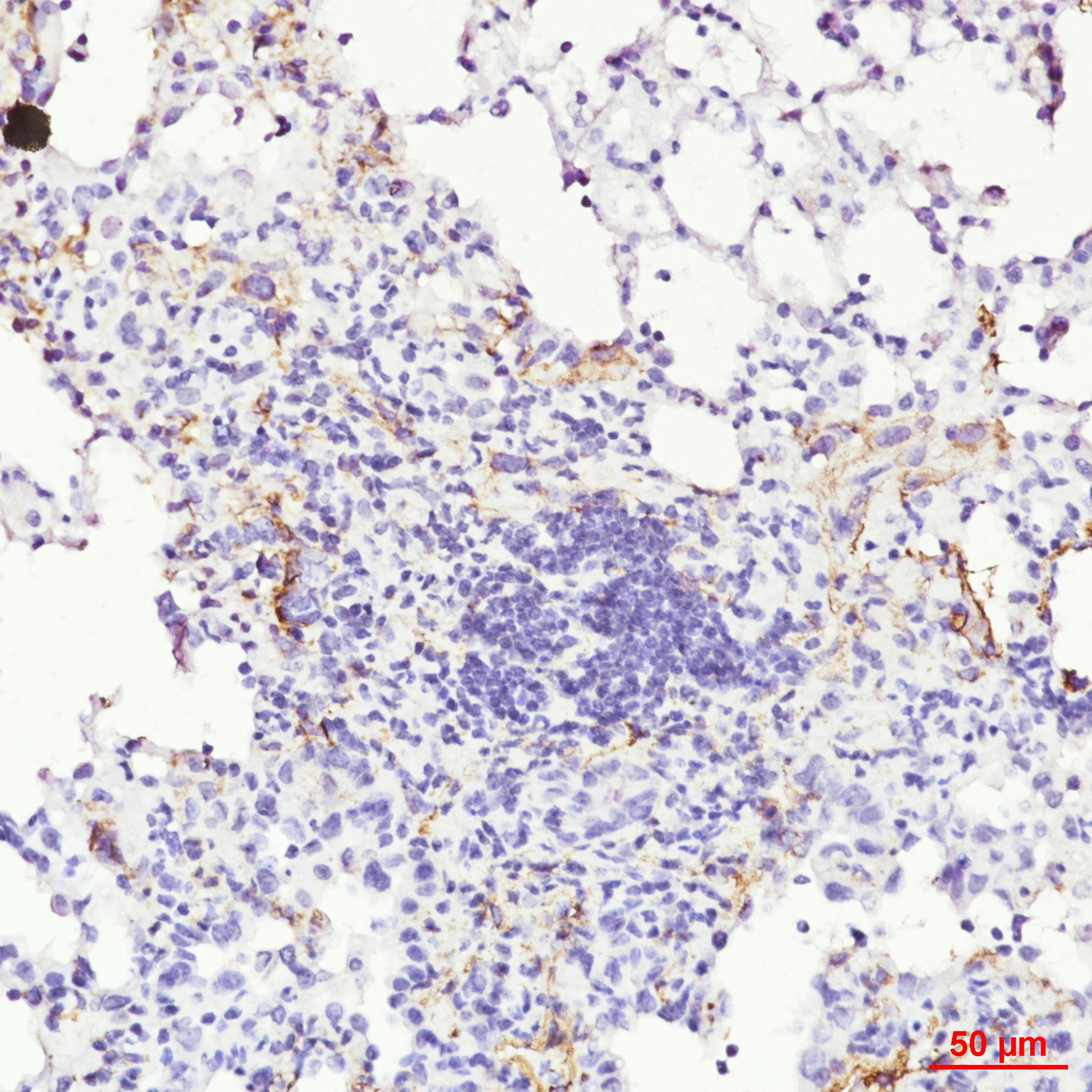

Supplement: Supplementary file 10 — Source Data EV Fig. 1 [file 44318_2023_3_MOESM10_ESM.zip › Figure EV1/1n-o/shYTHDC1 a-SMA.tif]

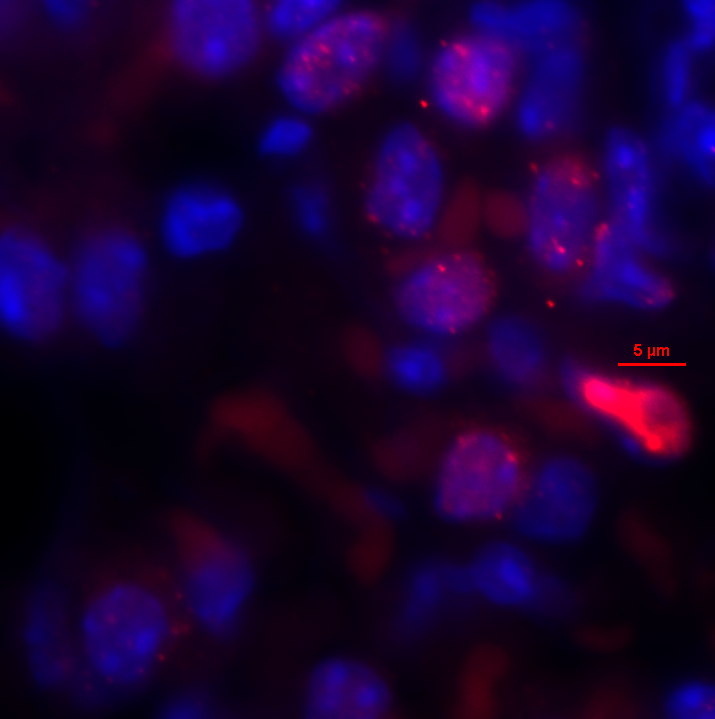

Supplement: Supplementary file 10 — Source Data EV Fig. 1 [file 44318_2023_3_MOESM10_ESM.zip › Figure EV1/1p-r/nc yh2ax IF/nc yh2ax IF.tif]
