# Supplementary figures and images for: YTHDC1 delays cellular senescence and pulmonary fibrosis by activating ATR in an m6A-independent manner (part 3 of 4)
Source: EMBO J. 2023 Dec 15;43(1):4. doi: 10.1038/s44318-023-00003-2 (PMC10883269; doi:10.1038/s44318-023-00003-2)

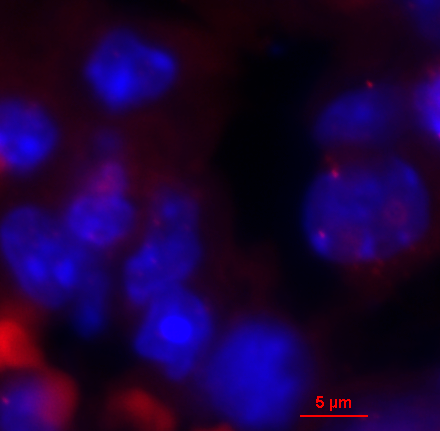

Supplement: Supplementary file 10 — Source Data EV Fig. 1 [file 44318_2023_3_MOESM10_ESM.zip › Figure EV1/1p-r/saline yh2ax IF/saline yh2ax IF.tif]

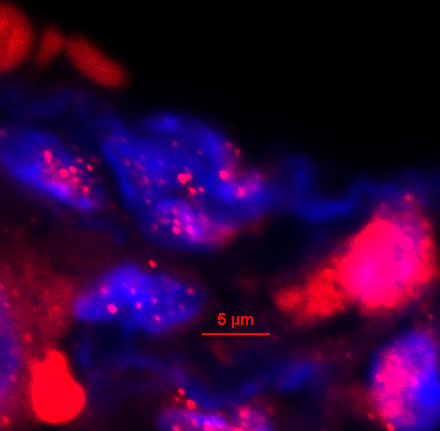

Supplement: Supplementary file 10 — Source Data EV Fig. 1 [file 44318_2023_3_MOESM10_ESM.zip › Figure EV1/1p-r/shYTHDC1 yh2ax IF/sh1 011.tif]

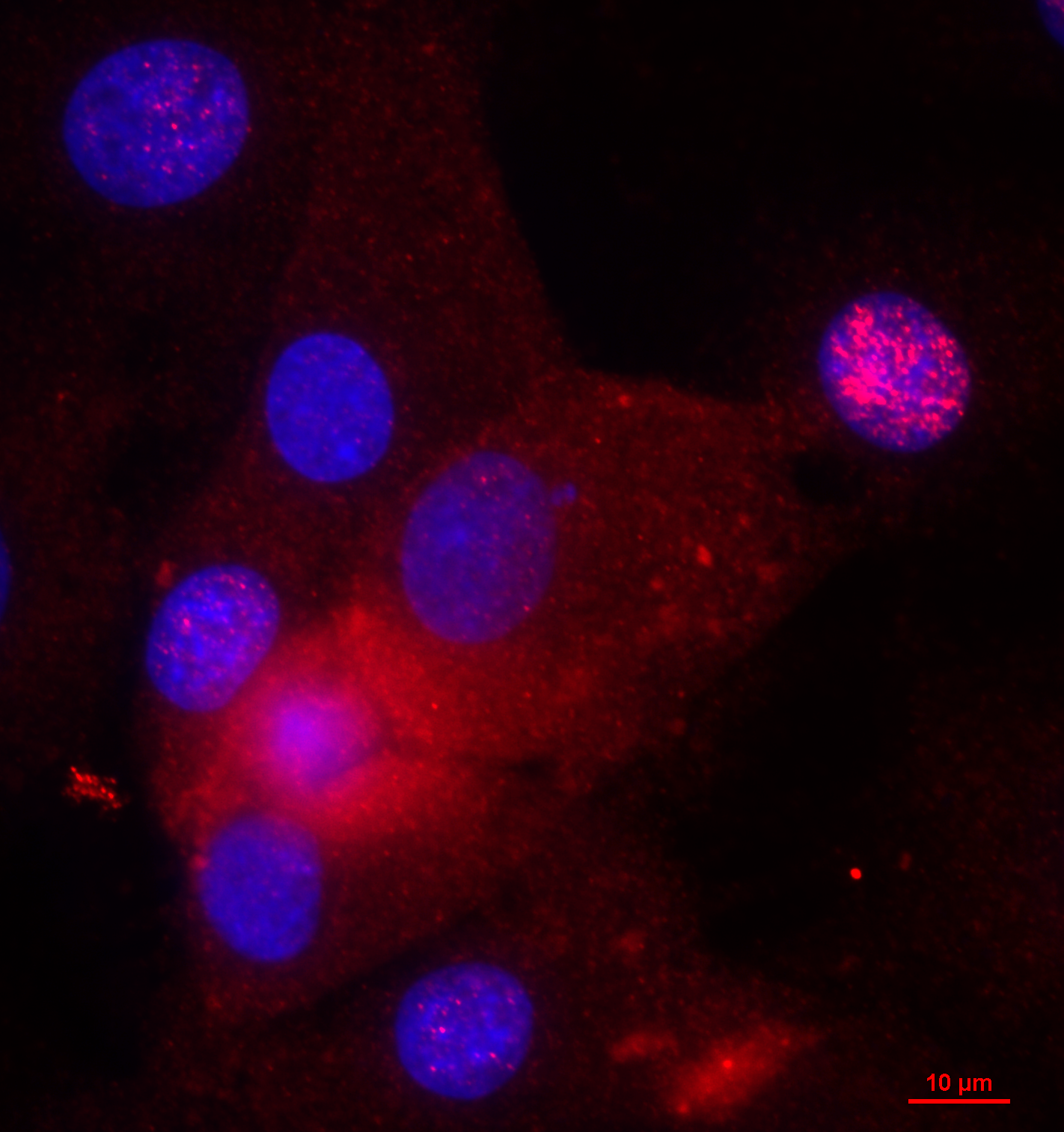

Supplement: Supplementary file 11 — Source Data EV Fig. 2 [file 44318_2023_3_MOESM11_ESM.zip › Figure EV2/2b-c/Vector ki67 IF.tif]

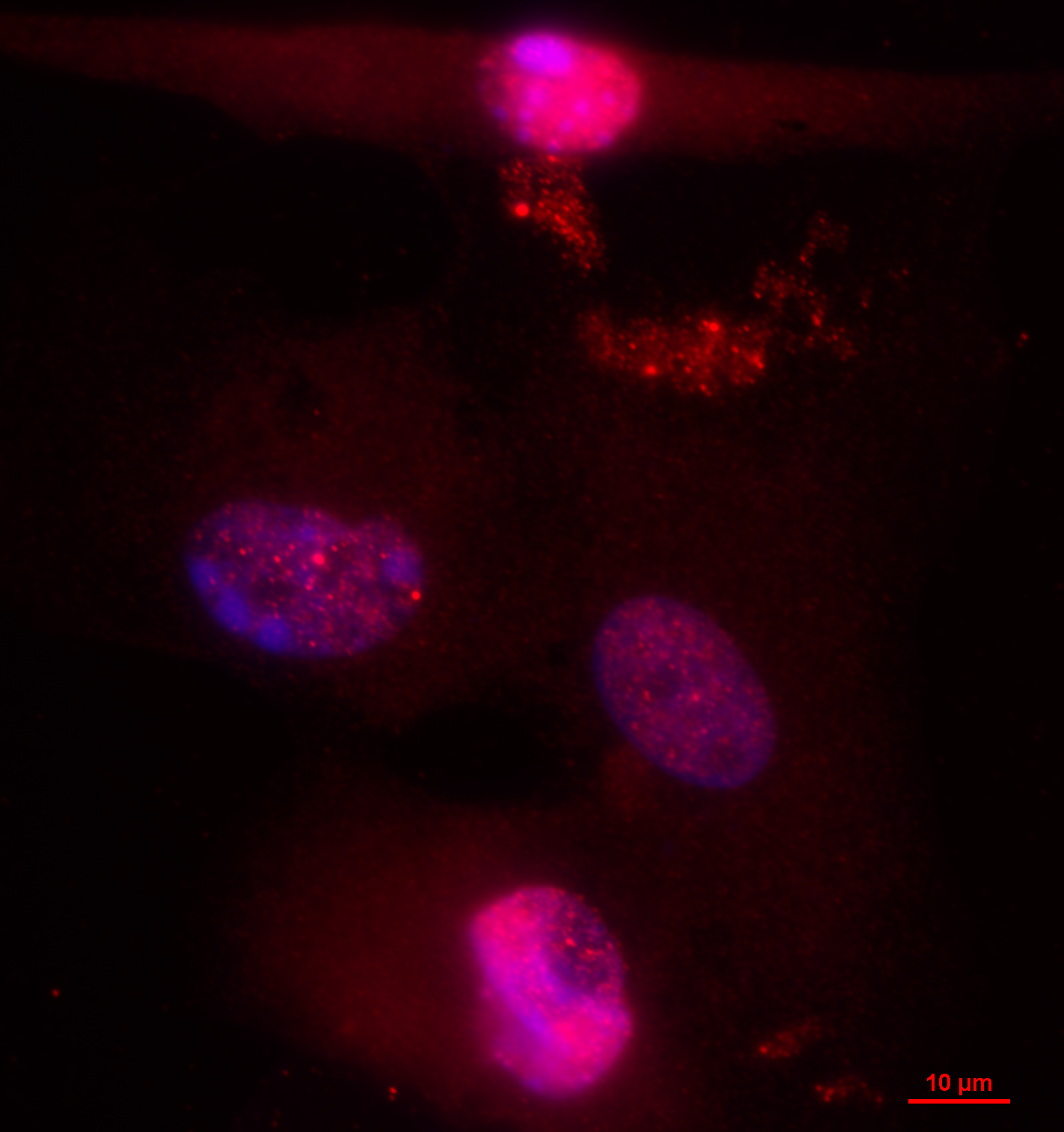

Supplement: Supplementary file 11 — Source Data EV Fig. 2 [file 44318_2023_3_MOESM11_ESM.zip › Figure EV2/2b-c/YTHDC1-MUT ki67 IF.tif]

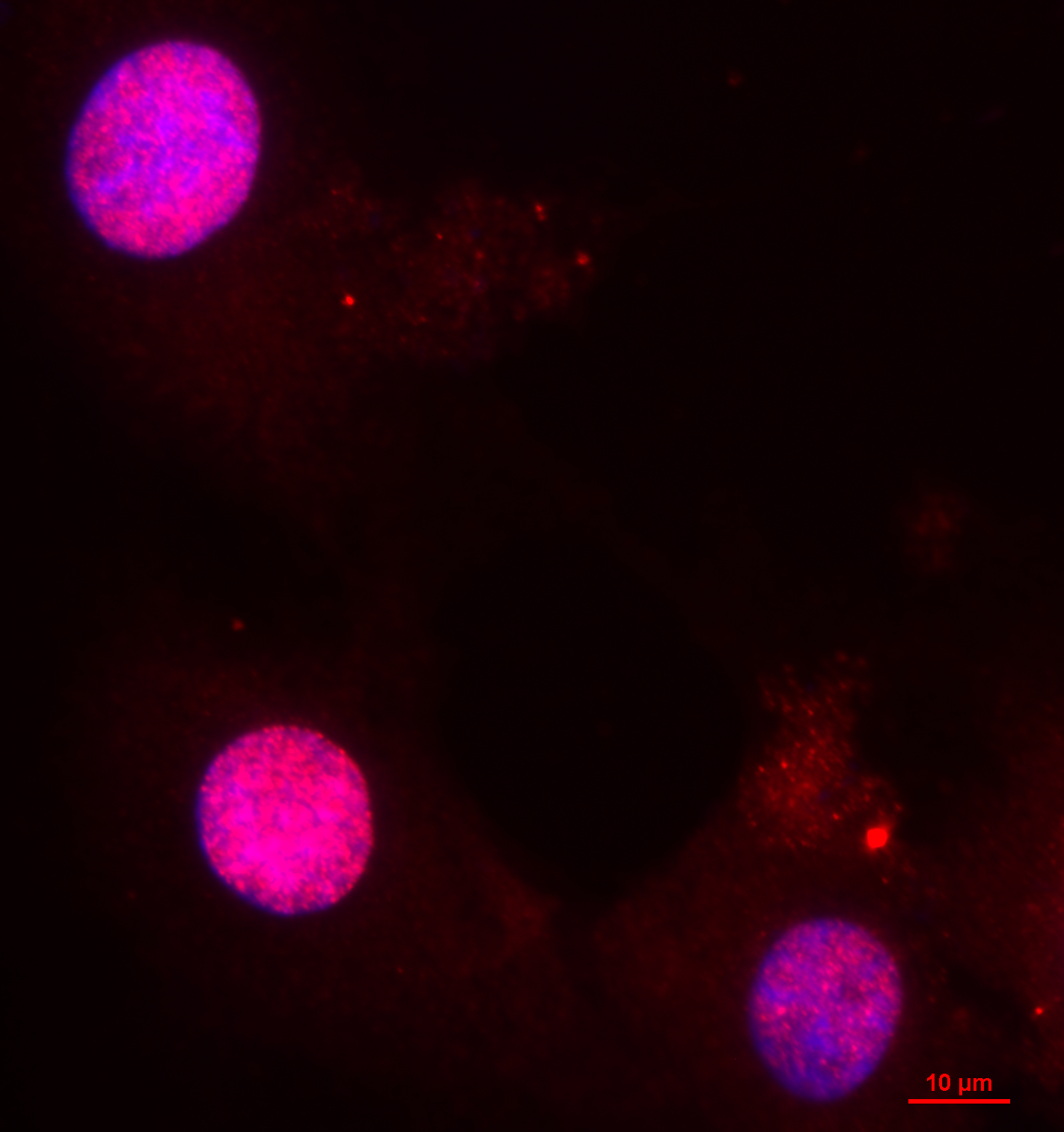

Supplement: Supplementary file 11 — Source Data EV Fig. 2 [file 44318_2023_3_MOESM11_ESM.zip › Figure EV2/2b-c/YTHDC1-WT ki67 IF.tif]

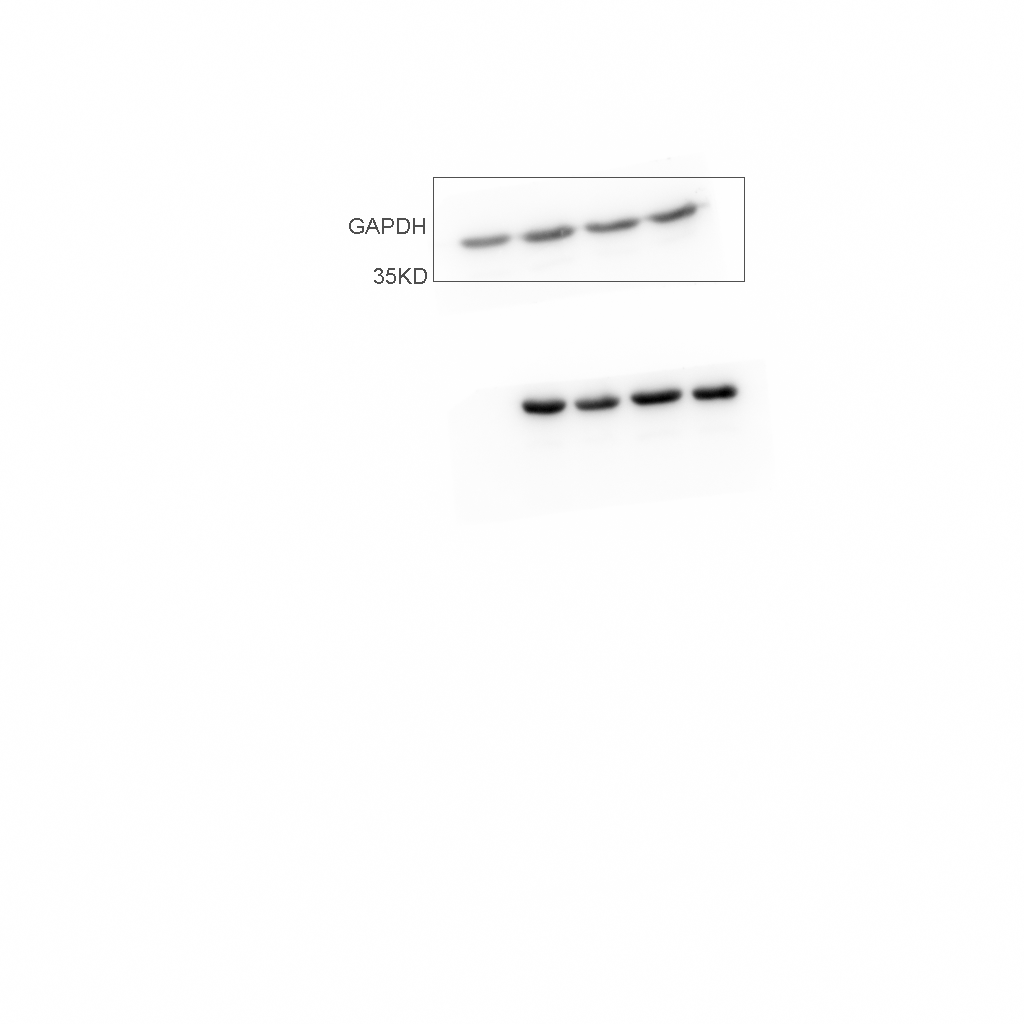

Supplement: Supplementary file 11 — Source Data EV Fig. 2 [file 44318_2023_3_MOESM11_ESM.zip › Figure EV2/2d/GAPDH NC siDC1 siM3 siDC1+siM3 .tif]

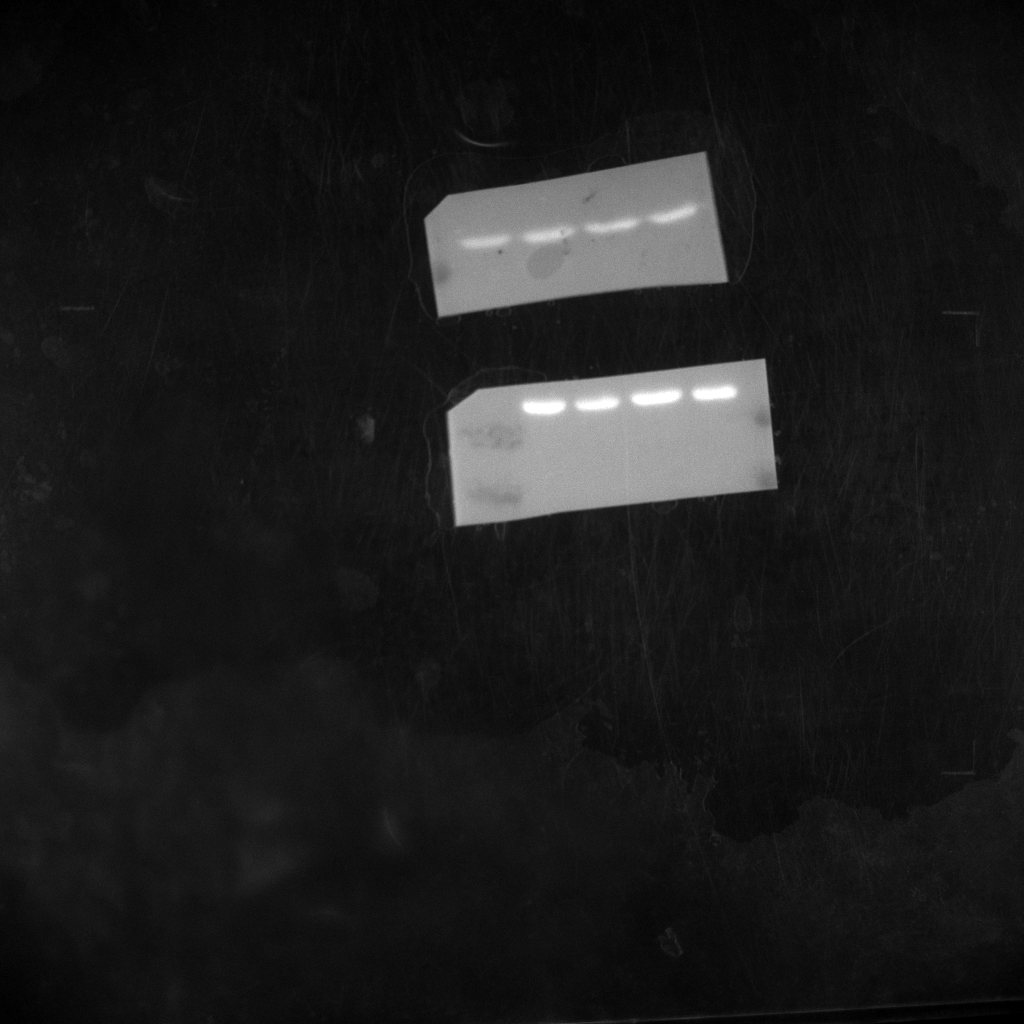

Supplement: Supplementary file 11 — Source Data EV Fig. 2 [file 44318_2023_3_MOESM11_ESM.zip › Figure EV2/2d/GAPDH NC siDC1 siM3 siDC1+siM3 w.tif]

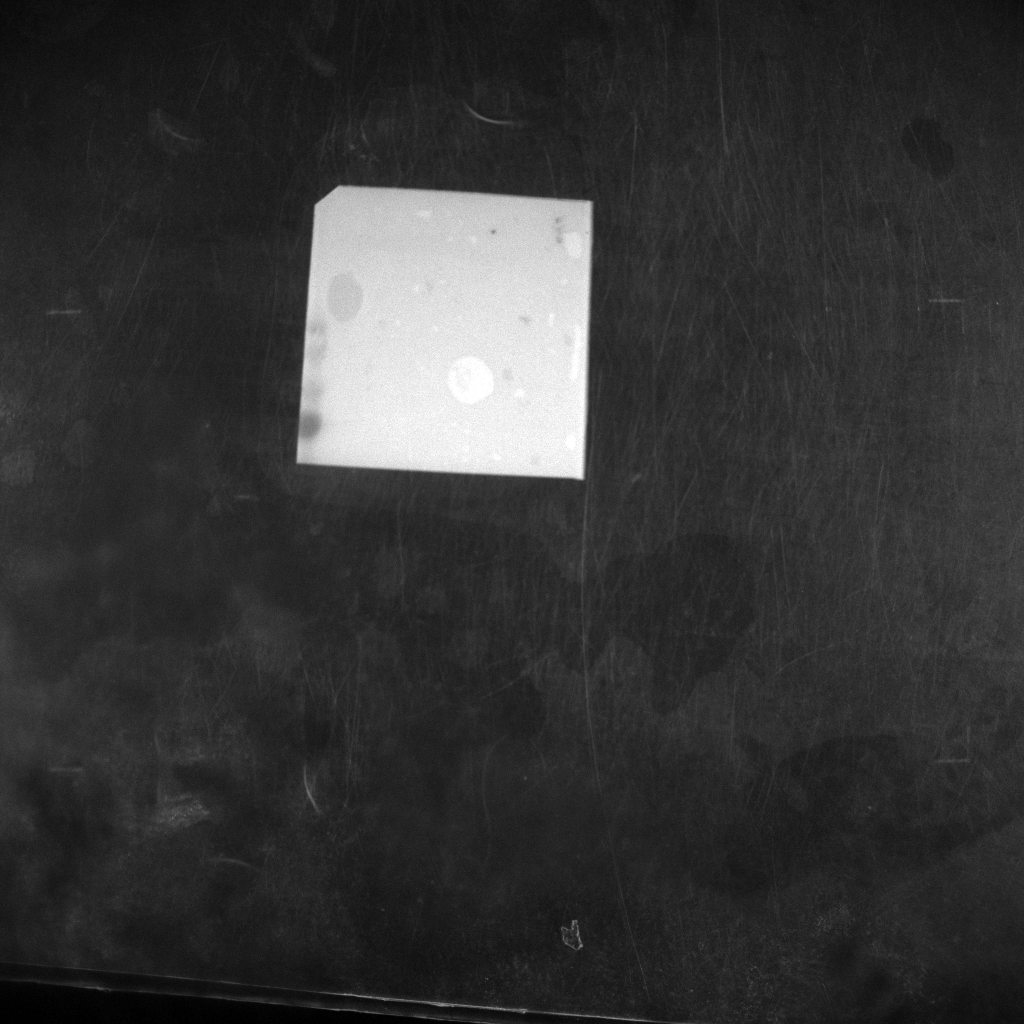

Supplement: Supplementary file 11 — Source Data EV Fig. 2 [file 44318_2023_3_MOESM11_ESM.zip › Figure EV2/2d/METTL3 NC siDC1 siM3 siDC1+siM3 1 W.tif]

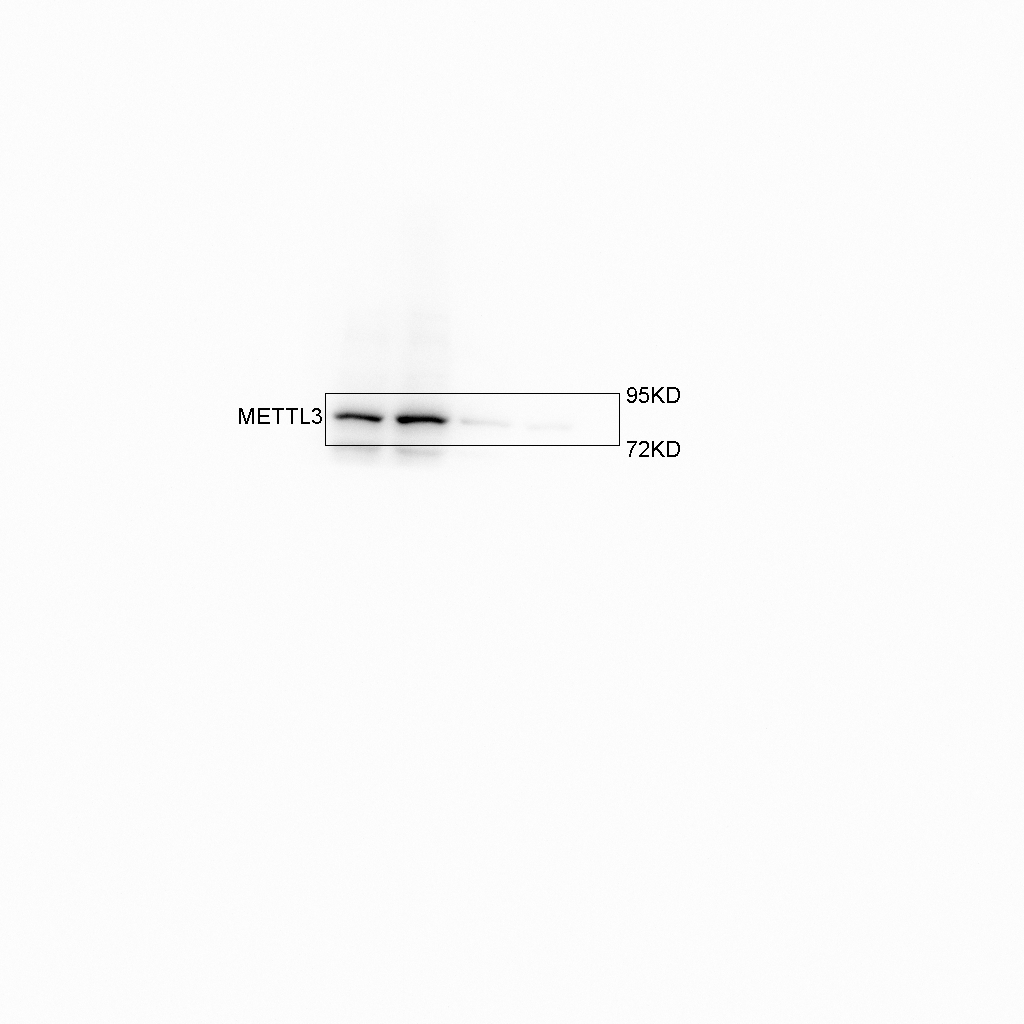

Supplement: Supplementary file 11 — Source Data EV Fig. 2 [file 44318_2023_3_MOESM11_ESM.zip › Figure EV2/2d/METTL3 NC siDC1 siM3 siDC1+siM3 1.tif]

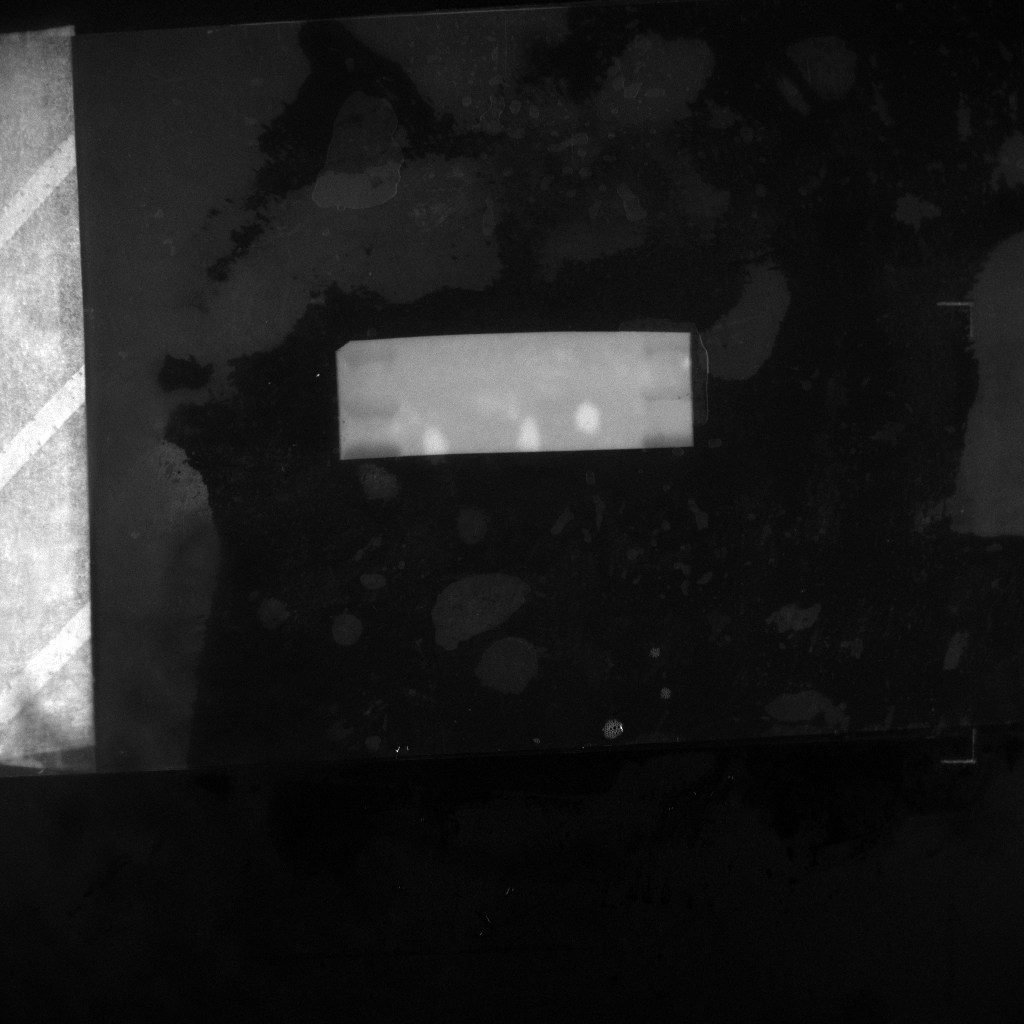

Supplement: Supplementary file 11 — Source Data EV Fig. 2 [file 44318_2023_3_MOESM11_ESM.zip › Figure EV2/2d/YTHDC1 NC siDC1 siM3 siDC1+siM3 W.tif]

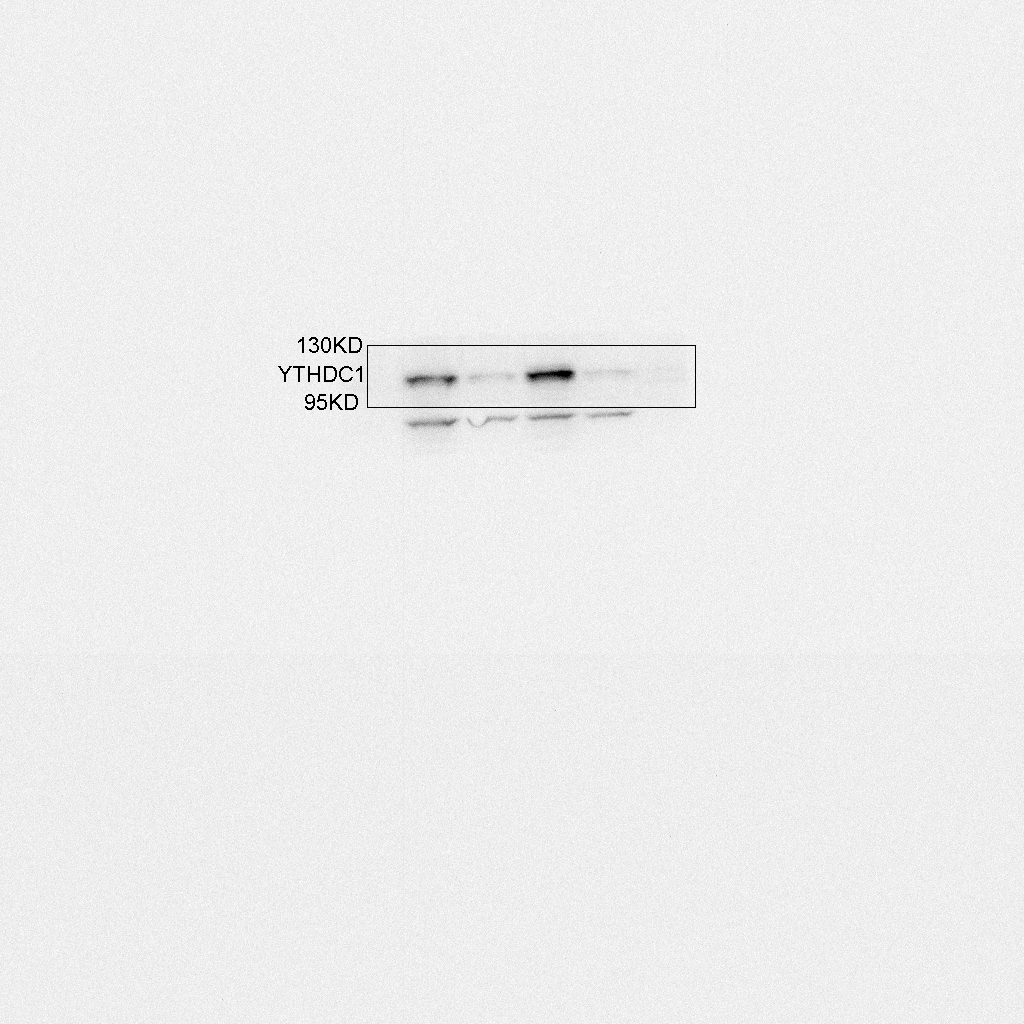

Supplement: Supplementary file 11 — Source Data EV Fig. 2 [file 44318_2023_3_MOESM11_ESM.zip › Figure EV2/2d/YTHDC1 NC siDC1 siM3 siDC1+siM3.tif]

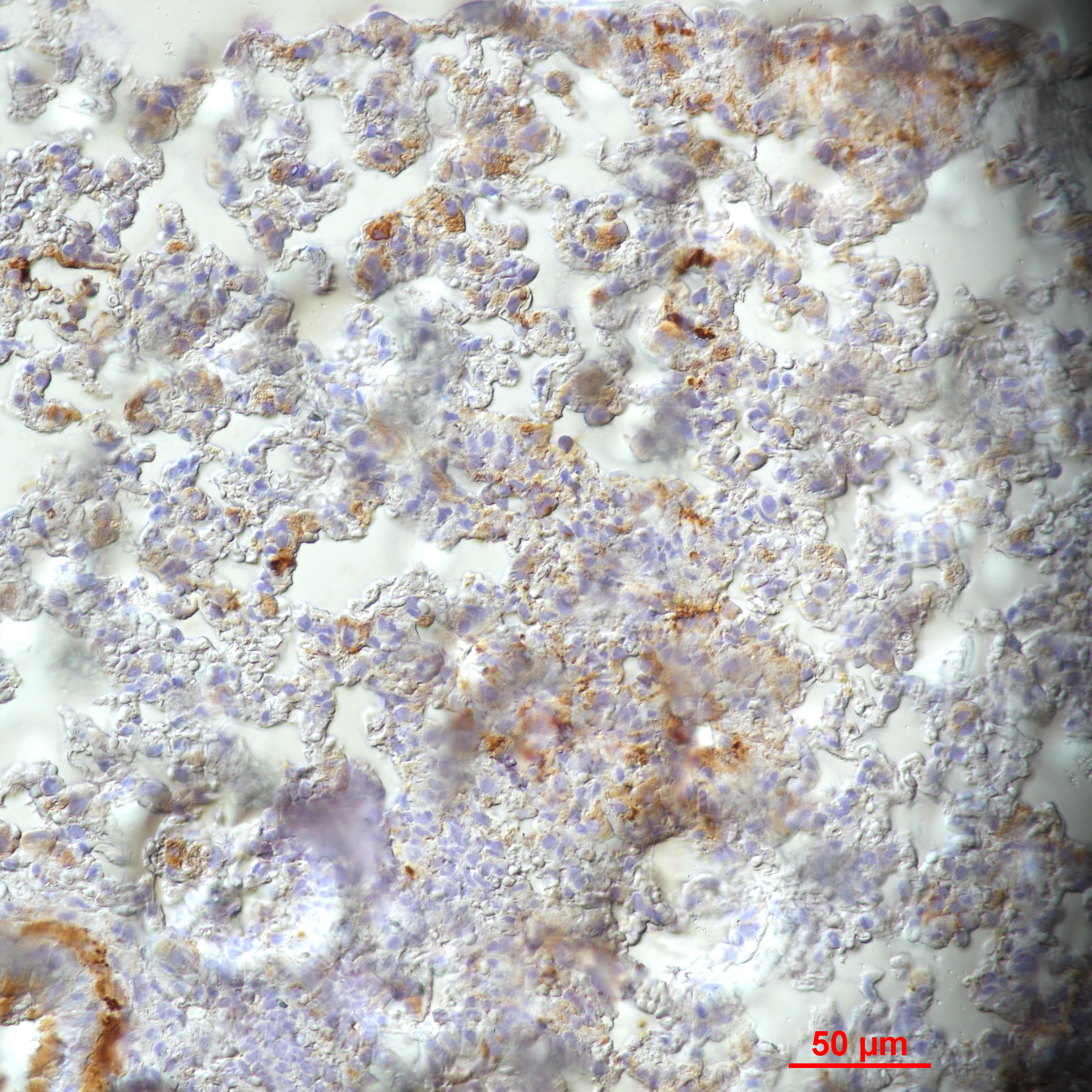

Supplement: Supplementary file 11 — Source Data EV Fig. 2 [file 44318_2023_3_MOESM11_ESM.zip › Figure EV2/2f-g/Vector a-SMA IHC.tif]

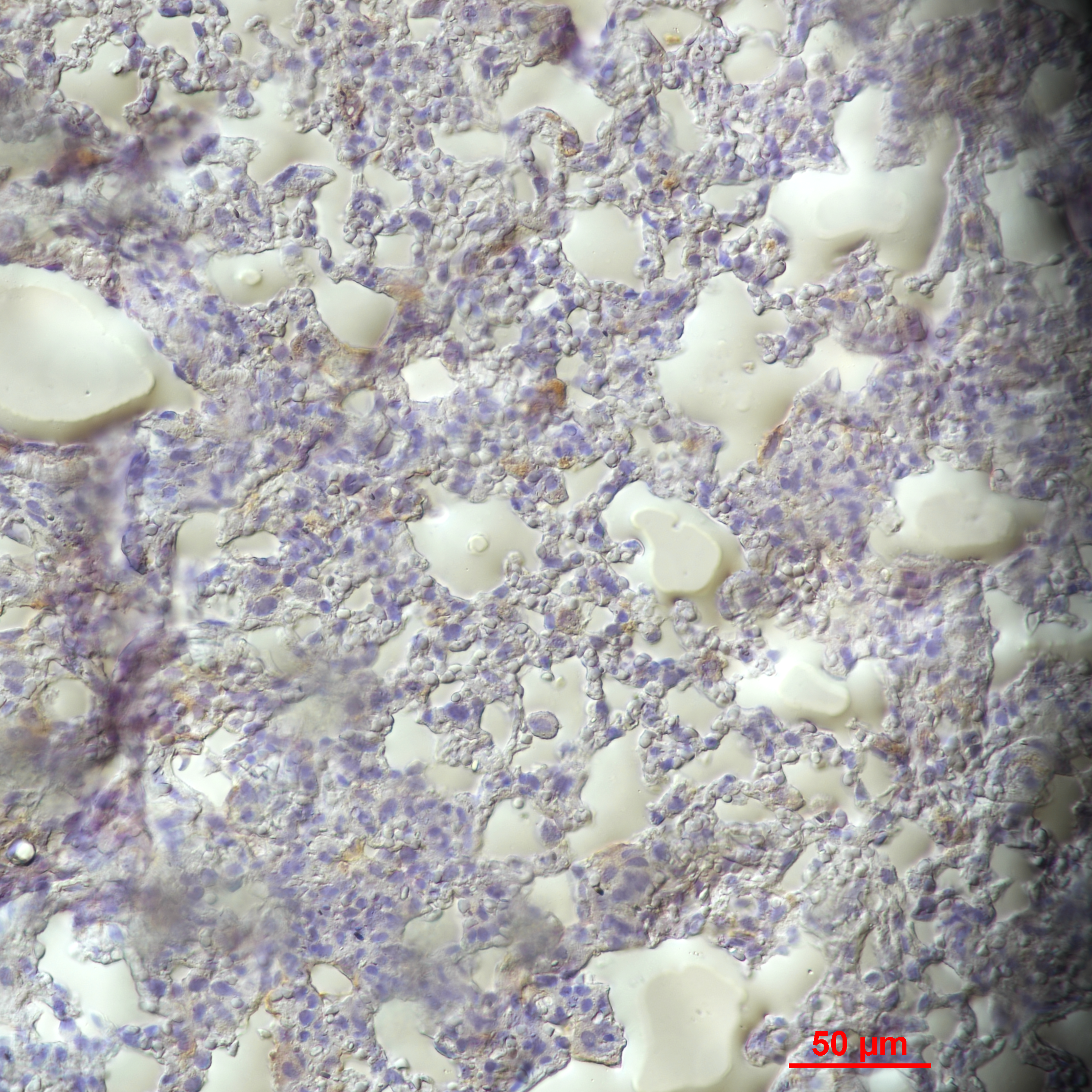

Supplement: Supplementary file 11 — Source Data EV Fig. 2 [file 44318_2023_3_MOESM11_ESM.zip › Figure EV2/2f-g/YTHDC1-MUT a-SMA IHC.tif]

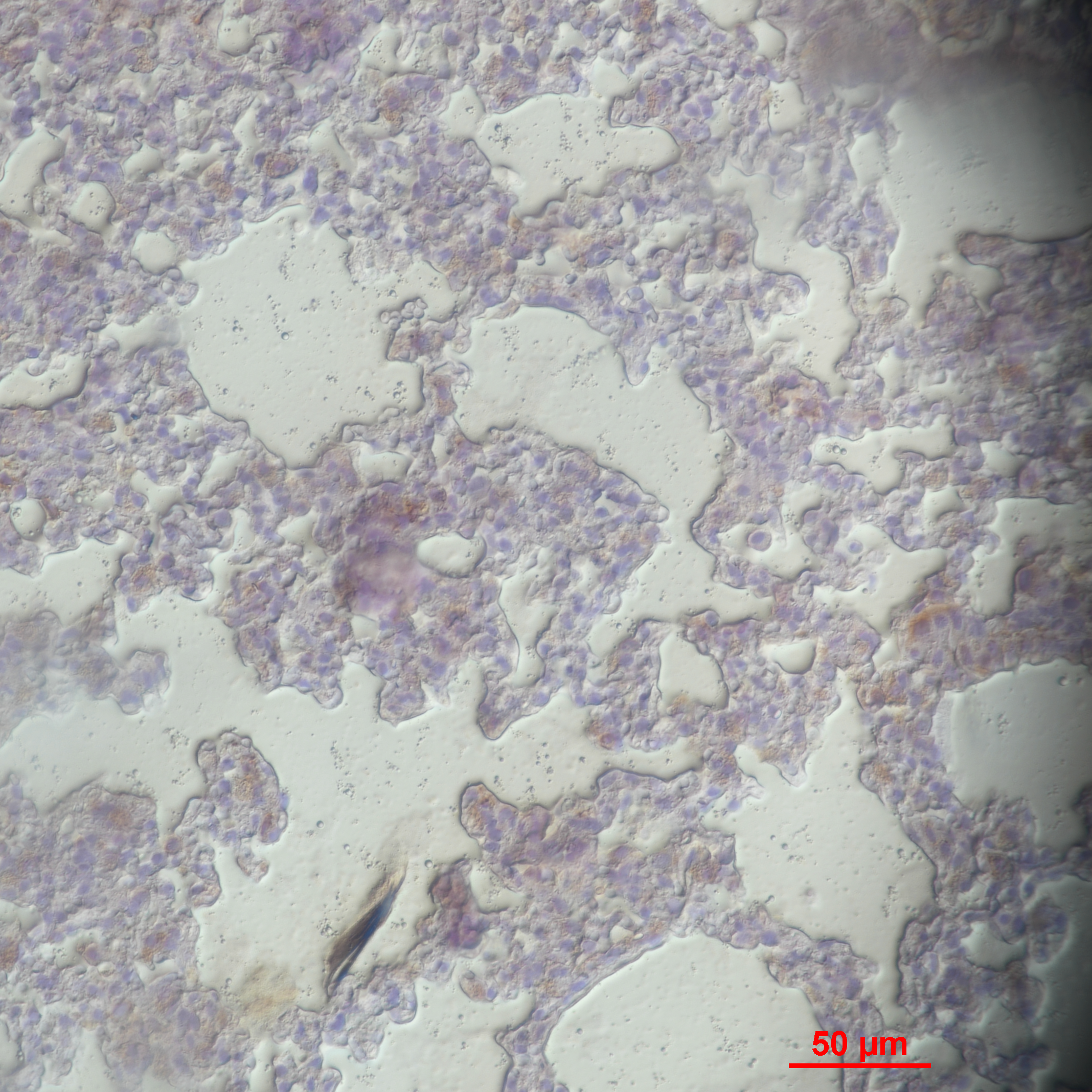

Supplement: Supplementary file 11 — Source Data EV Fig. 2 [file 44318_2023_3_MOESM11_ESM.zip › Figure EV2/2f-g/YTHDC1-WT a-SMA IHC.tif]

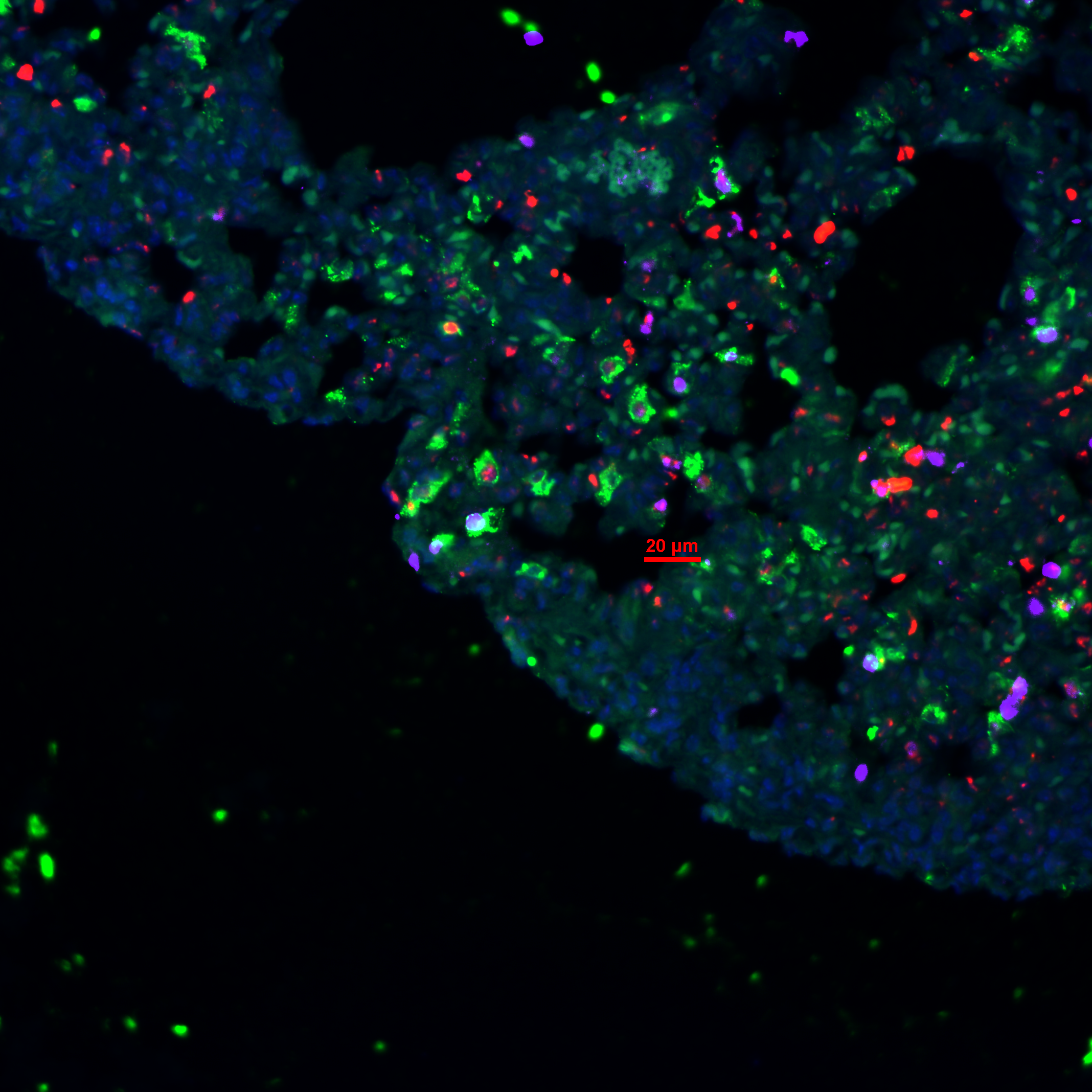

Supplement: Supplementary file 11 — Source Data EV Fig. 2 [file 44318_2023_3_MOESM11_ESM.zip › Figure EV2/2h-j/Vector YTHDC1, p21 and SPC IF/merge.tif]

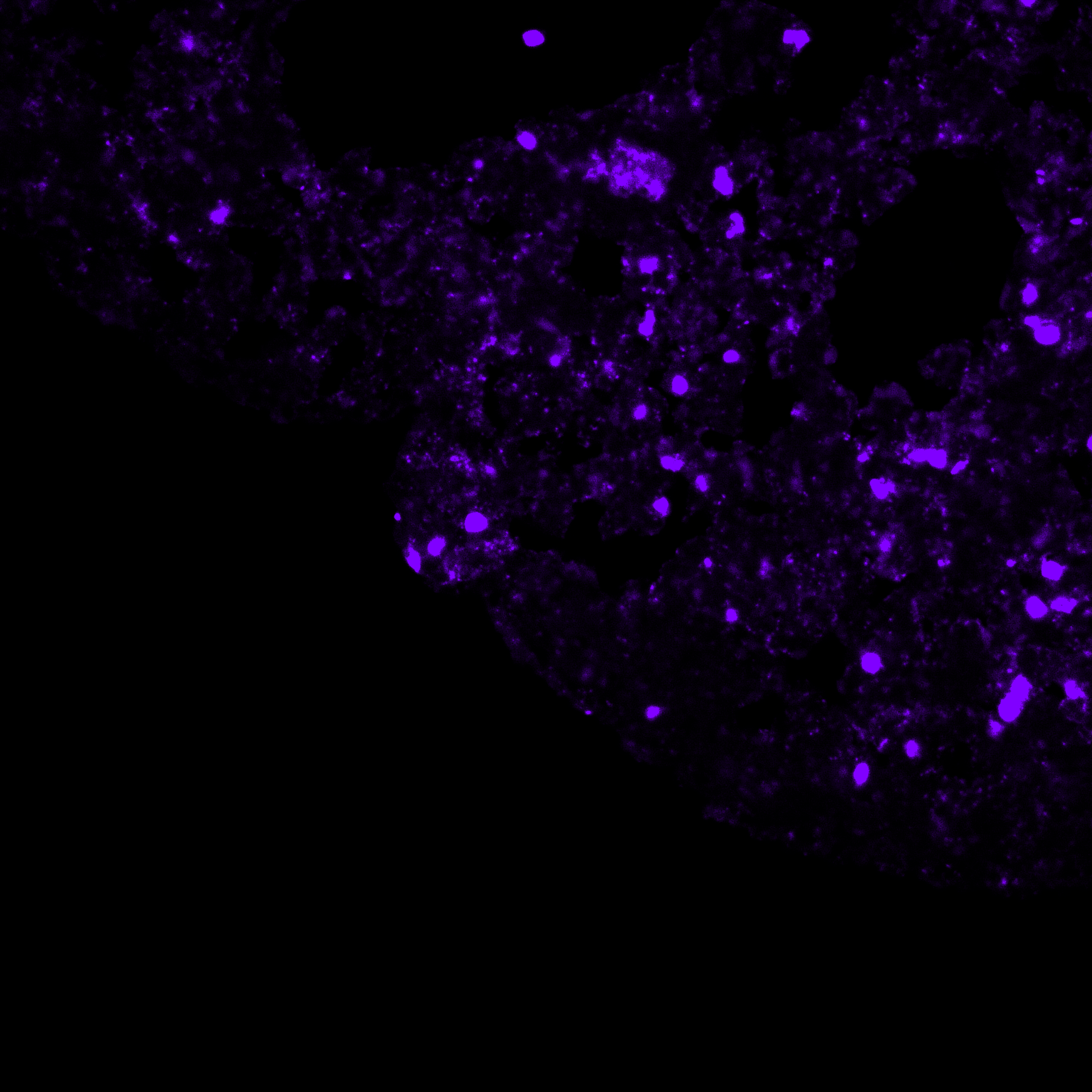

Supplement: Supplementary file 11 — Source Data EV Fig. 2 [file 44318_2023_3_MOESM11_ESM.zip › Figure EV2/2h-j/Vector YTHDC1, p21 and SPC IF/P21.tif]

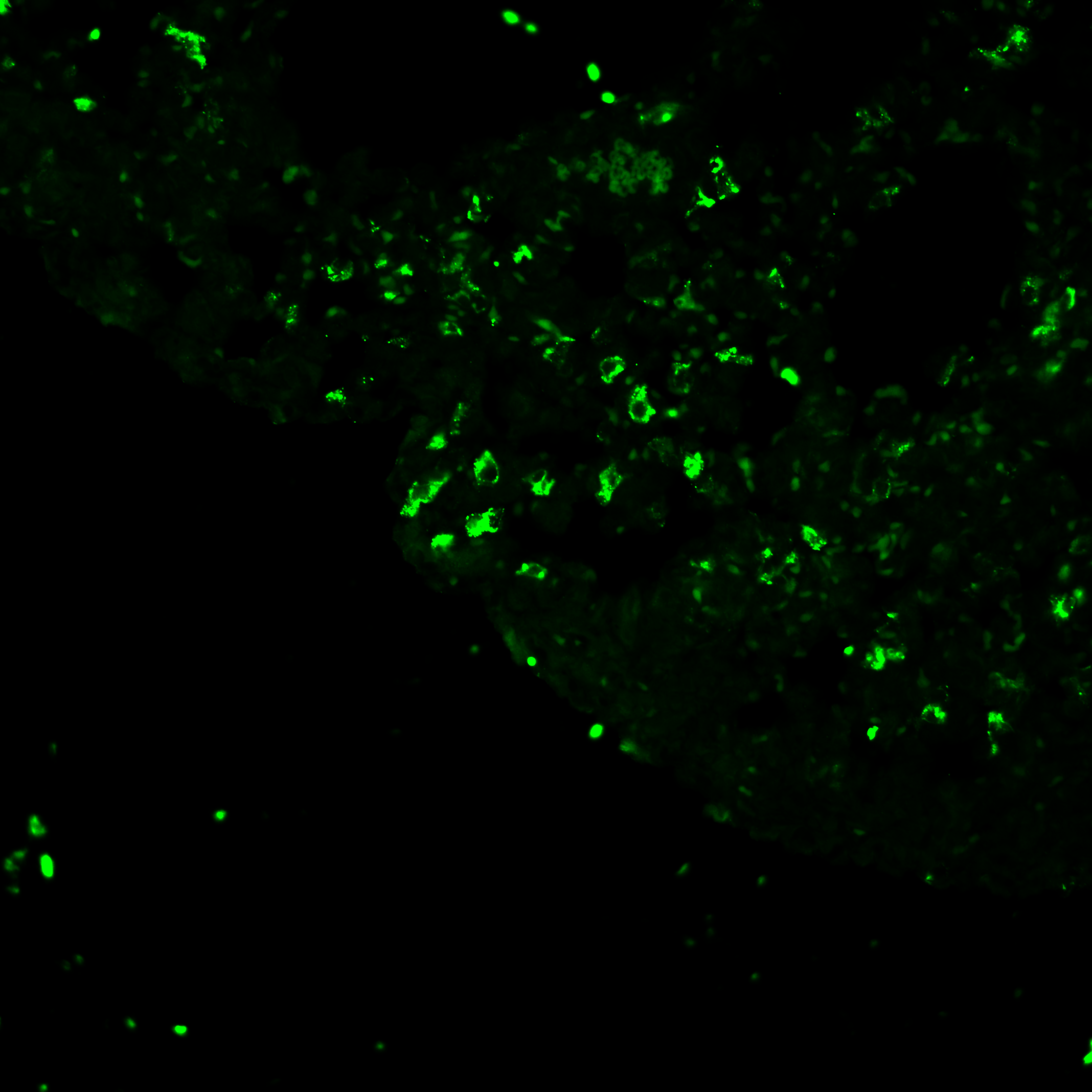

Supplement: Supplementary file 11 — Source Data EV Fig. 2 [file 44318_2023_3_MOESM11_ESM.zip › Figure EV2/2h-j/Vector YTHDC1, p21 and SPC IF/SPC.tif]

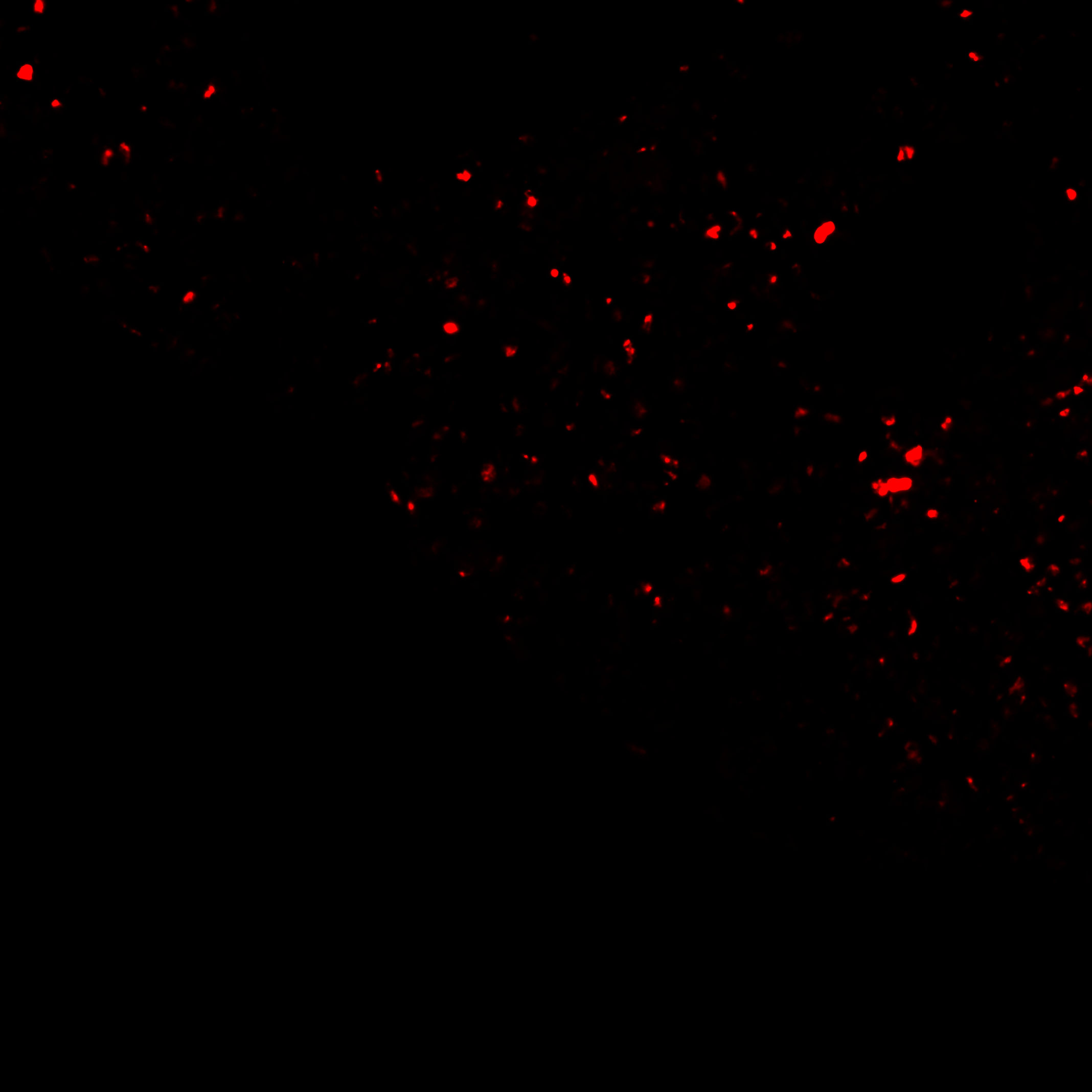

Supplement: Supplementary file 11 — Source Data EV Fig. 2 [file 44318_2023_3_MOESM11_ESM.zip › Figure EV2/2h-j/Vector YTHDC1, p21 and SPC IF/YTHDC1.tif]

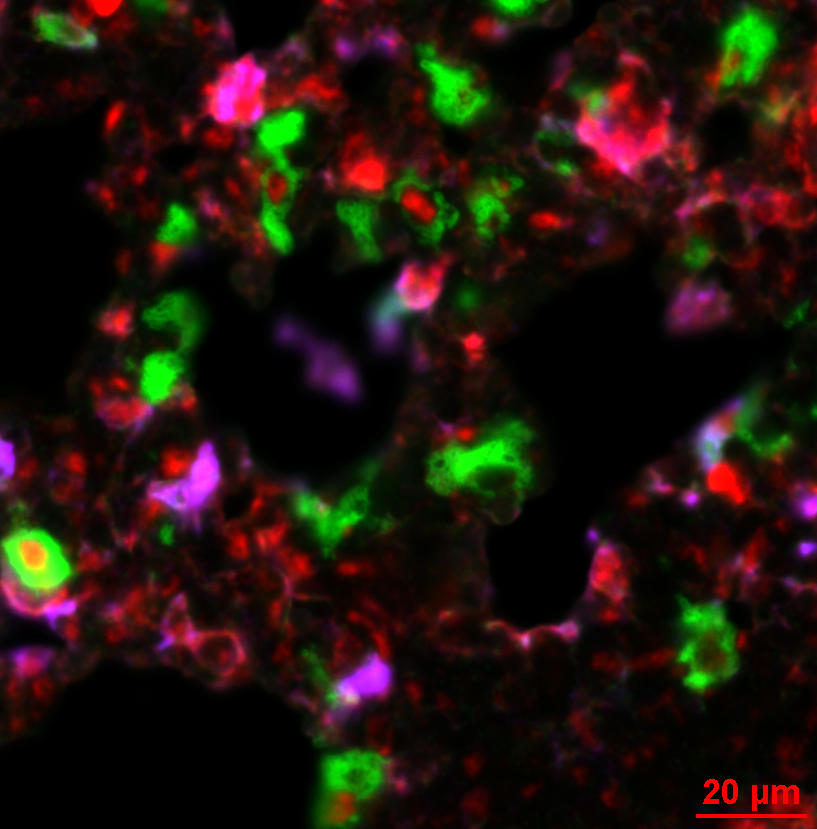

Supplement: Supplementary file 11 — Source Data EV Fig. 2 [file 44318_2023_3_MOESM11_ESM.zip › Figure EV2/2h-j/YTHDC1-MUT YTHDC1, p21 and SPC IF/2_mut 002_crop_RGB.tif]

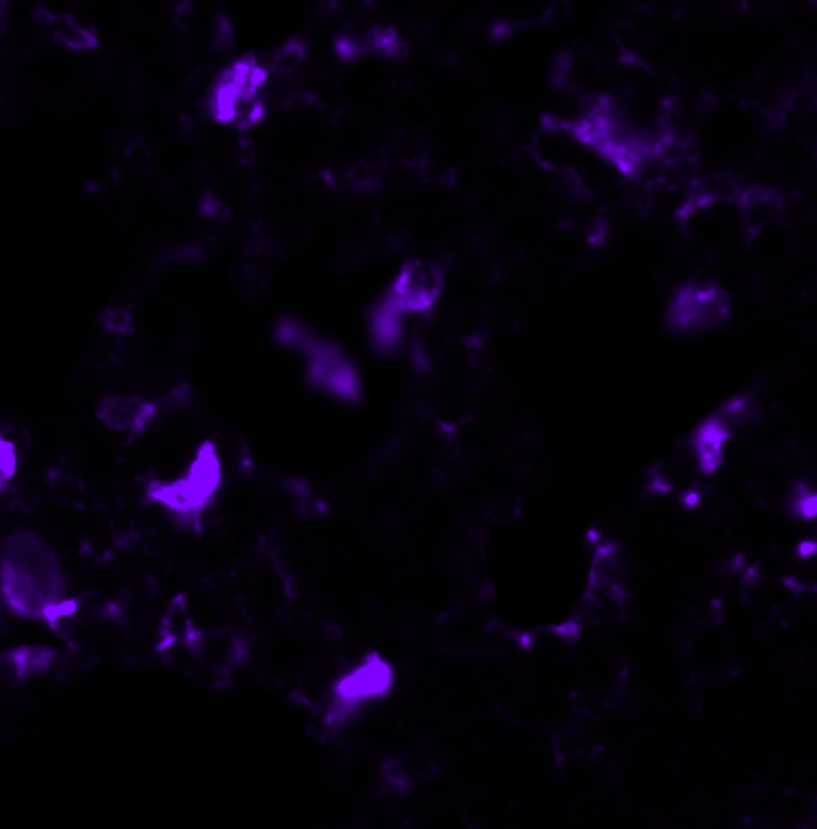

Supplement: Supplementary file 11 — Source Data EV Fig. 2 [file 44318_2023_3_MOESM11_ESM.zip › Figure EV2/2h-j/YTHDC1-MUT YTHDC1, p21 and SPC IF/p21.tif]

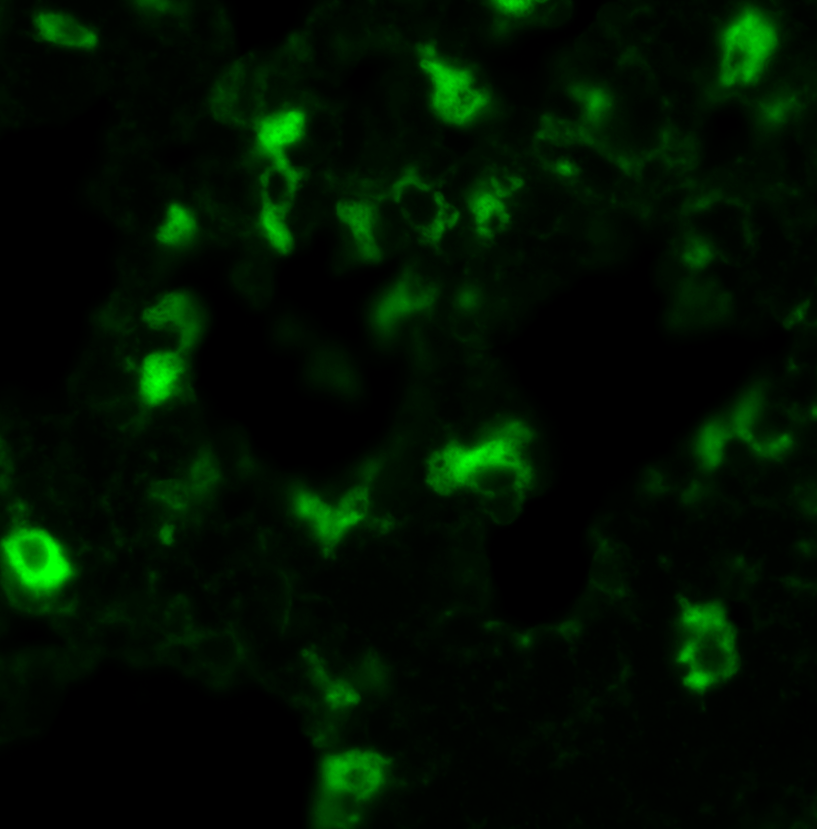

Supplement: Supplementary file 11 — Source Data EV Fig. 2 [file 44318_2023_3_MOESM11_ESM.zip › Figure EV2/2h-j/YTHDC1-MUT YTHDC1, p21 and SPC IF/SPC.tif]

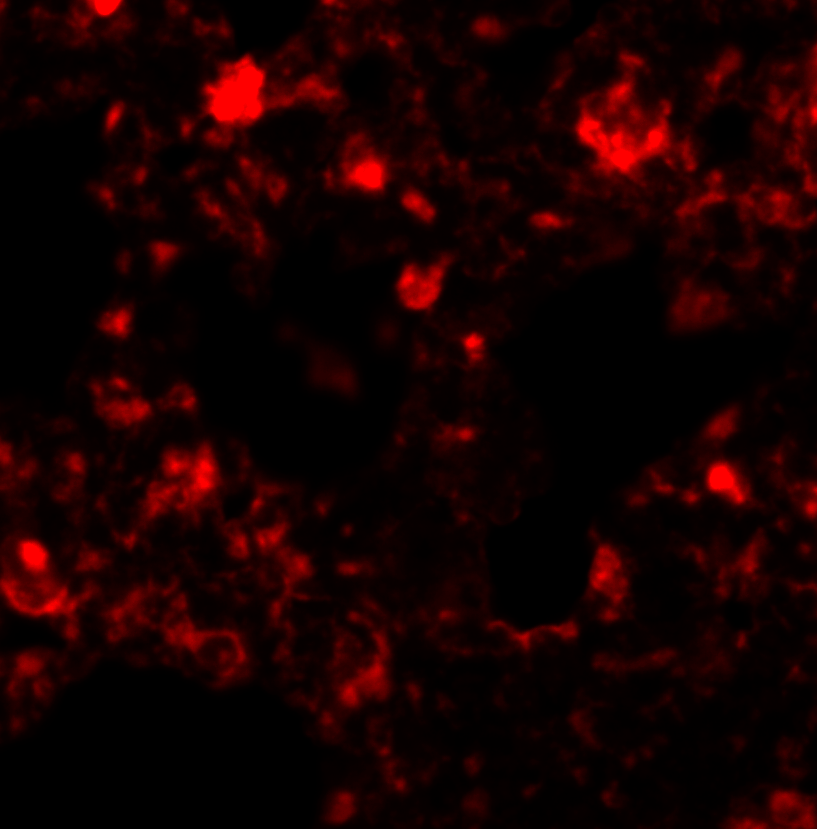

Supplement: Supplementary file 11 — Source Data EV Fig. 2 [file 44318_2023_3_MOESM11_ESM.zip › Figure EV2/2h-j/YTHDC1-MUT YTHDC1, p21 and SPC IF/YTHDC1.tif]

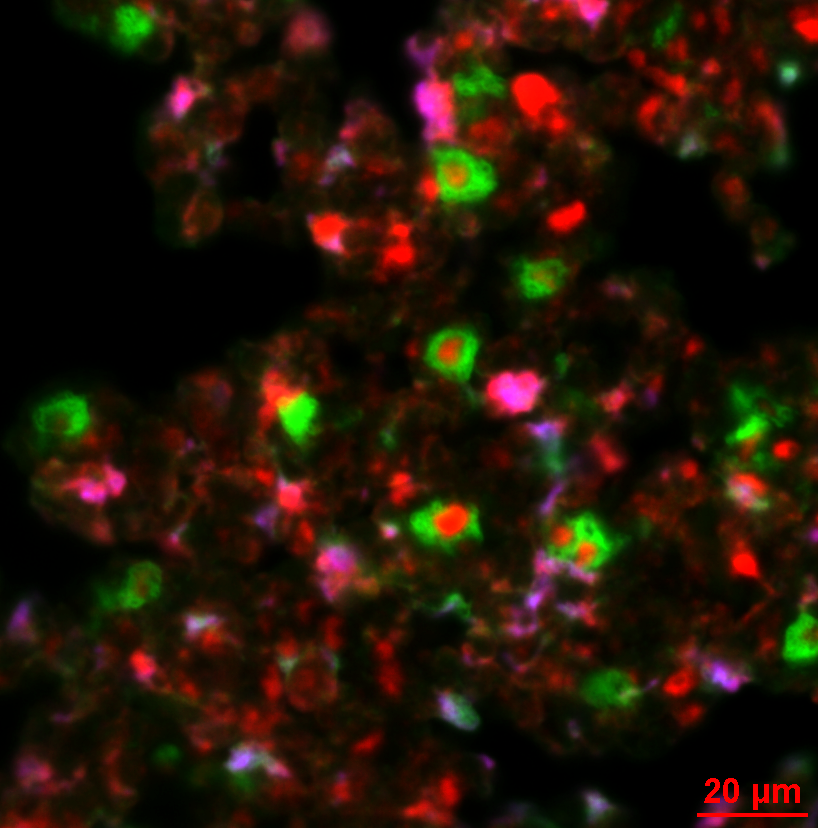

Supplement: Supplementary file 11 — Source Data EV Fig. 2 [file 44318_2023_3_MOESM11_ESM.zip › Figure EV2/2h-j/YTHDC1-WT YTHDC1, p21 and SPC IF/merge.tif]

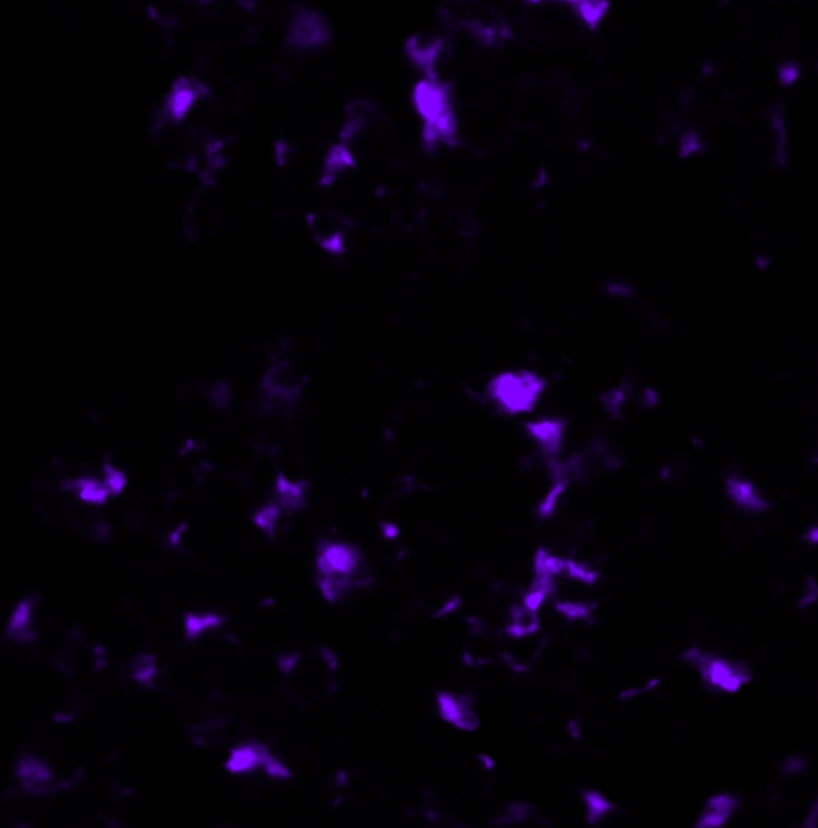

Supplement: Supplementary file 11 — Source Data EV Fig. 2 [file 44318_2023_3_MOESM11_ESM.zip › Figure EV2/2h-j/YTHDC1-WT YTHDC1, p21 and SPC IF/p21.tif]

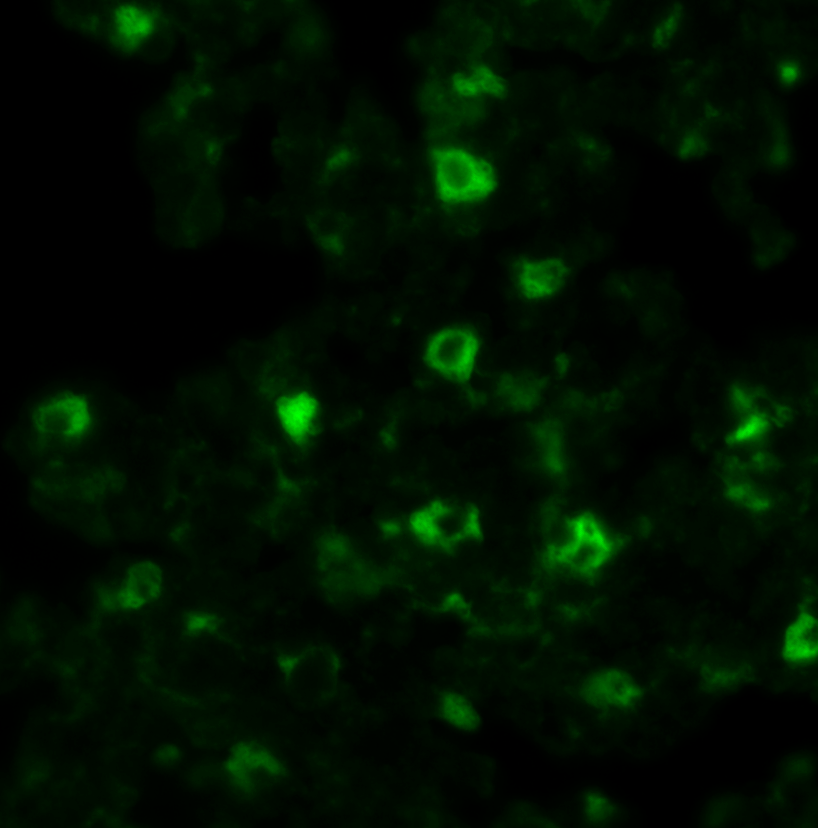

Supplement: Supplementary file 11 — Source Data EV Fig. 2 [file 44318_2023_3_MOESM11_ESM.zip › Figure EV2/2h-j/YTHDC1-WT YTHDC1, p21 and SPC IF/SPC.tif]

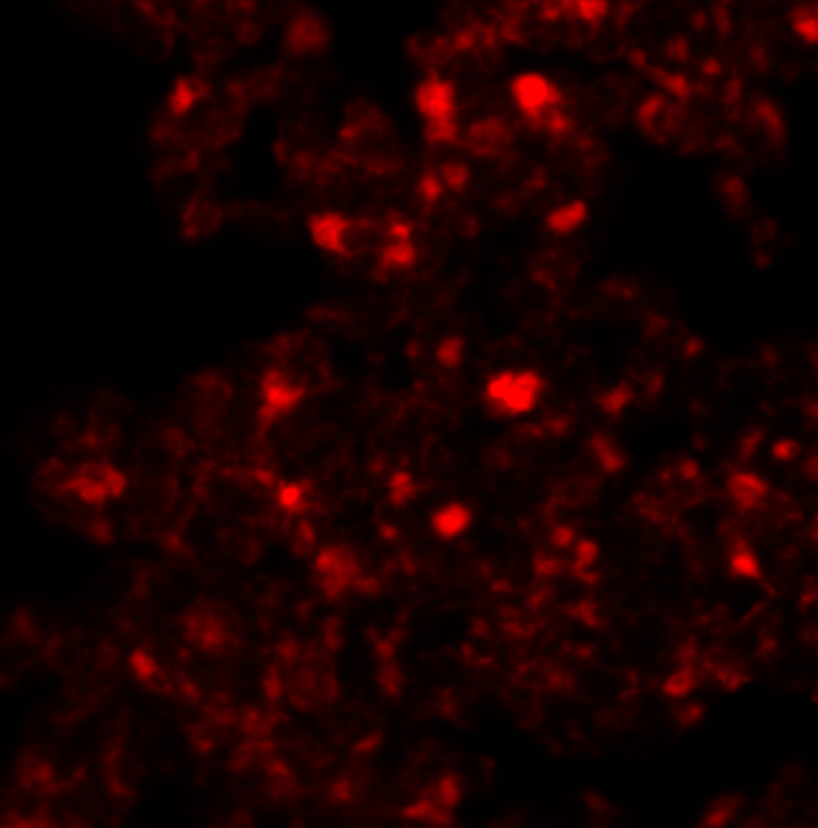

Supplement: Supplementary file 11 — Source Data EV Fig. 2 [file 44318_2023_3_MOESM11_ESM.zip › Figure EV2/2h-j/YTHDC1-WT YTHDC1, p21 and SPC IF/YTHDC1.tif]

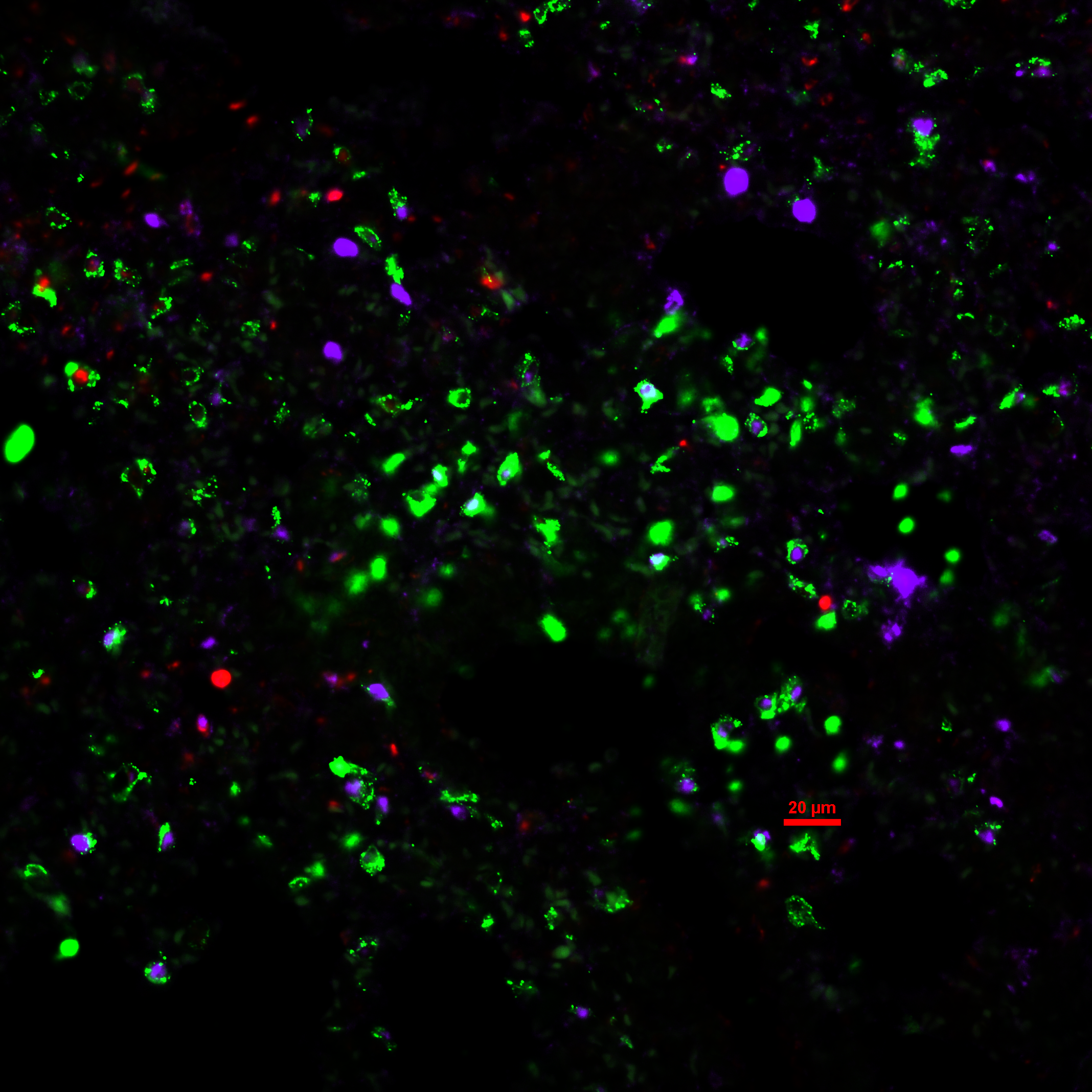

Supplement: Supplementary file 11 — Source Data EV Fig. 2 [file 44318_2023_3_MOESM11_ESM.zip › Figure EV2/2k-m/Vector YTHDC1, p16 and SPC IF/MERGE.tif]

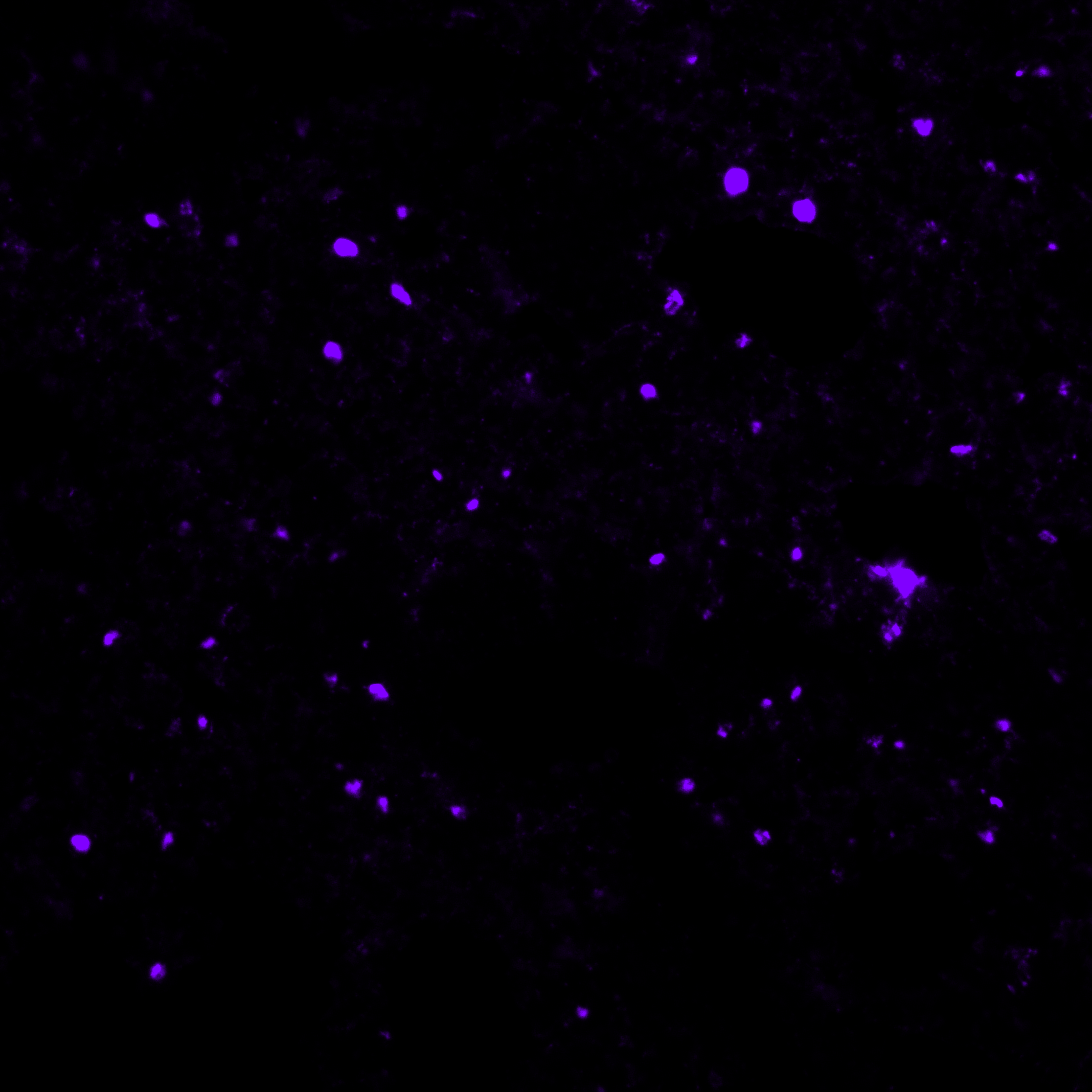

Supplement: Supplementary file 11 — Source Data EV Fig. 2 [file 44318_2023_3_MOESM11_ESM.zip › Figure EV2/2k-m/Vector YTHDC1, p16 and SPC IF/p16.tif]

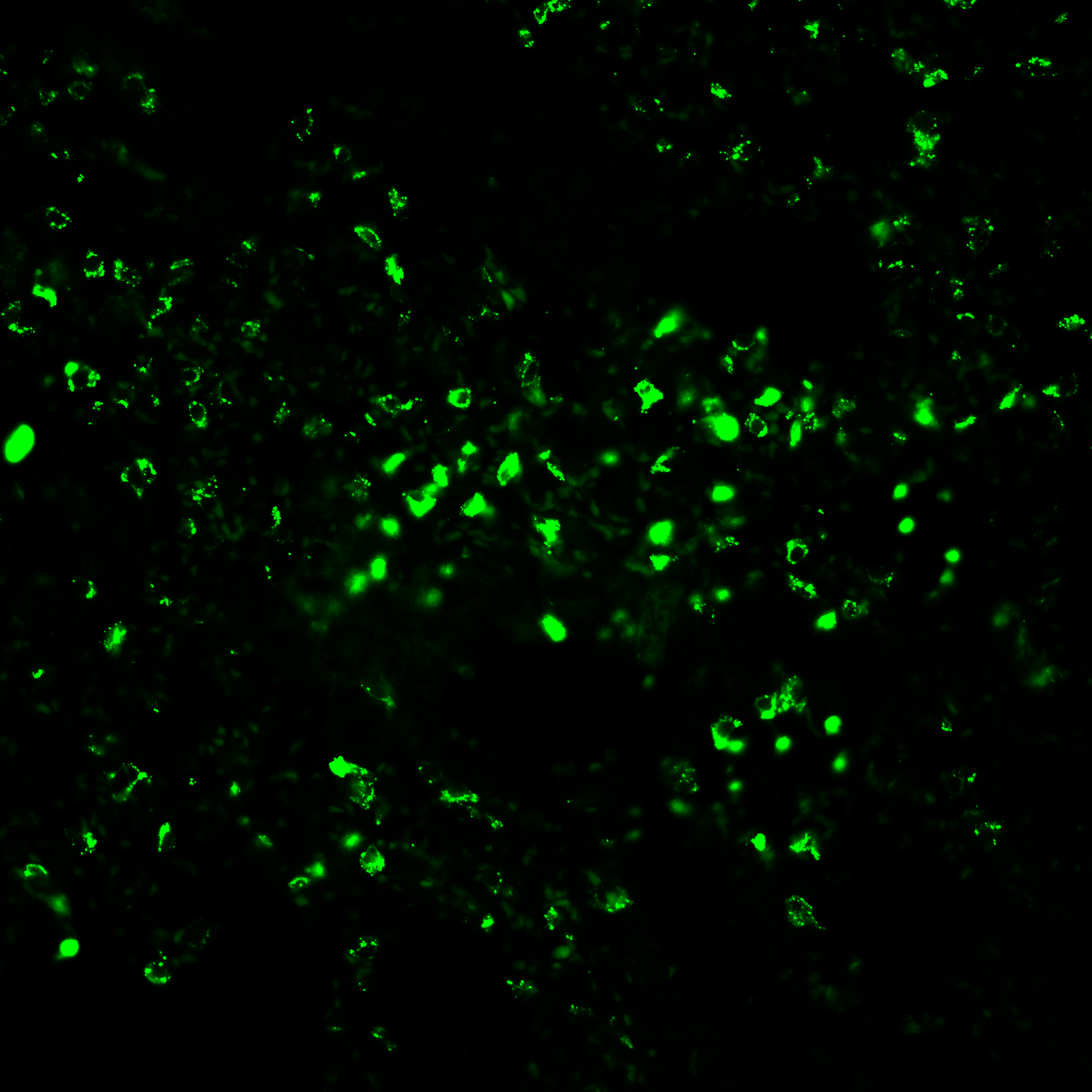

Supplement: Supplementary file 11 — Source Data EV Fig. 2 [file 44318_2023_3_MOESM11_ESM.zip › Figure EV2/2k-m/Vector YTHDC1, p16 and SPC IF/SPC.tif]

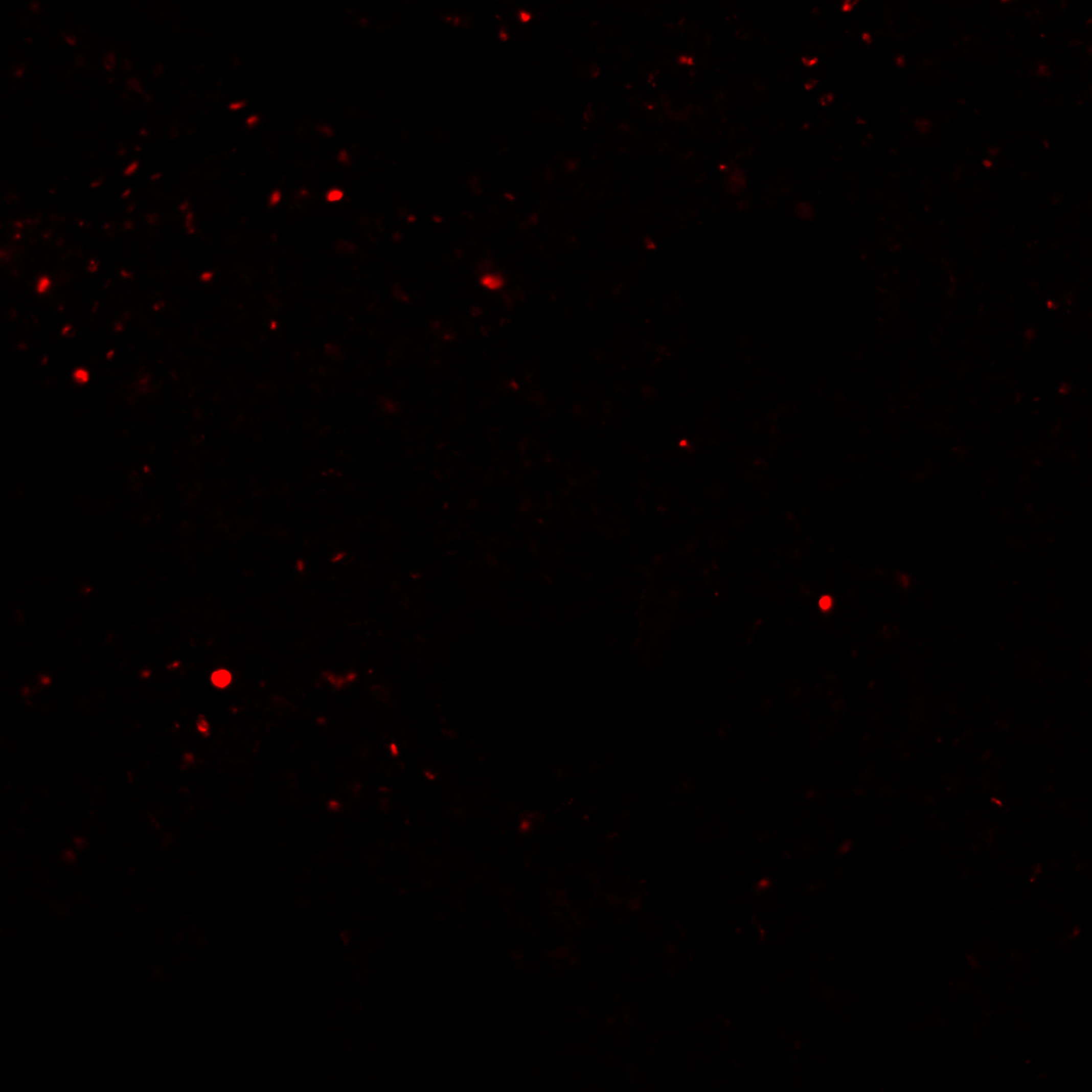

Supplement: Supplementary file 11 — Source Data EV Fig. 2 [file 44318_2023_3_MOESM11_ESM.zip › Figure EV2/2k-m/Vector YTHDC1, p16 and SPC IF/YTHDC1.tif]

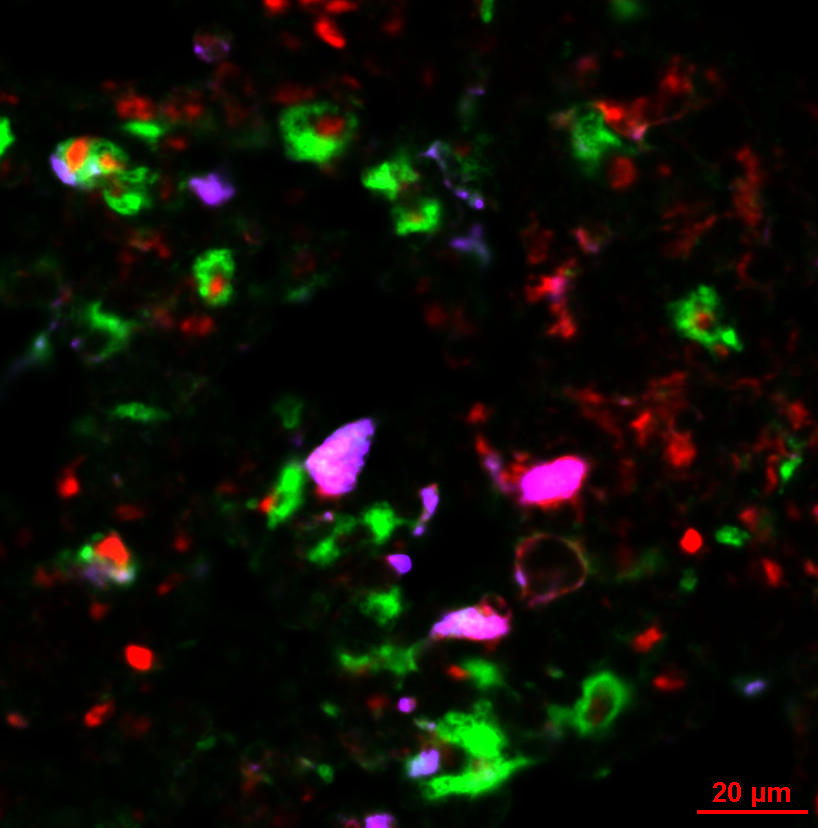

Supplement: Supplementary file 11 — Source Data EV Fig. 2 [file 44318_2023_3_MOESM11_ESM.zip › Figure EV2/2k-m/YTHDC1-MUT YTHDC1, p16 and SPC IF/merge.tif]

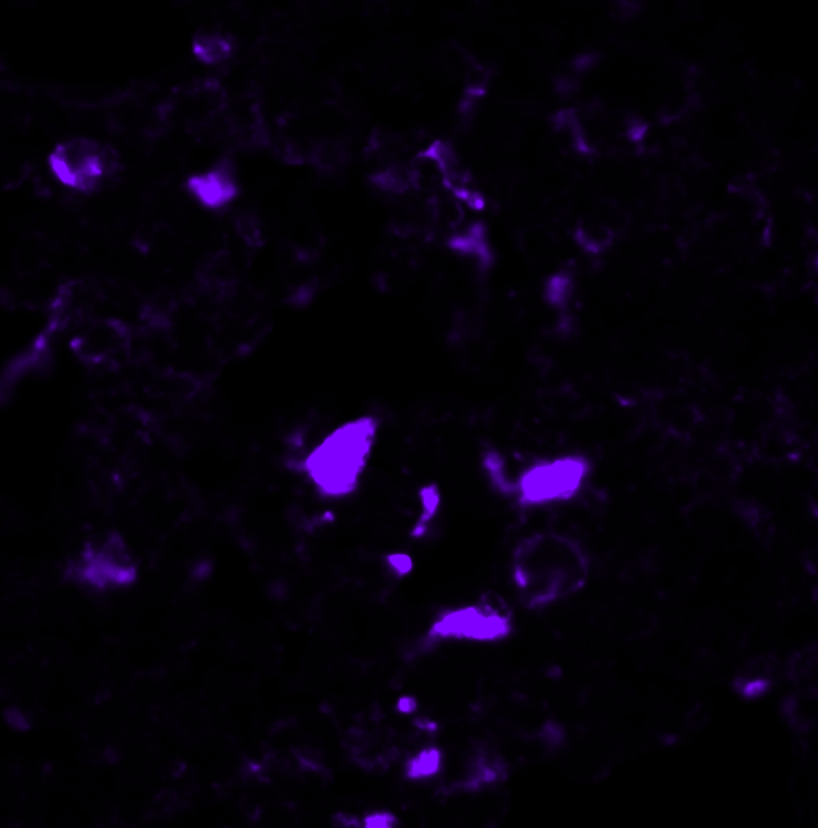

Supplement: Supplementary file 11 — Source Data EV Fig. 2 [file 44318_2023_3_MOESM11_ESM.zip › Figure EV2/2k-m/YTHDC1-MUT YTHDC1, p16 and SPC IF/p16.tif]

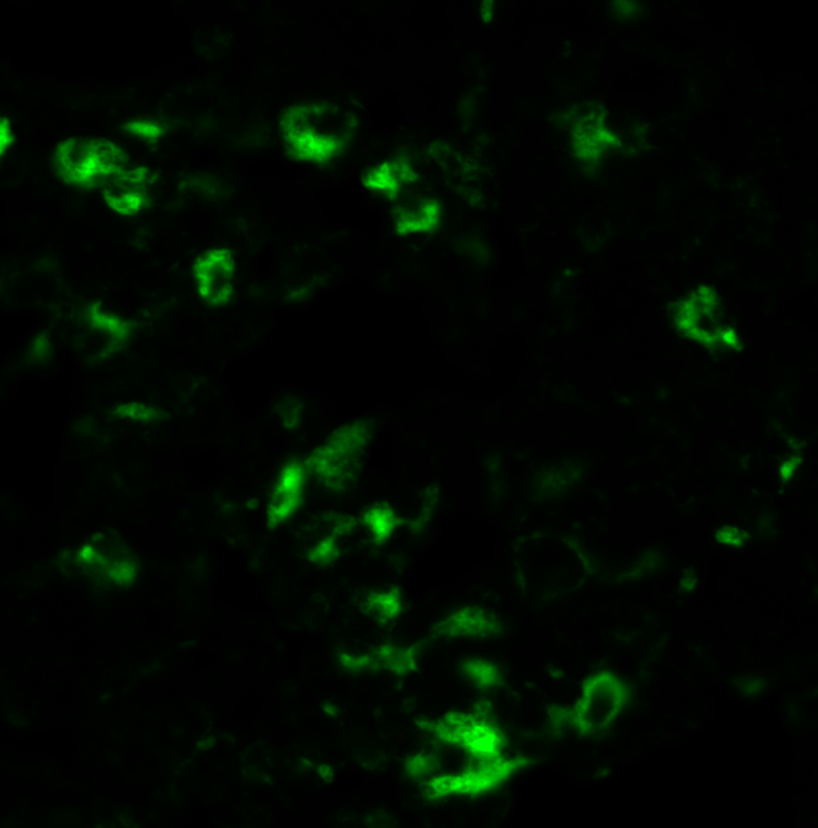

Supplement: Supplementary file 11 — Source Data EV Fig. 2 [file 44318_2023_3_MOESM11_ESM.zip › Figure EV2/2k-m/YTHDC1-MUT YTHDC1, p16 and SPC IF/SPC.tif]

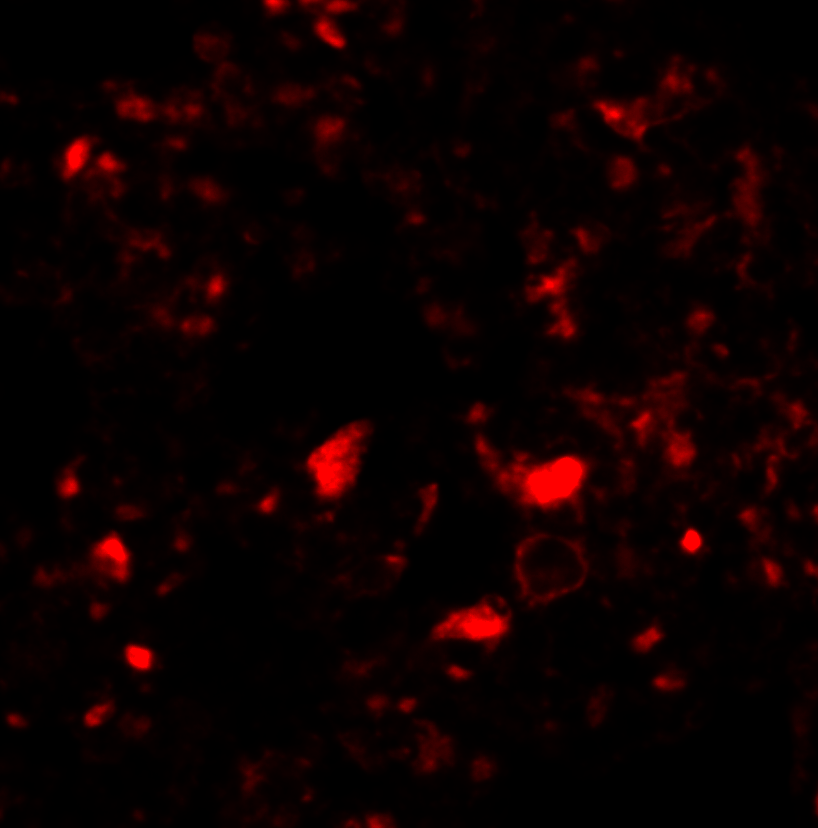

Supplement: Supplementary file 11 — Source Data EV Fig. 2 [file 44318_2023_3_MOESM11_ESM.zip › Figure EV2/2k-m/YTHDC1-MUT YTHDC1, p16 and SPC IF/YTHDC1.tif]

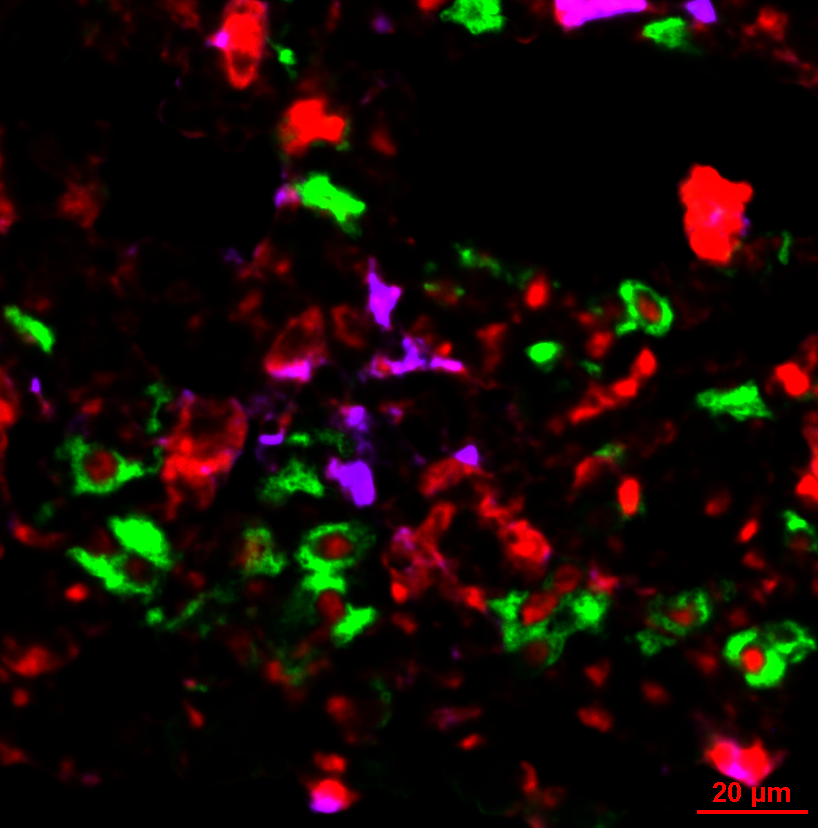

Supplement: Supplementary file 11 — Source Data EV Fig. 2 [file 44318_2023_3_MOESM11_ESM.zip › Figure EV2/2k-m/YTHDC1-WT YTHDC1, p16 and SPC IF/MERGE.tif]

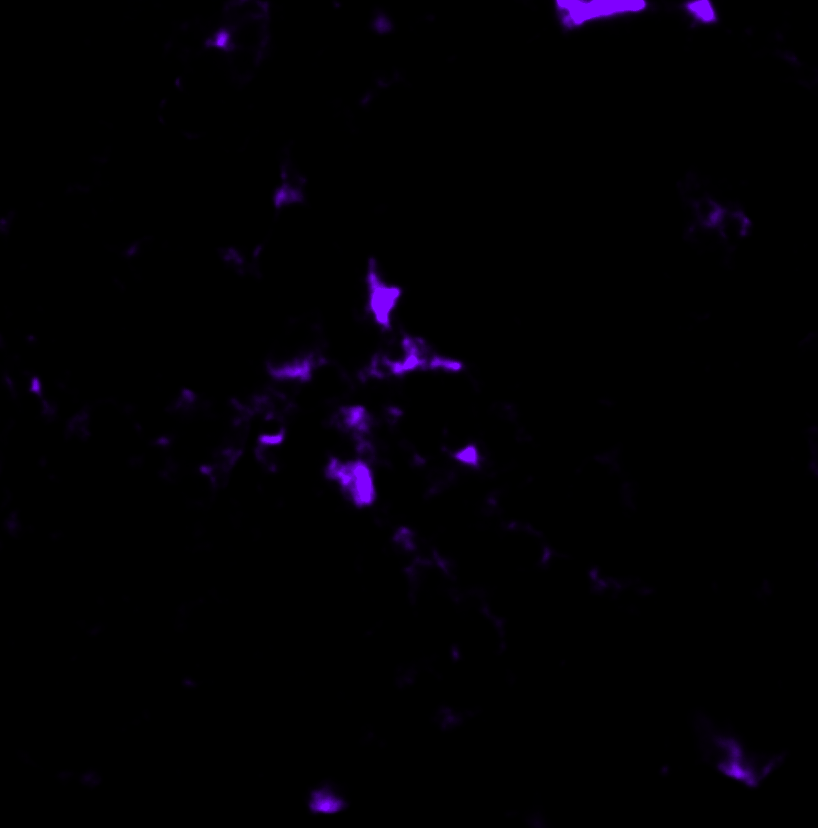

Supplement: Supplementary file 11 — Source Data EV Fig. 2 [file 44318_2023_3_MOESM11_ESM.zip › Figure EV2/2k-m/YTHDC1-WT YTHDC1, p16 and SPC IF/p16.tif]

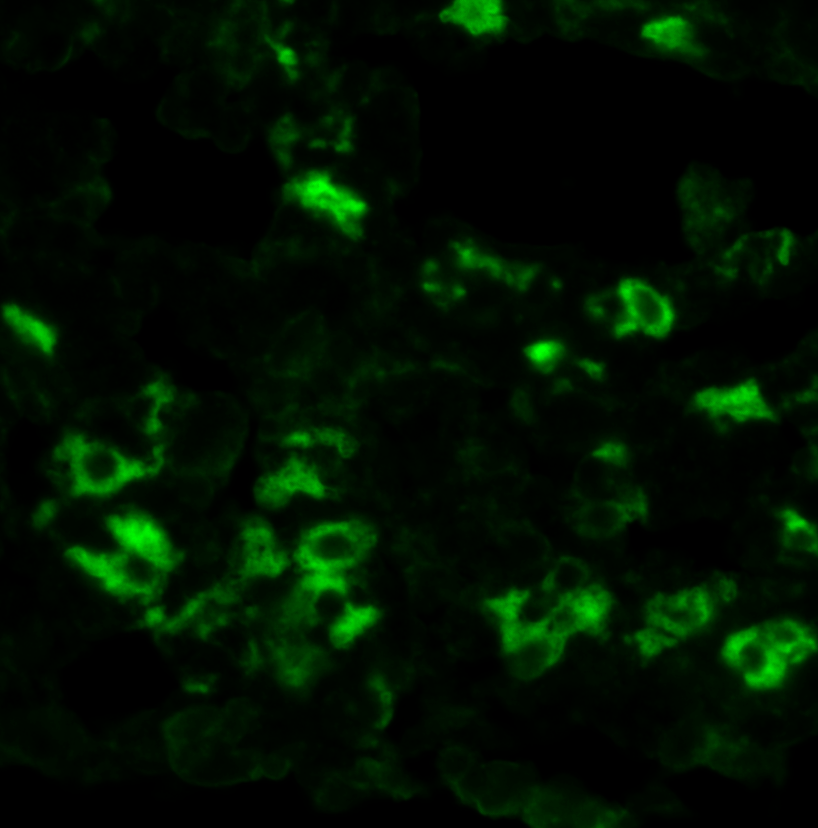

Supplement: Supplementary file 11 — Source Data EV Fig. 2 [file 44318_2023_3_MOESM11_ESM.zip › Figure EV2/2k-m/YTHDC1-WT YTHDC1, p16 and SPC IF/SPC.tif]

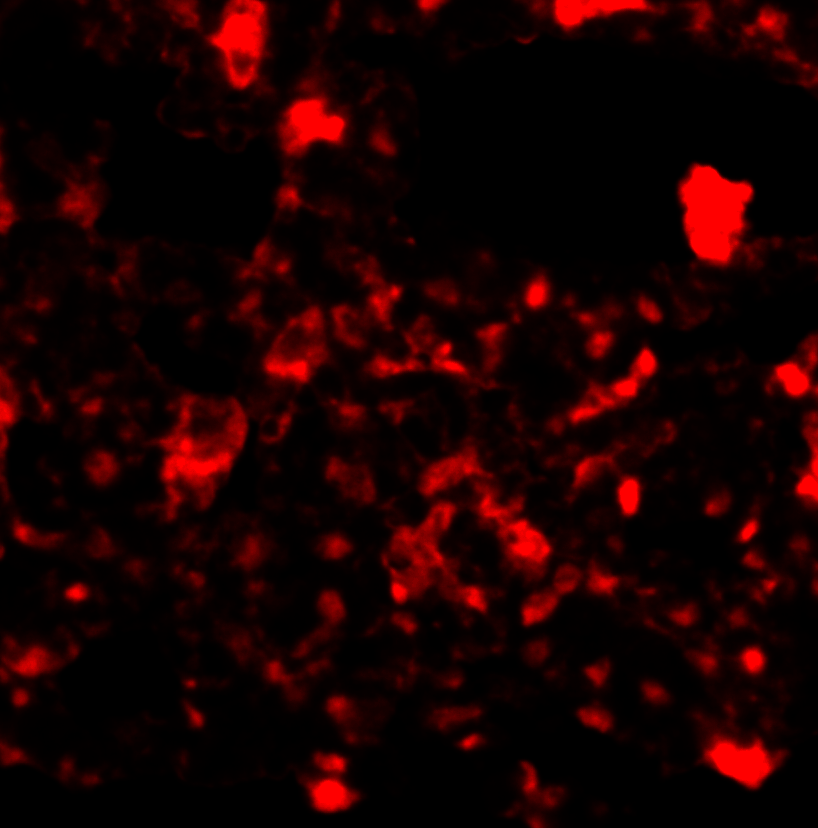

Supplement: Supplementary file 11 — Source Data EV Fig. 2 [file 44318_2023_3_MOESM11_ESM.zip › Figure EV2/2k-m/YTHDC1-WT YTHDC1, p16 and SPC IF/YTHDC1.tif]

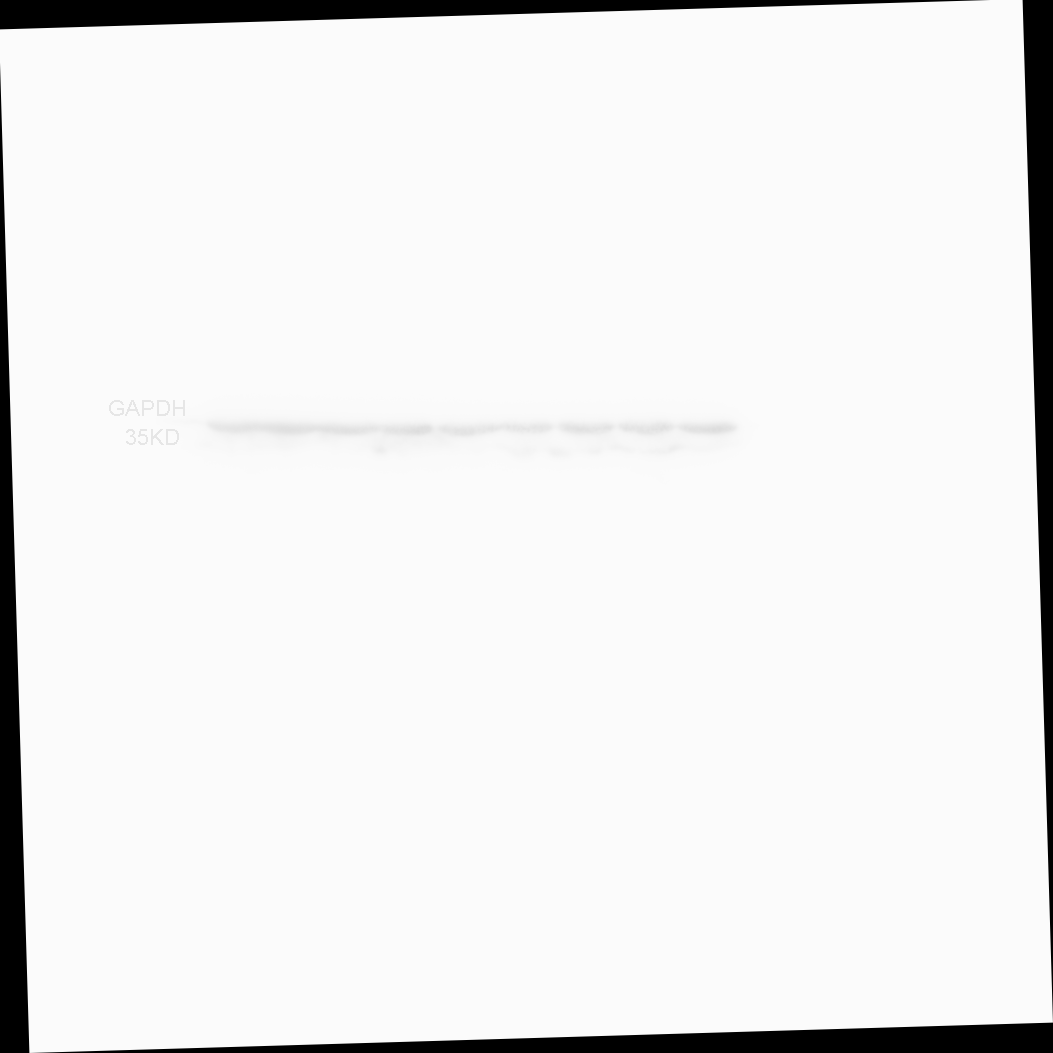

Supplement: Supplementary file 12 — Source Data EV Fig. 3 [file 44318_2023_3_MOESM12_ESM.zip › Figure EV3/3b-d/GAPDH NC-DMSO NC siDC1-1 -2 siDF2-1 -2 siIGFBP1 BP2 BP3 BLM .tif]

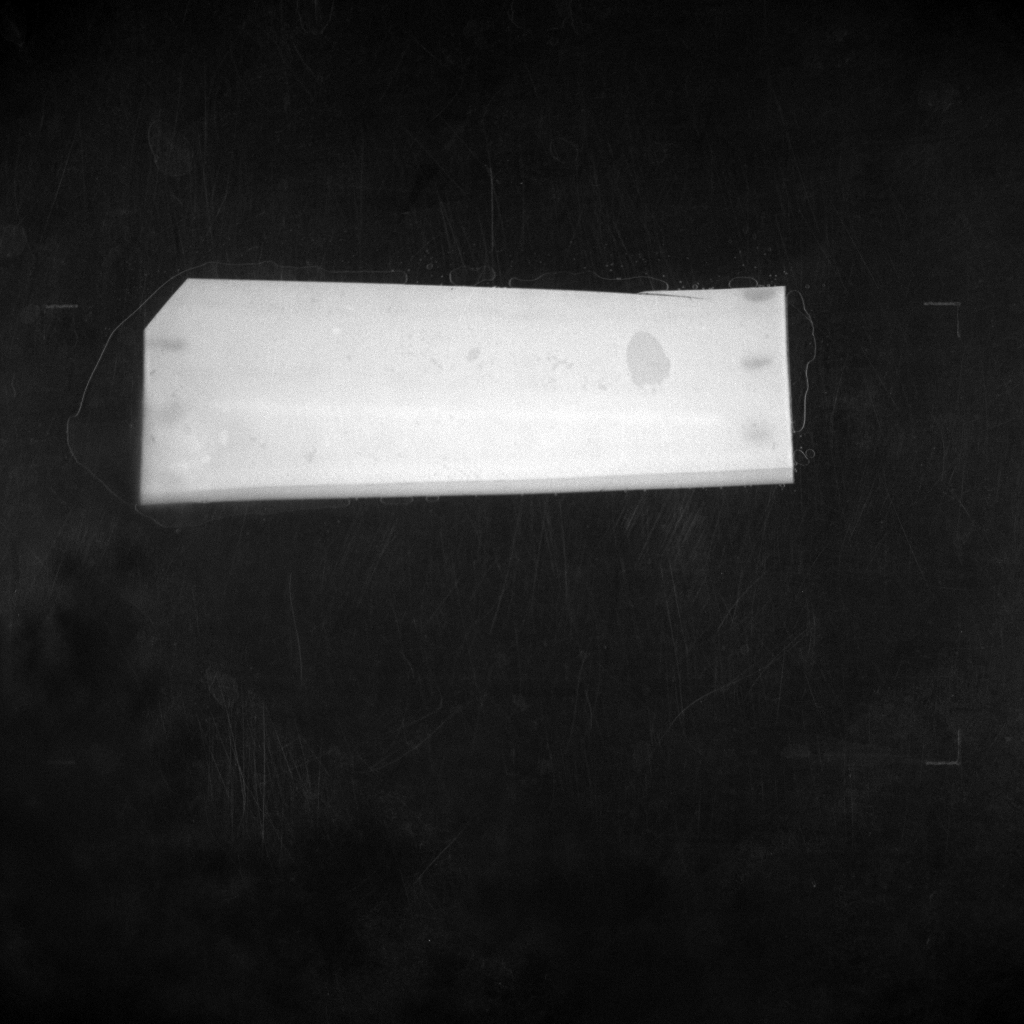

Supplement: Supplementary file 12 — Source Data EV Fig. 3 [file 44318_2023_3_MOESM12_ESM.zip › Figure EV3/3b-d/GAPDH NC-NC-VP16 siDC1-1 -2 siDF2-1 -2 siIGFBP1 BP2 BP3 BLM W .tif]

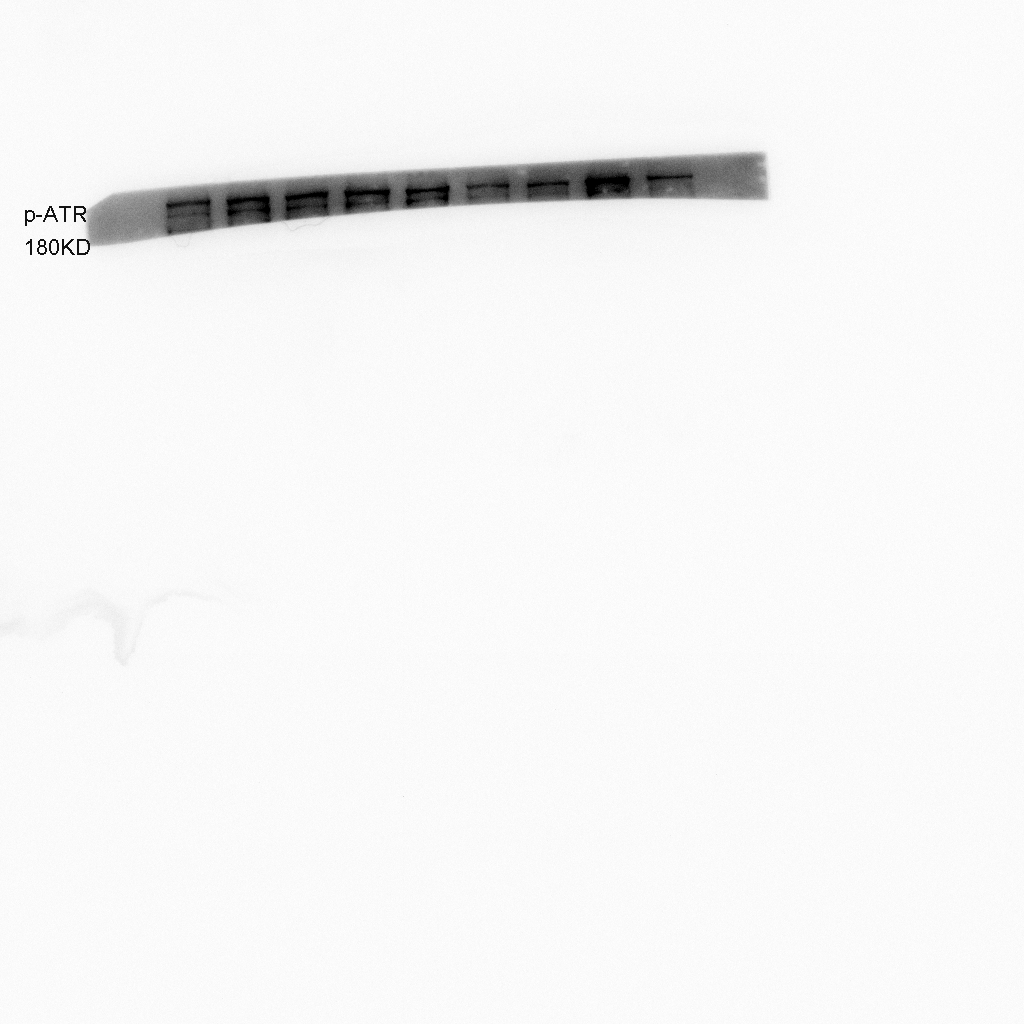

Supplement: Supplementary file 12 — Source Data EV Fig. 3 [file 44318_2023_3_MOESM12_ESM.zip › Figure EV3/3b-d/p-ATR NC-DMSO NC siDC1-1 -2 siDF2-1 -2 siIGFBP1 BP2 BP3 BLM .tif]

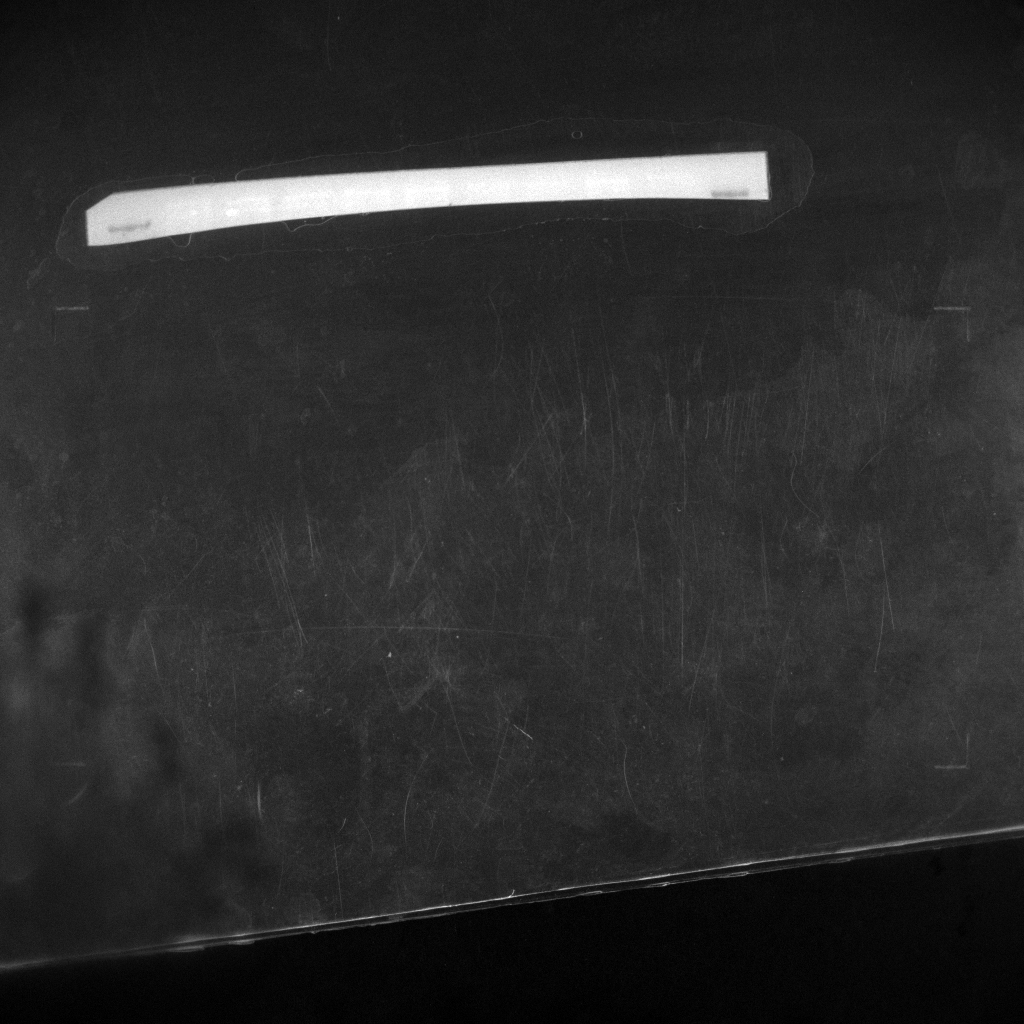

Supplement: Supplementary file 12 — Source Data EV Fig. 3 [file 44318_2023_3_MOESM12_ESM.zip › Figure EV3/3b-d/p-ATR NC-DMSO NC siDC1-1 -2 siDF2-1 -2 siIGFBP1 BP2 BP3 BLM w .tif]

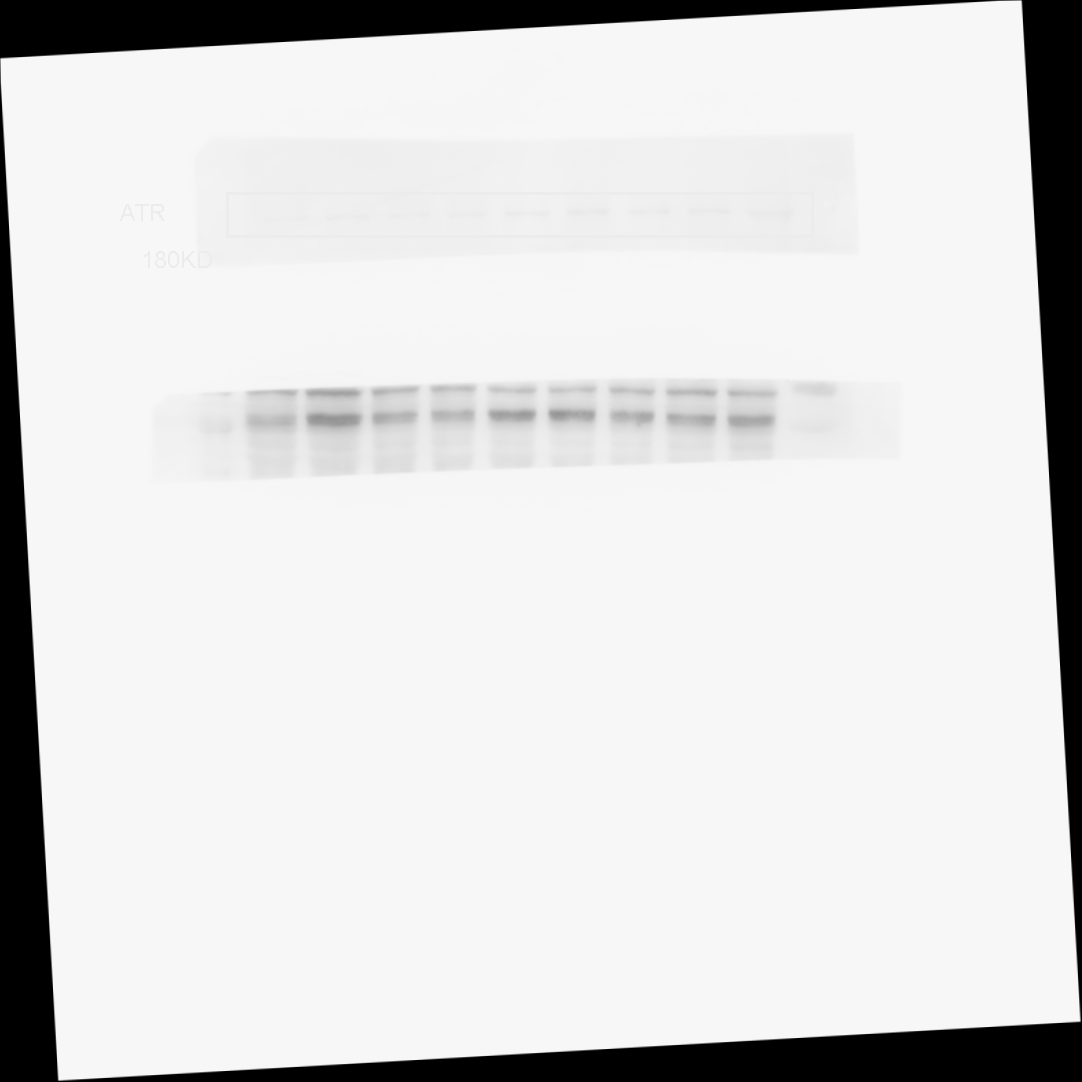

Supplement: Supplementary file 12 — Source Data EV Fig. 3 [file 44318_2023_3_MOESM12_ESM.zip › Figure EV3/3b-d/up ATR down chk1 NC NC-blm siDC1-1 -2 siDF2-1 -2 siIGFBP1 BP2 BP3 BLM w .tif]

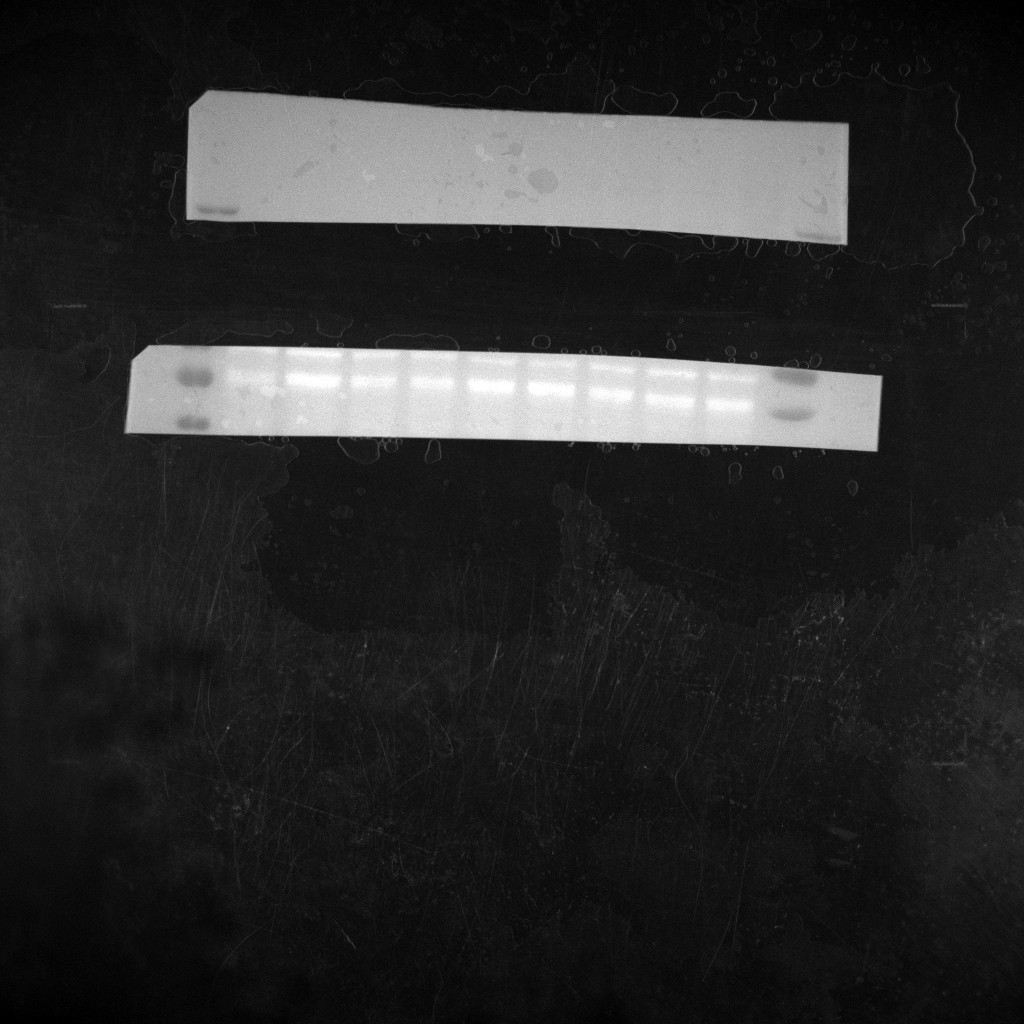

Supplement: Supplementary file 12 — Source Data EV Fig. 3 [file 44318_2023_3_MOESM12_ESM.zip › Figure EV3/3b-d/up ATR down chk1 NC-DMSO NC siDC1-1 -2 siDF2-1 -2 siIGFBP1 BP2 BP3 BLM .tif]

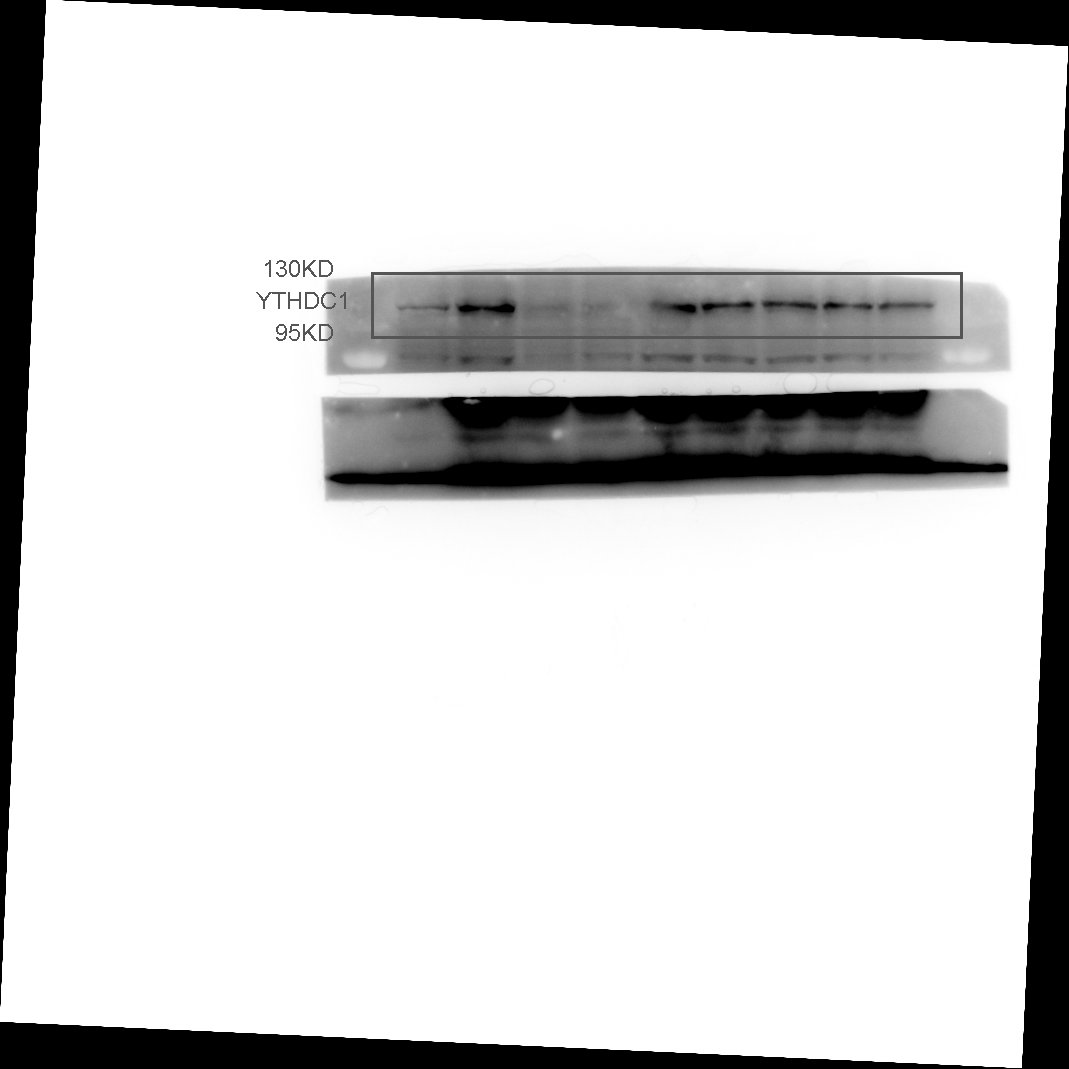

Supplement: Supplementary file 12 — Source Data EV Fig. 3 [file 44318_2023_3_MOESM12_ESM.zip › Figure EV3/3b-d/up dc1 down yh2ax NC-DMSO NC siDC1-1 -2 siDF2-1 -2 siIGFBP1 BP2 BP3 BLM .jpg]

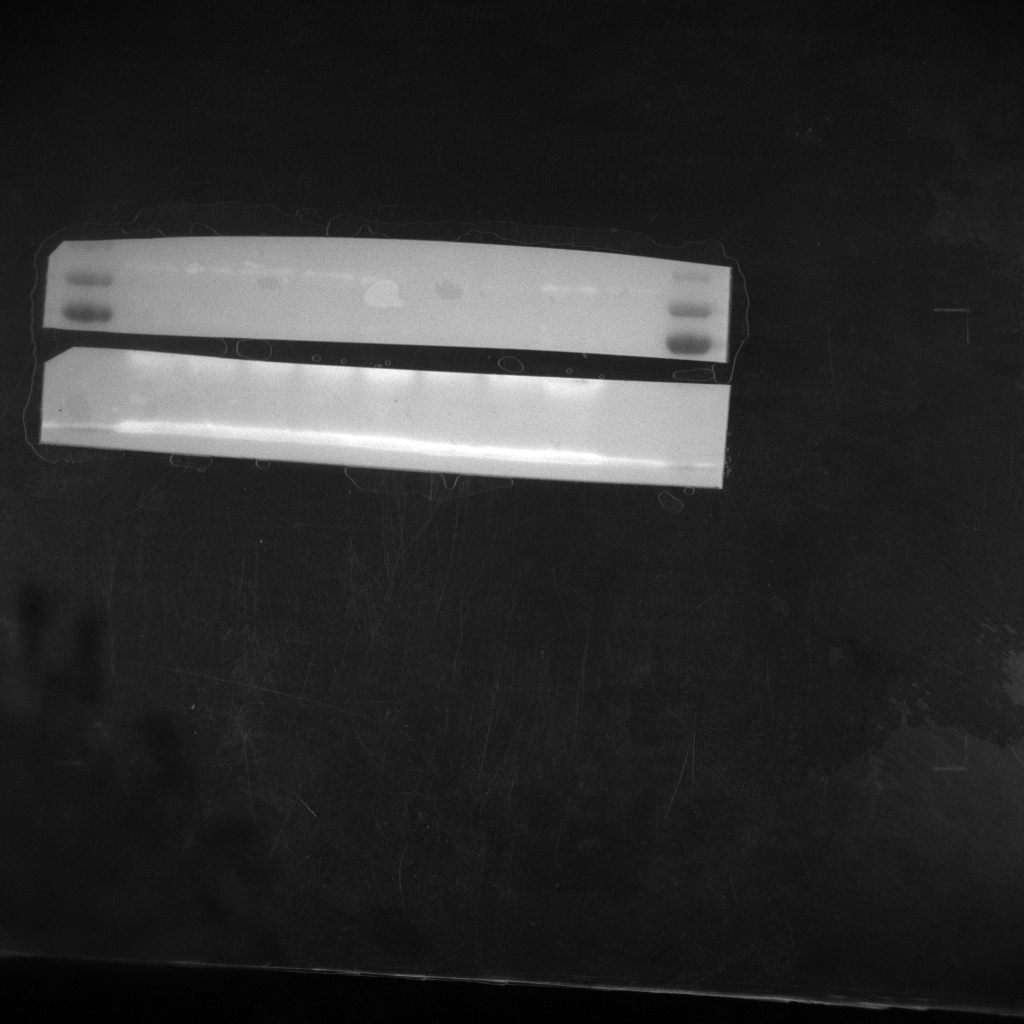

Supplement: Supplementary file 12 — Source Data EV Fig. 3 [file 44318_2023_3_MOESM12_ESM.zip › Figure EV3/3b-d/up dc1 down yh2ax NC-DMSO NC siDC1-1 -2 siDF2-1 -2 siIGFBP1 BP2 BP3 BLM w .tif]

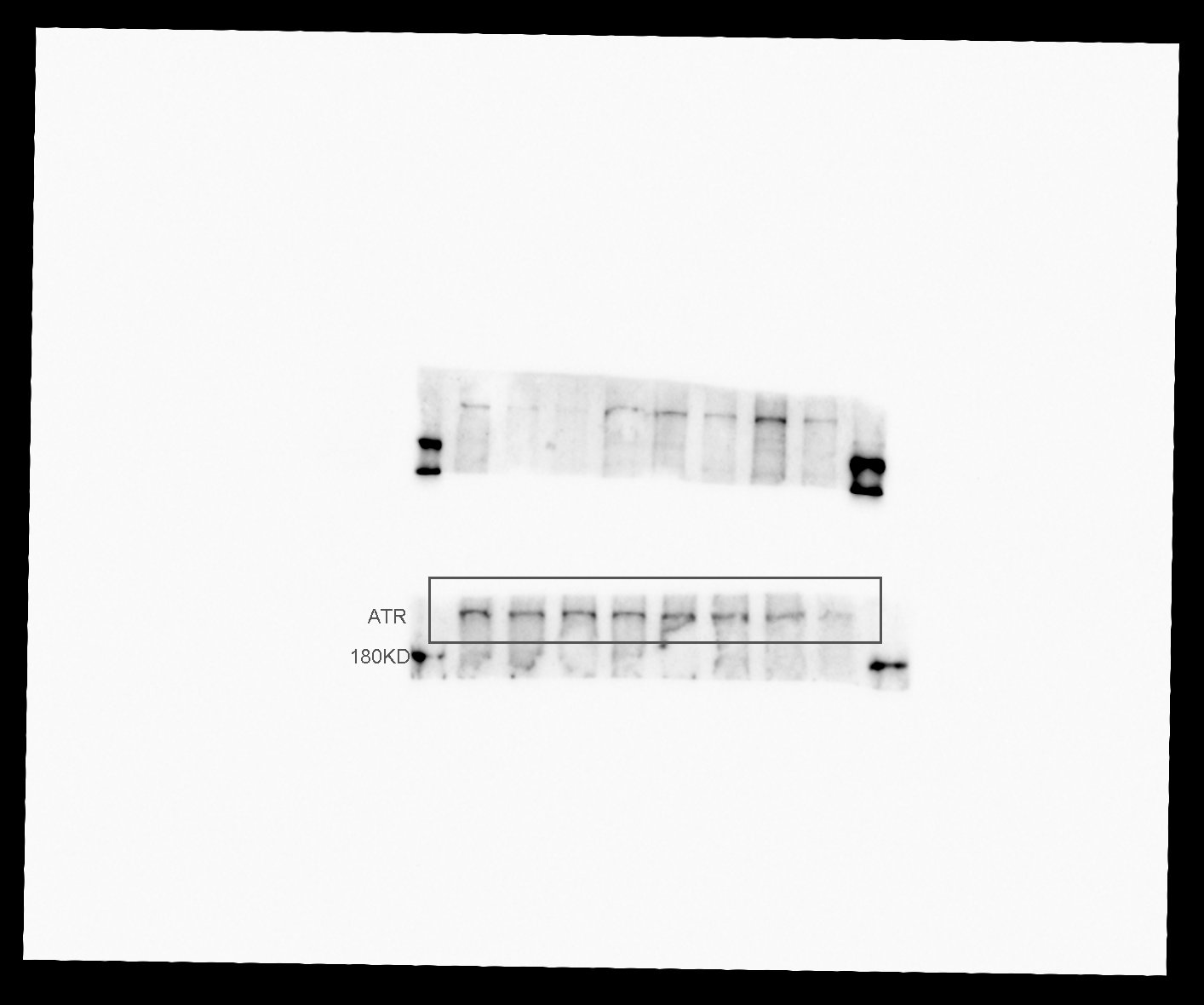

Supplement: Supplementary file 12 — Source Data EV Fig. 3 [file 44318_2023_3_MOESM12_ESM.zip › Figure EV3/3e-g/20211026 down ATR NC-DMSO NC-blm siDC1-1 -2 siDF2-1 -2 siIGFBP1 BP2 BP3 .jpg]

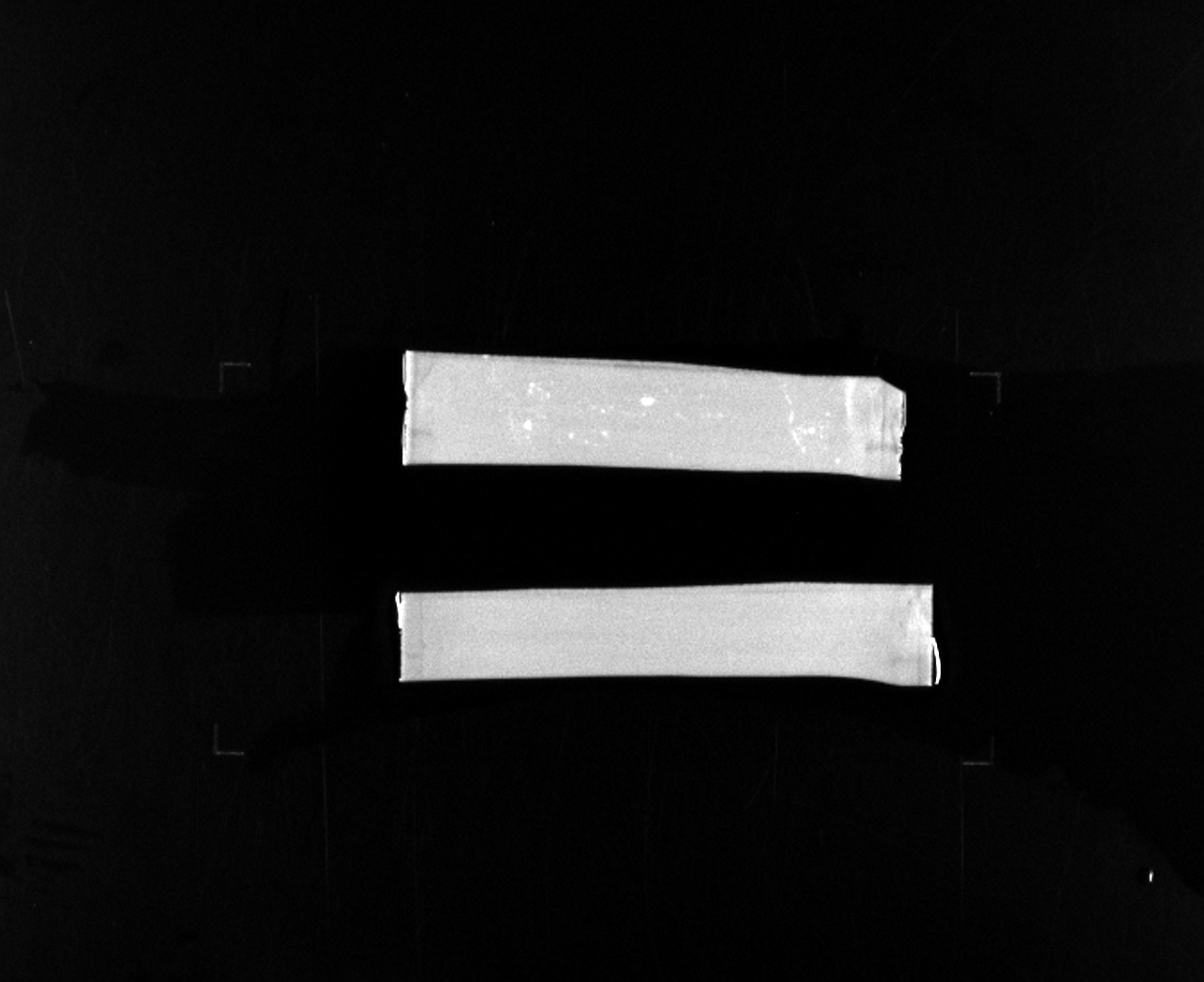

Supplement: Supplementary file 12 — Source Data EV Fig. 3 [file 44318_2023_3_MOESM12_ESM.zip › Figure EV3/3e-g/20211026 down ATR NC-DMSO NC-blm siDC1-1 -2 siDF2-1 -2 siIGFBP1 BP2 BP3 .Tif]

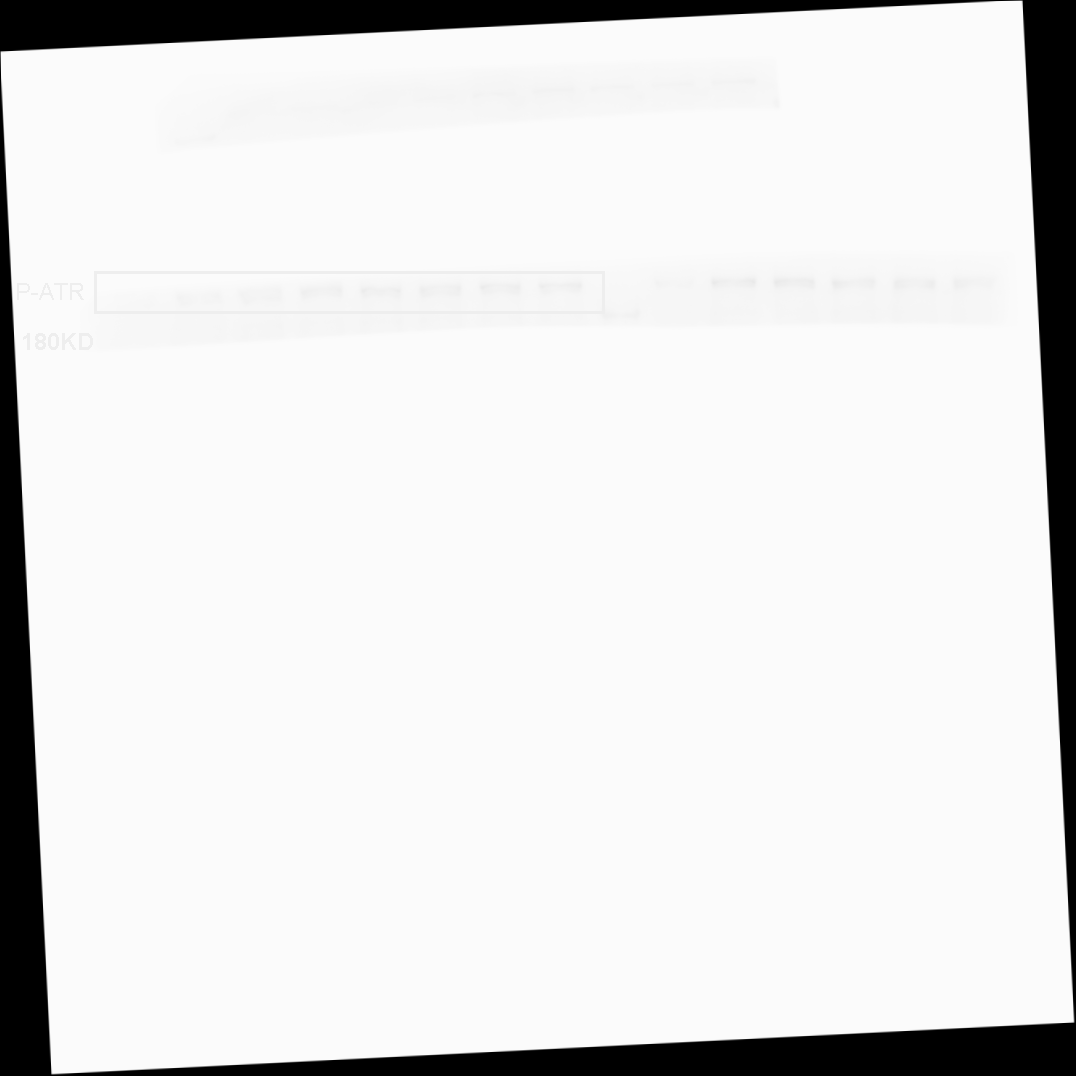

Supplement: Supplementary file 12 — Source Data EV Fig. 3 [file 44318_2023_3_MOESM12_ESM.zip › Figure EV3/3e-g/20211026 p-ATR UP NC-DMSO NC-blm siDC1-1 -2 siDF2-1 -2 siIGFBP1 BP2 BP3 down NC-DMSO NC siM3-1 -2 siM14-1 -2 siWTAP-1 -2 M NC-DMSO nc siFTO-1 -2 siALKBH5-1 -2 .tif]

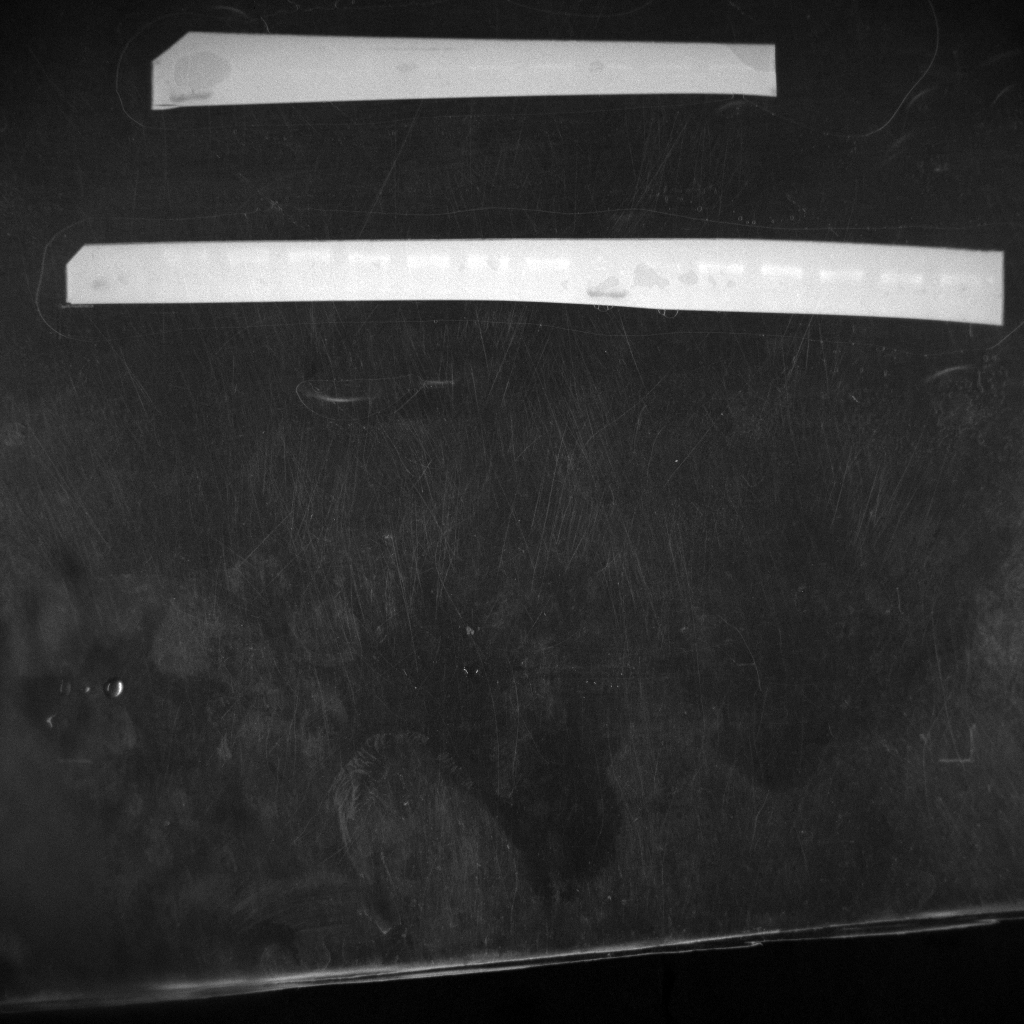

Supplement: Supplementary file 12 — Source Data EV Fig. 3 [file 44318_2023_3_MOESM12_ESM.zip › Figure EV3/3e-g/20211026 p-ATR UP NC-DMSO NC-blm siDC1-1 -2 siDF2-1 -2 siIGFBP1 BP2 BP3 down NC-DMSO NC siM3-1 -2 siM14-1 -2 siWTAP-1 -2 M NC-DMSO nc siFTO-1 -2 siALKBH5-1 -2 W .tif]

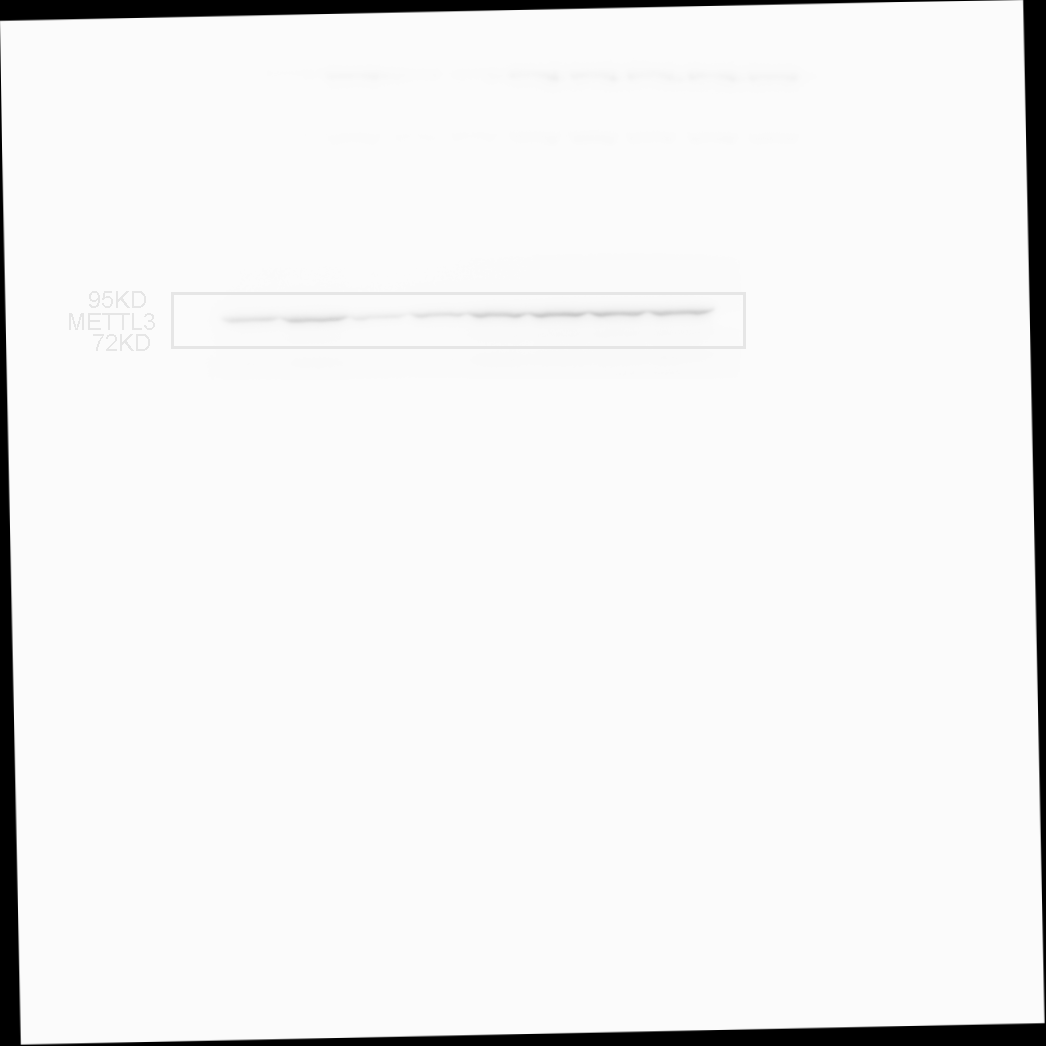

Supplement: Supplementary file 12 — Source Data EV Fig. 3 [file 44318_2023_3_MOESM12_ESM.zip › Figure EV3/3e-g/20211026 up YTHDC1 NC-DMSO NC-blmsiDC1-1 -2 siDF2-1 -2 siIGFBP1 BP2 BP3 down METTL3 NC-DMSO NC siM3-1 -2 siM14-1 -2 siWTAP-1 -2 .tif]

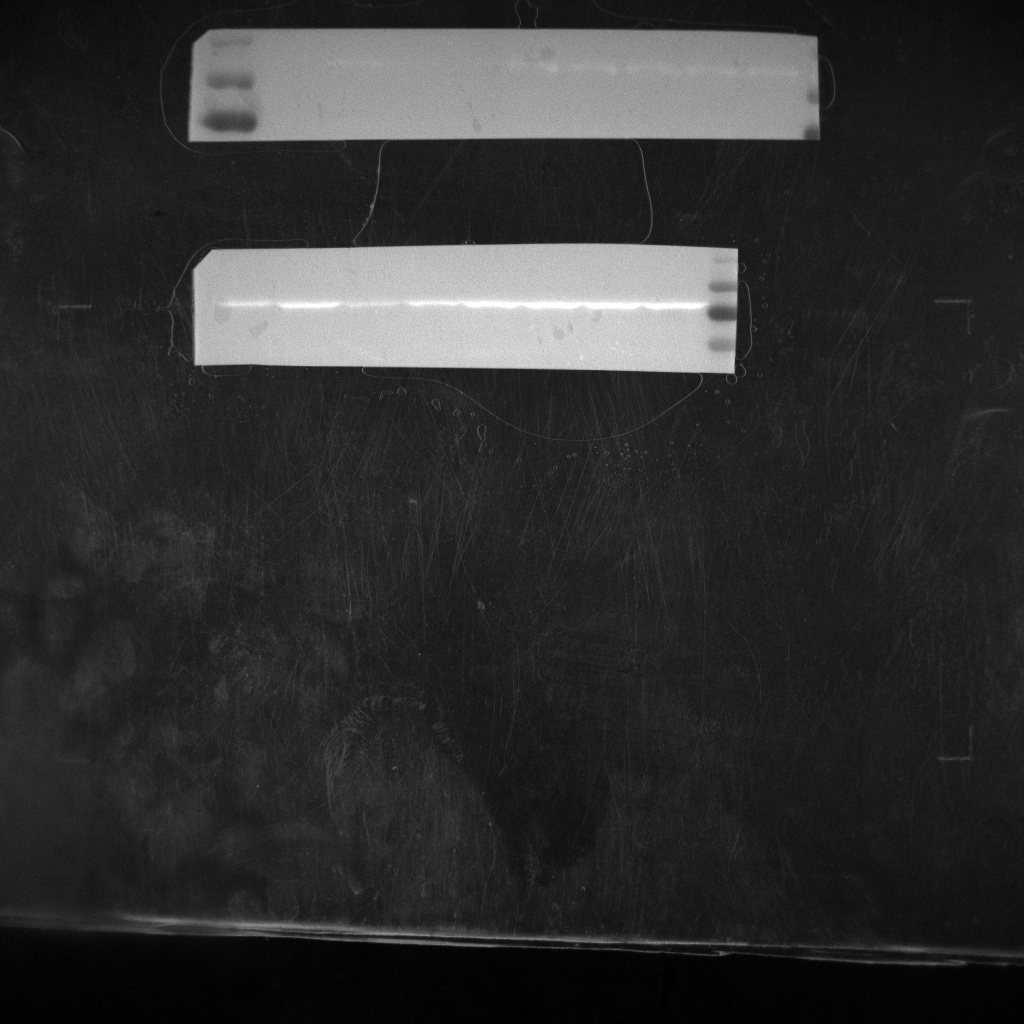

Supplement: Supplementary file 12 — Source Data EV Fig. 3 [file 44318_2023_3_MOESM12_ESM.zip › Figure EV3/3e-g/20211026 up YTHDC1 NC-DMSO NC-blmsiDC1-1 -2 siDF2-1 -2 siIGFBP1 BP2 BP3 down METTL3 NC-DMSO NC siM3-1 -2 siM14-1 -2 siWTAP-1 -2 W .tif]

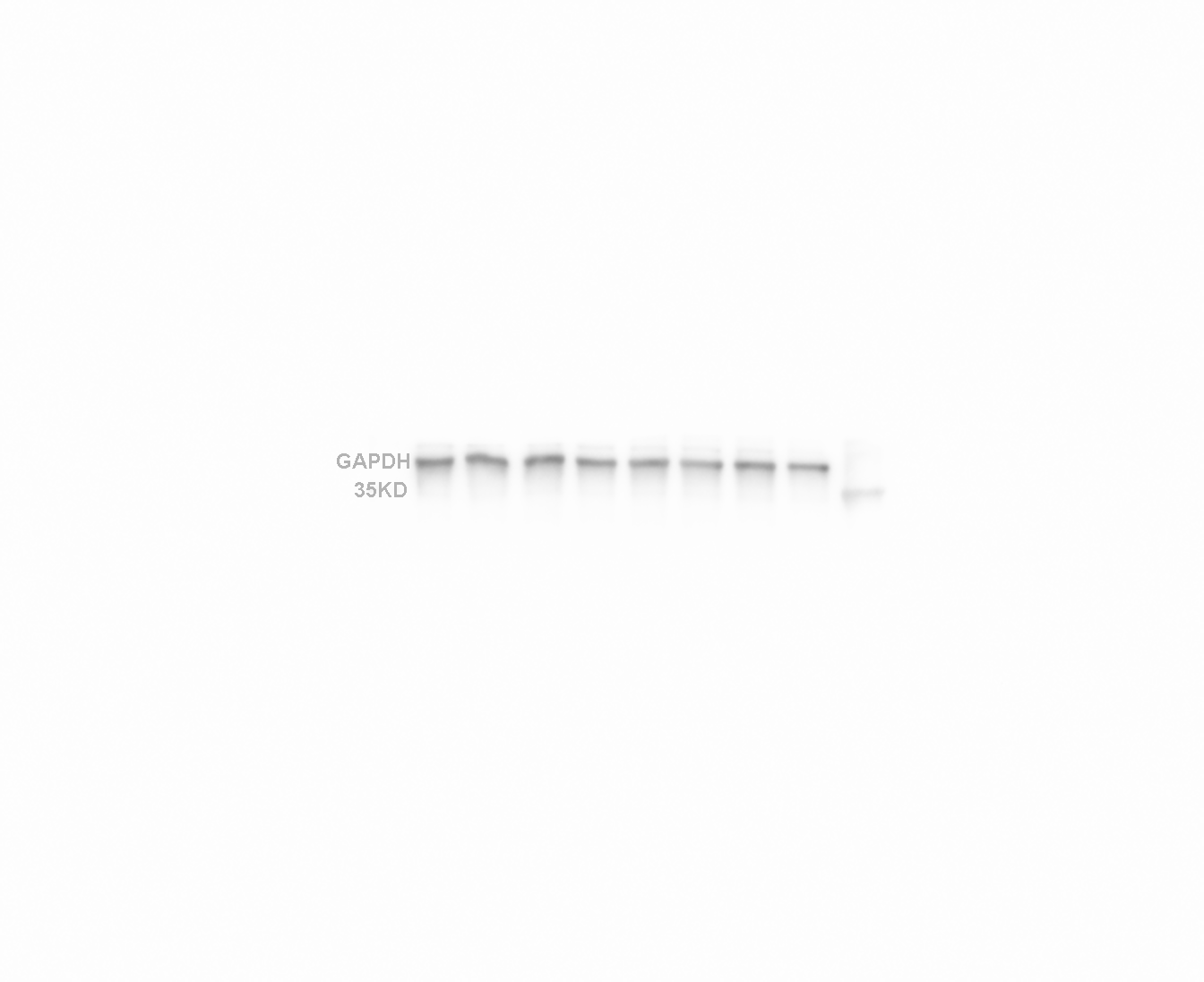

Supplement: Supplementary file 12 — Source Data EV Fig. 3 [file 44318_2023_3_MOESM12_ESM.zip › Figure EV3/3e-g/gapdh nc shm3 14 wtap blm .Tif]

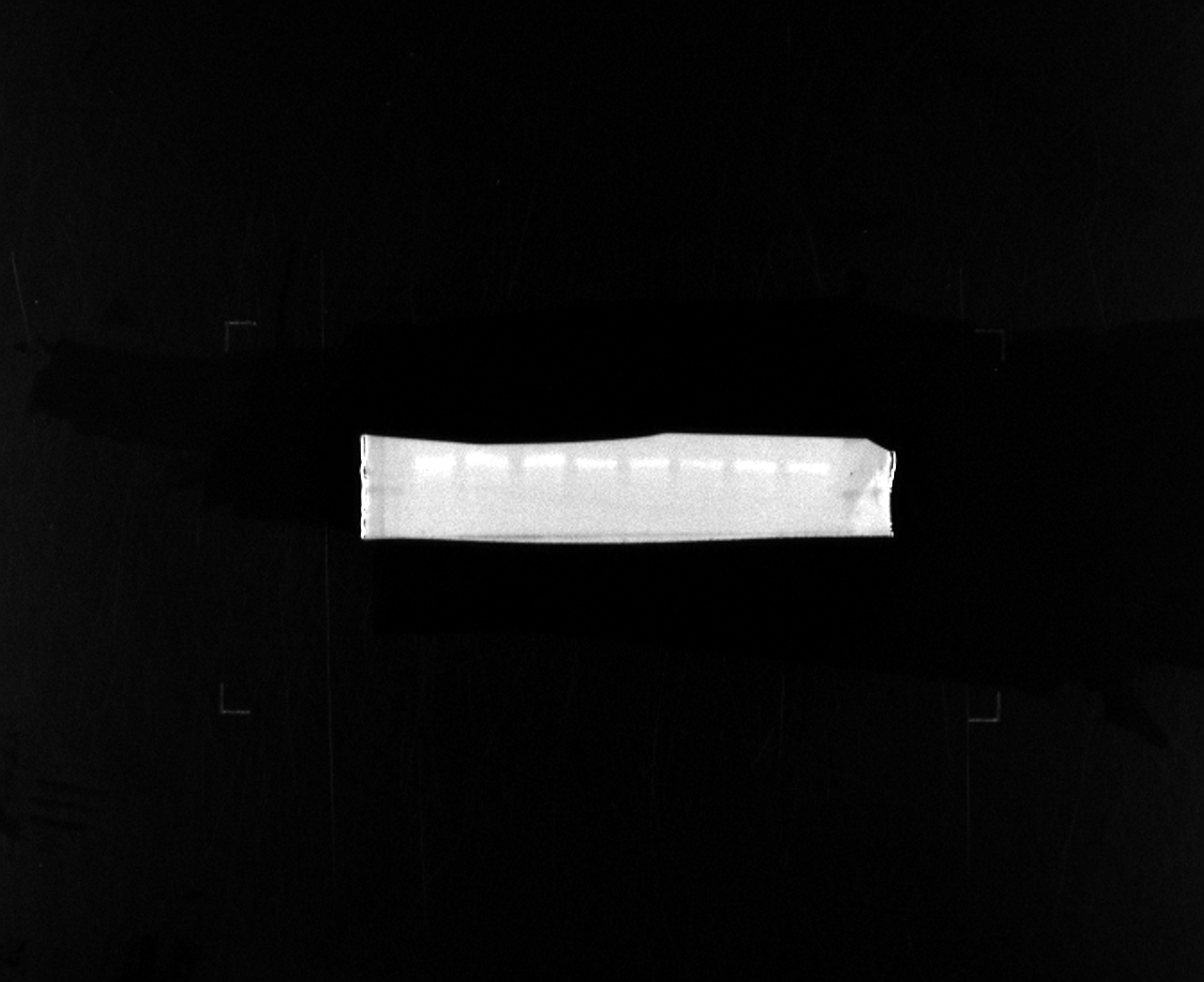

Supplement: Supplementary file 12 — Source Data EV Fig. 3 [file 44318_2023_3_MOESM12_ESM.zip › Figure EV3/3e-g/gapdh nc shm3 14 wtap blm w.Tif]

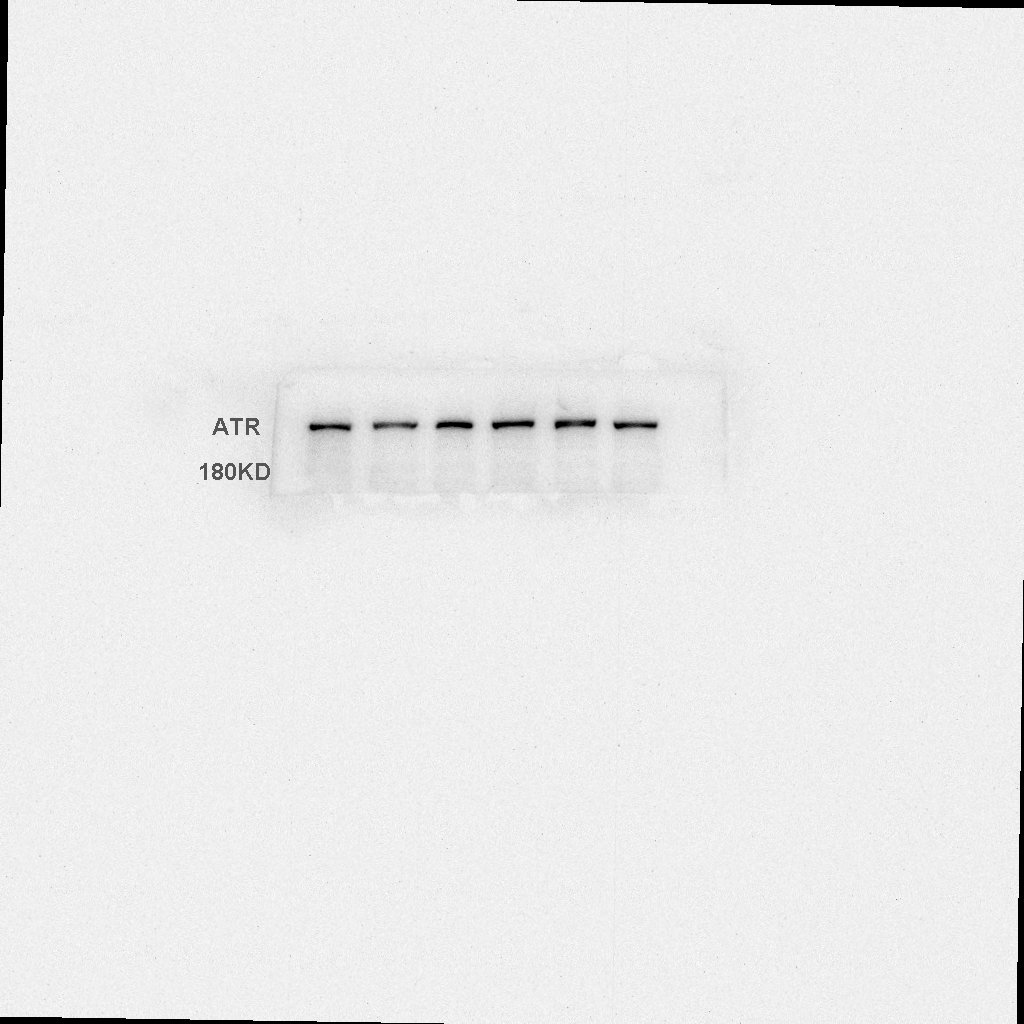

Supplement: Supplementary file 12 — Source Data EV Fig. 3 [file 44318_2023_3_MOESM12_ESM.zip › Figure EV3/3h-j/ATR right to left DMSO NC siFTO-1 -2 siALKBH5-1 -2 -1 (BLM).jpg]

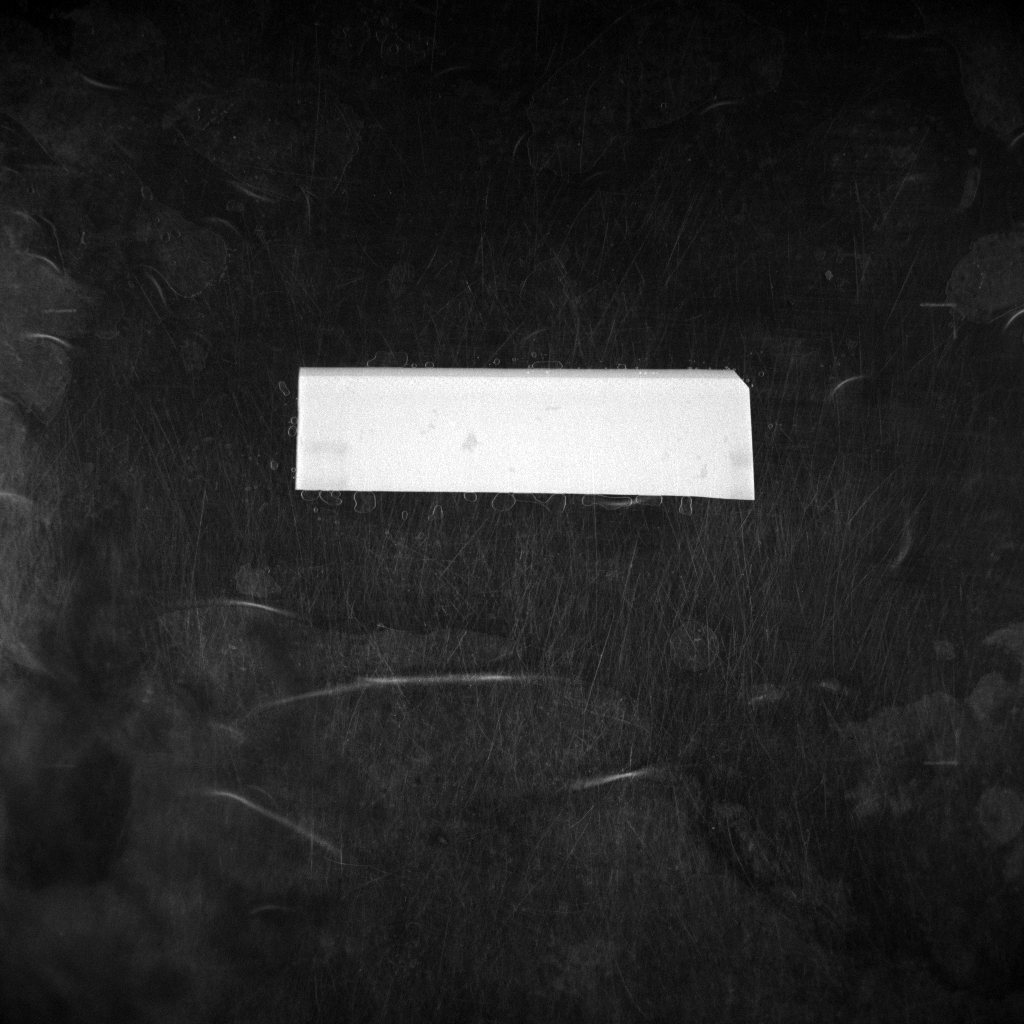

Supplement: Supplementary file 12 — Source Data EV Fig. 3 [file 44318_2023_3_MOESM12_ESM.zip › Figure EV3/3h-j/ATR right to left DMSO NC siFTO-1 -2 siALKBH5-1 -2 W -1 (BLM).tif]

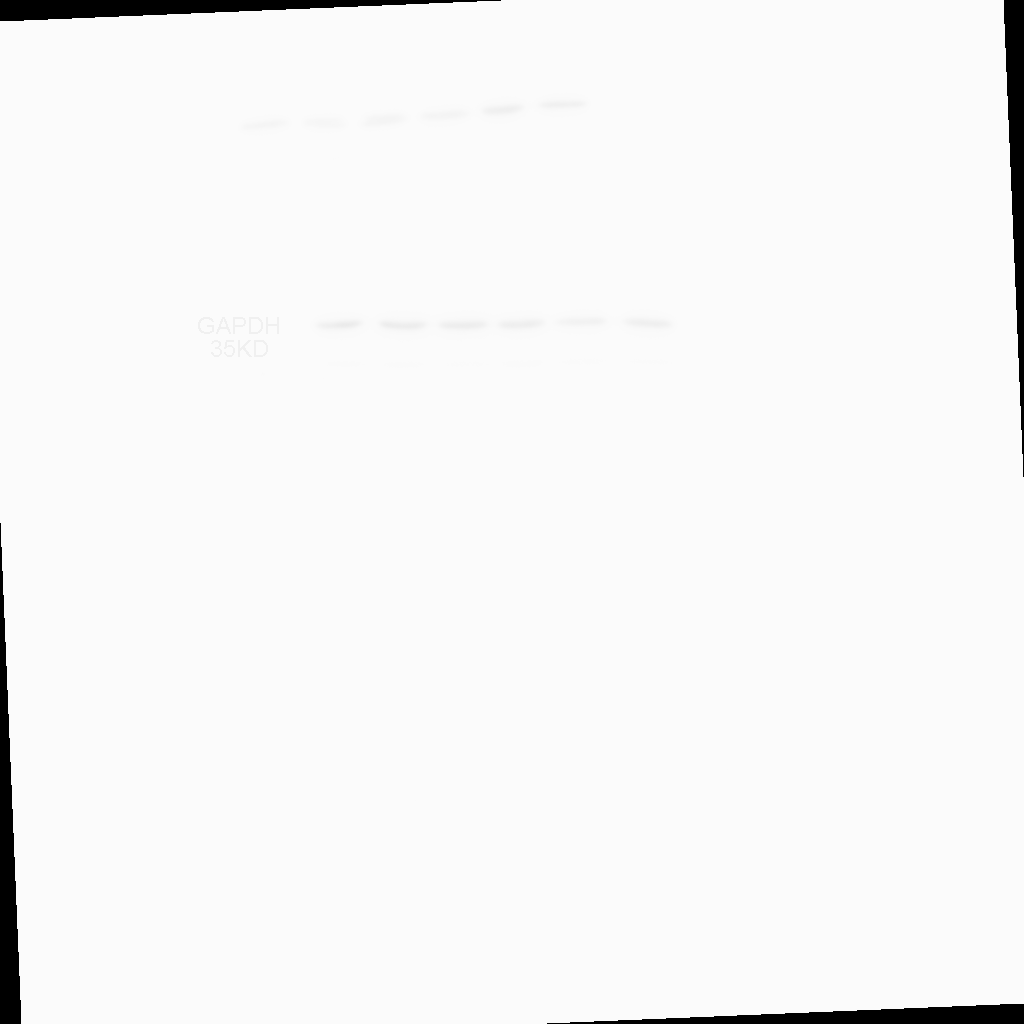

Supplement: Supplementary file 12 — Source Data EV Fig. 3 [file 44318_2023_3_MOESM12_ESM.zip › Figure EV3/3h-j/GAPDH right to left DMSO NC siFTO-1 -2 siALKBH5-1 -2 (BLM) .tif]

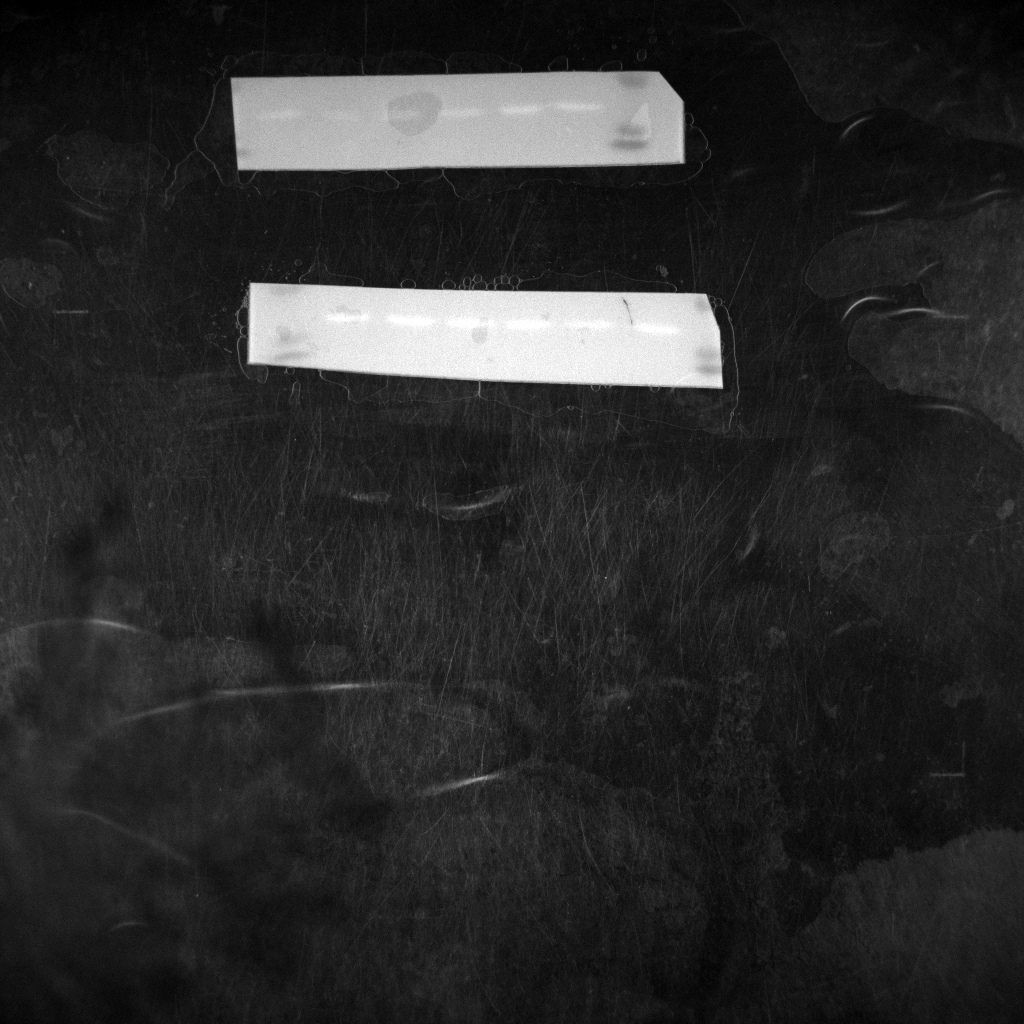

Supplement: Supplementary file 12 — Source Data EV Fig. 3 [file 44318_2023_3_MOESM12_ESM.zip › Figure EV3/3h-j/GAPDH right to left DMSO NC siFTO-1 -2 siALKBH5-1 -2 (BLM) W.tif]

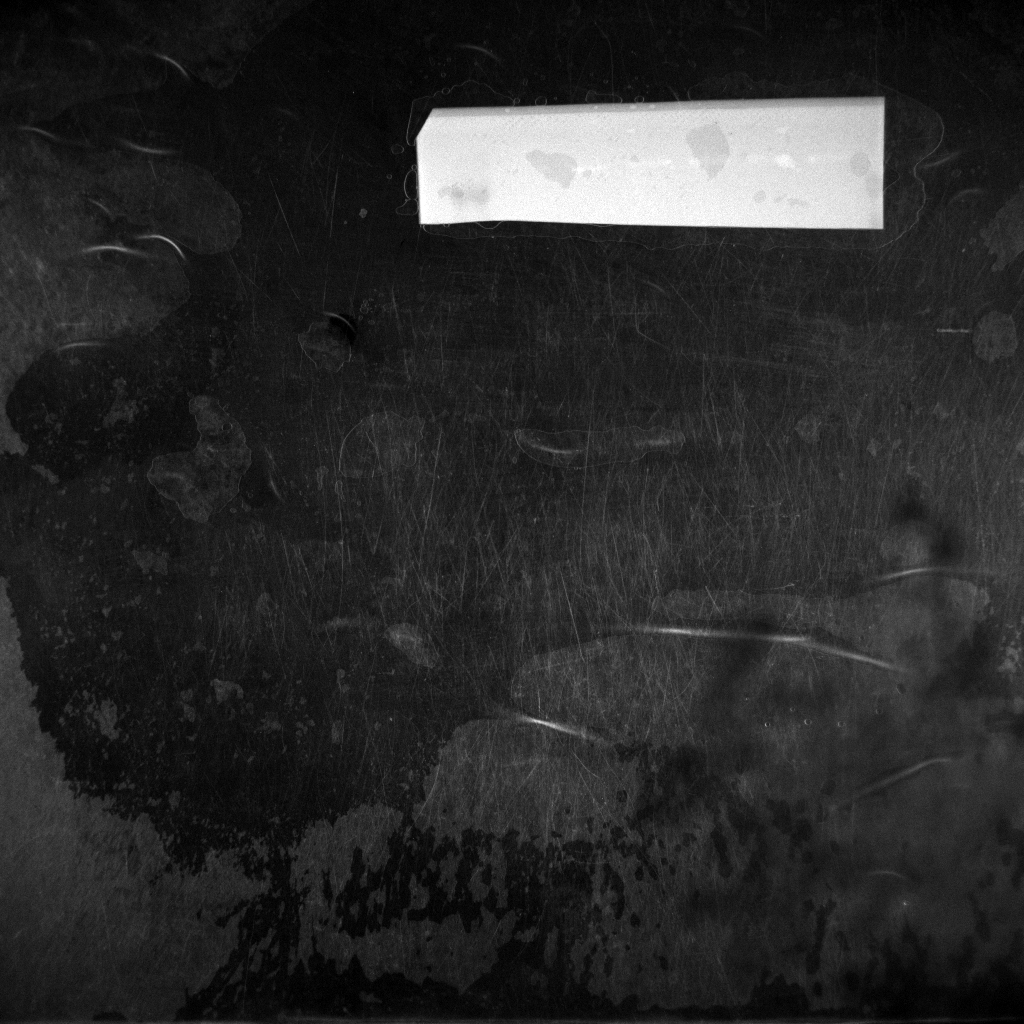

Supplement: Supplementary file 12 — Source Data EV Fig. 3 [file 44318_2023_3_MOESM12_ESM.zip › Figure EV3/3h-j/p-ATR right to left DMSO NC siFTO-1 -2 siALKBH5-1 -2 (BLM) W.tif]

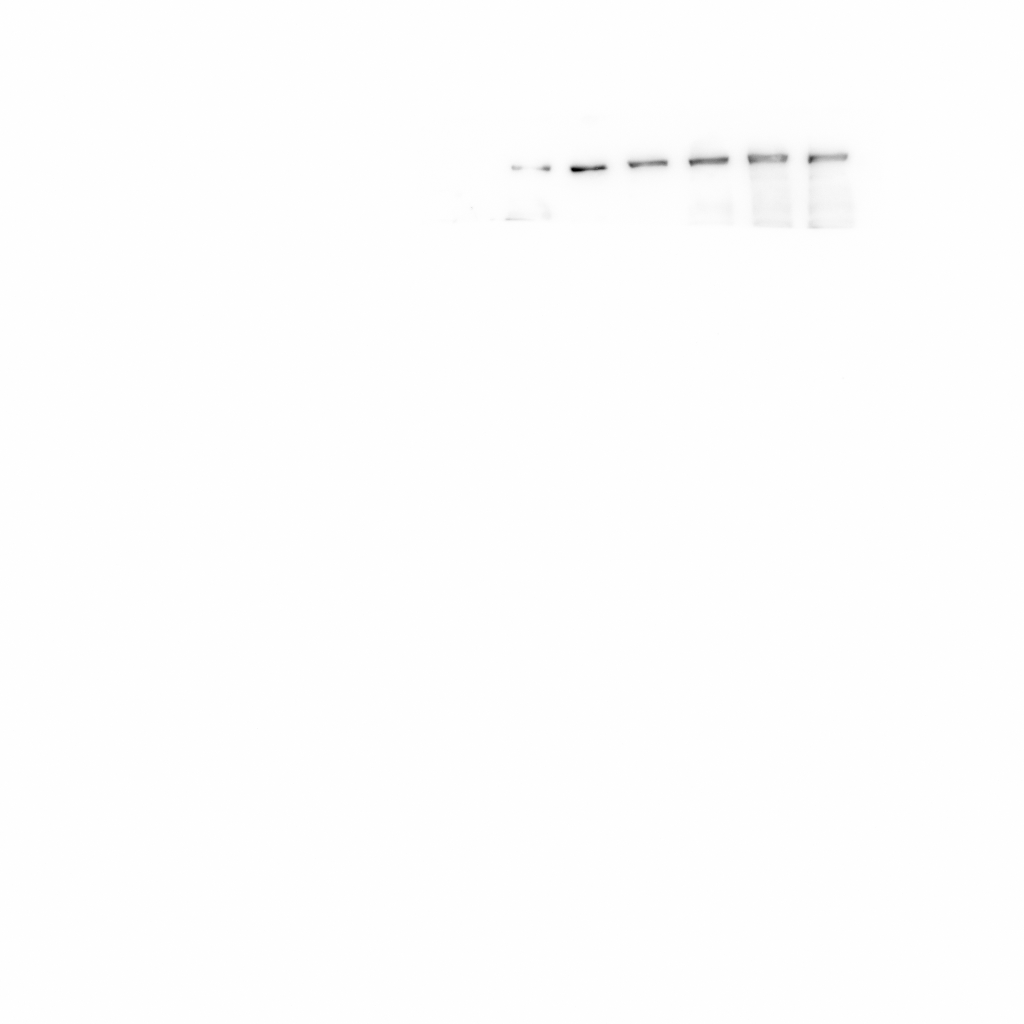

Supplement: Supplementary file 12 — Source Data EV Fig. 3 [file 44318_2023_3_MOESM12_ESM.zip › Figure EV3/3h-j/p-ATR right to left DMSO NC siFTO-1 -2 siALKBH5-1 -2 (BLM).tif]

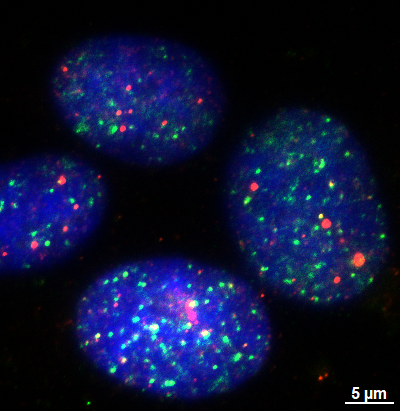

Supplement: Supplementary file 13 — Source Data EV Fig. 4 [file 44318_2023_3_MOESM13_ESM.zip › Figure EV4/4a-b/nc RPA1 and yh2ax IF/MERGE_(DAPI+FITC+Texasred).TIF]

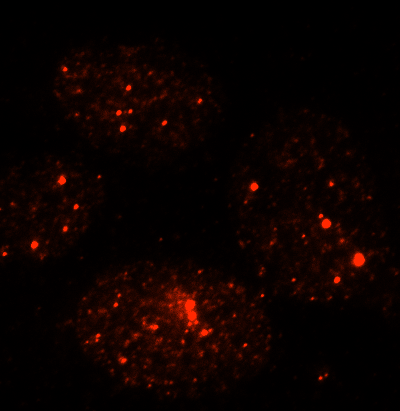

Supplement: Supplementary file 13 — Source Data EV Fig. 4 [file 44318_2023_3_MOESM13_ESM.zip › Figure EV4/4a-b/nc RPA1 and yh2ax IF/RPA1_Texasred.TIF]

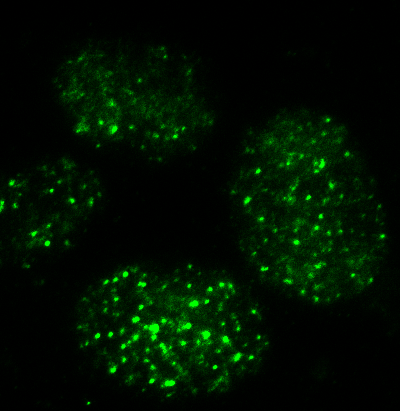

Supplement: Supplementary file 13 — Source Data EV Fig. 4 [file 44318_2023_3_MOESM13_ESM.zip › Figure EV4/4a-b/nc RPA1 and yh2ax IF/yh2ax_FITC.TIF]

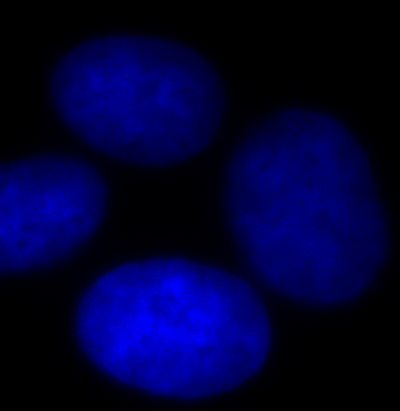

Supplement: Supplementary file 13 — Source Data EV Fig. 4 [file 44318_2023_3_MOESM13_ESM.zip › Figure EV4/4a-b/nc RPA1 and yh2ax IF/_DAPI.TIF]

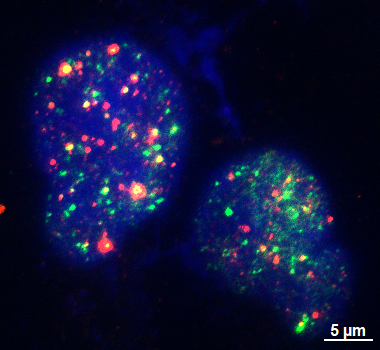

Supplement: Supplementary file 13 — Source Data EV Fig. 4 [file 44318_2023_3_MOESM13_ESM.zip › Figure EV4/4a-b/siDC1-1 RPA1 and yh2ax IF/merge_(DAPI+FITC+Texasred).TIF]

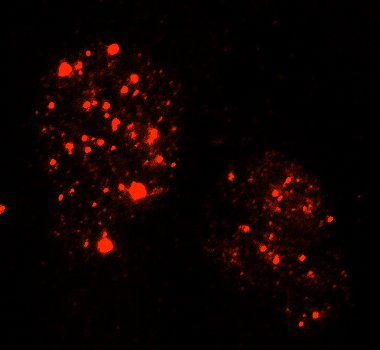

Supplement: Supplementary file 13 — Source Data EV Fig. 4 [file 44318_2023_3_MOESM13_ESM.zip › Figure EV4/4a-b/siDC1-1 RPA1 and yh2ax IF/RPA1_Texasred.TIF]

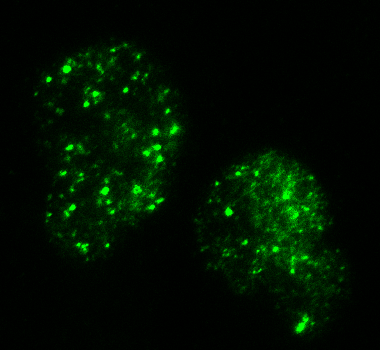

Supplement: Supplementary file 13 — Source Data EV Fig. 4 [file 44318_2023_3_MOESM13_ESM.zip › Figure EV4/4a-b/siDC1-1 RPA1 and yh2ax IF/yh2ax_FITC.TIF]

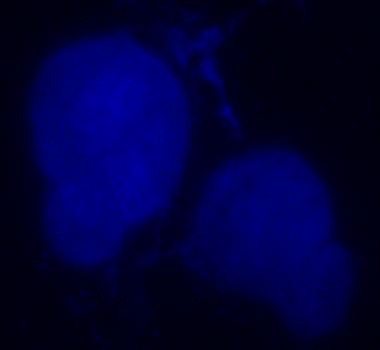

Supplement: Supplementary file 13 — Source Data EV Fig. 4 [file 44318_2023_3_MOESM13_ESM.zip › Figure EV4/4a-b/siDC1-1 RPA1 and yh2ax IF/_DAPI.TIF]

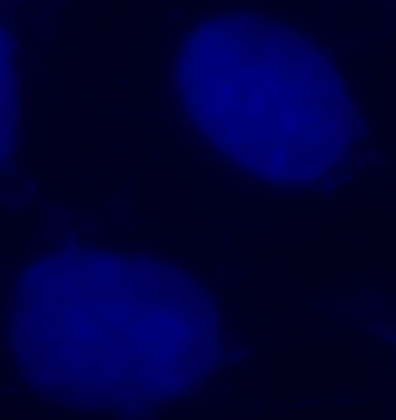

Supplement: Supplementary file 13 — Source Data EV Fig. 4 [file 44318_2023_3_MOESM13_ESM.zip › Figure EV4/4a-b/siDC1-2 RPA1 and yh2ax IF/DAPI.TIF]

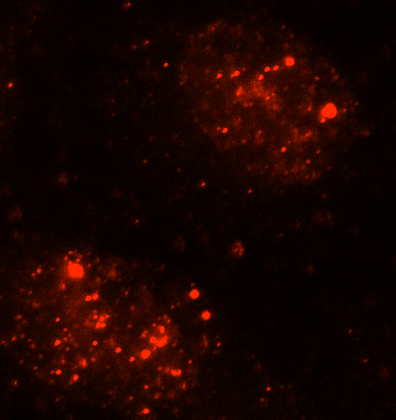

Supplement: Supplementary file 13 — Source Data EV Fig. 4 [file 44318_2023_3_MOESM13_ESM.zip › Figure EV4/4a-b/siDC1-2 RPA1 and yh2ax IF/RPA1_Texasred.TIF]

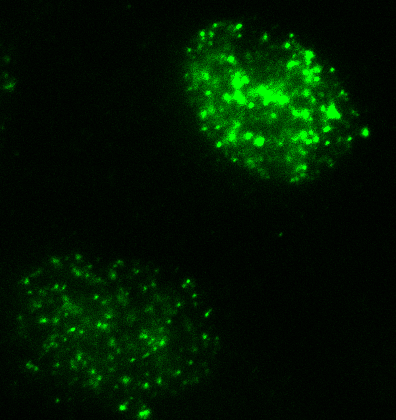

Supplement: Supplementary file 13 — Source Data EV Fig. 4 [file 44318_2023_3_MOESM13_ESM.zip › Figure EV4/4a-b/siDC1-2 RPA1 and yh2ax IF/yh2ax FITC.TIF]

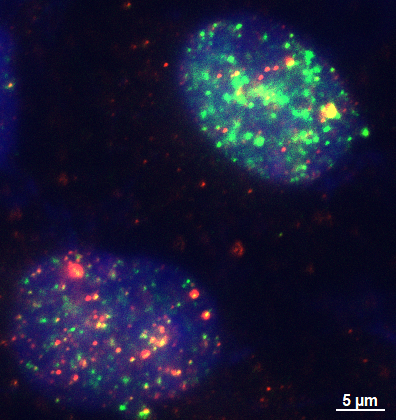

Supplement: Supplementary file 13 — Source Data EV Fig. 4 [file 44318_2023_3_MOESM13_ESM.zip › Figure EV4/4a-b/siDC1-2 RPA1 and yh2ax IF/_(DAPI+FITC+Texasred).TIF]

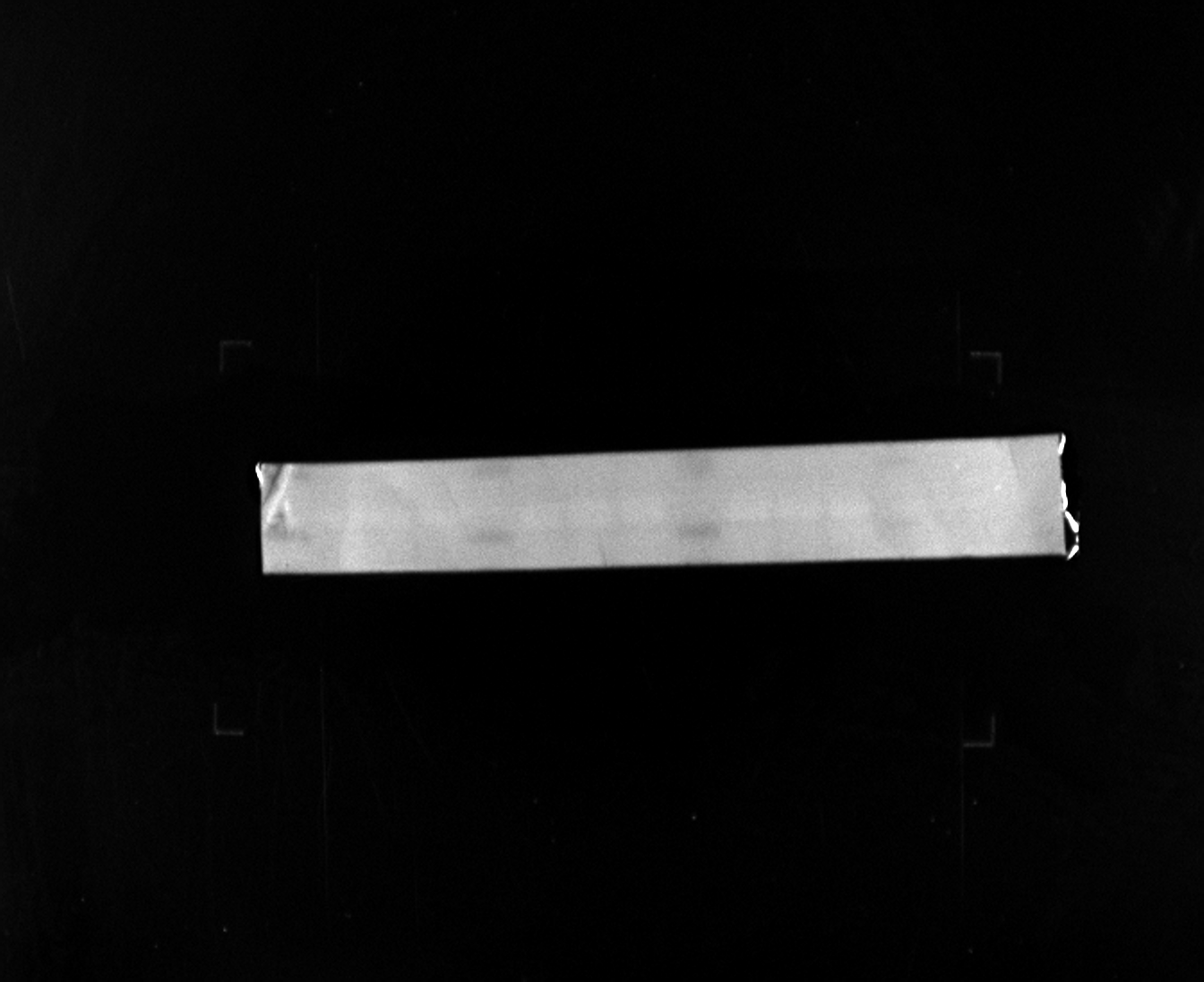

Supplement: Supplementary file 13 — Source Data EV Fig. 4 [file 44318_2023_3_MOESM13_ESM.zip › Figure EV4/4c/GAPDH W.Tif]

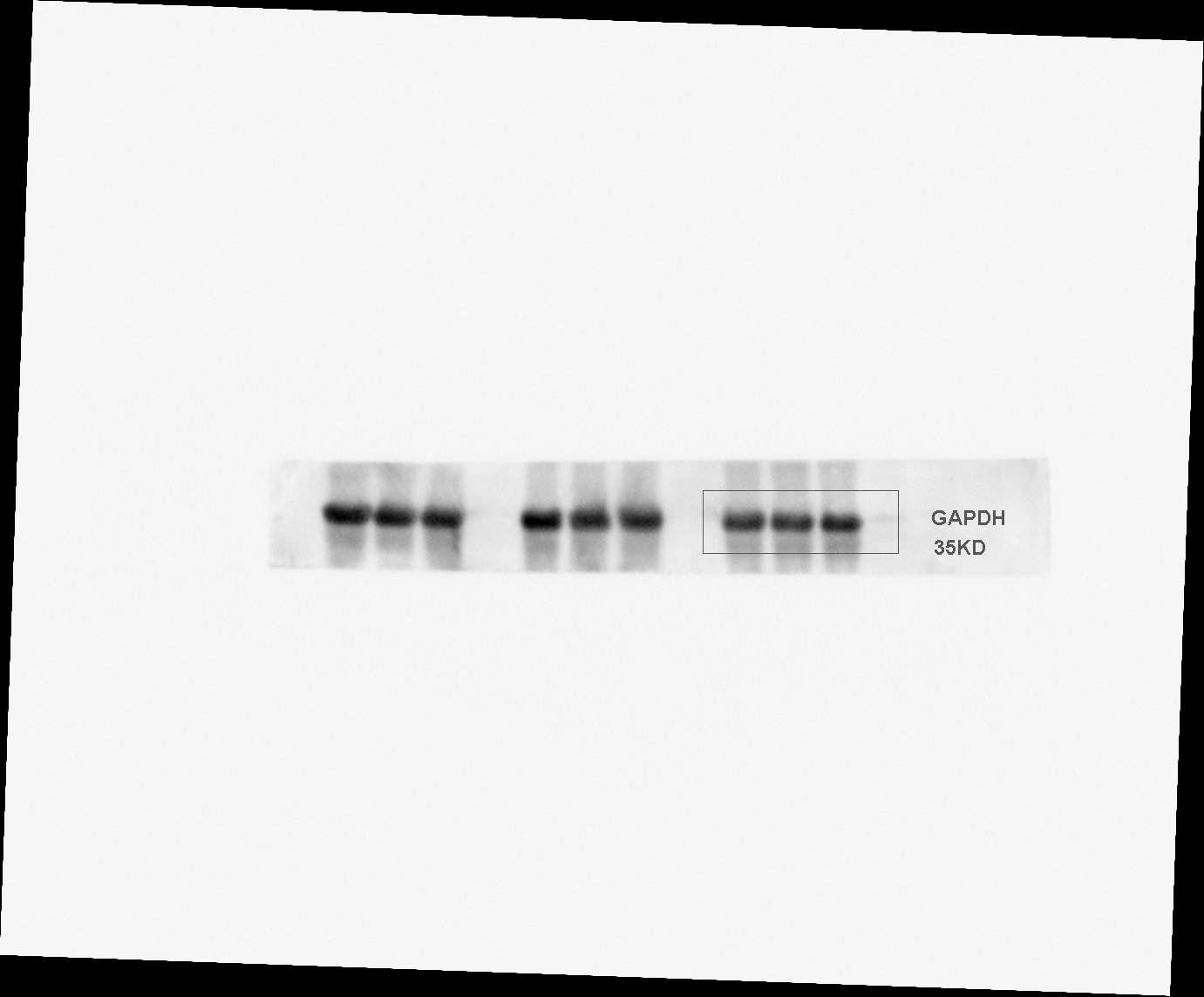

Supplement: Supplementary file 13 — Source Data EV Fig. 4 [file 44318_2023_3_MOESM13_ESM.zip › Figure EV4/4c/GAPDH.jpg]

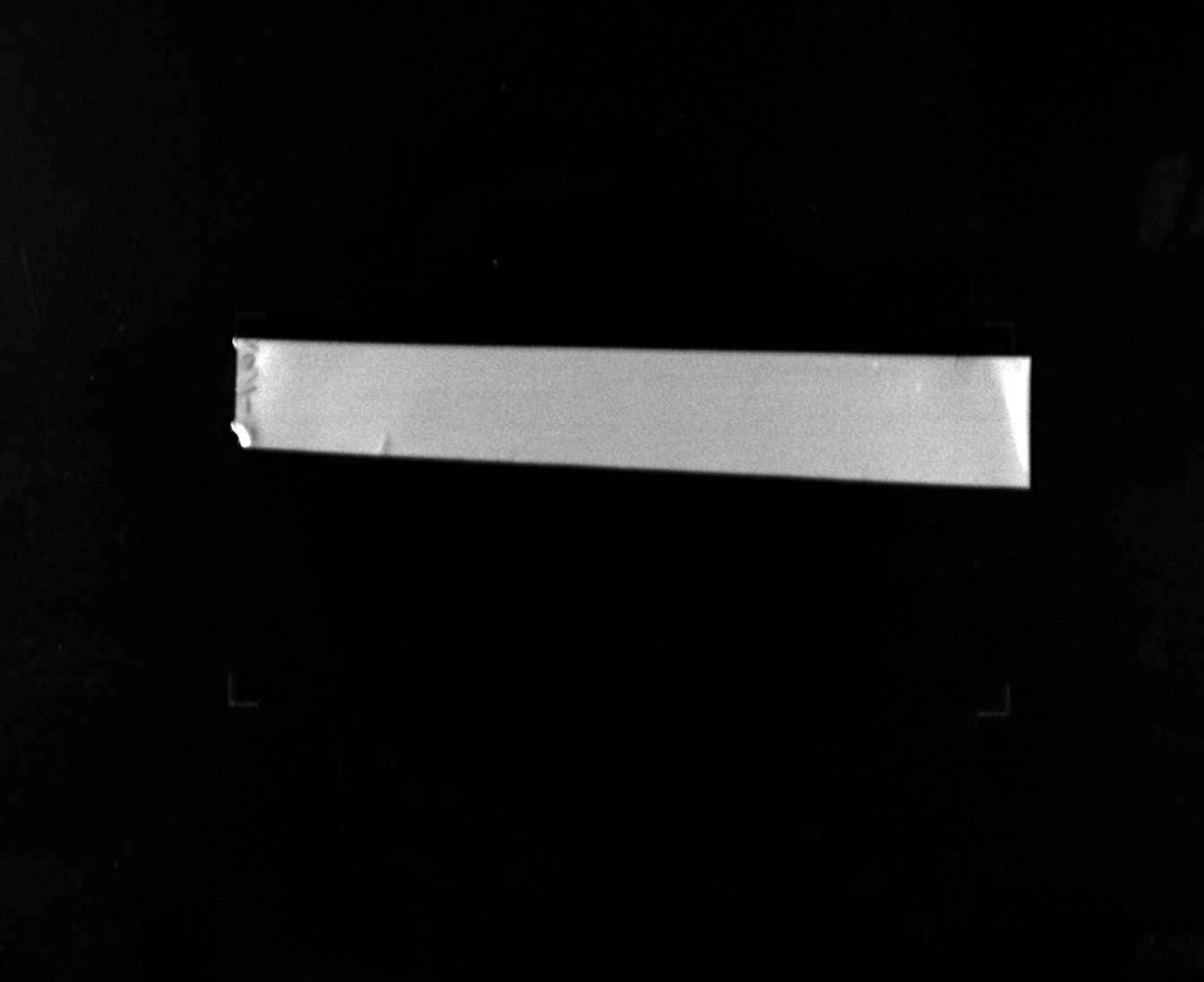

Supplement: Supplementary file 13 — Source Data EV Fig. 4 [file 44318_2023_3_MOESM13_ESM.zip › Figure EV4/4c/TopBP1 W.Tif]

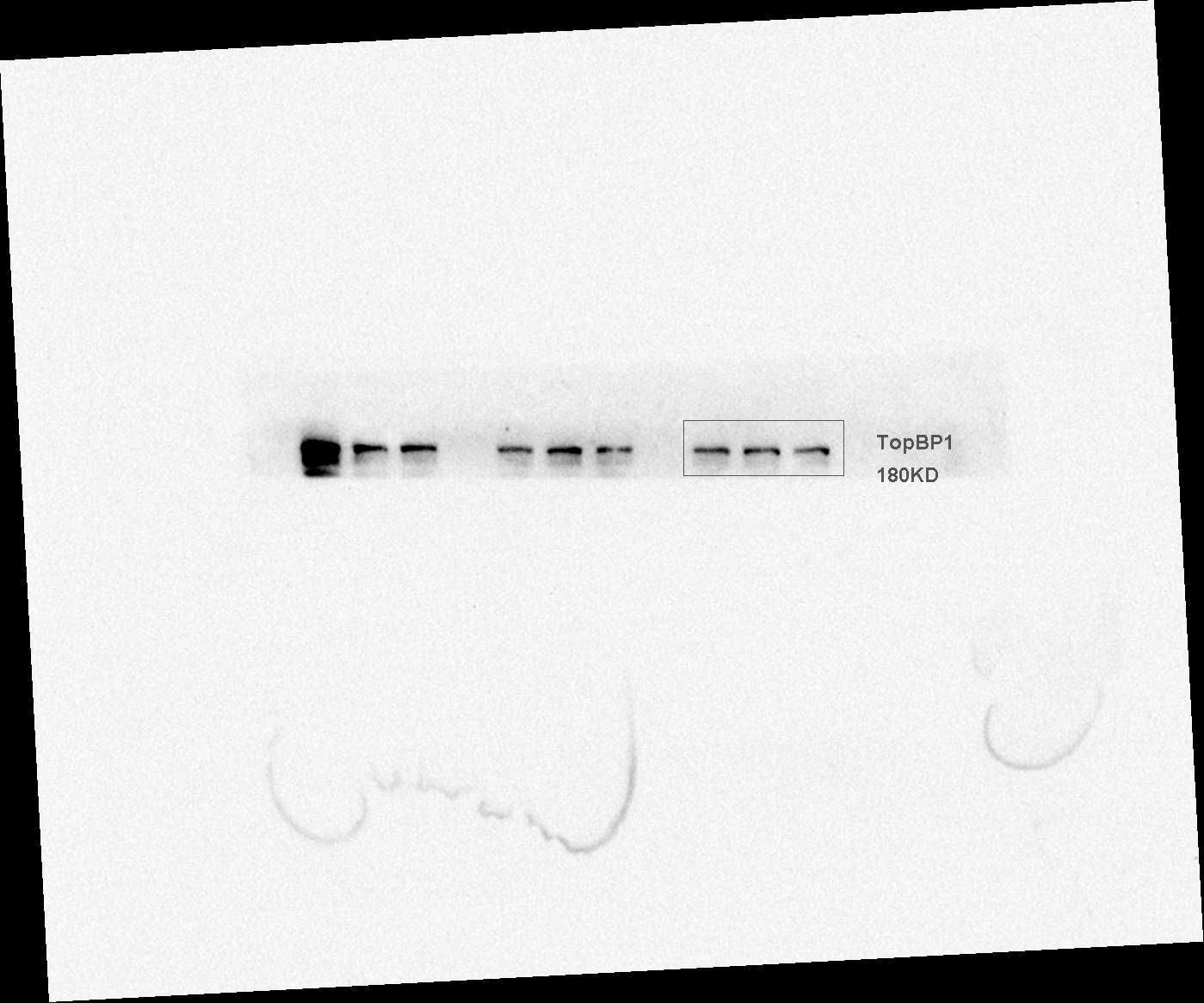

Supplement: Supplementary file 13 — Source Data EV Fig. 4 [file 44318_2023_3_MOESM13_ESM.zip › Figure EV4/4c/TopBP1.jpg]

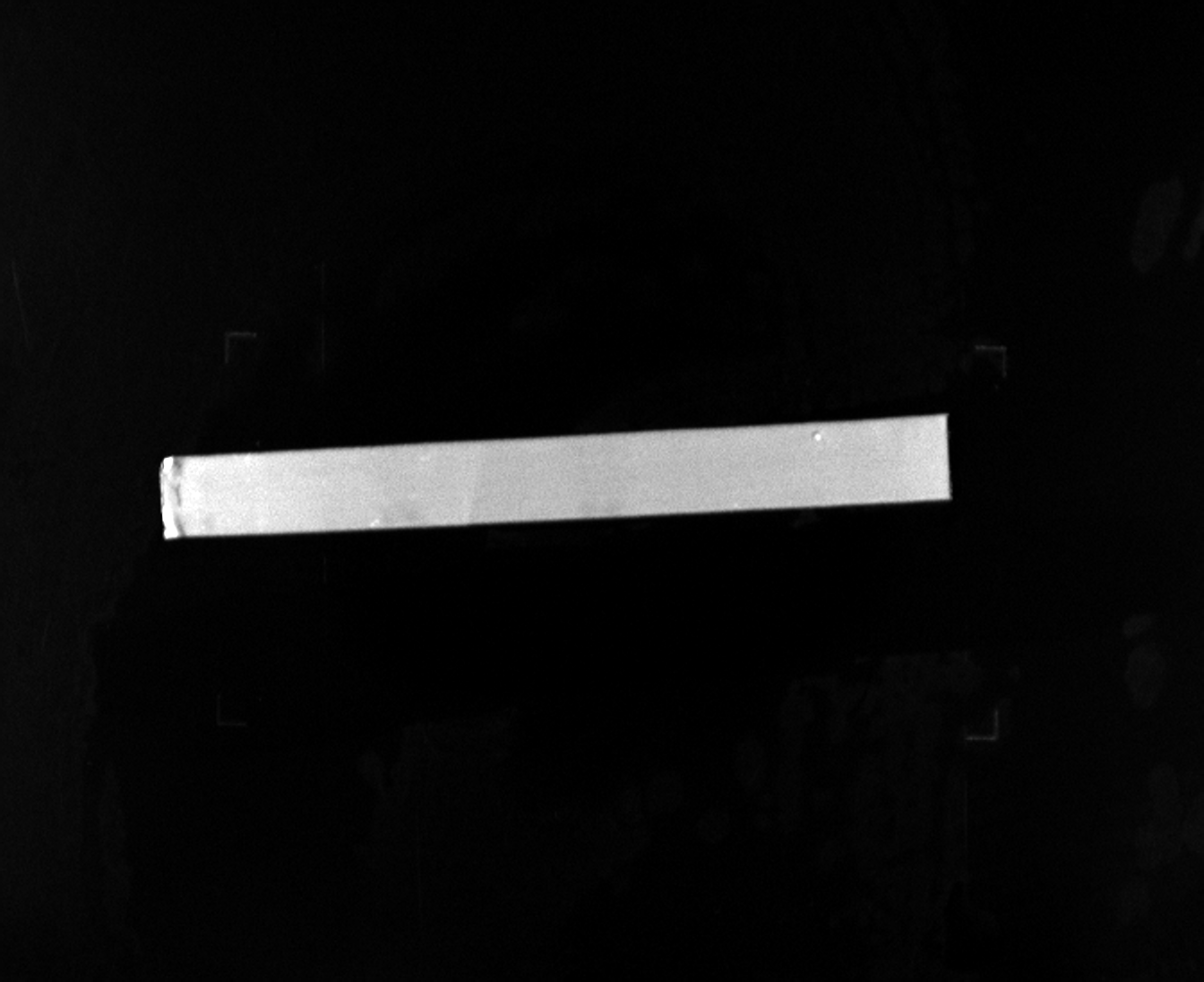

Supplement: Supplementary file 13 — Source Data EV Fig. 4 [file 44318_2023_3_MOESM13_ESM.zip › Figure EV4/4c/YTHDC1 w.Tif]

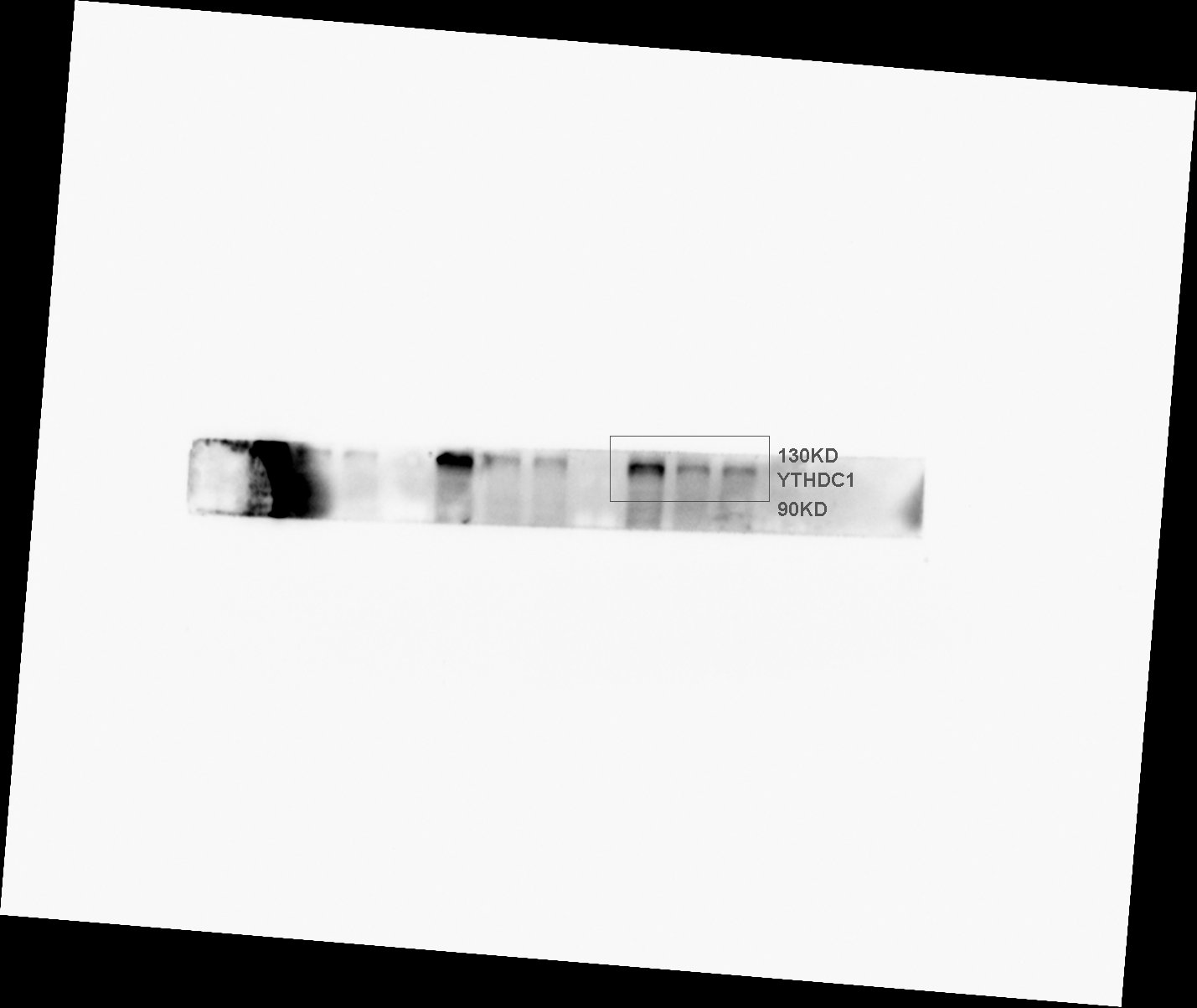

Supplement: Supplementary file 13 — Source Data EV Fig. 4 [file 44318_2023_3_MOESM13_ESM.zip › Figure EV4/4c/YTHDC1.jpg]

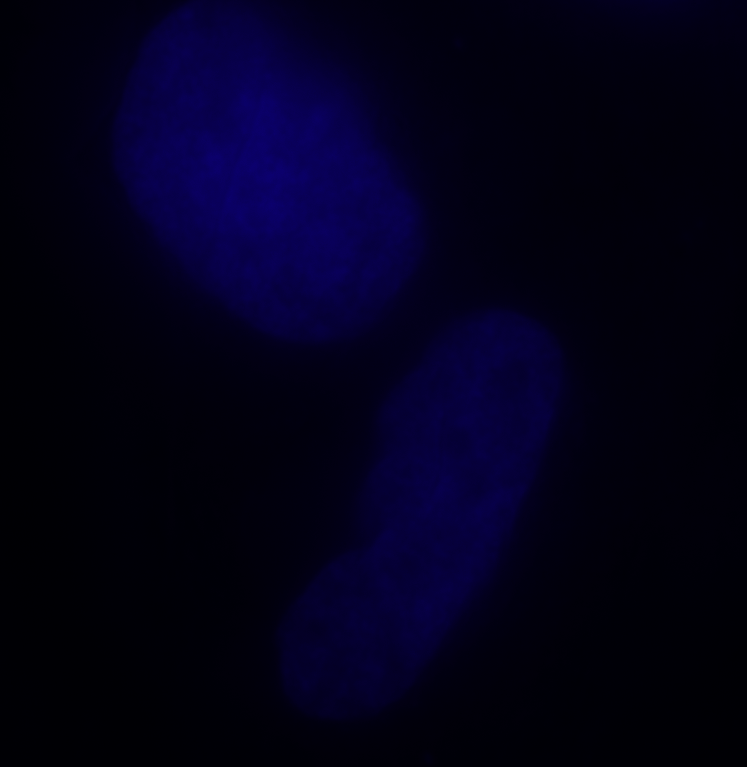

Supplement: Supplementary file 13 — Source Data EV Fig. 4 [file 44318_2023_3_MOESM13_ESM.zip › Figure EV4/4d-e/nc topbp1 IF/DAPI.tif]

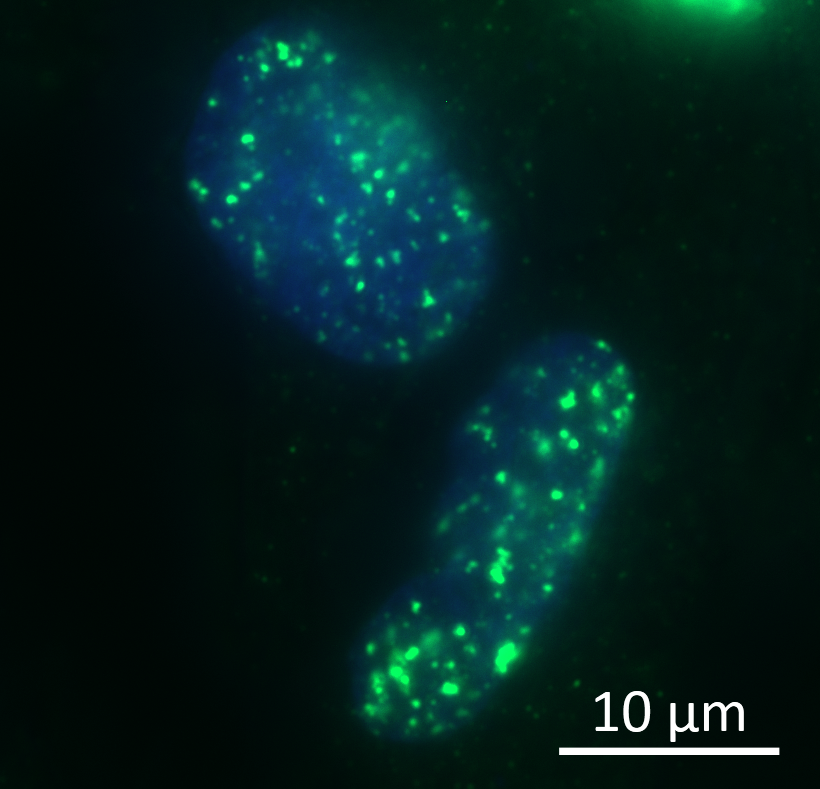

Supplement: Supplementary file 13 — Source Data EV Fig. 4 [file 44318_2023_3_MOESM13_ESM.zip › Figure EV4/4d-e/nc topbp1 IF/MERGE.tif]

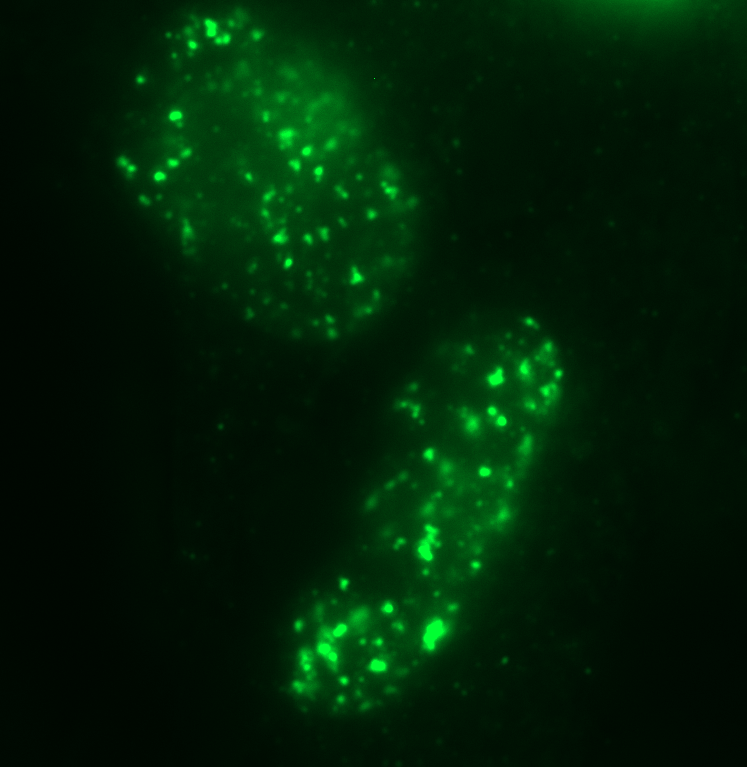

Supplement: Supplementary file 13 — Source Data EV Fig. 4 [file 44318_2023_3_MOESM13_ESM.zip › Figure EV4/4d-e/nc topbp1 IF/TopBP1.tif]

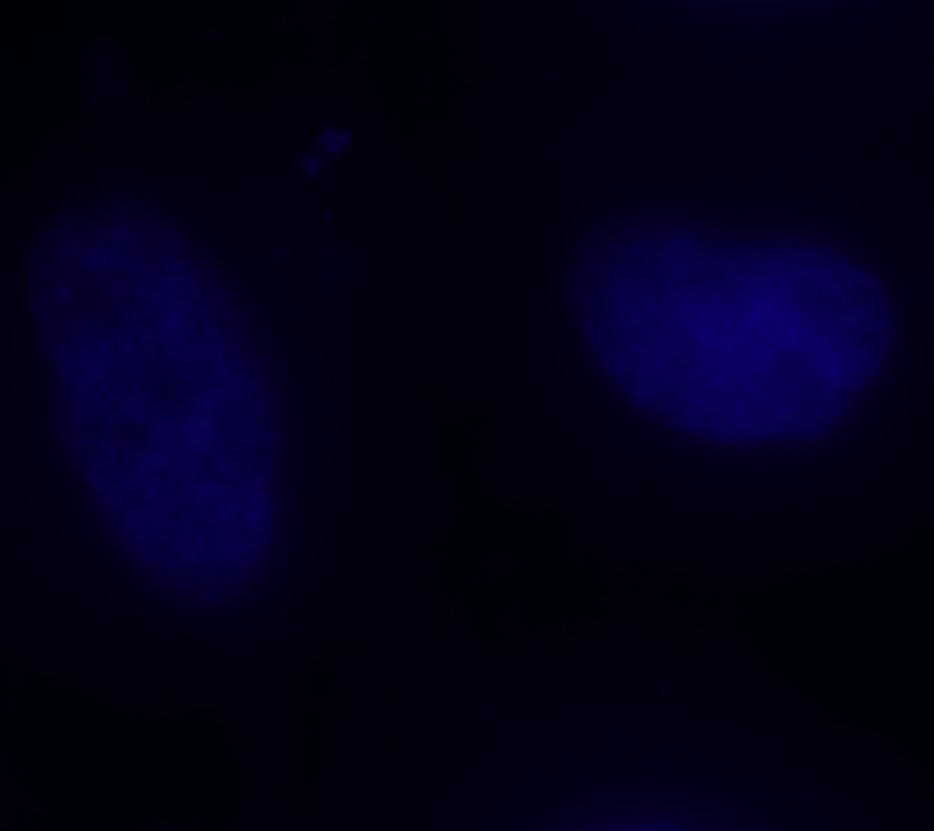

Supplement: Supplementary file 13 — Source Data EV Fig. 4 [file 44318_2023_3_MOESM13_ESM.zip › Figure EV4/4d-e/siYTHDC1-1 topbp1 IF/DAPI.tif]

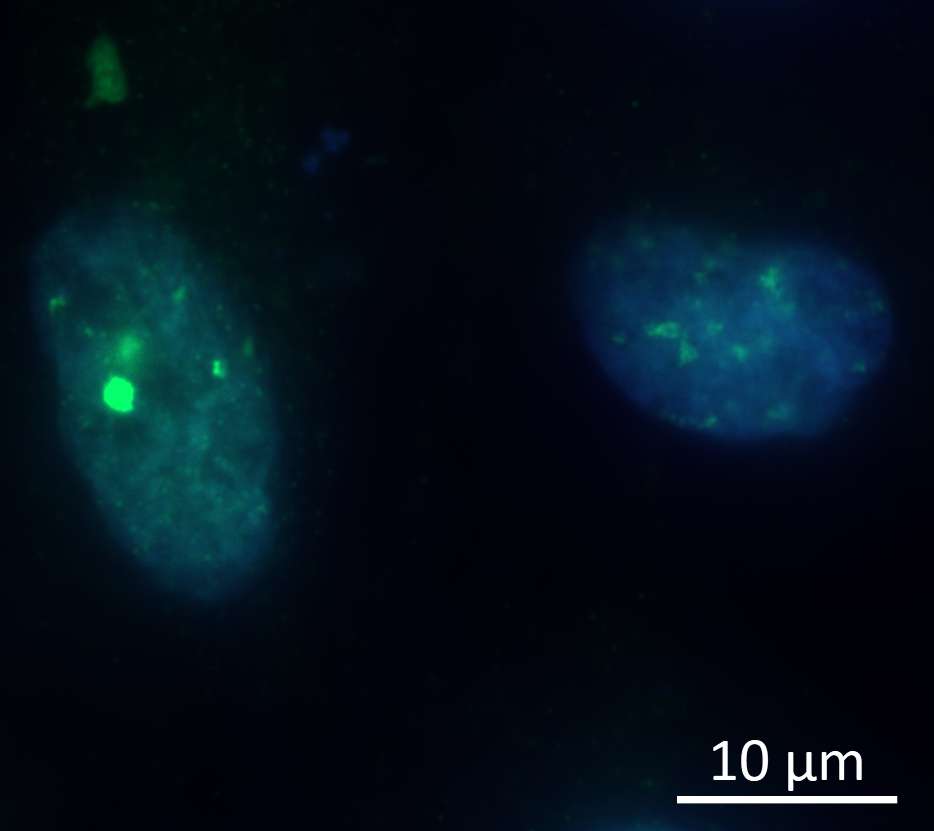

Supplement: Supplementary file 13 — Source Data EV Fig. 4 [file 44318_2023_3_MOESM13_ESM.zip › Figure EV4/4d-e/siYTHDC1-1 topbp1 IF/MERGE.tif]

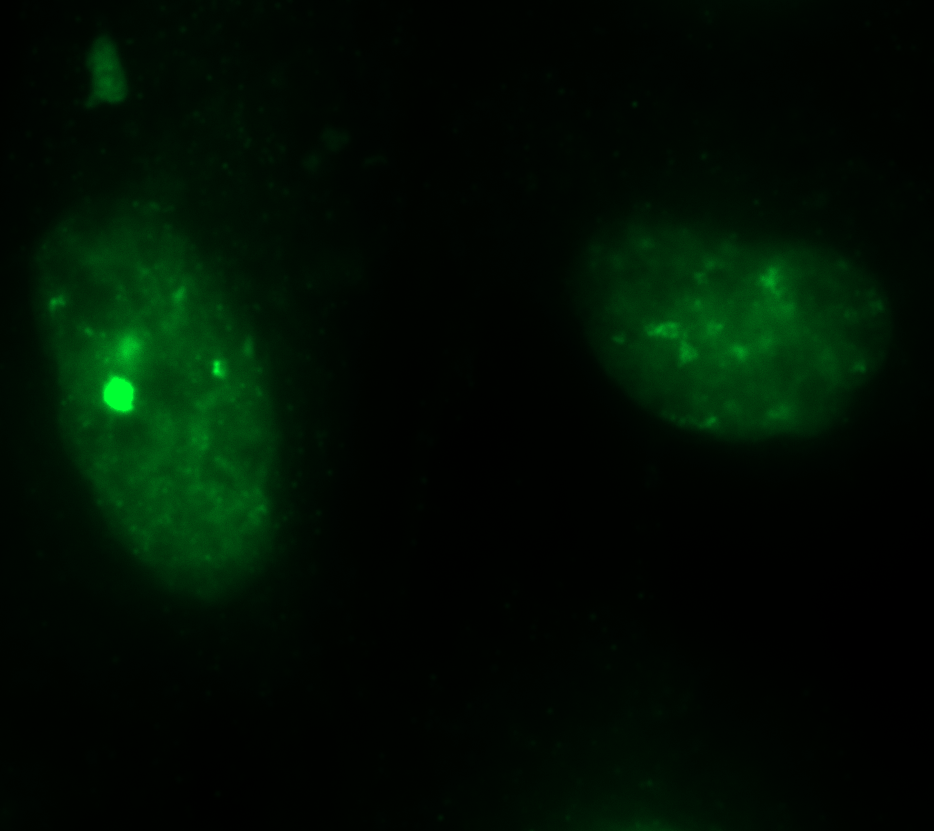

Supplement: Supplementary file 13 — Source Data EV Fig. 4 [file 44318_2023_3_MOESM13_ESM.zip › Figure EV4/4d-e/siYTHDC1-1 topbp1 IF/Topbp1.tif]

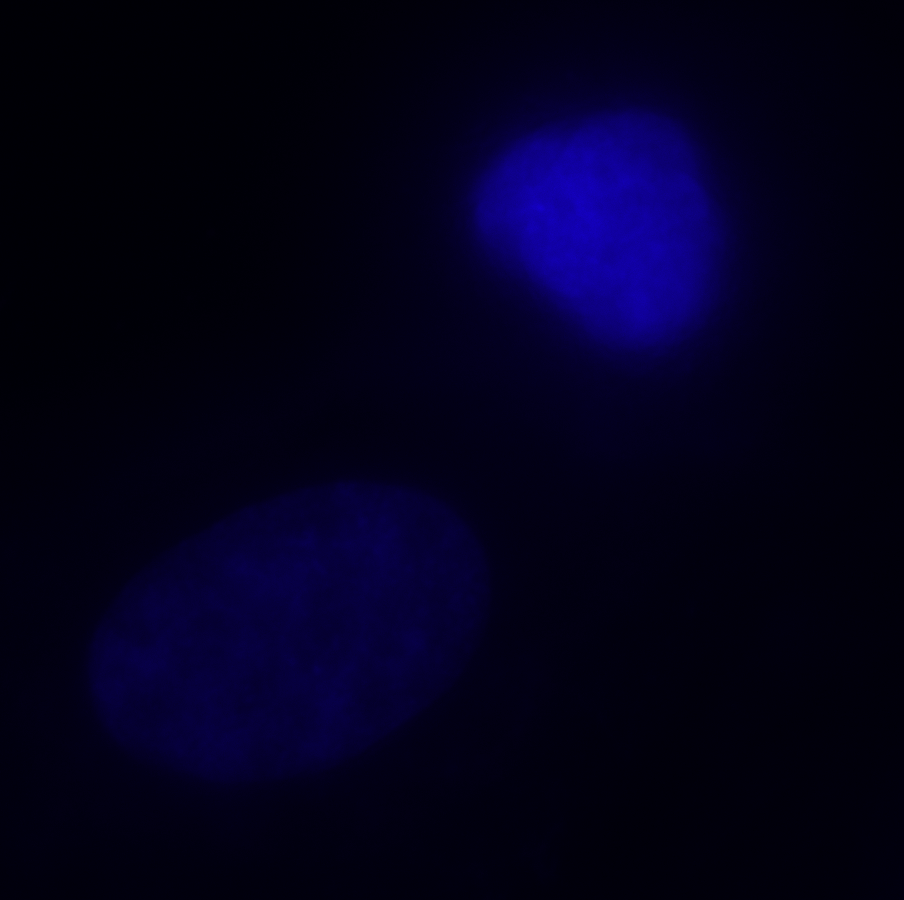

Supplement: Supplementary file 13 — Source Data EV Fig. 4 [file 44318_2023_3_MOESM13_ESM.zip › Figure EV4/4d-e/siYTHDC1-2 topbp1 IF/DAPI.tif]

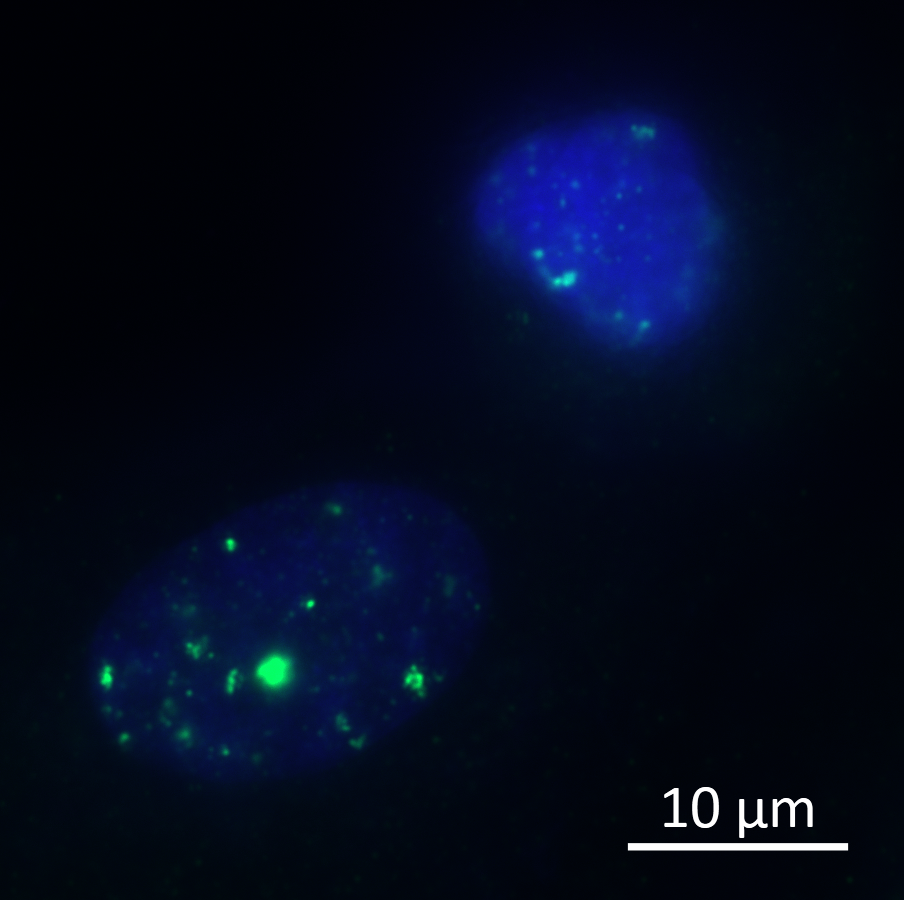

Supplement: Supplementary file 13 — Source Data EV Fig. 4 [file 44318_2023_3_MOESM13_ESM.zip › Figure EV4/4d-e/siYTHDC1-2 topbp1 IF/merge.tif]

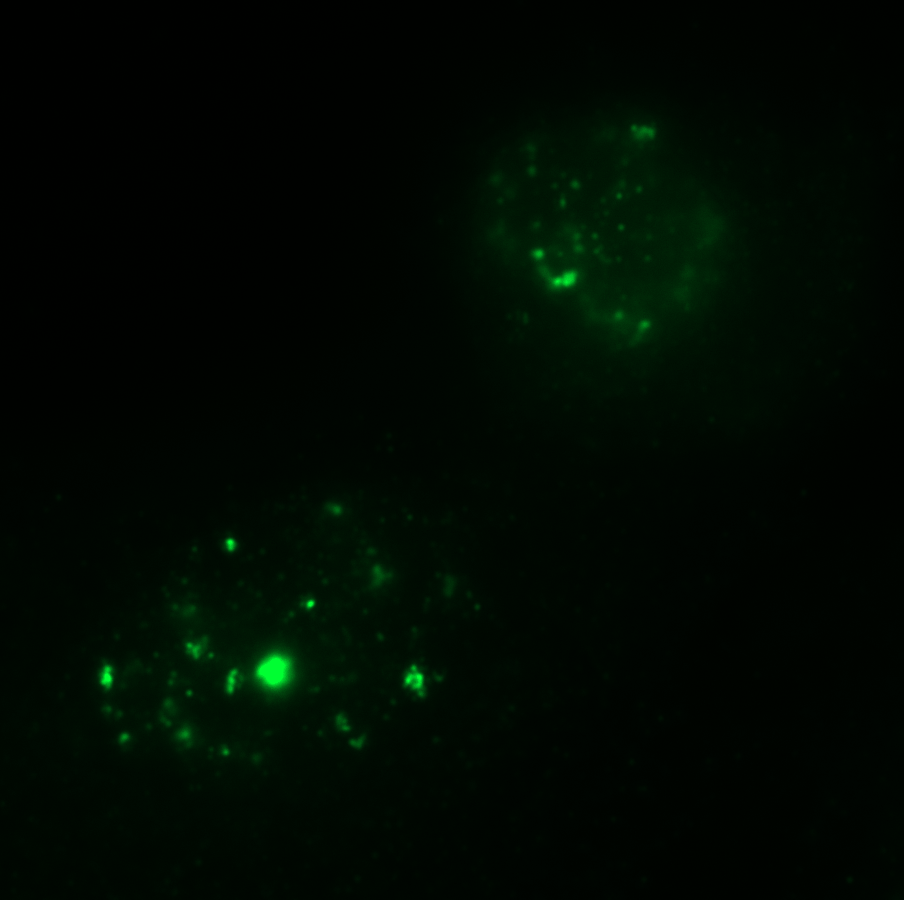

Supplement: Supplementary file 13 — Source Data EV Fig. 4 [file 44318_2023_3_MOESM13_ESM.zip › Figure EV4/4d-e/siYTHDC1-2 topbp1 IF/TopBP1.tif]

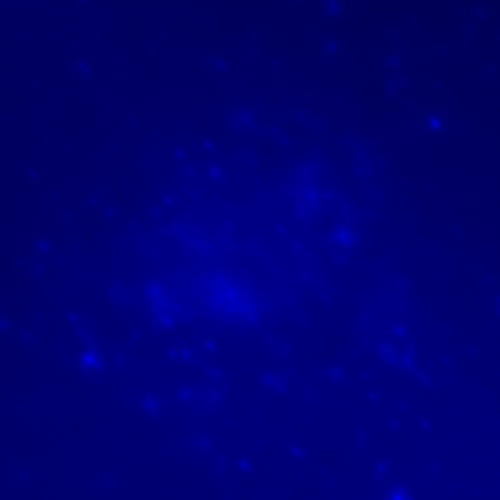

Supplement: Supplementary file 13 — Source Data EV Fig. 4 [file 44318_2023_3_MOESM13_ESM.zip › Figure EV4/4f-g/nc TopBP1 IF/DAPI.tif]

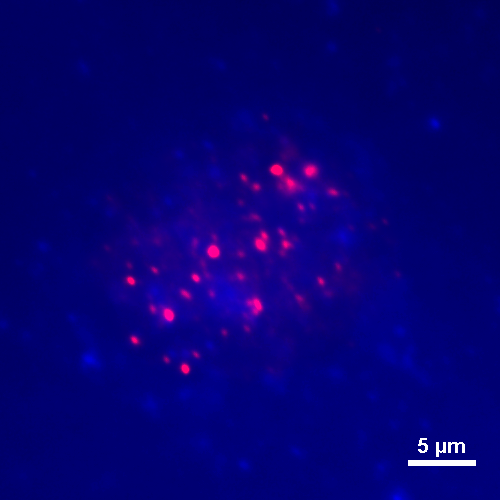

Supplement: Supplementary file 13 — Source Data EV Fig. 4 [file 44318_2023_3_MOESM13_ESM.zip › Figure EV4/4f-g/nc TopBP1 IF/MERGE.tif]

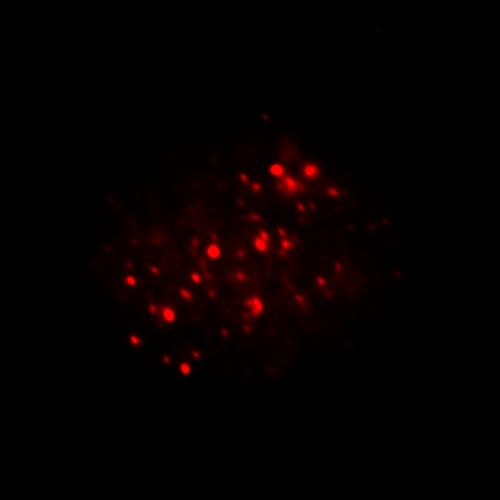

Supplement: Supplementary file 13 — Source Data EV Fig. 4 [file 44318_2023_3_MOESM13_ESM.zip › Figure EV4/4f-g/nc TopBP1 IF/TopBP1.tif]

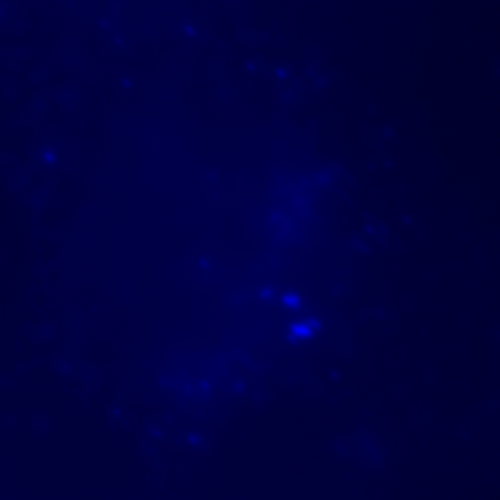

Supplement: Supplementary file 13 — Source Data EV Fig. 4 [file 44318_2023_3_MOESM13_ESM.zip › Figure EV4/4f-g/siRAD17 TopBP1 IF/DAPI.tif]

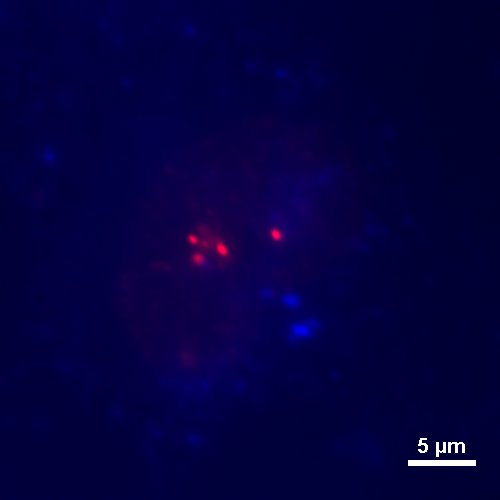

Supplement: Supplementary file 13 — Source Data EV Fig. 4 [file 44318_2023_3_MOESM13_ESM.zip › Figure EV4/4f-g/siRAD17 TopBP1 IF/MERGE.tif]

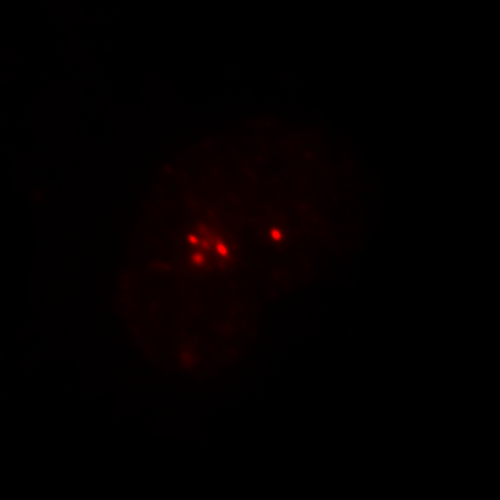

Supplement: Supplementary file 13 — Source Data EV Fig. 4 [file 44318_2023_3_MOESM13_ESM.zip › Figure EV4/4f-g/siRAD17 TopBP1 IF/Topbp1.tif]

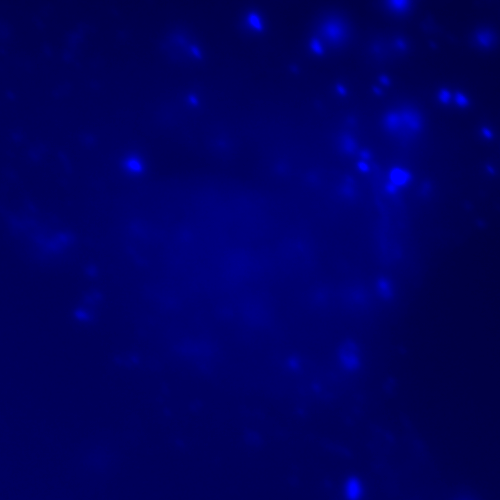

Supplement: Supplementary file 13 — Source Data EV Fig. 4 [file 44318_2023_3_MOESM13_ESM.zip › Figure EV4/4f-g/siRAD9A TopBP1 IF/DAPI.tif]

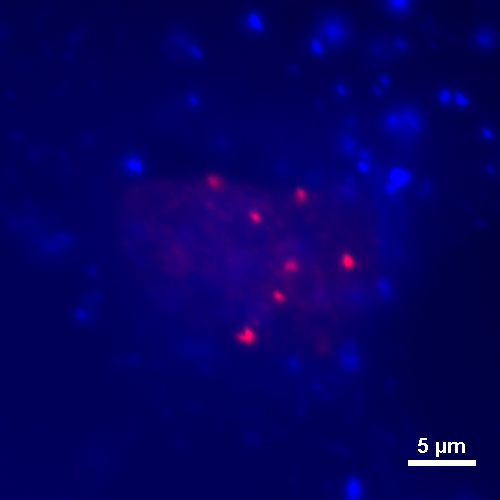

Supplement: Supplementary file 13 — Source Data EV Fig. 4 [file 44318_2023_3_MOESM13_ESM.zip › Figure EV4/4f-g/siRAD9A TopBP1 IF/merge.tif]

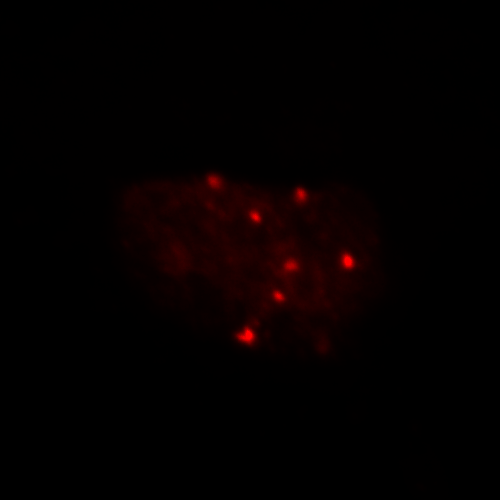

Supplement: Supplementary file 13 — Source Data EV Fig. 4 [file 44318_2023_3_MOESM13_ESM.zip › Figure EV4/4f-g/siRAD9A TopBP1 IF/TopBP1.tif]

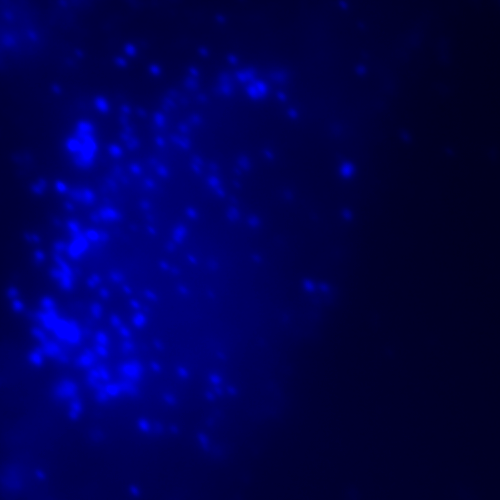

Supplement: Supplementary file 13 — Source Data EV Fig. 4 [file 44318_2023_3_MOESM13_ESM.zip › Figure EV4/4f-g/siYTHDC1 TopBP1 IF/DAPI.tif]

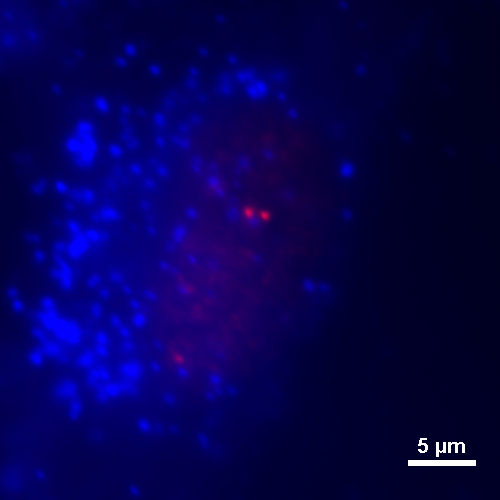

Supplement: Supplementary file 13 — Source Data EV Fig. 4 [file 44318_2023_3_MOESM13_ESM.zip › Figure EV4/4f-g/siYTHDC1 TopBP1 IF/MERGE.tif]

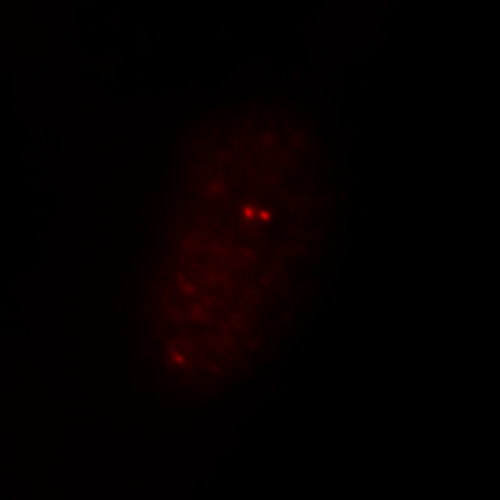

Supplement: Supplementary file 13 — Source Data EV Fig. 4 [file 44318_2023_3_MOESM13_ESM.zip › Figure EV4/4f-g/siYTHDC1 TopBP1 IF/TopBP1.tif]

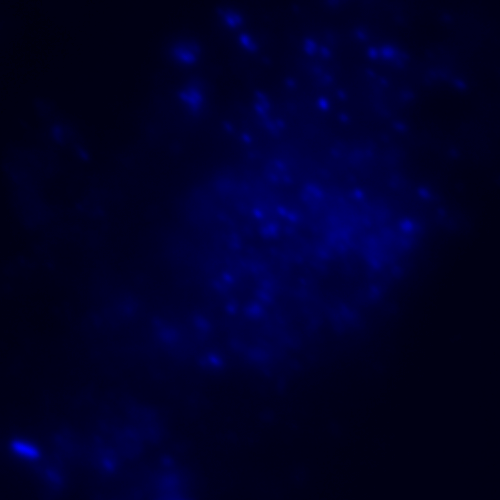

Supplement: Supplementary file 13 — Source Data EV Fig. 4 [file 44318_2023_3_MOESM13_ESM.zip › Figure EV4/4f-g/siYTHDC1-siRAD17 TopBP1 IF/DAPI.tif]
