# Supplementary figures and images for: YTHDC1 delays cellular senescence and pulmonary fibrosis by activating ATR in an m6A-independent manner (part 4 of 4)
Source: EMBO J. 2023 Dec 15;43(1):4. doi: 10.1038/s44318-023-00003-2 (PMC10883269; doi:10.1038/s44318-023-00003-2)

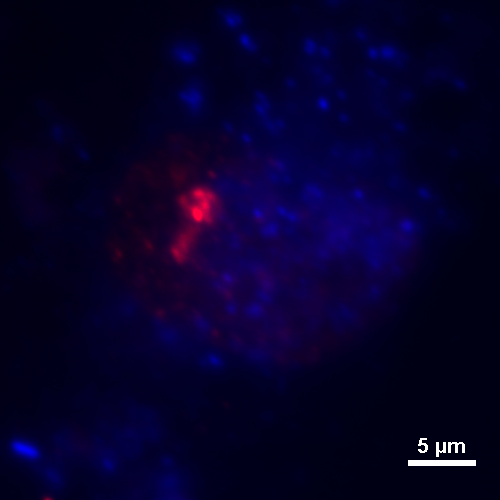

Supplement: Supplementary file 13 — Source Data EV Fig. 4 [file 44318_2023_3_MOESM13_ESM.zip › Figure EV4/4f-g/siYTHDC1-siRAD17 TopBP1 IF/MERGE.tif]

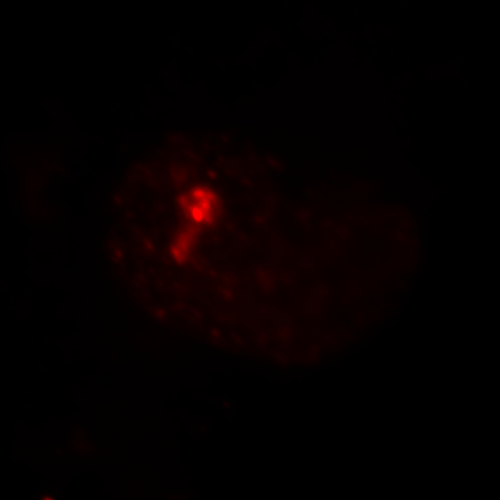

Supplement: Supplementary file 13 — Source Data EV Fig. 4 [file 44318_2023_3_MOESM13_ESM.zip › Figure EV4/4f-g/siYTHDC1-siRAD17 TopBP1 IF/TopBP1.tif]

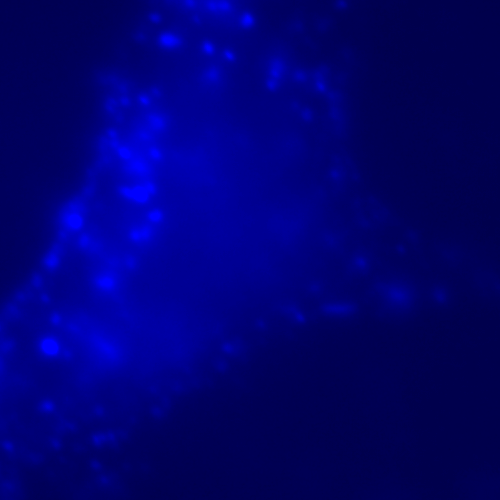

Supplement: Supplementary file 13 — Source Data EV Fig. 4 [file 44318_2023_3_MOESM13_ESM.zip › Figure EV4/4f-g/siYTHDC1-siRAD9A TopBP1 IF/DAPI.tif]

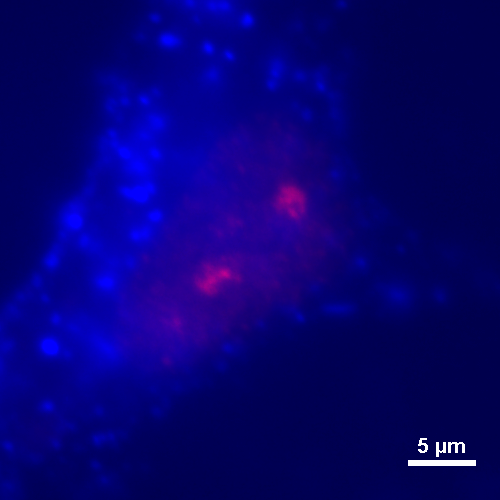

Supplement: Supplementary file 13 — Source Data EV Fig. 4 [file 44318_2023_3_MOESM13_ESM.zip › Figure EV4/4f-g/siYTHDC1-siRAD9A TopBP1 IF/MERGE.tif]

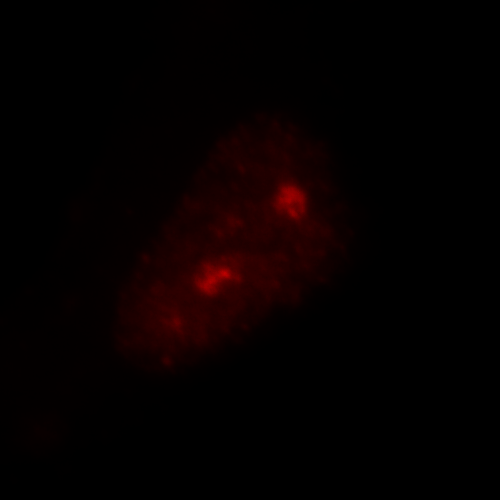

Supplement: Supplementary file 13 — Source Data EV Fig. 4 [file 44318_2023_3_MOESM13_ESM.zip › Figure EV4/4f-g/siYTHDC1-siRAD9A TopBP1 IF/TopBP1.tif]

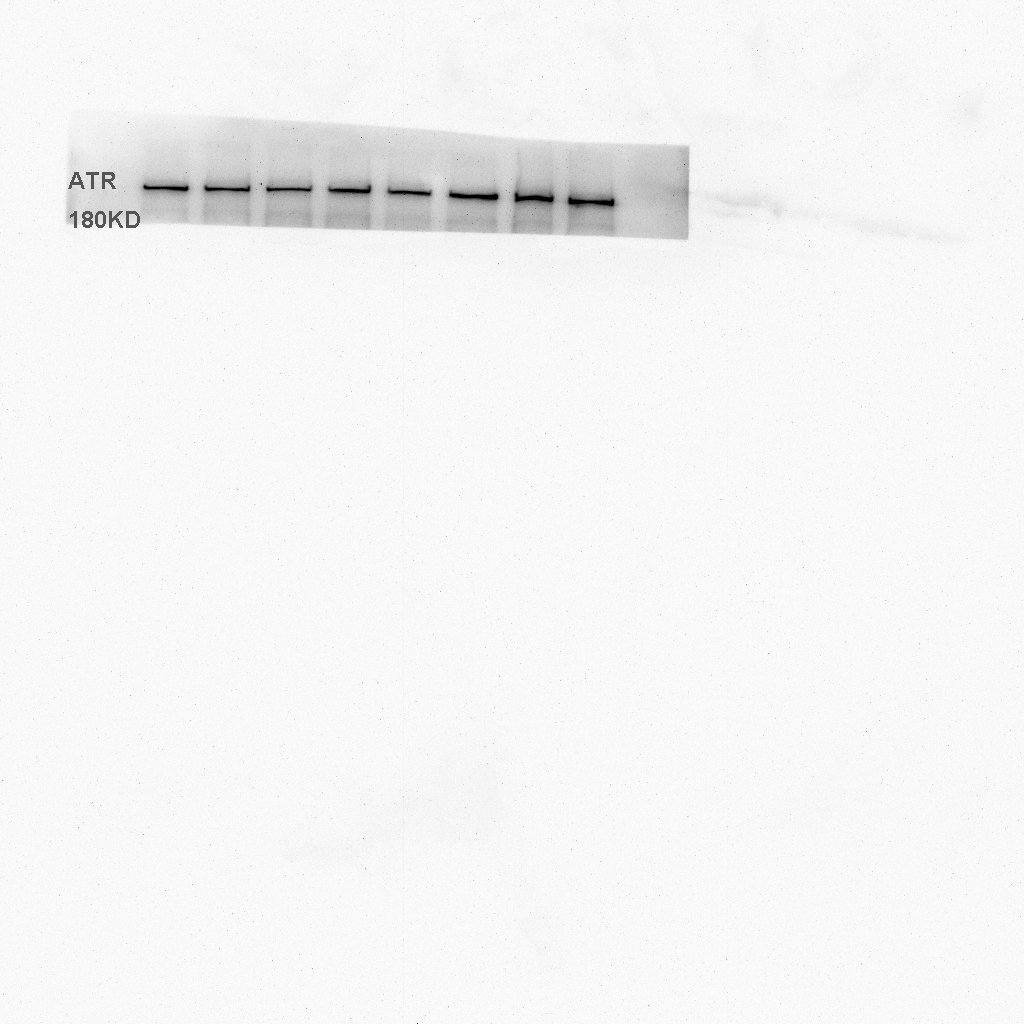

Supplement: Supplementary file 13 — Source Data EV Fig. 4 [file 44318_2023_3_MOESM13_ESM.zip › Figure EV4/4h/atr DMSO nc shDC1 siRAD9 siRAD17 shDC1 (siRAD9 siRAD17) siRAD9-siRAD17 .jpg]

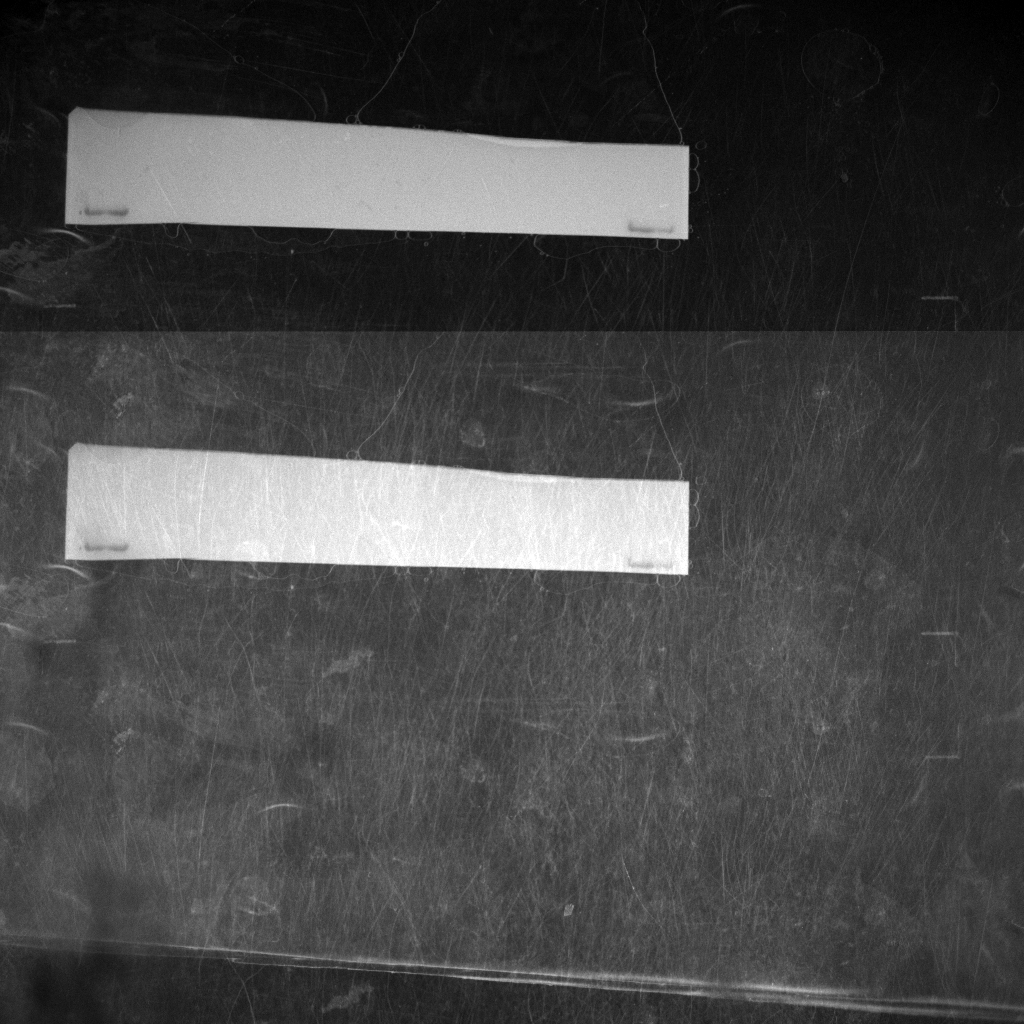

Supplement: Supplementary file 13 — Source Data EV Fig. 4 [file 44318_2023_3_MOESM13_ESM.zip › Figure EV4/4h/atr DMSO nc shDC1 siRAD9 siRAD17 shDC1 (siRAD9 siRAD17) siRAD9-siRAD17 w .tif]

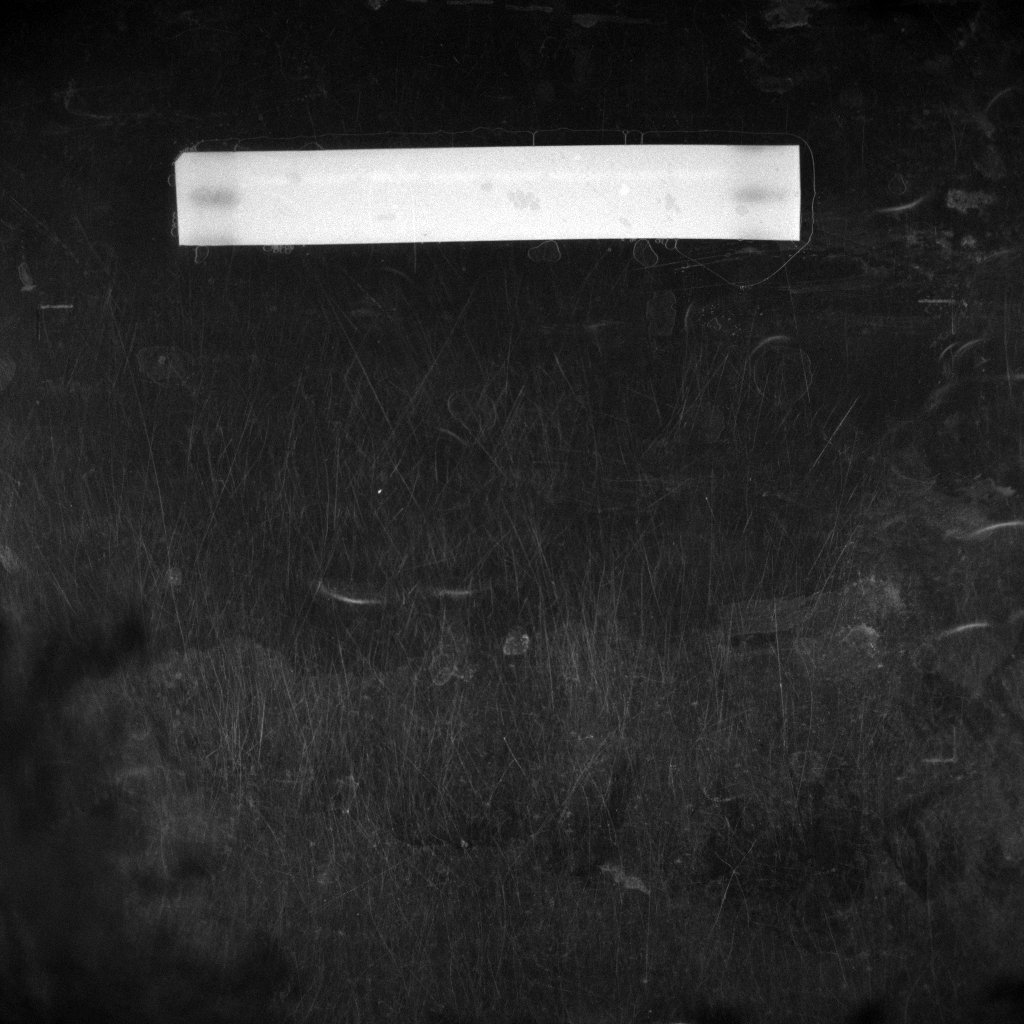

Supplement: Supplementary file 13 — Source Data EV Fig. 4 [file 44318_2023_3_MOESM13_ESM.zip › Figure EV4/4h/gapdh DMSO nc shDC1 siRAD9 siRAD17 shDC1 (siRAD9 siRAD17) siRAD9-siRAD17 W.tif]

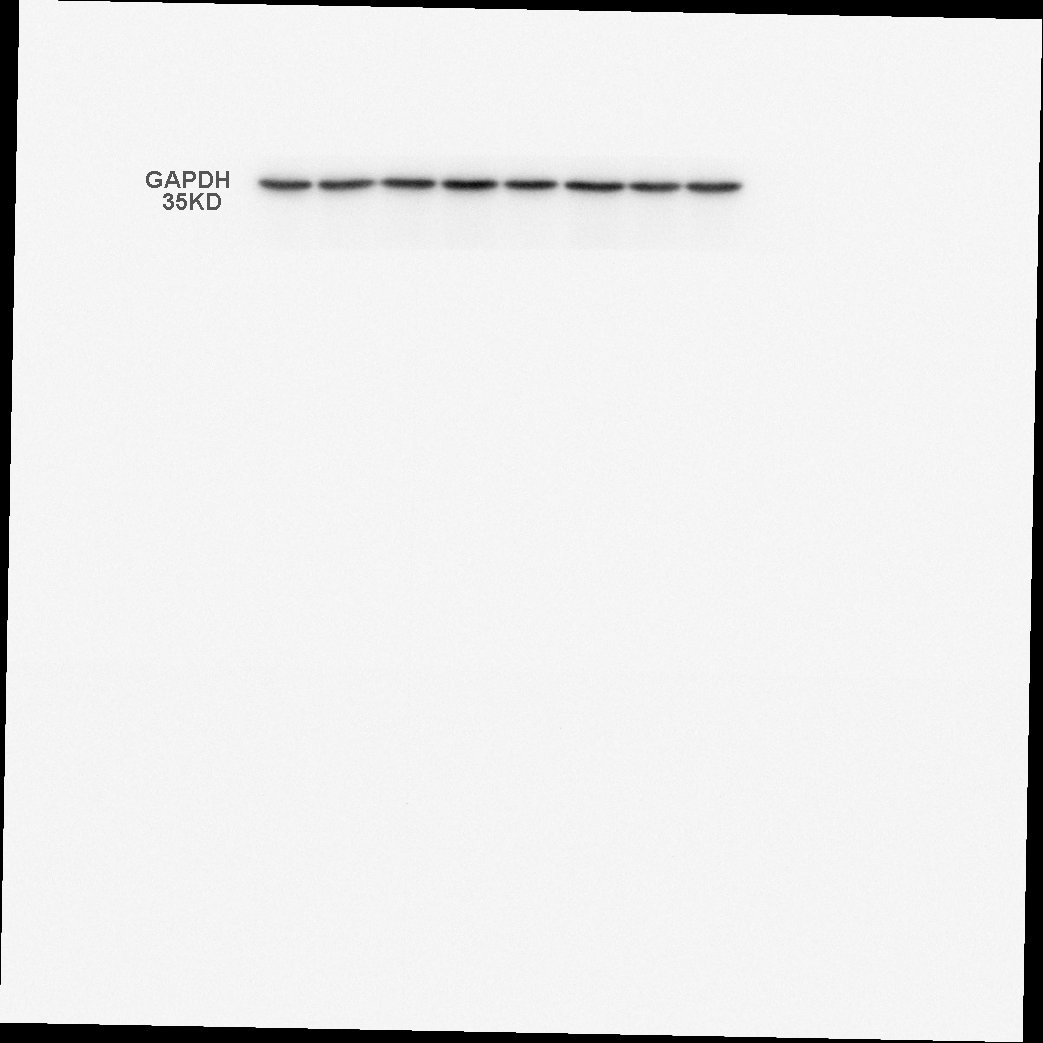

Supplement: Supplementary file 13 — Source Data EV Fig. 4 [file 44318_2023_3_MOESM13_ESM.zip › Figure EV4/4h/gapdh DMSO nc shDC1 siRAD9 siRAD17 shDC1 (siRAD9 siRAD17) siRAD9-siRAD17 .jpg]

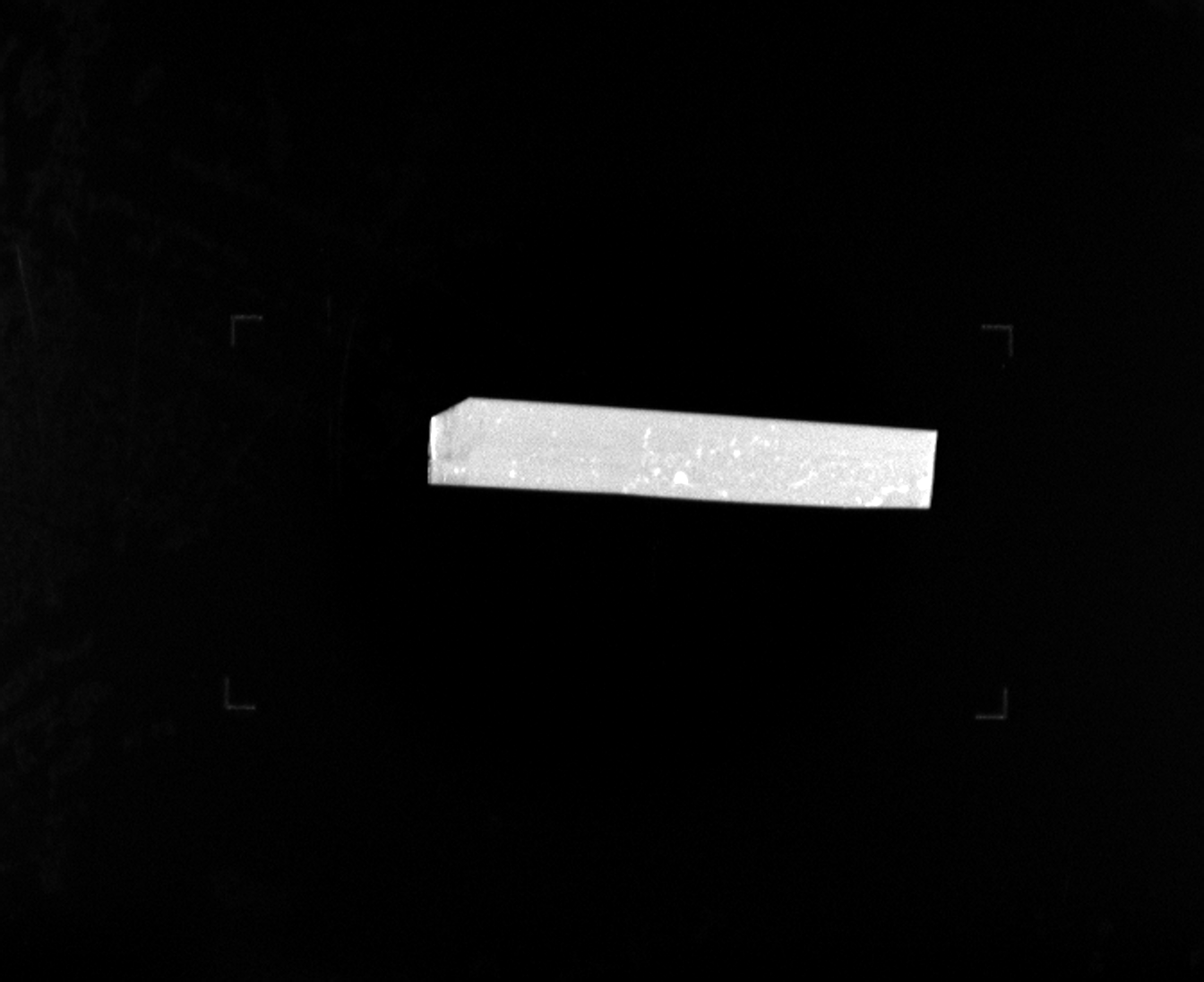

Supplement: Supplementary file 13 — Source Data EV Fig. 4 [file 44318_2023_3_MOESM13_ESM.zip › Figure EV4/4h/p-ATR DMSO nc shDC1 siRAD9 siRAD17 shDC1 (siRAD9 siRAD17) siRAD9-siRAD17 w.Tif]

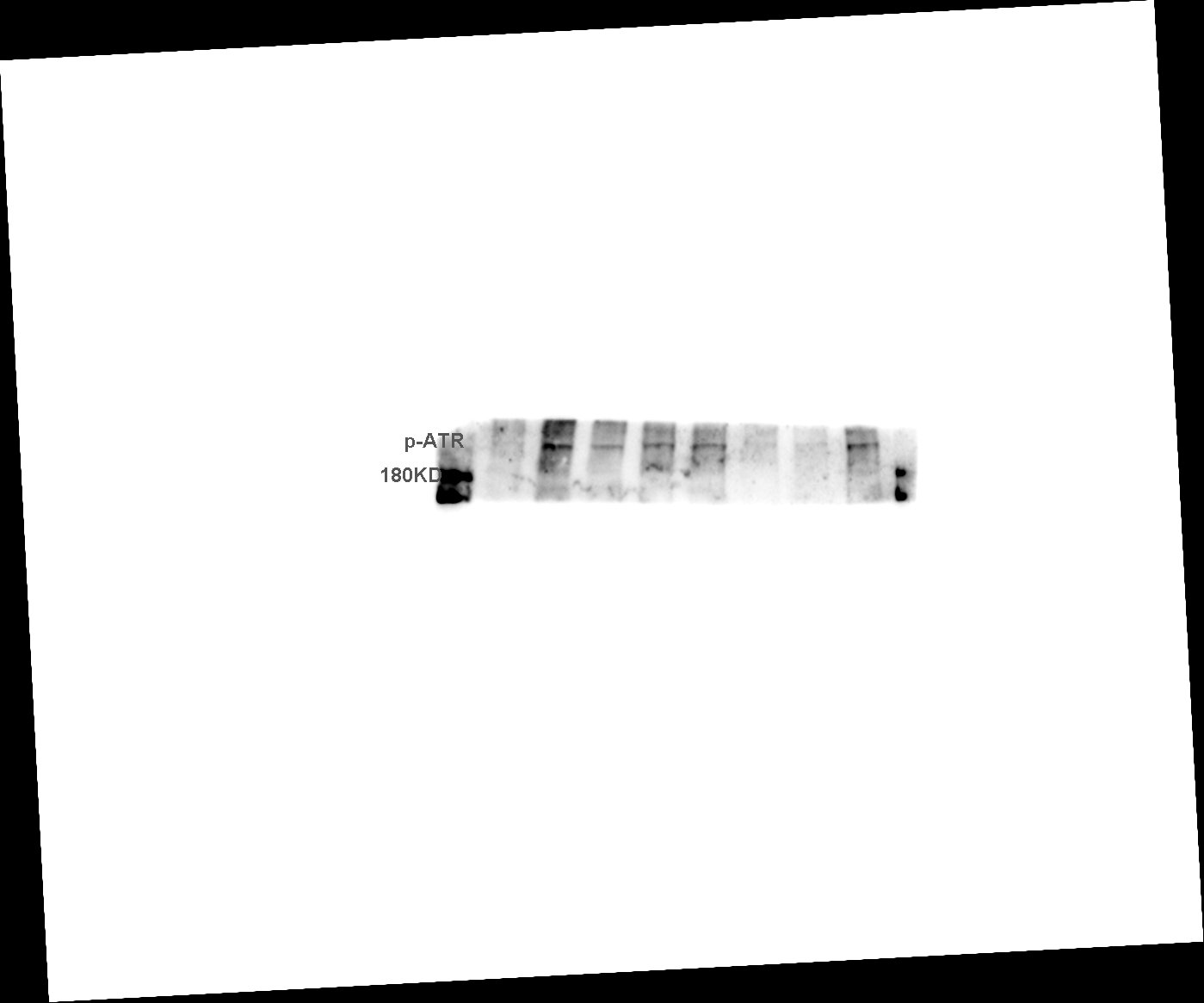

Supplement: Supplementary file 13 — Source Data EV Fig. 4 [file 44318_2023_3_MOESM13_ESM.zip › Figure EV4/4h/p-ATR DMSO nc shDC1 siRAD9 siRAD17 shDC1 (siRAD9 siRAD17) siRAD9-siRAD17.jpg]

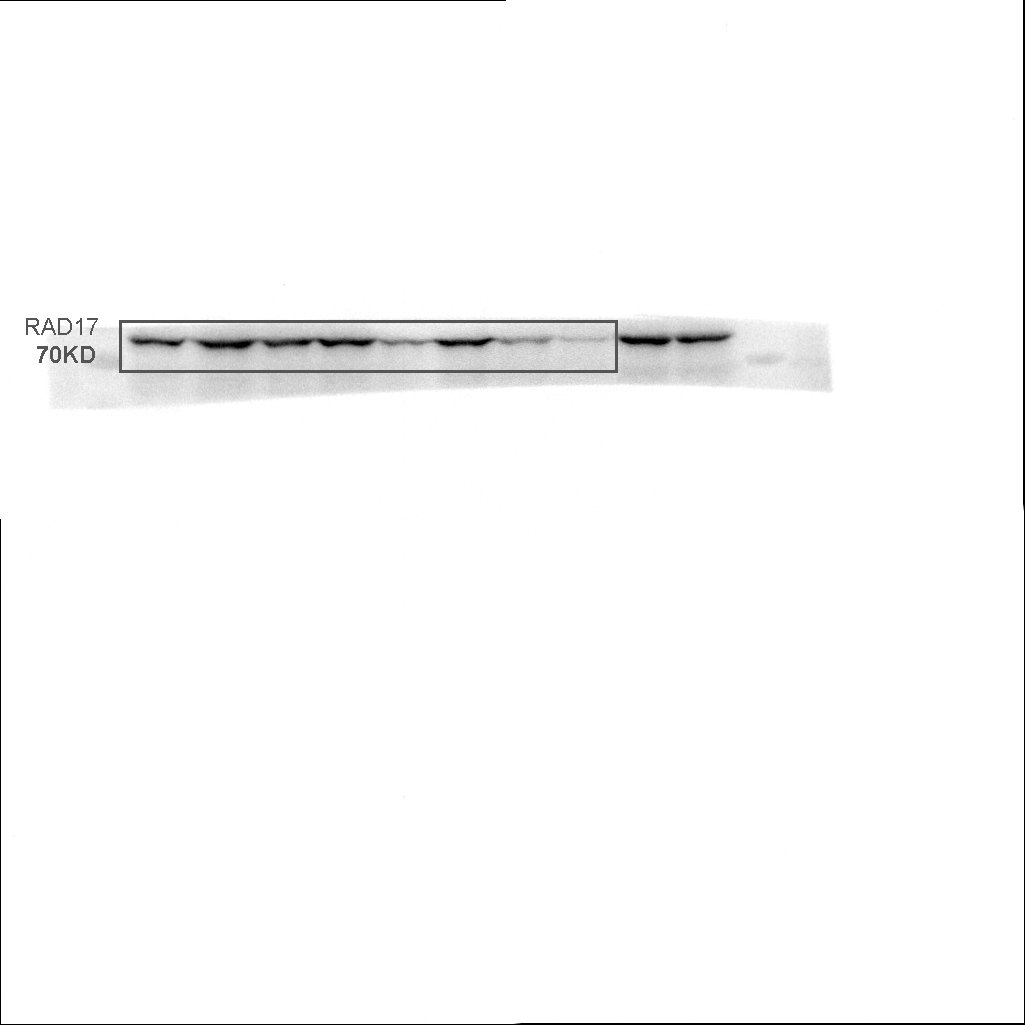

Supplement: Supplementary file 13 — Source Data EV Fig. 4 [file 44318_2023_3_MOESM13_ESM.zip › Figure EV4/4h/RAD17 NC siDC1 siRAD9 siRAD17 siDC1(siRAD9 siRAD17 ) siRAD9-siRAD17 siRBMX siDC1-siRBMX -1 .jpg]

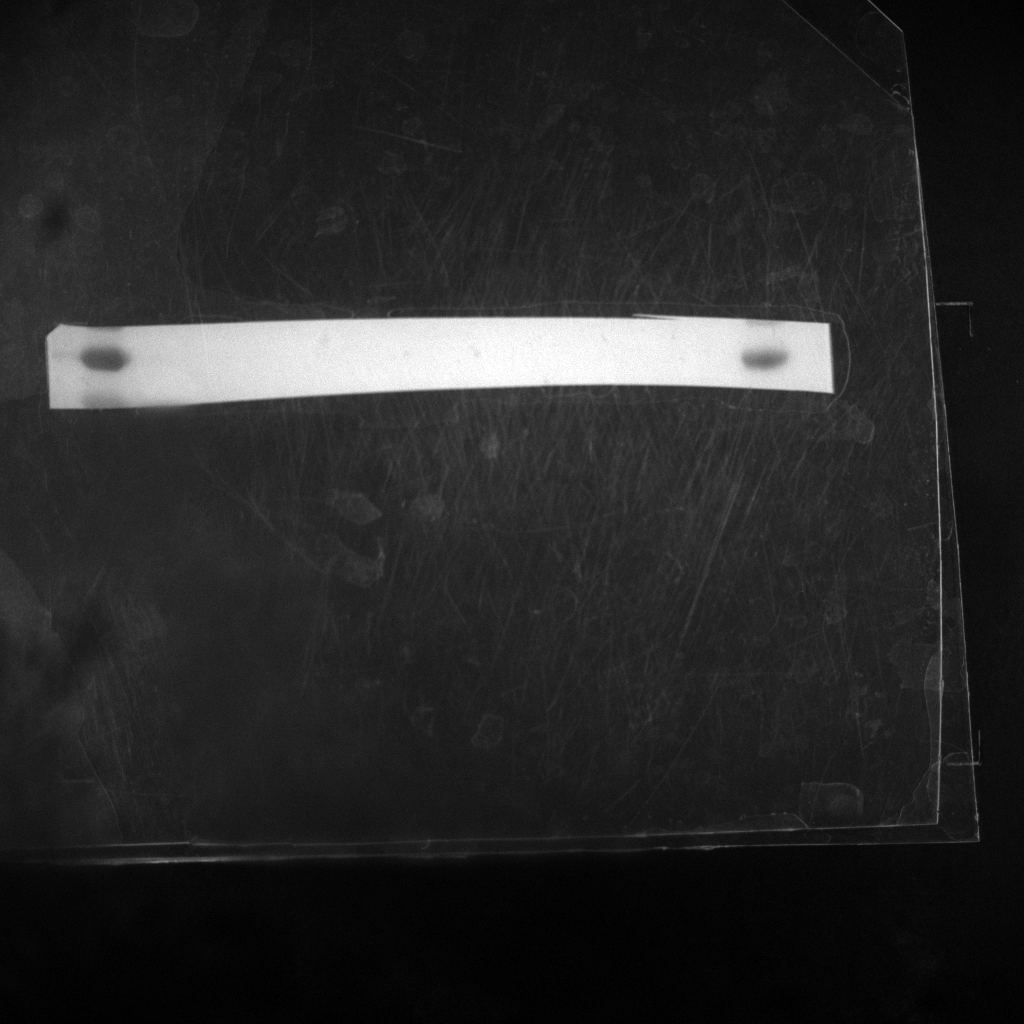

Supplement: Supplementary file 13 — Source Data EV Fig. 4 [file 44318_2023_3_MOESM13_ESM.zip › Figure EV4/4h/RAD17 NC siDC1 siRAD9 siRAD17 siDC1(siRAD9 siRAD17 ) siRAD9-siRAD17 siRBMX siDC1-siRBMX -1 WHITE .tif]

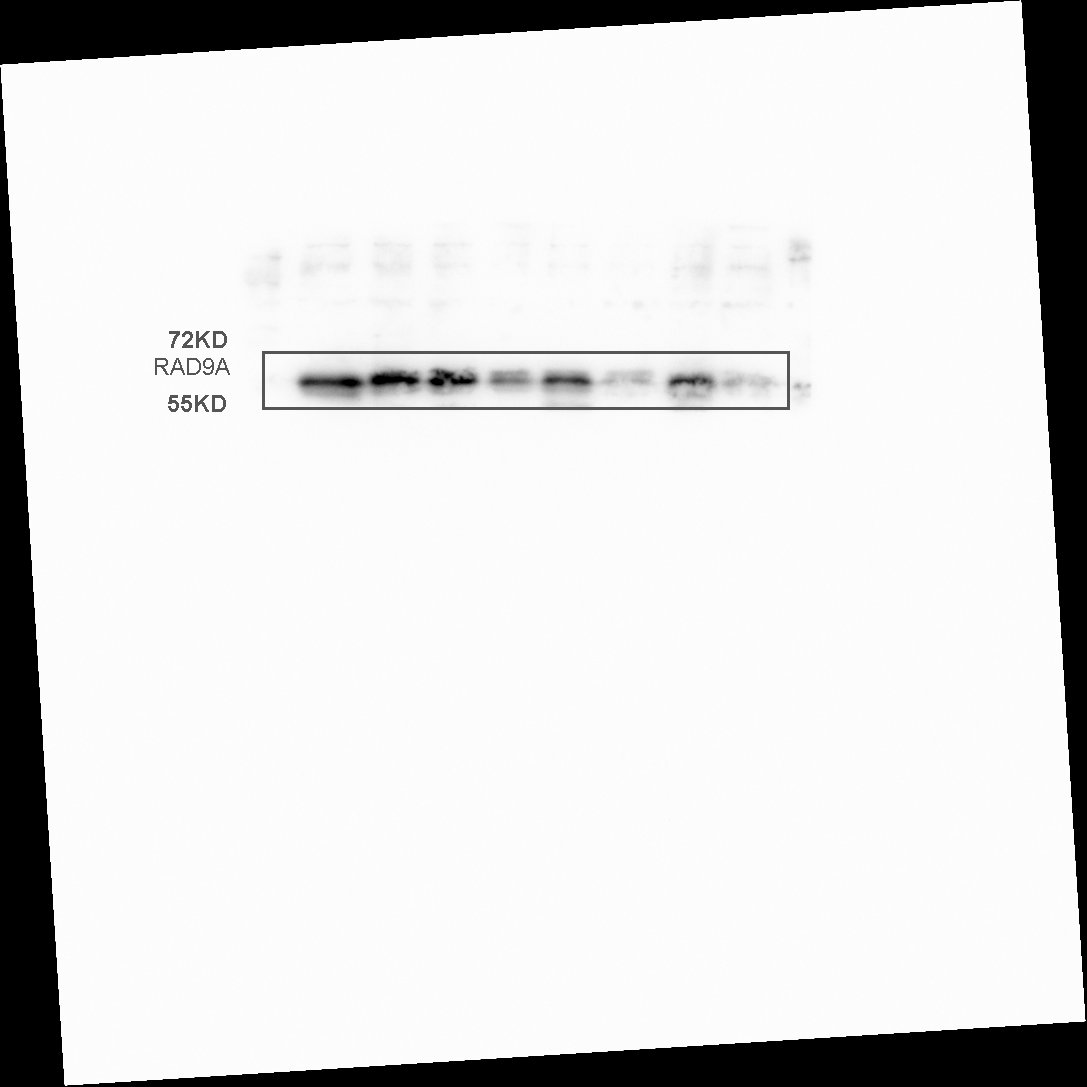

Supplement: Supplementary file 13 — Source Data EV Fig. 4 [file 44318_2023_3_MOESM13_ESM.zip › Figure EV4/4h/rad9 dmso NC siDC1 siRAD9 siRAD17 siDC1+(siRAD9 siRAD17 ) siRAD9+siRAD17 .jpg]

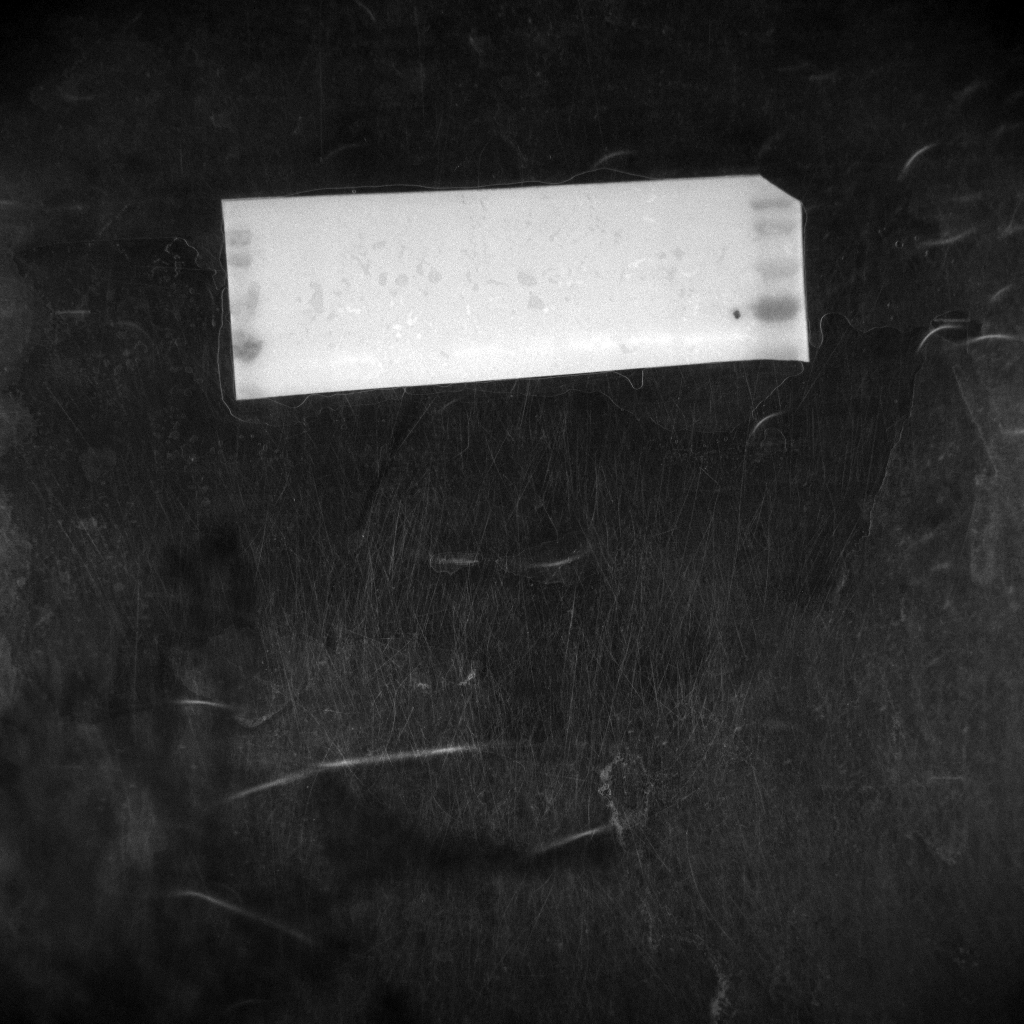

Supplement: Supplementary file 13 — Source Data EV Fig. 4 [file 44318_2023_3_MOESM13_ESM.zip › Figure EV4/4h/rad9 dmso NC siDC1 siRAD9 siRAD17 siDC1+(siRAD9 siRAD17 ) siRAD9+siRAD17 W.tif]

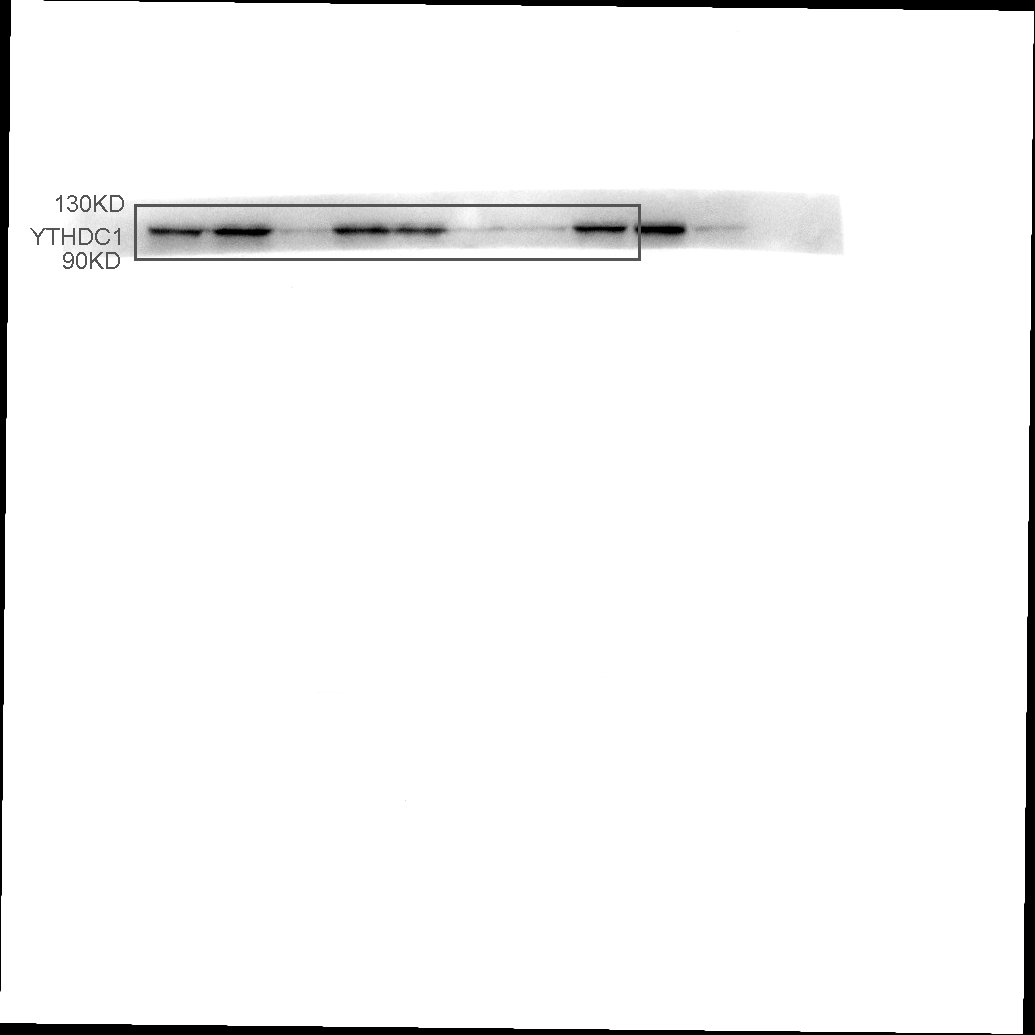

Supplement: Supplementary file 13 — Source Data EV Fig. 4 [file 44318_2023_3_MOESM13_ESM.zip › Figure EV4/4h/YTHDC1 NC siDC1 siRAD9 siRAD17 siDC1(siRAD9 siRAD17 ) siRAD9-siRAD17 siRBMX siDC1-siRBMX -1.jpg]

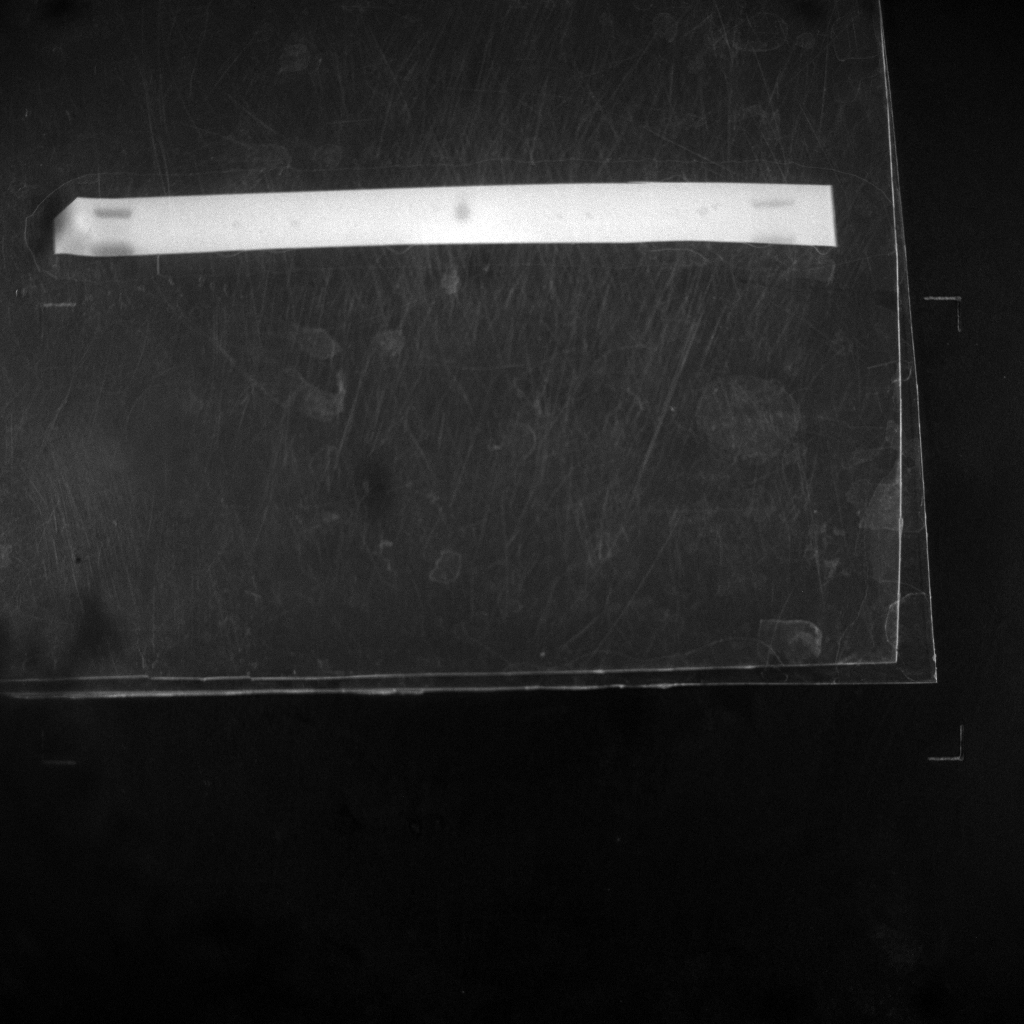

Supplement: Supplementary file 13 — Source Data EV Fig. 4 [file 44318_2023_3_MOESM13_ESM.zip › Figure EV4/4h/YTHDC1 NC siDC1 siRAD9 siRAD17 siDC1(siRAD9 siRAD17 ) siRAD9-siRAD17 siRBMX siDC1-siRBMX -1 W.tif]

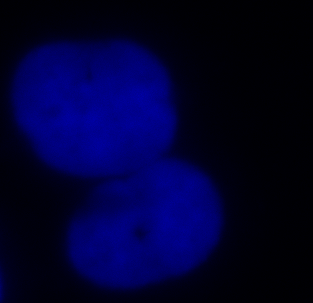

Supplement: Supplementary file 14 — Source Data EV Fig. 5 [file 44318_2023_3_MOESM14_ESM.zip › Figure EV5/5a-b/nc/DAPI.TIF]

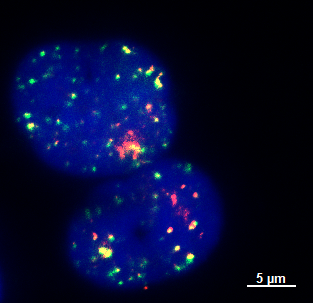

Supplement: Supplementary file 14 — Source Data EV Fig. 5 [file 44318_2023_3_MOESM14_ESM.zip › Figure EV5/5a-b/nc/MERGE.TIF]

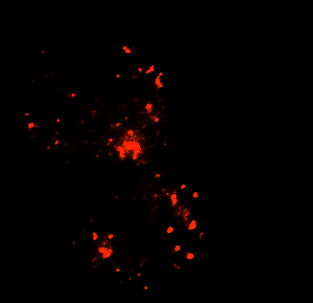

Supplement: Supplementary file 14 — Source Data EV Fig. 5 [file 44318_2023_3_MOESM14_ESM.zip › Figure EV5/5a-b/nc/MRE11.TIF]

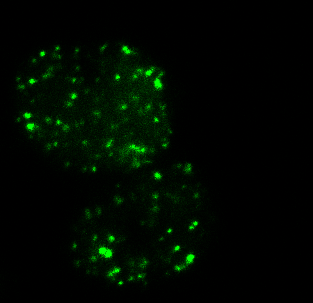

Supplement: Supplementary file 14 — Source Data EV Fig. 5 [file 44318_2023_3_MOESM14_ESM.zip › Figure EV5/5a-b/nc/yh2ax.TIF]

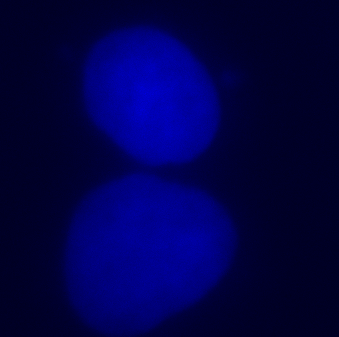

Supplement: Supplementary file 14 — Source Data EV Fig. 5 [file 44318_2023_3_MOESM14_ESM.zip › Figure EV5/5a-b/siYTHDC1-1/Image0002_DAPI.TIF]

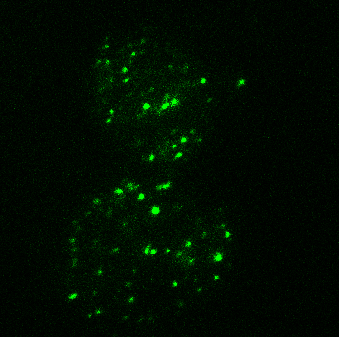

Supplement: Supplementary file 14 — Source Data EV Fig. 5 [file 44318_2023_3_MOESM14_ESM.zip › Figure EV5/5a-b/siYTHDC1-1/Image0002_FITC.TIF]

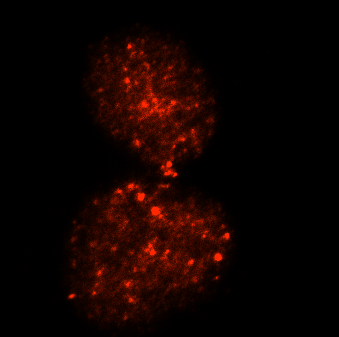

Supplement: Supplementary file 14 — Source Data EV Fig. 5 [file 44318_2023_3_MOESM14_ESM.zip › Figure EV5/5a-b/siYTHDC1-1/Image0002_Texasred.TIF]

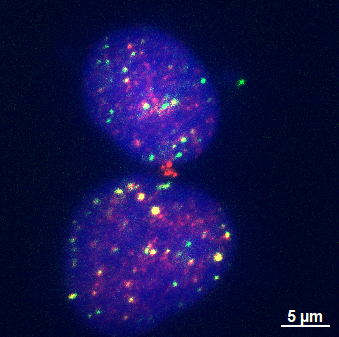

Supplement: Supplementary file 14 — Source Data EV Fig. 5 [file 44318_2023_3_MOESM14_ESM.zip › Figure EV5/5a-b/siYTHDC1-1/merge.TIF]

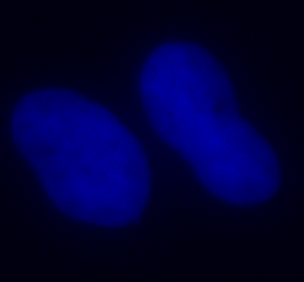

Supplement: Supplementary file 14 — Source Data EV Fig. 5 [file 44318_2023_3_MOESM14_ESM.zip › Figure EV5/5a-b/siYTHDC1-2/Image0004_DAPI.TIF]

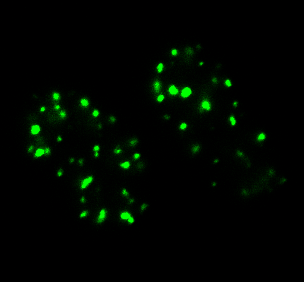

Supplement: Supplementary file 14 — Source Data EV Fig. 5 [file 44318_2023_3_MOESM14_ESM.zip › Figure EV5/5a-b/siYTHDC1-2/Image0004_FITC.TIF]

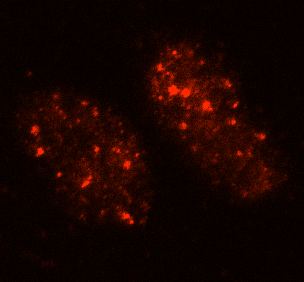

Supplement: Supplementary file 14 — Source Data EV Fig. 5 [file 44318_2023_3_MOESM14_ESM.zip › Figure EV5/5a-b/siYTHDC1-2/Image0004_Texasred.TIF]

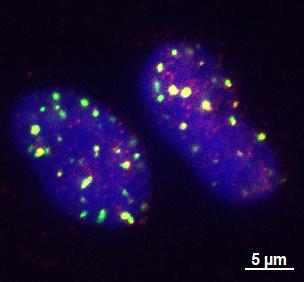

Supplement: Supplementary file 14 — Source Data EV Fig. 5 [file 44318_2023_3_MOESM14_ESM.zip › Figure EV5/5a-b/siYTHDC1-2/merge.TIF]

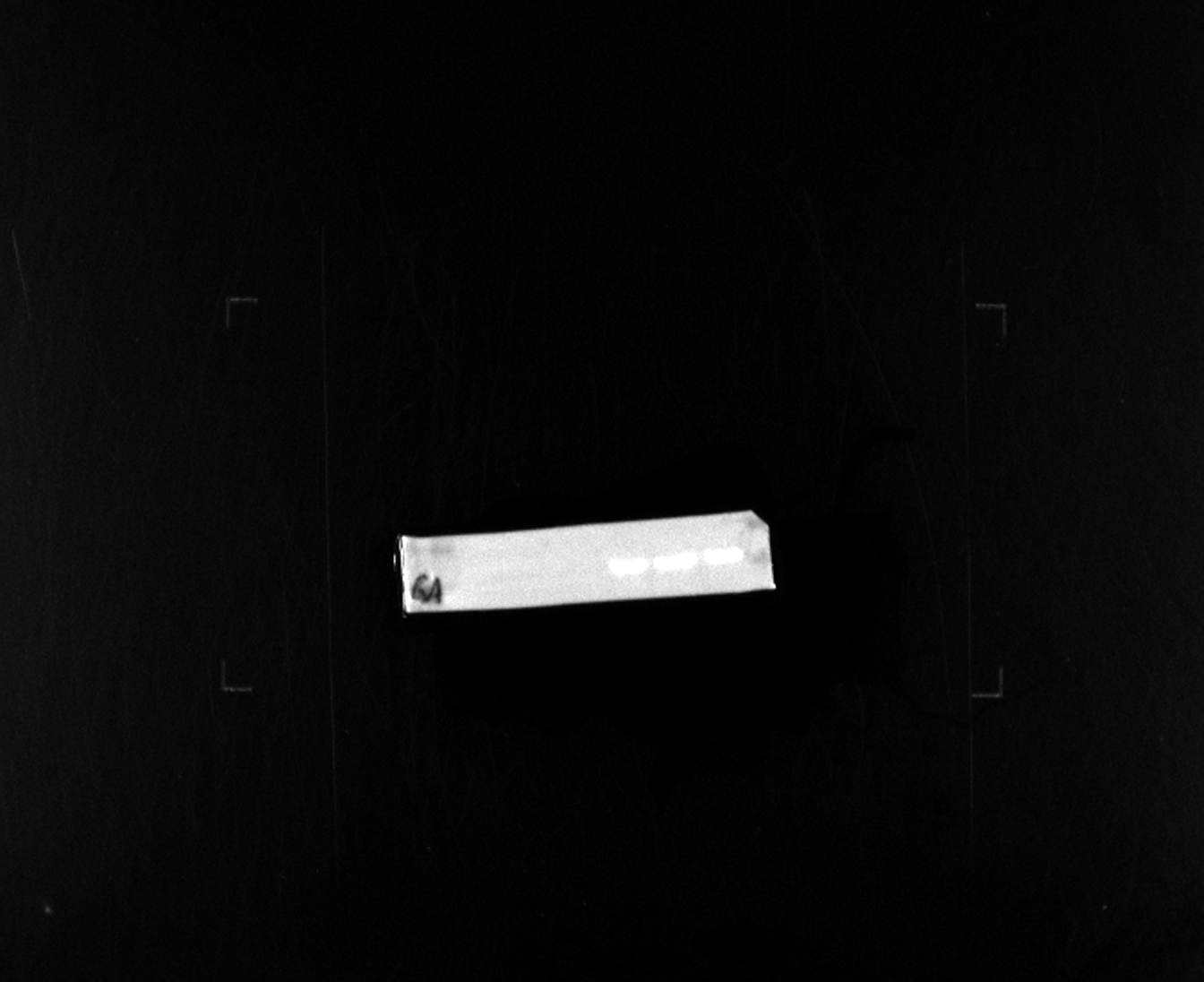

Supplement: Supplementary file 14 — Source Data EV Fig. 5 [file 44318_2023_3_MOESM14_ESM.zip › Figure EV5/5c/gapdh v TOP-IP-blm (nc siDC1) (input IP) 3 W.Tif]

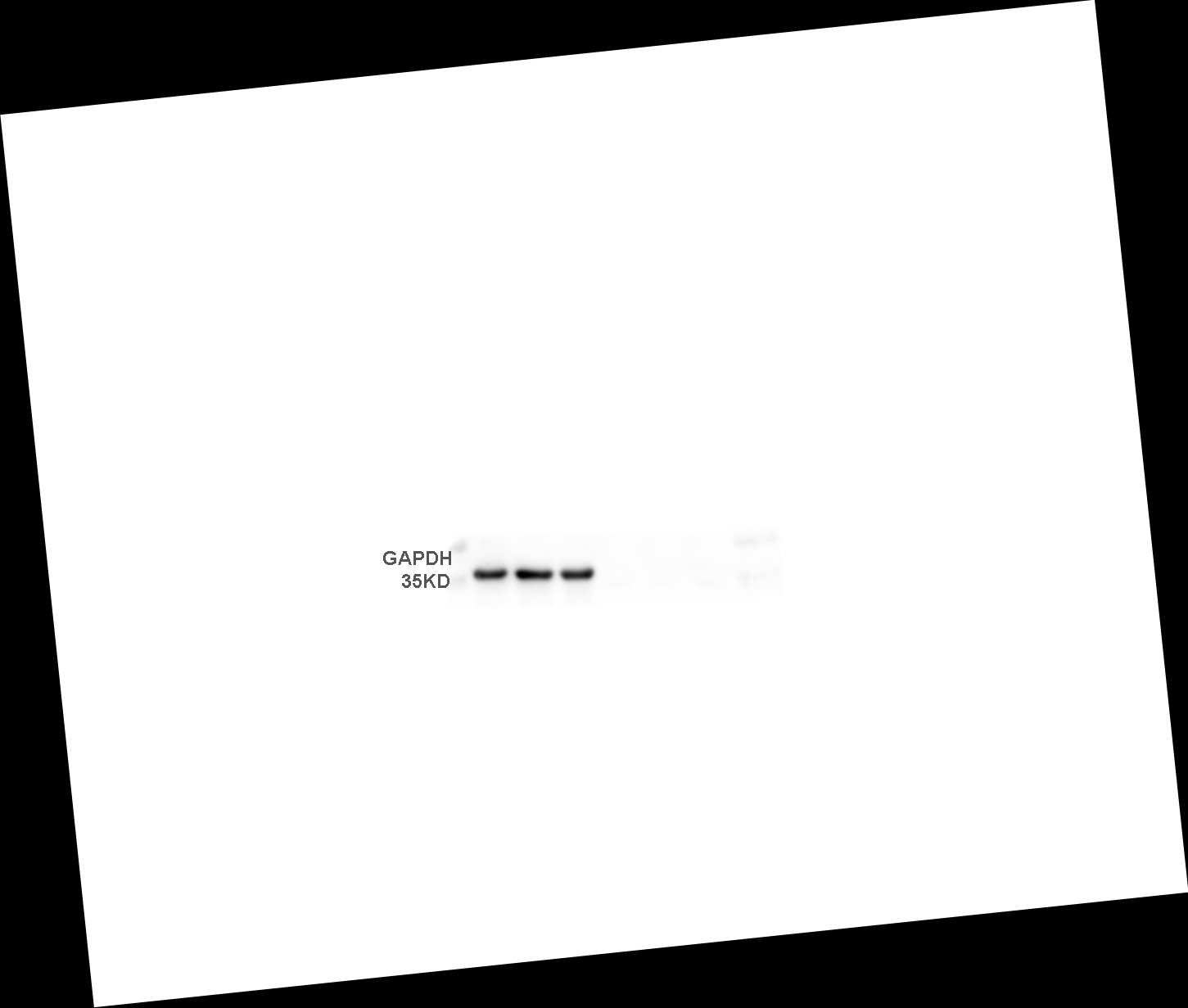

Supplement: Supplementary file 14 — Source Data EV Fig. 5 [file 44318_2023_3_MOESM14_ESM.zip › Figure EV5/5c/gapdh v TOP-IP-blm (nc siDC1) (input IP) 3.jpg]

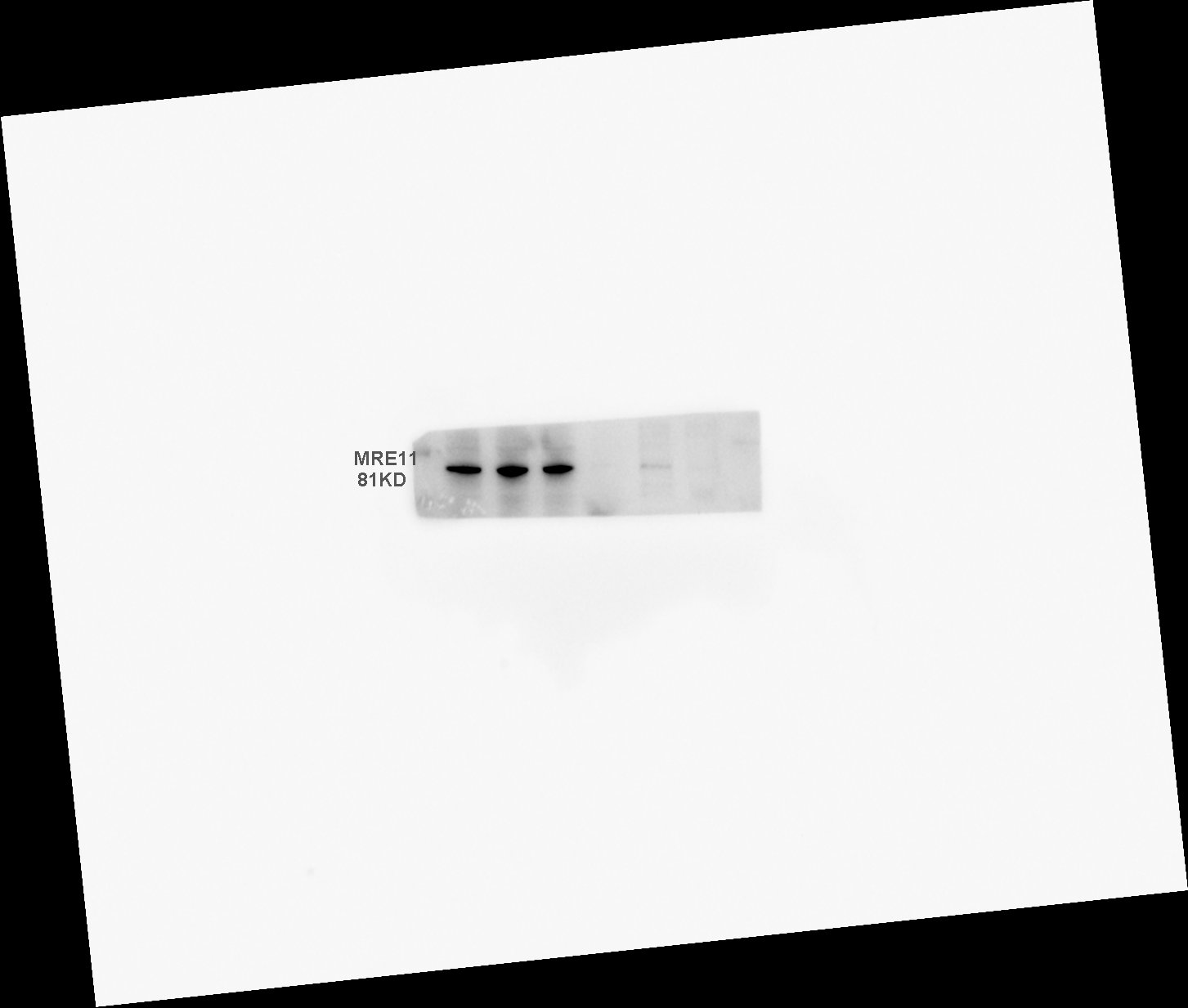

Supplement: Supplementary file 14 — Source Data EV Fig. 5 [file 44318_2023_3_MOESM14_ESM.zip › Figure EV5/5c/mre11 v TOP-IP-blm (nc siDC1) (input IP) .jpg]

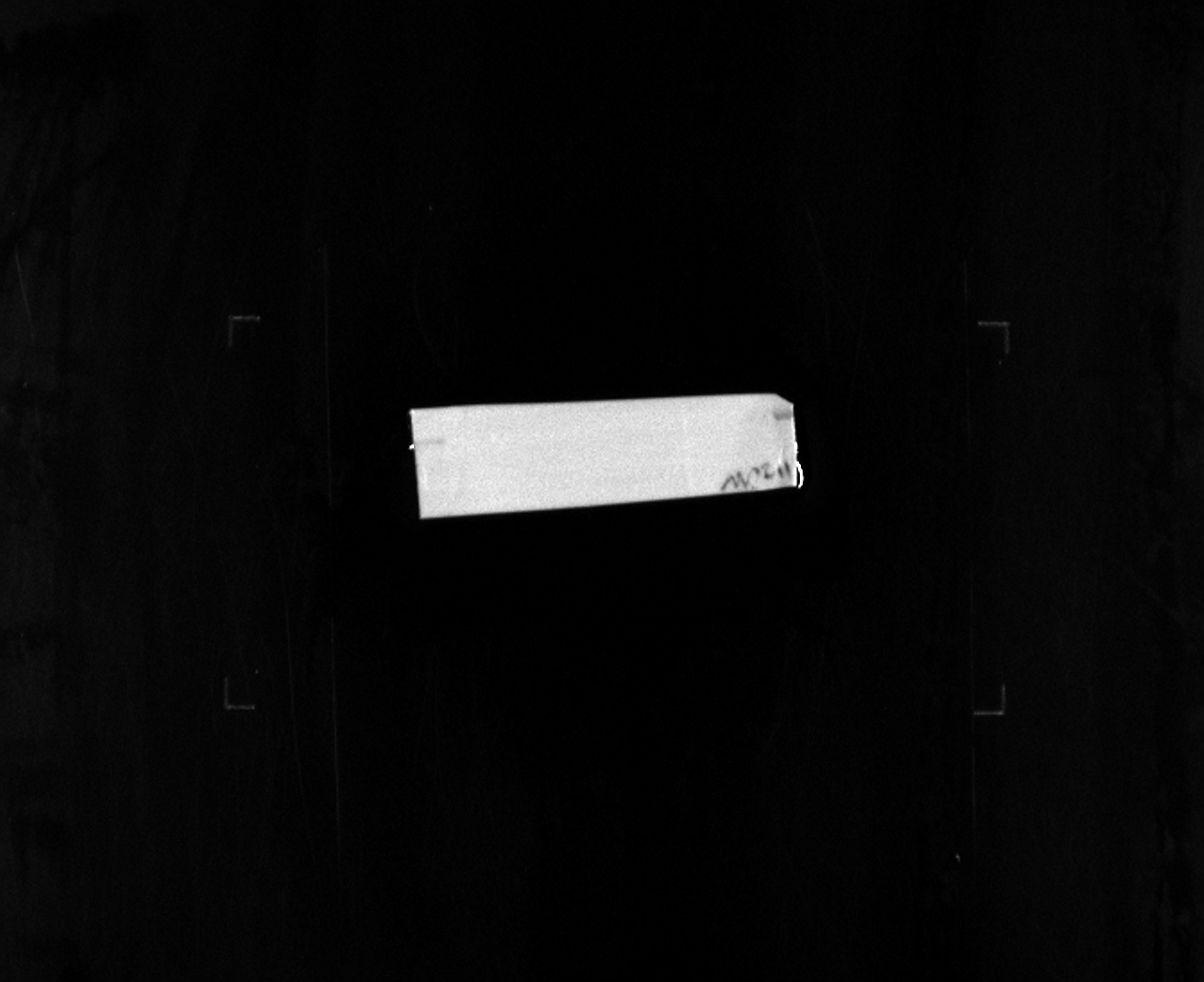

Supplement: Supplementary file 14 — Source Data EV Fig. 5 [file 44318_2023_3_MOESM14_ESM.zip › Figure EV5/5c/mre11 v TOP-IP-blm (nc siDC1) (input IP) w.Tif]

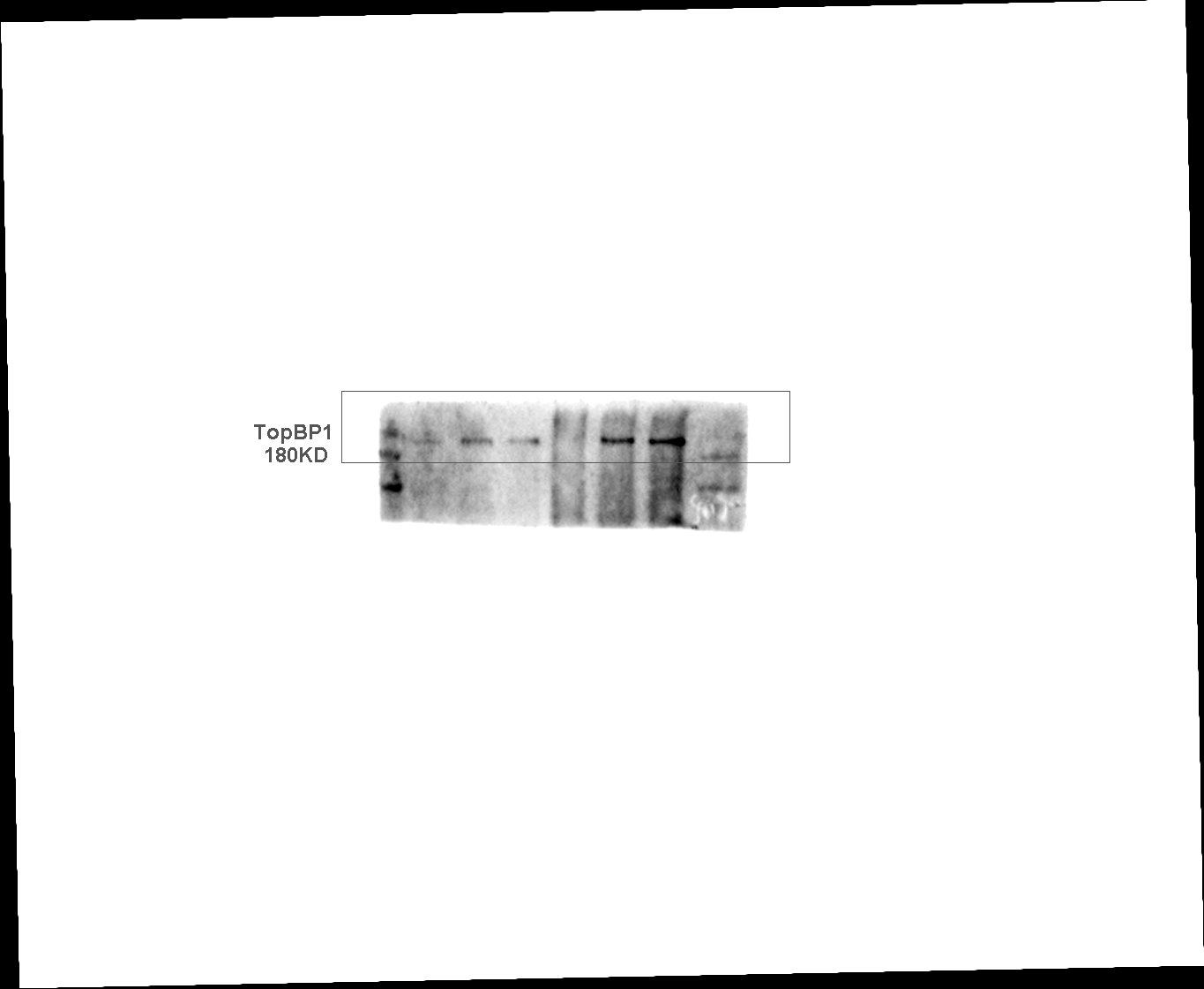

Supplement: Supplementary file 14 — Source Data EV Fig. 5 [file 44318_2023_3_MOESM14_ESM.zip › Figure EV5/5c/TOP v TOP-IP-blm (nc siDC1) (input IP) 3.jpg]

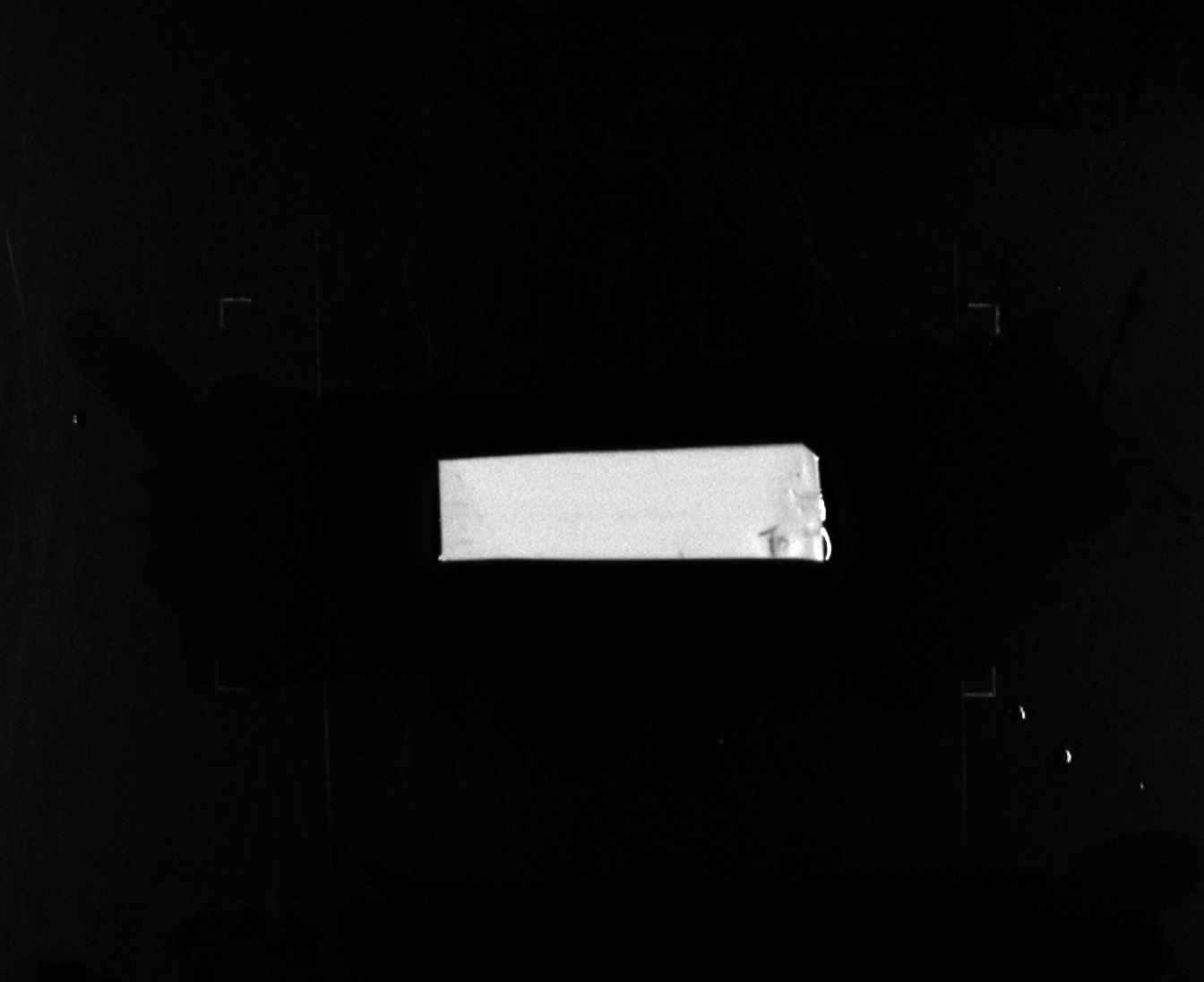

Supplement: Supplementary file 14 — Source Data EV Fig. 5 [file 44318_2023_3_MOESM14_ESM.zip › Figure EV5/5c/TOP v TOP-IP-blm (nc siDC1) (input IP) 4 W.Tif]

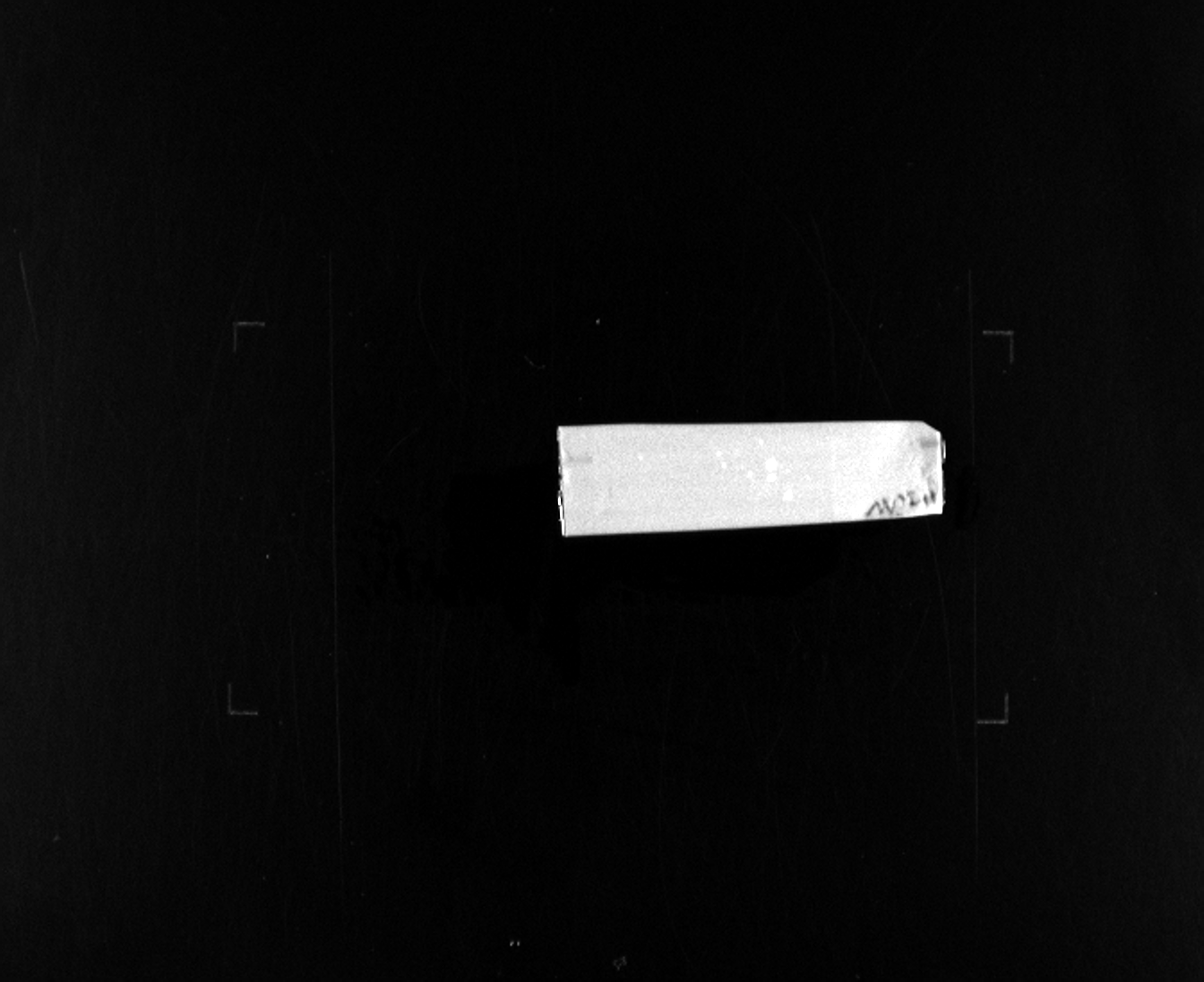

Supplement: Supplementary file 14 — Source Data EV Fig. 5 [file 44318_2023_3_MOESM14_ESM.zip › Figure EV5/5c/YTHDC1 v TOP-IP-blm (nc siDC1) (input IP) w.Tif]

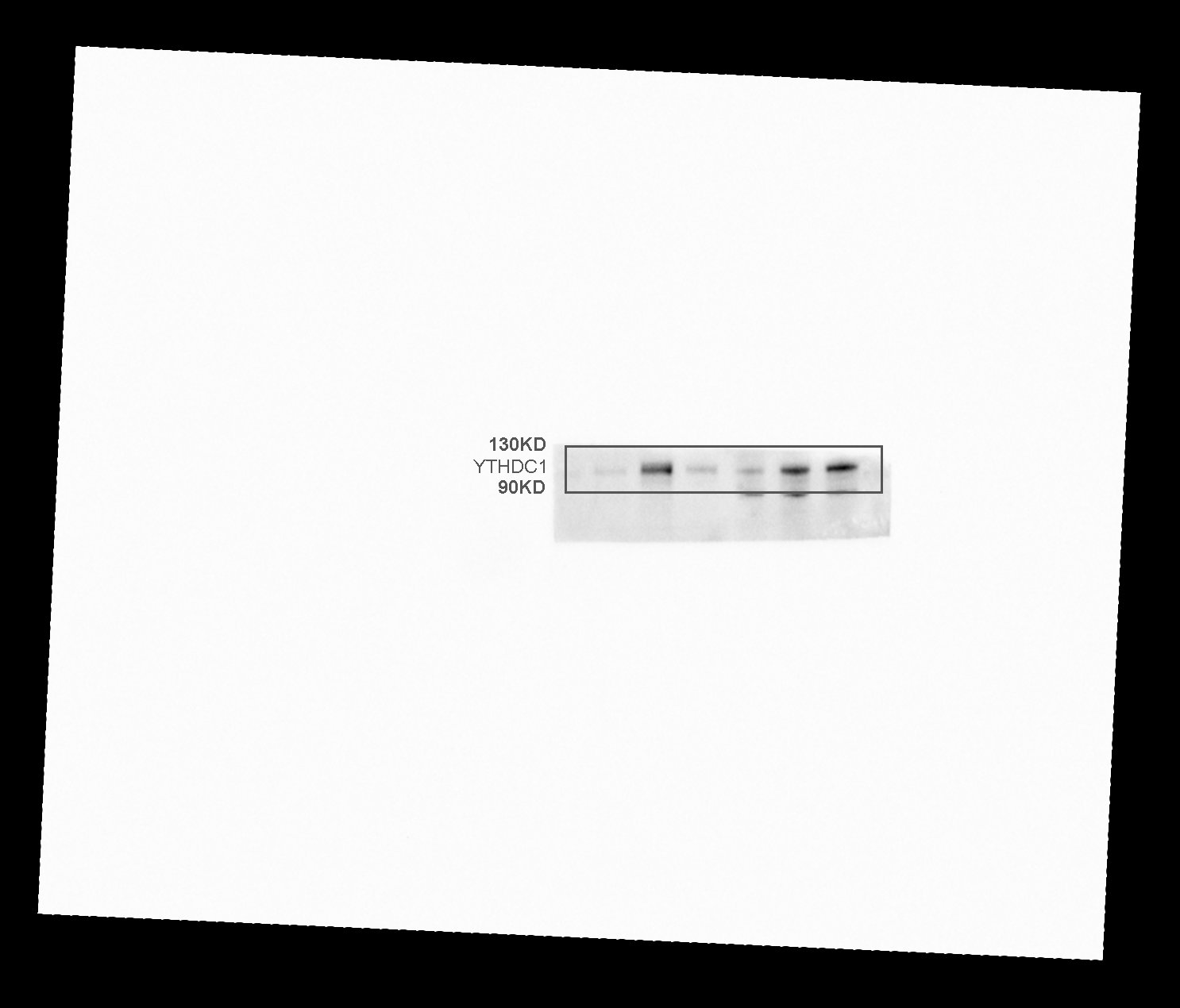

Supplement: Supplementary file 14 — Source Data EV Fig. 5 [file 44318_2023_3_MOESM14_ESM.zip › Figure EV5/5c/YTHDC1 v TOP-IP-blm (nc siDC1) (input IP).jpg]

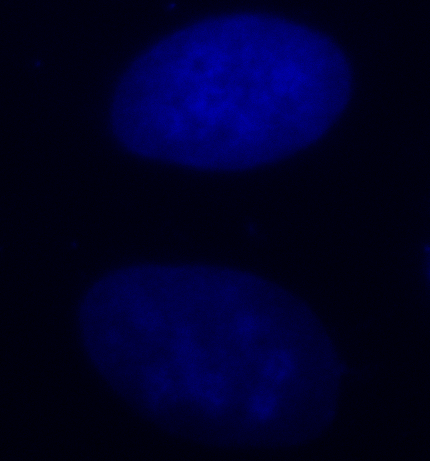

Supplement: Supplementary file 14 — Source Data EV Fig. 5 [file 44318_2023_3_MOESM14_ESM.zip › Figure EV5/5d-f/NC Topbp1 and yh2ax IF/Image0006_DAPI.TIF]

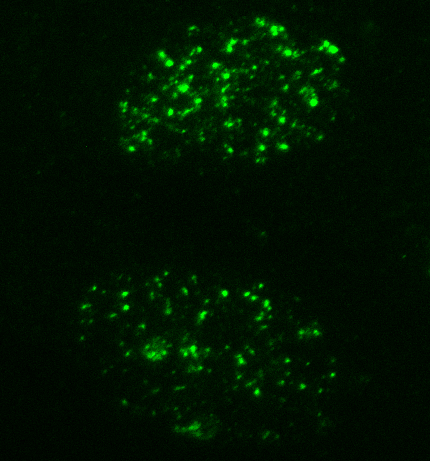

Supplement: Supplementary file 14 — Source Data EV Fig. 5 [file 44318_2023_3_MOESM14_ESM.zip › Figure EV5/5d-f/NC Topbp1 and yh2ax IF/Image0006_FITC.TIF]

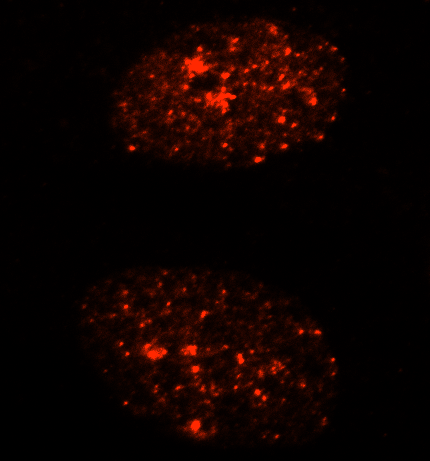

Supplement: Supplementary file 14 — Source Data EV Fig. 5 [file 44318_2023_3_MOESM14_ESM.zip › Figure EV5/5d-f/NC Topbp1 and yh2ax IF/Image0006_Texasred.TIF]

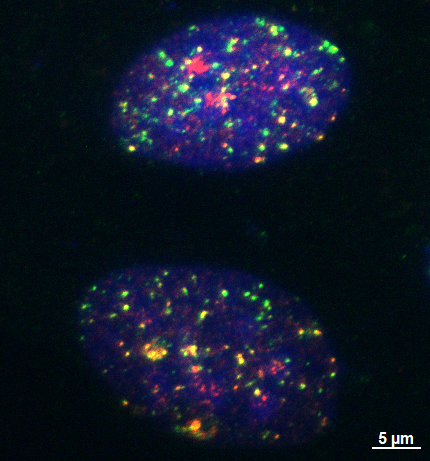

Supplement: Supplementary file 14 — Source Data EV Fig. 5 [file 44318_2023_3_MOESM14_ESM.zip › Figure EV5/5d-f/NC Topbp1 and yh2ax IF/MERGE.TIF]

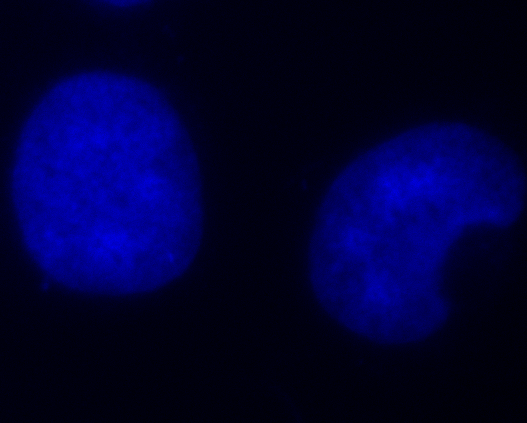

Supplement: Supplementary file 14 — Source Data EV Fig. 5 [file 44318_2023_3_MOESM14_ESM.zip › Figure EV5/5d-f/shYTHDC Topbp1 and yh2ax IF/Image0007_DAPI.TIF]

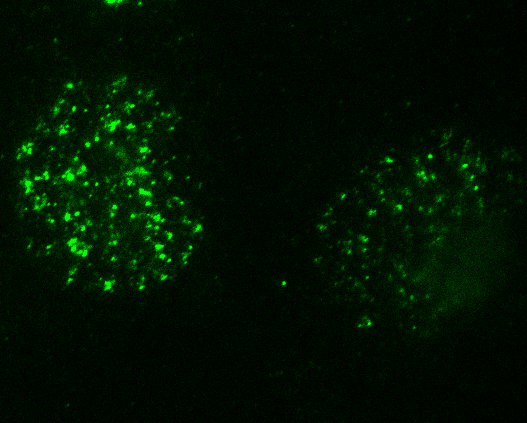

Supplement: Supplementary file 14 — Source Data EV Fig. 5 [file 44318_2023_3_MOESM14_ESM.zip › Figure EV5/5d-f/shYTHDC Topbp1 and yh2ax IF/Image0007_FITC.TIF]

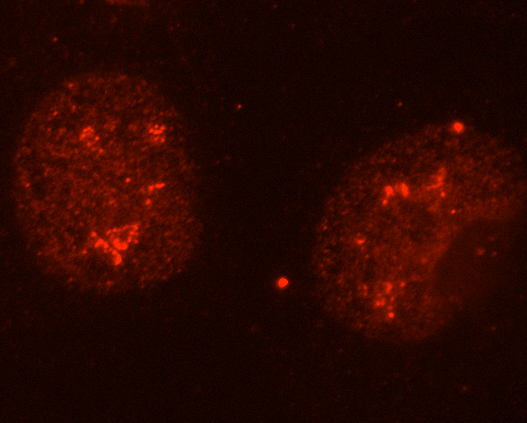

Supplement: Supplementary file 14 — Source Data EV Fig. 5 [file 44318_2023_3_MOESM14_ESM.zip › Figure EV5/5d-f/shYTHDC Topbp1 and yh2ax IF/Image0007_Texasred.TIF]

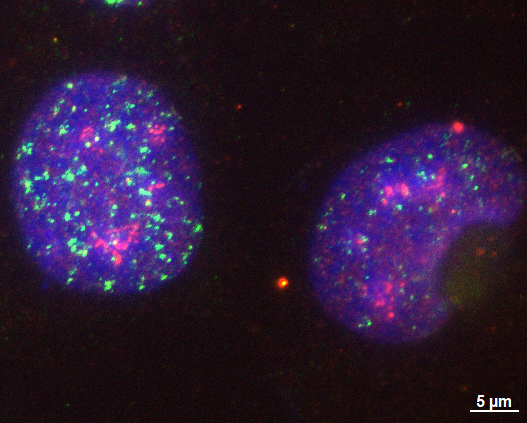

Supplement: Supplementary file 14 — Source Data EV Fig. 5 [file 44318_2023_3_MOESM14_ESM.zip › Figure EV5/5d-f/shYTHDC Topbp1 and yh2ax IF/merge.TIF]

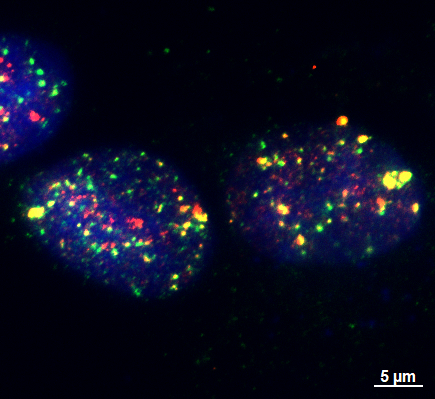

Supplement: Supplementary file 14 — Source Data EV Fig. 5 [file 44318_2023_3_MOESM14_ESM.zip › Figure EV5/5d-f/shYTHDC1+YTHDC1-mut Topbp1 and yh2ax IF/Image0009_(DAPI+FITC+Texasred).TIF]

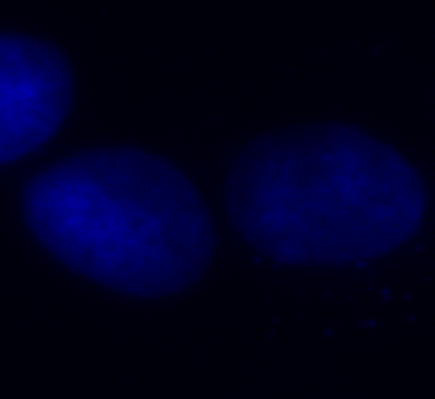

Supplement: Supplementary file 14 — Source Data EV Fig. 5 [file 44318_2023_3_MOESM14_ESM.zip › Figure EV5/5d-f/shYTHDC1+YTHDC1-mut Topbp1 and yh2ax IF/Image0009_DAPI.TIF]

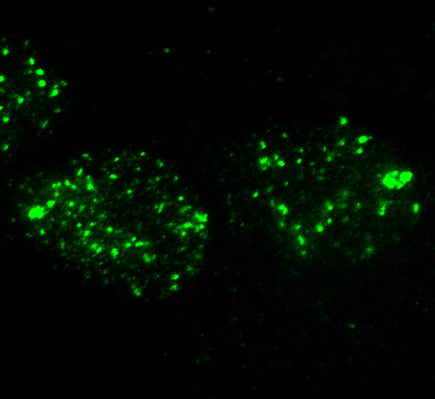

Supplement: Supplementary file 14 — Source Data EV Fig. 5 [file 44318_2023_3_MOESM14_ESM.zip › Figure EV5/5d-f/shYTHDC1+YTHDC1-mut Topbp1 and yh2ax IF/Image0009_FITC.TIF]

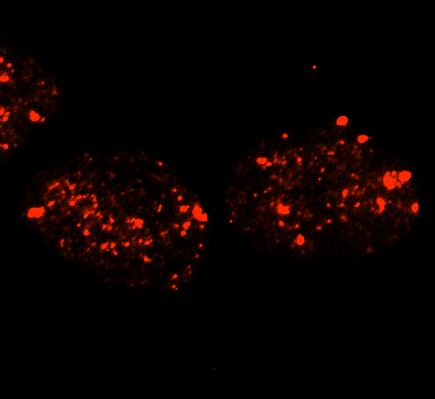

Supplement: Supplementary file 14 — Source Data EV Fig. 5 [file 44318_2023_3_MOESM14_ESM.zip › Figure EV5/5d-f/shYTHDC1+YTHDC1-mut Topbp1 and yh2ax IF/Image0009_Texasred.TIF]

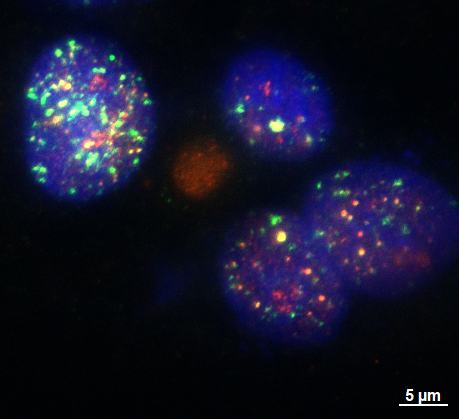

Supplement: Supplementary file 14 — Source Data EV Fig. 5 [file 44318_2023_3_MOESM14_ESM.zip › Figure EV5/5d-f/shYTHDC1+YTHDC1-WT Topbp1 and yh2ax IF/Image0008_(DAPI+FITC+Texasred).TIF]

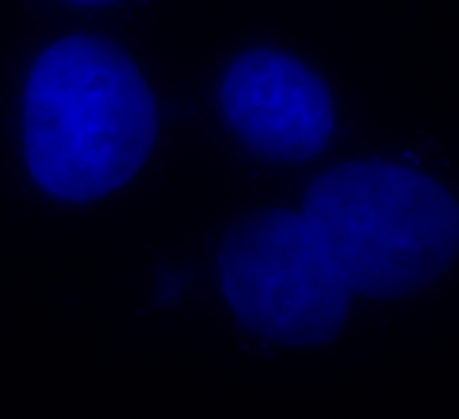

Supplement: Supplementary file 14 — Source Data EV Fig. 5 [file 44318_2023_3_MOESM14_ESM.zip › Figure EV5/5d-f/shYTHDC1+YTHDC1-WT Topbp1 and yh2ax IF/Image0008_DAPI.TIF]

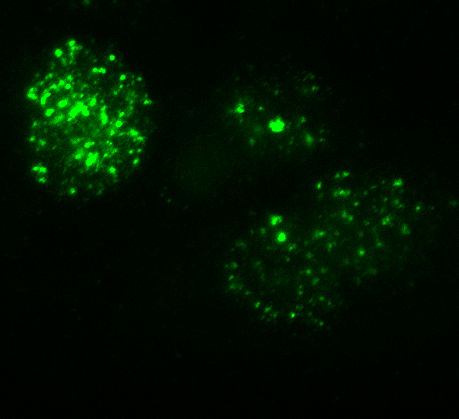

Supplement: Supplementary file 14 — Source Data EV Fig. 5 [file 44318_2023_3_MOESM14_ESM.zip › Figure EV5/5d-f/shYTHDC1+YTHDC1-WT Topbp1 and yh2ax IF/Image0008_FITC.TIF]

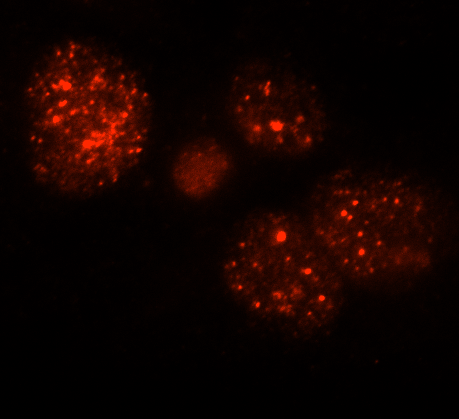

Supplement: Supplementary file 14 — Source Data EV Fig. 5 [file 44318_2023_3_MOESM14_ESM.zip › Figure EV5/5d-f/shYTHDC1+YTHDC1-WT Topbp1 and yh2ax IF/Image0008_Texasred.TIF]

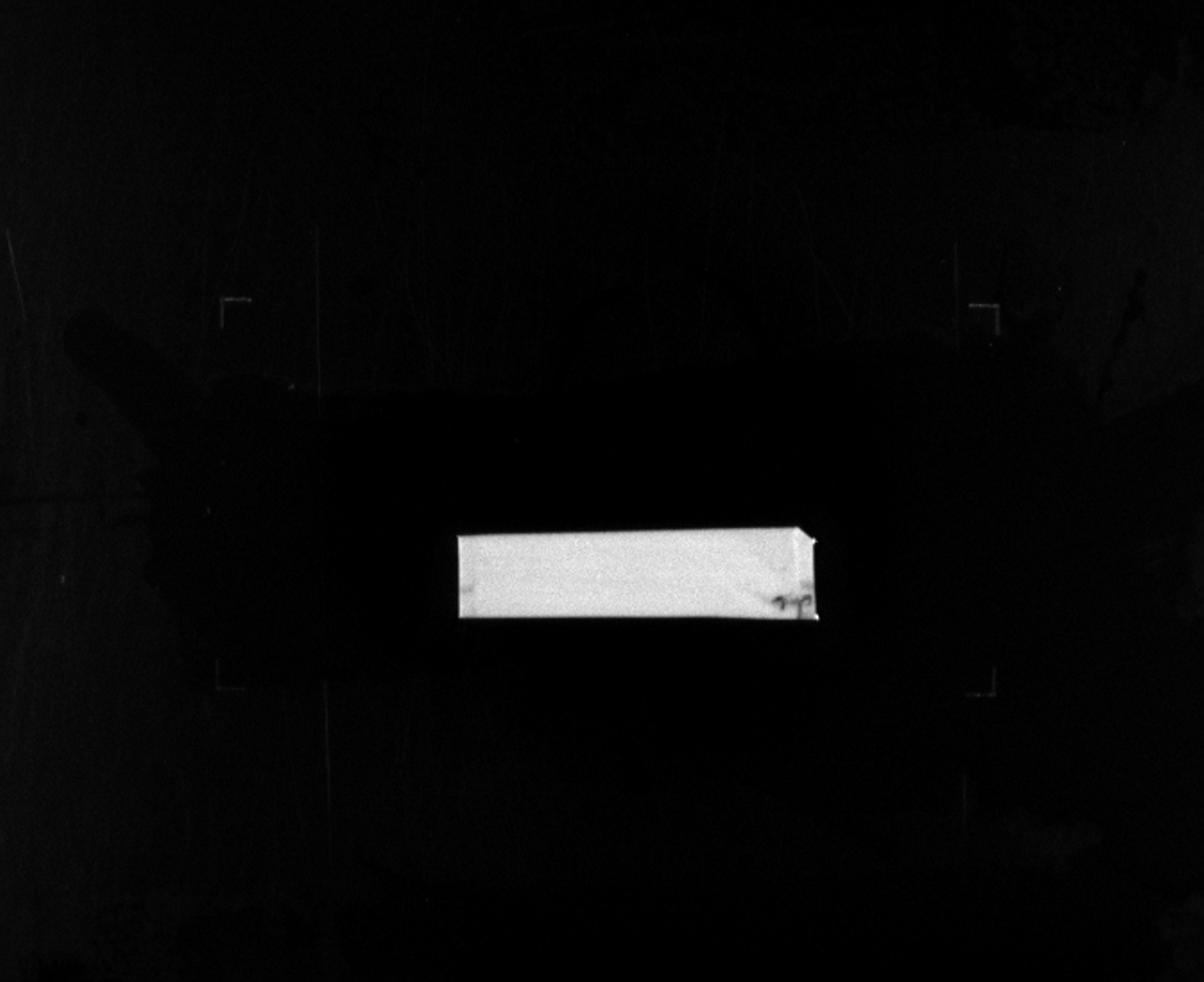

Supplement: Supplementary file 14 — Source Data EV Fig. 5 [file 44318_2023_3_MOESM14_ESM.zip › Figure EV5/5g/20230403 TOPBP1 v DC1-saline DC1-BLM (input IP) 2W.Tif]

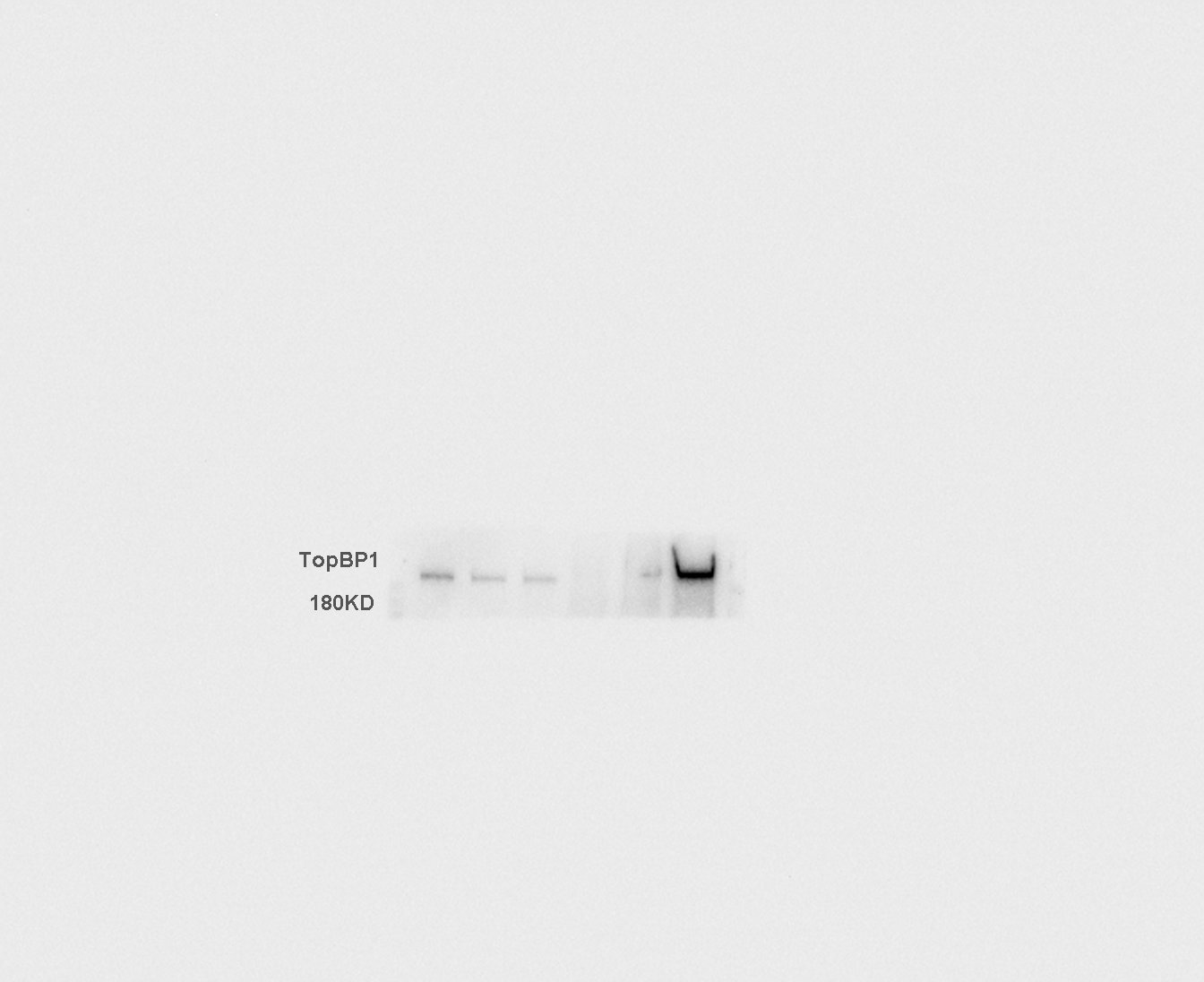

Supplement: Supplementary file 14 — Source Data EV Fig. 5 [file 44318_2023_3_MOESM14_ESM.zip › Figure EV5/5g/20230403 TOPBP1 v DC1-saline DC1-BLM (input IP) 2.jpg]

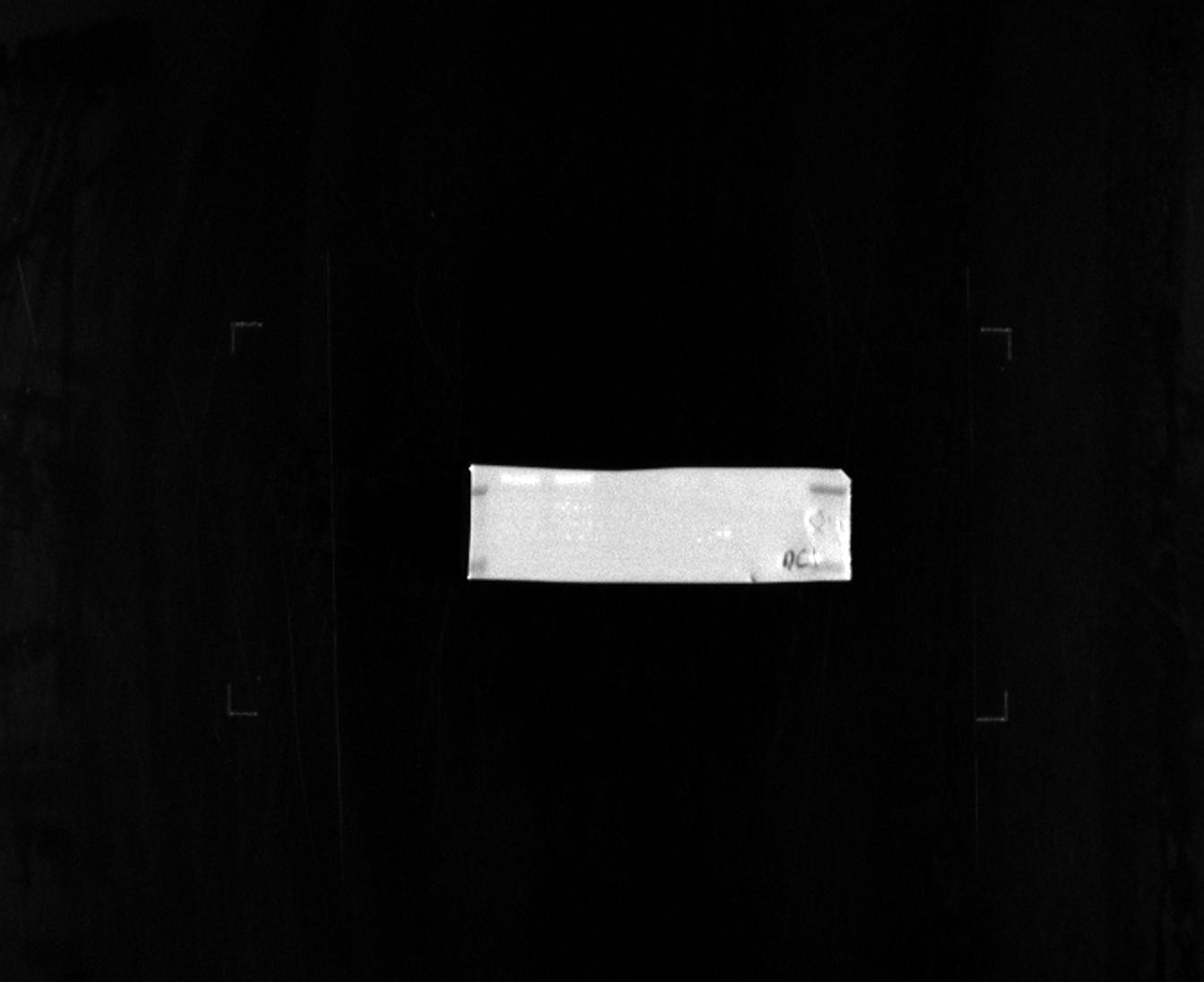

Supplement: Supplementary file 14 — Source Data EV Fig. 5 [file 44318_2023_3_MOESM14_ESM.zip › Figure EV5/5g/YTHDC1 v DC1-saline DC1-BLM (input IP) W.Tif]

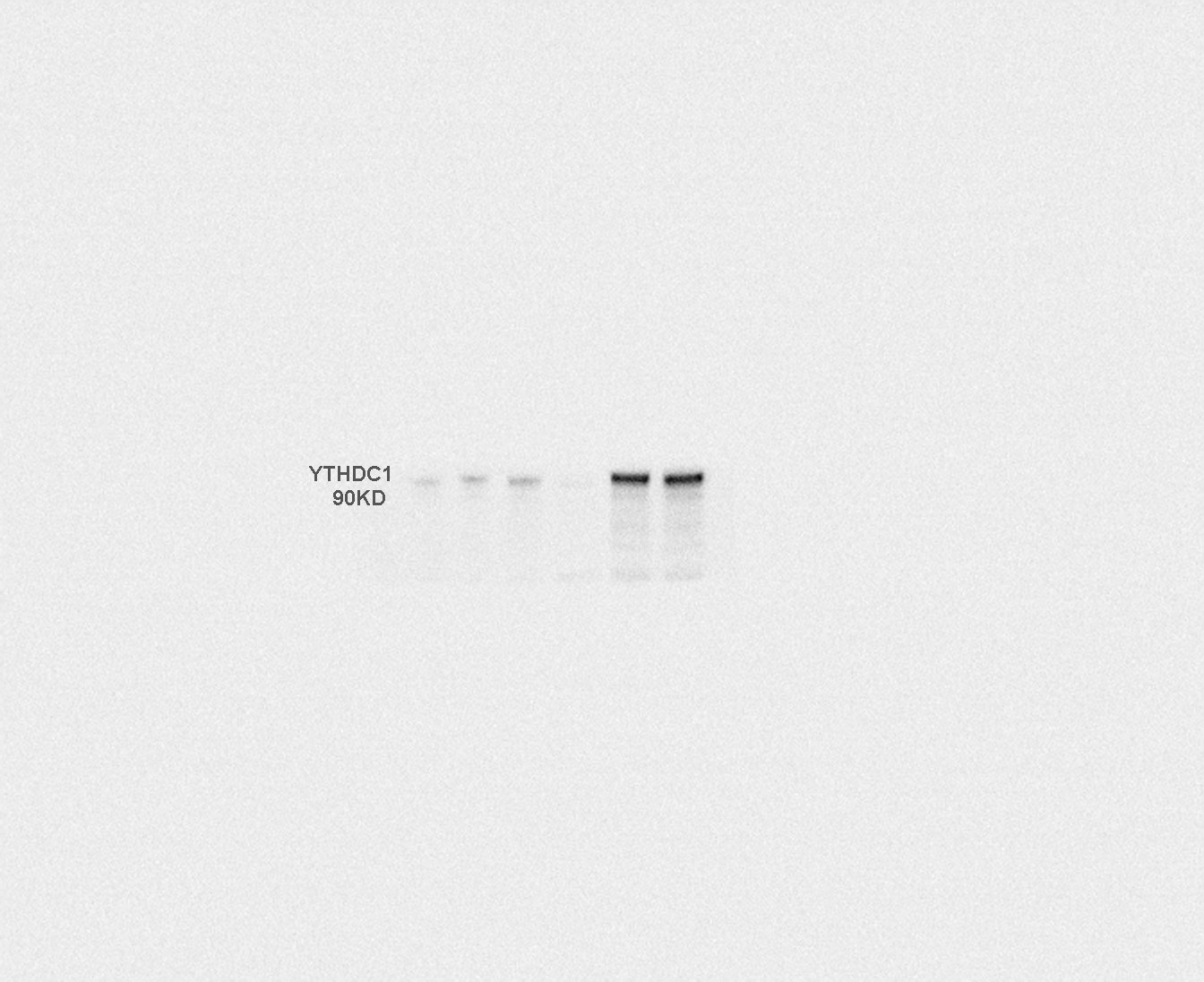

Supplement: Supplementary file 14 — Source Data EV Fig. 5 [file 44318_2023_3_MOESM14_ESM.zip › Figure EV5/5g/YTHDC1 v DC1-saline DC1-BLM (input IP).jpg]

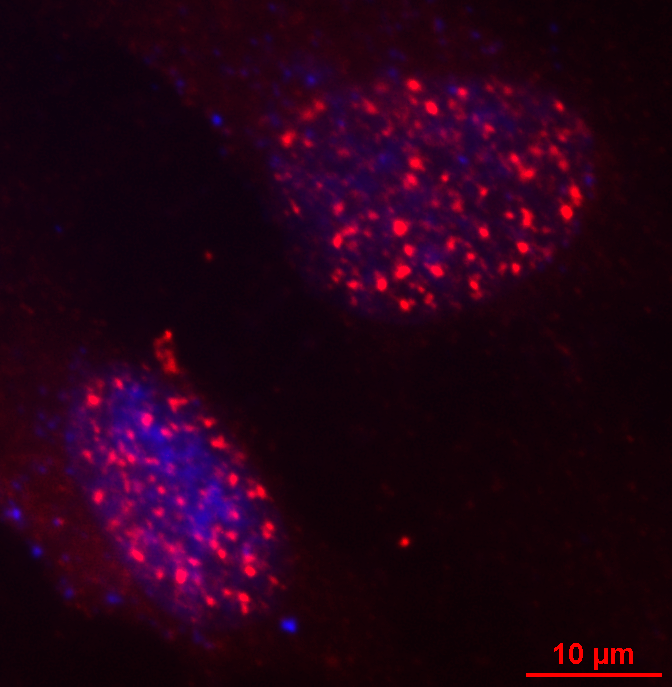

Supplement: Supplementary file 14 — Source Data EV Fig. 5 [file 44318_2023_3_MOESM14_ESM.zip › Figure EV5/5h-i/nc topnbp1-555 vp16/merge.tif]

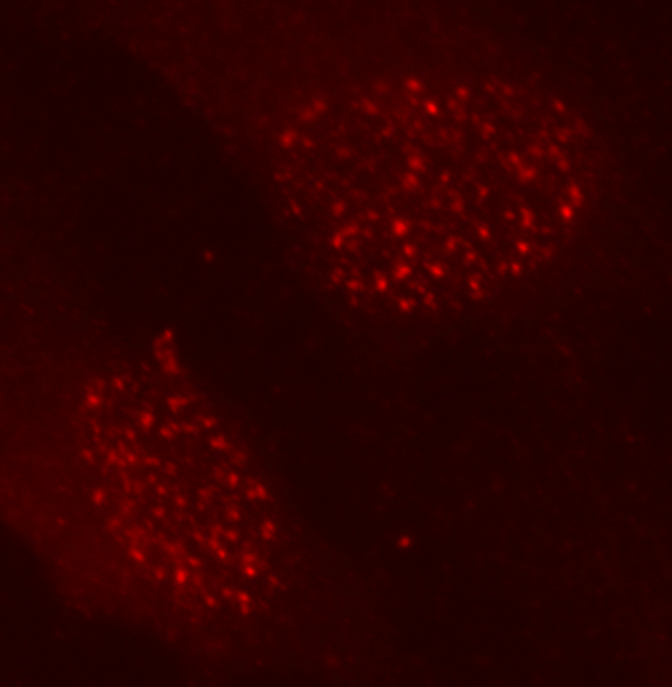

Supplement: Supplementary file 14 — Source Data EV Fig. 5 [file 44318_2023_3_MOESM14_ESM.zip › Figure EV5/5h-i/nc topnbp1-555 vp16/topbp1.tif]

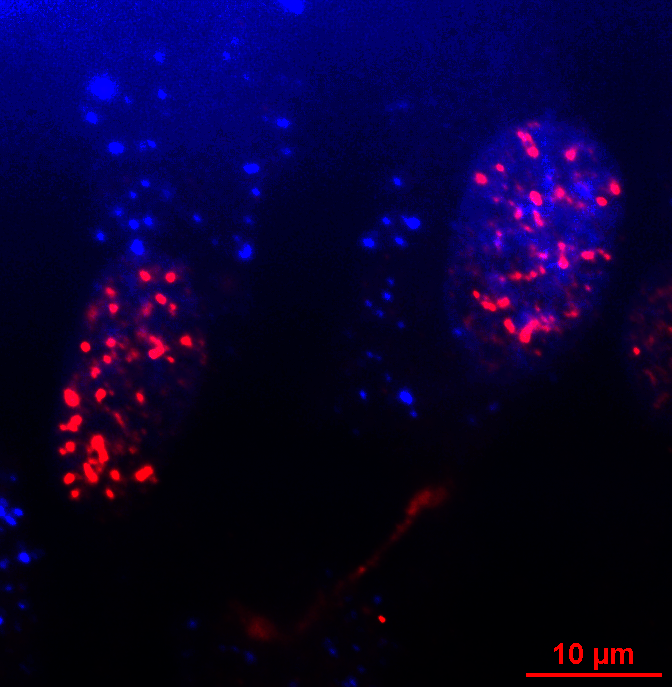

Supplement: Supplementary file 14 — Source Data EV Fig. 5 [file 44318_2023_3_MOESM14_ESM.zip › Figure EV5/5h-i/shYTHDC1 +NTD topBP1-555 vp16/merge.tif]

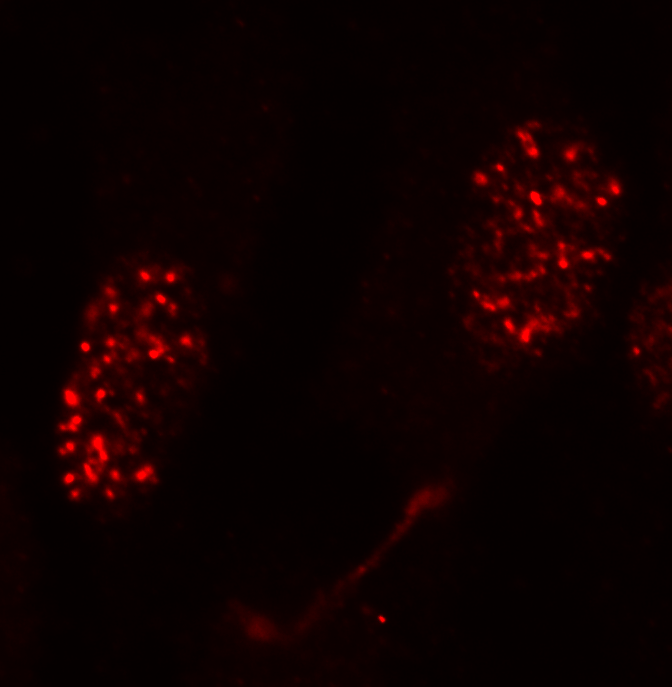

Supplement: Supplementary file 14 — Source Data EV Fig. 5 [file 44318_2023_3_MOESM14_ESM.zip › Figure EV5/5h-i/shYTHDC1 +NTD topBP1-555 vp16/topbp1.tif]

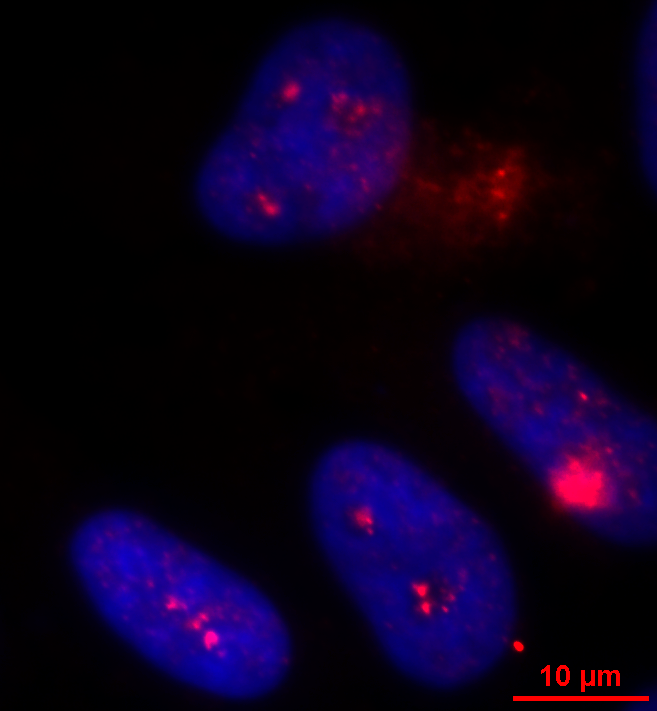

Supplement: Supplementary file 14 — Source Data EV Fig. 5 [file 44318_2023_3_MOESM14_ESM.zip › Figure EV5/5h-i/shYTHDC1 Topbp1-555/MERGE.tif]

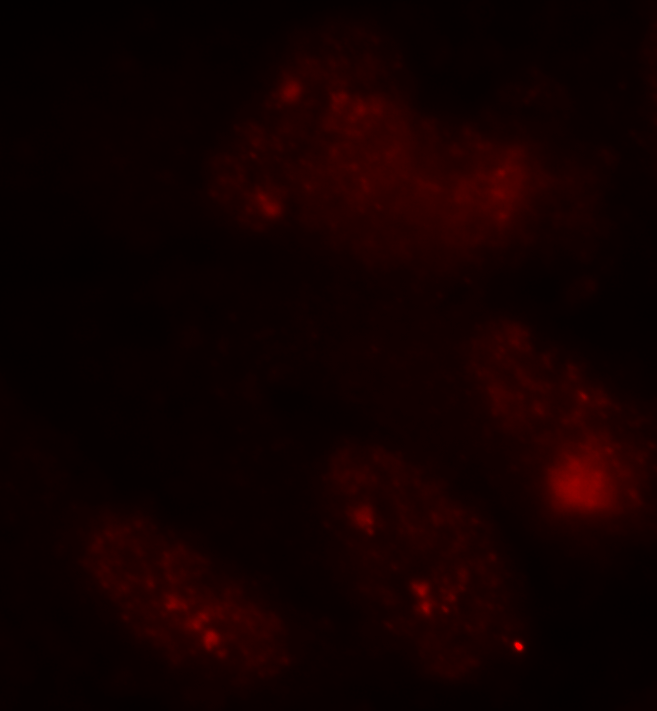

Supplement: Supplementary file 14 — Source Data EV Fig. 5 [file 44318_2023_3_MOESM14_ESM.zip › Figure EV5/5h-i/shYTHDC1 Topbp1-555/Topbp1.tif]

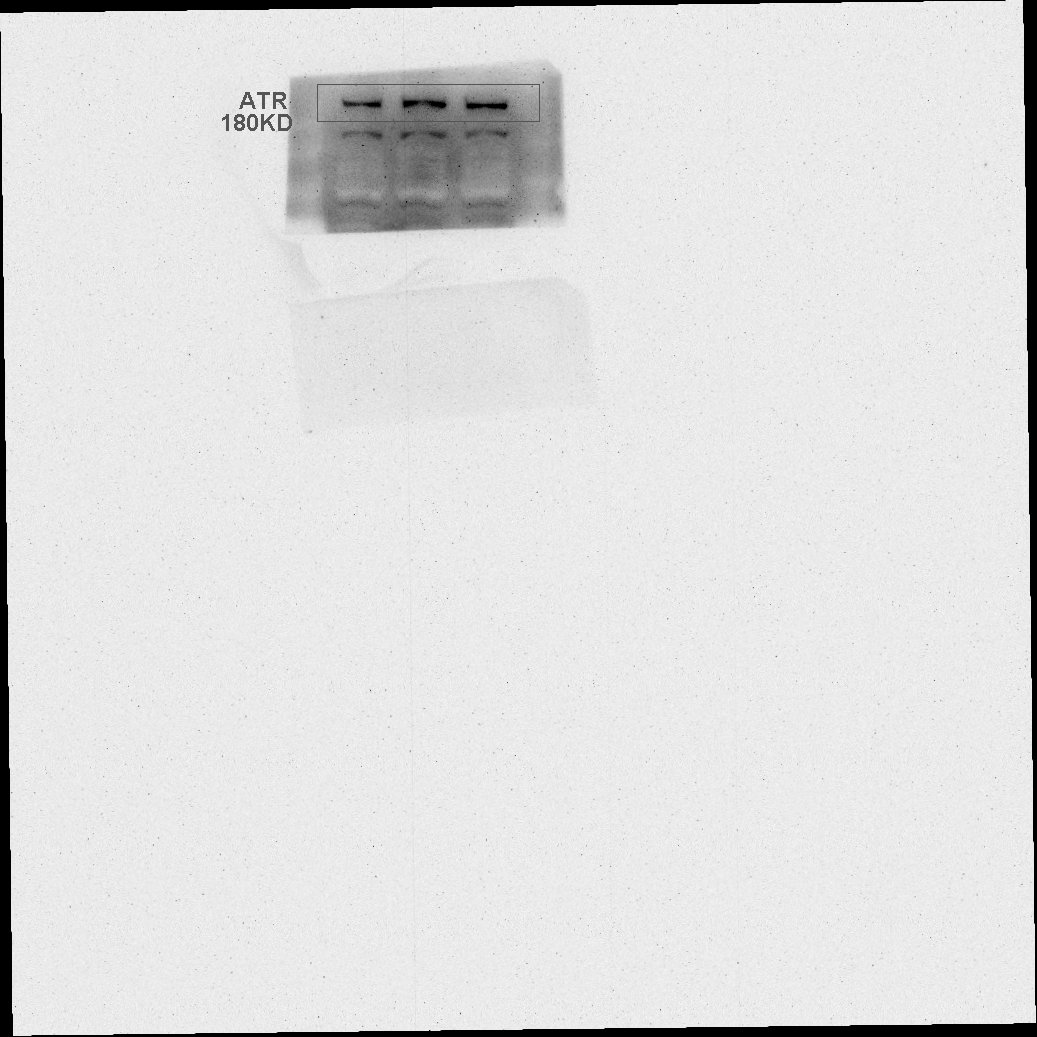

Supplement: Supplementary file 14 — Source Data EV Fig. 5 [file 44318_2023_3_MOESM14_ESM.zip › Figure EV5/5j-l/ATR NC siDC1 siDC1+N .jpg]

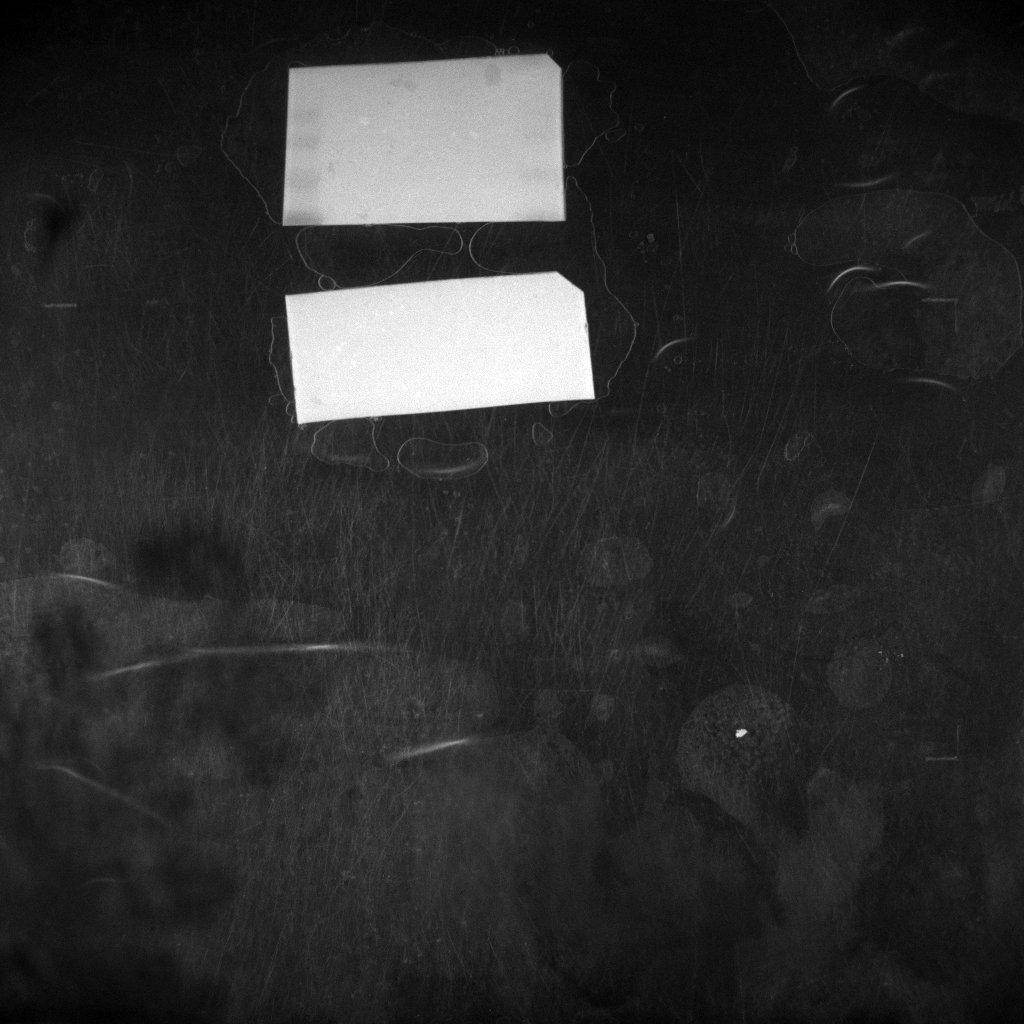

Supplement: Supplementary file 14 — Source Data EV Fig. 5 [file 44318_2023_3_MOESM14_ESM.zip › Figure EV5/5j-l/ATR NC siDC1 siDC1+N w .tif]

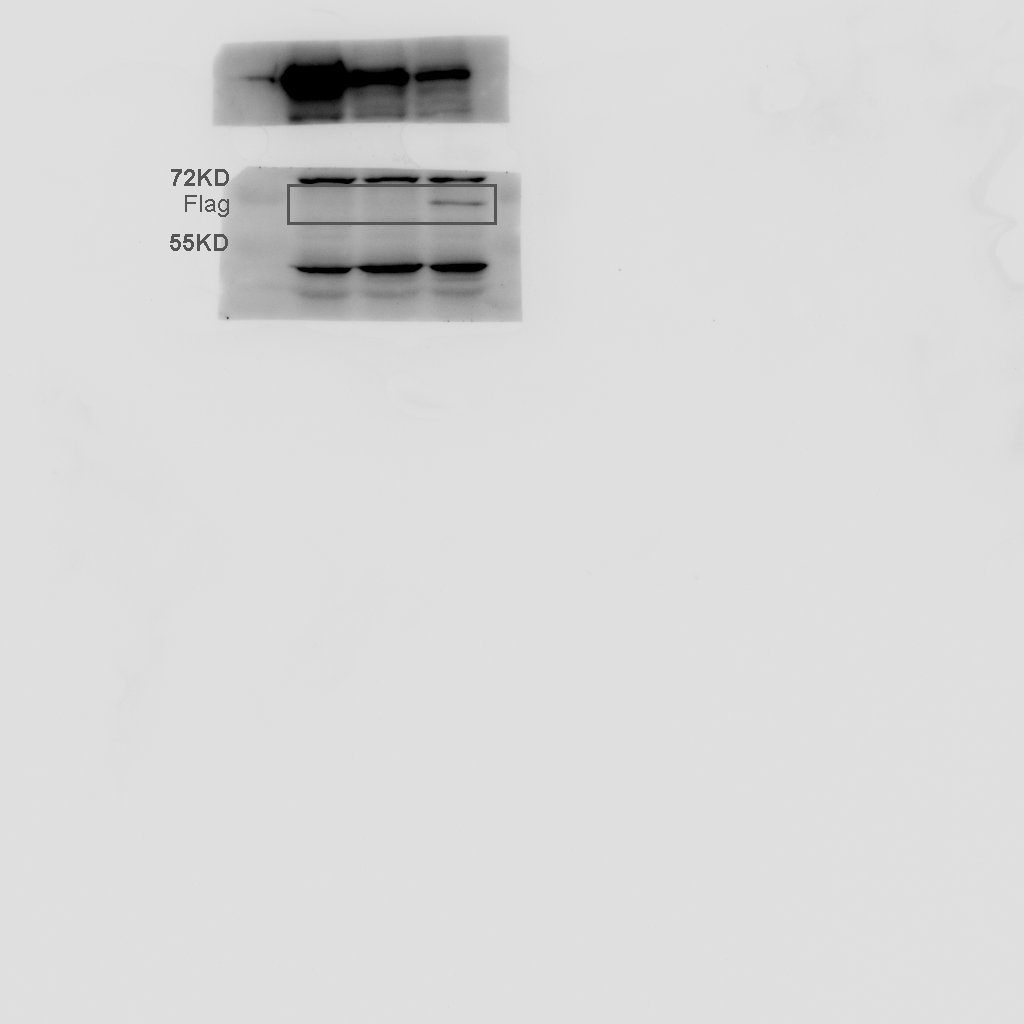

Supplement: Supplementary file 14 — Source Data EV Fig. 5 [file 44318_2023_3_MOESM14_ESM.zip › Figure EV5/5j-l/flag.jpg]

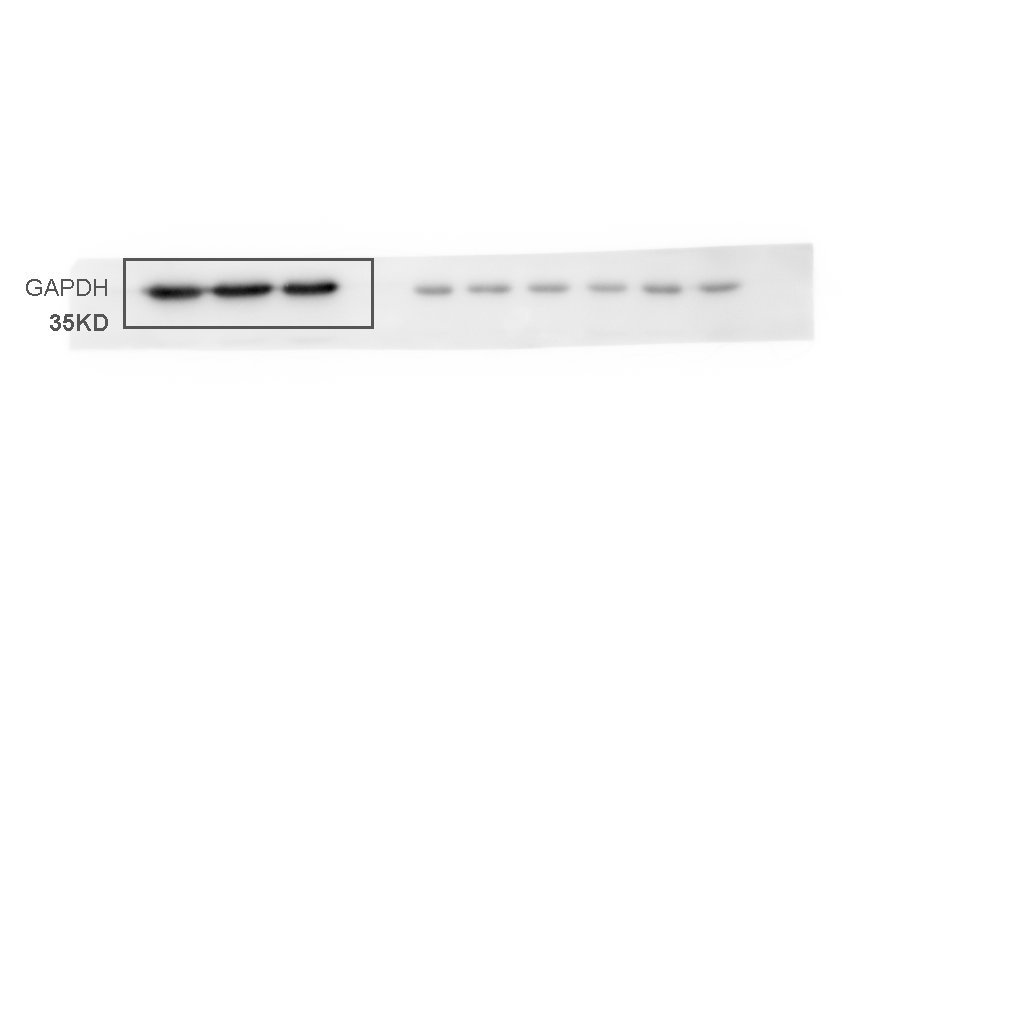

Supplement: Supplementary file 14 — Source Data EV Fig. 5 [file 44318_2023_3_MOESM14_ESM.zip › Figure EV5/5j-l/GAPDH NC siDC1 siDC1+N .jpg]

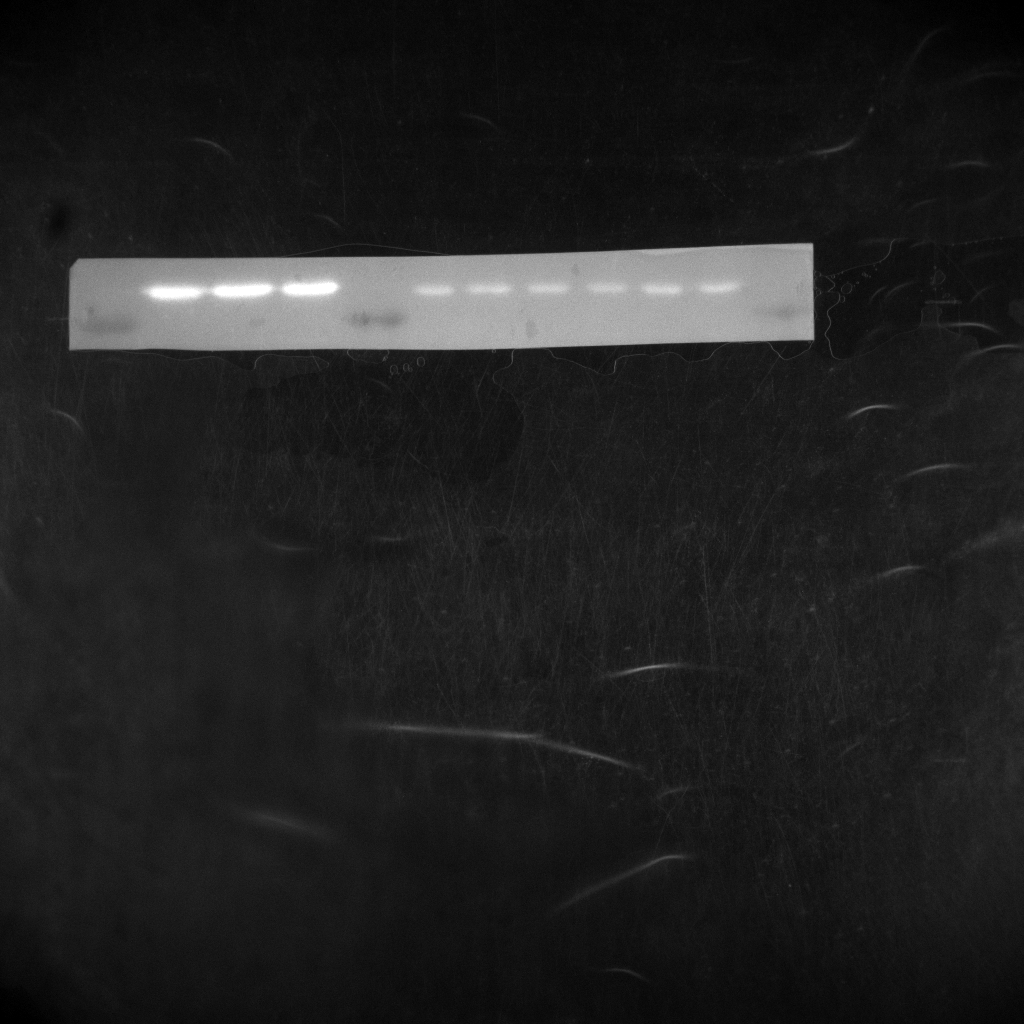

Supplement: Supplementary file 14 — Source Data EV Fig. 5 [file 44318_2023_3_MOESM14_ESM.zip › Figure EV5/5j-l/GAPDH NC siDC1 siDC1+N W.tif]

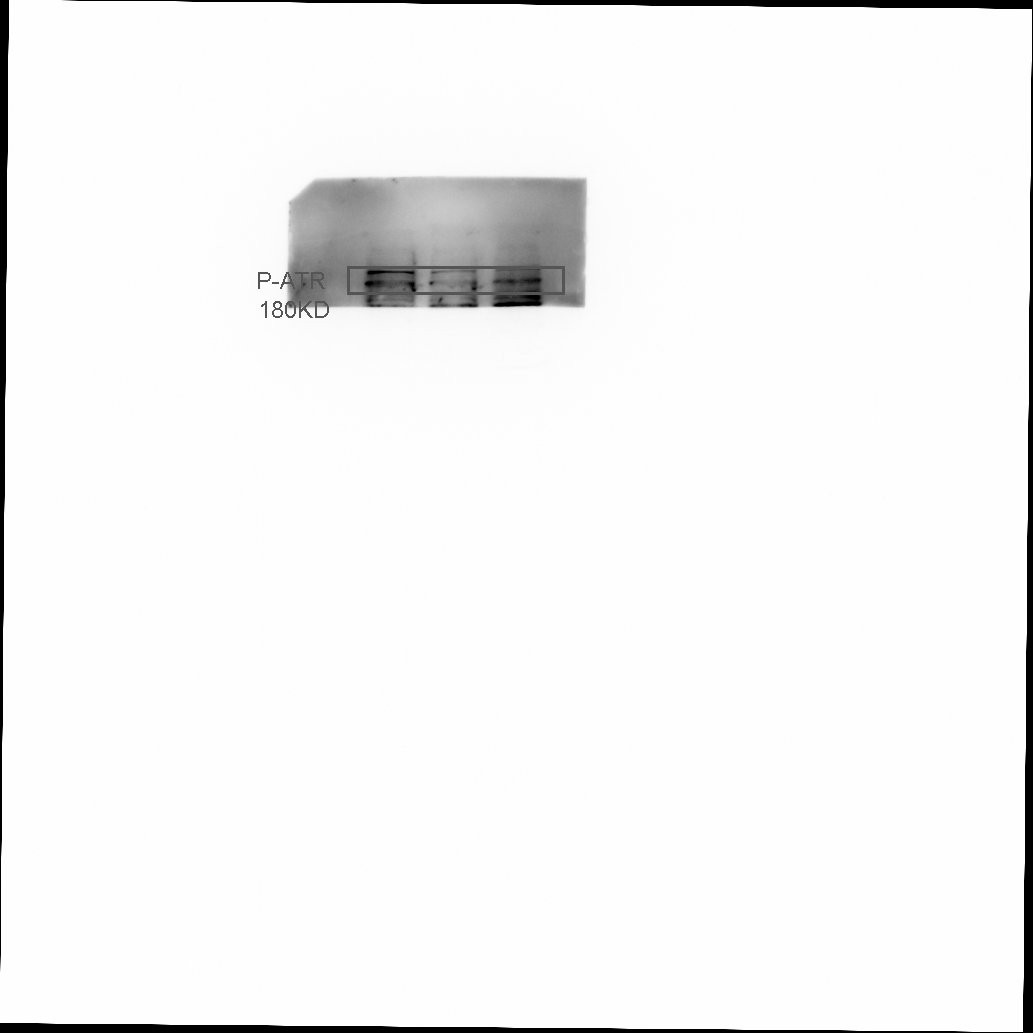

Supplement: Supplementary file 14 — Source Data EV Fig. 5 [file 44318_2023_3_MOESM14_ESM.zip › Figure EV5/5j-l/P-ATR NC siDC1 siDC1+N -1 .jpg]

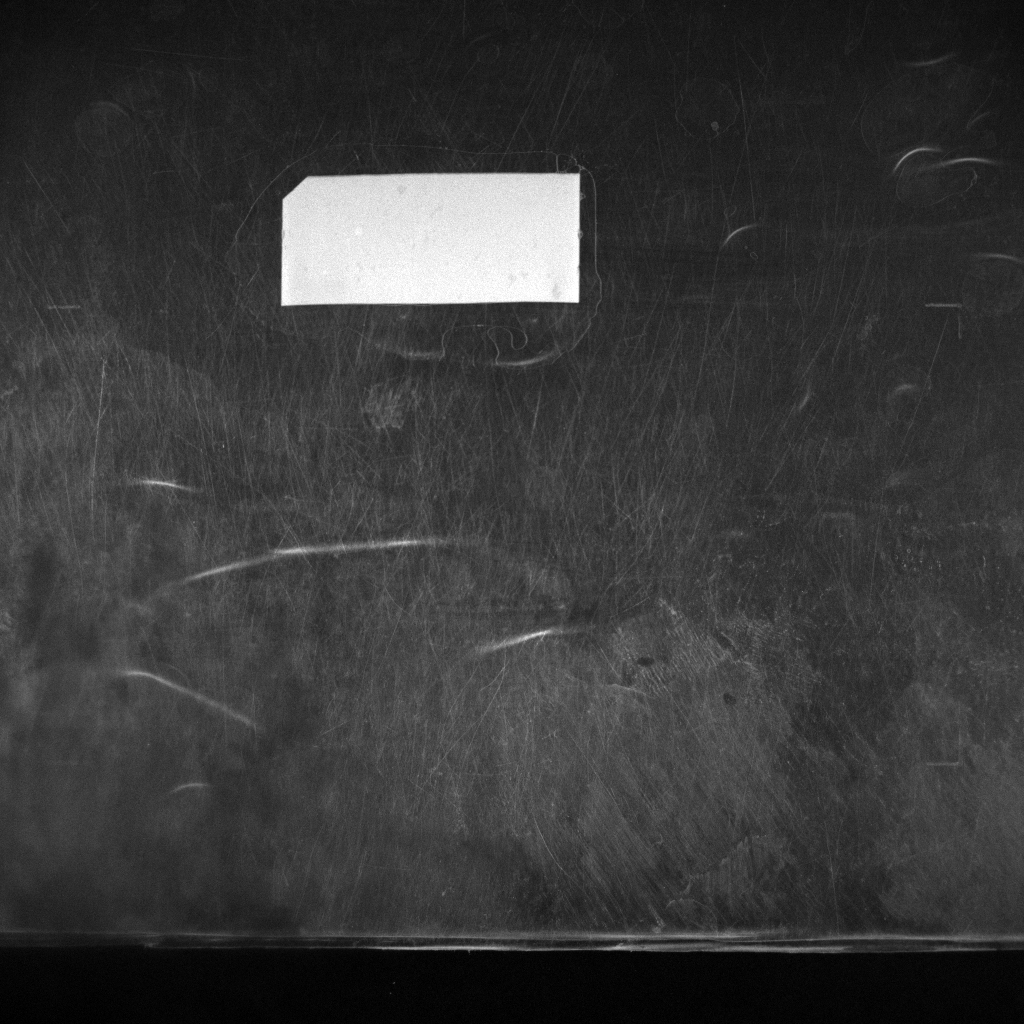

Supplement: Supplementary file 14 — Source Data EV Fig. 5 [file 44318_2023_3_MOESM14_ESM.zip › Figure EV5/5j-l/P-ATR NC siDC1 siDC1+N -1 w .tif]

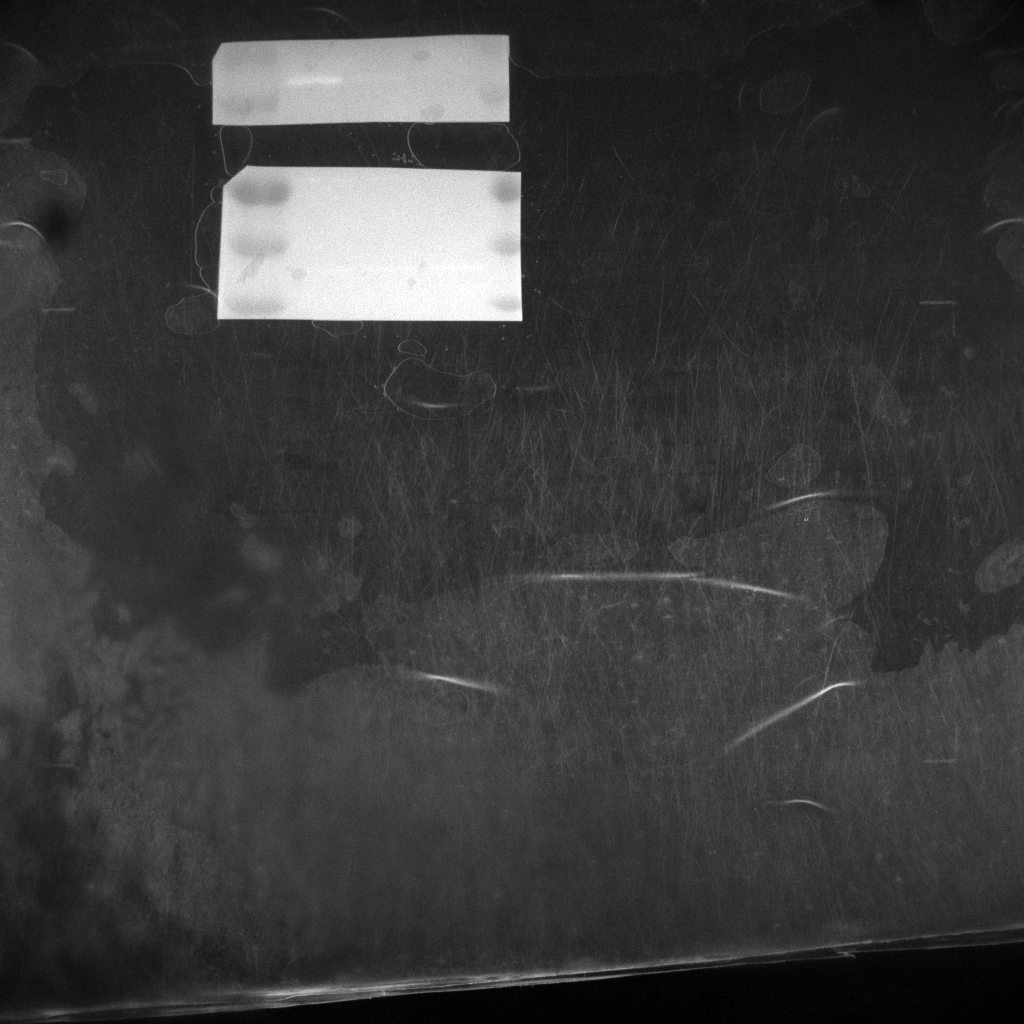

Supplement: Supplementary file 14 — Source Data EV Fig. 5 [file 44318_2023_3_MOESM14_ESM.zip › Figure EV5/5j-l/YTHDC1 w.tif]

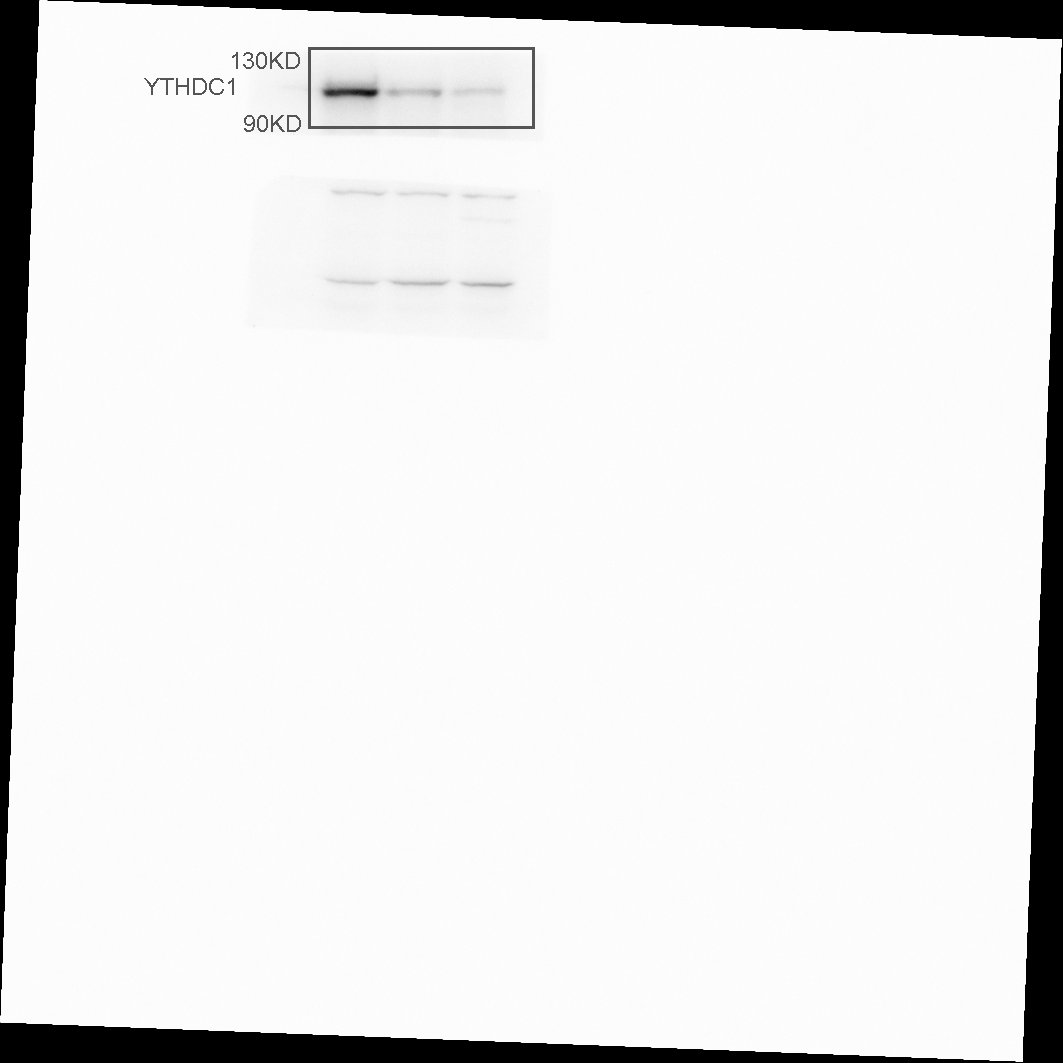

Supplement: Supplementary file 14 — Source Data EV Fig. 5 [file 44318_2023_3_MOESM14_ESM.zip › Figure EV5/5j-l/YTHDC1.jpg]
