# Supplementary material for: YTHDC1 delays cellular senescence and pulmonary fibrosis by activating ATR in an m6A-independent manner
Source: EMBO J. 2023 Dec 15;43(1):4. doi: 10.1038/s44318-023-00003-2 (PMC10883269; doi:10.1038/s44318-023-00003-2)
Supplement: Supplementary file 15 — Expanded View Figures [file 44318_2023_3_MOESM15_ESM.pdf]

Expanded View Figures

Figure EV1. Knockdown of YTHDC1 accelerates stress-induced pulmonary epithelial cell senescence.

(**A**) METTL3, METTL14, WTAP, FTO and ALKBH5 expression levels in normal (35 people) and IPF (49 people) human lung tissues were determined using published datasets (Data ref: Ahangari et al, 2019). (**B**) YTHDF1, YTHDF2 and YTHDC2 expression levels in normal (35 people) and IPF (49 people) human lung tissues were determined using published datasets (Data ref: Ahangari et al, 2019). (**C**) Immunofluorescence was performed to determine the localization of WTAP/FTO (red) and SPC (green) in mice lungs that treated with saline or BLM for 7 days. Scale bar: 100 μm. (**D**) Quantification of panel **C**. The percentage of double-positive cells in WTAP/FTO-positive cells was calculated ($n = 5$ per group). (**E**) IF detection of Ki67 in control or YTHDC1-depleted L2 cells. Cells were treated with BLM for 4 days. Scale bar: 10 μm. (**F**) Quantification of **E**. The percentage of Ki67 positive cells was calculated ($n \geq 100$ cells × three repeats). (**G**) YTHDC1, p21, p16 and SASP factors were detected by RT-qPCR using L2 cells transfected with NC or siYTHDC1. Forty-eight hours after transfection, cells were treated with BLM or saline for 4 days. (**H**) SA-β-gal staining of L2 cells transfected with NC or siYTHDC1. Forty-eight hours after transfection, cells were treated with VP-16 for 1 days and released to 7 days and subjected to SA-β-gal staining. Scale bars: 100 μm. (**I**) Quantification of **H**. The percentage of SA-β-gal positive cells was calculated ($n \geq 100$ cells × three repeats). (**J**) Representative images of p21 in the mice lungs ($n \geq 4$ per group). C57/BL6 mice transfected with indicated AAV vectors were treated with BLM for 7 days ($n \geq 4$ per group). Inset shows positive signals at high magnification. from the mice lungs. Scale bar: 50μm. (**K**) Quantification of panel **J**. The percentage of p21 positive cells was calculated. (**L**, **M**), as in panels **J**,**K**, except using p16 antibody to perform the IHC. ($n \geq 4$ per group). Scale bar: 50 μm. (**N**,**O**) as in panels **J**,**K**, except using α-SMA antibody to perform the IHC. ($n \geq 4$ per group). Scale bar: 50 μm. (**P**) Immunofluorescence (IF) detection of γH2AX foci in the mice lung from panel **J**. ($n \geq 4$ per group). Scale bar: 5 μm. (**Q**) Quantification of panel **P**. The percentage of cells with γH2AX foci was calculated. (**R**) Quantification of panel **P**. The average number of γH2AX foci per cell. Data information: All values are mean ± SEM. The unpaired Student's two-tailed *t*-test was used to determine the statistical significance (*$P < 0.05$, **$P < 0.01$, ***$P < 0.001$, ****$P < 0.0001$). $n =$ number of biological replicates. Source data are available online for this figure.

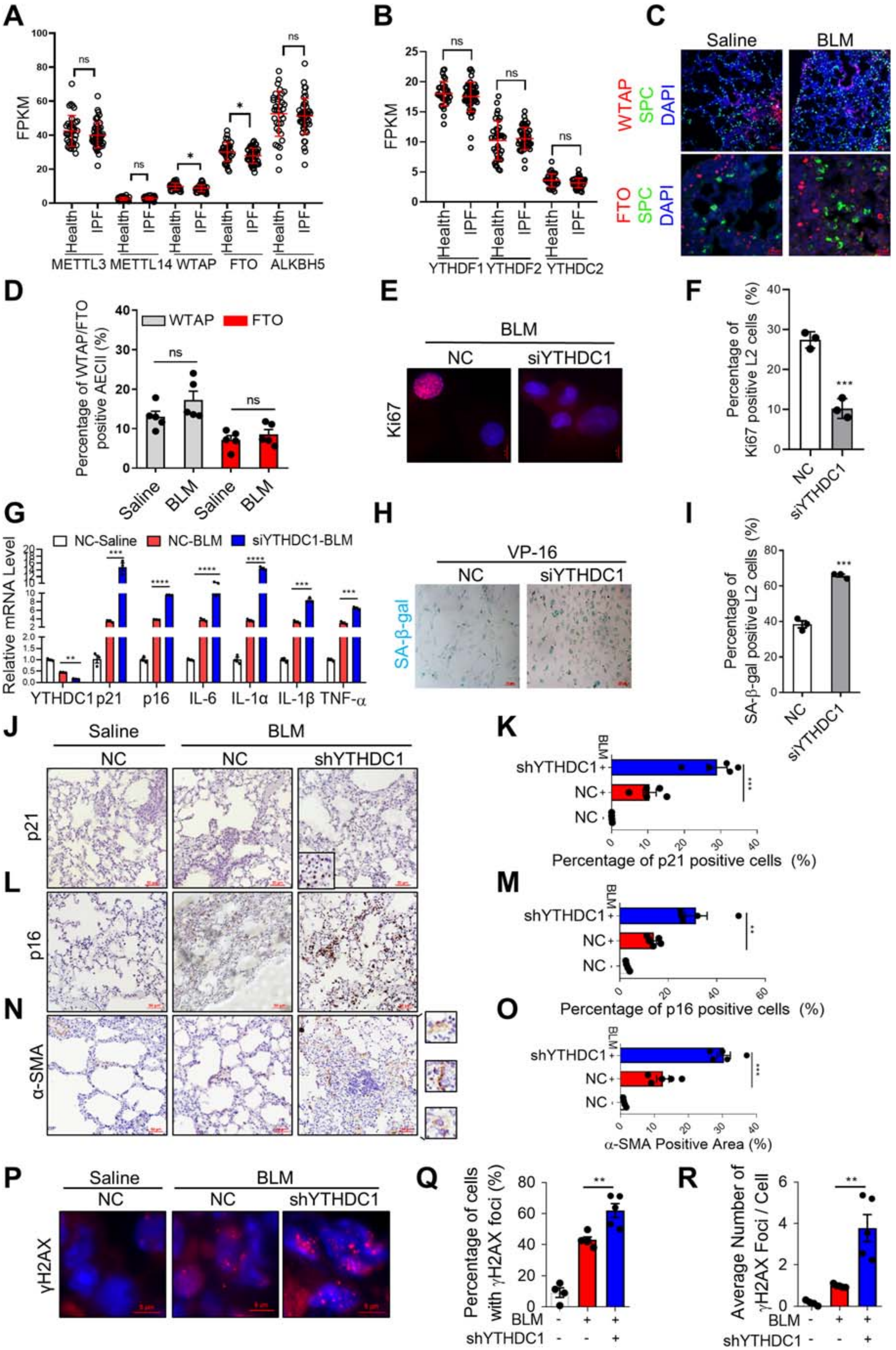
A
FPKM
METTL3 METTL14 WTAP FTO ALKBH5
Health IPF
ns
*
B
YTHDF1 YTHDF2 YTHDC2
C
Saline BLM
WTAP SPC DAPI
FTO SPC DAPI
D
Percentage of WTAP/FTO positive AECII (%)
WTAP FTO
Saline BLM Saline BLM
E
BLM
NC siYTHDC1
Ki67
F
Percentage of Ki67 positive L2 cells (%)
NC siYTHDC1
G
NC-Saline NC-BLM siYTHDC1-BLM
Relative mRNA Level
YTHDC1 p21 p16 IL-6 IL-1α IL-1β TNF-α
H
VP-16
NC siYTHDC1
SA-β-gal
I
Percentage of SA-β-gal positive L2 cells (%)
NC siYTHDC1
J
Saline BLM
NC NC shYTHDC1
p21
K
Percentage of p21 positive cells (%)
L
p16
M
Percentage of p16 positive cells (%)
N
α-SMA
O
α-SMA Positive Area (%)
P
Saline BLM
NC NC shYTHDC1
γH2AX
Q
Percentage of cells with γH2AX foci (%)
BLM
shYTHDC1
R
Average Number of γH2AX Foci / Cell

 © The Author(s)

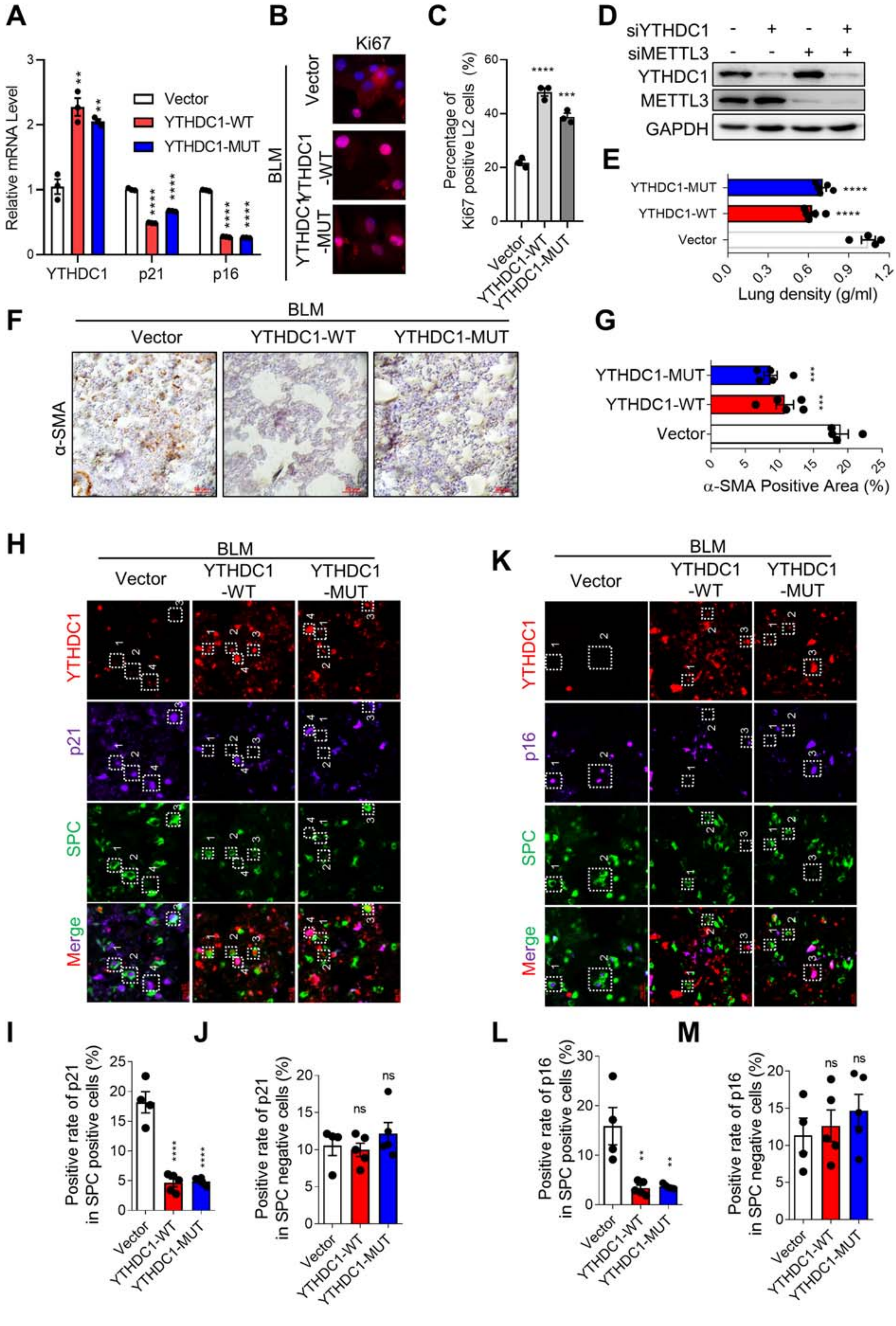
A
Relative mRNA Level
Vector
YTHDC1-WT
YTHDC1-MUT
YTHDC1
p21
p16
B
Ki67
BLM
Vector
YTHDC1 -WT
YTHDC1 -MUT
C
Percentage of Ki67 positive L2 cells (%)
Vector
YTHDC1-WT
YTHDC1-MUT
D
siYTHDC1
siMETTL3
YTHDC1
METTL3
GAPDH
E
Lung density (g/ml)
F
BLM
α-SMA
G
α-SMA Positive Area (%)
H
BLM
YTHDC1
p21
SPC
Merge
K
p16
I
Positive rate of p21 in SPC positive cells (%)
J
Positive rate of p21 in SPC negative cells (%)
L
Positive rate of p16 in SPC positive cells (%)
M
Positive rate of p16 in SPC negative cells (%)
ns

Figure EV2. YTHDC1 counteracts stress-induced pulmonary epithelial cell senescence independent of its m6A binding activity.

(**A**) RT-qPCR analysis of YTHDC1, p21 and p16 mRNA level in the L2 cells overexpressed with Vector, YTHDC1-WT or YTHDC1-MUT. Forty-eight hours after transfection, cells were treated with BLM for 4 days and subjected to RT-qPCR. ($n = 3$). (**B**) IF detection of Ki67 in L2 cells overexpressed with Vector, YTHDC1-WT or YTHDC1-MUT. Cells were treated with BLM for 4 days. Scale bar: 10 μm. (**C**) Quantification of **B**. The percentage of Ki67 positive cells was calculated ($n \geq 100$ cells × three repeats). (**D**) Immunoblot analysis of YTHDC1 and METTL3 in L2 cells transfected with indicated siRNAs. Forty-eight hours after transfection, cells were treated with BLM for 4 days prior to analysis. ($n = 3$). (**E**) Quantification of mice lung density. C57/BL6 mice transfected with indicated AAV vectors were treated with BLM for 7 days ($n \geq 4$ per group). (**F**) Representative images of α-SMA IHC in the mice lungs from panel **E**. ($n \geq 4$ per group). Scale bar: 50 μm. (**G**) Quantification of panel **F**. The percentage of α-SMA positive cells was calculated. (**H**) Immunofluorescence was performed to determine the localization of YTHDC1 (red), p21 (purple) and SPC (green) in mice lungs from panel **E**. ($n \geq 4$ per group). Scale bar: 20 μm. (**I**) Quantification of panel **H**. The percentage of p21 positive cells in SPC positive cells was calculated ($n \geq 4$ per group). (**J**) Quantification of panel **H**. The percentage of p21 positive cells in SPC negative cells was calculated ($n \geq 4$ per group). (**K**–**M**) as in panels **H**–**J** except using p16 antibody to perform the IF. ($n \geq 4$ per group). Scale bar: 20 μm. Data information: All values are mean ± SEM. The One-way ANOVA was used to determine the statistical significance (**$P < 0.01$, ***$P < 0.001$, ****$P < 0.0001$). n = number of biological replicates except. Source data are available online for this figure.

© The Author(s)

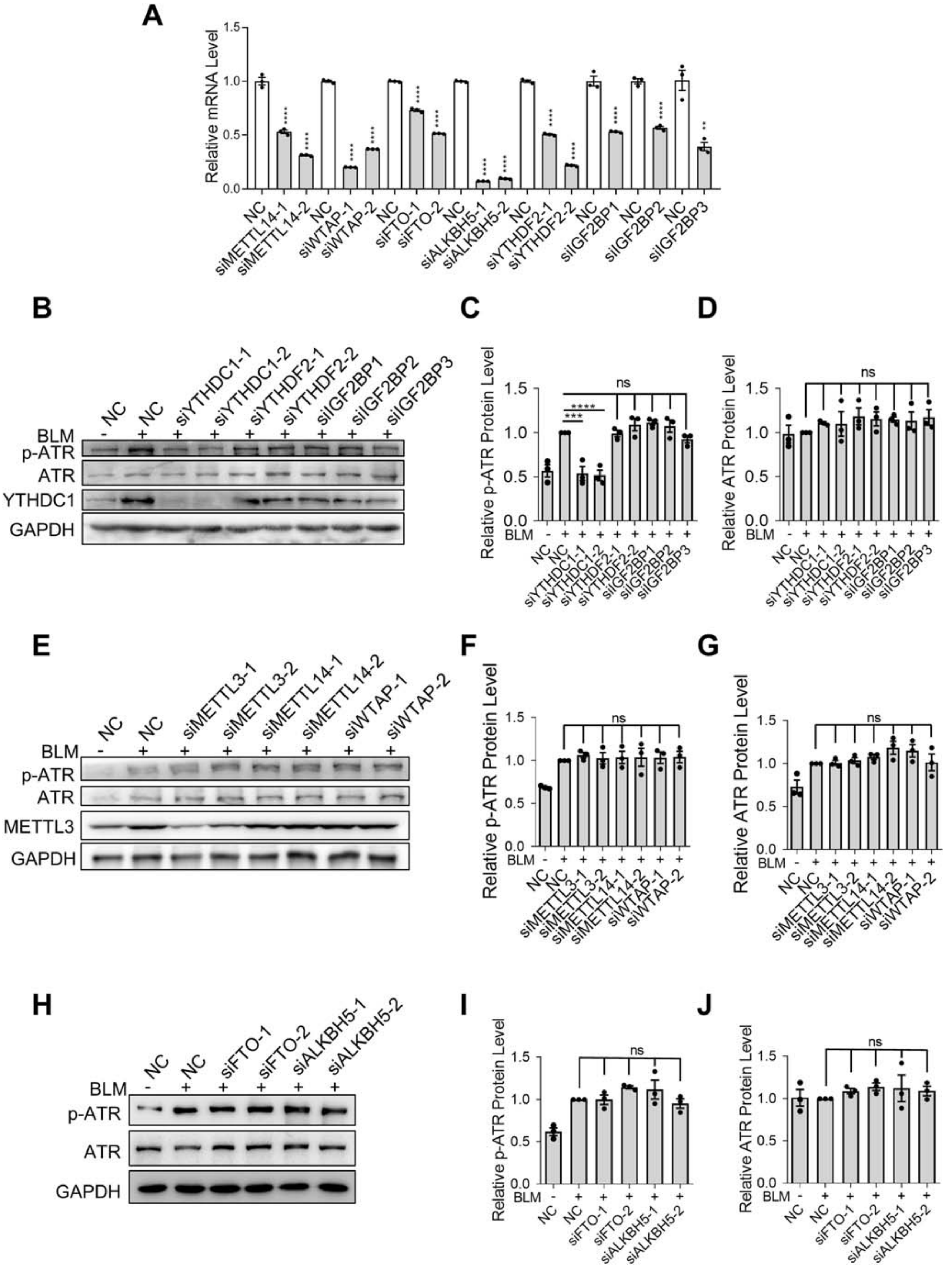
A
Relative mRNA Level
NC
siMETTL14-1
siMETTL14-2
siWTAP-1
siWTAP-2
siFTO-1
siFTO-2
siALKBH5-1
siALKBH5-2
siYTHDF2-1
siYTHDF2-2
siIGF2BP1
siIGF2BP2
siIGF2BP3
B
NC
siYTHDC1-1
siYTHDC1-2
siYTHDF2-1
siYTHDF2-2
siIGF2BP1
siIGF2BP2
siIGF2BP3
BLM
p-ATR
ATR
YTHDC1
GAPDH
C
Relative p-ATR Protein Level
ns
****
***
D
Relative ATR Protein Level
ns
E
siMETTL3-1
siMETTL3-2
siMETTL14-1
siMETTL14-2
siWTAP-1
siWTAP-2
METTL3
F
G
H
siFTO-1
siFTO-2
siALKBH5-1
siALKBH5-2
I
J

Figure EV3. YTHDC1 regulates the activation of ATR.

(**A**) RT-qPCR analysis of METTL14, WTAP, FTO, ALKBH5, YTHDF2, IGF2BP1, IGF2BP2 and IGF2BP3 mRNA level in A549 cells transfected with indicated siRNAs. ($n = 3$). (**B**) Immunoblot analysis of activated ATR, total ATR and YTHDC1 in A549 cells transfected with indicated siRNAs. A549 cells were treated with BLM or saline for 4 h prior to analysis. (**C**,**D**), Quantification of panel **B**. The relative protein p-ATR or ATR levels were determined by normalizing the intensities of p-ATR or ATR to the intensity of GAPDH. ($n = 3$). (**E**) Immunoblot analysis of activated ATR, total ATR and METTL3 in A549 cells transfected with indicated siRNAs. A549 cells were treated with BLM or saline for 4 h prior to analysis. (**F**,**G**) Quantification of panel **E**. The relative protein p-ATR or ATR levels were determined by normalizing the intensities of p-ATR or ATR to the intensity of GAPDH. ($n = 3$). (**H**) Immunoblot analysis of activated ATR and total ATR in A549 cells transfected with indicated siRNAs. A549 cells were treated with BLM or saline for 4 h prior to analysis. (**I**,**J**), Quantification of panel **H**. The relative protein p-ATR or ATR levels were determined by normalizing the intensity of p-ATR or ATR to the intensities of GAPDH. ($n = 3$). Data information: All values are mean ± SEM. The One-way ANOVA was used to determine the statistical significance (**$P < 0.01$, ***$P < 0.001$, ****$P < 0.0001$). n = number of biological replicates. Source data are available online for this figure.

© The Author(s)

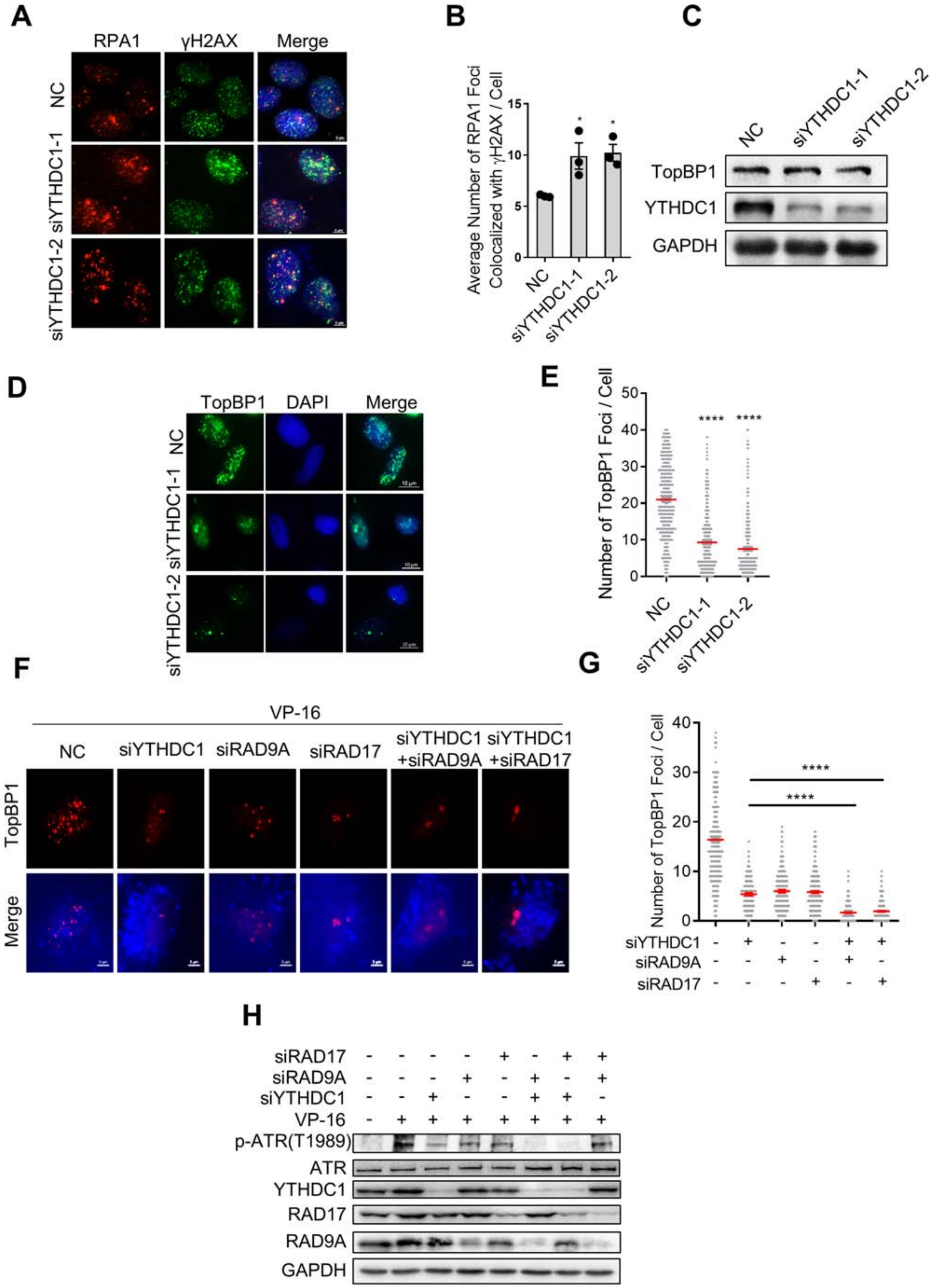
A
RPA1
γH2AX
Merge
NC
siYTHDC1-1
siYTHDC1-2
B
Average Number of RPA1 Foci Colocalized with γH2AX / Cell
NC
siYTHDC1-1
siYTHDC1-2
C
NC
siYTHDC1-1
siYTHDC1-2
TopBP1
YTHDC1
GAPDH
D
TopBP1
DAPI
Merge
NC
siYTHDC1-1
siYTHDC1-2
E
Number of TopBP1 Foci / Cell
NC
siYTHDC1-1
siYTHDC1-2
F
VP-16
NC
siYTHDC1
siRAD9A
siRAD17
siYTHDC1 +siRAD9A
siYTHDC1 +siRAD17
TopBP1
Merge
G
Number of TopBP1 Foci / Cell
siYTHDC1
siRAD9A
siRAD17
H
siRAD17
siRAD9A
siYTHDC1
VP-16
p-ATR(T1989)
ATR
YTHDC1
RAD17
RAD9A
GAPDH

Figure EV4. YTHDC1 regulates the recruitment of TopBP1 to activate ATR independent of 9-1-1 complex.

(**A**) IF detection of RPA1 and γH2AX foci in control or YTHDC1-depleted A549 cells. Cells were treated with VP-16 for 24 h. Scale bar: 5 μm. (**B**) Quantification of **A**. The average number of RPA1 foci colocalized with γH2AX foci per cell ($n \geq 100$ cells × three repeats). (**C**) Immunoblot analysis of TopBP1 and YTHDC1 in A549 cells transfected with NC, siYTHDC1-1 or siYTHDC1-2. A549 cells were treated with VP-16 for 24 h prior to analysis ($n = 3$). (**D**) Immunofluorescence (IF) detection of TopBP1 foci in control or YTHDC1-depleted A549 cells. Cells were treated with BLM for 4 h before detection. Scale bar: 5 μm. (**E**) Quantification of **D**. The number of TopBP1 foci per cell (n ≥ 100 cells × three repeats). (**F**) IF detection of TopBP1 foci in A549 cells transfected with indicated siRNAs. Cells were treated with VP-16 for 24 h. Scale bar: 5 μm. (**G**) Quantification of **F**. The number of TopBP1 foci per cell ($n \geq 100$ cells × three repeats). (**H**) Immunoblot analysis of activated ATR, total ATR, YTHDC1, RAD17, and RAD9A in A549 cells transfected with indicated siRNAs. Cells were treated with VP-16 or DMSO for 24 h prior to analysis. ($n = 3$). Data information: All values are mean ± SEM. The One-way ANOVA was used to determine the statistical significance (*$P < 0.05$, **$P < 0.01$, ****$P < 0.0001$). $n =$ number of biological replicates. Source data are available online for this figure.

 © The Author(s)

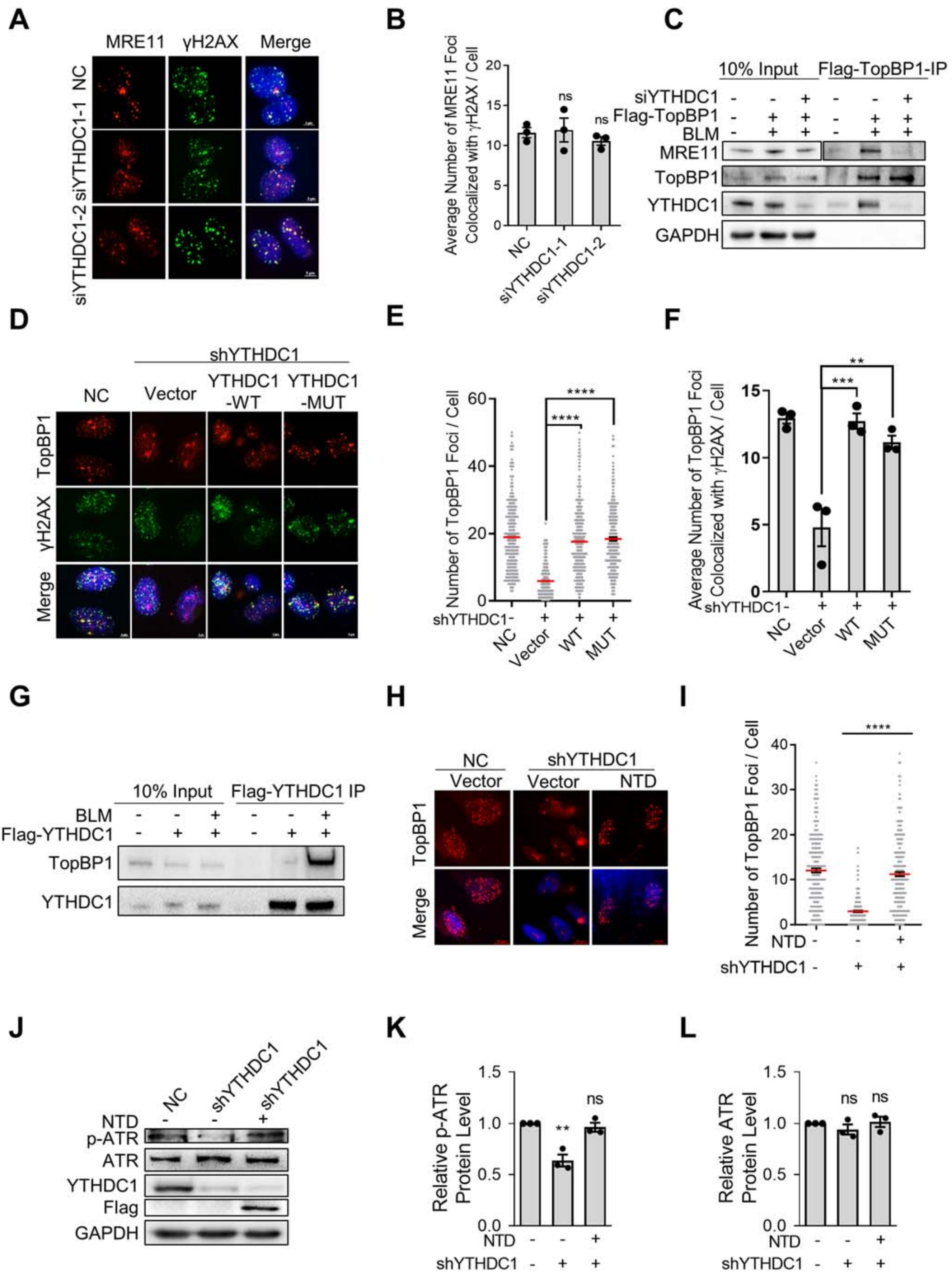
A
MRE11
γH2AX
Merge
NC
siYTHDC1-1
siYTHDC1-2
B
Average Number of MRE11 Foci Colocalized with γH2AX / Cell
ns
ns
NC
siYTHDC1-1
siYTHDC1-2
C
10% Input
Flag-TopBP1-IP
siYTHDC1
Flag-TopBP1
BLM
MRE11
TopBP1
YTHDC1
GAPDH
D
shYTHDC1
NC
Vector
YTHDC1 -WT
YTHDC1 -MUT
TopBP1
γH2AX
Merge
E
Number of TopBP1 Foci / Cell
****
****
shYTHDC1
NC
Vector
WT
MUT
F
Average Number of TopBP1 Foci Colocalized with γH2AX / Cell
**
***
shYTHDC1
NC
Vector
WT
MUT
G
10% Input
Flag-YTHDC1 IP
BLM
Flag-YTHDC1
TopBP1
YTHDC1
H
NC
shYTHDC1
Vector
Vector
NTD
TopBP1
Merge
I
Number of TopBP1 Foci / Cell
****
NTD
shYTHDC1
J
NC
shYTHDC1
shYTHDC1
NTD
p-ATR
ATR
YTHDC1
Flag
GAPDH
K
Relative p-ATR Protein Level
**
ns
NTD
shYTHDC1
L
Relative ATR Protein Level
ns
ns
NTD
shYTHDC1

Figure EV5. YTHDC1 regulates the recruitment of TopBP1 independent of its m6A binding activity.

(**A**) IF detection of MRE11 and γH2AX foci in control or YTHDC1-depleted A549 cells. Cells were treated with VP-16 for 24 h. Scale bar: 5 μm. (**B**) Quantification of **A**. The average number of MRE11 foci colocalized with γH2AX foci per cell ($n \geq 100$ cells × three repeats). (**C**) Co-IP assay to determine the interaction of TopBP1 with MRE11 and YTHDC1 in HEK293T cells. YTHDC1 depleted cells were transfected with Flag-TopBP1 or Vector and treated with BLM for 4 h. Cell lysates were used for immunoprecipitation with Flag-beads. Immunoprecipitates were immunoblotted with MRE11, TopBP1, YTHDC1 and GAPDH antibody, respectively. ($n = 3$). (**D**) IF detection of TopBP1 and γH2AX foci in YTHDC1-depleted A549 cells overexpressed with Vector, YTHDC1-WT or YTHDC1-MUT. Cells were treated with VP-16 for 24 h. Scale bar: 5 μm. (**E**) Quantification of **D**. The number of TopBP1 foci per cell (n ≥ 100 cells × three repeats). (**F**), Quantification of **D**. The average number of TopBP1 foci colocalized with γH2AX foci per cell ($n \geq 100$ cells × three repeats). (**G**) Co-IP assay to determine the interaction between YTHDC1 and TopBP1. HEK293T cells transfected with Flag-YTHDC1 were treated with BLM for 4 h. Cell lysates were used for immunoprecipitation with Flag-beads. Immunoprecipitates were immunoblotted with TopBP1 and YTHDC1 antibody, respectively. ($n = 3$). (**H**) IF detection of TopBP1 foci in YTHDC1 defect A549 cells overexpressed Vector or NTD of YTHDC1. Forty-eight hours after transfection, cells were treated with VP-16 for 24 h. Scale bar: 5 μm. (**I**) Quantification of **H**. The number of TopBP1 foci per cell ($n \geq 100$ cells × three repeats). (**J**) Immunoblot analysis of activated ATR, total ATR, YTHDC1 and Flag from panel **H**. (**K**,**L**) Quantification of panel **J**. The relative protein p-ATR or ATR levels were determined by normalizing the intensities of p-ATR or ATR to the intensity of GAPDH. ($n = 3$). Data information: All values are mean ± SEM. The unpaired Student's two-tailed *t*-test was used to determine the statistical significance between two groups. The One-way ANOVA was used to determine the statistical significance for more than two groups (**$P < 0.01$, ***$P < 0.001$, ****$P < 0.0001$). n = number of biological replicates. Source data are available online for this figure.

 © The Author(s)
